# Selenoxanthylium Salts—Organic Photocatalysts for Low-Energy Photoredox Catalysis

**DOI:** 10.1021/jacs.6c13607

**Published:** 2026-08-10

**Authors:** Suwanan Uipanit, Till Schreyer, Kirill Zhiliaev, Jan-Hendrik Kaup, Mirco Natali, Javier Mateos

**Affiliations:** † Institute of Organic Chemistry, 27258 University of Vienna, Vienna 1090, Austria; ‡ Unit for Advanced Magnetic Resonance (AMR), Department of Chemistry and Center of Excellence for Innovation in Chemistry, Faculty of Science, Kasetsart University, 50 Ladyao Road, Chatuchak District, Bangkok 10900, Thailand; § Department of Chemical, Pharmaceutical and Agricultural Sciences (DOCPAS), University of Ferrara, Via L. Borsari 46, Ferrara 44121, Italy

## Abstract

The design of low energy organic photoredox catalysts relies predominantly on π-extended frameworks. Incorporation of selenium into the xanthylium core shifts absorption toward the red region relative to lighter chalcogens (O and S), enabling catalysis with 650 nm light-sources. Selenoxanthylium salts are low-energy-absorbing organic photocatalysts with defined excited-state topology, distinguishing locally excited and charge-transfer states. Incorporation of a heavier chalcogen constitutes a design principle complementary to extended π-frameworks commonly used in organic photocatalysts. Under red-light irradiation, the catalysts engage in reductive quenching and operate on up to 5.0 mmol scale. Catalytic activity is demonstrated across multiple transformations, including [4 + 2] and [2 + 2] cycloadditions, C–H aminations, boronic acid oxidations, aza-Henry reactions, and trifluoromethylations.

## Graphical abstract

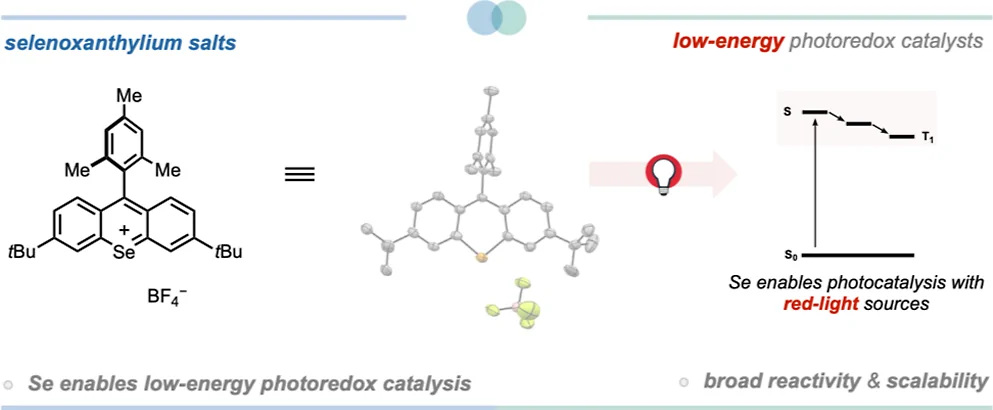

## Introduction

Photocatalysis aims to convert photon energy into chemical reactivity, ideally with minimal energy loss from absorbed photons.^1^ The fundamental requirements of photocatalysts are (i) balanced ground- and excited-state redox potentials that enable substrate oxidation or reduction, (ii) sufficiently long excited-state lifetimes—spanning from nanoseconds (10^–9^ s) to milliseconds (10^–3^ s)—that allow productive single-electron or energy-transfer events,^2^ and (iii) absorption in the visible region (λ_exc_ > 400 nm)^3–5^ to minimize ultraviolet-induced side reactions, and ideally extending to the red-light region (λ_exc_ > 600 nm)^6,7^ to enable lower-energy excitation.^8^ Both transition-metal-based and organic photocatalysts are available, with distinct design elements.^9,10^ Systematic studies on redox properties and excited-state lifetimes have been developed.^11–13^ Yet, the portfolio of red-light-absorbing photocatalysts remains limited, particularly in organic systems.^14^

Red-light-absorbing photocatalysts have attracted increasing interest as low-energy alternatives (600 nm = 47.7 kcal·mol^–1^) to commonly used blue-light-absorbing chromophores.^15–19^ Advantages include improved penetration of the reaction medium and reduced Rayleigh scattering,^14^ which may benefit reaction scale-up.^20,21^ Organic compounds used as low-photon-energy-absorbing photocatalysts rely on strongly delocalized π frameworks to achieve red-shifted absorption. Representative examples include cyanine dyes, porphyrins, trianguleniums and helical carbenium ions (Figure 1A).^22–27^ The versatility of helical carbenium photocatalysts in promoting a range of transformations under red-light irradiation is well established.^24,28–34^ Absorption bands centered around 600 nm and relatively long singlet excited-state lifetimes (S_0_ → S_1_, τ = 5.5–19.1 ns) enable photocatalysis. Helical carbenium photocatalysts operate through both reductive and oxidative quenching pathways.^35^

**Figure 1:**
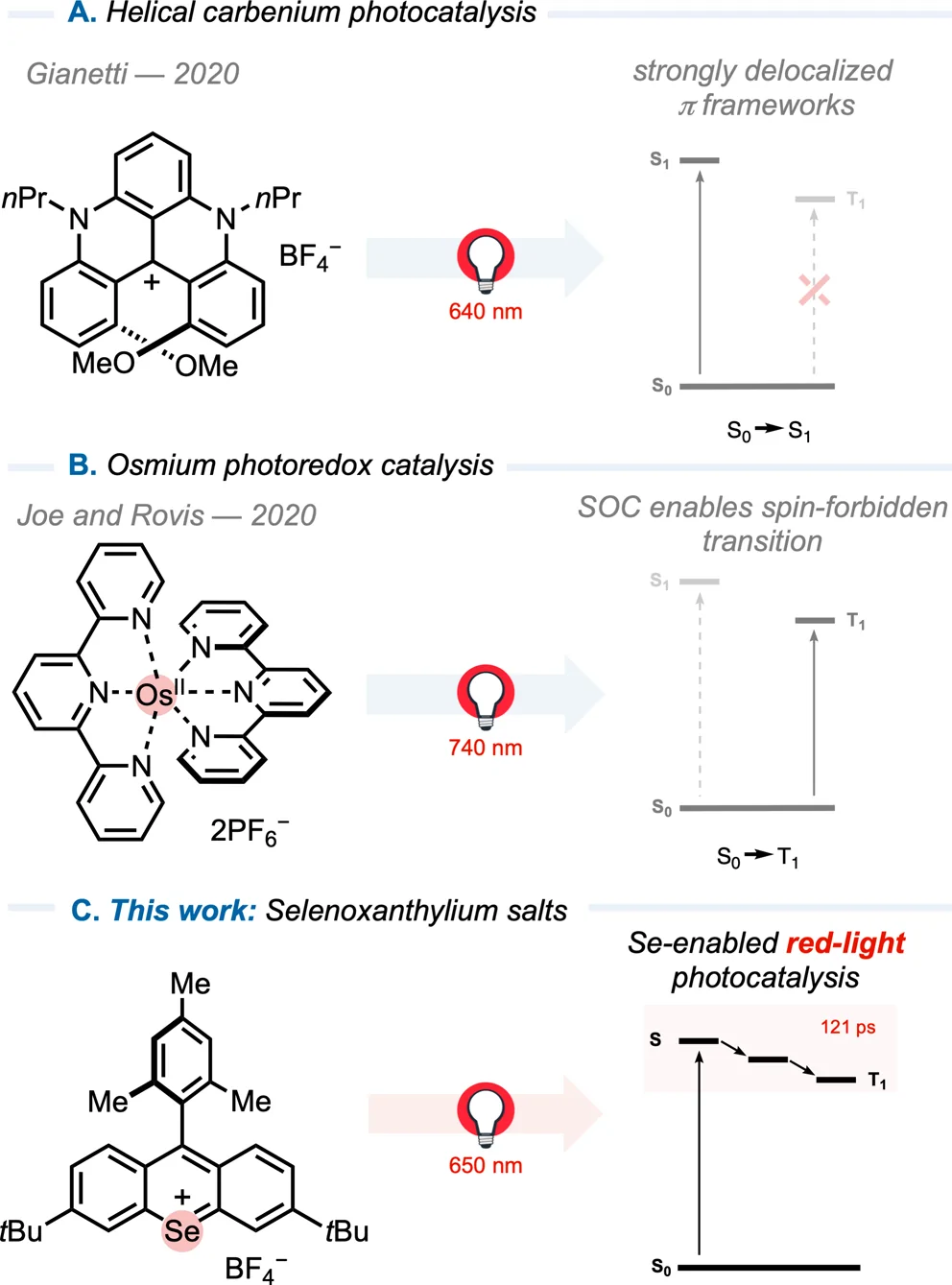
(A). Red-light organophotoredox catalysts based on delocalized π-frameworks. (B). Near-IR organometallic photoredox catalysis enabled by spin–orbit coupling (SOC). (C). This work: selenoxanthylium salts as low-energy-absorbing photocatalysts.

Incorporation of rigid π-conjugated bipyridyl ligands into transition-metal photocatalysts has been exploited to achieve red-shifted absorption.^36^ In contrast, incorporation of heavy atoms represents an additional photocatalyst design principle (Figure 1B).^37,38^ Strong spin–orbit coupling (SOC) reduces energy loss through excitation of formally spin-forbidden transitions (S_0_ → T_1_) .^11,21,39,40^ Exploitation of enhanced SOC in catalysis has been extended to osmium-, iridium- and ruthenium-based photocatalysts.^40,41^ Translation of such design principles to organic photocatalysis has not been yet implemented.

The synthesis, photophysical characterization, and catalytic properties of selenoxanthylium salts are reported. Established principles in acridinium^42,43^ and xanthylium^44–47^ photochemistry, combined with the heavy-atom effect of selenium,^48^ enable access to organic photocatalysts that operate under red-light irradiation. In contrast to delocalized π-systems that rely on extended conjugation, incorporation of selenium represents an additional design strategy for low-energy organic photocatalysts. No direct excitation of a formally spin-forbidden S_0_ → T_1_ transition was observed. Instead, reactive triplet states are accessed through conventional singlet excitation followed by intersystem crossing. Heavy-atom design principles widely exploited in transition-metal photocatalysis can thus be extended to purely organic scaffolds for the development of low-energy-absorbing photocatalysts. Selenoxanthylium salts display red-shifted absorption, tunable excited-state topology, and access to reactive triplet states via fast intersystem crossing (ISC) under 650 nm irradiation. The resulting photocatalysts operate in electron-transfer manifolds and enable red-light-driven transformations across multiple reaction classes, including [4 + 2] cycloadditions on up to 5.0 mmol scale.

## Results and Discussion

### Synthesis and Photocatalyst Design

The design of selenoxanthylium photocatalysts integrates: (i) established photophysical and photocatalytic principles of acridinium and xanthylium salts, and (ii) increased heavy-atom effects along the chalcogen series (O/S/Se) (Figure 2A).^49,50^ Initial studies on the photostability of acridinium salts identified the mesityl group at the 9-position as both an electron donor and a protecting group against radical addition at this site.^49^ Further structural refinement of acridinium photocatalysts demonstrated that incorporation of *tert*-butyl groups at the 3- and 6-positions (**[1a]­BF**_**4**_) increases stability toward nucleophile and radical addition leading to catalyst deactivation.^51^ The N-to-O comparison has been achieved to access photostable xanthylium salts as stronger photooxidants (E*_red_ of **[2a]­BF**_**4**_ = 2.51 V vs SCE vs E*_red_ of **[1a]­BF**_**4**_ = 2.10 V vs SCE).^52^ Thioxanthylium salts have been employed as photocatalysts, typically bearing electron-donating substituents on the core, establishing the O-to-S comparison.^53,54^ In contrast, the O-to-Se comparison remains unexplored (**[4a]­BF**_**4**_). Access to selenoxanthylium salts enables evaluation of influence of the chalcogen on the photocatalyst scaffold in both ground and excited states, including the role of heavy atom incorporation.

**Figure 2:**
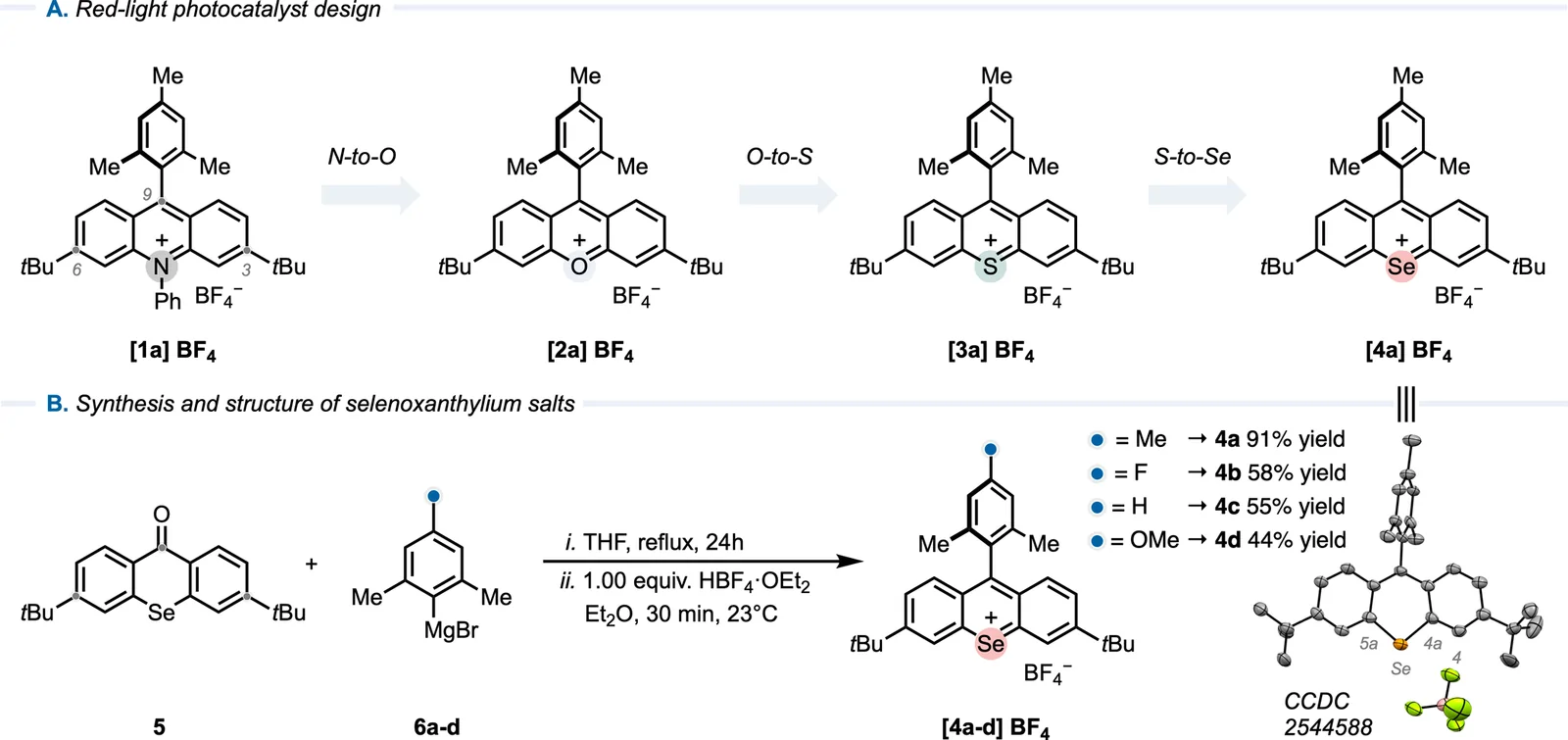
(A). Design of selenoxanthylium photocatalysts from acridinium and xanthylium frameworks. (B). Synthesis of selenoxanthylium salts **[4a**–**d]­BF**_**4**_ and solid-state structure of **[4a]­BF**_**4**_ determined by X-ray diffraction (50% probability ellipsoids; H atoms omitted).

Selenoxanthylium salts **[4a-d]­BF**_**4**_ are prepared through a scalable four-step sequence (Figure 2B and Supporting Information, pages S25–S32). Addition of aryl Grignard reagents **6a**–**d** to selenoxanthone **5**, followed by in situ dehydration of the resulting 9-arylselenoxanthol, affords **[4a**–**d]­BF**_**4**_ in 44–91% yield (Figure 2B). The synthetic route enables variation of both the chalcogen atom and the aryl substituent at the 9-position. Electron-poor (**[4b]­BF**_**4**_), neutral (**[4c]­BF**_**4**_), and electron-rich (**[4d]­BF**_**4**_) derivatives were prepared. The parent compound **[4a]­BF**_**4**_ was obtained in 91% yield on 0.85 mmol scale, providing 600 mg of selenoxanthylium salt.

Ground-state photocatalyst stability was evaluated (see Supporting Information, Pages S115–S121). In the presence of primary amines, the oxygen atom of **[2a]­BF**_**4**_ undergoes nucleophilic substitution to afford **[1a]­BF**_**4**_.^55,56^ The O-to-N transformation of **[2a]­BF**_**4**_ in the presence of aniline was monitored by ^1^H NMR spectroscopy. After 16 h, **[2a]­BF**_**4**_ is converted to acridinium **[1a]­BF**_**4**_ with 26% conversion; 64% conversion is observed after 96 h. In contrast, Se-to-N substitution is minimal: after 16 h, only traces of **[1a]­BF**_**4**_ are detected, and no substantial changes are observed after 96 h. Ground-state stability is therefore increased relative to oxygen-based xanthylium photocatalysts.

Single-crystal X-ray diffraction confirmed the molecular structure of photocatalyst **[4a]­BF**_**4**_ (Figure 2B). The solid-state structure shows an in-plane selenium atom (∠C4–C4a–Se–C5a = 178.1°), consistent with conjugation across the aromatic core.

### Photophysical Characterization

Chalcogen conjugation with the xanthylium ring influences its electronic structure. Chalcogen substitution therefore affects the absorption spectra across the O/S/Se series (Figure 3A). TD-DFT calculations reproduce the experimental trends and provide insight into the nature of the electronic transitions (Figure 3B,C and Supporting Information, pages S166–S183). For xanthylium **[2a]­BF**_**4**_, the absorption band at 394 nm arises from a locally excited (**LE**) π–π* transition dominated by the HOMO −3 → LUMO excitation localized on the xanthylium core (Figure 3Bi). A lower-lying charge-transfer (**CT**) state, corresponding to electron transfer from the mesityl donor to the xanthylium acceptor, lies at 2.300 eV (Figure 3Bii). Replacement of oxygen by sulfur and selenium has only a modest effect on the **LE** band centered near 400 nm (403 nm for **[3a]­BF**_**4**_, and 414 nm for **[4a]­BF**_**4**_). In contrast, selenoxanthylium **[4a]­BF**_**4**_ exhibits an additional low-energy absorption band at 560 nm arising from a π–π* transition localized on the xanthylium core (Figure 3Ci). The associated CT state remains nearly isoenergetic with the LE state (Δ*E*_LE‑CT_ = 0.223 eV, Figure 3Cii). Selenium substitution therefore extends the absorption profile to 560 nm without substantially perturbing the low-lying **CT** state.

**Figure 3:**
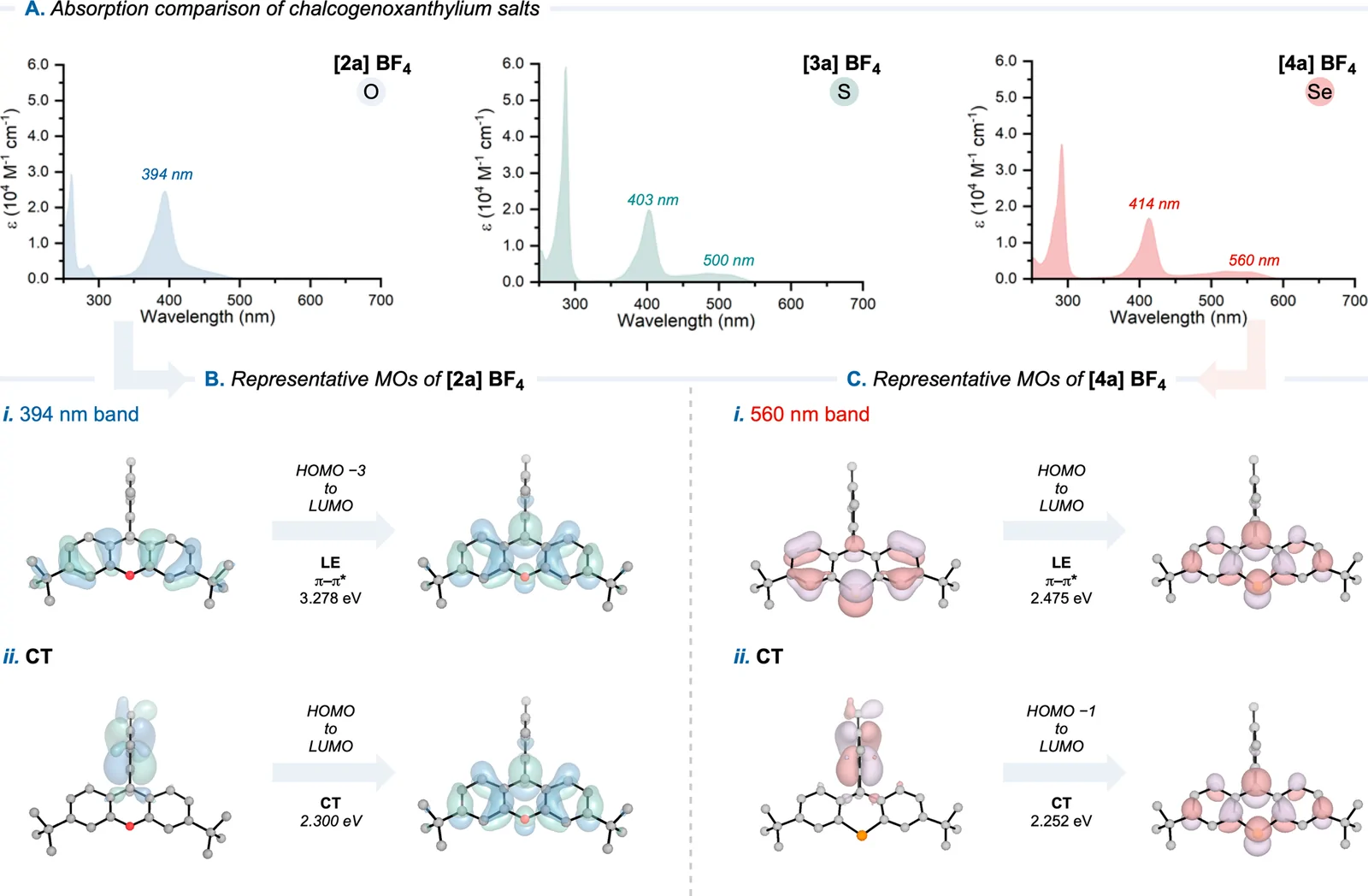
(A). Absorption spectra of chalcogenoxanthylium salts: xanthylium **[2a]­BF**_**4**_ (left), thioxanthylium **[3a]­BF**_**4**_ (middle), and selenoxanthylium **[4a]­BF**_**4**_ (right). (B). Representative molecular orbitals involved in the electronic transitions of **[2a]­BF**_**4**_ calculated at the TPSSh-D4/X2C-TZVPall/CPCM­(SMD, MeCN) level, illustrating the bright **LE** (π–π*) (i) and the low-energy **CT** excitation (ii). (C). Representative molecular orbitals involved in the dominant electronic transitions of **[4a]­BF**_**4**_ illustrating the bright **LE** (i) and the low-energy **CT** excitation (ii). (H atoms omitted).

Selective excitation monitored by ultrafast spectroscopy enables interpretation of the excited-state topology of chalcogenoxanthylium salts (Figure 4A,B).^57^ Excitation at 343 nm of xanthylium salt **[2a]­BF**_**4**_ in MeCN delivers a transient signal with absorption at ca. 500 nm, characteristic of the mesityl radical cation,^58^ which evolves monotonically to a transient species with maxima at 550 and 850 nm. The excited state dynamics indicate ultrafast population of a **CT** state, from which ISC to a triplet locally excited state (**LE** ^**T**^) occurs within 495 ps. The observation is consistent with the twisted intramolecular charge-transfer state proposed by Nicewicz and co-workers.^50^

**Figure 4:**
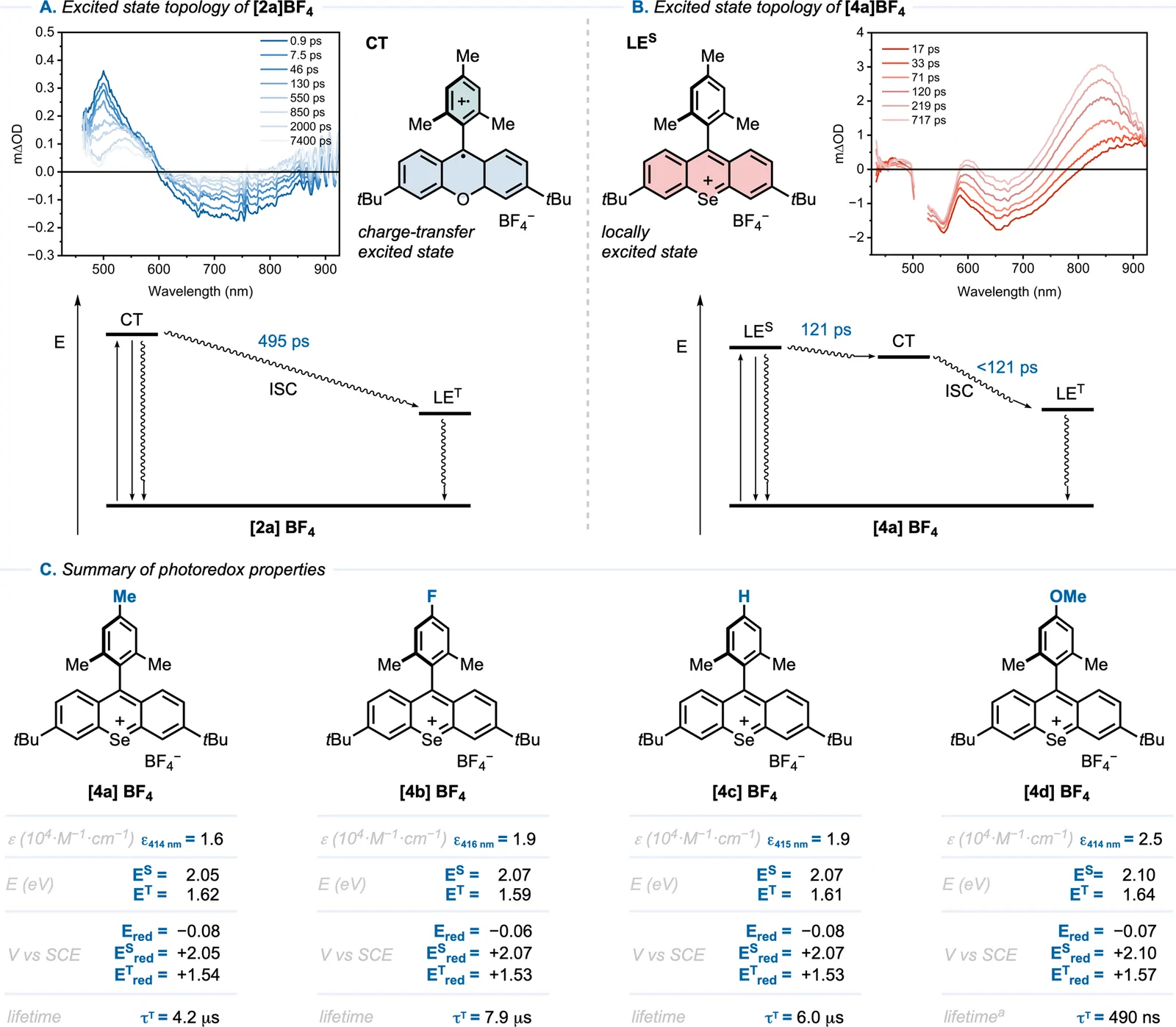
(A). Ultrafast spectroscopy of **[2a]­BF**_**4**_: top, TAS upon excitation at 343 nm in deoxygenated MeCN; bottom, excited-state energy diagram. (B). Ultrafast spectroscopy of **[4a]­BF**_**4**_: top, TAS upon excitation at 515 nm in deoxygenated MeCN; bottom, excited-state energy diagram. (C). Summary of the photophysical and photoredox properties of selenoxanthylium photocatalysts, including molar absorption coefficients, optical gaps (***E*** ^**S**^), triplet-state energies (***E* ^T^**), ground- and excited-state reduction potentials, and triplet lifetimes measured in acetonitrile. (a) Measured in CH_2_Cl_2_.

Selenoxanthylium salt **[4a]­BF**_**4**_ in MeCN displays a distinct excited-state topology that resembles that of Fukuzumi-type photocatalyst (Figure 4B).^42,43,49^ Excitation at 515 nm yields a transient spectrum characterized by bleaching of the ground-state absorption, stimulated emission, and an excited-state absorption band centered at 900 nm, assigned to the singlet locally excited state (**LE** ^**S**^). Following subtle ultrafast changes attributed to vibrational cooling (Figures S69 and S70), the spectrum evolves into a new transient signal with a maximum at 850 nm, characteristic of the corresponding triplet excited state (**LE** ^**T**^), as confirmed by laser flash photolysis (Figure S77). The **LE** ^**S**^-to-**LE** ^**T**^ process occurs with a time constant of 121 ps.

Similar spectral changes, associated with ISC, are observed for the 4-fluoroxylyl and 2,6-dimethylphenyl derivatives **[4b]­BF**_**4**_ and **[4c]­BF**_**4**_ (Figures S71 and S73, respectively), albeit with slower kinetics (ca. 1.2 ns). The aryl substituent at the 9-position modulates the energy of the CT state. For **[4b]­BF**_**4**_ and **[4c]­BF**_**4**_, the LE^S^ → CT process is anticipated to be endergonic. In contrast, the faster kinetics observed in **[4a]­BF**_**4**_ can be rationalized by a stepwise LE^S^ → CT → LE^T^ process. The 4-methoxyxylyl derivative **[4d]­BF**_**4**_ exhibits different excited state dynamics (Figure S75), with fast CT population (1 ps) followed by ground-state decay (16 ps) and no evidence of triplet state formation in acetonitrile.^57^ In CH_2_Cl_2_, ultrafast formation of a **CT** state (ca. 0.7 ps) is likewise observed (Figure S76). Unlike in acetonitrile, however, the CT state undergoes ISC to the **LE** ^**T**^ within 48 ps, highlighting the strong sensitivity of the excited-state topology to solvent polarity. Solvent polarity likewise affects the photocatalytic performance: catalyst **[4d]­BF**_**4**_ performs efficiently in 1,2-DCE and MeNO_2_ but underperforms in MeCN (vide infra).

Photophysical studies allowed us to contextualize the photoredox properties of the selenoxanthylium salts (Figure 4C). The ground-state reduction potentials remain nearly unchanged across the series (−0.08 to −0.06 V vs SCE), despite substantial differences in the excited-state lifetimes and intersystem crossing dynamics. Combined with the optical gaps (*E* ^S^ = 2.05–2.10 eV), these values give rise to strongly oxidizing singlet excited states (*E* ^S^_red_ = +2.05 to +2.10 V vs SCE) and triplet excited states (*E* ^T^_red_ = +1.54 to +1.57 V vs SCE).

### Photocatalytic Performance

Selective excitation with LEDs demonstrates the synthetic applicability of selenoxanthylium salts (Figure 5A). The [4 + 2] cycloaddition between styrene **7** and diene **8** was used to benchmark the reactivity of photocatalysts **1**–**4** under different light sources.^59^ No cyclohexene **9** was observed in the absence of irradiation (see Supporting Information, Tables S1–S4). Under near-isoabsorptive excitation conditions (405 nm), photocatalysts **[1**–**4a]** deliver **9** in >95% yield. Acridinium and xanthylium salts (**[1a]­BF**_**4**_ and **[2a]­BF**_**4**_) afford **9** under blue light (450 nm), whereas no product is detected under orange or red light. Thioxanthylium **[3a]­BF**_**4**_ furnishes **9** in 26% and 28% yield under green light (525 nm) and orange light (595 nm) and in 7% yield under red light (650 nm) after 1 h. Under 650 nm irradiation, selenoxanthylium salt **[4a]­BF**_**4**_ affords **9** in quantitative yield. Selective excitation of the absorption band centered at 560 nm, which extends to 620 nm at the reaction concentration (1.0 mol %, 1 mM; Figure S36), enables red-light photocatalysis. Mechanistic studies show that photocatalyst **[4a]­BF**_**4**_ operates as a triplet photocatalyst (see Supporting Information pages S111–S114) across all five wavelengths tested combining a high excited-state reduction potential (***E*** ^**T**^_**red**_ = +1.54 V vs SCE) and μs lifetime, enabling efficient reactivity under low-energy excitation.

**Figure 5:**
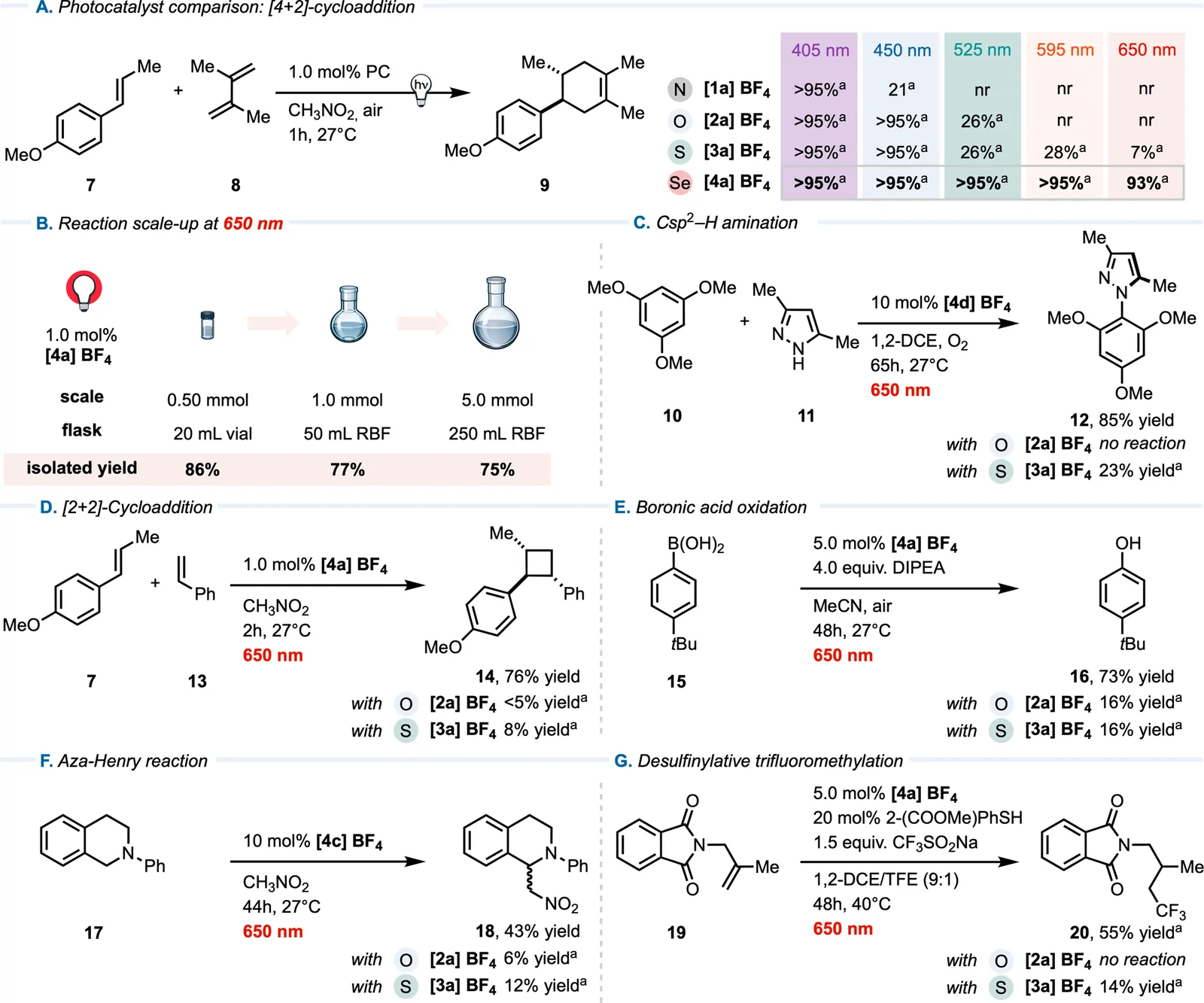
(A). Wavelength-dependent [4 + 2] cycloaddition using photocatalysts **[1a**–**4a]­BF**_**4**_ (405–650 nm). (B). Scalability of the [4 + 2] cycloaddition. (C). Red-light promoted Csp^2^–H amination. (D). Red-light promoted [2 + 2]-cycloaddition reaction. (E). Red-light promoted boronic acid oxidation. (F). Red-light promoted aza-Henry reaction with tetrahydroisoquinolines. (G). Red-light promoted desulfinylative trifluoromethylation of alkenes. Performance of selenoxanthylium photocatalysts **[4a-d]­BF**_**4**_ in C–H amination. ^a^Yield determined by ^1^H NMR spectroscopy using CH_2_Br_2_ as internal standard at 0.1 mmol scale. The Supporting Information provides detailed experimental procedures.

Red-light penetration in reaction media exceeds that of blue light.^6,7,14,20^ Increased penetration enables direct scale-up of photoredox reactions. The [4 + 2] cycloaddition enables comparison of reaction scalability with selenoxanthylium **[4a]­BF**_**4**_ under 1 h irradiation at 650 nm (Figure 5B). A 50fold scale-up of the transformation is achieved (75% yield at 5.0 mmol). Selenoxanthylium salts under red-light irradiation remain efficient across different reactor geometries, including scintillation vials and round-bottom flasks.

The selenoxanthylium series **[4a**–**d]** was also benchmarked. Minimal differences are observed in the redox properties of the photocatalysts. Photocatalyzed C–H amination of 1,3,5-trimethoxybenzene (**10**) was used to assess reactivity (Figure 5C).^51^ All four selenoxanthylium salts catalyze the transformation (from 62% yield with **[4c]­BF**_**4**_ to 92% yield with **[4d]­BF**_**4**_, Table S5). The methoxy-substituted catalyst **[4d]­BF**_**4**_ affords **12** in 87% yield on 1.0 mmol scale.

The photocatalyst platform operates across transformations previously achieved with acridinium or red-light photocatalysts. Selenoxanthylium salts **[4a**–**d]­BF**_**4**_ promote [2 + 2] cycloadditions (Figure 5D, 90% yield, >20:1 dr),^60^ boronic acid oxidations (Figure 5E, 73% yield),^24^ aza-Henry reactions (Figure 5F, 65% yield),^61^ and desulfinylative trifluoromethylations of alkenes (Figure 5G, 63% yield).^62^ Multiple solvents (CH_3_NO_2_, DMF, TFE, 1,2-DCE and CH_2_Cl_2_) are compatible. Reactions proceed under inert atmosphere (Figure 5G) or in air (Figure 5F). Across all benchmark reactions, the selenoxanthylium photocatalysts outperform the corresponding xanthylium and thioxanthylium analogues under identical reaction conditions (see Supporting Information pages S125–S163). These results establish selenoxanthylium salts as a general platform for low energy photocatalysis.

## Conclusions

In conclusion, a low energy photoredox catalysis platform based on the selenoxanthylium scaffold has been developed. Integration of established acridinium photocatalyst design with heavy-atom effects enables access to organic photocatalysts that operate under 650 nm irradiation. Photophysical analysis defines the excited-state topology and establishes charge-transfer and triplet locally excited states. Selenoxanthylium salts combine red-shifted absorption, enhanced ground-state stability, and tunable excited-state dynamics. The photocatalysts operate multiple photocatalyzed transformations and enable scalable reactions, including [4 + 2] cycloadditions on up to 5.0 mmol scale. C­(sp^2^)–H aminations, [2 + 2] cycloadditions, boronic acid oxidations, aza-Henry reactions, and desulfinylative trifluoromethylations of alkenes were performed. These results establish selenoxanthylium salts as a class of low energy photocatalysts and show that, in analogy to transition-metal complexes, heavy-atom incorporation complements π-extension as a design element for red-light-active organic photocatalysts.

## Supplementary Material



## Notes

Additional experimental details, materials, methods, crystallographic details, theoretical calculations, and characterization data (^1^H, ^13^C, ^19^F and ^77^Se NMR, and HRMS) for all new compounds (PDF)

## Abbreviations

SCE: saturated calomel electrode
SOC: spin–orbit coupling
NMR: nuclear magnetic resonance
TD-DFT: time dependent density functional theory
TAS: transient absorption spectroscopy
LE: locally excited
CT: charge-transfer
ISC: intersystem crossing
LED: light-emitting diode
dr: diastereomeric ratio
DMF: dimethylformamide
TFE: trifluoroethanol
1,2-DCE: 1,2-dichloroethane.
