# Supplementary material for: Selenoxanthylium Salts—Organic Photocatalysts for Low-Energy Photoredox Catalysis
Source: J Am Chem Soc. 2026 Aug 10;148(32):35139–46. doi: 10.1021/jacs.6c13607 (PMC13495763; doi:10.1021/jacs.6c13607)
Supplement: Supplementary file 1 [file ja6c13607_si_001.pdf]

SUPPORTING INFORMATION

Selenoxanthylium Salts – Organic Photocatalysts for Low-Energy Photoredox Catalysis

Suwanan Uipanit,‡ [a,b] Till Schreyer,‡ [a] Kirill Zhiliaev,[a] Jan-Hendrik Kaup,[a] Mirco Natali,[c] Javier Mateos [a] *

[a.] Institute of Organic Chemistry, University of Vienna, Vienna 1090, Austria

[b]Unit for Advanced Magnetic Resonance (AMR), Department of Chemistry and Center of Excellence for Innovation in Chemistry, Faculty of Science, Kasetsart University, 50 Ladyao Road, Chatuchak District, Bangkok, 10900, Thailand

[c.] Department of Chemical, Pharmaceutical and Agricultural Sciences (DOCPAS), University of Ferrara, Via L. Borsari 46, Ferrara, 44121 Italy

*E-mail: javier.mateos@univie.ac.at

TABLE OF CONTENTS

TABLE OF CONTENTS 1

MATERIALS AND METHODS 8

SOLVENTS 8

CHROMATOGRAPHY 8

SPECTROSCOPY AND INSTRUMENTS 8

COMPUTATIONAL DETAILS 10

STARTING MATERIALS 11

SAFETY STATEMENT 11

AUTHOR CONTRIBUTIONS 11

EXPERIMENTAL DATA – SYNTHETIC PROCEDURES 13

LIGHT SOURCES, PHOTOREACTORS AND EXPERIMENTAL SETUPS 13

Light sources for photoreactions 13

Photoreactor for reactions in 4-mL vials 14

Experimental setup for photoreactions at 0.50 mmol scale 15

Experimental setup for photoreactions at 1.00 mmol scale 15

Experimental setup for photoreactions at 5.00 mmol scale 16

PROCEDURES FOR THE SYNTHESIS OF PHOTOCATALYSTS 17

3,6-Di-tert-butyl-9-mesitylxanthylium tetrafluoroborate ([2a] BF_4) 17

Bis(terpyridine)osmium(II) hexafluorophosphate 18

GENERAL SCHEME OF THIOXANTHYLIUM PHOTOCATALYST SYNTHESIS 19

3-(*Tert*-butyl)benzenethiol (S1) 19

4-(*Tert*-butyl)-2-iodo-1-methylbenzene (S2) 20

4-(*Tert*-butyl)-2-iodobenzoic acid (S3) 21

Methyl 4-(*tert*-butyl)-2-iodobenzoate (S4) 22

Methyl 4-(*tert*-butyl)-2-((3-(*tert*-butyl)phenyl)thio)benzoate (S5) 22

3,6-Di-*tert*-butyl-thioxanthen-9-one (S6) 23

3,6-Di-*tert*-butyl-9-mesitylthioxanthylium tetrafluoroborate ([3a] BF_4) 24

GENERAL SCHEME OF SELENOXANTHYLIUM PHOTOCATALYSTS SYNTHESIS 25

1,2-Bis(3-(*tert*-butyl)phenyl)diselane (S7) 25

Methyl 4-(*tert*-butyl)-2-((3-(*tert*-butyl)phenyl)selanyl)benzoate (S8) 27

3,6-Di-*tert*-butyl-selenoxanthen-9-one (5) 27

3,6-Di-*tert*-butyl-9-mesitylselenoxanthylium tetrafluoroborate ([4a] BF_4) 28

3,6-Di-*tert*-butyl-9-(4-fluoro-2,6-dimethylphenyl)selenoxanthylium tetrafluoroborate ([4b] BF_4) 29
3,6-Di-*tert*-butyl-9-(2,6-dimethylphenyl)selenoxanthylium tetrafluoroborate ([4c] BF_4) 30
3,6-Di-*tert*-butyl-9-(4-methoxy-2,6-dimethylphenyl)selenoxanthylium tetrafluoroborate ([4d] BF_4) 31

CATALYTIC PROPERTIES OF XANTHYLIUM SALTS 33

[4+2]-Cycloaddition reaction catalyzed by acridinium and xanthylium salts 33
C–H amination reaction catalyzed by selenoxanthylium salts 38
[2+2]-Cycloaddition reaction catalyzed by acridinium and xanthylium salts 40
Boronic acid oxidation reaction catalyzed by xanthylium salts 41
Aza-Henry reaction catalyzed by xanthylium salts 44
Desulfinylative trifluoromethylation reaction catalyzed by xanthylium salts 46
Oxidation of anthracene catalyzed by selenoxanthylium salts at 0.10 mmol scale. 50

PHOTOCATALYST CHARACTERIZATION 52

ELECTROCHEMICAL DATA 52
3,6-Di-tert-butyl-9-mesitylacridinium tetrafluoroborate ([1a] BF_4) 52
3,6-Di-*tert*-butyl-9-mesitylxanthylium tetrafluoroborate ([2a] BF_4) 52
3,6-Di-*tert*-butyl-9-mesitylthioxanthylium tetrafluoroborate ([3a] BF_4) 53
3,6-Di-*tert*-butyl-9-mesitylselenoxanthylium tetrafluoroborate ([4a] BF_4) 54
3,6-Di-*tert*-butyl-9-(4-fluoro-2,6-dimethylphenyl)selenoxanthylium tetrafluoroborate ([4b] BF_4) 55
3,6-Di-*tert*-butyl-9-(2,6-dimethylphenyl)selenoxanthylium tetrafluoroborate ([4c] BF_4) 56
3,6-Di-*tert*-butyl-9-(4-methoxy-2,6-dimethylphenyl)selenoxanthylium tetrafluoroborate ([4d] BF_4) 57
Electrochemical properties comparison – O vs S vs Se (i.e., [2a] vs [3a] vs [4a]) 58
Electrochemical properties comparison – Reduction – F vs H vs Me vs OMe (i.e., [4a] vs [4b] vs [4c] vs [4d]) 59
SPECTROPHOTOMETRIC DATA 60
3,6-Di-*tert*-butyl-9-mesitylacridinium tetrafluoroborate ([1a] BF_4) 60
3,6-Di-*tert*-butyl-9-mesitylxanthylium tetrafluoroborate ([2a] BF_4) 61
3,6-Di-*tert*-butyl-9-mesitylthioxanthylium tetrafluoroborate ([3a] BF_4) 63
3,6-Di-*tert*-butyl-9-mesitylselenoxanthylium tetrafluoroborate ([4a] BF_4) 65
3,6-Di-*tert*-butyl-9-(4-fluoro-2,6-dimethylphenyl)selenoxanthylium tetrafluoroborate ([4b] BF_4) 68
3,6-Di-*tert*-butyl-9-(2,6-dimethylphenyl)selenoxanthylium tetrafluoroborate ([4c] BF_4) 70
3,6-Di-*tert*-butyl-9-(4-methoxy-2,6-dimethylphenyl)selenoxanthylium tetrafluoroborate ([4d] BF_4) 72
SUMMARY OF PHOTOCATALYST ABSORPTION PROPERTIES 74
Absorption comparison – [4a] BF_4 in polar solvents 74
Absorption comparison – [2a] vs [3a] vs [4a] 75
Absorption comparison – F vs H vs Me vs OMe (i.e., [4a] vs [4b] vs [4c] vs [4d]) 76
LUMINESCENCE PROPERTIES AT ROOM TEMPERATURE 77

STATIONARY EMISSION DATA 77
3,6-Di-*tert*-butyl-9-mesitylacridinium tetrafluoroborate ([1a] BF_4) 77
3,6-Di-*tert*-butyl-9-mesitylxanthylium tetrafluoroborate ([2a] BF_4) 77
3,6-Di-*tert*-butyl-9-mesitylthioxanthylium tetrafluoroborate ([3a] BF_4) 78
3,6-Di-*tert*-butyl-9-mesitylselenoxanthylium tetrafluoroborate ([4a] BF_4) 78
3,6-Di-*tert*-butyl-9-(4-fluoro-2,6-dimethylphenyl)selenoxanthylium tetrafluoroborate ([4b] BF_4) 79
3,6-Di-*tert*-butyl-9-(2,6-dimethylphenyl)selenoxanthylium tetrafluoroborate ([4c] BF_4) 79
3,6-Di-*tert*-butyl-9-(4-methoxy-2,6-dimethylphenyl)selenoxanthylium tetrafluoroborate ([4d] BF_4) 80
TIME-RESOLVED EMISSION DATA 81
3,6-Di-*tert*-butyl-9-mesitylacridinium tetrafluoroborate ([1a] BF_4) 81
3,6-Di-*tert*-butyl-9-mesitylxanthylium tetrafluoroborate ([2a] BF_4) 82
3,6-Di-*tert*-butyl-9-mesitylthioxanthylium tetrafluoroborate ([3a] BF_4) 83
3,6-Di-*tert*-butyl-9-mesitylselenoxanthylium tetrafluoroborate ([4a] BF_4) 84
3,6-Di-*tert*-butyl-9-(4-fluoro-2,6-dimethylphenyl)selenoxanthylium tetrafluoroborate ([4b] BF_4) 85
3,6-Di-*tert*-butyl-9-(2,6-dimethylphenyl)selenoxanthylium tetrafluoroborate ([4c] BF_4) 86
SUMMARY OF PHOTOCATALYST LUMINESCENCE PROPERTIES 87
LUMINESCENCE PROPERTIES AT 77 K 90
3,6-Di-*tert*-butyl-9-mesitylselenoxanthylium tetrafluoroborate ([4a] BF_4) 90
3,6-Di-*tert*-butyl-9-(4-fluoro-2,6-dimethylphenyl)selenoxanthylium tetrafluoroborate ([4b] BF_4) 90
3,6-Di-*tert*-butyl-9-(2,6-dimethylphenyl)selenoxanthylium tetrafluoroborate ([4c] BF_4) 91
3,6-Di-*tert*-butyl-9-(4-methoxy-2,6-dimethylphenyl)selenoxanthylium tetrafluoroborate ([4d] BF_4) 91
SUMMARY OF PHOTOCATALYST PHOTOREDOX PROPERTIES 92
ULTRAFAST SPECTROSCOPY 93
3,6-Di-*tert*-butyl-9-mesitylxanthylium tetrafluoroborate ([2a] BF_4) in CH_3CN 93
3,6-Di-*tert*-butyl-9-mesitylthioxanthylium tetrafluoroborate ([3a] BF_4) in CH_3CN 94
3,6-Di-*tert*-butyl-9-mesitylselenoxanthylium tetrafluoroborate ([4a] BF_4) 95
3,6-Di-*tert*-butyl-9-(4-fluoro-2,6-dimethylphenyl)selenoxanthylium tetrafluoroborate ([4b] BF_4) 97
3,6-Di-*tert*-butyl-9-(2,6-dimethylphenyl)selenoxanthylium tetrafluoroborate ([4c] BF_4) 99
3,6-Di-*tert*-butyl-9-(4-methoxy-2,6-dimethylphenyl)selenoxanthylium tetrafluoroborate ([4d] BF_4) 101
NANOSECOND LASER FLASH PHOTOLYSIS 103
3,6-Di-*tert*-butyl-9-mesitylselenoxanthylium tetrafluoroborate ([4a] BF_4) 103
3,6-Di-*tert*-butyl-9-(4-fluoro-2,6-dimethylphenyl)selenoxanthylium tetrafluoroborate ([4b] BF_4) 104
3,6-Di-*tert*-butyl-9-(2,6-dimethylphenyl)selenoxanthylium tetrafluoroborate ([4c] BF_4) 105
3,6-Di-*tert*-butyl-9-(4-methoxy-2,6-dimethylphenyl)selenoxanthylium tetrafluoroborate ([4d] BF_4) 106
SUMMARY OF LASER FLASH PHOTOLYSIS 107
EXCITED STATE TOPOLOGY OF THE SELENOXANTHYLIUM PHOTOCATALYSTS 108

3,6-Di-*tert*-butyl-9-mesitylselenoxanthylium tetrafluoroborate ([4a] BF_4) 108
3,6-Di-*tert*-butyl-9-(4-fluoro-2,6-dimethylphenyl)selenoxanthylium tetrafluoroborate ([4b] BF_4) 108
3,6-Di-*tert*-butyl-9-(2,6-dimethylphenyl)selenoxanthylium tetrafluoroborate ([4c] BF_4) 109
3,6-Di-*tert*-butyl-9-(4-methoxy-2,6-dimethylphenyl)selenoxanthylium tetrafluoroborate ([4d] BF_4) 109
FINAL COMMENTS ON THE PHOTOPHYSICS 110
PRIMARY PHOTOCHEMICAL EVENT INVOLVING SELENOXANTHYLIUM PHOTOCATALYSTS 111
[4+2] cycloaddition 111
C(sp^2)–H amination 113

PHOTOCATALYST STABILITY 115

PHOTOCATALYST STABILITY IN THE GROUND STATE 115
Reaction of [2a] BF_4 and [4a] BF_4 with aniline 115
^{1}H NMR spectrum of acridinium [1a] BF_4 in CD_2Cl_2 116
^{1}H NMR spectra of xanthylium [2a] BF_4 and reaction with aniline 117
^{1}H NMR spectra of selenoxanthylium [4a] BF_4 and reaction with aniline 119
Conversion of xanthylium salts to [1a] BF_4 121
PHOTOCATALYST STABILITY UNDER REACTION CONDITIONS 122
[4+2]-Cycloaddition reaction catalyzed by selenoxanthylium salts at 1.00 mmol scale 122
^{1}H NMR of [4a] BF_4 after after [4+2]-cycloaddition reaction in $CDCl_3$ 123
Desulfinylative trifluoromethylation reaction catalyzed by selenoxanthylium salt at 0.25 mmol scale 124

KINETIC STUDIES OF PHOTOCATALYTIC REACTIONS 125

[4+2]-Cycloaddition reaction at 0.10 mmol scale 125
C–H amination reaction at 0.10 mmol scale 126
EX SITU IRRADIATION NMR EXPERIMENTS 128
[4+2]-Cycloaddition reaction at 0.10 mmol scale catalyzed by photocatalysts 128
^{1}H NMR spectroscopy of [4+2] cycloaddition reaction catalyzed by [2a] BF_4 130
Reaction profile of [2a] BF_4 catalyzed [4+2] cycloaddition reaction 131
^{1}H NMR spectroscopy of [4+2] cycloaddition reaction catalyzed by [3a] BF_4 133
Reaction profile of [3a] BF_4 catalyzed [4+2] cycloaddition reaction 134
^{1}H NMR spectroscopy of [4+2] cycloaddition reaction catalyzed by [4a] BF_4 136
Reaction profile of [4a] BF_4 catalyzed [4+2] cycloaddition reaction 137
^{1}H NMR spectroscopy of [4+2] cycloaddition reaction catalyzed by Triangulenium Ion 139
^{1}H NMR spectroscopy of [4+2] cycloaddition reaction catalyzed by $Os(terpy)_2$ 141
^{1}H NMR spectroscopy of [4+2] cycloaddition reaction catalyzed by $Ru(bpy)_3$ 143
SUMMARY OF KINETIC DATA FOR [4+2] CYCLOADDITION REACTION 145
C–H amination reaction at 0.10 mmol scale catalyzed by photocatalysts 146

^{1}H NMR spectroscopy of C–H amination reaction catalyzed by [2a] BF_4 147
Reaction profile of [2a] BF_4 catalyzed C–H amination reaction 148
^{1}H NMR spectroscopy of C–H amination reaction catalyzed by [3a] BF_4 150
Reaction profile of [3a] BF_4 catalyzed C–H amination reaction 151
^{1}H NMR spectroscopy of C–H amination reaction catalyzed by [4d] BF_4 153
Reaction profile of [4d] BF_4 catalyzed C–H amination reaction 154
^{1}H NMR spectroscopy of C–H amination reaction catalyzed by Triangulelium Ion 156
Reaction profile of Triangulenium Ion catalyzed C–H amination reaction 157
^{1}H NMR spectroscopy of C–H amination reaction catalyzed by $Os(terpy)_2$ 159
^{1}H NMR spectroscopy of C–H amination reaction catalyzed by $Ru(bpy)_3$ 161
SUMMARY OF KINETIC DATA FOR C–H AMINATION REACTION 163

X-RAY CRYSTALLOGRAPHIC ANALYSIS 164

SELENOXANTHYLIUM SALT [4A] BF_4 (CCDC 2544588) 164

COMPUTATIONAL DATA 166

COMPARISON BETWEEN TD-DFT AND EXPERIMENTAL ABSORPTION SPECTRA 166
Xanthylium $[2a]^+$ 166
Thioxanthylium $[3a]^+$ 170
Selenoxanthylium $[4a]^+$ 173
SOC COMPARISON 176
COORDINATES FOR XANTHYLIUM $[2A]^+$ 176
COORDINATES FOR THIOXANTHYLIUM $[3A]^+$ 180
COORDINATES FOR SELENOXANTHYLIUM $[4A]^+$ 183

SPECTROSCOPIC DATA 186

^{1}H NMR OF [2A] BF_4 186
^{13}C NMR OF [2A] BF_4 187
^{19}F NMR OF [2A] BF_4 188
^{1}H NMR OF $OS(TERPY)_2$ $(PF_6)_2$ 189
^{13}C NMR OF $OS(TERPY)_2$ $(PF_6)_2$ 190
^{19}F NMR OF $OS(TERPY)_2$ $(PF_6)_2$ 191
^{1}H NMR OF [3A] BF_4 192
^{13}C NMR OF [3A] BF_4 193
^{19}F NMR OF [3A] BF_4 194
^{1}H NMR OF 3,6-DI-*TERT*-BUTYL-SELENOXANTHEN-9-ONE (5) 195

^{13}C NMR OF 3,6-DI-*TERT*-BUTYL-SELENOXANTHEN-9-ONE (5) 196
^{77}Se NMR OF 3,6-DI-*TERT*-BUTYL-SELENOXANTHEN-9-ONE (5) 197
^{1}H NMR OF [4A] BF_4 198
^{13}C NMR OF [4A] BF_4 199
^{19}F NMR OF [4A] BF_4 200
^{77}Se NMR OF [4A] BF_4 201
^{1}H NMR OF [4B] BF_4 202
^{13}C NMR OF [4B] BF_4 203
^{19}F NMR OF [4B] BF_4 204
^{77}Se NMR OF [4B] BF_4 205
^{1}H NMR OF [4C] BF_4) 206
^{13}C NMR OF [4C] BF_4 207
^{19}F NMR OF [4C] BF_4 208
^{77}Se NMR OF [4C] BF_4 209
^{1}H NMR OF [4D] BF_4) 210
^{13}C NMR OF [4D] BF_4 211
^{19}F NMR OF [4D] BF_4 212
^{77}Se NMR OF [4D] BF_4 213
^{1}H NMR OF (1*R*,2*R*)-4'-METHOXY-2,4,5-TRIMETHYL-1,2,3,6-TETRAHYDRO-1,1'-BIPHENYL (9) 214
^{13}C NMR OF (1*R*,2*R*)-4'-METHOXY-2,4,5-TRIMETHYL-1,2,3,6-TETRAHYDRO-1,1'-BIPHENYL (9) 215
^{1}H NMR OF 3,5-DIMETHYL-1-(2,4,6-TRIMETHOXYPHENYL)-1H-PYRAZOLE (12) 216
^{13}C NMR OF 3,5-DIMETHYL-1-(2,4,6-TRIMETHOXYPHENYL)-1H-PYRAZOLE (12) 217
^{1}H NMR OF 1-METHOXY-4-((1*S*,2*R*,4*S*)-2-METHYL-4-PHENYLCYCLOBUTYL)BENZENE (14) 218
^{13}C NMR OF 1-METHOXY-4-((1*S*,2*R*,4*S*)-2-METHYL-4-PHENYLCYCLOBUTYL)BENZENE (14) 219
^{1}H NMR OF 4-(*TERT*-BUTYL)PHENOL (16) 220
^{13}C NMR OF 4-(*TERT*-BUTYL)PHENOL (16) 221
^{1}H NMR OF 1-(NITROMETHYL)-2-PHENYL-1,2,3,4-TETRAHYDROISOQUINOLINE (18) 222
^{13}C NMR OF 1-(NITROMETHYL)-2-PHENYL-1,2,3,4-TETRAHYDROISOQUINOLINE (18) 223
^{1}H NMR OF 4-(*TERT*-BUTYL)-2-IODO-1-METHYLBENZENE (S2) 224
^{13}C NMR OF 4-(*TERT*-BUTYL)-2-IODO-1-METHYLBENZENE (S2) 225
^{1}H NMR OF METHYL 4-(*TERT*-BUTYL)-2-IODOBENZOATE (S4) 226
^{13}C NMR OF METHYL 4-(*TERT*-BUTYL)-2-IODOBENZOATE (S4) 227
^{1}H NMR OF METHYL 4-(*TERT*-BUTYL)-2-((3-(*TERT*-BUTYL)PHENYL)THIO)BENZOATE (S5) 228

^{13}C NMR OF METHYL 4-(*TERT*-BUTYL)-2-((3-(*TERT*-BUTYL)PHENYL)THIO)BENZOATE (S5) 229
^{1}H NMR OF 3,6-DI-*TERT*-BUTYL-THIOXANTHEN-9-ONE (S6) 230
^{13}C NMR OF 3,6-DI-*TERT*-BUTYL-THIOXANTHEN-9-ONE (S6) 231
^{1}H NMR OF METHYL 4-(*TERT*-BUTYL)-2-((3-(*TERT*-BUTYL)PHENYL)SELANYL)BENZOATE (S8) 232
^{13}C NMR OF METHYL 4-(*TERT*-BUTYL)-2-((3-(*TERT*-BUTYL)PHENYL)SELANYL)BENZOATE (S8) 233
^{77}Se NMR OF METHYL 4-(*TERT*-BUTYL)-2-((3-(*TERT*-BUTYL)PHENYL)SELANYL)BENZOATE (S8) 234

REFERENCES 235

MATERIALS AND METHODS

All air- and moisture-insensitive reactions were carried out under ambient atmosphere in round-bottom flasks, vials fitted with rubber septa, or Schlenck flasks. The reaction progress was monitored by thin-layer chromatography (TLC). Concentration under reduced pressure was performed by rotary evaporation at 40 °C at an appropriate pressure. Purified compounds were further dried under high vacuum (0.010–0.005 mbar). Yields refer to purified and spectroscopically pure compounds, unless otherwise stated.

SOLVENTS

Technical heptane and EtOAc were distilled using a 20-L Rotary evaporator. Dry CH_3CN, dry DMSO, dry CH_2Cl_2 were purchased from Acros Organics. Reactions were performed using technical solvents obtained from Acros Organics, TCI, and Sigma-Aldrich, unless otherwise stated.

CHROMATOGRAPHY

Thin layer chromatography (TLC) was performed on aluminum plates coated with silica gel 60 F_{254} with 0.20 mm thickness purchased from Macherey-Nagel®. Chromatograms were visualized by fluorescence quenching with UV light at 254 nm or by staining using potassium permanganate or phosphomolybdic acid stains followed by heating. Flash column chromatography was performed using silica gel 60 (230-400 mesh, Macherey-Nagel®).

SPECTROSCOPY AND INSTRUMENTS

NMR spectroscopy

^{1}H NMR, ^{13}C NMR, ^{19}F NMR, and ^{77}Se spectra were recorded using a Bruker AV-400, AV-500, AV-600 or AV-700 spectrometer at 300 K. Chemical shifts (δ) are given in parts per million (ppm), referenced to the solvent peak: for $CDCl_3$, defined at δ = 7.26 ppm (^{1}H NMR) and δ = 77.16 ppm (^{13}C NMR); for DMSO-d_6, defined at δ = 2.50 ppm (^{1}H NMR) and δ = 39.52 ppm (^{13}C NMR). Coupling constants (J) are reported in Hertz (Hz). ^{1}H NMR splitting patterns are designated as singlet (s), doublet (d), triplet (t), quartet (q), quintet (quint.) or a combination thereof, as they appeared in the spectrum. Splitting patterns that could not be interpreted or easily visualized are designated as multiplet (m) or broad (br). NMR shifts of heteronuclei were referenced using their respective Ξ value using the *xiref* au program in Bruker Topspin following IUPAC recommendations.[1]

Mass spectrometry

Mass spectra were obtained using a Bruker maXis UHR-TOF spectrometer (70 eV), using electrospray ionization (ESI) or atmospheric-pressure chemical ionization (APCI) or an Agilent 7200B GC/Q-TOF spectrometer (70 eV), using electron ionization (EI). MALDI-TOF Analysis: high-resolution MALDI-TOF MS analyses were performed using a Bruker timsTOF fleX ESI/MALDI dual source – trapped ion mobility separation – Q-TOF massspectrometer in positive ion mode. The sum formulas of the detected ions were determined using Bruker Compass DataAnalysis 5.3 based on the mass accuracy ($\Delta m/z \leq 5$

ppm) and isotopic pattern matching (SmartFormula algorithm). One microliter of a solution of the matrix (trans-2-[3-(4-t-butylphenyl)-2-methyl-2-propenylidene]malonitrile, DCTB) in CH_2Cl_2 was spotted onto a well of the MALDI plate, and the solvent was allowed to evaporate. The solvent was evaporated prior to analysis. Data were collected in positive polarity mode.

SXRD

Single crystal X-ray diffraction data were collected with a Stadivari Diffractometer (STOE & Cie GmbH, Germany) equipped with an EIGER2 R500 detector (Dectris Ltd, Switzerland). Data were processed and scaled with the STOE software suite X-Area (STOE & Cie GmbH). Structures were solved with SHELXT[2] and refined with SHELXL[3] or Olex2.[4] Model building was done with Olex2 or ShelXle.[5] Structures were validated with CHECKCIF (https://checkcif.iucr.org/).

See the respective CIF files for exact versions and more details.

Cyclic voltammetry

Cyclic voltammetry was measured using the Ossila potentiostat, an Ag/$AgNO_3$ (0.10 M in CH_3CN) reference electrode, a glassy carbon disc working electrode, and a platinum disk counter electrode (purchased from “ALS Electrochemistry & Spectroelectrochemistry”). All measurements were carried out in CH_3CN with 0.10 M NBu_4PF_6 as electrolyte if not stated otherwise. The electrolyte was purchased from BLDPharm®, recrystallized twice from ethanol, dried in vaccuo for 24h ($5 \cdot 10^{-3}$ bar), and stored in a desiccator. Voltammograms were referenced to the Fc/Fc^+ redox couple as internal standard. If Fc/Fc^+ was found to react with the target analyte, the voltammogram was first measured against the Fc/Fc^+ calibrated reference electrode (Ag/$AgNO_3$) and then externally referenced to the Fc/Fc^+ redox couple (+0.053 V). Conversion of the Fc/Fc^+ referenced potentials to SCE was done by adding the potentials reported by *Pavlishchuk* and *Addison* (Fc/Fc^+: 0.380 V vs SCE).[6]

UV-Vis absorption spectroscopy

UV-Vis absorption spectroscopy was recorded using a Jasco V-750 UV-Vis Spectrophotometer running in double beam mode with a matched pair of quartz absorbance cuvettes (1 × 1 cm). All absorption measurements were performed at room temperature, unless specified otherwise. The molar attenuation coefficient (ε) was determined by dissolving a known amount of compound (typically 1–2 mg) and diluting the resulting stock solution to achieve 5 solutions with an appropriate concentration for measurements. The plot of absorbance versus concentration was fitted with a linear function and the molar attenuation coefficient was obtained from the slope.

Emission spectroscopy

Emission spectra were recorded on an Agilent Technologies Cary Eclipse spectrofluorometer. Fluorescence lifetimes were measured by time-correlated single-photon-counting (TCSPC) apparatus (PicoQuant Picoharp 300) equipped with subnanosecond LED sources (280, 380, 460, and 600 nm, 500−700 ps pulse width) powered by a PicoQuant PDL 800-B variable (2.5−40 MHz) pulsed power supply. The decays were analyzed by means of PicoQuant FluoFit Global fluorescence decay analysis software. Time-gated emission spectra at 77 K (4:1 EtOH:MeOH glassy matrix) were obtained using laser flash photolysis (LFP) performed on a custom laser spectrometer comprised of a Continuum

Surelite II Nd:YAG laser (FWHM = 8 ns) with a frequency doubled option (532 nm) as the excitation source and a Princeton Instruments PIMAX II gated intensified CCD camera as the detector.

Ultrafast spectroscopy

Ultrafast spectroscopy measurements were performed using the Harpia-Light spectrometer (Light Conversion) equipped with a carbide femtosecond laser operating with an output wavelength of 1030 nm, a repetition rate of 60 kHz, and a pulse width of ca 290 fs. The pump beam (515 or 343 nm) was provided by second or third harmonic generation, respectively, while the probe beam (approximately covering the 435-935 spectral range) was produced by white light continuum generation using a YAG crystal. Data processing, including chirp and baseline correction, was performed using CarpetView 2.0 (Light Conversion).

Nanosecond transient absorption spectroscopy

Nanosecond transient absorption measurements were obtained using laser flash photolysis (LFP) performed on a custom laser spectrometer comprised of a Continuum Surelite II Nd:YAG laser (FWHM = 8 ns) with a frequency doubled option (532 nm) and an Applied Photophysics xenon light source. Laser excitation was provided at 90° with respect to the white light probe beam. Light transmitted by the sample was focused onto the entrance slit of a 300 mm focal length Acton SpectraPro 2300i triple grating, double exit monochromator equipped with a photomultiplier detector (Hamamatsu R3896) and a Princeton Instruments PIMAX II gated intensified CCD camera. PMT signals (kinetic traces) were processed using a Teledyne LeCroy 604Zi (400 MHz, 20 GS/s) digital oscilloscope.

COMPUTATIONAL DETAILS

All calculations employed the ORCA 6.0.1 package.[7] Geometry optimizations were carried out at the ωB97X-D4/def2-SVP level of theory,[8] including Grimme's D4 dispersion correction.[9] Tight convergence criteria (TightSCF, TightOpt) and a fine integration grid (DefGrid3) were applied. Vibrational frequency calculations confirmed all stationary points as minima and provided zero-point energies and thermal corrections. Single-point energies were computed at the ωB97X-D4/def2-TZVP level with solvation effects included via the SMD model (acetonitrile).

Time-dependent DFT (TD-DFT) calculations were subsequently carried out as single-point calculations on the optimized geometries using five exchange-correlation functionals (ωB97X-D4, CAM-B3LYP-D4, PBE0-D4, B3LYP-D4, and TPSSh-D4) in combination with the def2-TZVP basis set. The performance of each functional was benchmarked against the experimental UV-Vis absorption spectra, and TPSSh-D4 was selected for the analysis of the excited-state properties, canonical molecular orbitals involved in the dominant electronic transitions, and spin-orbit coupling matrix elements (SOCMEs). Scalar relativistic effects were included through the exact two-component (X2C) Hamiltonian.[10,11] Calculated absorption intensities include electric dipole, magnetic dipole, and electric quadrupole contributions.

Spin–orbit coupling matrix elements (SOCMEs) between singlet and triplet states were computed using the SOMF(1X) approach as implemented in ORCA.[11] SOC calculations were performed on TD-DFT

states obtained at the TPSSh-D4/X2C-TZVPall level. Reported SOCME values correspond to $\langle S_n|\hat{H}_{SO}|T_m\rangle$ elements and are given in cm^{-1}.

STARTING MATERIALS

All substrates were used as received from commercial suppliers (Sigma-Aldrich, TCI, Fluorochem, or BLDPharm), unless otherwise stated.

SAFETY STATEMENT

The procedures reported in this work are intended for use only by individuals with proper training in experimental chemistry. All hazardous materials (solid, liquid, or gaseous) should be handled using the standard work procedures described in references such as Chapter 4 of "Prudent Practices in the Laboratory".[12] All chemical waste should be disposed of in accordance with local regulations. For general guidelines for the management of chemical waste, see Chapter 8 of "Prudent Practices in the Laboratory".[12]

During the course of this study, no explosions or violent decompositions occurred. A summary of the possible risks and hazards is described below:

Caution: When performing reactions in pressurized systems (such as closed vials, pressure tubes, and autoclaves), a blast shield must be used to minimize personal damage in case of an accident.

Caution: High-intensity LED sources can pose significant hazards, particularly due to emission capable of causing eye and skin damage. Direct or reflected exposure should be strictly avoided. Experiments should be conducted using appropriate shielding or enclosed setups, and wavelength-specific protective eyewear is recommended. In addition, thermal management is required, as high-power LEDs may generate substantial heat.

AUTHOR CONTRIBUTIONS

Conceptualization: Javier Mateos conceptualized the project.

Investigation: All the authors contributed to the investigation of this work.

Methodology: The synthetic methodology reported in this work was developed by Suwanan Uipanit, Till Schreyer, Kirill Zhiliaev, and Jan-Hendrik Kaup.

DFT calculations: The calculations reported in this work were performed by Javier Mateos.

Photophysical studies: UV-Vis absorption, fluorescence and cyclic voltammetry studies were performed by Till Schreyer. Transient absorption spectroscopy studies were performed by Mirco Natali.

Writing: The original draft was written by Javier Mateos. All authors contributed to the final version of this work.

Direction: Javier Mateos directed the project and secured the funding.

All authors have given approval to the final version of the manuscript.

Experimental data – Synthetic procedures

Light sources, photoreactors and experimental setups

Light sources for photoreactions

EvoluChem 18W LEDs (365PF, 405PF, 450PF, 525PF, 595PF, 650PF) were used for photoreactions. LEDs emission spectra are shown in **Figure S1**.

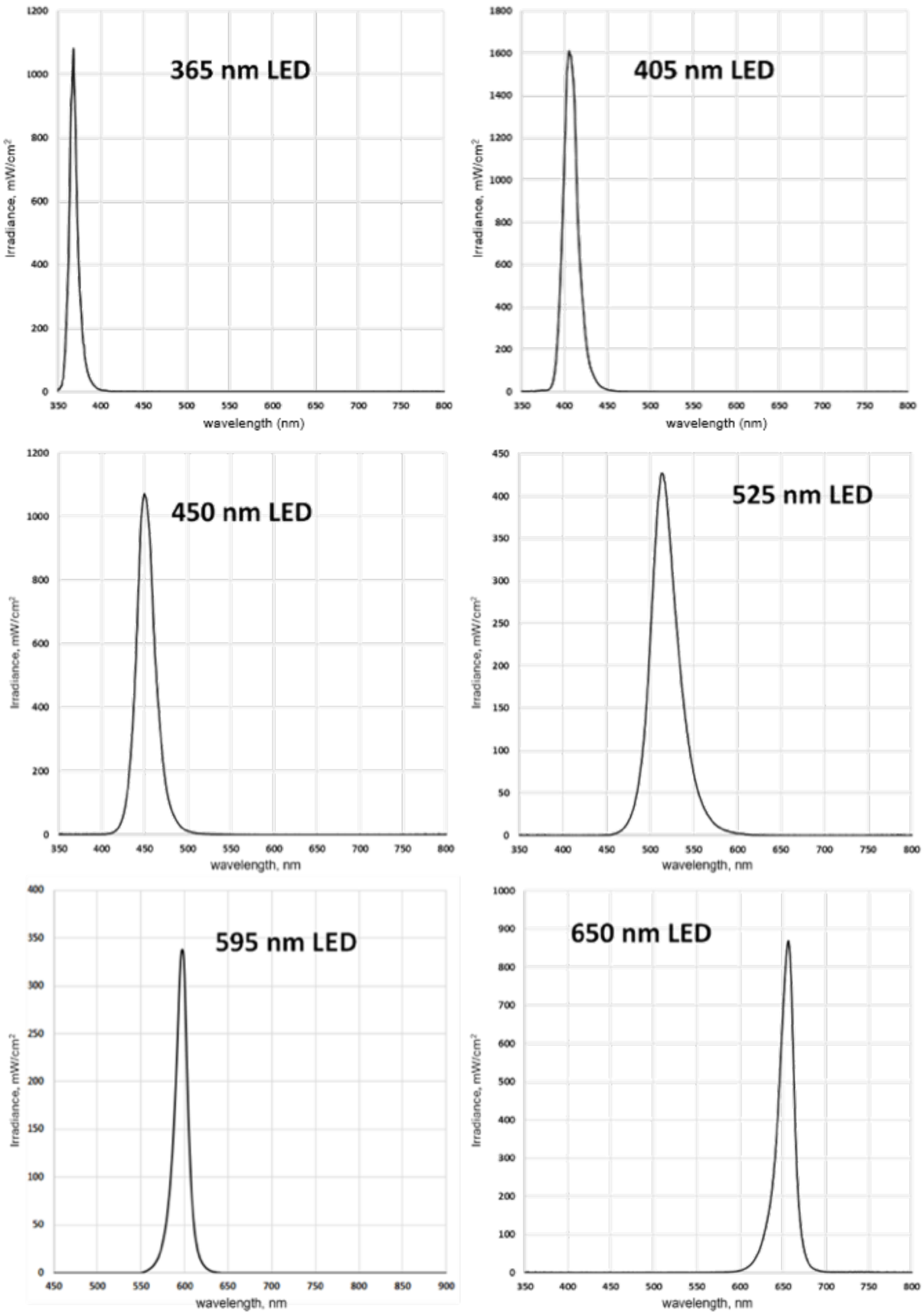

Figure S1. LEDs emission spectra, measured by the manufacturer. The original emission spectra can be accessed on the following link (accessed on 09/04/2026): https://hepatochem.com

Photoreactor for reactions in 4-mL vials

Pictures of the 3D printed photoreactor used for the reactions carried out in 4-mL scintillation vials are shown in **Figure S2**. During the experiments the temperature in the inner space of the reactor was 27±1 °C with a working cooling fan and 39±1 °C without cooling.

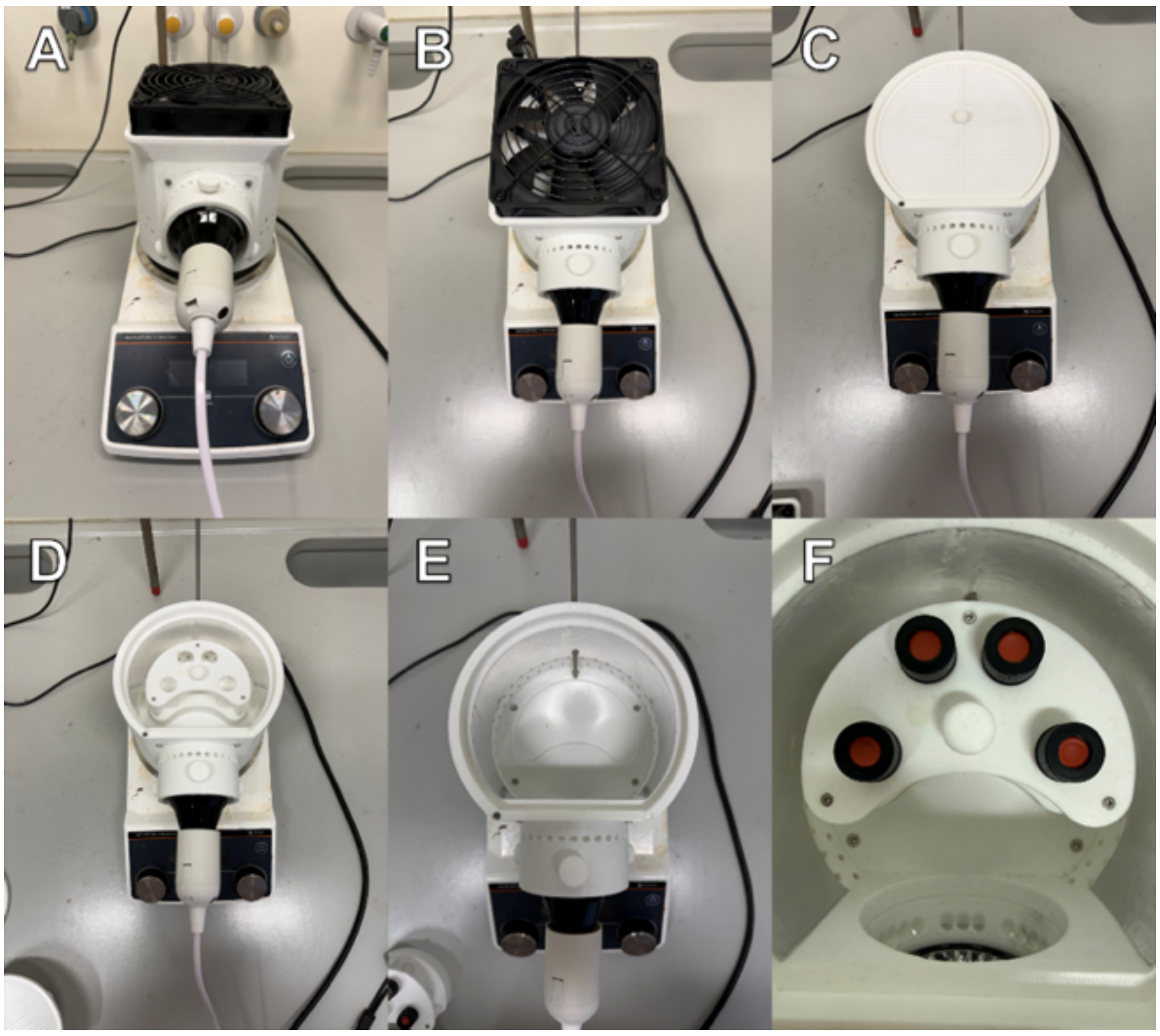

Figure S2. Photoreactor used for reactions in 4-mL scintillation borosilicate vials: **A.** Front view of reactor ready for usage; **B.** Top view of reactor ready for usage; **C.** Reactor without a cooling fan; **D.** Reactor without protecting screen; **E.** View of the reactor without the vial stand. The image also shows the position of the probe for temperature control; **F.** Inner space of the reactor equipped with four 4-mL scintillation borosilicate vials.

Experimental setup for photoreactions at 0.50 mmol scale

Photoreactions at 0.50 mmol scale were performed in 20-mL borosilicate vials with experimental setup shown in **Figure S3**. The distance from LED to the surface of the vial was set to 4.0 cm.

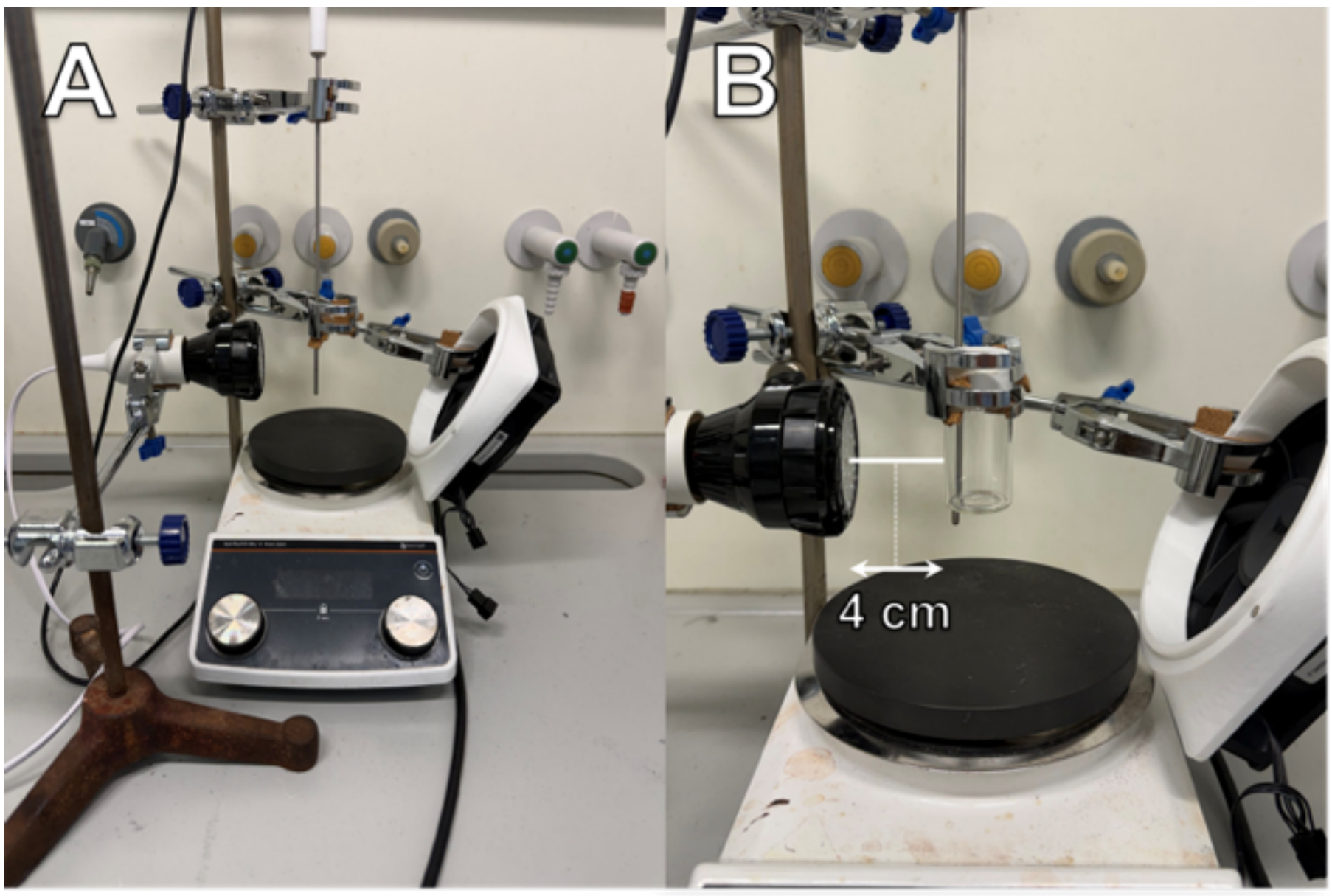

Figure S3. Experimental setup for the reactions at 0.50 mmol scale: **A.** General view of the experimental setup with a stirring plate, LED, and a cooling fan; **B.** A 20-mL borosilicate reaction vial fixed in a metal clamp.

Experimental setup for photoreactions at 1.00 mmol scale

Photoreactions at 1.00 mmol scale were performed in 50-mL round-bottom flasks with experimental setup shown in **Figure S4**. The distance from LED to the surface of the flask was set to 4.0 cm.

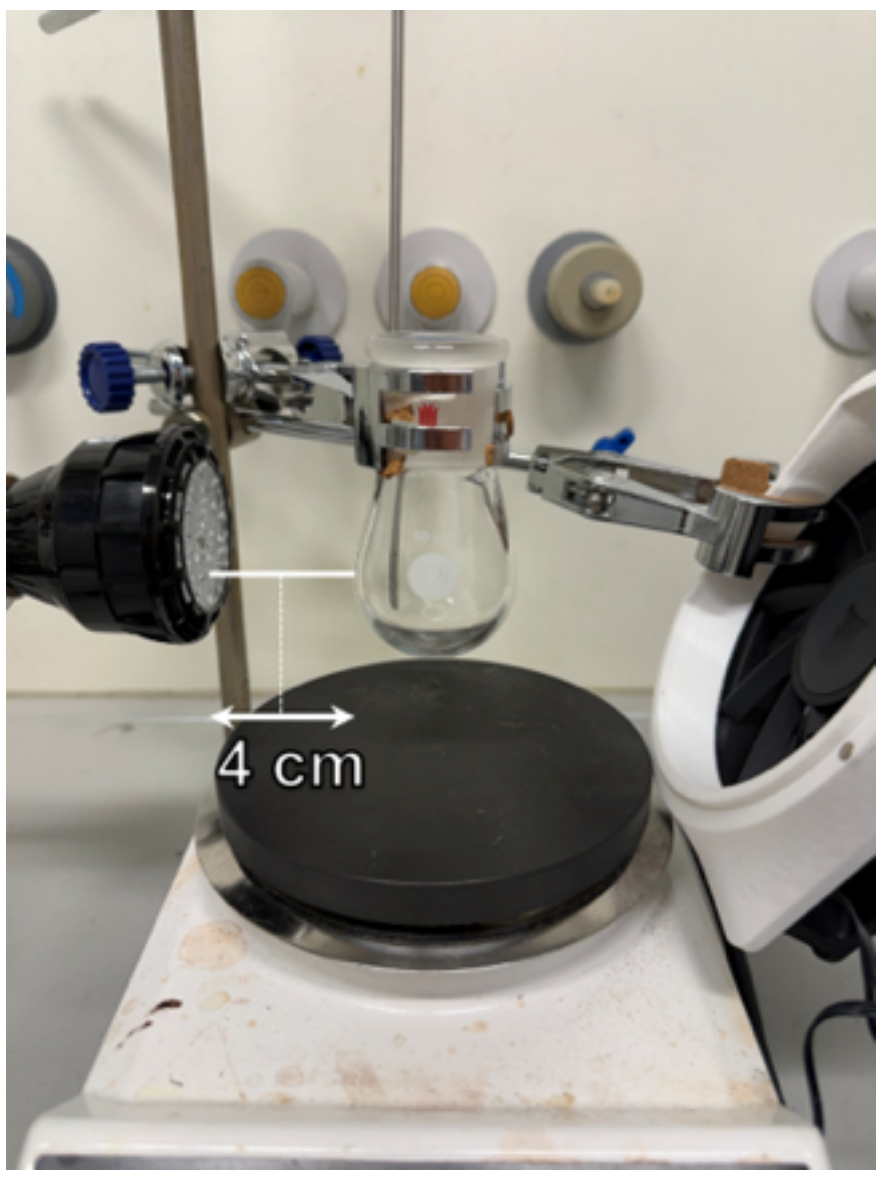

Figure S4. Experimental setup for the reactions at 1.00 mmol scale with a stirring plate, LED, a cooling fan and a 50-mL round-bottom reaction flask fixed in a metal clamp.

Experimental setup for photoreactions at 5.00 mmol scale

Photoreactions at 5.00 mmol scale were performed in 250-mL round-bottom flasks with experimental setup shown in **Figure S5**. The distance from LED to the surface of the flask was set to 4.0 cm.

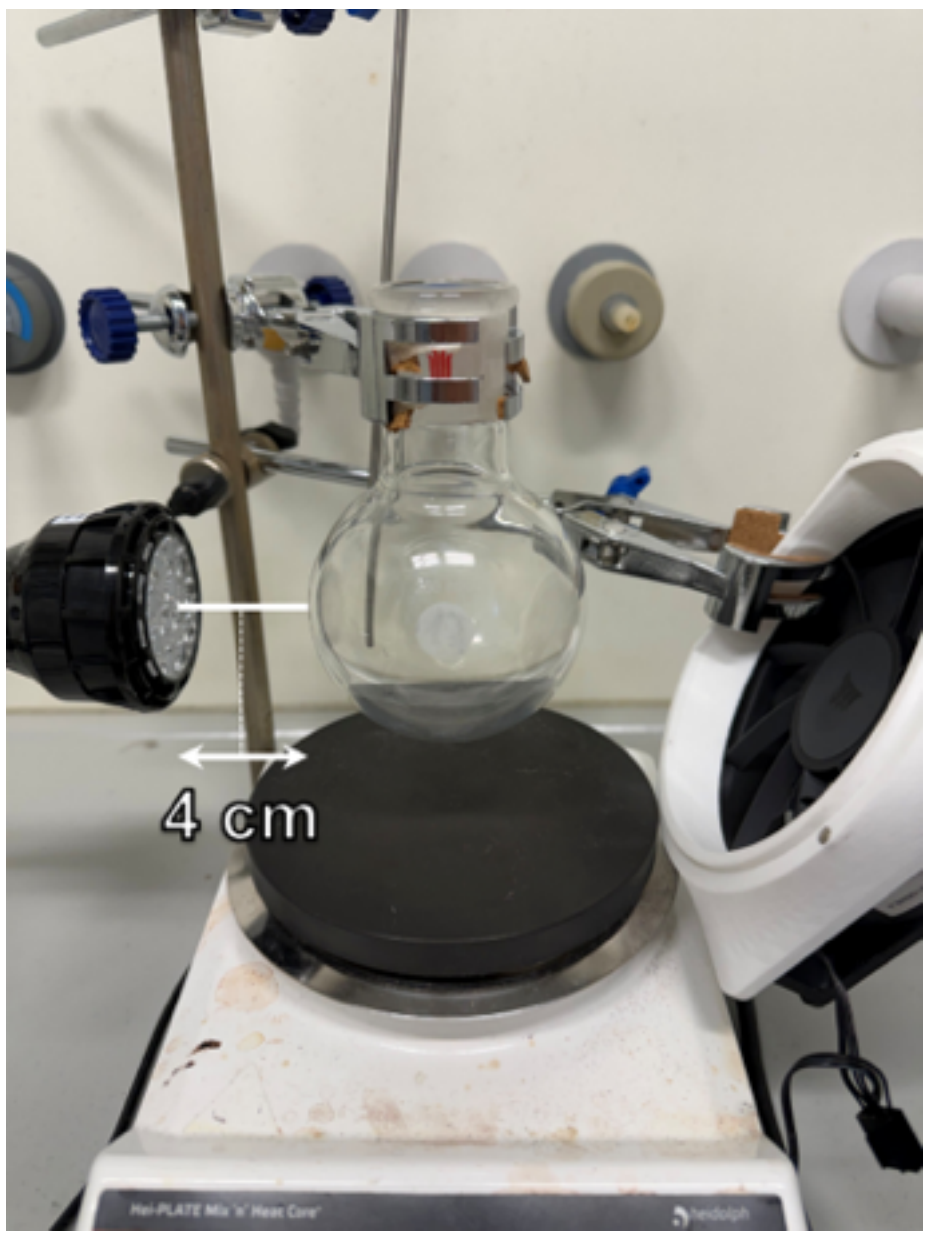

Figure S5. Experimental setup for the reactions at 5.00 mmol scale with a stirring plate, LED, a cooling fan and a 250-mL round-bottom reaction flask fixed in a metal clamp.

Procedures for the synthesis of photocatalysts

3,6-Di-tert-butyl-9-mesitylxanthylium tetrafluoroborate ([2a] BF_4)

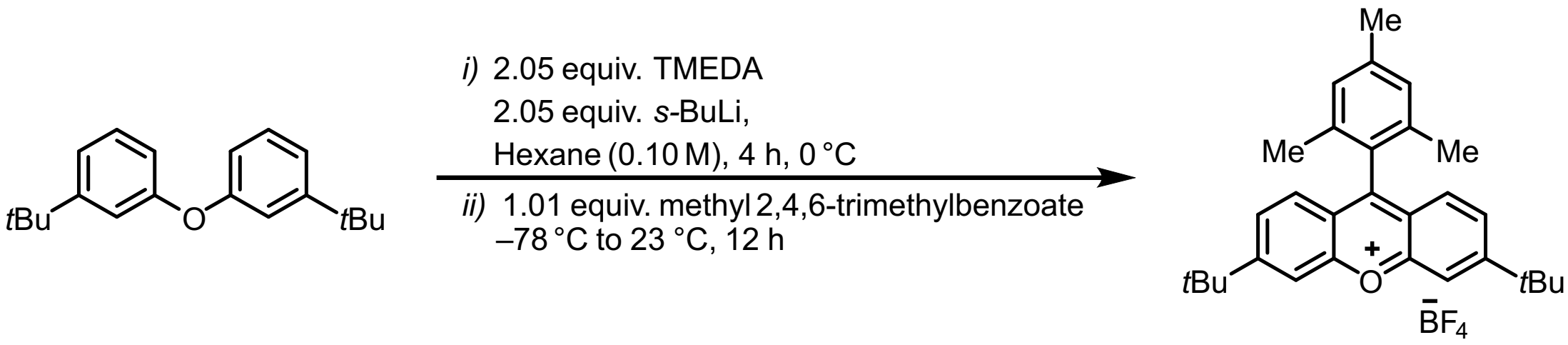

48% yield **[2a] BF_4**

i) To an oven-dried 250-mL round-bottomed Schlenk flask equipped with a magnetic stirring bar, 3,3'-oxybis(*tert*-butylbenzene) (1.20 g, 1.00 equiv., 4.25 mmol), and TMEDA (1.30 mL, 2.05 equiv., 8.71 mmol), were added. The flask was sealed with a septum, connected via a Tygon® tubing to a Schlenk line, and evacuated and purged with argon three times. Anhydrous *n*-hexane (4.2 mL) was added via 5-mL syringe, and the mixture was cooled to 0 °C using an ice bath. *Sec*-butyllithium (1.4 M solution in cyclohexane, 6.22 mL, 2.05 equiv., 8.71 mmol) was added via 10-mL syringe dropwise in 30 min. The ice bath was removed, and the reaction mixture was stirred at room temperature for 4 h.

ii) The reaction mixture was cooled to –78 °C using dry ice and an acetone bath, and a solution of methyl 2,4,6-trimethylbenzoate (765 mg, 1.01 equiv., 4.29 mmol) in anhydrous *n*-hexane (4.0 mL) was added slowly via cannula. After the addition, the reaction was allowed to slowly warm to room temperature and stirred for 12 h. The reaction was carefully quenched with water (4.0 mL), and the biphasic mixture was stirred vigorously for 30 min. The mixture was diluted with 25 mL of Et_2O and the layers were separated. The organic layer was washed with water (2 x 30 mL) and brine (1 x 30 mL). The organic layer was transferred to a 100 mL round-bottomed flask equipped with a stir bar. To the vigorously stirred solution was added concentrated HCl (3.0 mL) resulting in a bright yellow precipitate that slowly turned brown over the course of addition. The brown suspension was stirred vigorously for 30 min then diluted with water (30 mL). The layers were separated, and the organic layer was extracted with water (3 x 30 mL or until the washings become colorless). To the combined aqueous layers was added solid $NaBF_4$ (1.40 g, 3.00 equiv., 12.7 mmol) resulting in a bright yellow precipitate. The resulting suspension was extracted with CH_2Cl_2 (3 x 150 mL or until the washings become colorless). To the combined organic layers was added $HBF_4 \cdot Et_2O$ complex (50-55% w/w HBF_4, 760 µL, 1.00 equiv., 4.25 mmol). The solution was swirled to achieve homogeneity then washed with water (1×100 mL) and aqueous $NaBF_4$ (1.00 M, 100 mL). The organic layer was dried over solid $NaBF_4$, filtered, and concentrated to dryness. The residue was purified by trituration with hexanes and filtered. The solid was rinsed with *n*-pentane and dried in vacuo to give **[2a] BF_4** (1.03 g, 2.06 mmol, 48% yield) as a yellow solid.

NMR Spectroscopy:

^{1}H NMR (500 MHz, $CDCl_3$, 23 °C, δ): 8.50 (d, *J* = 1.8 Hz, 2H), 7.88 (dd, *J* = 8.9, 1.8 Hz, 2H), 7.74 – 7.71 (m, 2H), 7.15 (s, 2H), 2.47 (s, 3H), 1.85 (s, 6H), 1.52 (s, 18H).

^{13}C NMR (151 MHz, $CDCl_3$, 23 °C, δ): 185.6, 174.1, 171.1, 158.8, 141.4, 135.6, 129.2, 128.5, 122.2, 117.1, 37.7, 30.6, 21.4, 20.3.

^{19}F NMR (376 MHz, $CDCl_3$, 23 °C, δ): −153.34, −153.39.

HRMS-EI(m/z) calc'd for $C_{30}H_{35}O$ $[M]^+$, 411.2682; found, 411.2685; deviation: +0.7 ppm.

HRMS-ESI(m/z) calc'd for BF_4 $[M]^-$, 87.0035; found, 87.0033; deviation: −2.3 ppm.

Bis(terpyridine)osmium(II) hexafluorophosphate

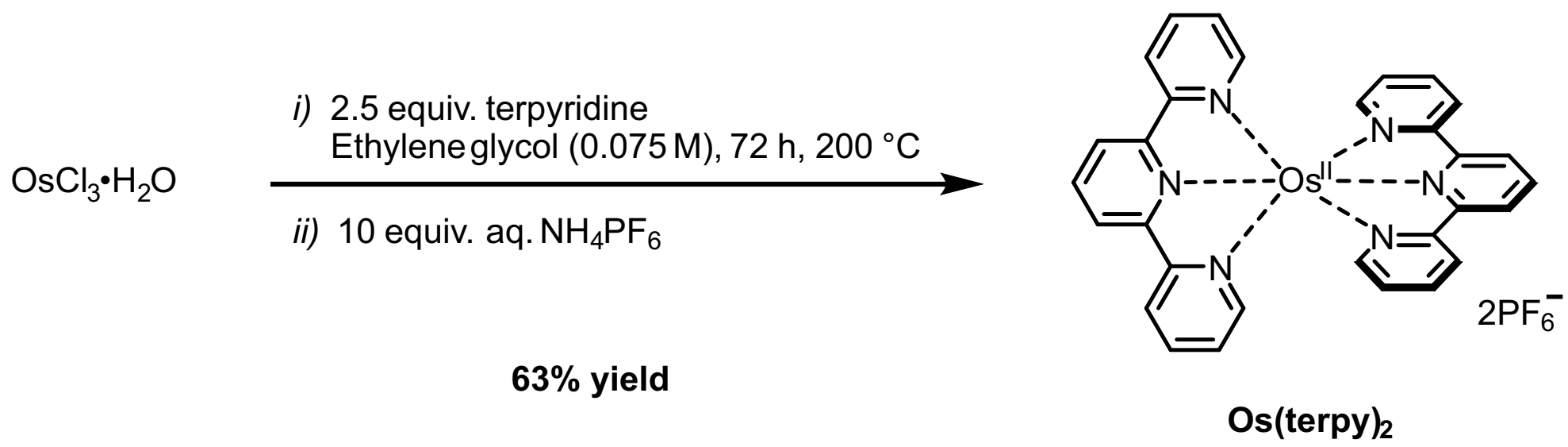

A modified version of the procedure reported by Rovis *et al.* was followed.[13]

i) An oven-dried 100-mL pressure tube was cycled into an argon-filled glovebox and charged sequentially with osmium(**III**) chloride hydrate (148 mg, 0.500 mmol, 1.00 equiv.), terpyridine (301 mg, 1.25 mmol, 2.50 equiv.), and anhydrous ethylene glycol (6.7 mL, 0.075 M). The tube was sealed tightly and removed from the glovebox, then heated to 200 °C in a sand bath. After 72 h, the reaction was allowed to cool to 60 °C and opened to ambient atmosphere.

ii) Ammonium hexafluorophosphate (823 mg, 5.0 mmol, 1.0 equiv.) was added in a single portion, and the flask walls were washed down with 7.0 mL distilled water. After 1 h, the resulting precipitate was collected by suction filtration over filter paper supported on a fritted funnel. The filter cake was washed successively with water, methanol, minimal dichloromethane, and diethyl ether, then dried under high vacuum to give the title compound as a black, friable solid (300 mg, 63% yield). The spectral data recorded were consistent with those previously reported.[13]

NMR Spectroscopy:

^{1}H NMR (500 MHz, DMSO-d6, 23 °C, δ): 9.10 (d, *J* = 8.1 Hz, 4H), 8.81 (d, *J* = 8.1 Hz, 4H), 8.05 (t, *J* = 8.1 Hz, 2H), 7.88 (td, *J* = 7.8, 1.4 Hz, 4H), 7.31 (dt, *J* = 5.5, 1.1 Hz, 4H), 7.15–7.22 (m, 4H).

^{13}C NMR (151 MHz, DMSO-d6, 23 °C, δ): 159.5, 154.4, 152.0, 137.8, 135.0, 128.0, 124.7, 122.8.

^{19}F NMR (376 MHz, DMSO-d6, 23 °C, δ): −70.14 (d, 711 Hz)

HRMS-ESI(m/z) calc'd for $C_{30}H_{22}N_6Os$ $[M]^{2+}$, 658.1521; found, 658.1498; deviation: −3.5 ppm.

HRMS-ESI(m/z) calc'd for PF_6 $[M]^-$, 144.9642; found, 144.9647; deviation: +3.4 ppm.

General scheme of thioxanthylium photocatalyst synthesis

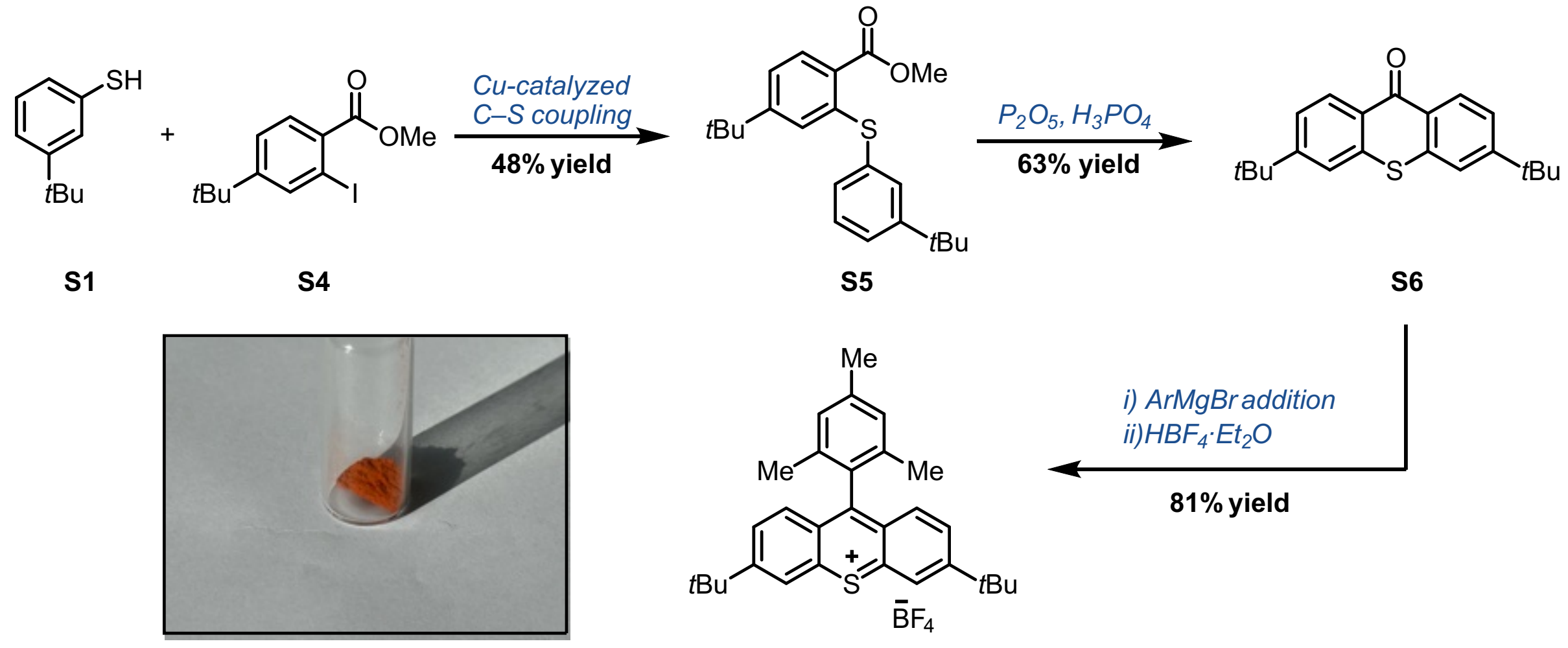

[3a] BF_4 in a 4-mL vial

[3a] BF_4

3-(*Tert*-butyl)benzenethiol (S1)

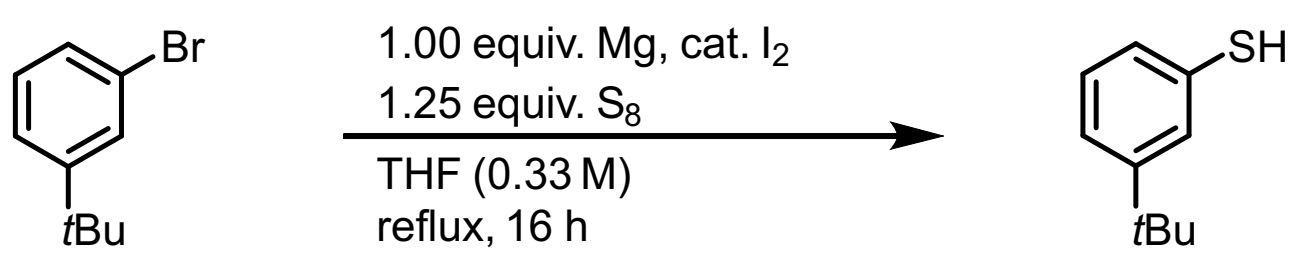

77% yield **S1**

A 250-mL two-necked round-bottomed flask fitted with a condenser and a magnetic stirrer was charged with magnesium powder (365 mg, 1.00 equiv., 15.0 mmol), dry THF (45.0 mL) and a crystal of iodine under argon pressure. Then, 1-bromo-3-(*tert*-butyl)benzene (3.21 g, 1.00 equiv., 15.0 mmol) was added dropwise over the magnesium suspension in 30 min. The reaction mixture was stirred under reflux for 1 h. The mixture was allowed to cool down to room temperature. Then an elemental sulfur (601 mg, 1.25 equiv., 18.8 mmol) was added portionwise to the mixture with the flow of argon. The reaction mixture was stirred at reflux under inert atmosphere (Ar) for 16 h. The reaction mixture was allowed to cool down to room temperature and quenched with aqueous NH_4Cl solution. This mixture was extracted with EtOAc (3×60 mL). The combined organic layer was dried over anhydrous $MgSO_4$ and concentrated by rotary evaporation under reduced pressure; the crude product was purified by flash column chromatography (silica gel, heptane/EtOAc 20:1), affording benzenethiol **S1** in 77% yield (1.92 g, 11.5 mmol) as a pale-yellow liquid with minor impurities. ^{1}H NMR spectra of the product **S1** is shown in **Figure S6**. Benzenethiol **S1** was used in the next steps without further purification.

R_f = 0.35 (heptane/EtOAc 20:1).

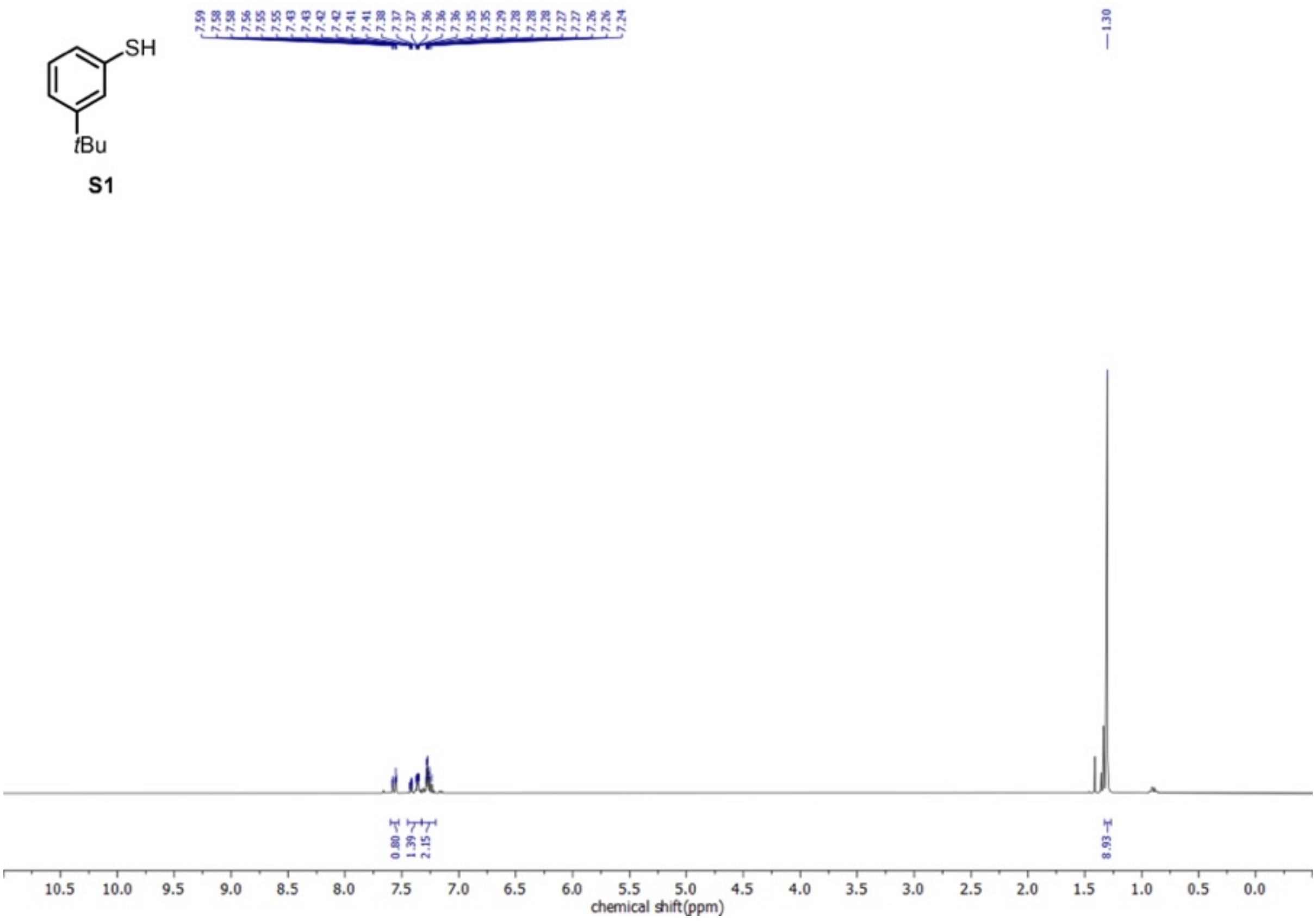

Figure S6. ^{1}H NMR (400 MHz, $CDCl_3$, 23 °C, δ) spectrum of product **S1**.

4-(*Tert*-butyl)-2-iodo-1-methylbenzene (S2)

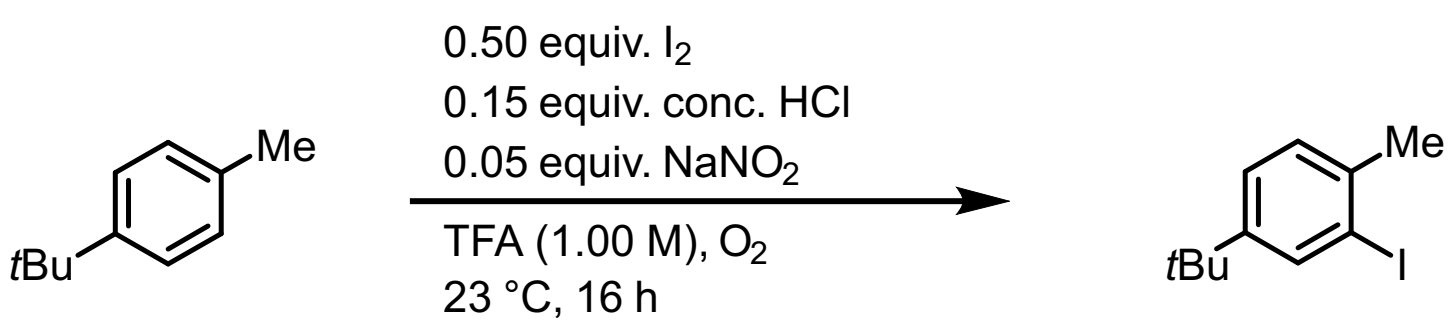

99% yield **S2**

A 250-mL round-bottom flask equipped with a magnetic stirring bar was charged with iodine (11.4 g, 0.50 equiv., 45.0 mmol) and trifluoroacetic acid (90 mL). Under stirring, concentrated HCl (37% w/w, 1.10 mL, 0.15 equiv, 13 mmol) and aqueous $NaNO_2$ (776 mg, 0.05 equiv., 4.50 mmol), and 4-*tert*-butyltoluene (15.5 mL, 1.00 equiv., 90.0 mmol) were added to the mixture. The flask was sealed with a septum, solution was sparged with an O_2 balloon for 5 min, and the reaction mixture was stirred under O_2 for 4 h. The reaction mixture was transferred to a 1-L beaker equipped with a magnetic stirring bar and quenched by slow addition of saturated sodium thiosulfate (200 mL) and saturated aqueous $NaHCO_3$ (200 mL). The mixture was extracted with CH_2Cl_2 (3×60 mL). The combined organic layer was dried over anhydrous $MgSO_4$ and concentrated by rotary evaporation under reduced pressure affording iodide **S2** in 94% yield (23.1 g, 84.3 mmol) as a yellow liquid.

NMR Spectroscopy:

^{1}H NMR (700 MHz, $CDCl_3$, 23 °C, δ): 7.80 (d, *J* = 2.0 Hz, 1H), 7.26 (dd, *J* = 2.0, *J* = 8.0 1H), 7.16

(d, J = 8.0 Hz, 1H), 2.39 (s, 3H), 1.29 (s, 9H).

^{13}C NMR (176 MHz, $CDCl_3$, 23 °C, δ): 150.9, 138.5, 136.1, 129.4, 125.5, 101.5, 34.4, 31.4, 27.6.

4-(*Tert*-butyl)-2-iodobenzoic acid (S3)

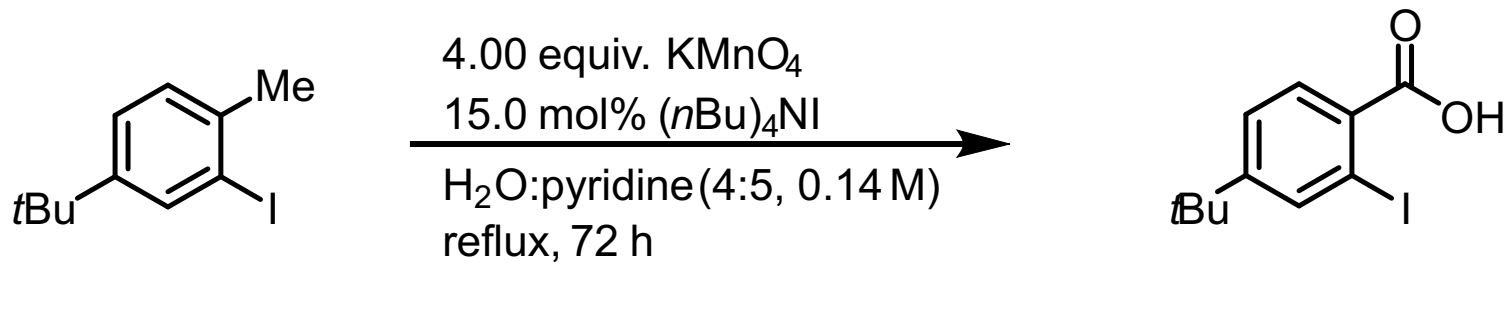

S2 **62% yield** **S3**

A 2-L round-bottom flask equipped with a magnetic stirring bar was charged with iodide **S2** (23.1 g, 1.00 equiv., 84.3 mmol), water (270 mL) and pyridine (340 mL). Potassium permanganate (53.3 g, 4.00 equiv., 337 mmol.) was added to the mixture, followed by tetrabutylammonium iodide (4.76 g, 15.0 mol%, 12.6 mmol). The reaction mixture was refluxed under vigorous stirring for 72 h. After stirring the reaction for the given time, the reaction mixture was allowed to cool down and the solids were filtered off through a short plug of celite, which was then washed with aqueous NaOH (2.0 M, 500 mL). Pyridine was removed from the filtrate by evaporation at the rotary evaporator under reduced pressure. The aqueous residue was washed with Et_2O (3×100 mL), and acidified (pH<2) by careful addition of concentrated aqueous HCl (37% w/w, ca. 100 mL). The aqueous layer was extracted with CH_2Cl_2 (3×800 mL), and the combined organic extracts were dried over $MgSO_4$, filtered, concentrated at the rotary evaporator under reduced pressure and dried under high vacuo to afford the desired iodobenzoic acid **S3** in 25% yield (8.98 g, 20.7 mmol) as an orange oil in 70% purity as estimated by ^{1}H NMR (**Figure S7**). Iodobenzoic acid **S3** was used in the next step without further purification.

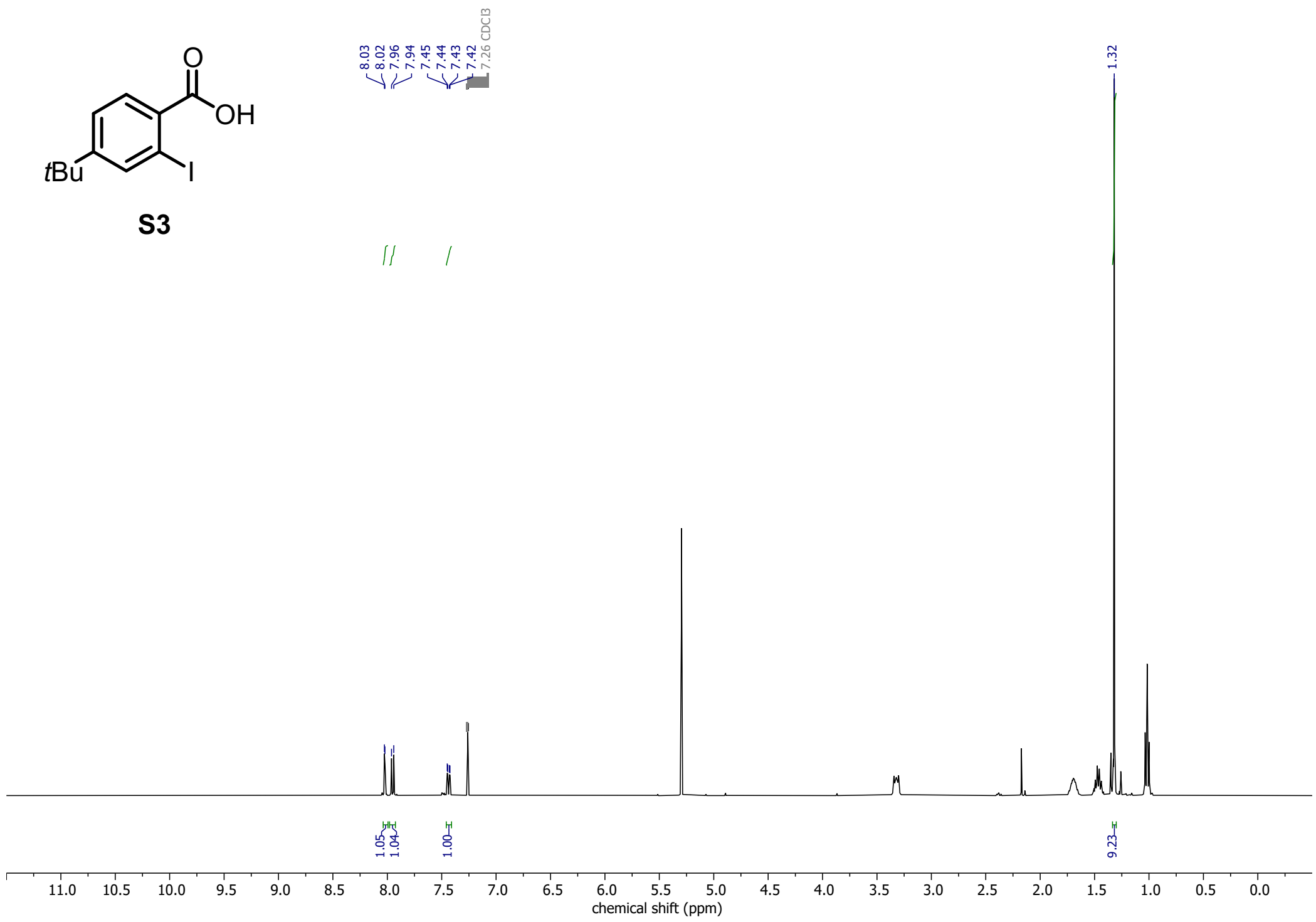

Figure S7. ^{1}H NMR (400 MHz, $CDCl_3$, 23 °C, δ) spectrum of product **S3**.

Methyl 4-(*tert*-butyl)-2-iodobenzoate (S4)

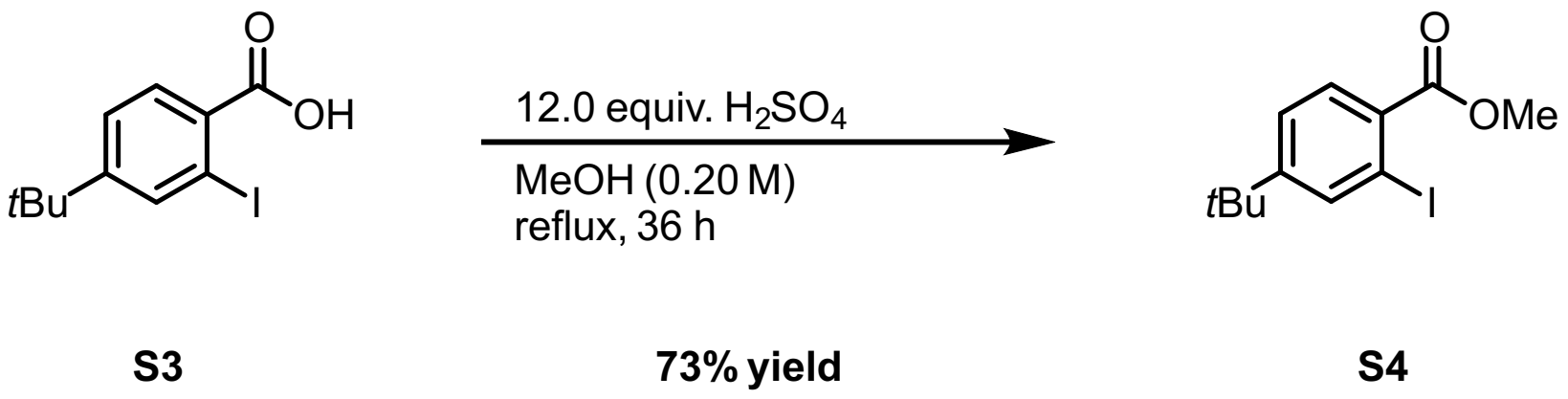

A 250-mL round-bottom flask equipped with a magnetic stirring bar and a reflux condenser was charged with iodobenzoic acid **S2** (6.08 g, 1.00 equiv., 20.0 mmol) and MeOH (100 mL). Under stirring concentrated sulfuric acid (12.8 mL, 12.0 equiv., 240 mmol) was added dropwise. The reaction mixture was stirred under reflux for 36 h. After stirring the reaction for the given time, mixture was allowed to cool down, quenched with aqueous NaOH (5.0 M, 94 mL), extracted with Et_2O (3×60 mL). The combined organic layer was dried over $MgSO_4$, filtered and concentrated at the rotary evaporator under reduced pressure. The crude product was purified by flash column chromatography (silica gel, heptane/EtOAc 20:1), affording methyl iodobenzoate **S4** in 73% yield (4.66 g, 14.6 mmol) as a colorless liquid.

R_f = 0.40 (heptane/EtOAc 20:1).

NMR Spectroscopy:

^{1}H NMR (400 MHz, $CDCl_3$, 23 °C, δ): 7.98 (d, *J* = 2.0 Hz, 1H), 7.76 (d, *J* = 8.2 Hz, 1H), 7.41 (dd, *J* = 8.3, 1.9 Hz, 1H), 3.91 (s, 3H), 1.31 (s, 9H).

^{13}C NMR (101 MHz, $CDCl_3$, 23 °C, δ): 166.9, 156.8, 138.8, 132.0, 131.0, 125.2, 94.7, 52.5, 34.9, 31.1.

HRMS-EI(m/z) calc'd for $C_{12}H_{16}IO_2S$ $[M+H]^+$, 319.0189; found, 319.0183; deviation: –1.9 ppm.

Methyl 4-(*tert*-butyl)-2-((3-(*tert*-butyl)phenyl)thio)benzoate (S5)

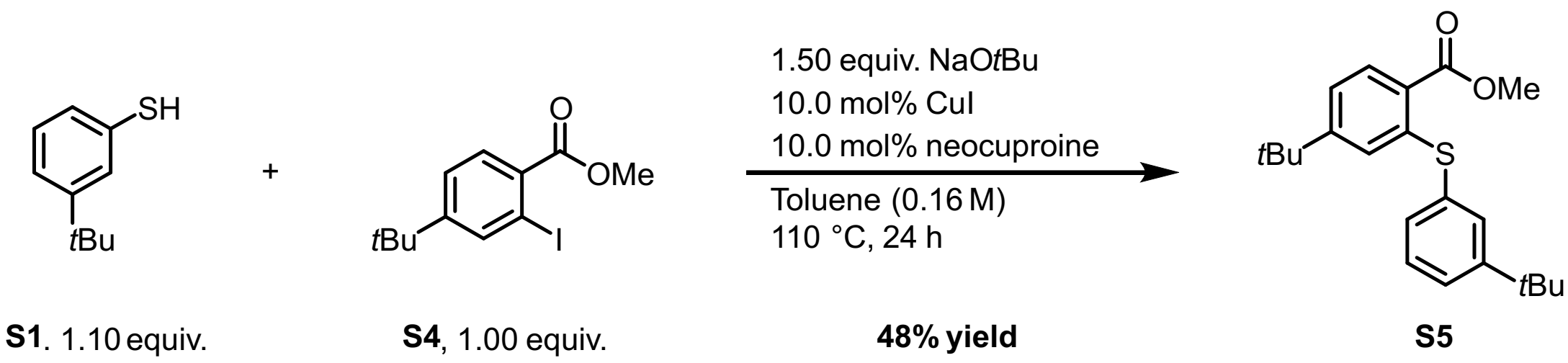

A 10-mL Schlenk tube equipped with a condenser and a magnetic stirring bar was charged with sodium *tert*-butoxide (147 mg, 1.50 equiv., 1.50 mmol), CuI (19.0 mg, 10.0 mol%, 0.10 mmol), and neocuproine (21.0 mg, 10.0 mol%, 0.10 mmol). The top condenser joint was then sealed with a rubber septum and the Schlenk tube was connected via a Tygon® tubing to a Schlenk line and evacuated and purged with argon three times. Benzenethiol **S1** (183 mg, 1.00 equiv., 1.10 mmol), methyl iodobenzoate **S4** (319 mg, 1.00 equiv., 1.00 mmol), and toluene (6.0 mL) were added into the tube via syringe through the septum. The reaction mixture was stirred at 110 °C for 24 h. After stirring the reaction for the given time,

the mixture was cooled to room temperature and filtered to remove any insoluble residues. The filtrate was concentrated in vacuo; the crude product was purified by flash column chromatography (silica gel, heptane/EtOAc 20:1), affording sulfide **S5** in 48% yield (206 mg, 0.48 mmol) as a pale-yellow liquid.

R_f = 0.35 (heptane/EtOAc 20:1).

NMR Spectroscopy:

^{1}H NMR (400 MHz, $CDCl_3$, 23 °C, δ): 7.91 (d, *J* = 8.3 Hz, 1H), 7.63 – 7.57 (m, 1H), 7.50 – 7.42 (m, 1H), 7.41 – 7.36 (m, 2H), 7.11 (dd, *J* = 8.3, 1.9 Hz, 1H), 6.76 (d, *J* = 1.9 Hz, 1H), 3.94 (s, 3H), 1.31 (s, 9H), 1.07 (s, 9H).

^{13}C NMR (101 MHz, $CDCl_3$, 23 °C, δ): 166.9, 155.7, 153.0, 143.4, 133.0, 132.7, 132.0, 131.0, 129.4, 126.2, 125.4, 124.5, 123.7, 121.4, 52.0, 34.9, 31.3, 30.7.

HRMS-EI(m/z) calc'd for $C_{22}H_{29}O_2S$ $[M+H]^+$, 357.1883; found, 357.1879; deviation: –1.1 ppm.

3,6-Di-*tert*-butyl-thioxanthen-9-one (S6)

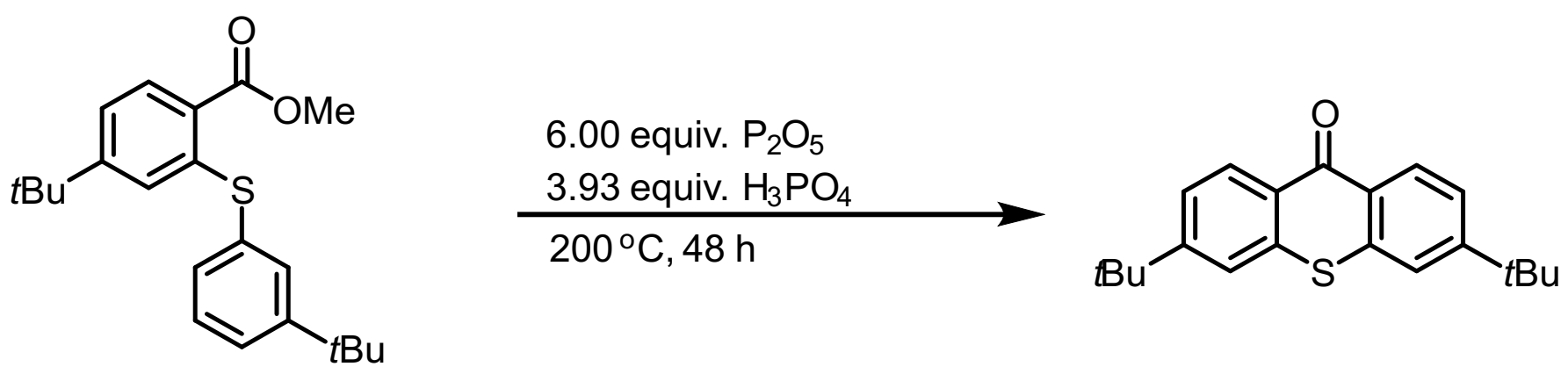

S5 **63% yield** **S6**

A 20-mL borosilicate vial equipped with a magnetic stirring bar was charged with phosphorous pentoxide (147 mg, 6.00 equiv., 1.04 mmol) and phosphoric acid (50.0 µL, 3.93 equiv., 0.680 mmol). The reaction mixture was stirred at 200 °C in an oil bath for 30 min to generate polyphosphoric acid. Then, sulfide **S5** (62.0 mg, 1.00 equiv., 0.173 mmol) was added into the reaction mixture. The reaction was stirred at 200 °C for 48 h. After stirring the reaction for the given time, the mixture was allowed to cool down to room temperature and quenched with ice and water mixture (5.0 mL). The mixture was extracted with CH_2Cl_2 (3×10 mL). The combined organic layers were dried over anhydrous Na_2SO_4 and concentrated by rotary evaporation under reduced pressure. The crude product was purified by flash column chromatography (silica gel, heptane/EtOAc 20:1), affording thioxanthenone **S6** in 63% yield (36.0 mg, 0.11 mmol) as a yellow solid.

R_f = 0.30 (heptane/EtOAc 20:1).

NMR Spectroscopy:

^{1}H NMR (400 MHz, $CDCl_3$, 23 °C, δ): δ 8.59 – 8.50 (m, 2H), 7.57 – 7.48 (m, 4H), 1.40 (s, 18H).

^{13}C NMR (101 MHz, $CDCl_3$, 23 °C, δ): 179.7, 156.1, 137.5, 129.7, 127.3, 124.4, 122.3, 35.4, 31.1.

HRMS-EI(m/z) calc'd for $C_{21}H_{25}OS$ $[M+H]^+$, 325.1621; found, 325.1617; deviation: –1.2 ppm.

3,6-Di-*tert*-butyl-9-mesitylthioxanthylium tetrafluoroborate ([3a] BF_4)

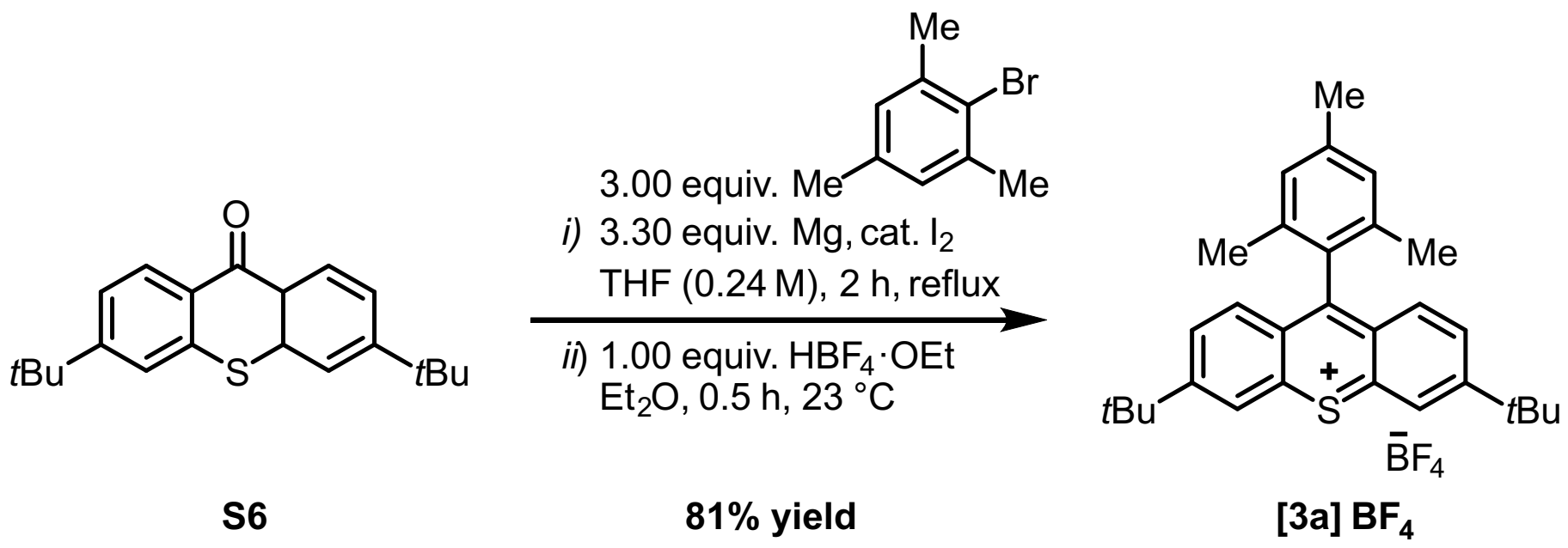

A 10-mL two-necked round-bottomed flask fitted with a condenser and a magnetic stirrer was charged with magnesium powder (93.0 mg, 3.30 equiv., 3.89 mmol), dry THF (3.0 mL) and a crystal of iodine under argon pressure. Then, 2,4,6-trimethylbromobenzene (704 mg, 540 µL, 3.00 equiv., 3.54 mmol) was added over the magnesium suspension. The reaction mixture was stirred under reflux for 2 h. The mixture was allowed to cool down to room temperature. Then a solution of thioxanthenone **S6** (383 mg, 1.00 equiv., 1.18 mmol) in THF (2.0 mL) was added. The reaction mixture was stirred at reflux under inert atmosphere (Ar) for 24 h. After that, the reaction mixture was allowed to cool down to room temperature and quenched with aqueous NH_4Cl solution. This mixture was extracted with EtOAc (3×10 mL). The combined organic layer was dried over anhydrous $MgSO_4$ and concentrated by rotary evaporation under reduced pressure. The residue was charged with Et_2O followed by $HBF_4{\cdot}Et_2O$ complex (50-55% w/w HBF_4, 210 µL, 1.00 equiv., 1.18 mmol). The mixture was stirred for 30 min at room temperature. Afterwards, the solid was filtered and washed with pentane. Then, the residue was dried under high vacuo to afford thioxanthylium tetrafluoroborate **3a [BF$_4$]** in 81% yield (493 mg, 0.96 mmol) as an orange solid.

NMR Spectroscopy:

^{1}H NMR (500 MHz, $CDCl_3$, 23 °C, δ): 8.81 (s, 2H), 7.99 – 7.94 (m, 4H), 7.16 (s, 2H), 2.49 (s, 3H), 1.73 (s, 6H), 1.53 (s, 18H).

^{13}C NMR (101 MHz, $CDCl_3$, 23 °C, δ): 170.0, 163.3, 148.8, 140.8, 135.7, 133.0, 131.5, 129.3, 127.7, 124.2, 66.2, 30.6, 21.4, 20.2, 15.3.

^{19}F NMR (376 MHz, $CDCl_3$, 23 °C, δ): −152.30, −152.35.

HRMS-EI(m/z) calc'd for $C_{30}H_{35}S$ $[M]^+$, 427.2454; found, 427.2453; deviation: –0.2 ppm.

HRMS-ESI(m/z) calc'd for BF_4 $[M]^-$, 87.0035; found, 87.0024; deviation: –12.6 ppm.

General scheme of selenoxanthylium photocatalysts synthesis

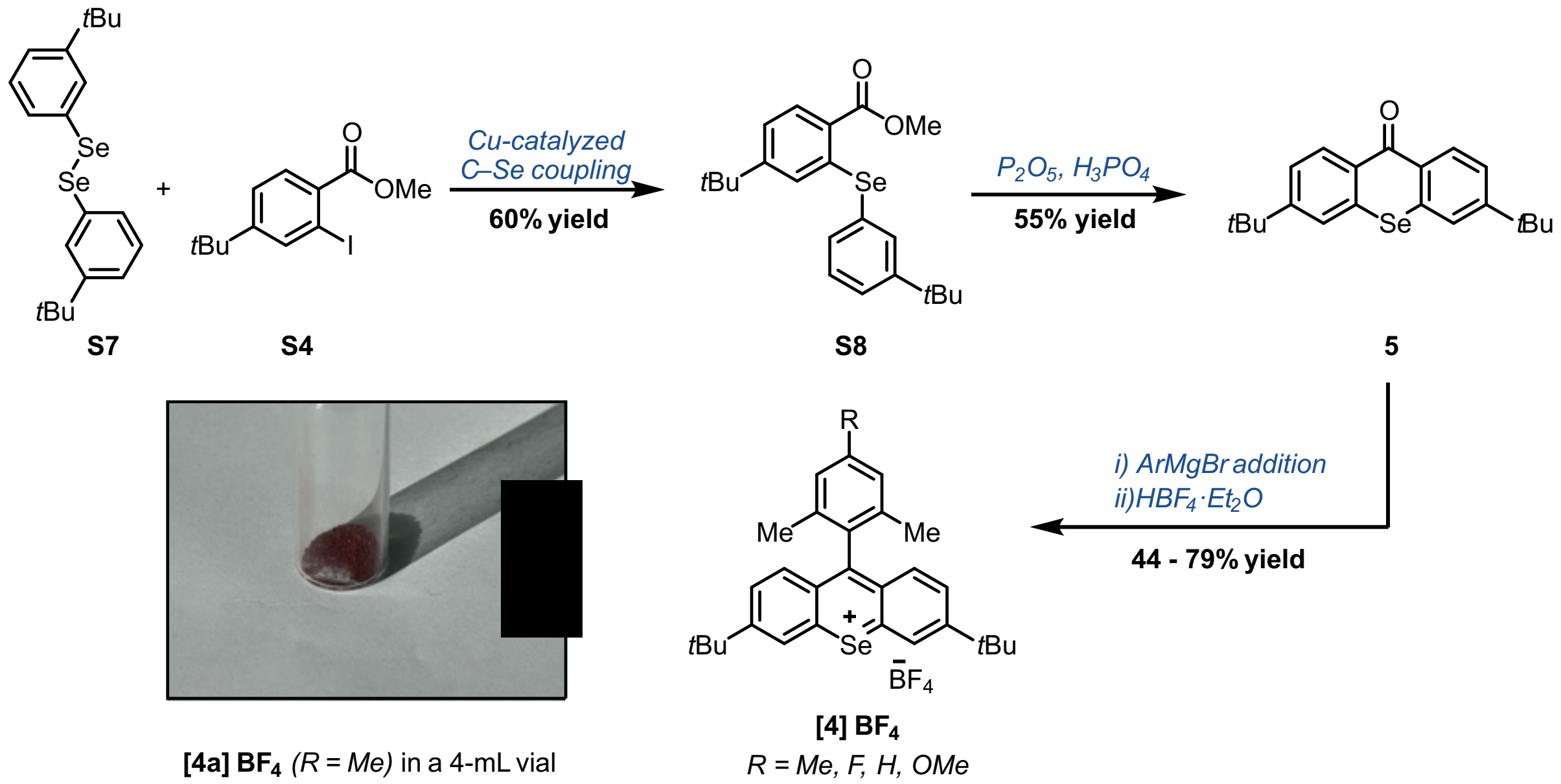

1,2-Bis(3-(*tert*-butyl)phenyl)diselane (S7)

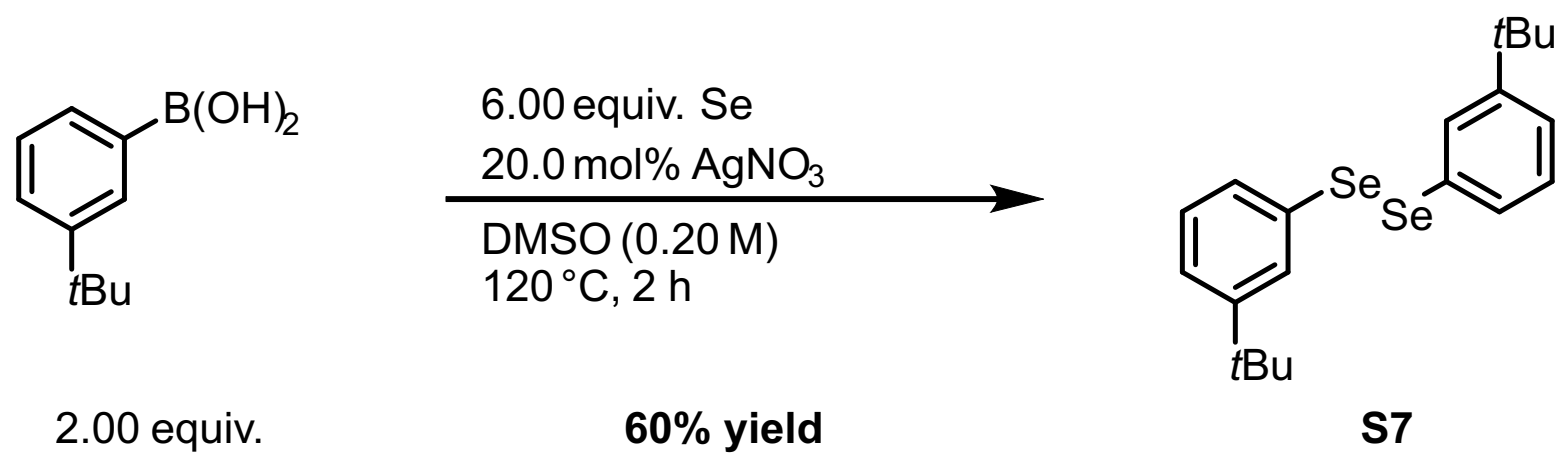

A 50 mL oven-dried round-bottom flask was charged with (3-(*tert*-butyl)phenyl)boronic acid (714 mg, 1.00 equiv., 4.00 mmol,), selenium (957 mg, 12.0 mmol, 6.00 equiv.), silver nitrate (67.9 mg, 20.0 mol%, 0.40 mmol), and DMSO) (20.0 mL). The reaction mixture was stirred at 120 °C for 2 h. After cooling to room temperature, the mixture was diluted with water (100 mL) and extracted with diethyl ether (3 × 15 mL). The combined organic layers were washed sequentially with water (30 mL) and brine (30 mL), dried over $MgSO_4$, and concentrated at the rotary evaporator under reduced pressure. The crude product was purified by flash column chromatography (silica gel, heptane/EtOAc 49:1), affording diselenide **S7** in 60% yield (507 mg, 1.20 mmol) as a yellow liquid with minor impurities of other selenium-containing products. ^{1}H and ^{77}Se NMR signals of the product **S7** are highlighted in **Figures S8** and **S9**. Diselenide **S7** was used in the next steps without further purification.

R_f = 0.35 (heptane/EtOAc 20:1).

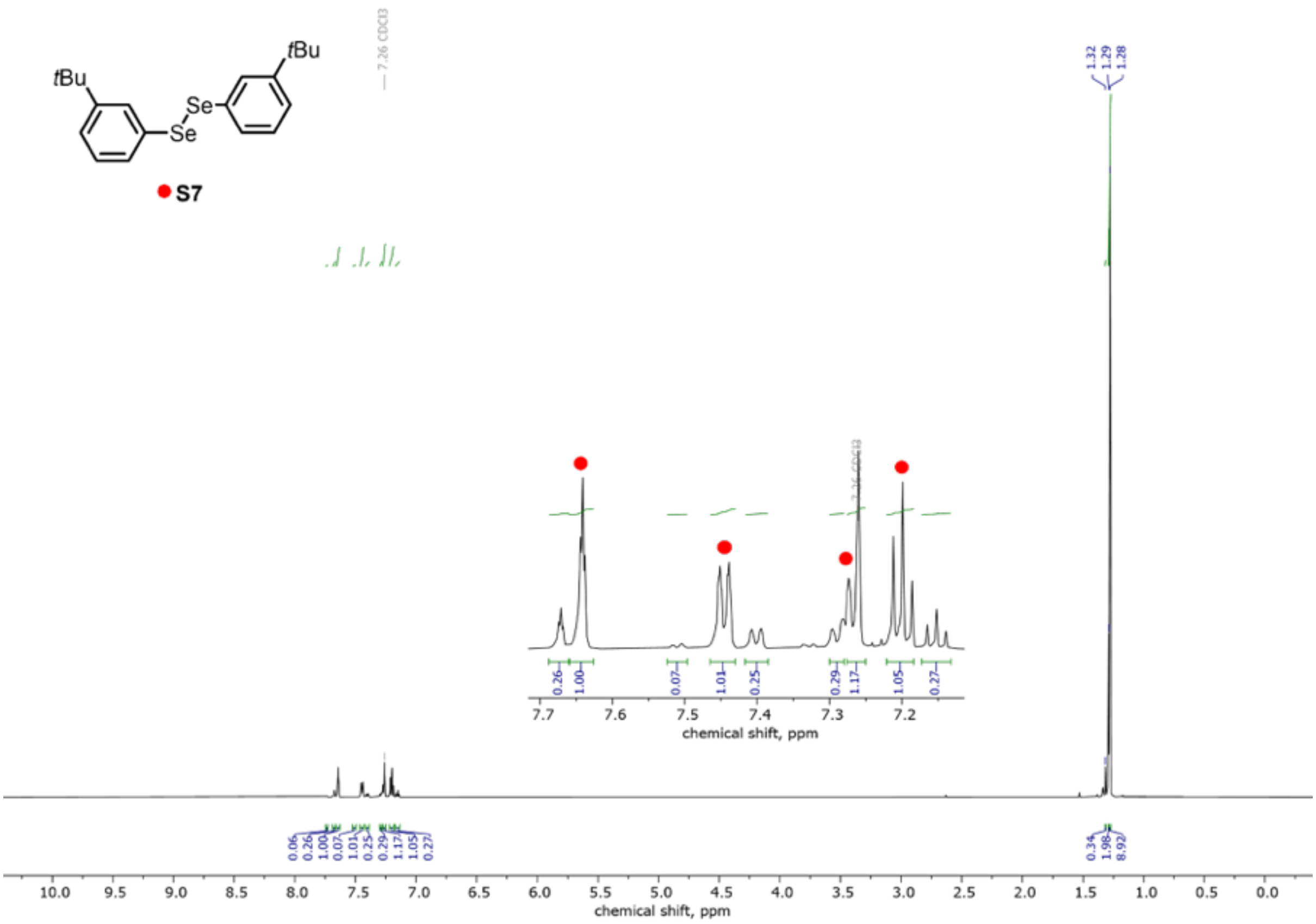

Figure S8. ^{1}H NMR (600 MHz, $CDCl_3$, 23 °C, δ) spectrum of product **S7**.

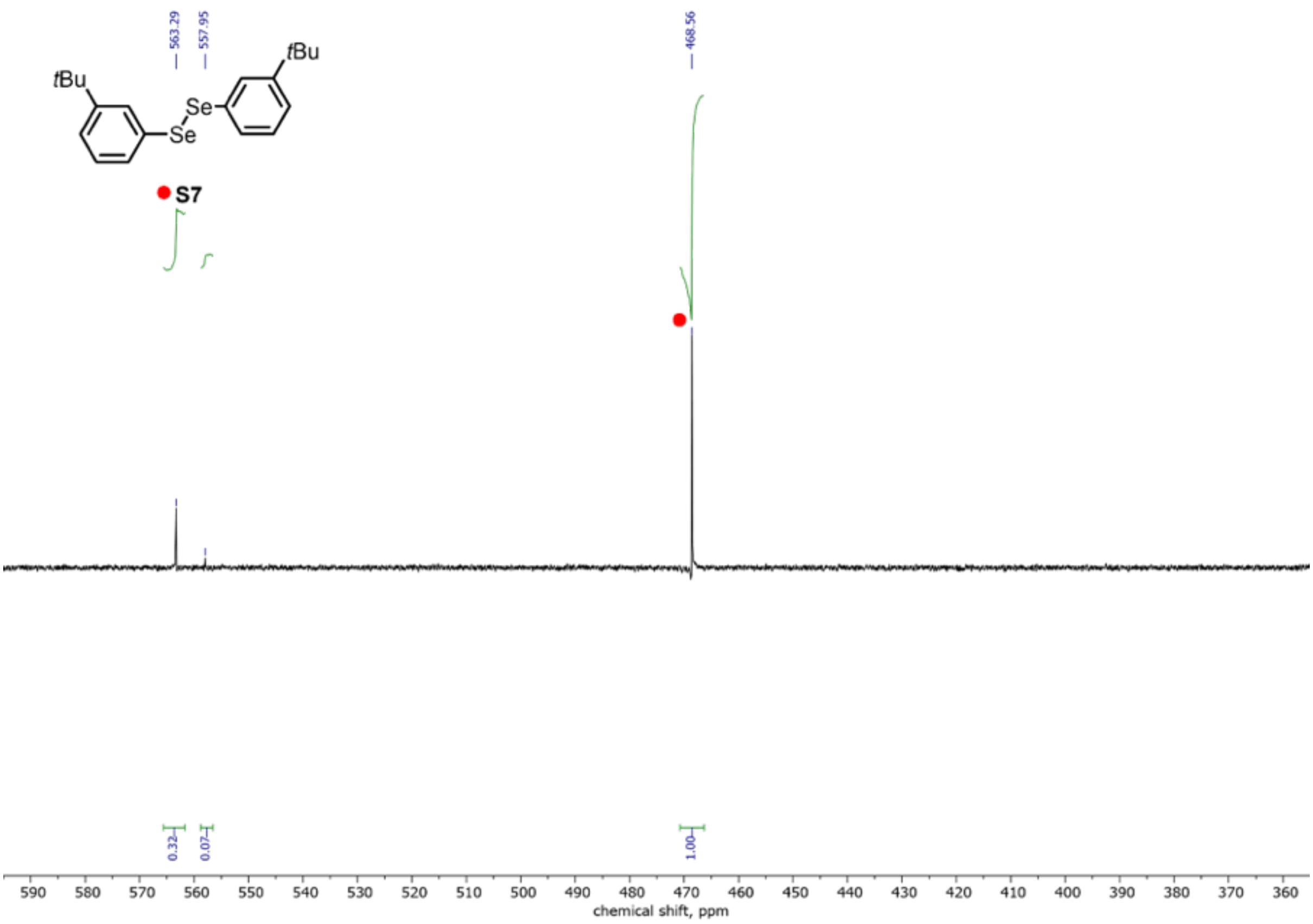

Figure S9. ^{77}Se NMR (115 MHz, $CDCl_3$, 23 °C, δ) spectrum of product **S7**.

Methyl 4-(*tert*-butyl)-2-((3-(*tert*-butyl)phenyl)selanyl)benzoate (S8)

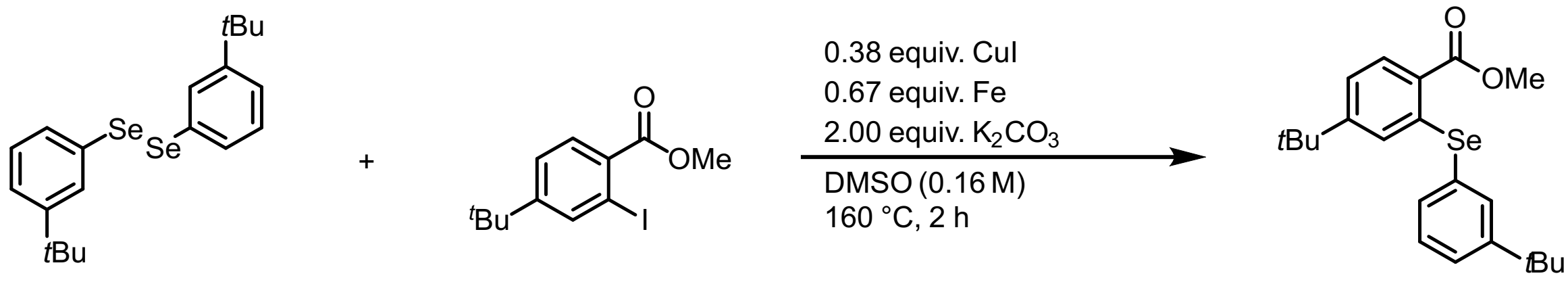

S7, 0.66 equiv. **S4**, 1.00 equiv. **60% yield** **S8**

An oven-dried 25-mL Schlenk tube equipped with a magnetic stirring bar was charged with iodobenzoate **S4** (400 mg, 1.00 equiv., 1.26 mmol), copper(I) iodide (90.0 mg, 0.38 equiv., 0.47 mmol), iron powder (48.0 mg, 0.670 equiv., 0.840 mmol), and K_2CO_3 (348 mg, 2.00 equiv., 2.51 mmol) in dry DMSO (8.0 mL) under argon pressure. With the flow of argon diselenide **S7** (352 mg, 0.66 equiv., 0.83 mol) was added portionwise into the reaction mixture. The reaction mixture was stirred at 160 °C for 2 h. After that time, the reaction mixture was allowed to cool down to room temperature and quenched with 50 mL of a saturated aqueous solution of NH_4Cl. The mixture was extracted with EtOAc (3 × 20 mL), and the organic phase was washed with brine. The combined organic layer was dried over anhydrous $MgSO_4$ and concentrated by rotary evaporation under reduced pressure. The crude product was purified by flash column chromatography (silica gel, heptane/EtOAc 20:1), affording selenide **S8** in 60% yield (302 mg, 0.749 mmol) as a yellow-pale liquid.

R_f = 0.35 (heptane/EtOAc 20:1).

NMR Spectroscopy:

^{1}H NMR (600 MHz, $CDCl_3$, 23 °C, δ): 7.96 (d, J = 8.3 Hz, 1H), 7.75 (t, J = 1.8 Hz, 1H), 7.56 – 7.52 (m, 1H), 7.49 (ddd, J = 7.9, 2.0, 1.1 Hz, 1H), 7.37 (t, J = 7.7 Hz, 1H), 7.15 (dd, J = 8.3, 1.9 Hz, 1H), 6.86 (d, J = 1.8 Hz, 1H), 3.96 (s, 3H), 1.33 (s, 9H), 1.04 (s, 9H).

^{13}C NMR (151 MHz, $CDCl_3$, 23 °C, δ): 167.4, 156.0, 153.1, 140.6, 135.0, 134.8, 131.2, 129.5, 128.8, 126.4, 126.3, 124.6, 122.0, 52.3, 35.1, 31.5, 30.8, 22.8, 14.3.

^{77}Se NMR (115 MHz, $CDCl_3$, 23 °C, δ): 470.5.

HRMS-EI(m/z) calc'd for $C_{22}H_{28}O_2SeNa$ [M+Na]$^+$, 427.1148; found, 427.1148; deviation: 0.0 ppm.

3,6-Di-*tert*-butyl-selenoxanthen-9-one (5)

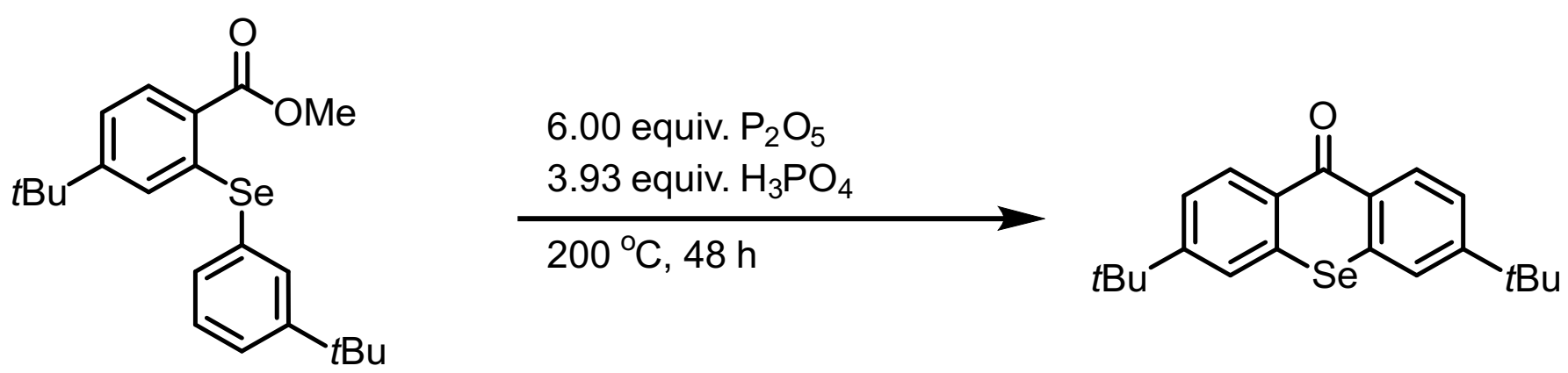

S8 **55% yield** **5**

A 20-mL borosilicate vial equipped with a magnetic stirring bar was charged with phosphorous pentoxide (639 mg, 6.00 equiv., 4.05 mmol) and phosphoric acid (200 µL, 3.93 equiv., 2.95 mmol). The reaction

mixture was stirred at 200 °C for 30 min to generate polyphosphoric acid. Then, selenide **S8** (302 mg, 1.00 equiv., 0.749 mmol) was added into the reaction mixture. The reaction was stirred at 200 °C for 48 h. After stirring the reaction for the given time, the mixture was allowed to cool down to room temperature and quenched with ice and water mixture (10 mL). The mixture was extracted with CH_2Cl_2 (3×20 mL). The combined organic layers were dried over anhydrous Na_2SO_4 and concentrated by rotary evaporation under reduced pressure. The crude product was purified by flash column chromatography (silica gel, heptane/EtOAc 20:1), affording selenoxanthenone **5** in 55% yield (152 mg, 0.409 mmol) as a yellow solid.

R_f = 0.30 (heptane/EtOAc 20:1).

NMR Spectroscopy:

^{1}H NMR (600 MHz, $CDCl_3$, 23 °C, δ): 8.57 (d, *J* = 8.5 Hz, 2H), 7.59 (d, *J* = 1.9 Hz, 2H), 7.50 (dd, *J* = 8.5, 1.9 Hz, 2H), 1.39 (s, 18H).

^{13}C NMR (151 MHz, $CDCl_3$, 23 °C, δ): 181.7, 155.9, 135.3, 131.2, 128.9, 124.8, 124.7, 35.4, 31.1.

^{77}Se NMR (115 MHz, $CDCl_3$, 23 °C, δ): 333.1.

HRMS-EI(m/z) calc'd for $C_{21}H_{24}OSeNa$ $[M+Na]^+$, 395.0886; found, 395.0887; deviation: +0.2 ppm.

3,6-Di-*tert*-butyl-9-mesitylselenoxanthylium tetrafluoroborate ([4a] BF_4)

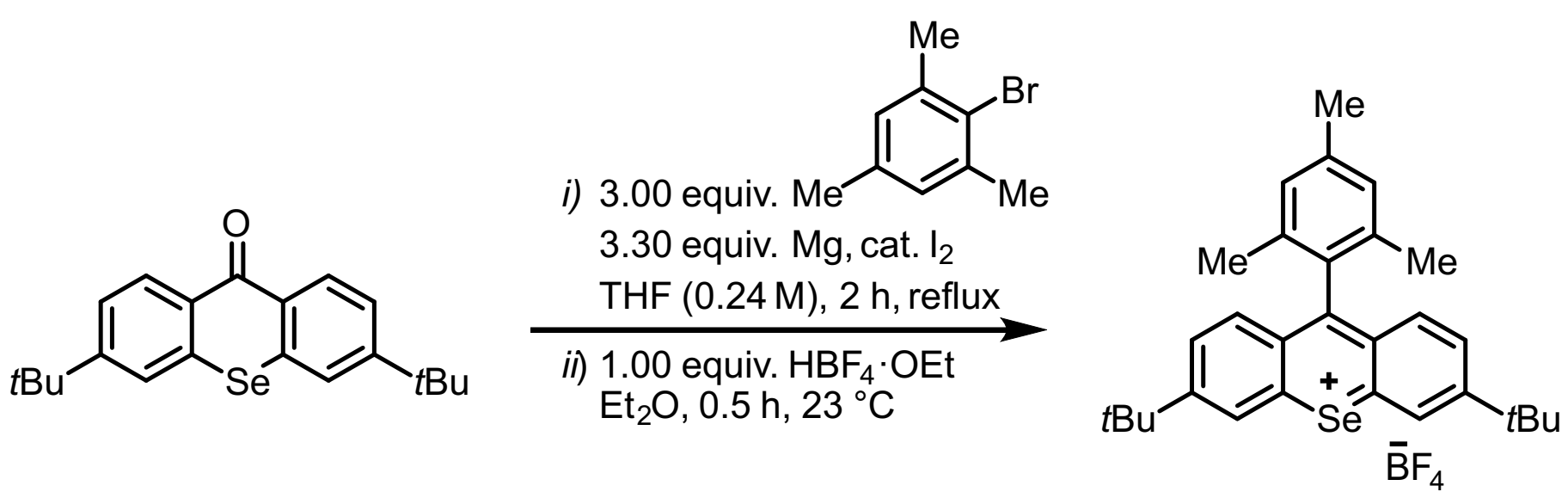

5 **79% yield** **4a [BF$_4$]**

A 10 mL two-necked round-bottomed flask fitted with a condenser and magnetic stirrer was charged with magnesium powder (31.6 mg, 3.30 equiv., 1.35 mmol), dry THF (1.5 mL) and a crystal of iodine under argon pressure. Then 2,4,6-Trimethylbromobenzene (244 mg, 180 µL, 3.00 equiv., 1.23 mmol) was added over the magnesium suspension. The reaction mixture was stirred under reflux for 2 h. The mixture was allowed to cool down to room temperature. Then a solution of selenoxanthenone **5** (152 mg, 1.00 equiv., 0.41 mmol) in THF (1 mL) was added. The reaction mixture was stirred at reflux under inert atmosphere (Ar) for 24 h. After that, the reaction mixture was allowed to cool down to room temperature and quenched with aqueous NH_4Cl solution. This mixture was extracted with EtOAc (3×10 mL). The combined organic layer was dried over anhydrous $MgSO_4$ and concentrated by rotary evaporation under reduced pressure. The residue was charged with Et_2O followed by $HBF_4·Et_2O$ complex (50-55% w/w HBF_4, 70.0 µL, 1.00 equiv., 0.41 mmol). The mixture was stirred for 30 min at

room temperature. Afterwards, the solid was filtered and washed with pentane. Then, the residue was dried under high vacuo to afford selenoxanthylium tetrafluoroborate **[4a] BF$_4$** in 79% yield (181 mg, 0.32 mmol) as a red solid.

NMR Spectroscopy:

^{1}H NMR (500 MHz, CDCl$_3$, 23 °C, δ):): 8.94 (t, *J* = 1.8 Hz, 2H), 8.09 (d, *J* = 9.2 Hz, 2H), 7.85 (dd, *J* = 9.2, 1.9 Hz, 2H), 7.14 (s, 2H), 2.48 (s, 3H), 1.71 (s, 6H), 1.51 (s, 18H).

^{13}C NMR (101 MHz, CDCl$_3$, 23 °C, δ): 171.3, 162.0, 155.1, 140.6, 135.4, 135.3, 132.8, 130.2, 129.2, 128.6, 127.3, 37.1, 30.5, 21.4, 20.2.

^{19}F NMR (377 MHz, CDCl$_3$, 23 °C δ): –151.37.

^{77}Se NMR (115 MHz, CDCl$_3$, 23 °C, δ): 710.7.

HRMS-EI(m/z) calc'd for $C_{30}H_{35}Se$ $[M]^+$, 475.1900; found, 475.1899; deviation: –0.2 ppm.

HRMS-ESI(m/z) calc'd for BF_4 $[M]^-$, 87.0024; found, 87.0035; deviation: +12.6 ppm.

3,6-Di-*tert*-butyl-9-(4-fluoro-2,6-dimethylphenyl)selenoxanthylium tetrafluoroborate ([4b] BF$_4$)

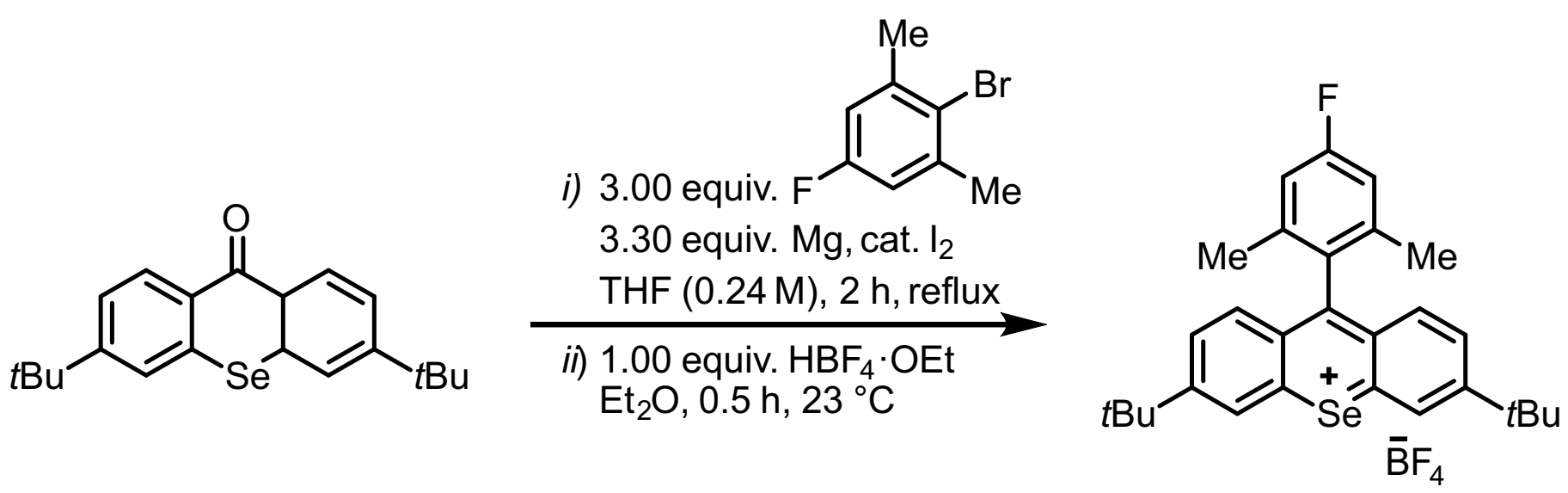

5 **58% yield** **[4b]BF$_4$**

A 10 mL 2-neck round-bottom flask fitted with a condenser and magnetic stirrer was charged with magnesium powder (25.7 mg, 3.30 equiv., 1.06 mmol), dry THF (1.5 mL) and a crystal of iodine under argon pressure. Then 2-bromo-5-fluoro-1,3-dimethylbenzene (206 mg, 140 µL, 3.00 equiv., 0.97 mmol) was added over the magnesium suspension. The reaction mixture was stirred under reflux for 2 h. The mixture was allowed to cool down to room temperature. Then a solution of selenoxanthenone **5** (120 mg, 1.00 equiv., 0.32 mmol) in THF (1 mL) was added. The reaction mixture was stirred at reflux under inert atmosphere (Ar) for 24 h. After that, the reaction mixture was allowed to cool down to room temperature and quenched with aqueous NH_4Cl solution. This mixture was extracted with EtOAc (3×10 mL). The combined organic layer was dried over anhydrous $MgSO_4$ and concentrated by rotary evaporation under reduced pressure. The residue was charged with Et_2O followed by HBF_4·Et_2O complex (50-55% w/w HBF_4, 60.0 µL, 1.00 equiv., 0.32 mmol). The mixture was stirred for 30 min at room temperature. Afterwards, the solid was filtered and washed with pentane. Then, the residue was dried under high vacuo to afford selenoxanthylium tetrafluoroborate **[4b] BF$_4$** in 58% yield (106 mg, 187 µmol) as a red solid.

NMR Spectroscopy:

^{1}H NMR (400 MHz, $CDCl_3$, 23 °C, δ): 9.00 (d, *J* = 1.9 Hz, 2H), 8.04 (d, *J* = 9.2 Hz, 2H), 7.89 (d, *J* = 1.8 Hz, 1H), 7.08 (d, *J* = 9.0 Hz, 2H), 1.77 (s, 6H), 1.53 (s, 18H).

^{13}C NMR (151 MHz, $CDCl_3$, 23 °C, δ): 168.7, 164.2, 162.6, 162.0, 155.5, 138.2, 134.6, 130.3, 128.3, 127.4, 115.5, 115.3, 53.4, 37.0, 30.4, 20.3.

^{19}F NMR (659 MHz, $CDCl_3$, 23 °C, δ): –111.37, –150.62, –150.67.

^{77}Se NMR (115 MHz, $CDCl_3$, 23 °C, δ): 721.98.

HRMS-EI(m/z) calc'd for $C_{29}H_{32}FSe$ $[M]^+$, 479.1650; found, 479.1656; deviation: +1.2 ppm.

HRMS-ESI(m/z) calc'd for BF_4 $[M]^-$, 87.0035; found, 87.0036; deviation: +1.1 ppm.

3,6-Di-*tert*-butyl-9-(2,6-dimethylphenyl)selenoxanthylium tetrafluoroborate ([4c] BF_4)

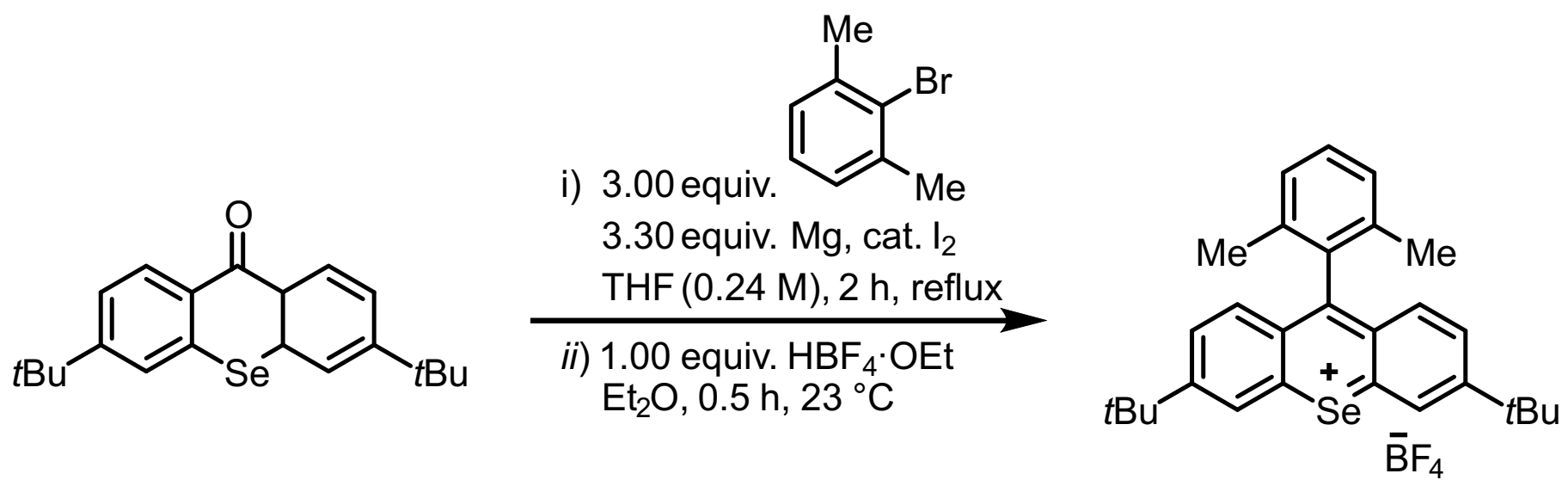

5 **55% yield** **[4c] BF_4**

A 10 mL 2-neck round-bottom flask fitted with a condenser and magnetic stirrer was charged with magnesium powder (25.7 mg, 3.30 equiv., 1.06 mmol), dry THF (1.5 mL) and a crystal of iodine under argon pressure. Then 2-bromo-1,3-dimethylbenzene (179 mg, 130 µL, 3.00 equiv., 0.97 mmol) was added over the magnesium suspension. The reaction mixture was stirred under reflux for 2 h. The mixture was allowed to cool down to room temperature. Then a solution of selenoxanthenone **5** (120 mg, 1.00 equiv., 0.32 mmol) in THF (1 mL) was added. The reaction mixture was stirred at reflux under inert atmosphere (Ar) for 24 h. After that, the reaction mixture was allowed to cool down to room temperature and quenched with aqueous NH_4Cl solution. This mixture was extracted with EtOAc (3×10 mL). The combined organic layer was dried over anhydrous $MgSO_4$ and concentrated by rotary evaporation under reduced pressure. The residue was charged with Et_2O followed by $HBF_4·Et_2O$ complex (50-55% w/w HBF_4, 60.0 µL, 1.00 equiv., 0.32 mmol). The mixture was stirred for 30 min at room temperature. Afterwards, the solid was filtered and washed with pentane. The residue was dried under high vacuo to afford selenoxanthylium tetrafluoroborate **[4c] BF_4** in 55% yield (97.0 mg, 177 µmol) as a red solid.

NMR Spectroscopy:

^{1}H NMR (500 MHz, $CDCl_3$, 23 °C, δ): 8.96 (q, *J* = 1.8 Hz, 2H), 8.05 (d, *J* = 9.2 Hz, 2H), 7.86 (dd, *J* = 9.2, 1.9 Hz, 2H), 7.52 (t, *J* = 7.7 Hz, 1H), 7.33 (d, *J* = 7.8 Hz, 2H), 1.76 (s, 6H), 1.52 (s, 18H).

^{13}C NMR (151 MHz, $CDCl_3$, 23 °C, δ): 170.4, 162.1, 155.4, 135.7, 135.6, 135.2, 130.5, 130.3, 128.5, 128.3, 127.4, 37.2, 30.5, 20.3.

^{19}**F NMR** (659 MHz, $CDCl_3$, 23 °C, δ): –150.97, –151.03.

^{77}**Se NMR** (115 MHz, $CDCl_3$, 23 °C, δ): 714.37.

HRMS-EI(m/z) calc'd for $C_{29}H_{33}Se$ $[M]^+$, 461.1744; found, 461.1742; deviation: –0.4 ppm.

HRMS-ESI(m/z) calc'd for BF_4 $[M]^-$, 87.0035; found, 87.0035; deviation: 0.0 ppm.

3,6-Di-*tert*-butyl-9-(4-methoxy-2,6-dimethylphenyl)selenoxanthylium tetrafluoroborate ([4d] BF_4)

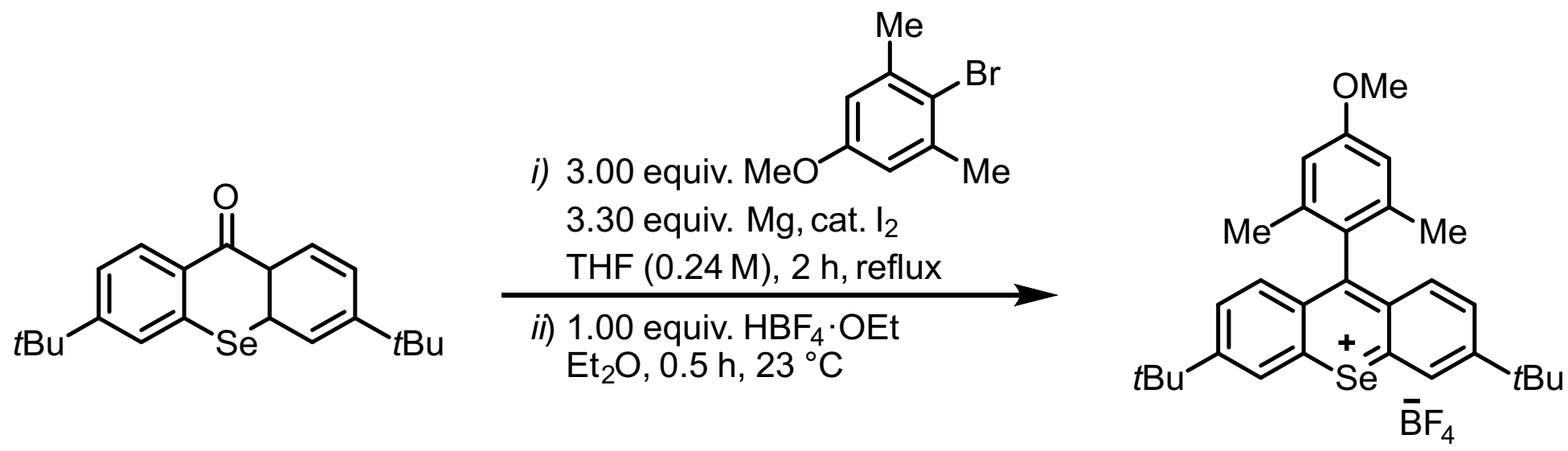

5 **44% yield** **[4d] BF_4**

A 10 mL 2-neck round-bottom flask fitted with a condenser and magnetic stirrer was charged with magnesium powder (25.7 mg, 3.30 equiv., 1.06 mmol), dry THF (1.5 mL) and a crystal of iodine under argon pressure. Then 2-bromo-5-methoxy-1,3-dimethylbenzene (208 mg, 160 µL, 3.00 equiv., 0.97 mmol)) was added over the magnesium suspension. The reaction mixture was stirred under reflux for 2 h. The mixture was allowed to cool down to room temperature. Then a solution of selenoxanthenone **5** (120 mg, 1.00 equiv., 0.32 mmol) in THF (1 mL) was added. The reaction mixture was stirred at reflux under inert atmosphere (Ar) for 24 h. After that, the reaction mixture was allowed to cool down to room temperature and quenched with aqueous NH_4Cl solution. This mixture was extracted with EtOAc (3×10 mL). The combined organic layer was dried over anhydrous $MgSO_4$ and concentrated by rotary evaporation under reduced pressure. The residue was charged with Et_2O followed by $HBF_4{\cdot}Et_2O$ complex (50-55% w/w HBF_4, 60.0 µL, 1.00 equiv., 32 µmol). The mixture was stirred for 30 min at room temperature, afterwards the solid was filtered and washed with pentane. Then, the residue was dried under high vacuo to afford selenoxanthylium tetrafluoroborate **[4d] BF_4** in 44% yield (82.0 mg, 142 µmol) as a red soild.

NMR Spectroscopy:

^{1}**H NMR** (400 MHz, $CDCl_3$, 23 °C, δ): 8.96 (d, J = 1.9 Hz, 2H), 8.11 (d, J = 9.1 Hz, 2H), 7.85 (dd, J = 9.2, 1.8 Hz, 2H), 6.87 (s, 2H), 3.95 (s, 3H), 1.73 (s, 6H), 1.52 (s, 18H).

^{13}**C NMR** (151 MHz, $CDCl_3$, 23 °C, δ): 171.0, 162.0, 161.0, 155.2, 137.2, 135.4, 130.2, 129.0, 128.0, 127.3, 113.9, 55.6, 37.1, 30.6, 20.6.

^{19}**F NMR** (377 MHz, $CDCl_3$, 23 °C, δ): –151.07.

^{77}**Se NMR** (115 MHz, $CDCl_3$, 23 °C, δ): 712.1.

HRMS-EI(m/z) calc'd for $C_{30}H_{35}Se$ $[M]^+$, 491.1850; found, 491.1848; deviation: –0.4 ppm.

HRMS-ESI(m/z) calc'd for BF_4 $[M]^-$, 87.0035; found, 87.0035; deviation: 0.0 ppm.

Catalytic properties of xanthylium salts

[4+2]-Cycloaddition reaction catalyzed by acridinium and xanthylium salts

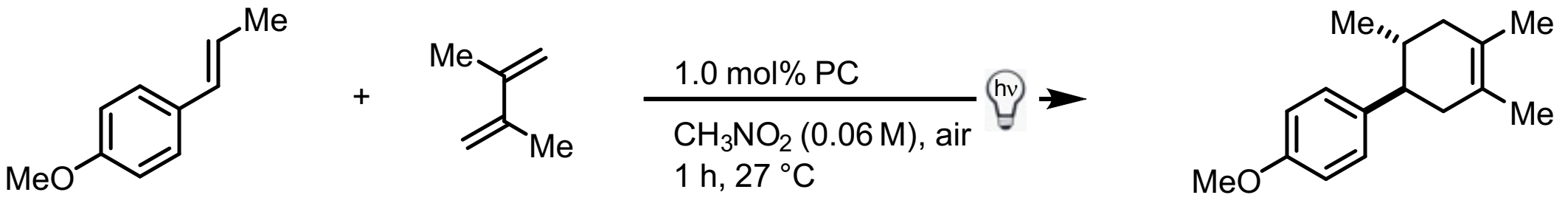

7 **8**, 3.00 equiv. **9**

Tested photocatalysts:

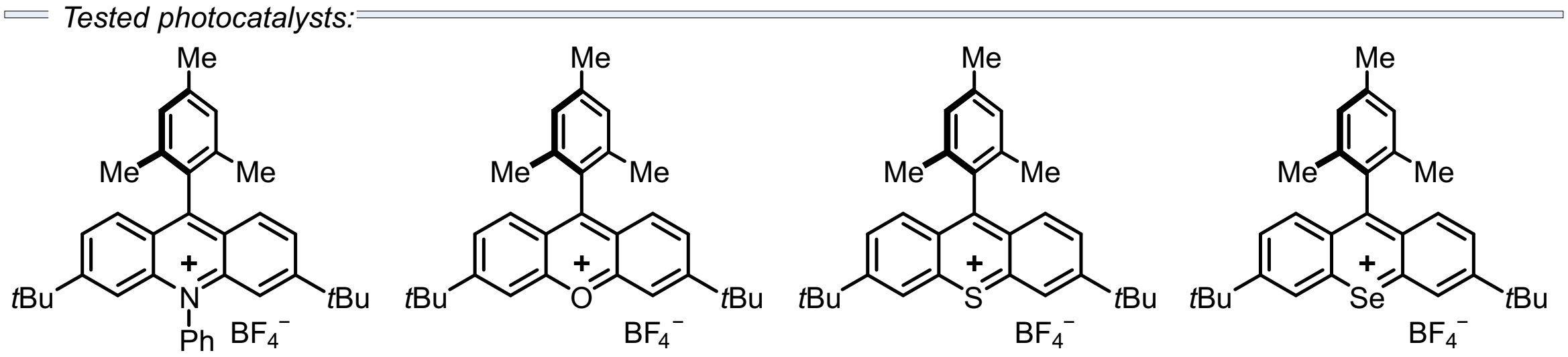

[1a] BF$_4$ **[2a] BF$_4$** **[3a] BF$_4$** **[4a] BF$_4$**

Oxidation potential of **7**: E_{ox} = 1.10V vs SCE[14]

General procedure for the [4+2]-cycloaddition reaction at 0.10 mmol scale

Under an ambient atmosphere, to a 4-mL borosilicate scintillation vial equipped with a magnetic stir bar, trans-anethol **7** (14.8 mg, 15.0 µL, 1.00 equiv. 0.10 mmol), the photocatalyst (1.0 mol%) and CH_3NO_2 (1.6 mL) were added. Then butadiene **8** (35.0 µL, 3.00 equiv., 0.30 mmol) was added to the stirring mixture. The resulting solution was irradiated with the selected irradiation wavelength while stirred. The reaction was carried out at 27 °C for 1 h. After stirring the reaction for the given time, the reaction mixture was concentrated by rotary evaporation under reduced pressure. The residue was fully solubilized in $CDCl_3$ (0.50 mL) and transferred to a NMR tube. CH_2Br_2 (7.1 µL, 0.10 mmol = theoretical maximum yield) was added as internal standard. The yield was determined by ^{1}H NMR spectroscopy using CH_2Br_2 as internal standard by comparing the integration of the CH_2Br_2 signal at 4.93 ppm (s, 2H) to the signal of product **9** at 7.09 ppm (d, 2H).

Photocatalyst comparison tables

Table S1. [4+2]-cycloaddition reaction using **[1a] BF$_4$** as a photocatalyst.

Entry	PC loading, mol%	solvent	wavelength, nm	yield **9*** (%)
1	1.00	CH_3NO_2	365	28
2	1.00	CH_3NO_2	405	>95
3	1.00	CH_3NO_2	450	21
4	1.00	CH_3NO_2	525	n.r.
5	1.00	CH_3NO_2	595	n.r.
6	1.00	CH_3NO_2	650	n.r.
7	1.00	CH_3NO_2	-	n.r.

* *The yield was determined by ^{1}H NMR spectroscopy using CH_2Br_2 as internal standard.*

Table S2. [4+2]-cycloaddition reaction using **[2a] BF$_4$** as a photocataust.

Entry	PC loading, mol%	solvent	wavelength, nm	yield **9*** (%)
1	1.00	CH_3NO_2	365	40
2	1.00	CH_3NO_2	405	>95
3	1.00	CH_3NO_2	450	>95
4	1.00	CH_3NO_2	525	26
5	1.00	CH_3NO_2	595	n.r.
6	1.00	CH_3NO_2	650	n.r.
7	1.00	CH_3NO_2	-	n.r.

* *The yield was determined by ^{1}H NMR spectroscopy using CH_2Br_2 as internal standard.*

Table S3. [4+2]-cycloaddition reaction using **[3a] BF$_4$** as a photocatalyst.

Entry	PC loading, mol%	solvent	wavelength, nm	yield **9*** (%)
1	1.00	CH_3NO_2	365	41
2	1.00	CH_3NO_2	405	>95
3	1.00	CH_3NO_2	450	>95
4	1.00	CH_3NO_2	525	26
5	1.00	CH_3NO_2	595	28
6	1.00	CH_3NO_2	650	7
7	1.00	CH_3NO_2	-	n.r.

* *The yield was determined by ^{1}H NMR spectroscopy using CH_2Br_2 as internal standard.*

Table S4. [4+2]-cycloaddition reaction using **[4a] BF$_4$** as a photocatalyst.

Entry	PC loading, mol%	solvent	wavelength, nm	yield **9*** (%)
1	1.00	CH_3NO_2	365	66
2	1.00	CH_3NO_2	405	>95
3	1.00	CH_3NO_2	450	>95
4	1.00	CH_3NO_2	525	>95
5	1.00	CH_3NO_2	595	>95
6	1.00	CH_3NO_2	650	93
7	1.00	CH_3NO_2	-	n.r.

** The yield was determined by ^{1}H NMR spectroscopy using CH_2Br_2 as internal standard.*

Procedure for the [4+2]-cycloaddition reaction at a 0.50 mmol scale

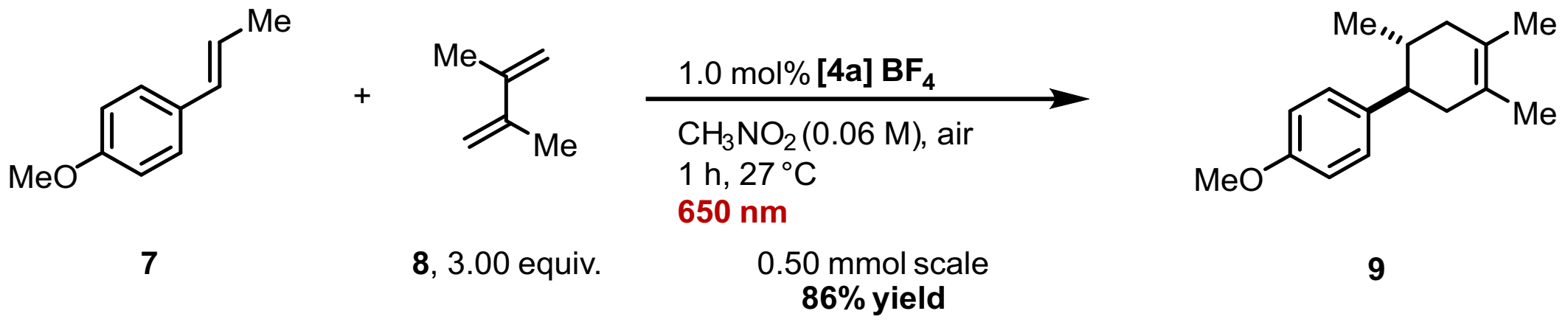

Under an ambient atmosphere, to a 20-mL borosilicate vial equipped with a magnetic stir bar, trans-anethol **7** (74.0 mg, 75.0 µL, 1.00 equiv., 0.50 mmol), photocatalyst **[4a] BF$_4$** (2.8 mg, 1.0 mol%, 5.0 µmol) and CH_3NO_2 (8.0 mL) were added. Then butadiene **8** (175 µL, 3.00 equiv., 1.50 mmol) was added to the stirring mixture. The resulting solution was irradiated with red-light (red-light irradiation, λ_{max} = 650 nm) while stirred. The reaction was carried out at 27 °C for 1 h. After stirring the reaction for the given time, the reaction mixture was concentrated by rotary evaporation under reduced pressure. The crude product was purified by flash column chromatography (silica gel, heptane/EtOAc 40:1), affording product **9** in 86% yield (98.0 mg, 0.43 mmol) as a colorless liquid.

R_f = 0.50 (heptane/EtOAc 20:1).

NMR Spectroscopy:

^{1}H NMR (400 MHz, $CDCl_3$, 23 °C, δ): 7.09 (d, *J* = 8.6 Hz, 2H), 6.84 (d, *J* = 8.7 Hz, 2H), 3.80 (s, 3H), 2.34 (td, *J* = 10.4, 5.8 Hz, 1H), 2.22 – 2.00 (m, 3H), 1.91 – 1.78 (m, 2H), 1.68 – 1.63 (m, 3H), 1.62 (s, 3H), 0.70 (d, *J* = 6.1 Hz, 3H).

^{13}C NMR (101 MHz, $CDCl_3$, 23 °C, δ): 157.9, 138.3, 128.6, 125.6, 125.5, 113.8, 55.4, 48.0, 42.0, 41.8, 34.4, 20.2, 18.9, 18.8.

HRMS-ESI(m/z) calc'd for $C_{16}H_{21}O$ [M–H]$^-$, 229.1587; found, 229.1586; deviation: –0.4 ppm

Procedure for the [4+2]-cycloaddition reaction at a 1.00 mmol scale

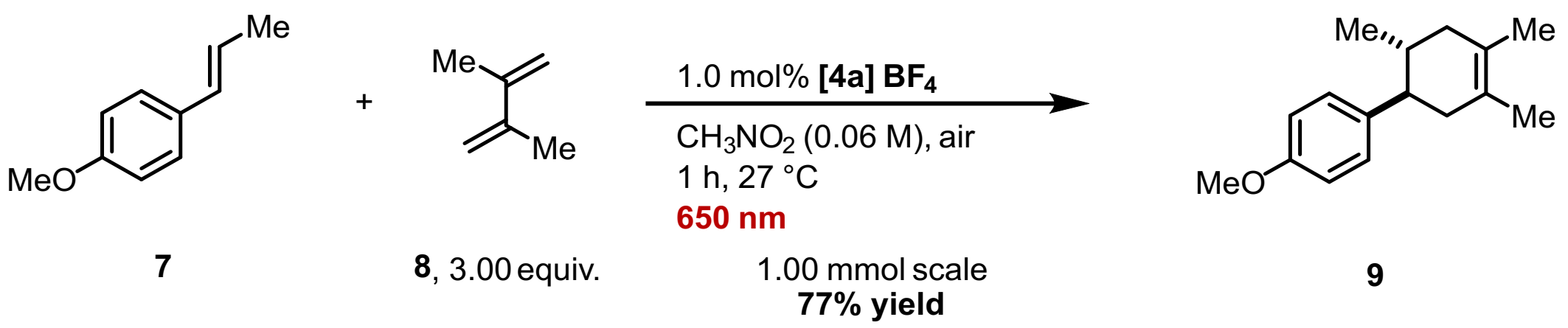

Under an ambient atmosphere, to a 50-mL round-bottom flask equipped with a magnetic stir bar, trans-anethol **7** (148 mg, 150 µL, 1.00 equiv., 1.00 mmol), photocatalyst **[4a] BF$_4$** (5.6 mg, 1.0 mol%, 10.0 µmol) and CH_3NO_2 (16.0 mL) were added. Then butadiene **8** (350 µL, 3.00 equiv., 3.00 mmol) was added dropwise via 1.00 mL syringe to the stirring mixture. The resulting solution was irradiated with red-light (red-light irradiation, λ_{max} = 650 nm) while stirred. The reaction was carried out at 27 °C for 1 h. After stirring the reaction for the given time, the reaction mixture was concentrated by rotary evaporation under reduced pressure. The crude product was purified by flash column chromatography

(silica gel, heptane/EtOAc40:1), affording product **9** in 77% yield (177 mg, 0.77 mmol) as a colorless liquid.

R_f = 0.50 (heptane/EtOAc 20:1).

Procedure for the [4+2]-cycloaddition reaction at a 5.00 mmol scale

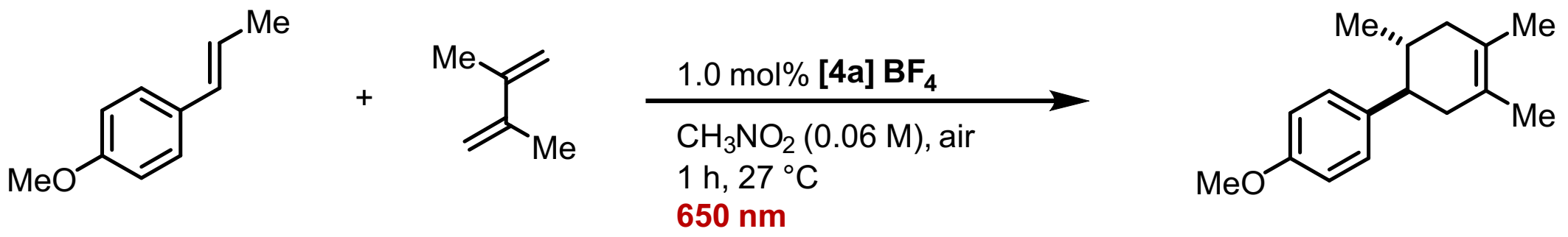

7 | **8**, 3.00 equiv. | 5.00 mmol scale **75% yield** | **9**

Under an ambient atmosphere, to a 250-mL round-bottom flask equipped with a magnetic stir bar, trans-anethol **7** (741 mg, 750 μL, 1.00 equiv., 5.00 mmol), photocatalyst **[4a] BF$_4$** (28.1 mg, 1.0 mol%, 0.05 mmol), and CH_3NO_2 (80 mL) were added. Then butadiene **8** (1.73 mL, 3.00 equiv., 0.30 mmol) was added dropwise via a 2.0 mL syringe to the stirring mixture. The resulting solution was irradiated with red-light (red-light irradiation, λ_{max} = 650 nm) while stirred. The reaction was carried out at 27 °C for 1 h. After stirring the reaction for the given time, the reaction mixture was concentrated by rotary evaporation under reduced pressure. The crude product was purified by flash column chromatography (silica gel, heptane/EtOAc 40:1), affording product **9** in 75% yield (865 mg, 3.76 mmol) as a colorless liquid.

R_f = 0.50 (heptane/EtOAc 20:1).

C–H amination reaction catalyzed by selenoxanthylium salts

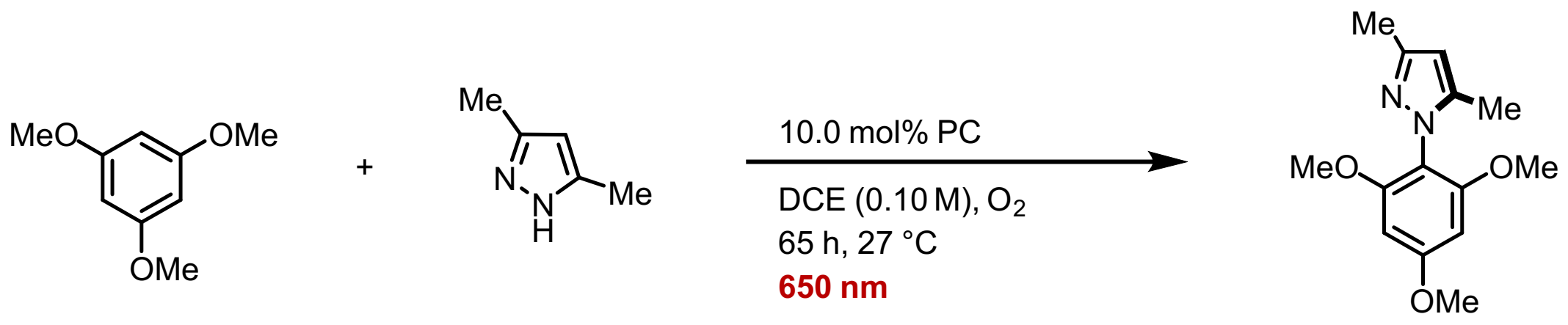

10 **11**, 5.00 equiv. **12**

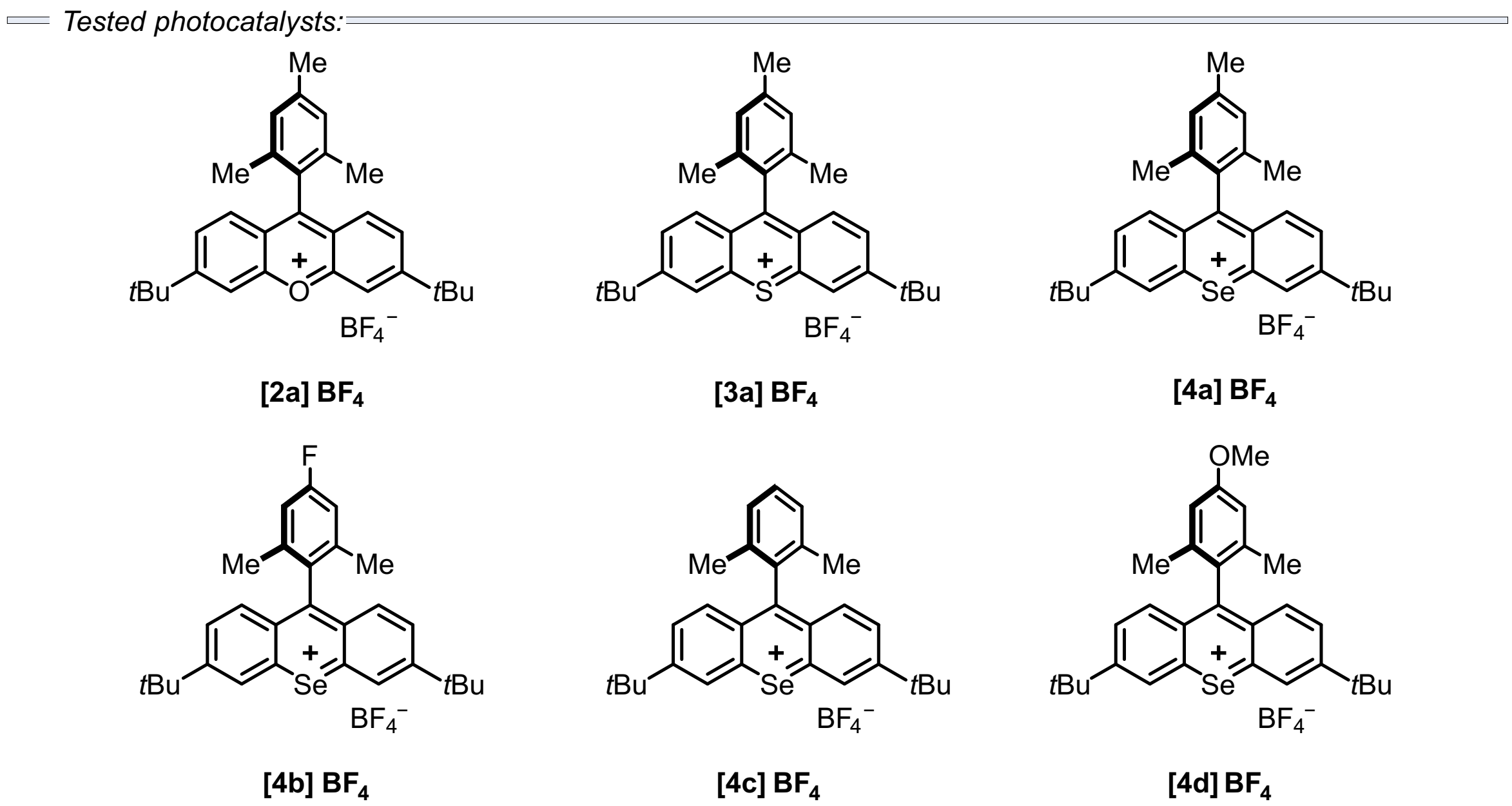

Oxidation potential of **10**:

E_{ox} = 1.54V vs SCE[15]

General procedure for the C–H amination reaction at a 0.10 mmol scale

To a 4-mL borosilicate vial equipped with a magnetic stir bar, 1,3,5-trimethoxybenzene (**10**) (17.0 mg, 1.00 equiv., 0.100 mmol), 3,5-dimethylpyrazole (**11**) (49.0 mg, 5.00 equiv., 0.50 mmol), photocatalyst (10.0 mol%, 0.01 mmol), and dry DCE (1.0 mL) were added. The vial was sealed with a cap equipped with a septum. The solution was sparged with an O_2 balloon for 5 min. After sparging, the vent needle was removed, and the O_2 balloon was maintained. The resulting solution was irradiated with red-light (red-light irradiation, λ_{max} = 650 nm) while stirred. The reaction was carried out at 27 °C for 65 h. After stirring the reaction for the given time, the reaction mixture was concentrated by rotary evaporation under reduced pressure. The residue was fully solubilized in $CDCl_3$ (0.50 mL), transferred to a NMR tube. CH_2Br_2 (7.1 µL, 0.10 mmol = theoretical maximum yield) was added as internal standard, and the yield was determined by ^{1}H NMR spectroscopy using CH_2Br_2 as internal standard by comparing the integration of the CH_2Br_2 signal at 4.89 ppm (s, 2H) to the signal of **12** at 3.82 ppm (s, 3H).

Table S5. C–H amination reaction using different xanthylium photocatalysts.

PC	[2a] BF_4	[3a] BF_4	[4a] BF_4	[4b] BF_4	[4c] BF_4	[4d] BF_4
yield* (%)	n.r.	23	87	80	62	92

** The yield was determined by ^{1}H NMR spectroscopy using CH_2Br_2 as internal standard.*

Procedure for the C–H amination reaction at a 1.00 mmol scale

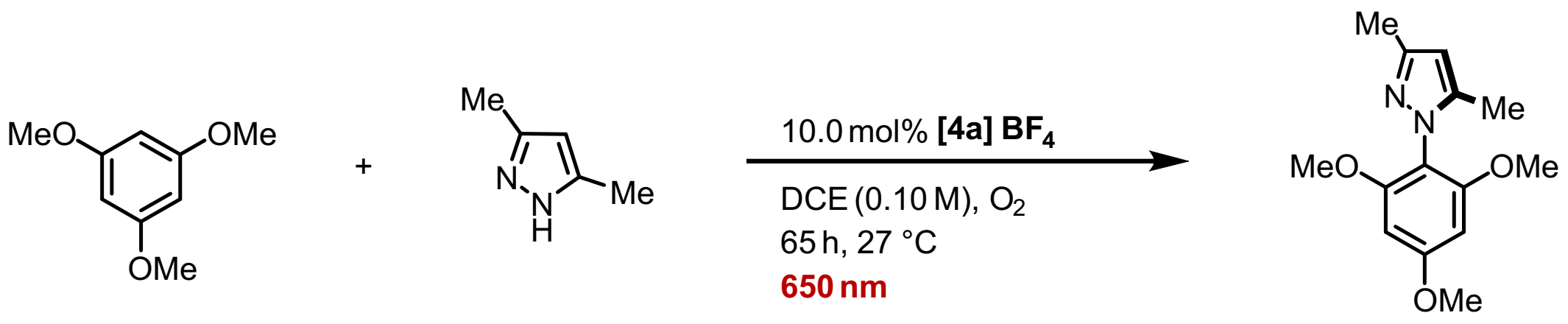

10 | **11**, 5.00 equiv. | 1.00 mmol scale **85% yield** | **12**

To a 20-mL borosilicate vial equipped with a magnetic stir bar,1,3,5-trimethoxybenzene (**10**) (170 mg, 1.00 equiv., 1 mmol), 3,5-dimethylpyrazole (**11**) (490 mg, 5.00 equiv., 5.00 mmol), photocatalyst **[4a] BF_4** (56.1 mg, 10.0 mol%, 0.1 mmol) and dry CH_2Cl_2 (10 mL) were added. The vial was sealed with a septum-lined cap, and the solution was sparged with an O_2 balloon for 5 min. After sparging, the vent needle was removed, and the O_2 balloon was maintained. The resulting solution was irradiated with red-light (red-light irradiation, λ_{max} = 650 nm) while stirred. The reaction was carried out at 27 °C for 65 h. After stirring the reaction for the given time, the mixture was concentrated by rotary evaporation under reduced pressure. The crude product was purified by flash column chromatography (silica gel, heptane/EtOAc 40:1), affording product **12** in 85% yield (152 mg, 0.409 mmol) as a colorless liquid.

R_f = 0.30 (heptane/EtOAc 5:4).

NMR Spectroscopy:

^{1}H NMR (400 MHz, CD_3CN, 23 ºC, δ): 6.16 (d, *J* = 1.9 Hz, 2H), 5.93 (s, 1H), 3.82 (d, *J* = 2.3 Hz, 2H), 3.71 (d, *J* = 2.3 Hz, 6H), 2.28 (d, *J* = 2.0 Hz, 3H), 1.98 (d, *J* = 2.3 Hz, 3H).

^{13}C NMR (101 MHz, CD_3CN, 23 ºC, δ): 161.4, 157.8, 148.71, 148.70, 141.9, 110.7, 104.4, 90.7, 56.1, 55.5, 13.9, 10.9.

HRMS-ESI(m/z) calc'd for $C_{14}H_{18}N_2O_3$ $[M+H]^+$, 263.1390; found, 263.1385; deviation: –1.9 ppm.

[2+2]-Cycloaddition reaction catalyzed by acridinium and xanthylium salts

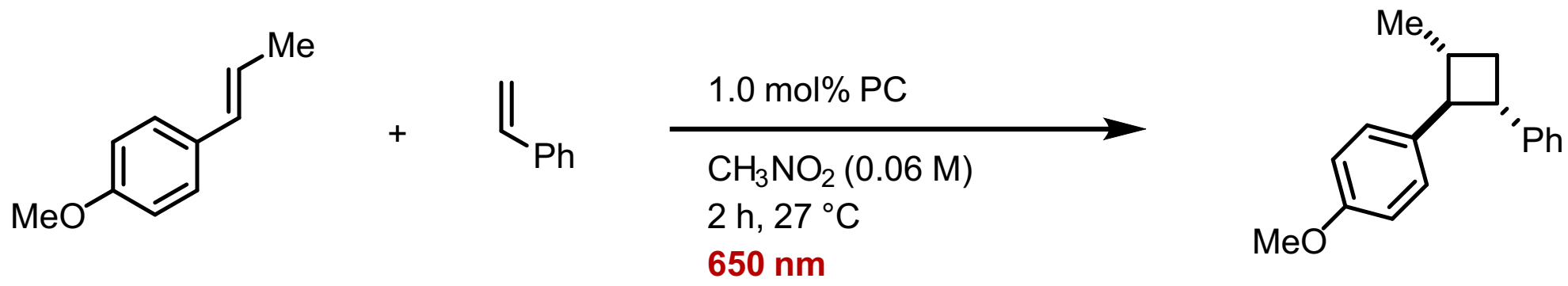

7 **13** **14**

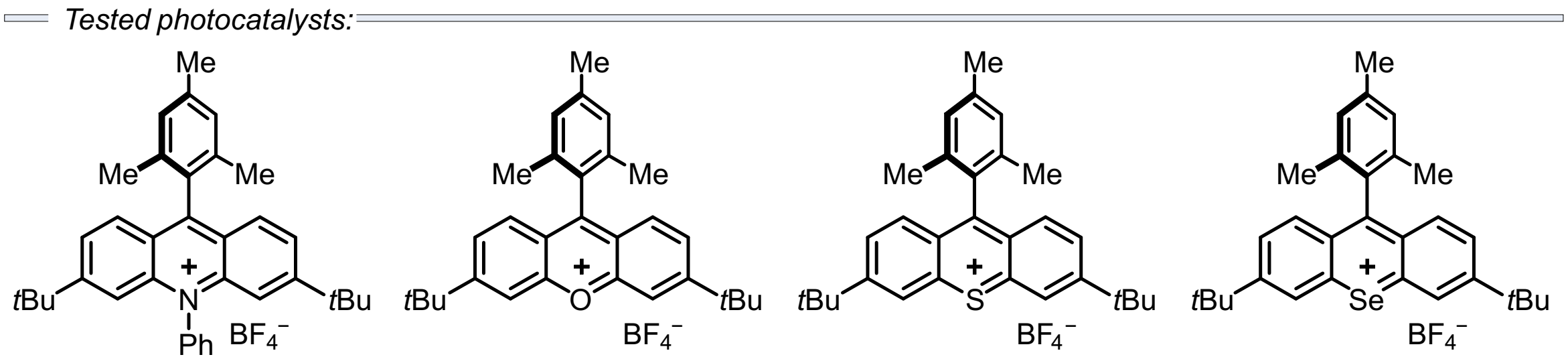

[1a] BF$_4$ **[2a] BF$_4$** **[3a] BF$_4$** **[4a] BF$_4$**

Oxidation potential of **7**: E_{ox} = 1.10V vs SCE[14]

Procedure for the [2+2]-cycloaddition reaction at a 0.10 mmol scale

Under an ambient atmosphere, to a 4-mL borosilicate vial equipped with a magnetic stir bar, trans-anethol **7** (14.8 mg, 15.0 µL, 1.00 equiv., 0.10 mmol), photocatalyst (1.0 mol%) and CH_3NO_2 (1.6 mL) were added. Then styrene (**13**) (12.0 µL, 1.00 equiv., 0.10 mmol) was added to the stirring mixture. The resulting solution was stirred in the photoreactor (red LED irradiation (λ max = 650 nm)) at room temperature under air for 2 h. After stirring the reaction for the given time, the mixture was concentrated by rotary evaporation under reduced pressure. The residue was fully solubilized in $CDCl_3$ (0.50 mL), transferred to the NMR tube, CH_2Br_2 (7.1 µL, 0.10 mmol = theoretical maximum yield) was added as internal standard, and the yield was determined by ^{1}H NMR spectroscopy using CH_2Br_2 as internal standard by comparing the integration of the CH_2Br_2 signal at 4.93 ppm (s, 2H) to the signal of product **14** at 2.98 ppm (t, 1H).

Table S6. [2+2]-Cycloaddition reaction using different photocatalysts.

PC	**[1a]BF$_4$**	**[2a]BF$_4$**	**[3a]BF$_4$**	**[4a]BF$_4$**
yield* (%)	n.r.	<5	8	90

** The yield was determined by ^{1}H NMR spectroscopy using CH_2Br_2 as internal standard.*

Procedure for the [2+2]-cycloaddition reaction at a 1.00 mmol scale

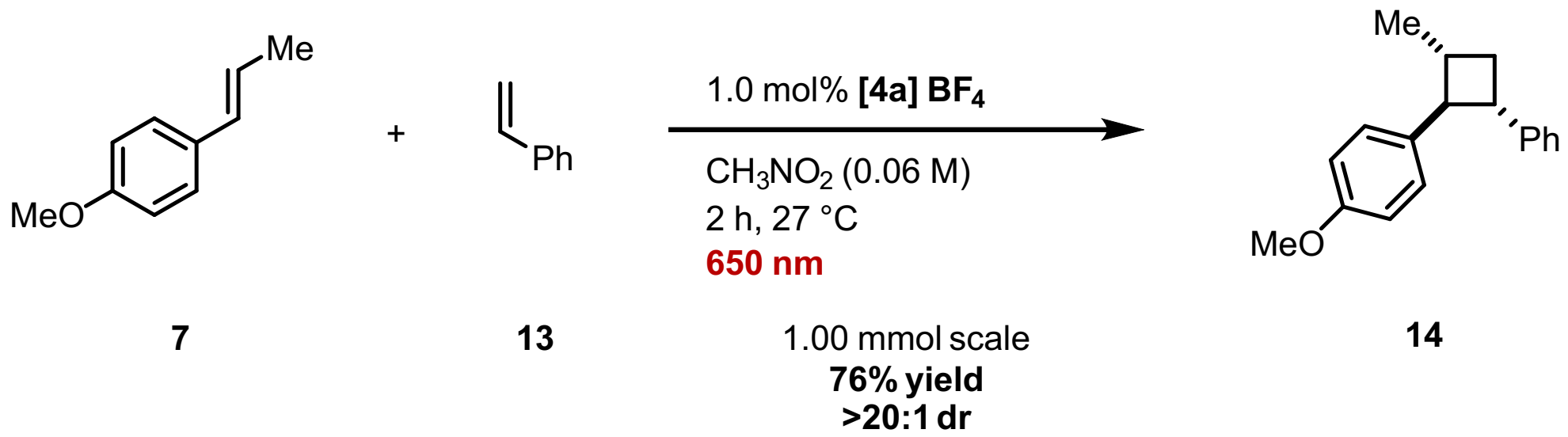

Under an ambient atmosphere, to a 50-mL round-bottom flask equipped with a magnetic stir bar, trans-anethol **7** (148 mg, 150 µL, 1.00 equiv., 1.00 mmol), **[4a] BF₄** (56.1 mg, 1.0 mol%, 0.1 mmol) and CH_3NO_2 (16 mL) were added. Then styrene (**13**) (120 µL, 1.00 equiv., 1.00 mmol) was added to the stirring mixture. The resulting solution was stirred in the photoreactor (red LED irradiation (λ max = 650 nm)) at room temperature under air for 2 h. After stirring the reaction for the given time, the mixture was concentrated by rotary evaporation under reduced pressure. The crude product was purified by flash column chromatography (silica gel, heptane/EtOAc 40:1), affording **14** in 76% yield (192 mg, 0.761 mmol) as a colorless liquid; R_f = 0.38 (heptane/EtOAc 20:1).

NMR Spectroscopy:

^{1}H NMR (400 MHz, $CDCl_3$, 23 °C, δ): 7.32 – 7.23 (m, 2H), 7.22 – 7.12 (m, 5H), 6.90 – 6.80 (m, 2H), 3.80 (s, 3H), 3.40 (td, *J* = 10.0, 7.9 Hz, 1H), 2.95 (t, *J* = 9.5 Hz, 1H), 2.52 (dtd, *J* = 10.2, 7.8, 0.8 Hz, 1H), 2.42 – 2.26 (m, 1H), 1.71 (q, *J* = 10.1 Hz, 1H), 1.19 (d, *J* = 6.5 Hz, 3H).

^{13}C NMR (101 MHz, $CDCl_3$, 23 °C, δ): 158.2, 144.8, 136.0, 128.4, 127.9, 126.8, 126.0, 113.9, 55.7, 55.4, 44.2, 35.6, 34.1, 20.6.

HRMS-ESI(m/z) calc'd for $C_{18}H_{19}O$ $[M–H]^-$, 251.1430; found, 251.1430; deviation: 0.0 ppm.

Boronic acid oxidation reaction catalyzed by xanthylium salts

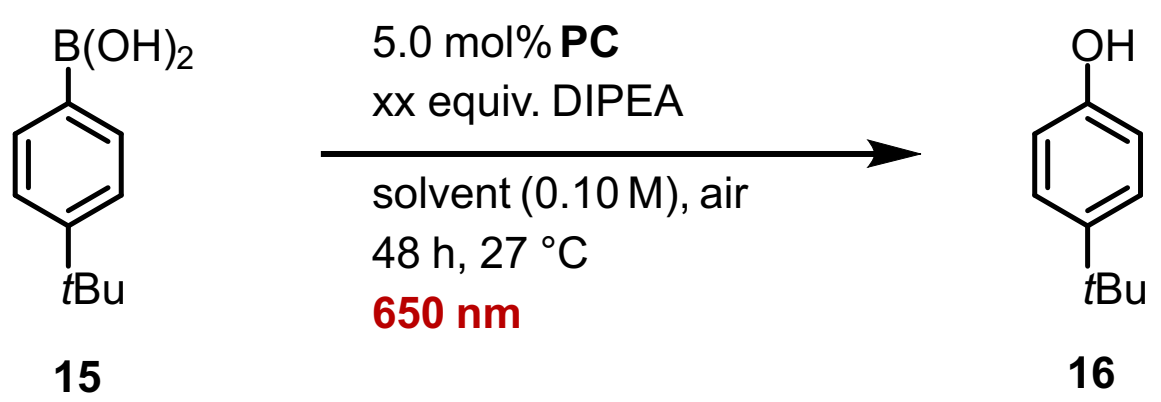

Tested photocatalysts:

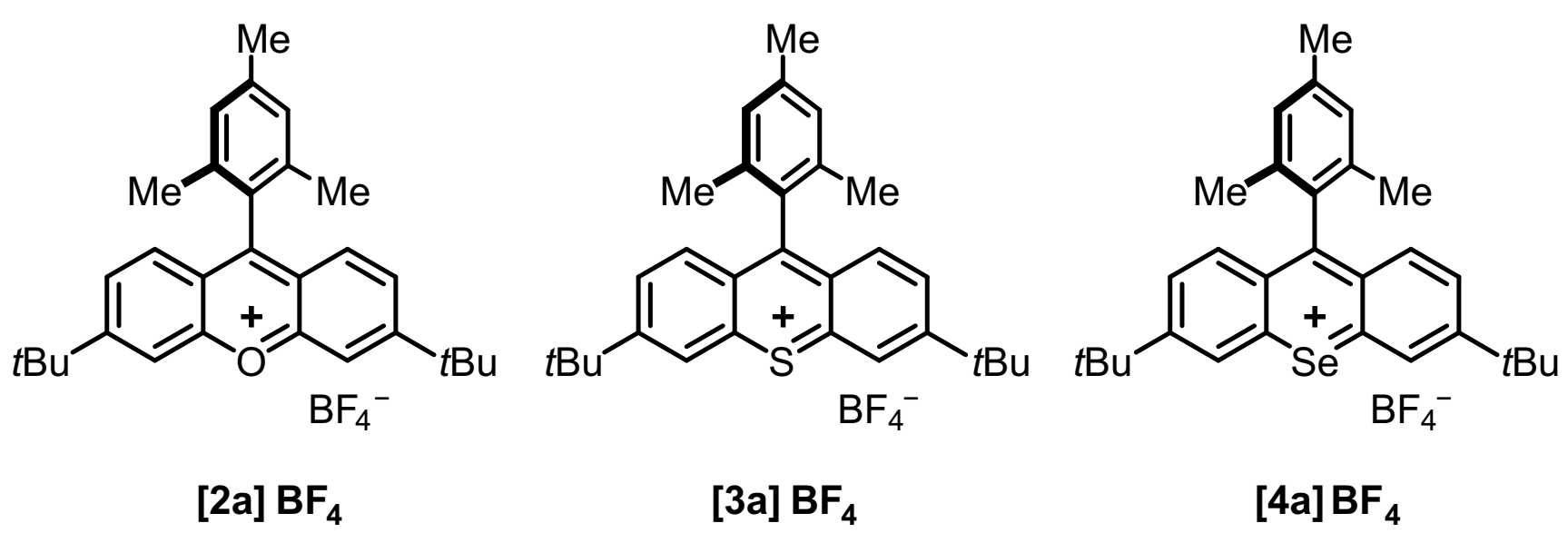

Procedure for the boronic acid oxidation reaction at a 0.10 mmol scale

Under an ambient atmosphere, to a 4-mL borosilicate vial equipped with a magnetic stir bar, (4-(*tert*-butyl)phenyl)boronic acid (**15**) (17.8 mg, 1.00 equiv., 0.10 mmol), photocatalyst (5.00 mol%, 5.00 μmol) and solvent (1.00 mL) were added. Then, DIPEA (1-4 equiv.) was added portionwise to the stirring mixture. The resulting solution was stirred in the photoreactor (red LED irradiation (λ max = 650 nm)) at room temperature under air for 48 h. After stirring the reaction for the given time, the mixture was concentrated by rotary evaporation under reduced pressure. The residue was fully solubilized in $CDCl_3$ (0.50 mL), transferred to the NMR tube, CH_2Br_2 (7.1 μL, 0.10 mmol = theoretical maximum yield) was added as internal standard, and the yield was determined by ^1H NMR spectroscopy using CH_2Br_2 as internal standard by comparing the integration of the CH_2Br_2 signal at 4.93 ppm (s, 2H) to the signal of product **16** at 6.80 ppm (d, 2H).

Process optimization table

Table S7. Boronic acid oxidation reaction optimization.

Entry	PC	DIPEA loading, equiv.	Solvent	Septum-lined cap	Yield **16*** (%)
1	**[4a] BF_4**	1.00	DMF	close	trace
2	**[4a] BF_4**	2.00	DMF	close	5
3	**[4a] BF_4**	4.00	DMF	close	11
4	**[4a] BF_4**	2.00	DMF	close with needle	25
5	**[4a] BF_4**	2.00	DMF	open air	<5
6	**[4a] BF_4**	2.00	DMF/H_2O	open air	<5
7	**[4a] BF_4**	4.00	DMF	close with needle	28
8	**[4a] BF_4**	2.00	CH_3CN	close with needle	38
9	**[4a] BF_4**	2.00	MeOH	close with needle	21
**10**	**[4a] BF_4**	**4.00**	**CH_3CN**	**close with needle**	**73**
11	**[4a] BF_4**	2.00	HFIP	close with needle	9
12	**[2a] BF_4**	4.00	CH_3CN	close with needle	16
13	**[3a] BF_4**	4.00	CH_3CN	close with needle	16
14	**[2a] BF_4**	4.00	CH_3CN	close with needle	<5**
15	**[3a] BF_4**	4.00	CH_3CN	close with needle	<5**
16	**[4a] BF_4**	4.00	CH_3CN	close with needle	<5**
17	**Without PC**	4.00	CH_3CN	close with needle	<5

** The yield was determined by ^1H NMR spectroscopy using CH_2Br_2 as internal standard. ** Reaction in the dark.*

Procedure for the Boronic acid oxidation reaction at a 1.00 mmol scale

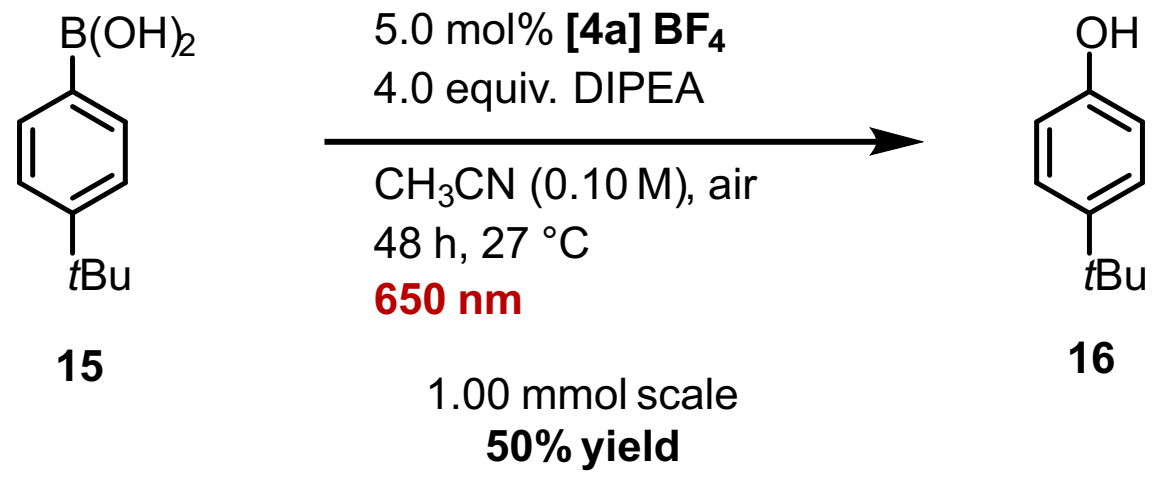

Under an ambient atmosphere, to a 50-mL round-bottom flask equipped with a magnetic stir bar, (4-(*tert*-butyl)phenyl)boronic acid (**15**) (178 mg, 1.00 equiv., 1.00 mmol), photocatalyst **[4a] BF_4** (28.1 mg, 5.0 mol%, 0.05 mmol) and CH_3CN (10.0 mL) were added. Then DIPEA (692 µL, 4.00 equiv., 4.00 mmol) was added portionwise to the stirring mixture. The resulting solution was stirred in the photoreactor (red LED irradiation (λ max = 650 nm)) at room temperature under air for 2 h. After stirring the reaction for the given time, the mixture was concentrated by rotary evaporation under reduced pressure. This mixture was extracted with EtOAc (3×10 mL) and aqueous NH_4Cl solution. The combined organic layer was dried over anhydrous $MgSO_4$ and concentrated by rotary evaporation under reduced pressure. The crude product was purified by flash column chromatography (silica gel, heptane/EtOAc 10:1 to 3:1), affording product **16** in 50% yield (75 mg, 0.50 mmol) as a white solid.

R_f = 0.35 (heptane/EtOAc 2:1).

NMR Spectroscopy:

^{1}H NMR (400 MHz, $CDCl_3$, 23 °C, δ): 7.26 (d, *J* = 8.8 Hz, 2H), 6.77 (d, *J* = 8.7 Hz, 1H), 4.57 (s, 1H), 1.30 (s, 9H).

^{13}C NMR (101 MHz, $CDCl_3$, 23 °C, δ): 153.2, 143.7, 126.6, 114.9, 34.2, 31.7.

HRMS-ESI(m/z) calc'd for $C_{10}H_{14}O$ $[M]^+$, 150.1006; found, 150.1006; deviation: 0.0 ppm.

Aza-Henry reaction catalyzed by xanthylium salts

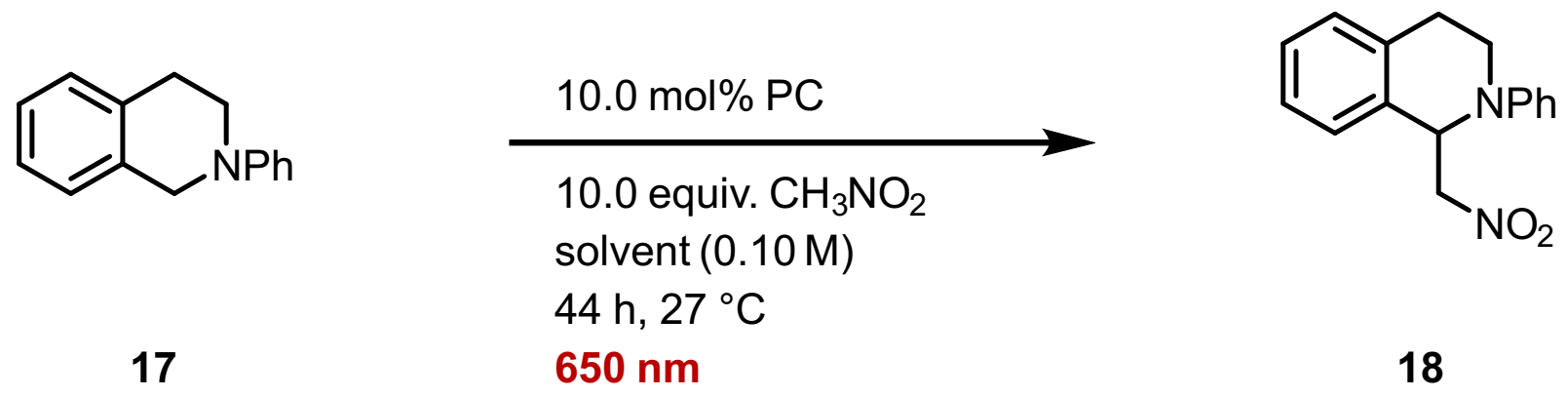

Tested photocatalysts:

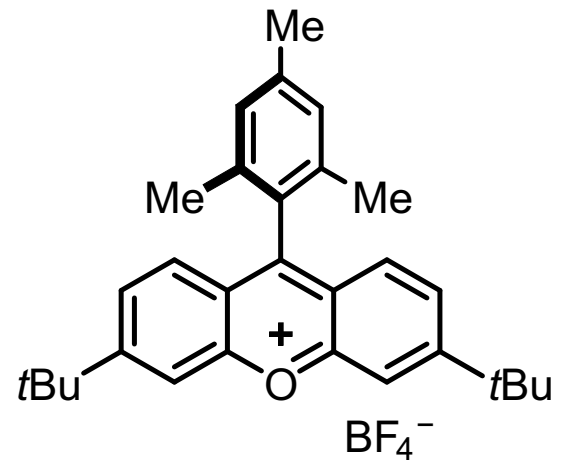

[2a] BF$_4$

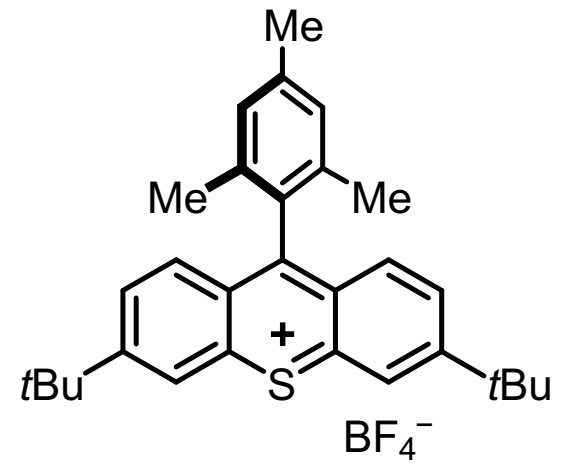

[3a] BF$_4$

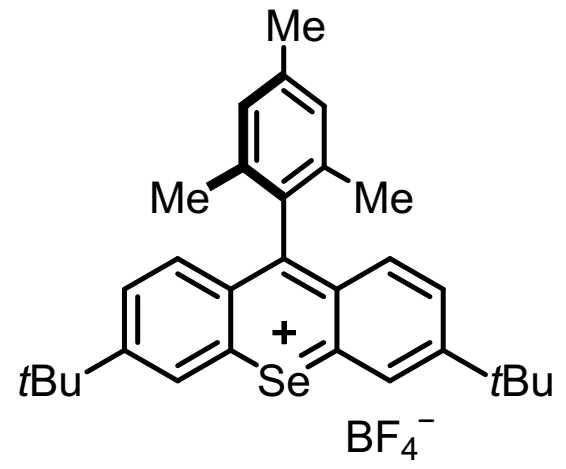

[4a] BF$_4$

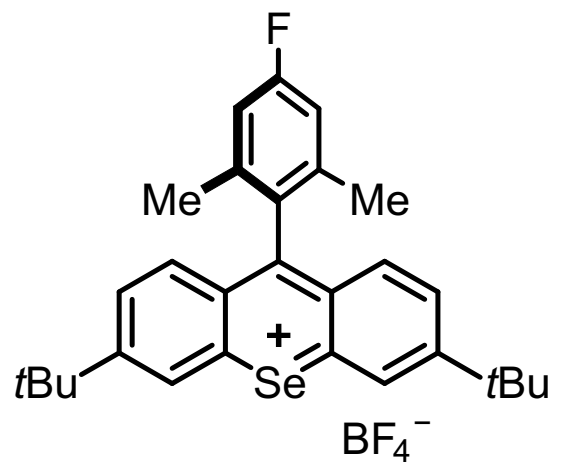

[4b] BF$_4$

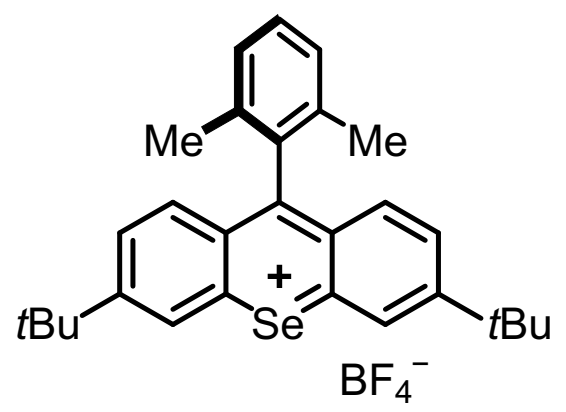

[4c] BF$_4$

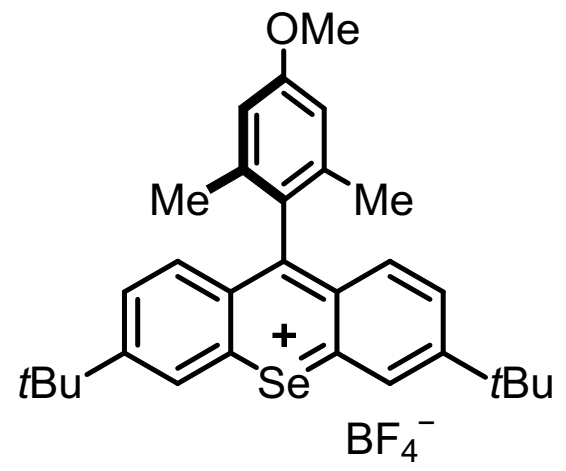

[4d] BF$_4$

Oxidation potential of **7**: E_{ox} = 0.88V vs SCE[16]

General procedure for the aza-Henry reaction at 0.12 mmol scale

Under an ambient atmosphere, to a 4-mL borosilicate scintillation vial equipped with a magnetic stir bar, 2-phenyl-1,2,3,4-tetrahydroisoquinoline (**17**) (25.1 mg, 1.00 equiv. 0.12 mmol), the photocatalyst (1.0 mol%), CH_3NO_2 (67.4 µL, 10.0 equiv., 1.20 mmol) and the solvent (1.2 mL) were added to the stirring mixture. The resulting solution was stirred in the photoreactor (red LED irradiation (λ max = 650 nm)) at room temperature under air for 44 h. After stirring the reaction for the given time, the reaction mixture was concentrated by rotary evaporation under reduced pressure. The residue was fully solubilized in $CDCl_3$ (0.50 mL) and transferred to a NMR tube. CH_2Br_2 (7.1 µL, 0.10 mmol = theoretical maximum yield) was added as internal standard. The yield was determined by ^{1}H NMR spectroscopy using CH_2Br_2 as internal standard by comparing the integration of the CH_2Br_2 signal at 4.94 ppm (s, 2H) to the signal of product **18** at 5.57 ppm (t, 1H).

Process optimization table

Table S8. Alkene trifluoromethylation reaction optimization.

Entry	PC	Solvent	Time (h)	Septum-lined cap	Yield **18*** (%)
1	**[4a] BF_4**	DMSO-*d6*	44	Close	20
2	**[4a] BF_4**	CH_3CN	65	Close	22
3	**[4a] BF_4**	CHCl3	65	Close	23
4	**[4a] BF_4**	CH_3NO_2	44	Close	43
5	**[4a] BF_4**	CH_3NO_2	44	Open air	27
6	**[4a] BF_4**	CH_3NO_2	65	Close	48
7	**[4a] BF_4**	CH_3NO_2	44	Close with needle	56
8	**[4b] BF_4**	CH_3NO_2	44	Close with needle	52
**9**	**[4c] BF_4**	**CH_3NO_2**	**44**	**Close with needle**	**65**
10	**[4d] BF_4**	CH_3NO_2	44	Close with needle	35
11	**[2a] BF_4**	CH_3NO_2	44	Close with needle	6
12	**[3a] BF_4**	CH_3NO_2	44	Close with needle	12
13	**[2a] BF_4**	CH_3NO_2	44	Close with needle	<5**
14	**[3a] BF_4**	CH_3NO_2	44	Close with needle	<5**
15	**[4a] BF_4**	CH_3NO_2	44	Close with needle	<5**
16	**Without PC**	CH_3NO_2	44	Close with needle	<5

** The yield was determined by ^{1}H NMR spectroscopy using CH_2Br_2 as internal standard.*

Procedure for the aza-Henry reaction at 0.50 mmol scale

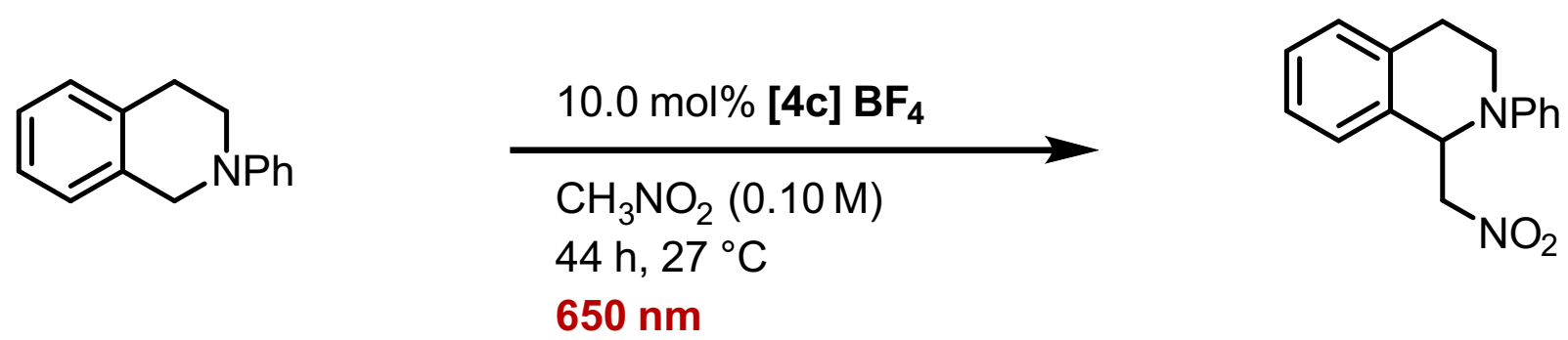

17 0.50 mmol scale **43% yield** **18**

Under an ambient atmosphere, to a 20-mL borosilicate vial equipped with a magnetic stir bar2-phenyl-1,2,3,4-tetrahydroisoquinoline (**17**) (25.1 mg, 1.00 equiv. 0.120 mmol), **[4c] BF_4** (27.4 mg, 1.0 mol%, 0.05 mmol) and CH_3NO_2 (4.2 mL) were added to the stirring mixture. The resulting solution was stirred in the photoreactor (red LED irradiation (λ max = 650 nm)) at room temperature under air for 44 h. After stirring the reaction for the given time, the reaction mixture was concentrated by rotary evaporation under reduced pressure. The crude product was purified by flash column chromatography (silica gel, heptane/EtOAc 20:1), affording product **9** in 43% yield (57.1 mg, 0.213 mmol) as a yellow solid.

R_f = 0.20 (heptane/EtOAc 10:1).

NMR Spectroscopy:

^{1}H NMR (400 MHz, $CDCl_3$, 23 °C, δ): 7.34 – 7.11 (m, 6H), 7.00 (dd, *J* = 8.9, 1.1 Hz, 2H), 6.87 (t, *J* = 7.3 Hz, 1H), 5.57 (t, *J* = 7.2 Hz, 1H), 4.88 (dd, *J* = 11.8, 7.8 Hz, 1H), 4.57 (dd, *J* = 11.8, 6.6 Hz, 1H), 3.74 – 3.55 (m, 2H), 3.10 (ddd, *J* = 16.3, 8.7, 5.7 Hz, 1H), 2.81 (dt, *J* = 16.3, 4.9 Hz, 1H).

^{13}C NMR (101 MHz, $CDCl_3$, 23 °C, δ): 148.6, 135.4, 133.0, 129.6, 129.3, 128.2, 127.1, 126.8, 119.6, 115.2, 78.9, 58.3, 42.2, 26.6.

HRMS-ESI(m/z) calc'd for $C_{16}H_{16}N_2O_2$ $[M+H]^+$, 269.1285; found, 269.1281; deviation: –1.5 ppm.

Desulfinylative trifluoromethylation reaction catalyzed by xanthylium salts

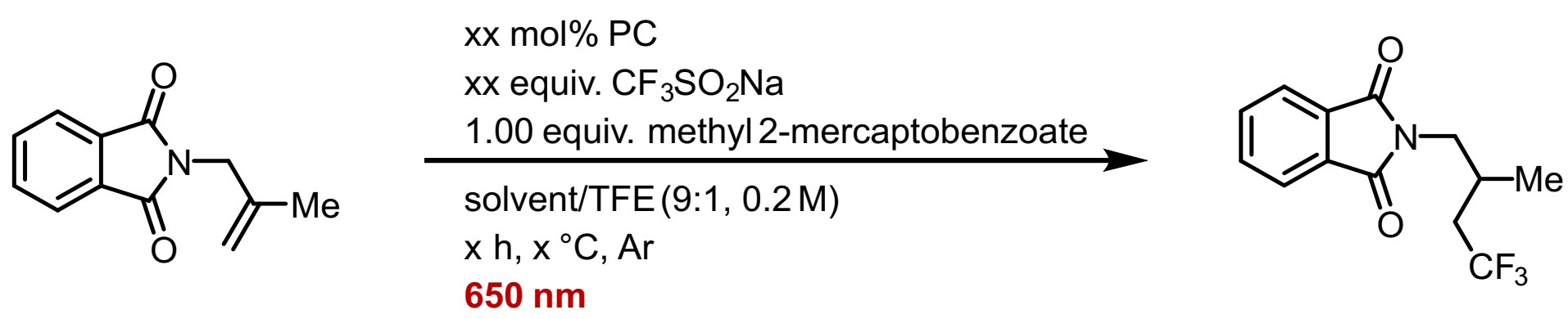

19 **20**

Procedure for the alkene trifluoromethylation reaction at a 0.10 mmol scale

To a 4-mL borosilicate vial equipped with a magnetic stir bar, 2-(2-methylallyl)isoindoline-1,3-dione (**19**) (20.5 mg, 1.00 equiv., 0.10 mmol), sodium trifluoromethanesulfinate (1.5-2.5 equiv.), and photocatalyst were added. The vial was sealed with a membrane cap, connected via a needle and a Tygon® tubing to a Schlenk line and evacuated and purged with argon three times. Solvent (0.45 mL), TFE (0.05 mL) were added via 1-mL syringes, and methyl 2-mercaptobenzoate (13.8 µL, 1.00 equiv., 0.10 mmol) was added via a microsyringe. The resulting solution was stirred in the photoreactor (red LED irradiation (λ max = 650 nm)) with or without cooling for the reaction time. After stirring, the mixture was concentrated by rotary evaporation under reduced pressure. The residue was fully solubilized in $CDCl_3$ (0.50 mL), transferred to the NMR tube, CH_2Br_2 (7.1 µL, 0.10 mmol = theoretical maximum yield) was added as internal standard, and the yield was determined by ^{1}H NMR spectroscopy using CH_2Br_2 as internal standard by comparing the integration of the CH_2Br_2 signal at 4.93 ppm (s, 2H) to the signal of product **20** at 1.07 ppm (d, *J* = 6.8 Hz, 3H).

Process optimization table

Table S9. Desulfinylative trifluoromethylation reaction optimization.

Entry	PC	Solvent	Temperature, °C	PC loading, mol%	Reaction time, h	CF_3SO_2Na equiv.	Yield **20*** (%)
1	**[4a] BF$_4$**	$CHCl_3$	27	5.0	65	1.5	11
2	**[4a] BF$_4$**	CH_3CN	27	5.0	65	1.5	12
3	**[4a] BF$_4$**	CH_2Cl_2	27	5.0	65	1.5	18

4	**[4a] BF$_4$**	CH_2Cl_2	39	5.0	24	1.5	28
5	**[4a] BF$_4$**	1,2-DCE	39	5.0	24	1.5	57
6	**[4a] BF$_4$**	1,2-DCE	39	5.0	24	2.5	40
7	**[4a] BF$_4$**	1,2-DCE	39	10	24	1.5	52
**8**	**[4a] BF$_4$**	**1,2-DCE**	**39**	**5.0**	**48**	**1.5**	**63**
9	**[4a] BF$_4$**	1,2-DCE	27	5.0	48	1.5	31
10	**[4a] BF$_4$**	1,2-DCE	27	5.0	24	1.5	<5**
11	**[4a] BF$_4$**	1,2-DCE	27	-	24	1.5	-
12	**[2a] BF$_4$**	1,2-DCE	39	5.0	48	1.5	0
13	**[3a] BF$_4$**	1,2-DCE	39	5.0	48	1.5	14

** The yield was determined by ^{1}H NMR spectroscopy using CH_2Br_2 as internal standard. ** Reaction in the dark.*

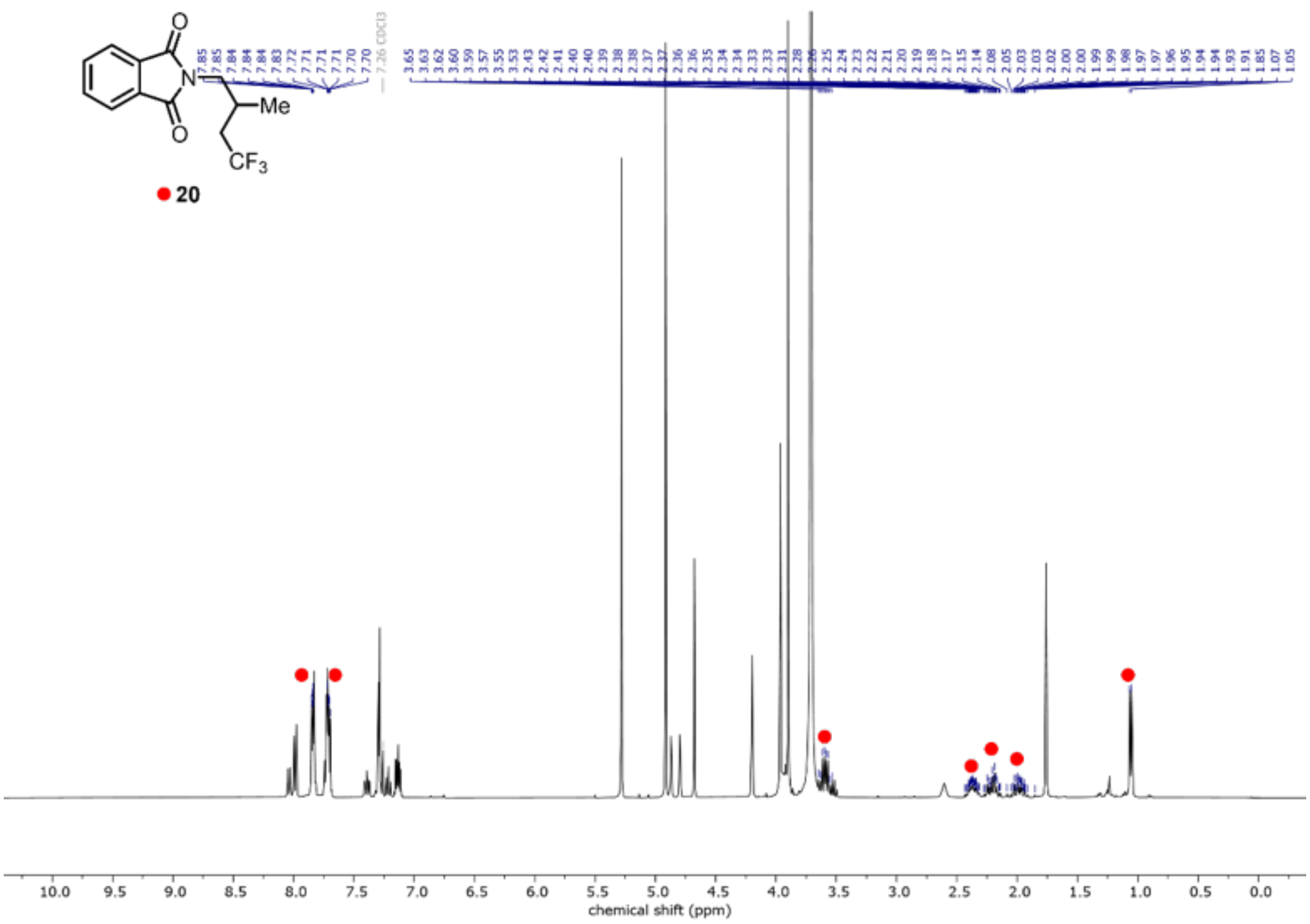

Figure S10. ^{1}H NMR (400 MHz, $CDCl_3$, 23 °C, δ) spectrum of desulfinylative trifluoromethylation crude after the reaction.

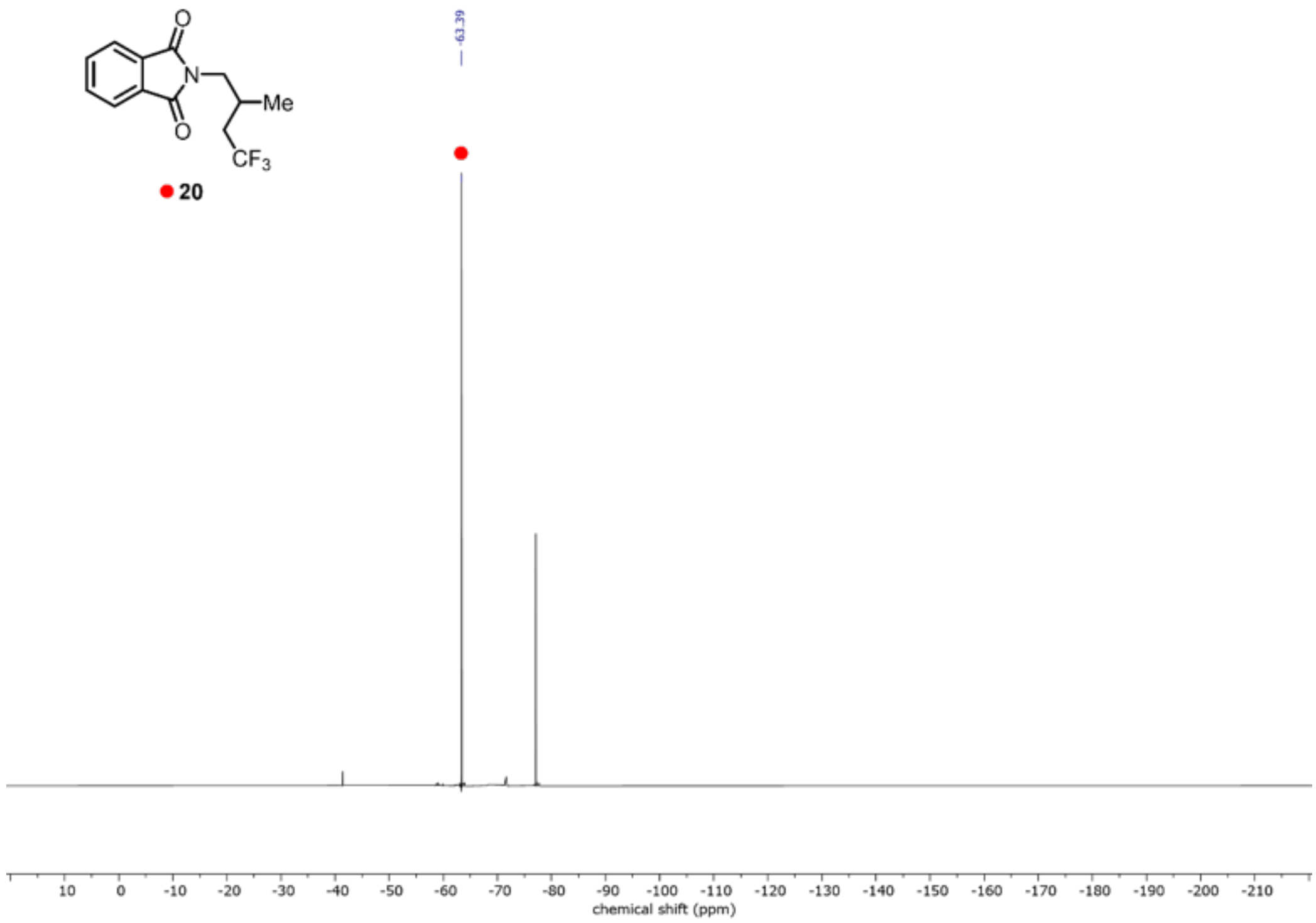

Figure S11. ^{19}F NMR (376 MHz, $CDCl_3$, 23 °C, δ) spectrum of desulfinylative trifluoromethylation crude after the reaction.

Procedure for the desulfinylative trifluoromethylation reaction at a 0.25 mmol scale

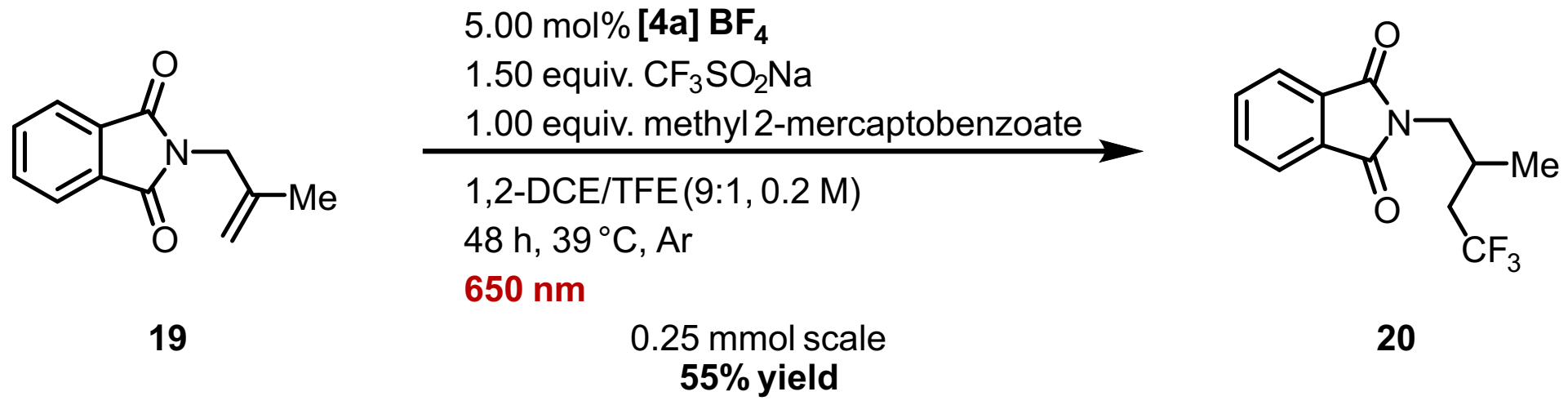

To a 4-mL borosilicate vial equipped with a magnetic stir bar, 2-(2-methylallyl)isoindoline-1,3-dione (**19**) (20.5 mg, 1.00 equiv., 0.10 mmol), sodium trifluoromethanesulfinate (1.5-2.5 equiv.), and photocatalyst **[4a] BF_4** were added. The vial was sealed with a membrane cap, connected via a needle and a Tygon® tubing to a Schlenk line and evacuated and purged with argon three times. Solvent (0.45 mL), TFE (0.05 mL) were added via 1-mL syringes, and methyl 2-mercaptobenzoate (13.8 µL, 1.00 equiv., 0.10 mmol) was added via a microsyringe. The resulting solution was stirred in the photoreactor (red LED irradiation (λ max = 650 nm)) with or without cooling for the reaction time. After stirring, the mixture was concentrated by rotary evaporation under reduced pressure. The crude product was purified by flash column chromatography (silica gel, heptane/EtOAc 10:1), affording product **20** in 55% yield (37.0 mg, 136 µmol) as a yellow solid.

R_f = 0.18 (heptane/EtOAc 10:1).

NMR Spectroscopy:

^{1}H NMR (400 MHz, $CDCl_3$, 23 °C, δ): 7.86 – 7.84 (m, 2H), 7.74 – 7.70 (m, 2H), 3.65 (dd, *J* = 13.8

Hz, *J* = 7.2 Hz, 1H), 3.59 (dd, *J* = 13.8 Hz, *J* = 6.6 Hz, 1H), 2.43 – 2.31 (m, 1H), 2.28 – 2.14 (m, 1H), 2.08 – 1.91 (m, 1H) 1.06 (d, *J* = 6.9 Hz, 3H).

^{13}C NMR (101 MHz, $CDCl_3$, 23 °C, δ): 168.5, 134.2, 131.8, 123.4, 126.7 (q, J = 275.8 Hz), 43.4, 38.1 (q, *J* = 27.9 Hz), 27.9 (q, *J* = 2.4 Hz), 17.7.

^{19}F NMR (377 MHz, $CDCl_3$, 23 °C, δ): –63.38.

Oxidation of anthracene catalyzed by selenoxanthylium salts at 0.10 mmol scale.

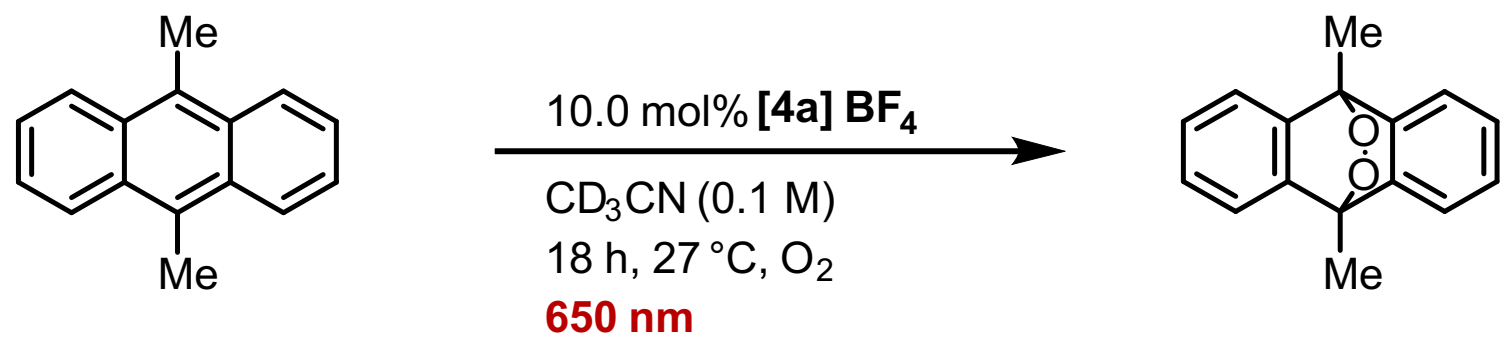

20 21

Procedure for the oxidation of anthracene reaction at a 0.10 mmol scale

To a J-Young NMR tube, anthracene (**20**) (18.4 mg, 1.00 equiv., 0.10 mmol), photocatalyst **[4a] BF₄** (5.61 mg, 10.0 mol%, 0.01 mmol) were added. The headspace of the tube was flushed with an O_2 balloon for 5 min. Separately, CD_3CN was sparged with an O_2 balloon for 5 min. Then, oxygenated CD_3CN (0.60 mL) was added to the NMR tube using a 1.0-mL syringe under oxygen shower. The tube was capped, and the mixture was analyzed by ^1H NMR spectroscopy (see **Figure S12**). The NMR tube was irradiated with red-light (red-light irradiation, λ_{max} = 650 nm) for 18h. Irradiation was stopped and CH_2Br_2 (7.1 µL, 0.10 mmol = theoretical maximum yield) was added as internal standard. The yield was determined by ^1H NMR spectroscopy using CH_2Br_2 as internal standard by comparing the integration of the CH_2Br_2 signal at 5.09 ppm (s, 2H) to the signal of product **21** at 7.31–7.33 ppm (m, 4H) (see **Figure S13**).

Process optimization table

Table S10. Oxidation of anthracene reaction using selenoxanthylium photocatalysts.

Solvent	CH_3CN
yield* (%)	72

** The yield was determined by ^1H NMR spectroscopy using CH_2Br_2 as internal standard.*

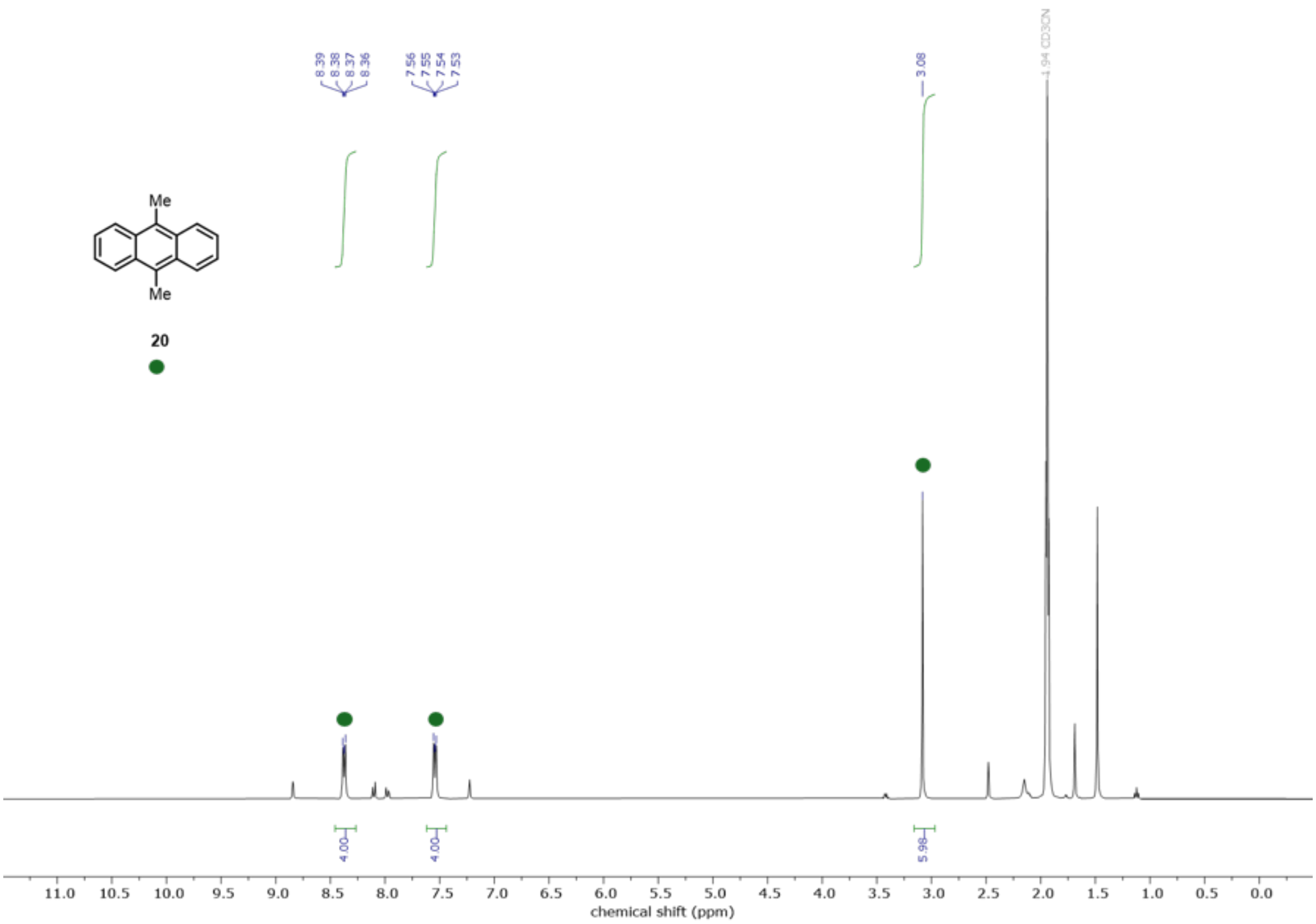

Figure S12. ^{1}H NMR (400 MHz, $CDCl_3$, 23 °C, δ) spectrum of crude before the reaction.

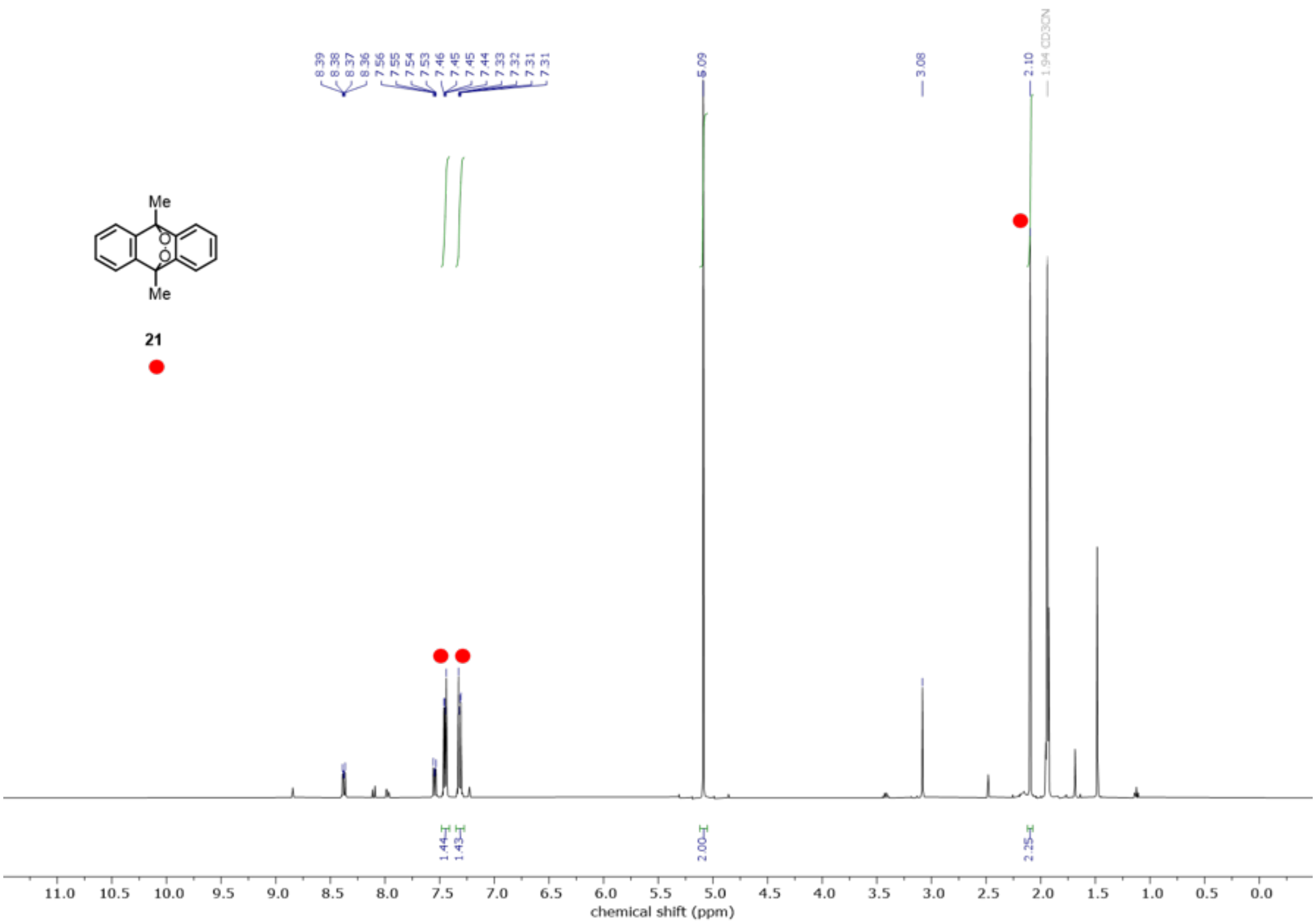

Figure S13. 1H NMR (400 MHz, $CDCl_3$, 23 °C, δ) spectrum of crude after the reaction.

Photocatalyst characterization

Electrochemical data

3,6-Di-tert-butyl-9-mesitylacridinium tetrafluoroborate ([1a] BF4)

Reduction – Redox couple ([1a]$^+$/[1a]$^{\cdot}$): $E_{p/2}$ = −1.01 V vs Fc/Fc$^+$ (= −0.63 V vs. SCE).

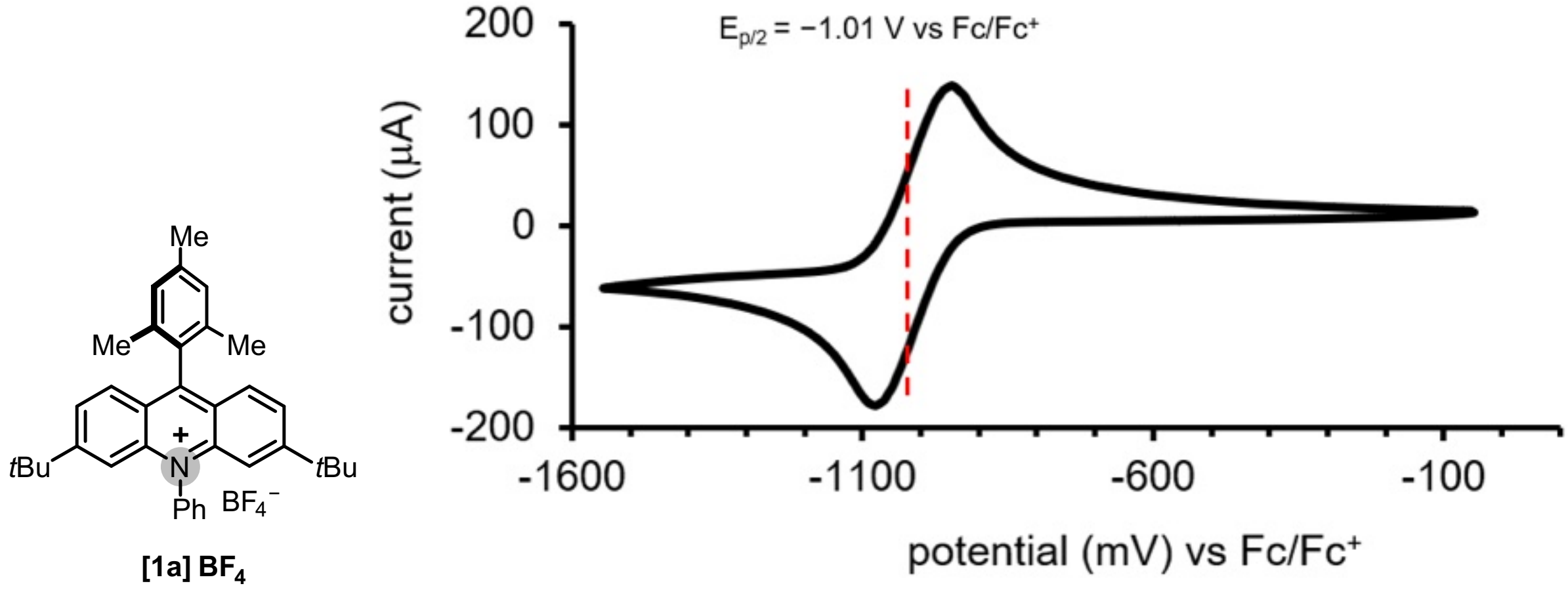

Figure S15. Cathodic CV of **[1a] BF$_4$** in a 0.10 M NBu_4PF_6 CH_3CN solution. GC electrode. Scan rate: 100 mV/s, potential referred to Ag/$AgNO_3$ (0.10 M in CH_3CN) using Fc/Fc$^+$ as internal reference at 298 K with a platinum disk as counter electrode.

3,6-Di-*tert*-butyl-9-mesitylxanthylium tetrafluoroborate ([2a] BF$_4$)

Reduction – Redox couple ([2a]$^+$/[2a]$^{\cdot}$): $E_{p/2}$ = −0.54 V vs Fc/Fc$^+$ (= −0.16 V vs. SCE).

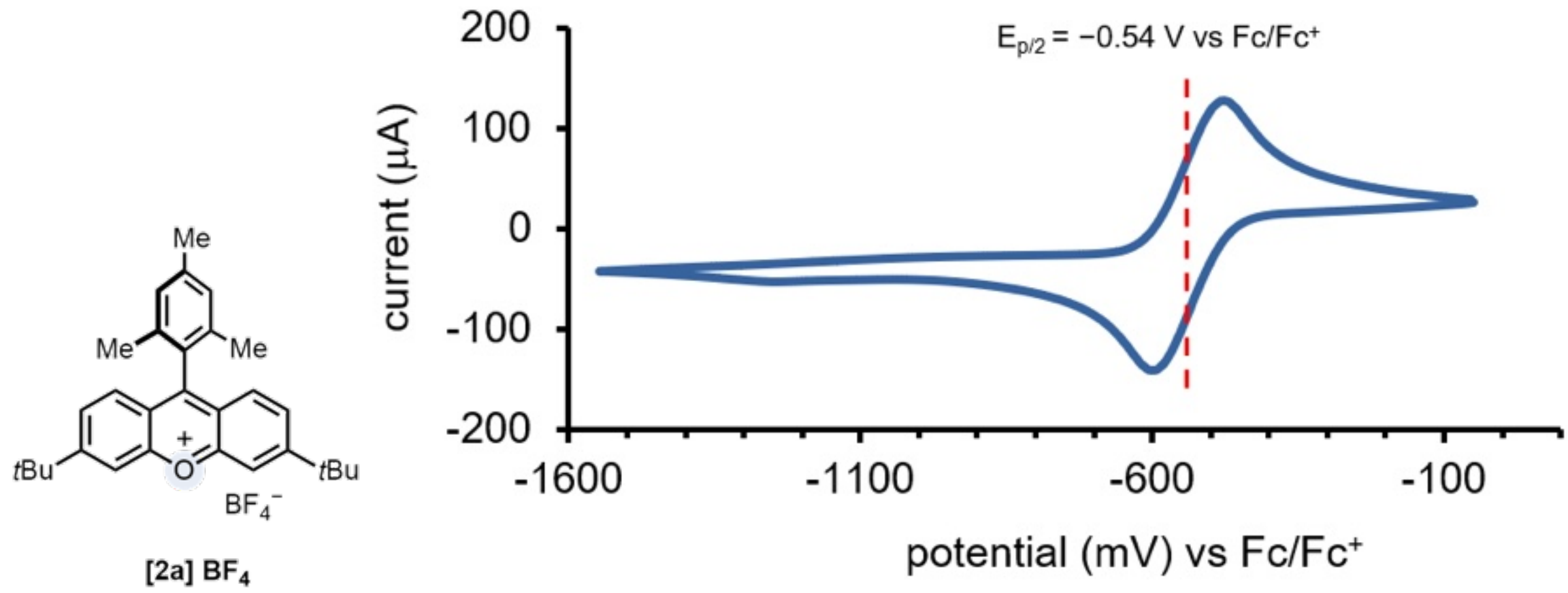

Figure S16. Cathodic CV of **[2a] BF$_4$** in a 0.10 M NBu_4PF_6 CH_3CN solution. GC electrode. Scan rate: 100 mV/s, potential referred to Ag/$AgNO_3$ (0.10 M in CH_3CN) using Fc/Fc$^+$ as internal reference at 298 K with a platinum disk as counter electrode.

3,6-Di-*tert*-butyl-9-mesitylthioxanthylium tetrafluoroborate ([3a] BF_4)

Reduction – Redox couple ([3a]$^+$/[3a]$^{\cdot}$): $E_{p/2}$ = −0.52 V vs Fc/Fc$^+$ (= −0.14 V vs. SCE).

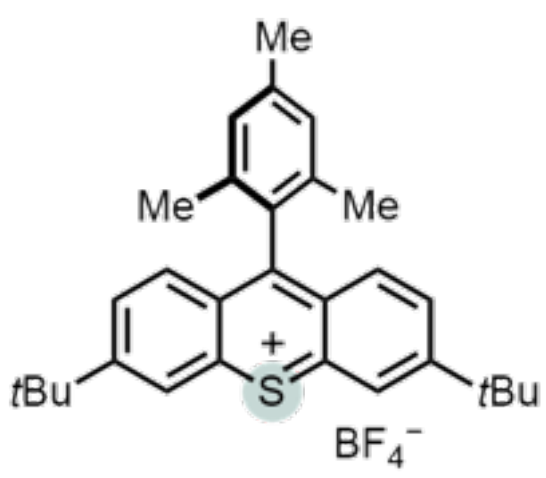

[3a] BF_4

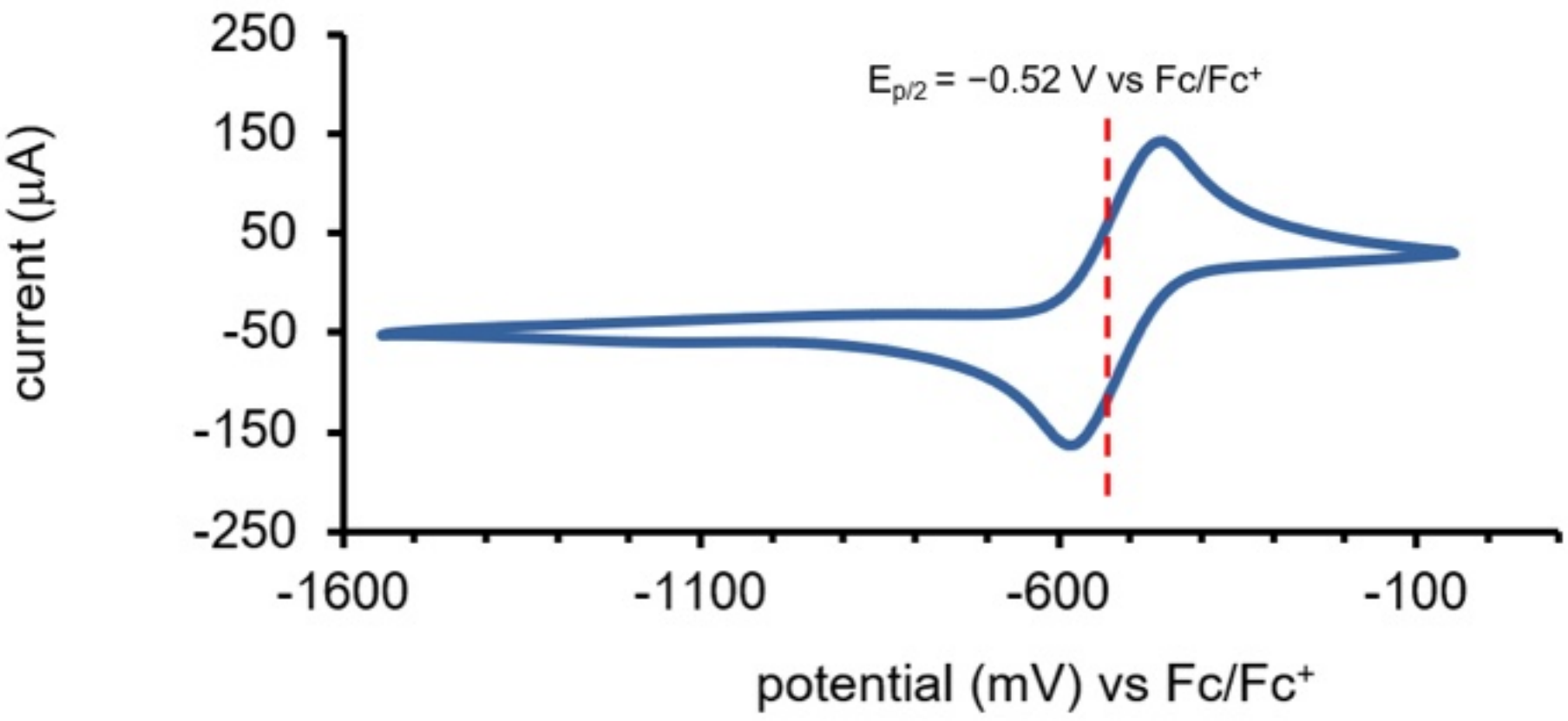

Figure S17. Cathodic CV of **[3a] BF_4** in a 0.10 M NBu_4PF_6 CH_3CN solution. GC electrode. Scan rate: 100 mV/s, potential referred to Ag/$AgNO_3$ (0.10 M in CH_3CN) using Fc/Fc$^+$ as internal reference at 298 K with a platinum disk as counter electrode.

3,6-Di-*tert*-butyl-9-mesitylselenoxanthylium tetrafluoroborate ([4a] BF_4)

Reduction – Redox couple ($[4a]^+/[4a]^{\cdot}$): $E_{p/2}$ = −0.46 V vs Fc/Fc^+ (= −0.08 V vs. SCE).

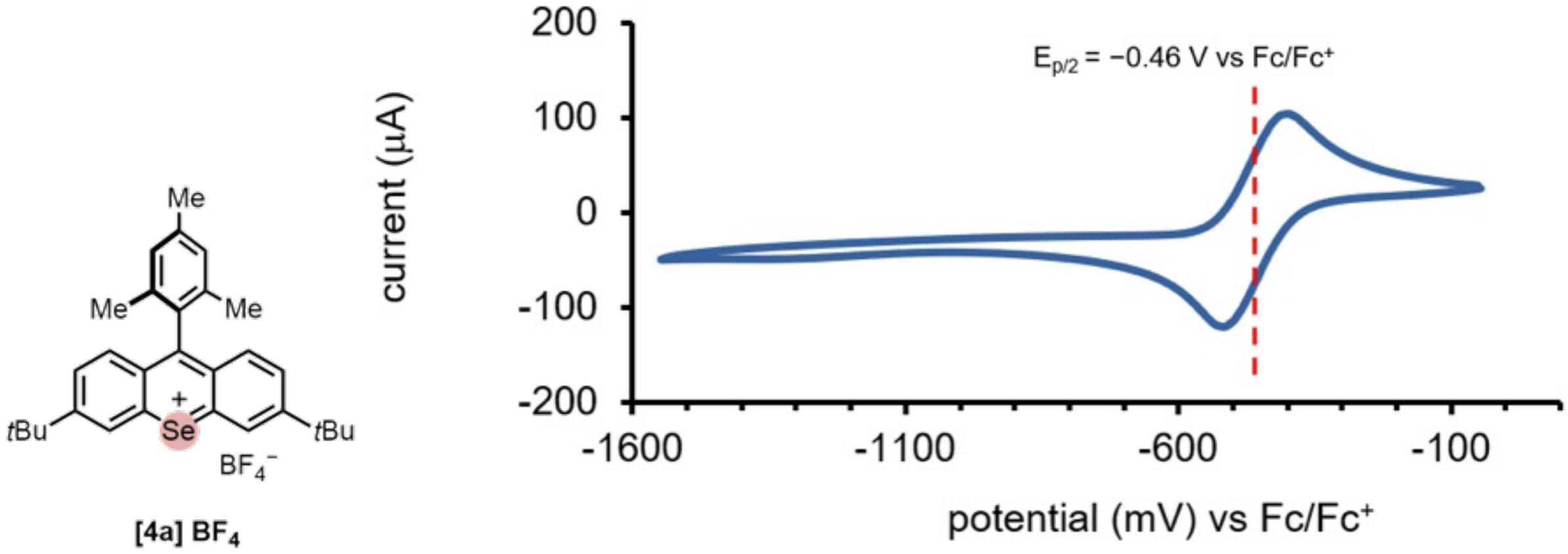

Figure S18. Cathodic CV of **[4a] BF_4** in a 0.10 M NBu_4PF_6 CH_3CN solution. GC electrode. Scan rate: 100 mV/s, potential referred to $Ag/AgNO_3$ (0.10 M in CH_3CN) using Fc/Fc^+ as internal reference at 298 K with a platinum disk as counter electrode.

Oxidation – ($[4a]^+$): $E_{p/2}$ = +0.90 V vs Fc/Fc^+ (= +1.28 V vs. SCE).

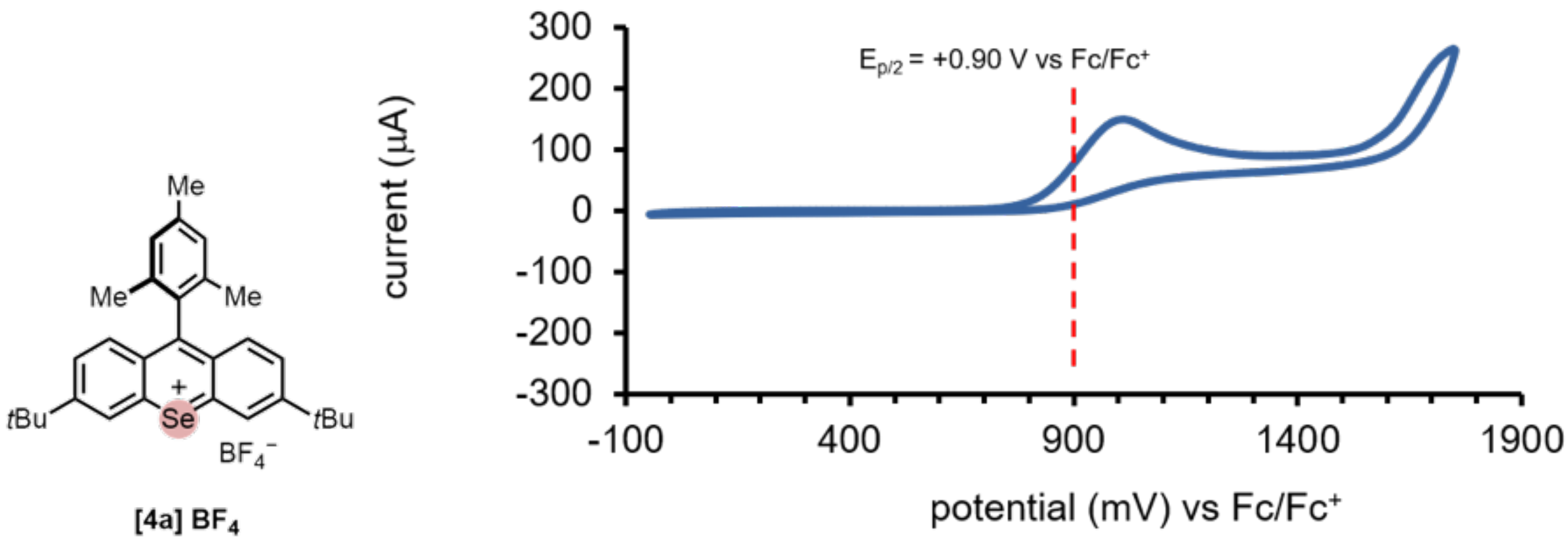

Figure S19. Anodic CV of **[4a] BF_4** in a 0.10 M NBu_4PF_6 CH_3CN solution. GC electrode. Scan rate: 100 mV/s, potential referred to $Ag/AgNO_3$ (0.10 M in CH_3CN) using Fc/Fc^+ as internal reference at 298 K with a platinum disk as counter electrode.

3,6-Di-*tert*-butyl-9-(4-fluoro-2,6-dimethylphenyl)selenoxanthylium tetrafluoroborate ([4b] BF_4)

Reduction – Redox couple ($[4b]^{+}/[4b]^{\cdot}$): $E_{p/2}$ = −0.44 V vs Fc/Fc^+ (= −0.06 V vs. SCE).

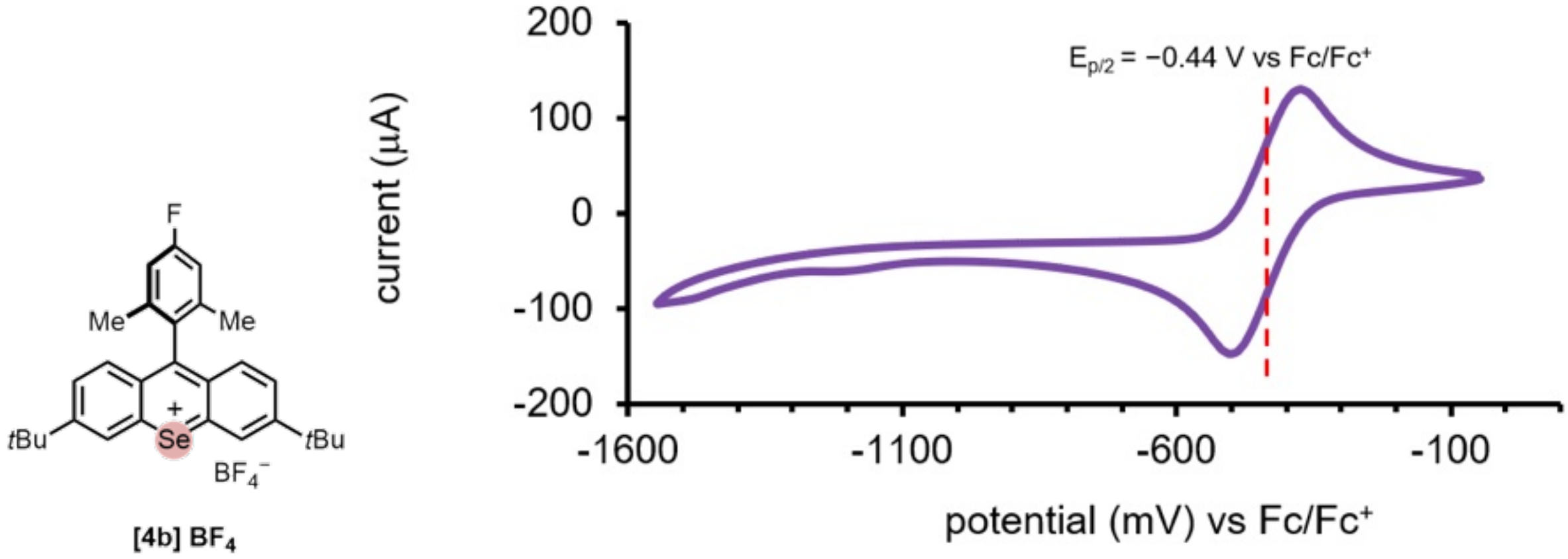

Figure S20. Cathodic CV of **[4b] BF_4** in a 0.10 M NBu_4PF_6 CH_3CN solution. GC electrode. Scan rate: 100 mV/s, potential referred to $Ag/AgNO_3$ (0.10 M in CH_3CN) using Fc/Fc^+ as internal reference at 298 K with a platinum disk as counter electrode.

Oxidation – ($[4b]^{+}$): $E_{p/2}$ = +1.28 V vs Fc/Fc^+ (= +1.66 V vs. SCE).

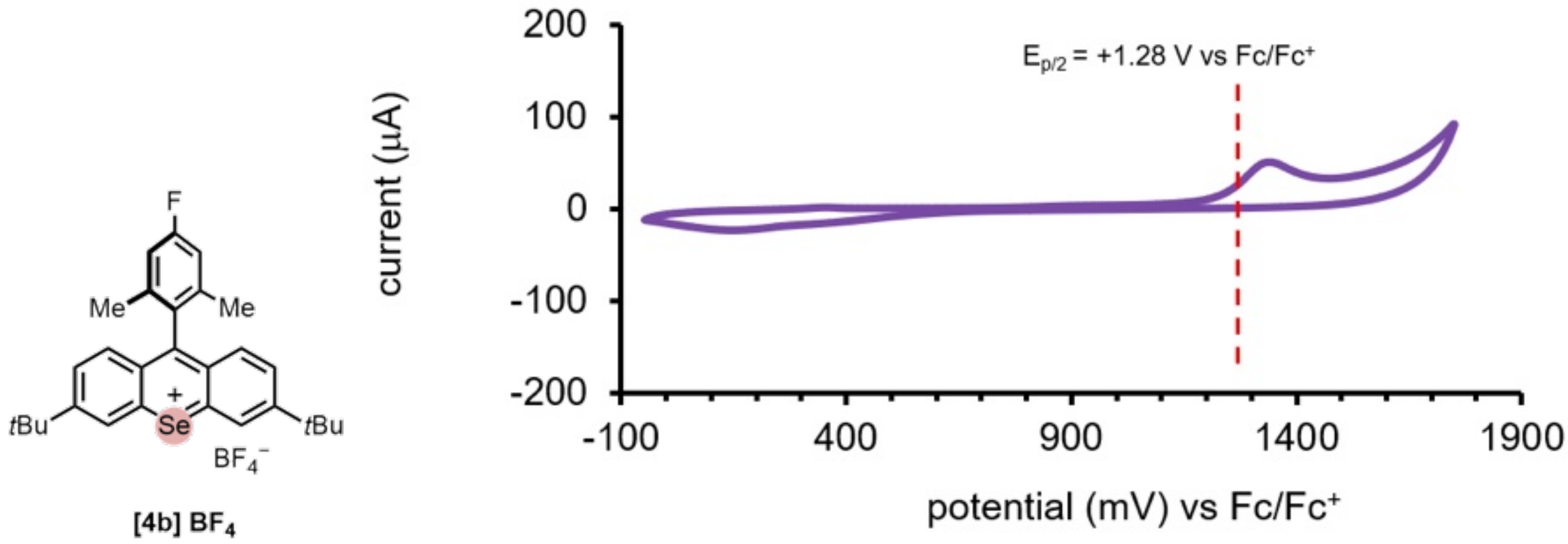

Figure S21. Anodic CV of **[4b] BF_4** in a 0.10 M NBu_4PF_6 CH_3CN solution. GC electrode. Scan rate: 100 mV/s, potential referred to $Ag/AgNO_3$ (0.10 M in CH_3CN) using Fc/Fc^+ as internal reference at 298 K with a platinum disk as counter electrode.

3,6-Di-*tert*-butyl-9-(2,6-dimethylphenyl)selenoxanthylium tetrafluoroborate ([4c] BF_4)

Reduction – Redox couple ($[4c]^+/[4c]^\cdot$): $E_{p/2}$ = −0.46 V vs Fc/Fc^+ (= −0.08 V vs. SCE).

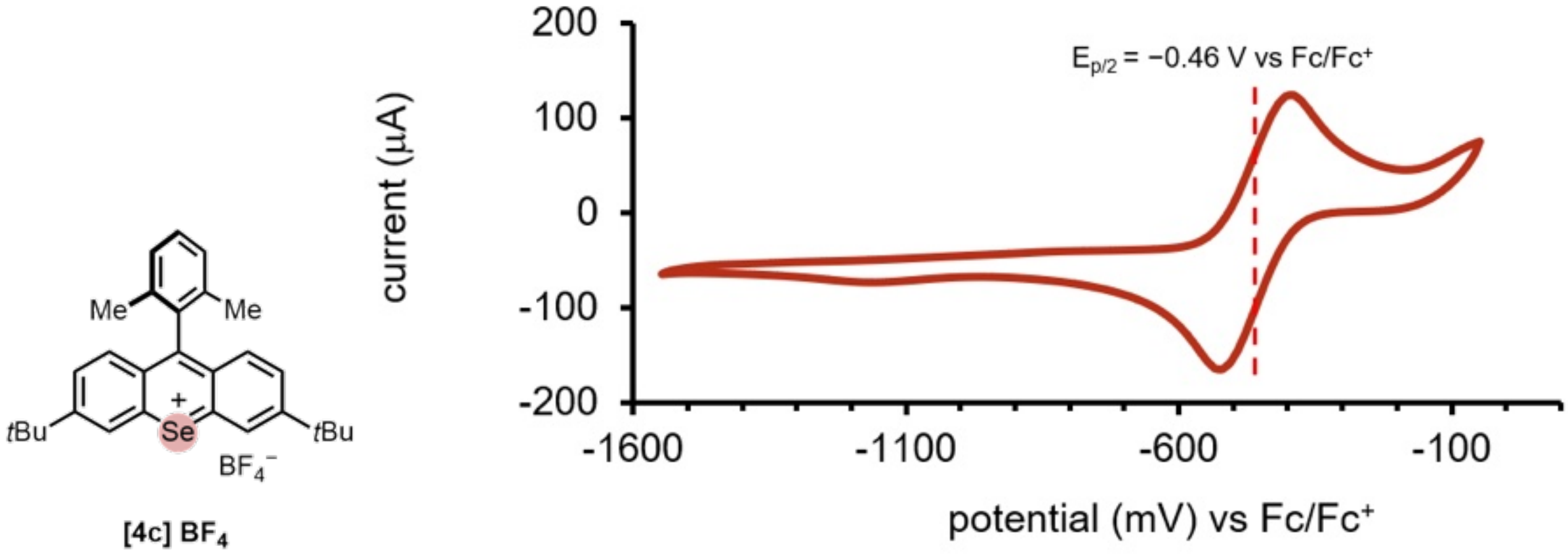

Figure S22. Cathodic CV of **[4c] BF_4** in a 0.10 M NBu_4PF_6 CH_3CN solution. GC electrode. Scan rate: 100 mV/s, potential referred to $Ag/AgNO_3$ (0.10 M in CH_3CN) using Fc/Fc^+ as internal reference at 298 K with a platinum disk as counter electrode.

Oxidation – ($[4c]^+$): $E_{p/2}$ = +1.61 V vs Fc/Fc^+ (= +1.99 V vs. SCE).

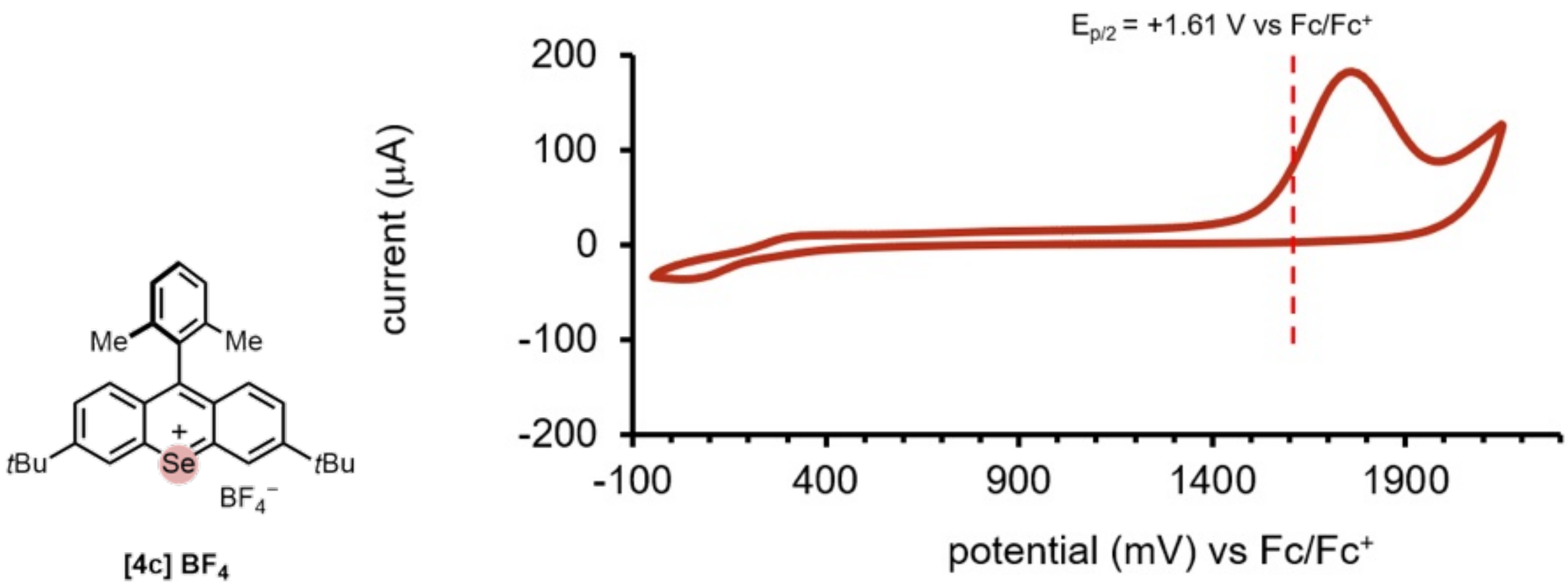

Figure S23. Anodic CV of **[4c] BF_4** in a 0.10 M NBu_4PF_6 CH_3CN solution. GC electrode. Scan rate: 100 mV/s, potential referred to $Ag/AgNO_3$ (0.10 M in CH_3CN) using Fc/Fc^+ as internal reference at 298 K with a platinum disk as counter electrode.

3,6-Di-*tert*-butyl-9-(4-methoxy-2,6-dimethylphenyl)selenoxanthylium tetrafluoroborate ([4d] BF_4)

Reduction – Redox couple ($[4d]^+/[4d]^•$): $E_{p/2}$ = −0.45 V vs Fc/Fc^+ (= −0.07 V vs. SCE).

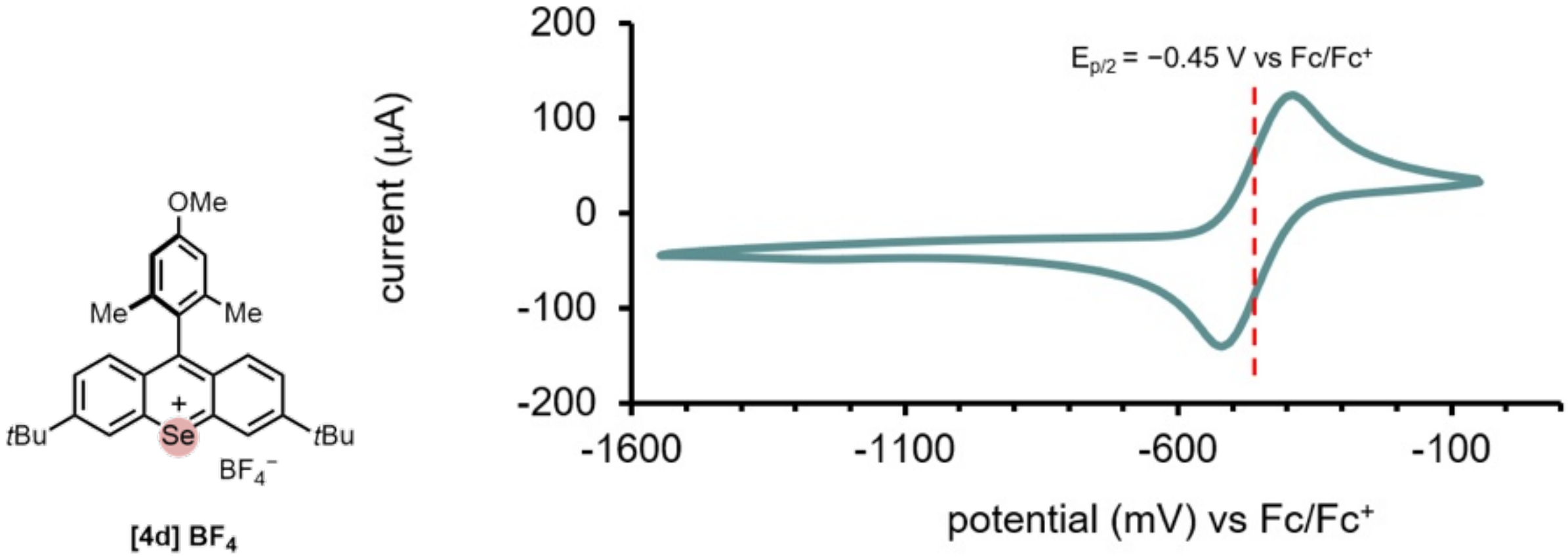

Figure S24. Cathodic CV of **[4d] BF_4** in a 0.10 M NBu_4PF_6 CH_3CN solution. GC electrode. Scan rate: 100 mV/s, potential referred to $Ag/AgNO_3$ (0.10 M in CH_3CN) using Fc/Fc^+ as internal reference at 298 K with a platinum disk as counter electrode.

Oxidation – ($[4d]^+$): $E_{p/2}$ = +1.46 V vs Fc/Fc^+ (= +1.84 V vs. SCE).

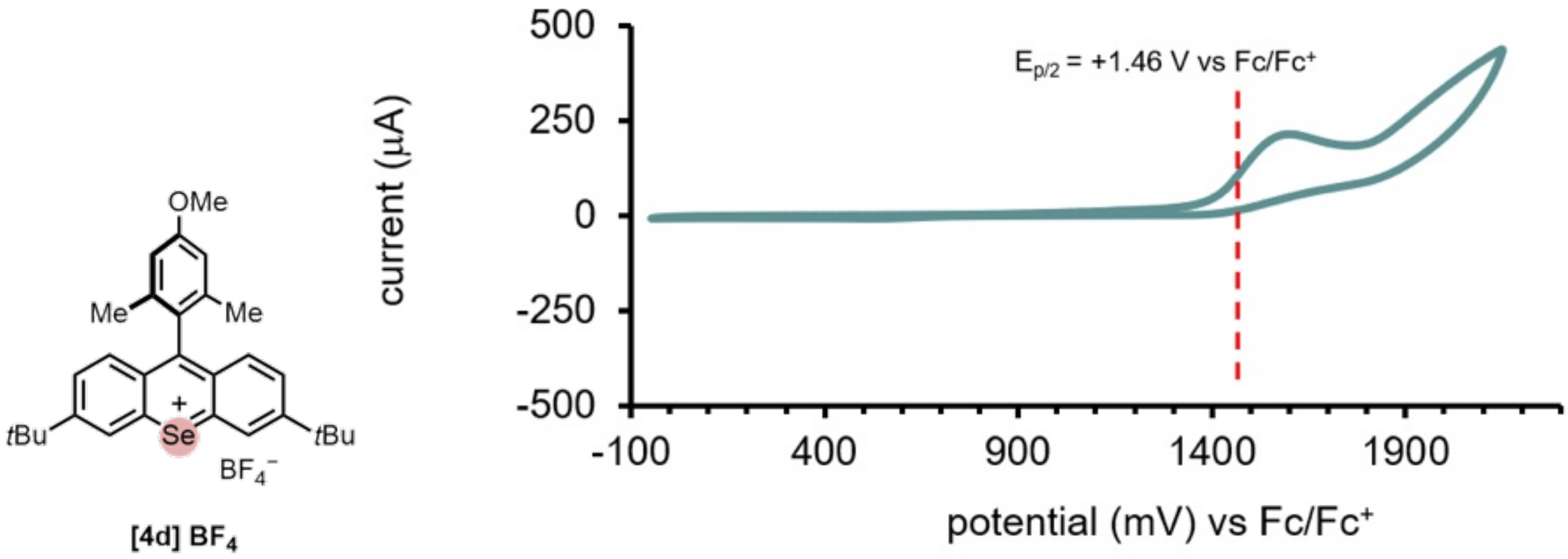

Figure S25. Anodic CV of **[4d] BF_4** in a 0.10 M NBu_4PF_6 CH_3CN solution. GC electrode. Scan rate: 100 mV/s, potential referred to $Ag/AgNO_3$ (0.10 M in CH_3CN) using Fc/Fc^+ as internal reference at 298 K with a platinum disk as counter electrode.

Electrochemical properties comparison – O vs S vs Se (i.e., [2a] vs [3a] vs [4a])

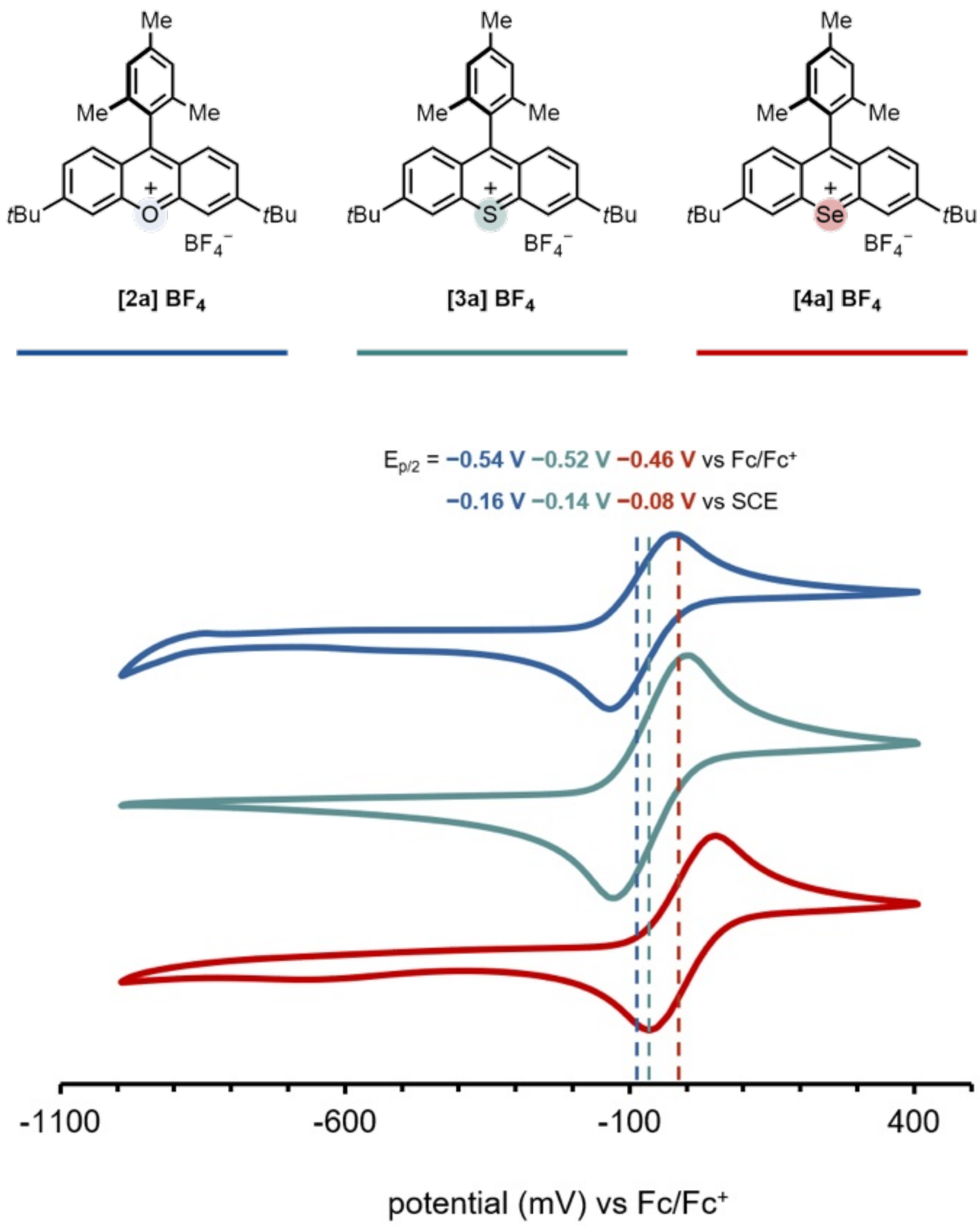

Figure S26. Cathodic CV comparison between **[2a] BF_4** (blue traces), **[3a] BF_4** (green traces), and **[4a] BF_4** (blue traces) in a 0.10 M NBu_4PF_6 CH_3CN solution. GC electrode. Scan rate: 100 mV/s, potential referred to Ag/$AgNO_3$ (0.10 M in CH_3CN) using Fc/Fc$^+$ as internal reference at 298 K with a platinum disk as counter electrode.

Electrochemical properties comparison – Reduction – F vs H vs Me vs OMe (i.e., [4a] vs [4b] vs [4c] vs [4d])

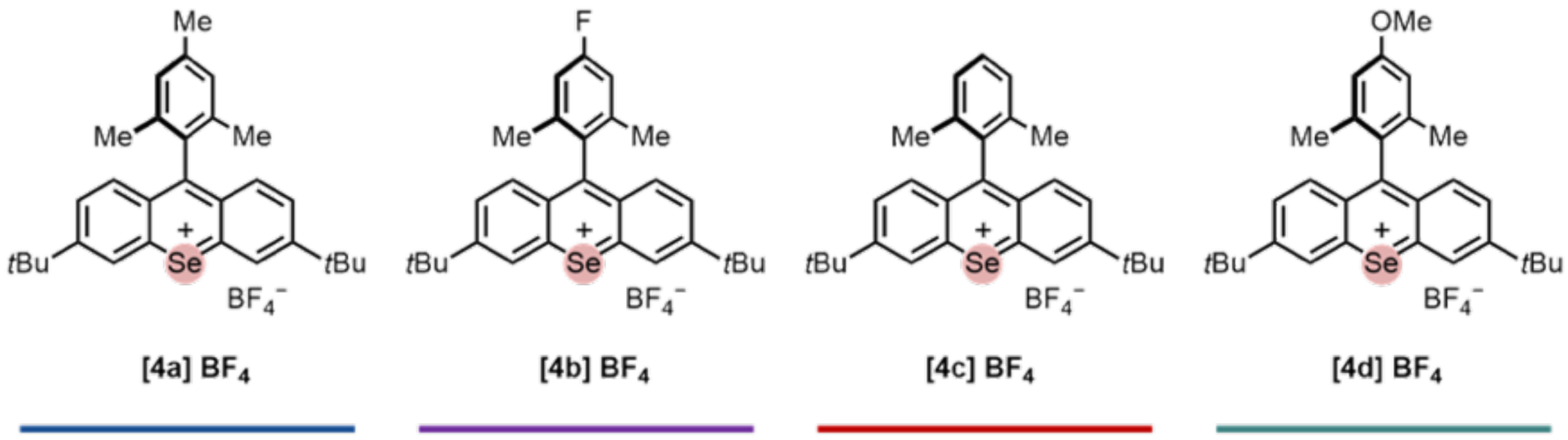

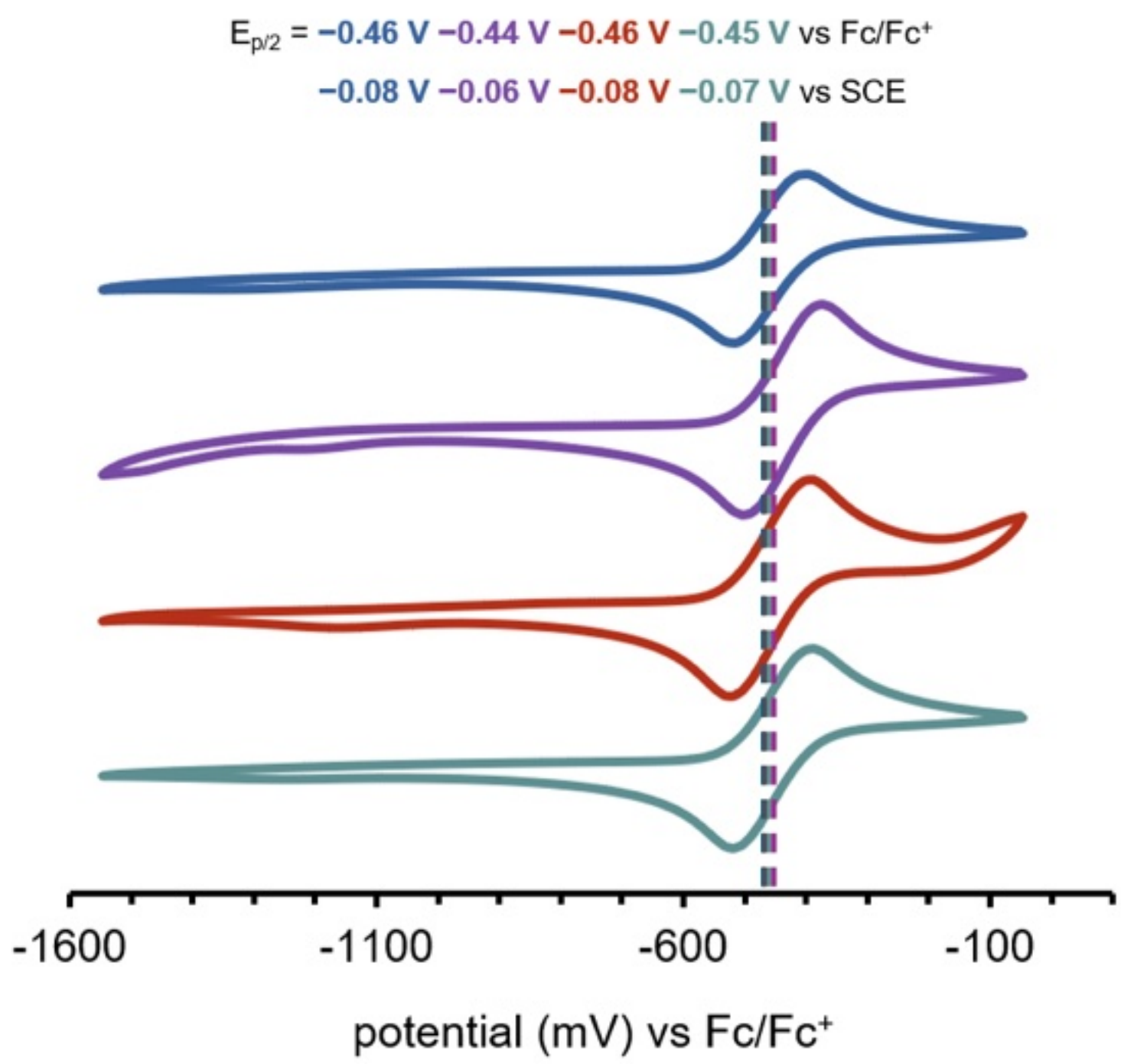

Figure S27. Cathodic CV comparison between **[4a] BF4** (blue traces), **[4b] BF4** (purple traces), **[4c] BF4** (red traces) and **[4d] BF4** (green traces) in a 0.10 M NBu_4PF_6 CH_3CN solution. GC electrode. Scan rate: 100 mV/s, potential referred to $Ag/AgNO_3$ (0.10 M in CH_3CN) using Fc/Fc^+ as internal reference at 298 K with a platinum disk as counter electrode.

Spectrophotometric Data

3,6-Di-*tert*-butyl-9-mesitylacridinium tetrafluoroborate ([1a] BF_4)

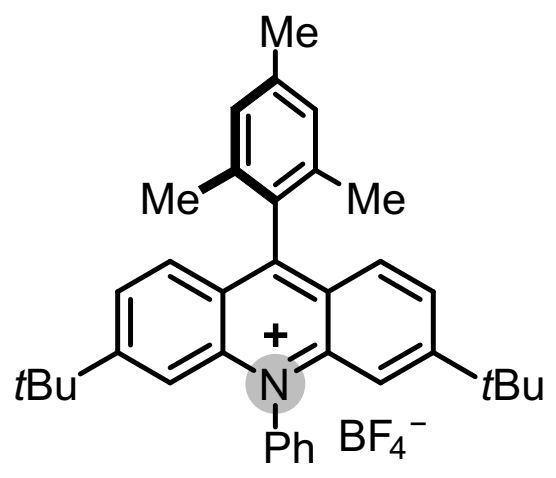

[1a] BF_4

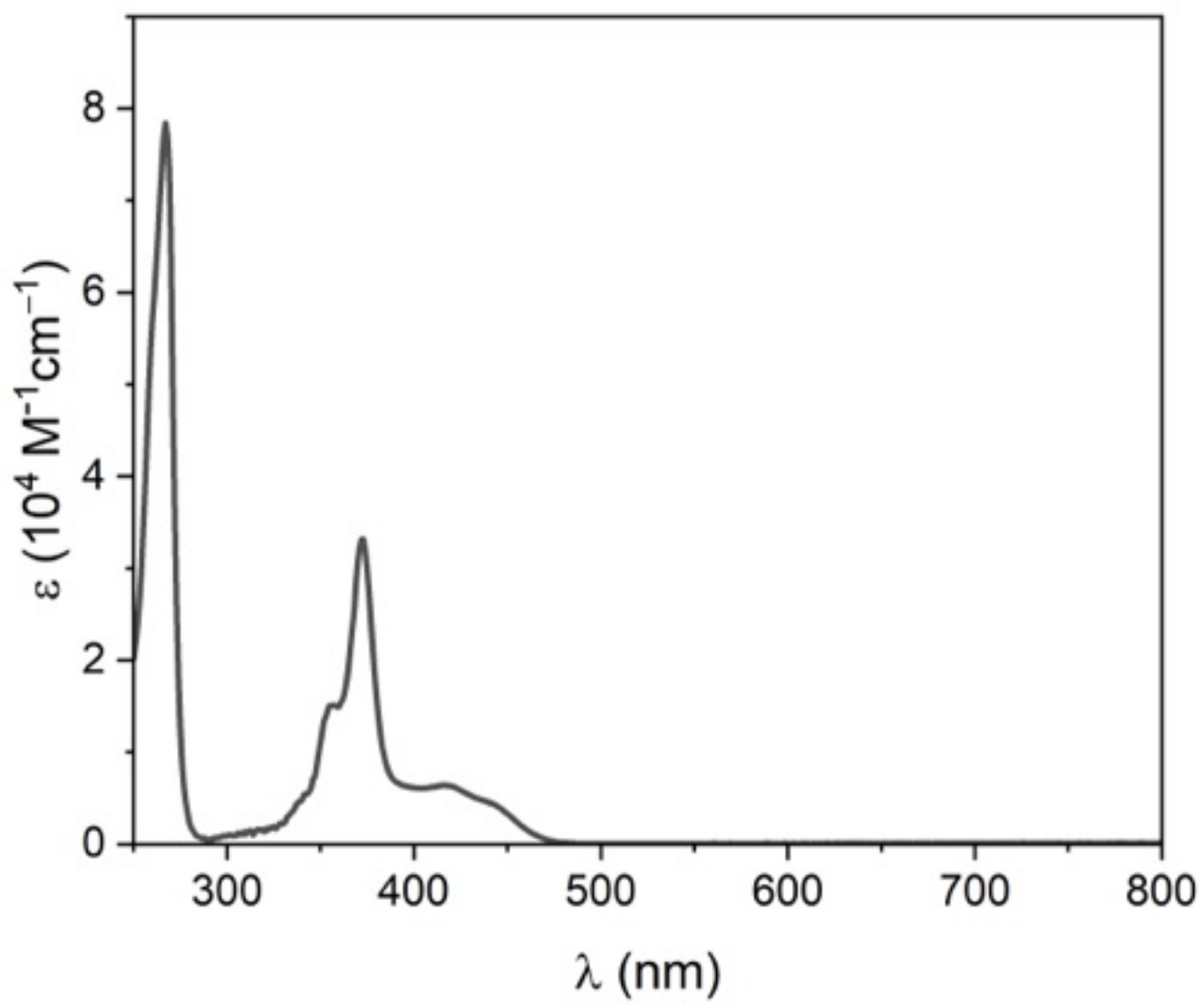

Figure S28. Absorption spectrum of **[1a] BF_4** in CH_3CN.

3,6-Di-*tert*-butyl-9-mesitylxanthylium tetrafluoroborate ([2a] BF_4)

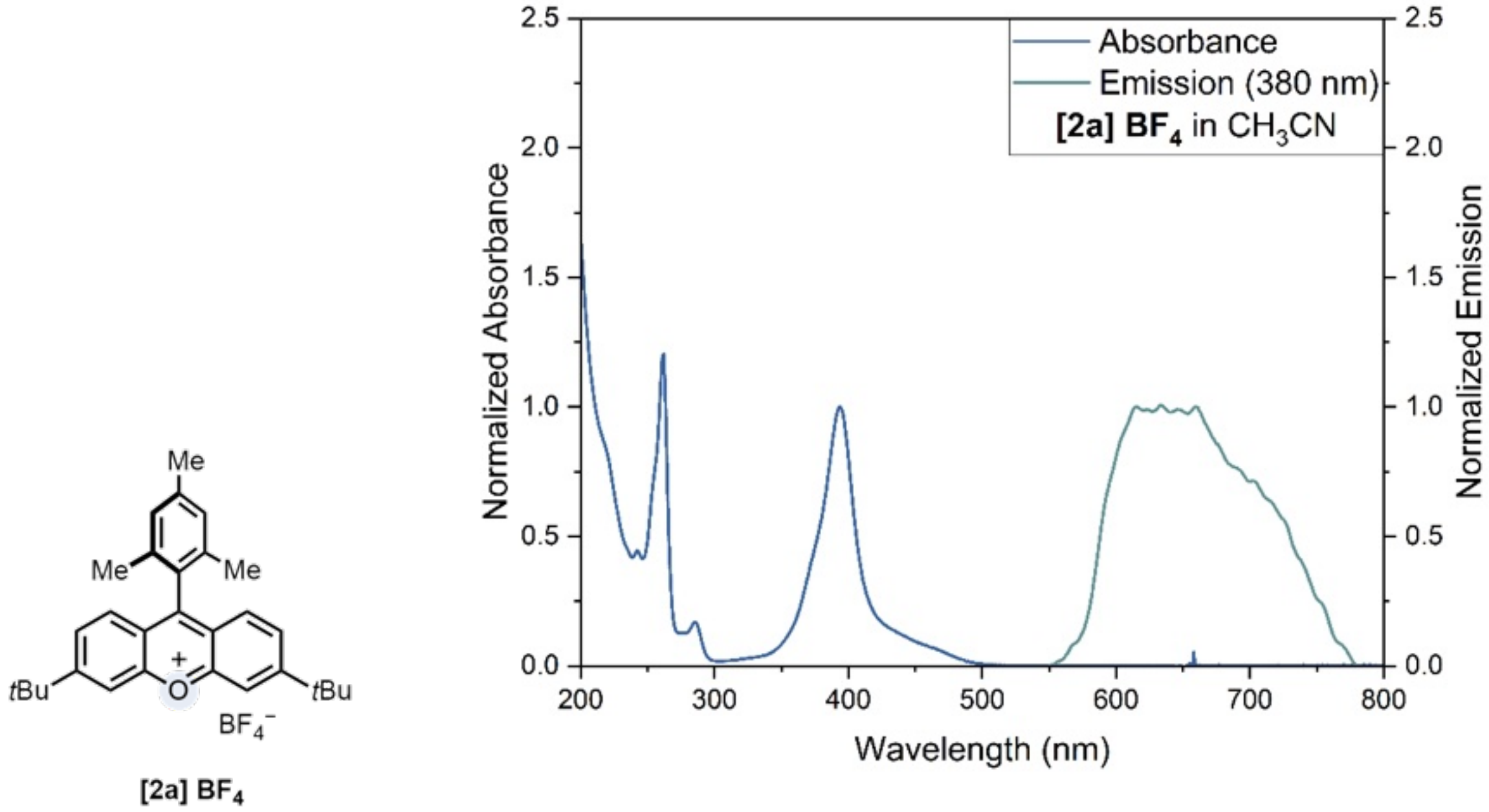

Figure S29. Absorption (blue traces, normalized to 394 nm) and emission (λ_{exc}= 380 nm, green traces, normalized to 615 nm) spectrum of **[2a] BF_4** in CH_3CN.

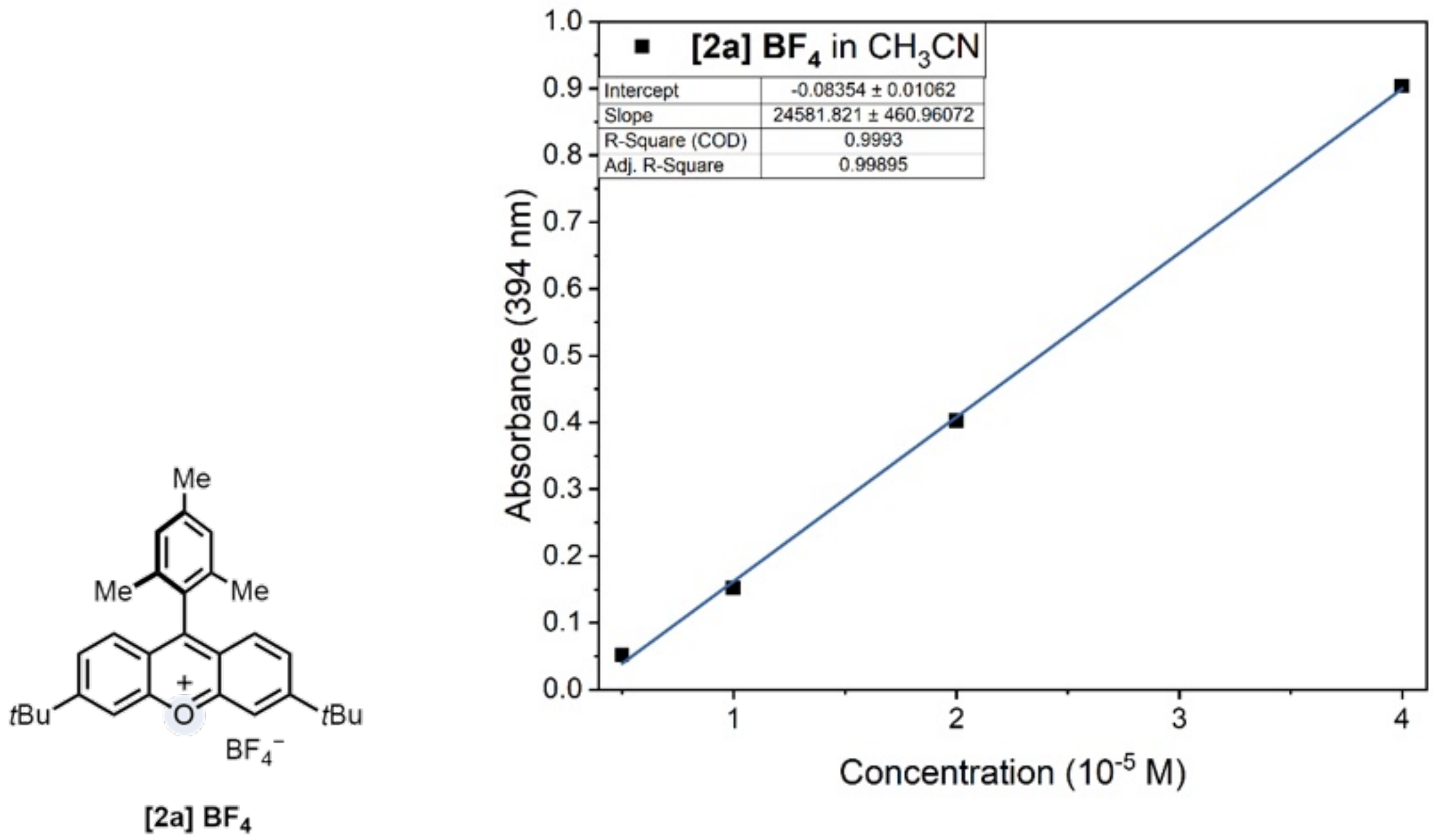

Figure S30. Linear fit of absorbances of **[2a] BF_4** measured at 394 nm in CH_3CN.

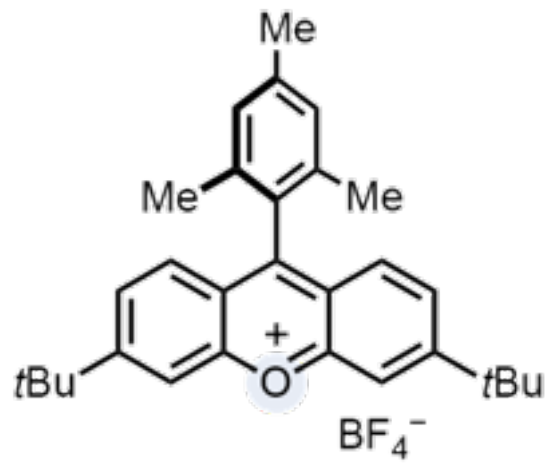

[2a] BF_4

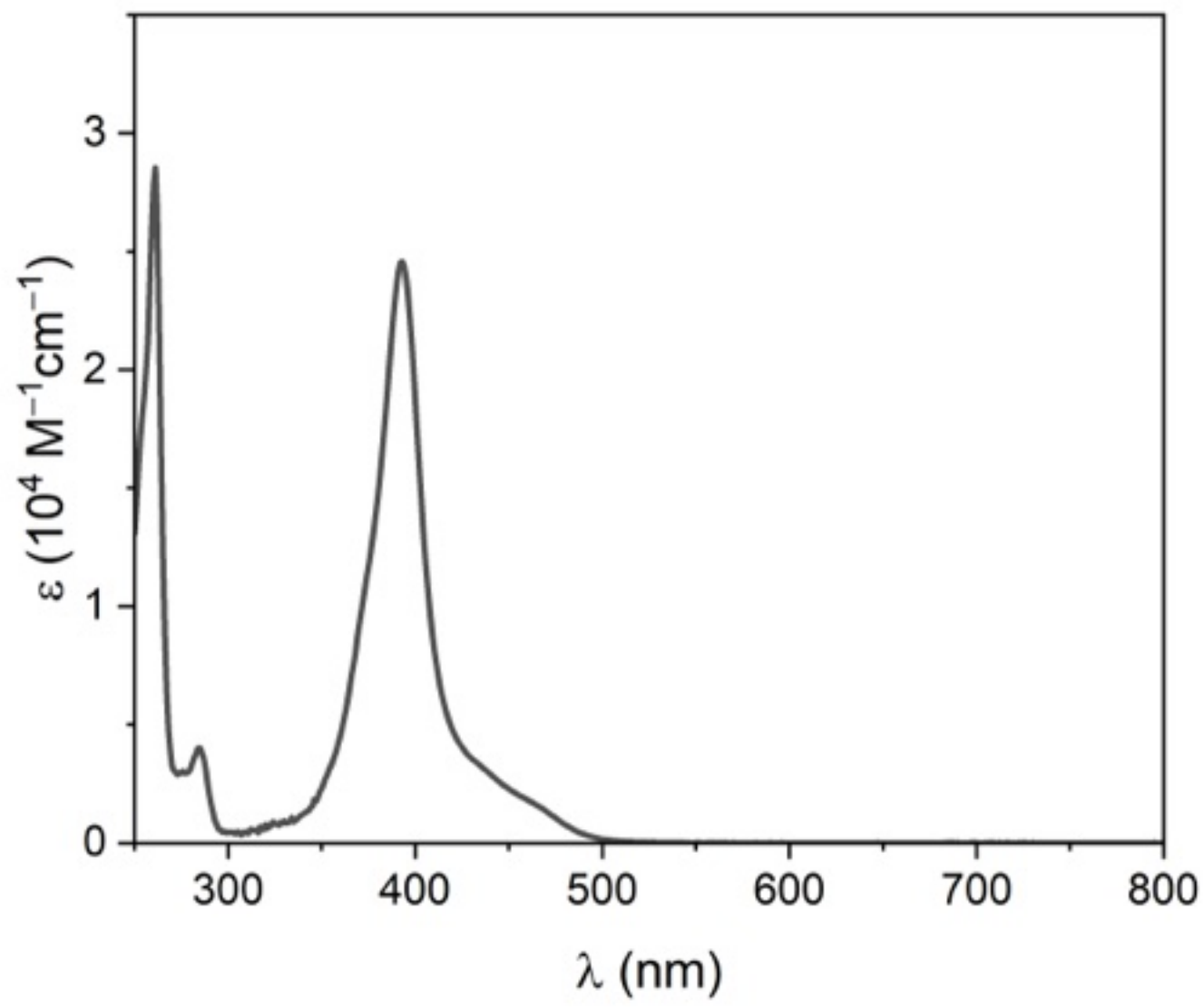

Figure S31. Absorption spectrum of **[2a] BF_4** in CH_3CN.

Summary

ε (394 nm) = 24582 M^{-1} cm^{-1}

$E_{0,0}$ = 2.62 eV [17]

E_{red} (**[2a]**$^+$/**[2a]**) = −0.16 V vs SCE

E^*_{red} (**[2a]**$^{*+}$/**[2a]**) = 2.62 + (−0.16) V = **+2.46 V vs. SCE**

3,6-Di-*tert*-butyl-9-mesitylthioxanthylium tetrafluoroborate ([3a] BF_4)

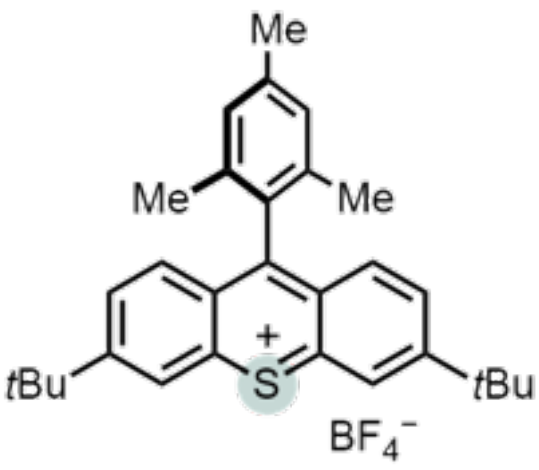

[3a] BF_4

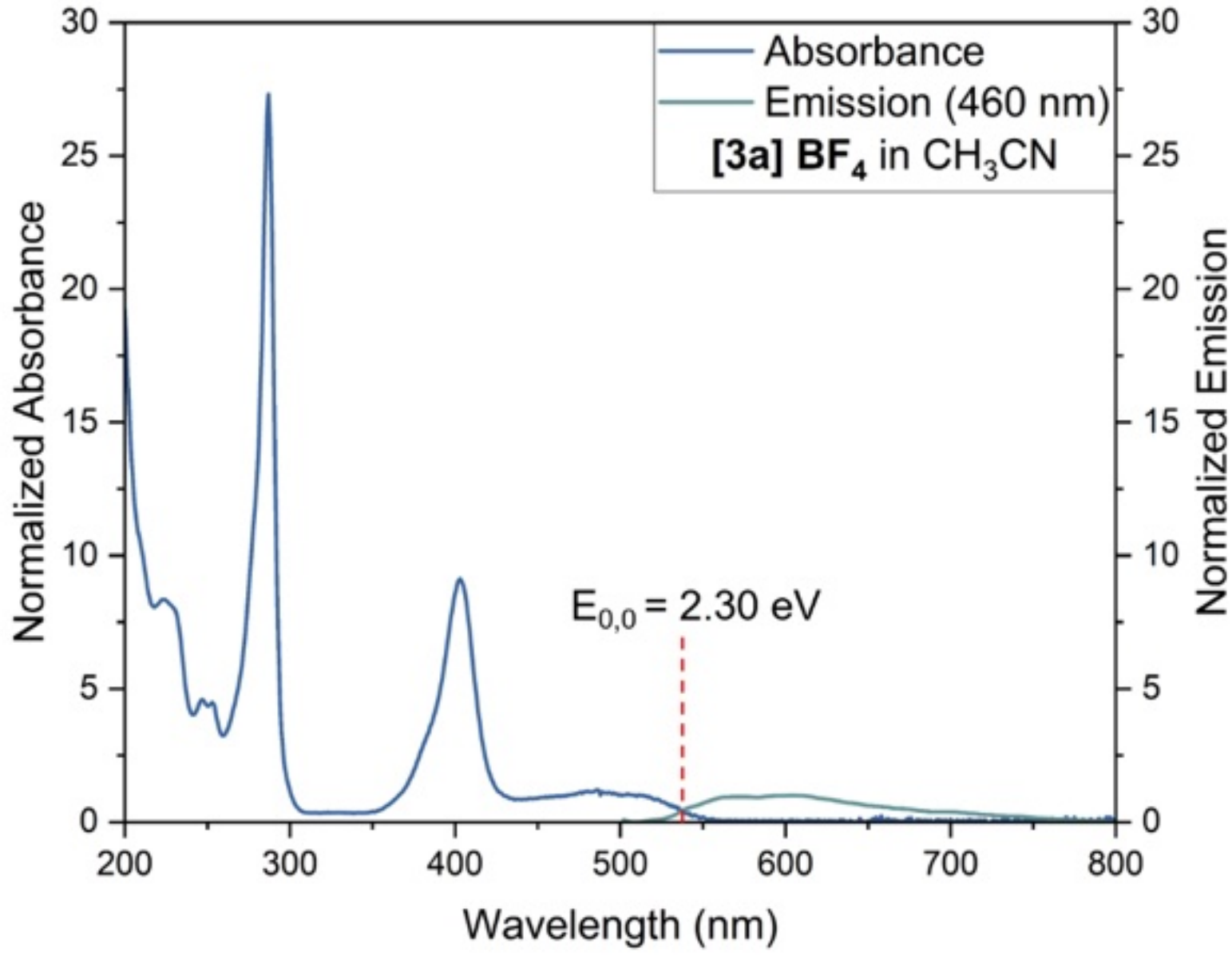

Figure S32. Absorption (blue traces, normalized to 512 nm) and emission (λ_{exc}= 460 nm, green traces, normalized to 605 nm) spectrum of **[3a] BF_4** in CH_3CN.

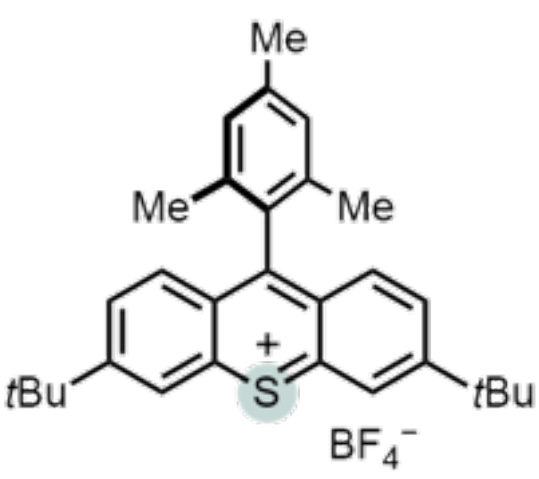

[3a] BF_4

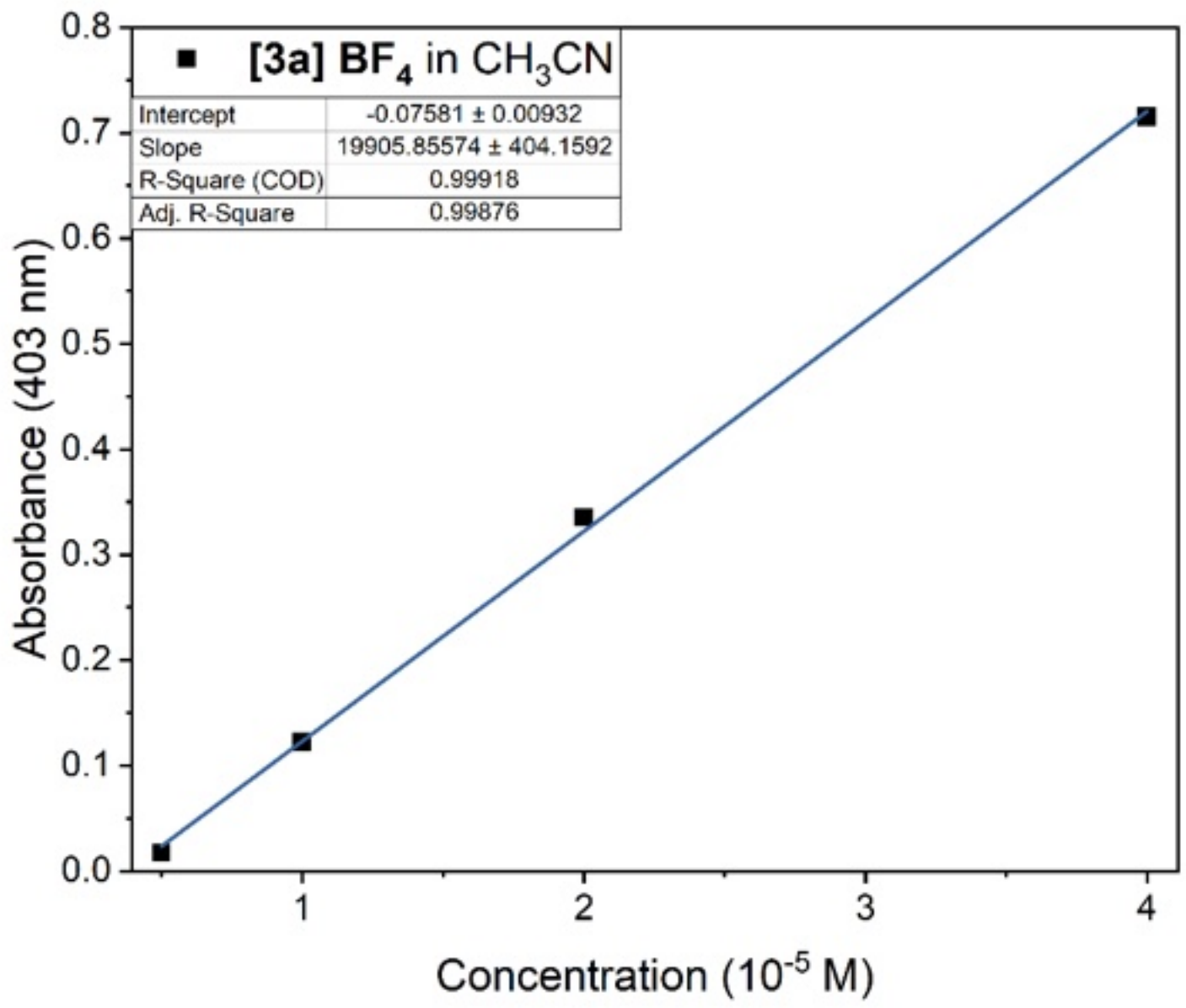

Figure S33. Linear fit of absorbances of **[3a] BF_4** measured at 403 nm in CH_3CN.

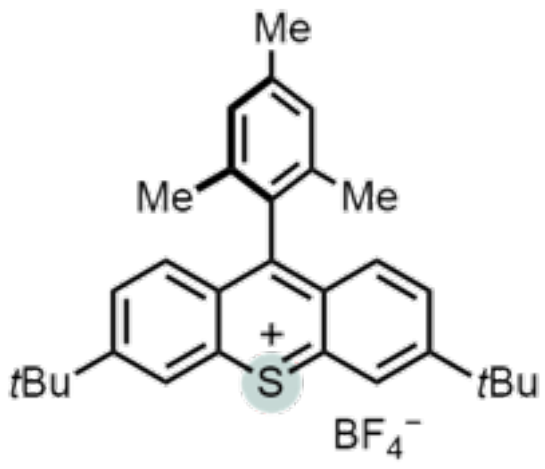

[3a] BF4

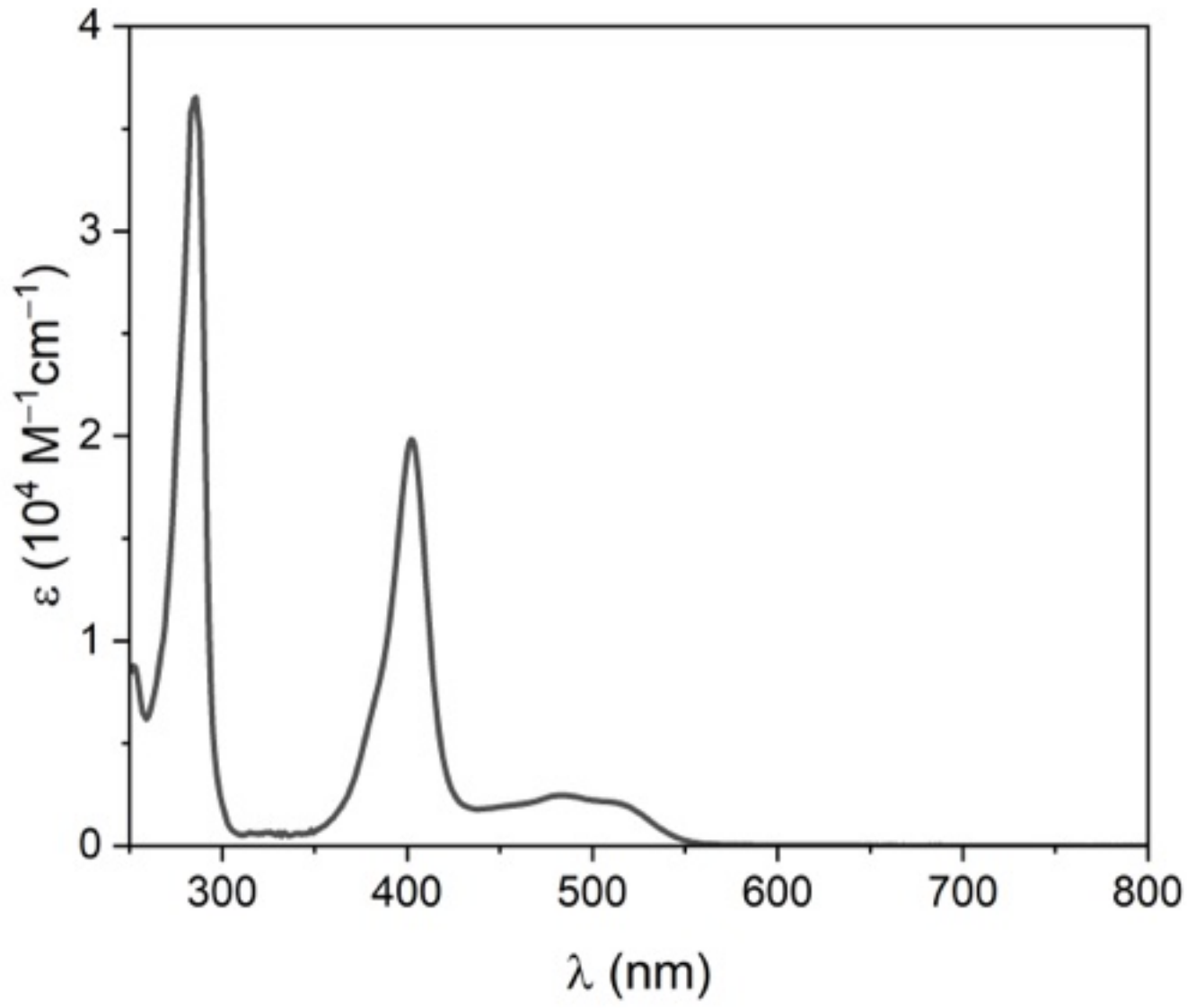

Figure S34. Absorption spectrum of **[3a] BF**$_4$ in CH_3CN.

Summary

ε (403 nm) = 19906 M^{-1} cm^{-1}

$E_{0,0}$ = 2.30 eV

E_{red} (**[3a]**$^+$/**[3a]**$^{\cdot}$) = −0.14 V vs SCE

$^{S}E^*_{red}$ (^{S}**[3a]**$^{*+}$/**[3a]**$^{\cdot}$) = 2.30 + (−0.14) V = **+2.16 V vs SCE**

3,6-Di-*tert*-butyl-9-mesitylselenoxanthylium tetrafluoroborate ([4a] BF_4)

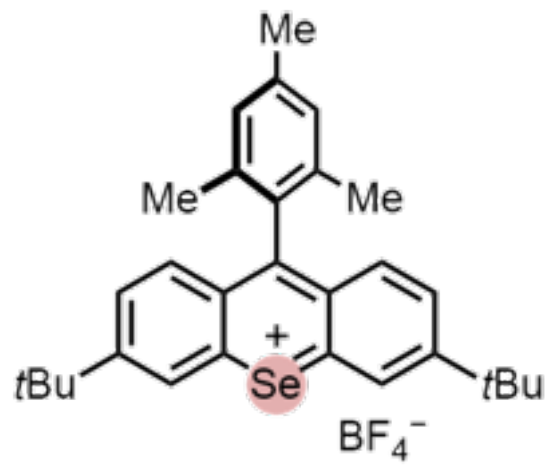

[4a] BF_4

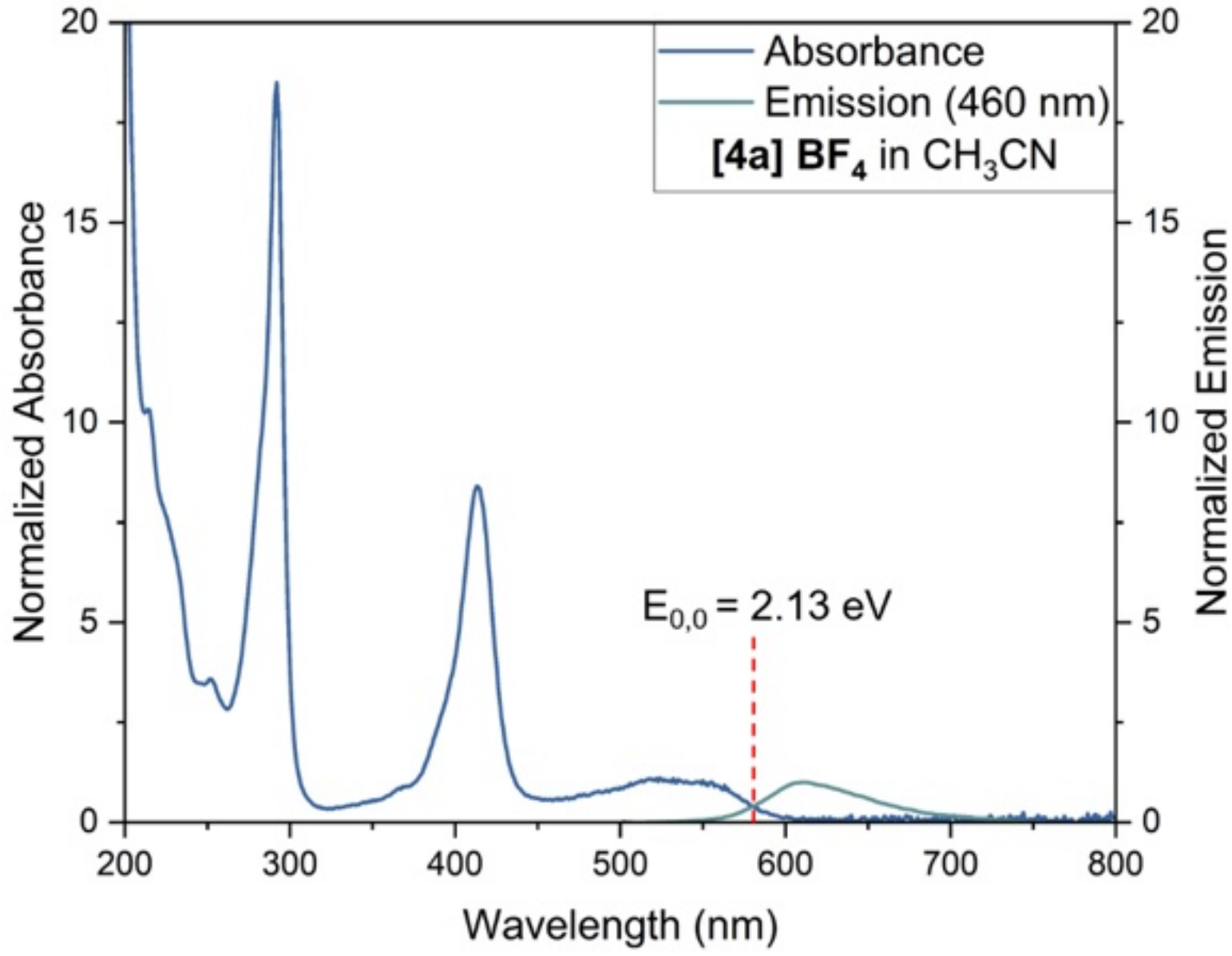

Figure S35. Absorption (blue traces, normalized to 555 nm) and emission (λ_{exc}= 460 nm, green traces, normalized to 610 nm) spectrum of **[4a] BF_4** in CH_3CN.

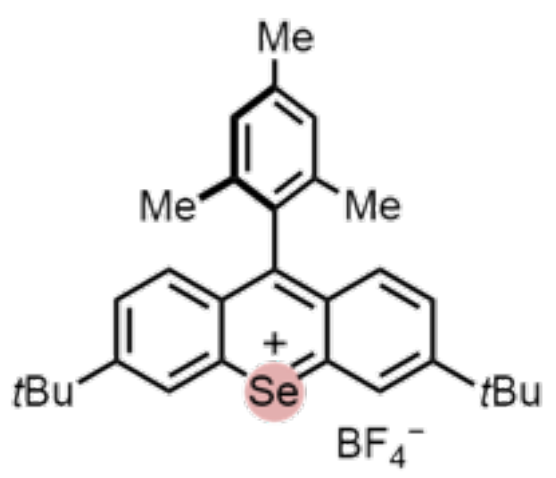

[4a] BF_4

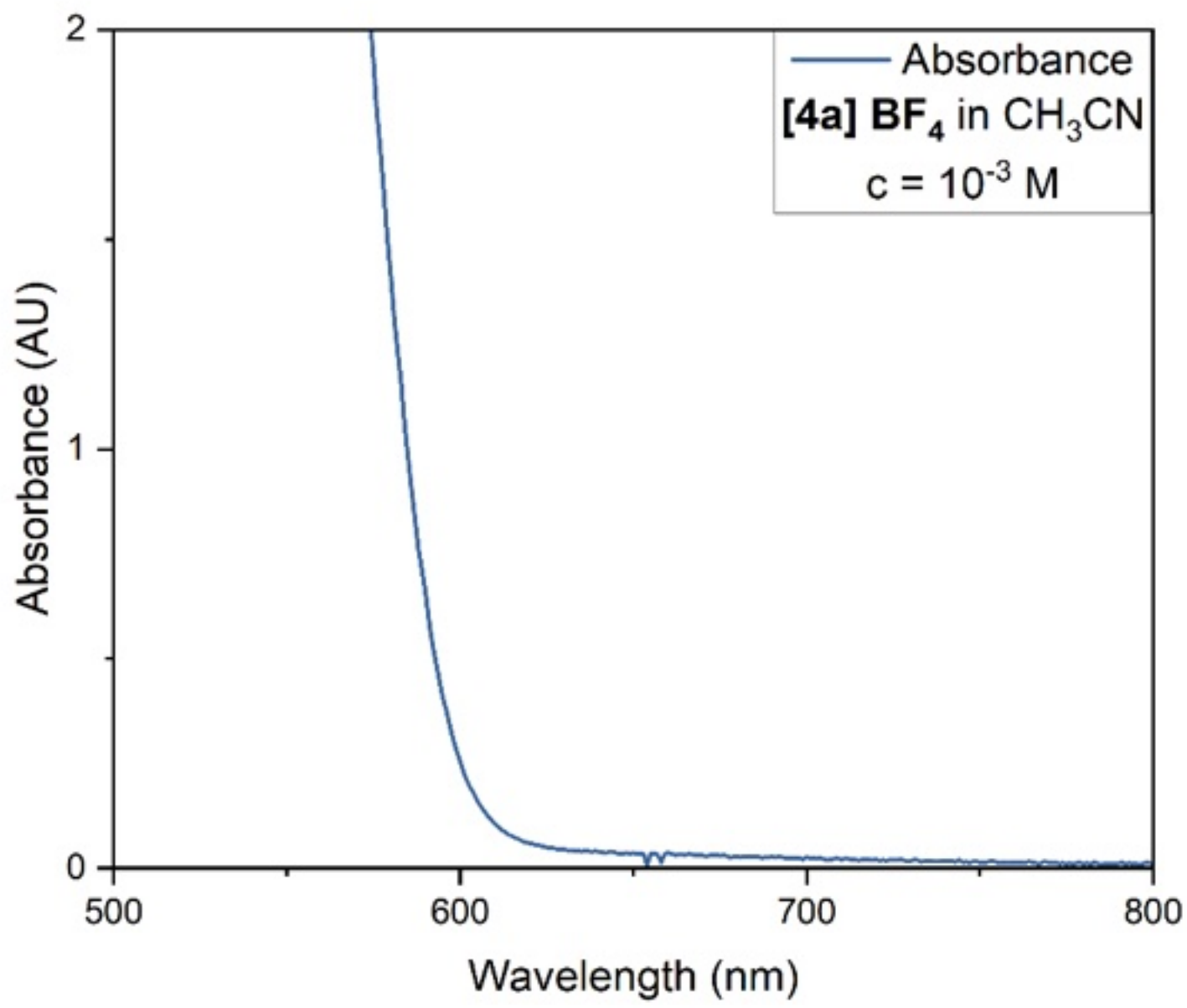

Figure S36. Absorption spectrum (blue traces) of **[4a] BF_4** at c = 10^{-3} M in CH_3CN.

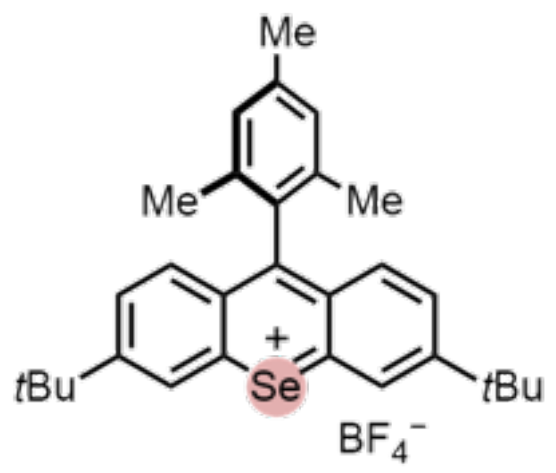

[4a] BF$_4$

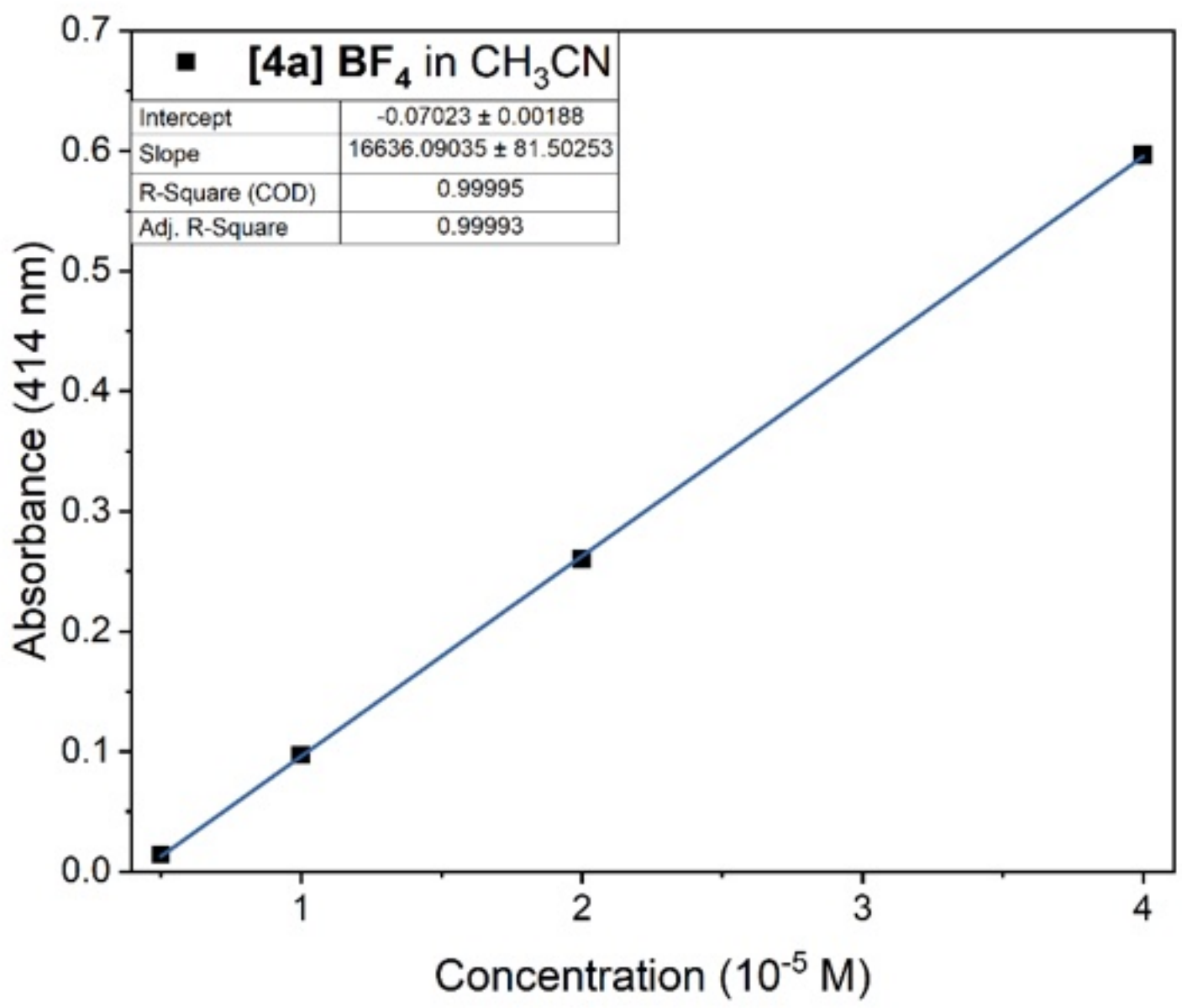

Figure S37. Linear fit of absorbances of **[4a] BF$_4$** measured at 414 nm in CH_3CN.

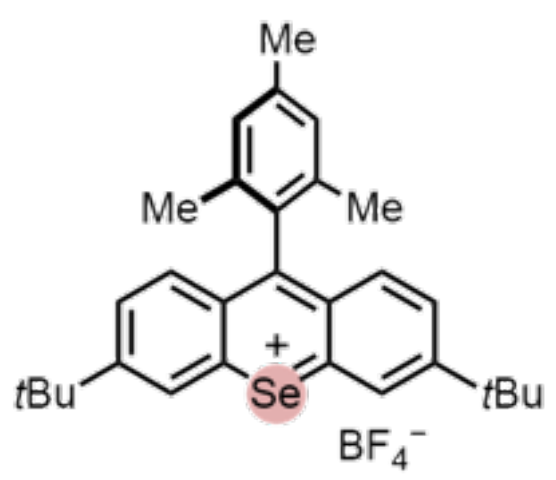

[4a] BF$_4$

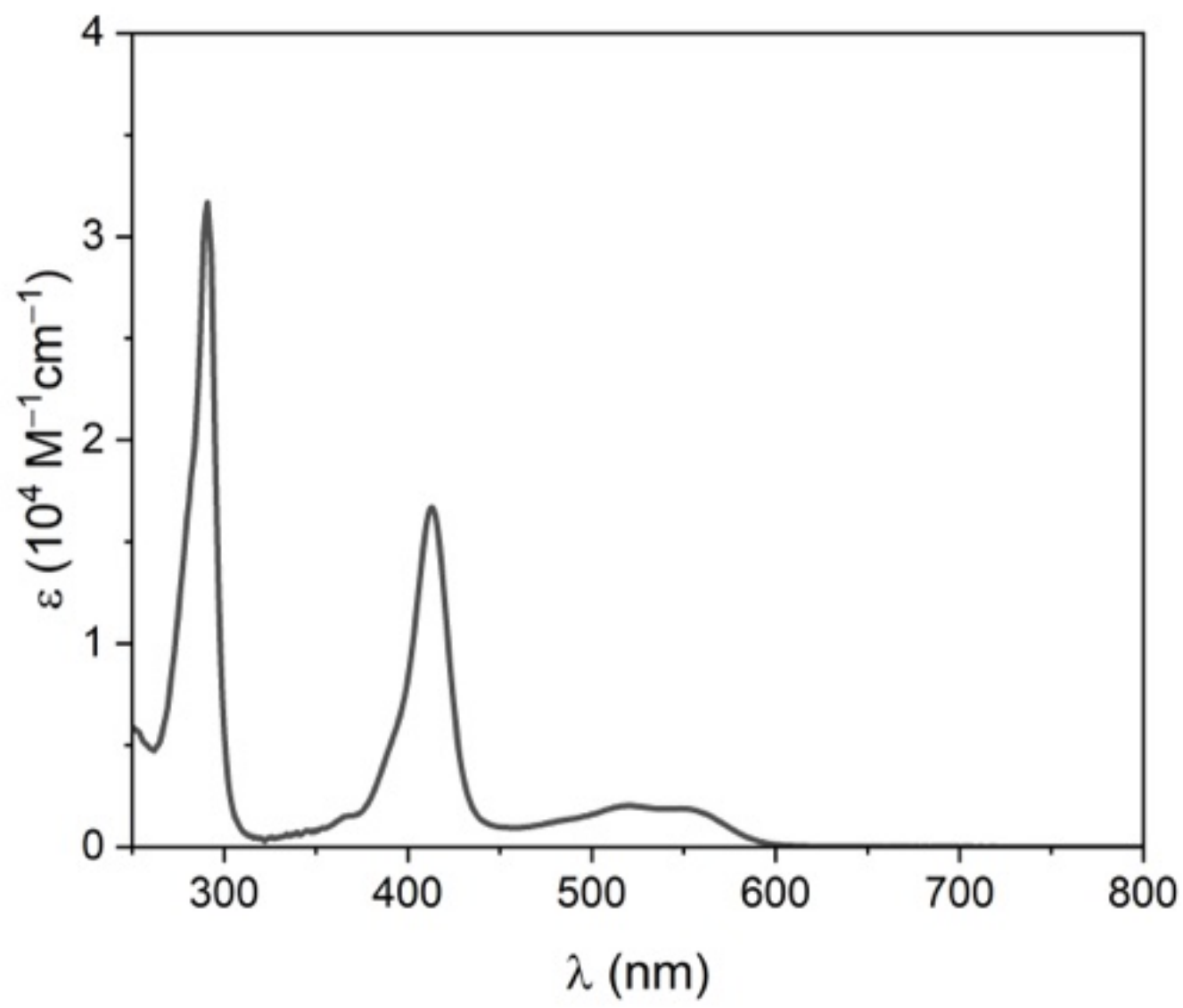

Figure S38. Absorption spectrum of **[4a] BF$_4$** in CH_3CN.

Summary

ε (414 nm) = 16636 M^{-1} cm^{-1}

$E_{0,0}$ = 2.13 eV

E_T = 1.62 eV

τ_T = 1.7 μs

E_{red} (**[4a]**$^+$/**[4a]**$^·$) = −0.08 V vs SCE

$^SE^*_{red}$ (^{S}**[4a]**$^{*+}$/**[4a]**$^·$) = 2.13 + −0.08 V = **+2.05 V vs SCE**

$^TE^*_{red}$ (^{T}**[4a]**$^{*+}$/**[4a]**$^·$) = 1.62 + −0.08 V = **+1.54 V vs SCE**

3,6-Di-*tert*-butyl-9-(4-fluoro-2,6-dimethylphenyl)selenoxanthylium tetrafluoroborate ([4b] BF_4)

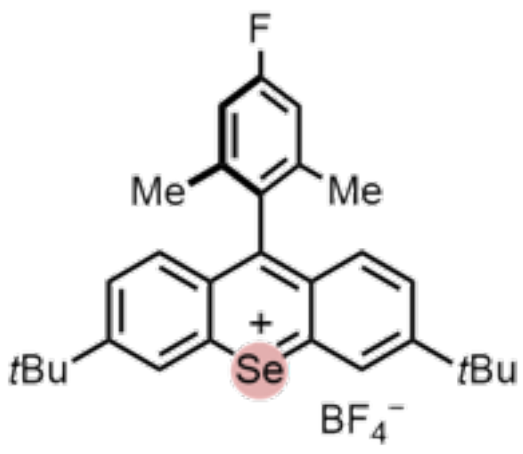

[4b] BF_4

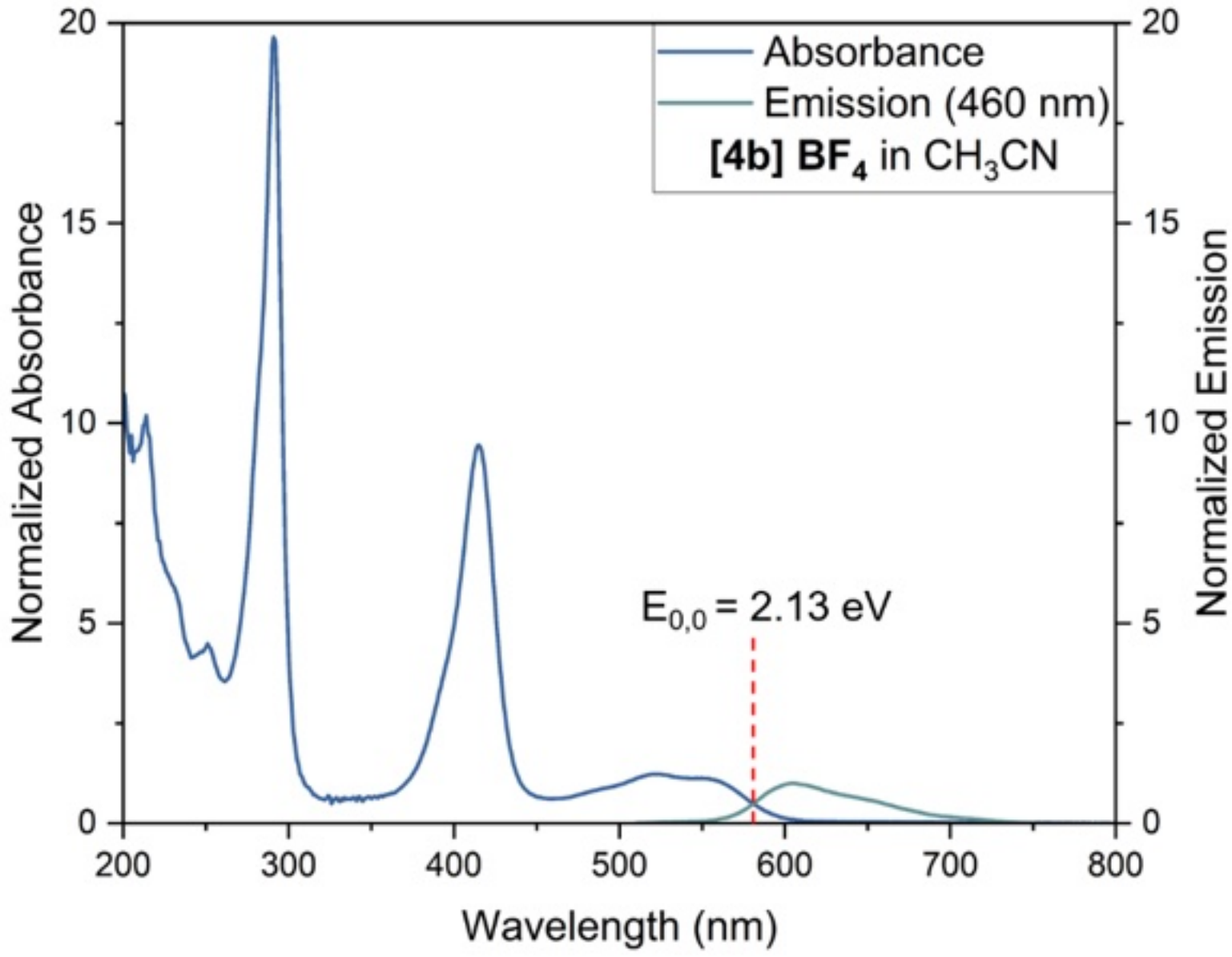

Figure S39. Absorption (blue traces, normalized to 555 nm) and emission (λ_{exc}= 460 nm, green traces, normalized to 610 nm) spectrum of **[4a] BF_4** in CH_3CN.

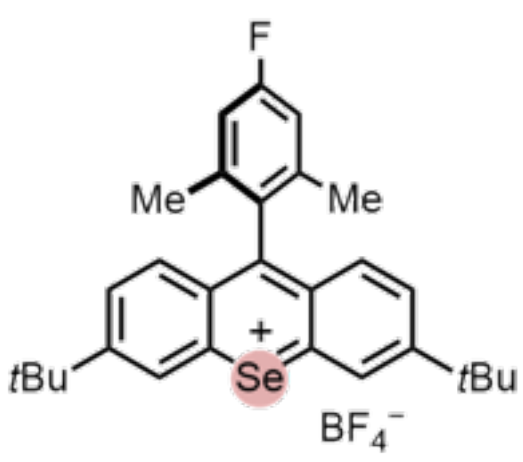

[4b] BF_4

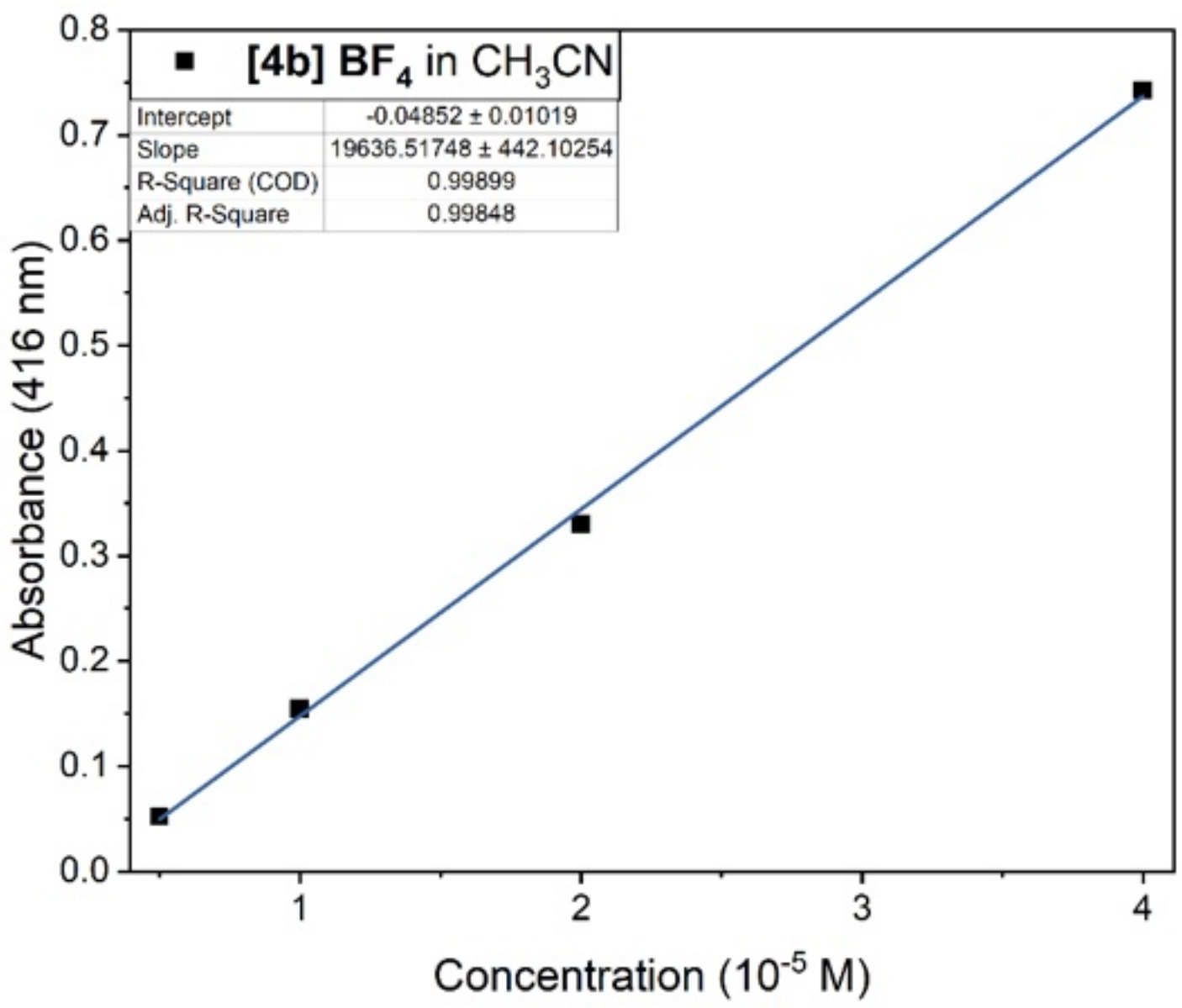

Figure S40. Linear fit of absorbances of **[4b] BF_4** measured at 416 nm in CH_3CN.

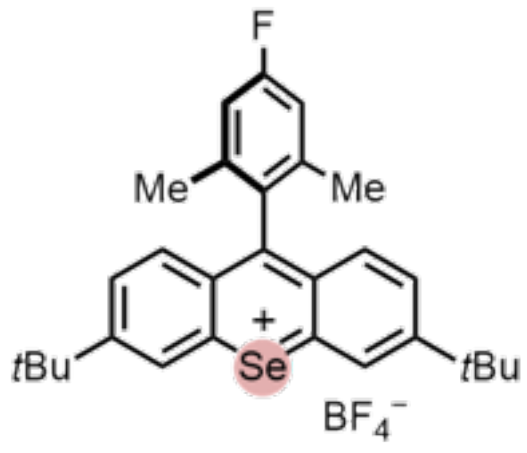

[4b] BF$_4$

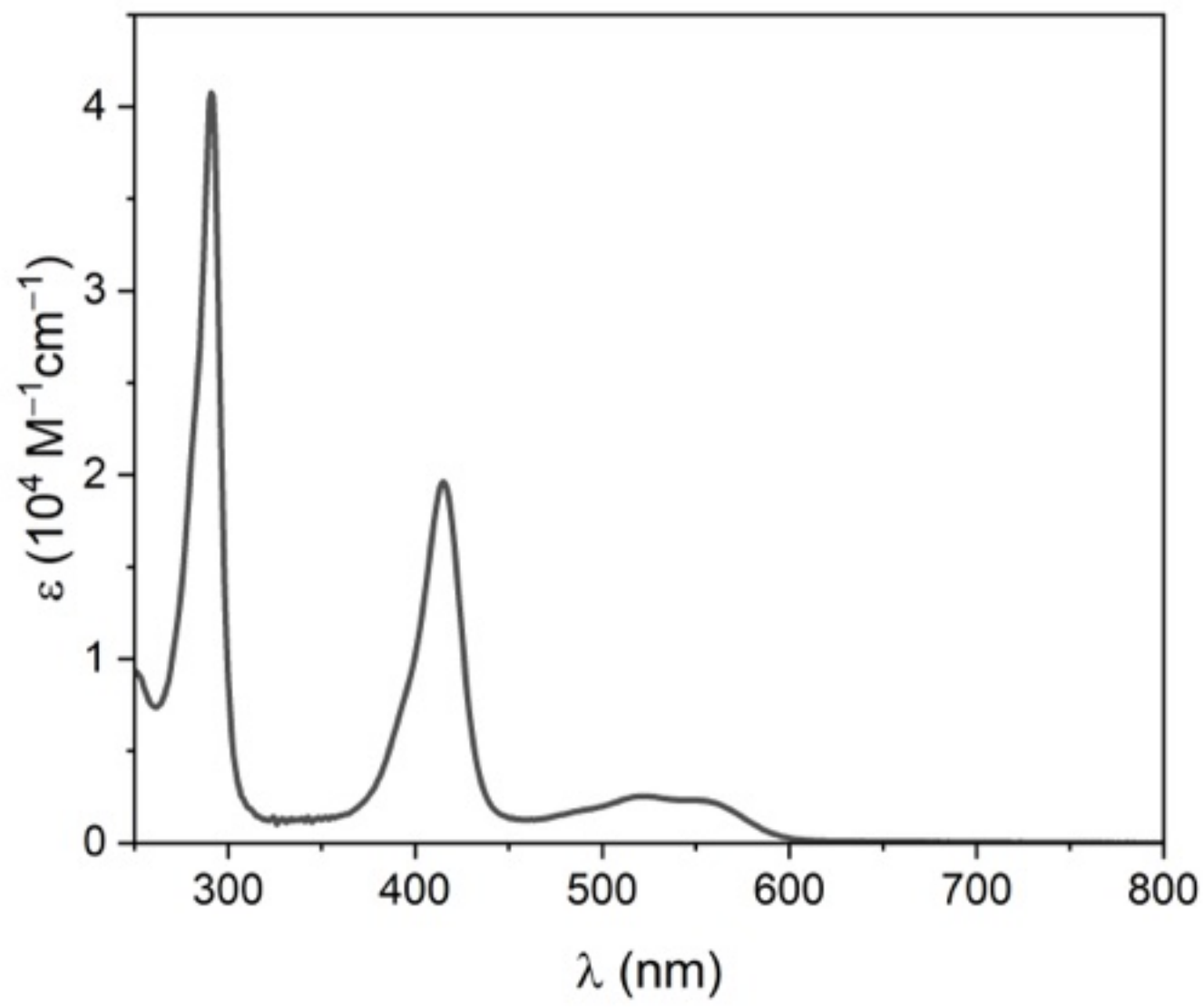

Figure S41. Absorption spectrum of **[4b] BF$_4$** in CH_3CN.

Summary

ε (416 nm) = 19637 M^{-1} cm^{-1}

$E_{0,0}$ = 2.13 eV

E_T = 1.59 eV

τ_T = 4.9 μs

E_{red} (**[4b]**$^+$/**[4b]**$^\cdot$) = −0.06 V vs SCE

$^{S}E^*_{red}$ (^{S}**[4b]**$^{*+}$/**[4b]**$^\cdot$) = 2.13 + (−0.06 V) = **+2.07 V vs SCE**

$^{T}E^*_{red}$ (^{T}**[4b]**$^{*+}$/**[4b]**$^\cdot$) = 1.59 + (−0.06 V) = **+1.53 V vs SCE**

3,6-Di-*tert*-butyl-9-(2,6-dimethylphenyl)selenoxanthylium tetrafluoroborate ([4c] BF_4)

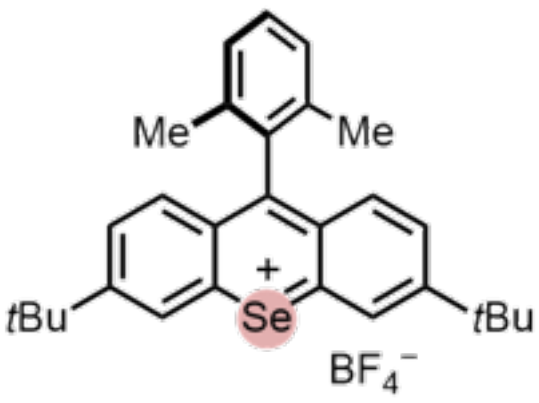

[4c] BF_4

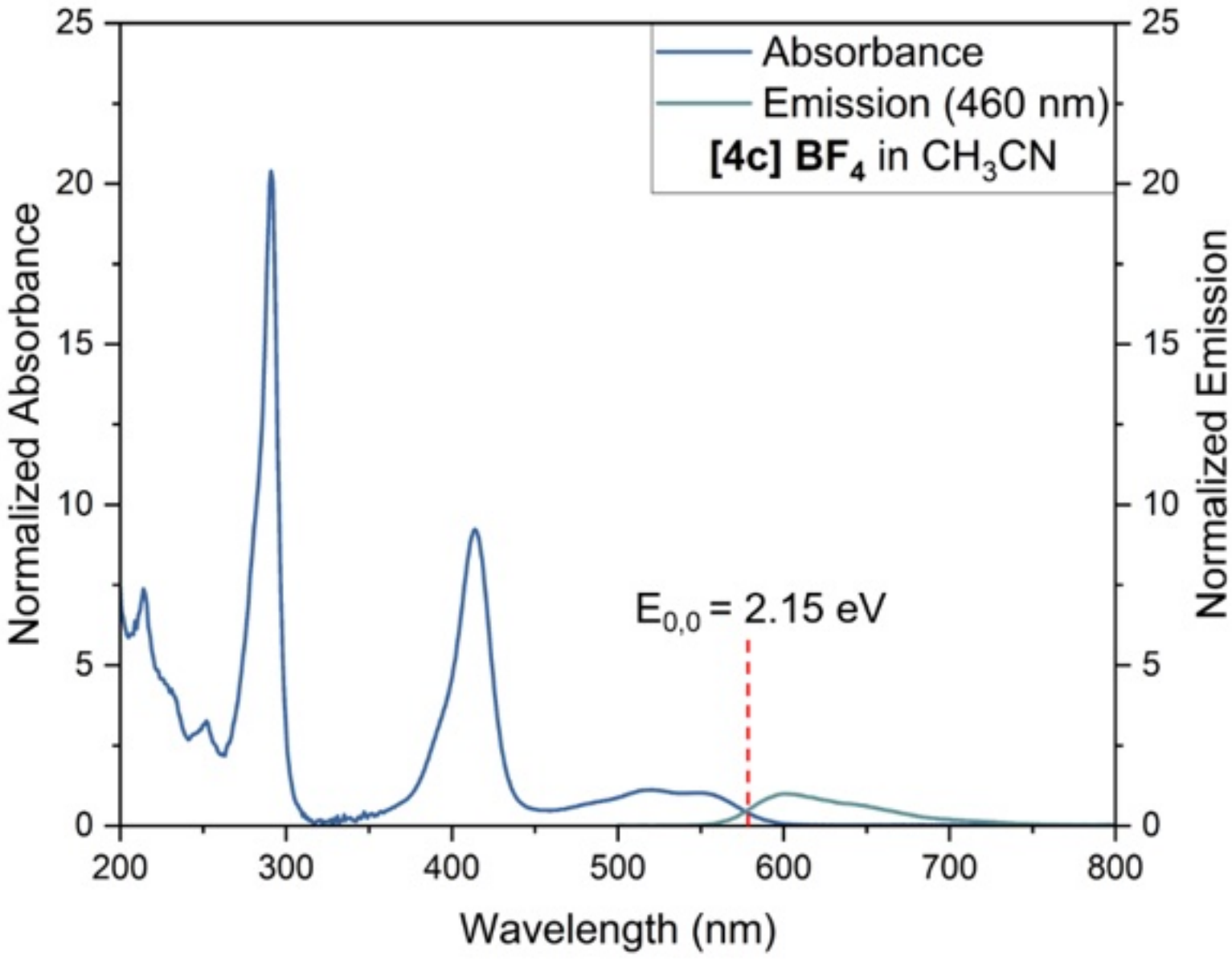

Figure S42. Absorption (blue traces, normalized to 555 nm) and emission (λ_{exc}= 460 nm, green traces, normalized to 600 nm) spectrum of **[4c] BF_4** in CH_3CN.

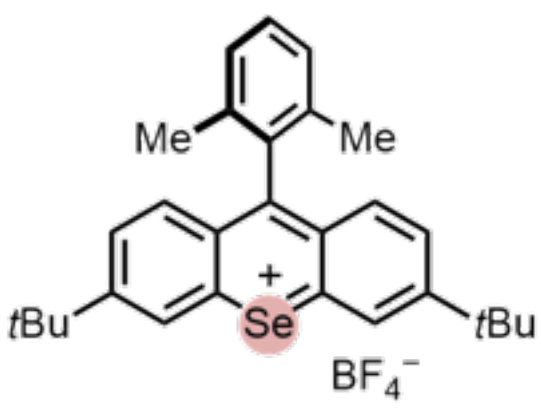

[4c] BF_4

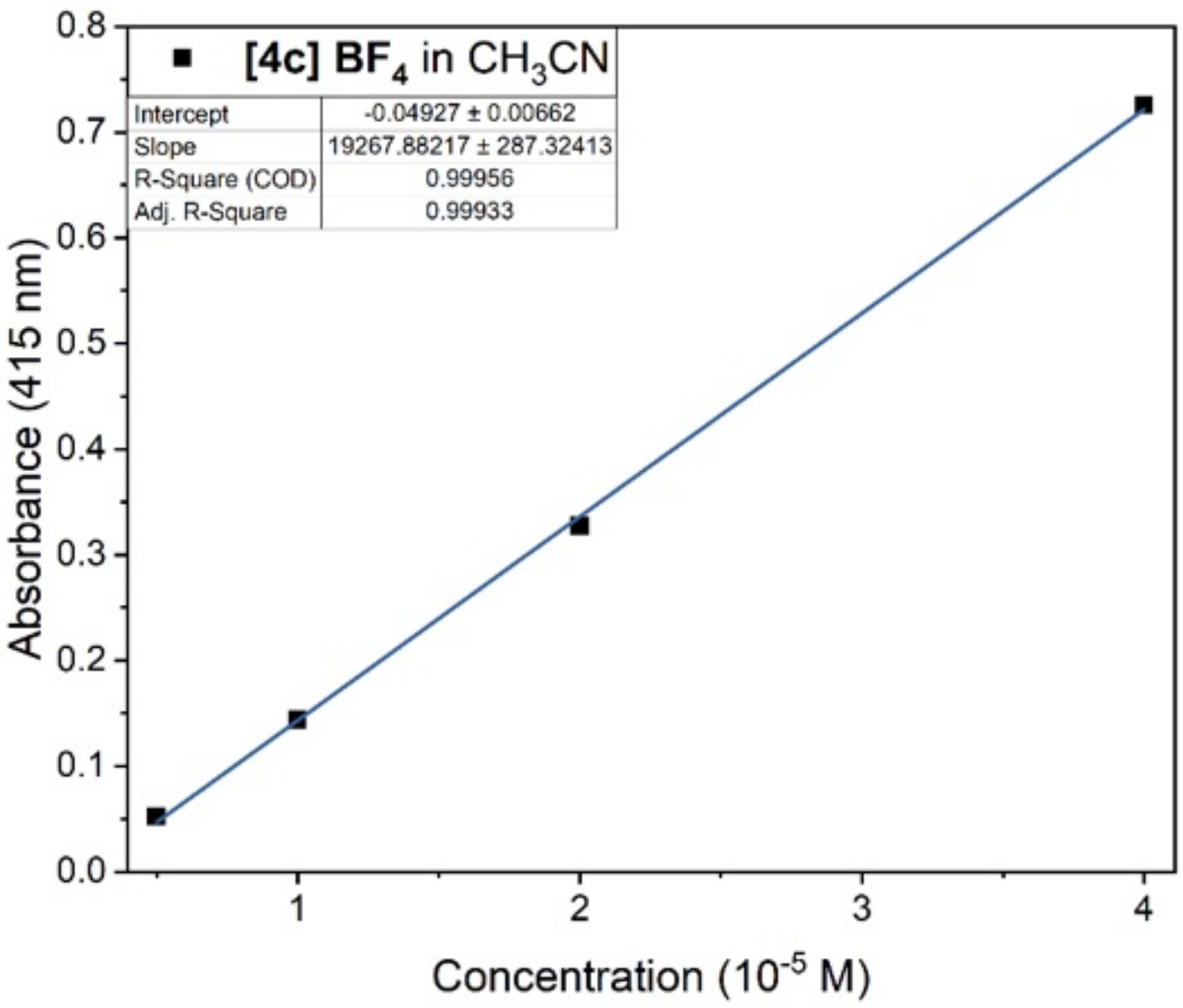

Figure S43. Linear fit of absorbances of **[4c] BF_4** measured at 415 nm in CH_3CN.

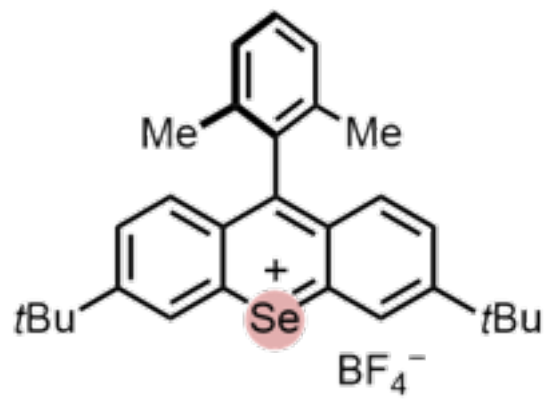

[4c] BF$_4$

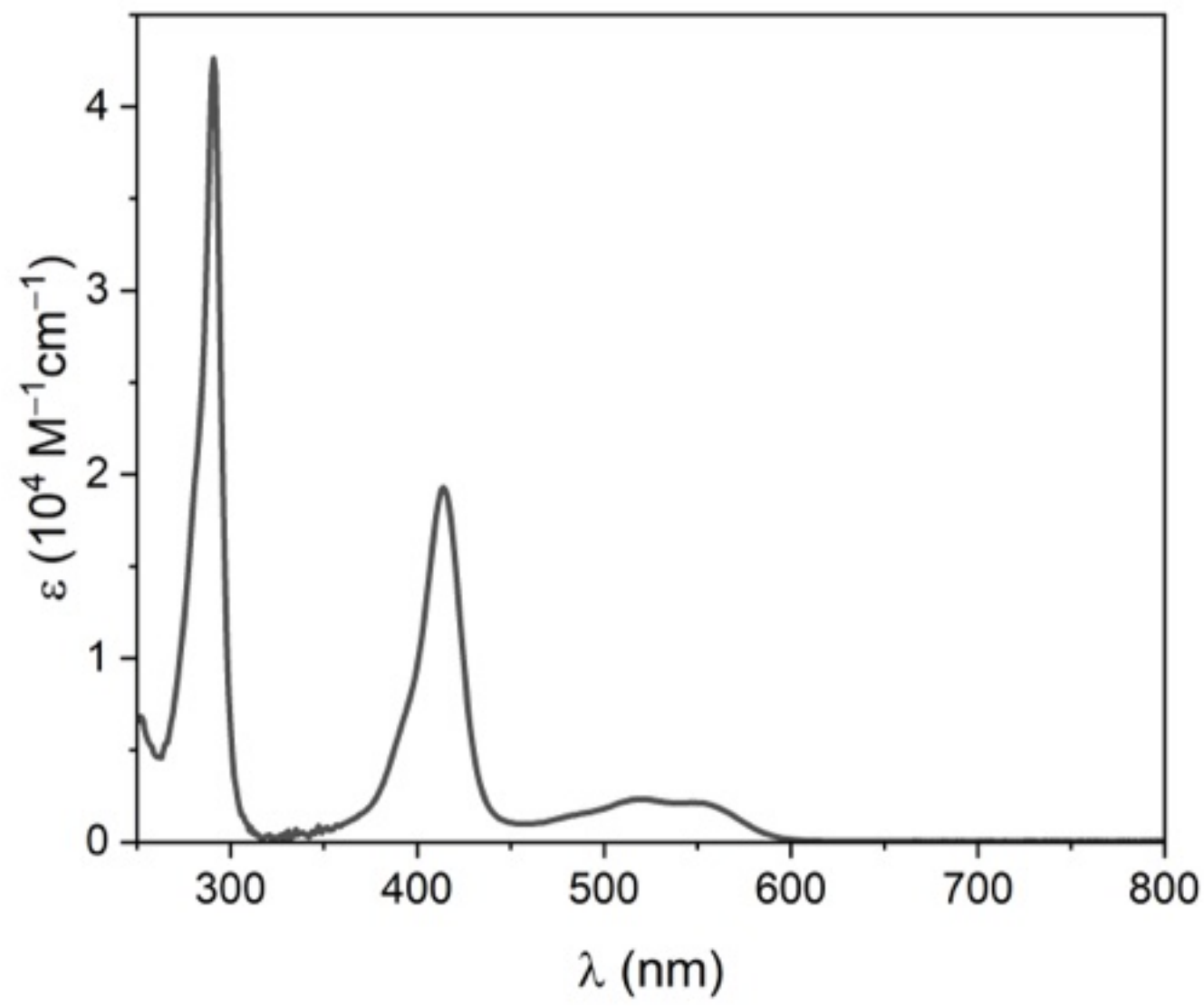

Figure S44. Absorption spectrum of **[4c] BF$_4$** in CH_3CN.

Summary

ε (415 nm) = 19268 M^{-1} cm^{-1}

$E_{0,0}$ = 2.15 eV

E_T = 1.61 eV

τ_T = 3.1 μs

E_{red} (**[4c]**$^+$/**[4c]**$^{\cdot}$) = −0.08 V vs SCE

$^{S}E^*_{red}$ ($^{\mathbf{S}}$**[4c]**$^{*+}$/**[4c]**$^{\cdot}$) = 2.15 + (−0.08 V) = **+2.07 V vs SCE**

$^{T}E^*_{red}$ ($^{\mathbf{T}}$**[4c]**$^{*+}$/**[4c]**$^{\cdot}$) = 1.61 + (−0.08 V) = **+1.53 V vs SCE**

3,6-Di-*tert*-butyl-9-(4-methoxy-2,6-dimethylphenyl)selenoxanthylium tetrafluoroborate ([4d] BF_4)

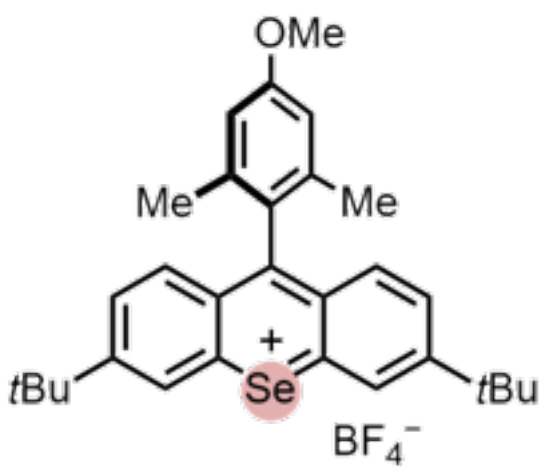

[4d] BF4

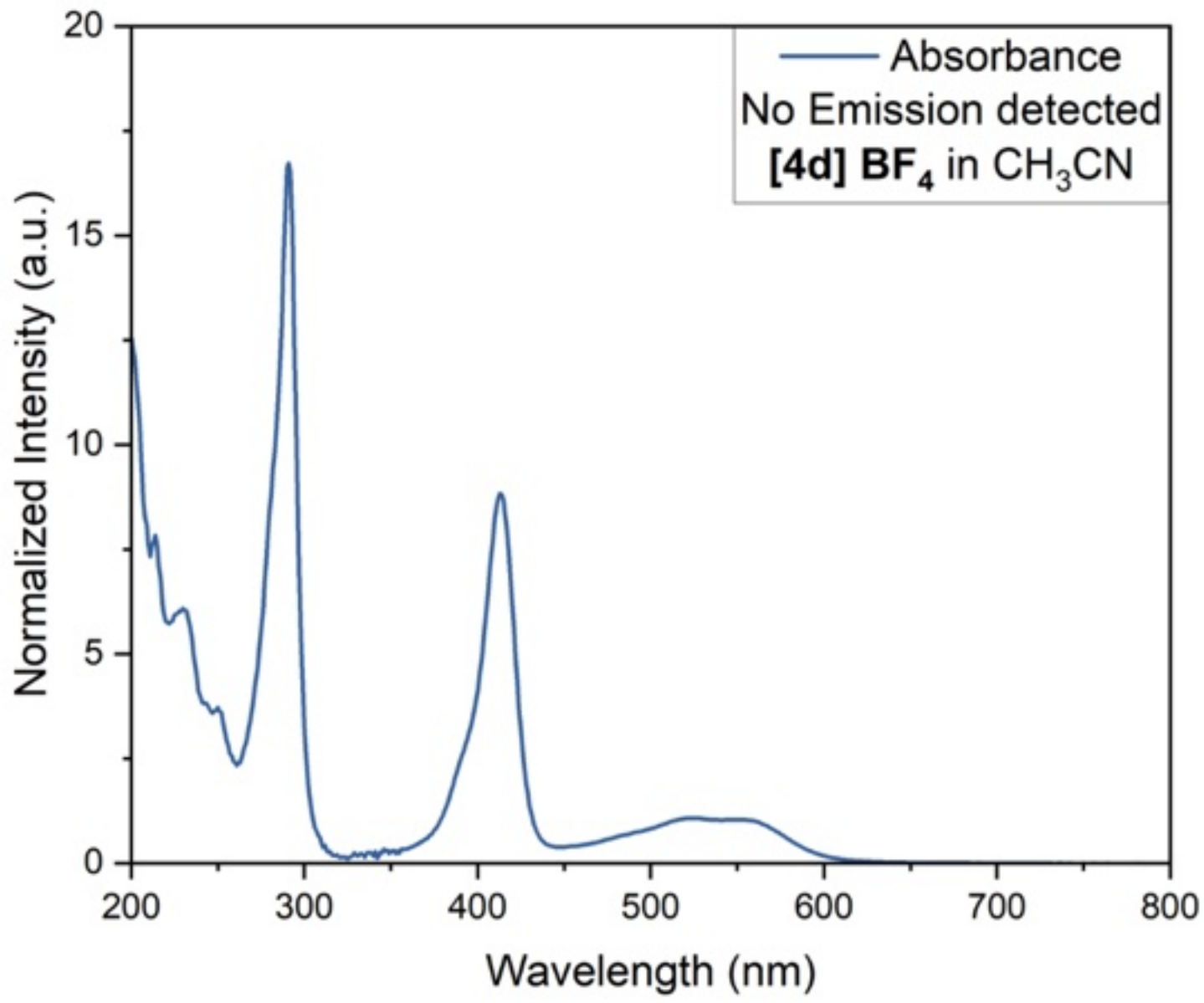

Figure S45. Absorption (blue traces, normalized to 560 nm) spectrum of **[4d] BF_4** in CH_3CN.

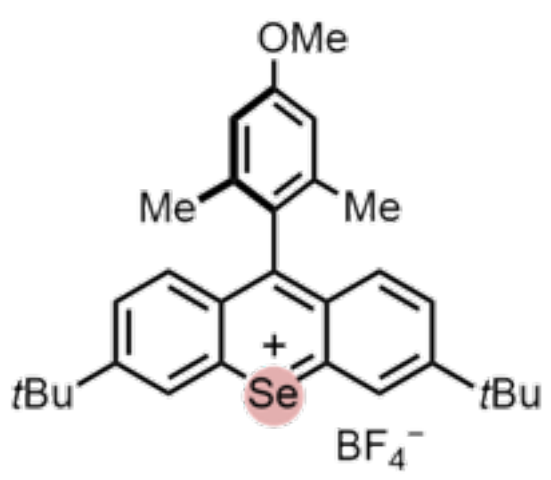

[4d] BF4

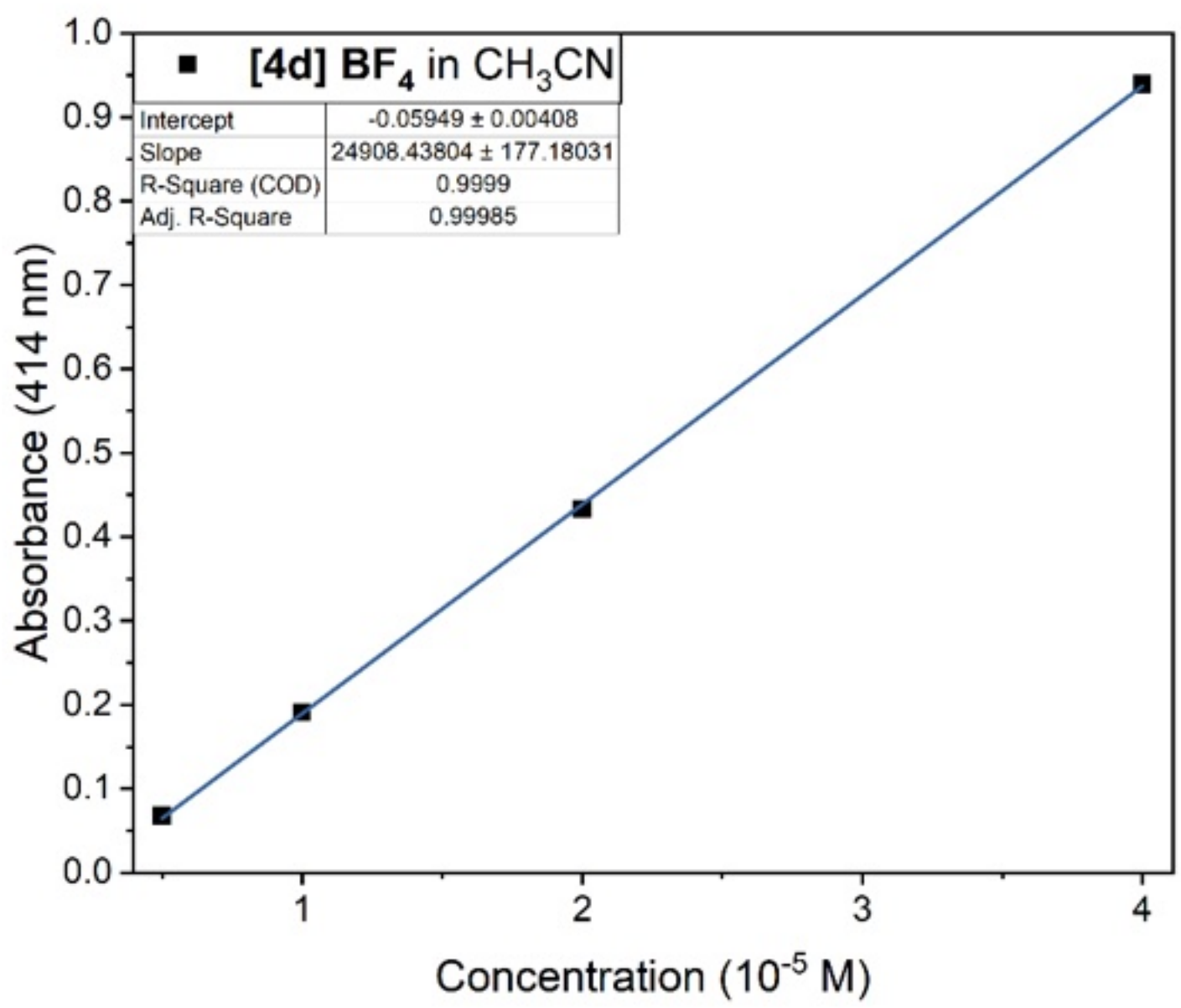

Figure S46. Linear fit of absorbances of **[4d] BF_4** measured at 414 nm in CH_3CN.

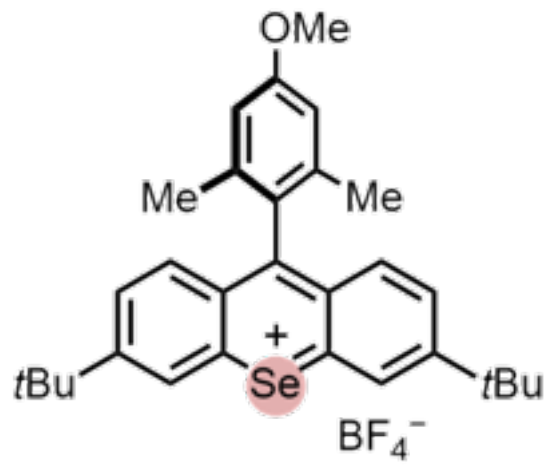

[4d] BF_4

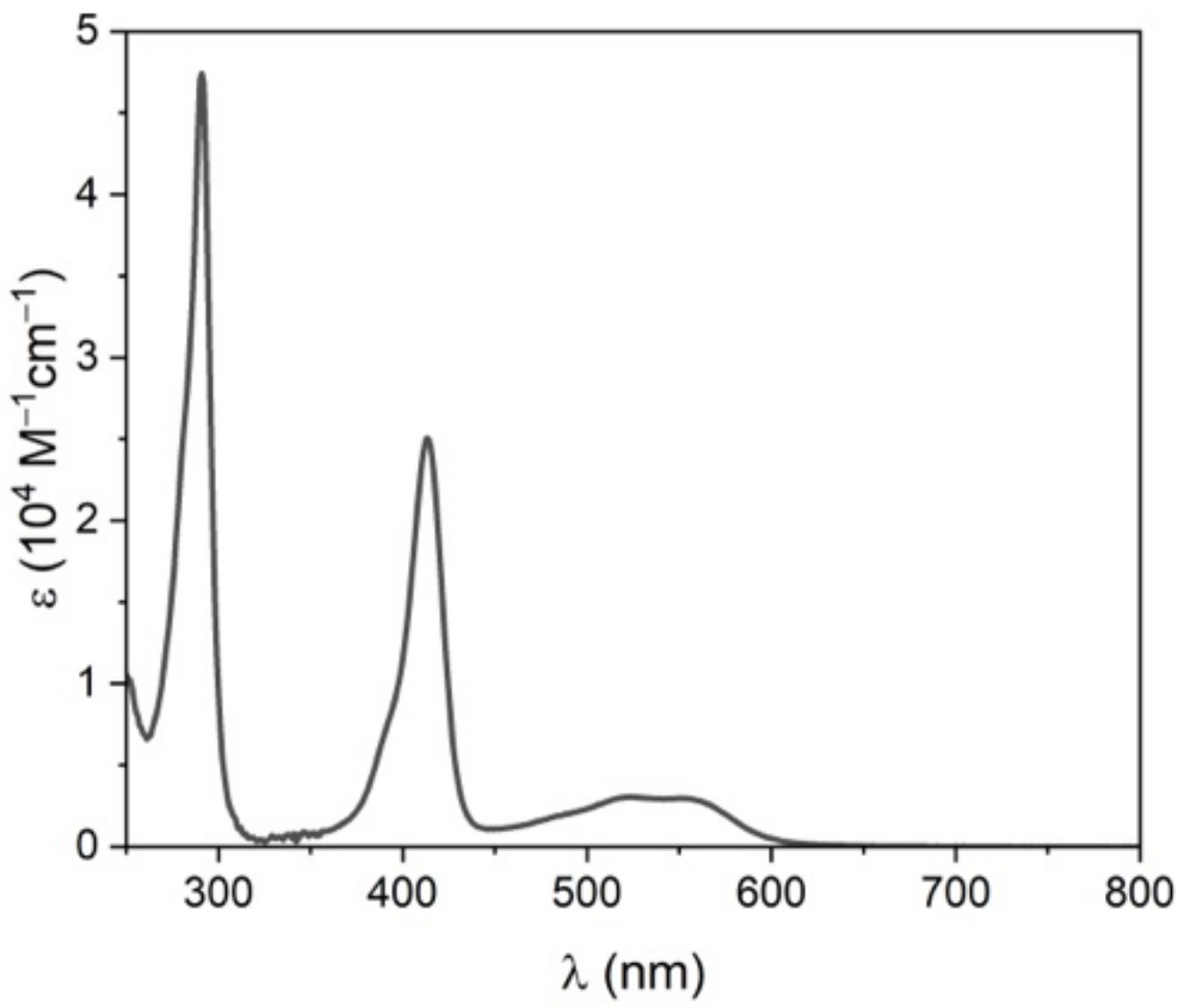

Figure S47. Absorption spectrum of **[4d] BF_4** in CH_3CN.

Summary

ε (414 nm) = 24908 M^{-1} cm^{-1}

$E_{0,0}$ = 2.17 eV

E_T = 1.64 eV

E_{red} (**[4d]**$^+$/**[4d]**$^\cdot$) = −0.07 V vs SCE

$^SE^*_{red}$ (**^{S}[4d]**$^{*+}$/**[4d]**$^\cdot$) = 2.17 + (−0.07 V) = **+2.10 V vs SCE**

$^TE^*_{red}$ (**^{T}[4d]**$^{*+}$/**[4d]**$^\cdot$) = 1.64 + (−0.07 V) = **+1.57 V vs SCE**

SUMMARY OF PHOTOCATALYST ABSORPTION PROPERTIES

Table S10. Absorption properties of the photocatalysts in acetonitrile at 298 K.

	λ / nm	ε / $M^{-1}cm^{-1}$
**[1a] BF_4**	267, 372, 416	78100, 33050, 6500
**[2a] BF_4**	261, 285, 393	28500, 3990, 24600
**[3a] BF_4**	286, 402, 483	36600, 19800, 2470
**[4a] BF_4**	291, 412, 521, 549	31650, 16630, 2030, 1900
**[4b] BF_4**	291, 414, 522, 549	40760, 19500, 2560, 2330
**[4c] BF_4**	291, 414, 520, 549	42560, 19300, 2340, 2150
**[4d] BF_4**	291, 413, 523, 553	47400, 25100, 3050, 2970

Absorption comparison – [4a] BF_4 in polar solvents

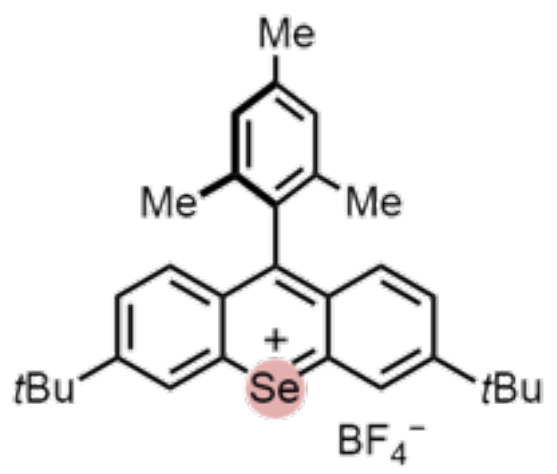

[4a] BF_4

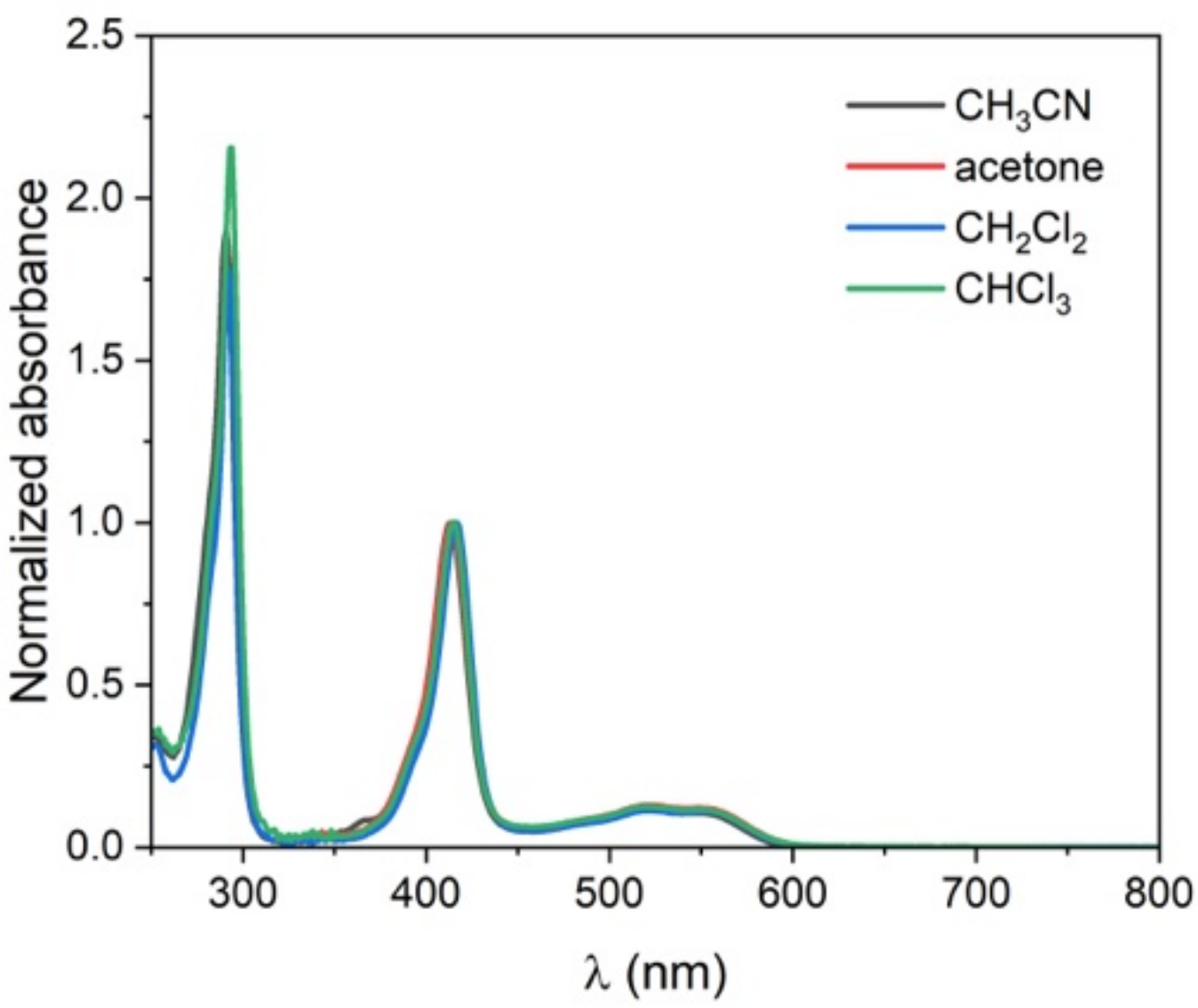

Figure S48. Normalized absorption spectra of **[4a] BF_4** in different solvents.

Absorption comparison – [2a] vs [3a] vs [4a]

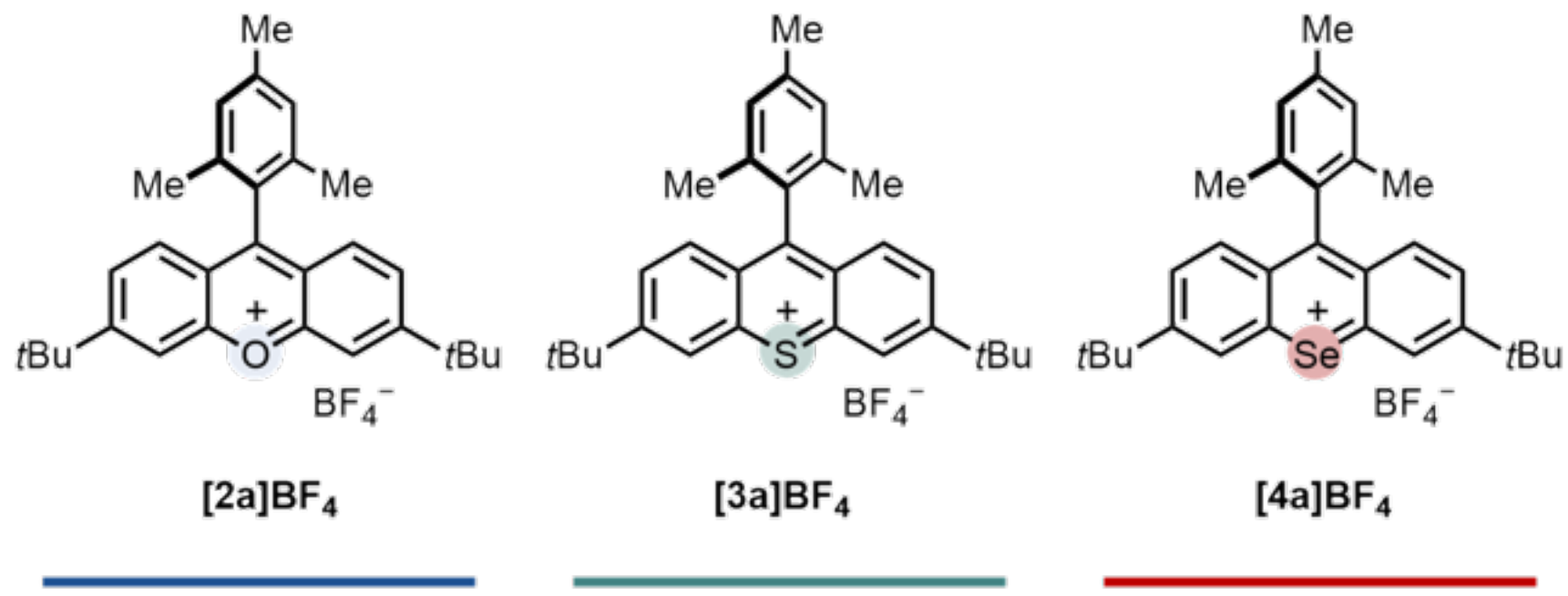

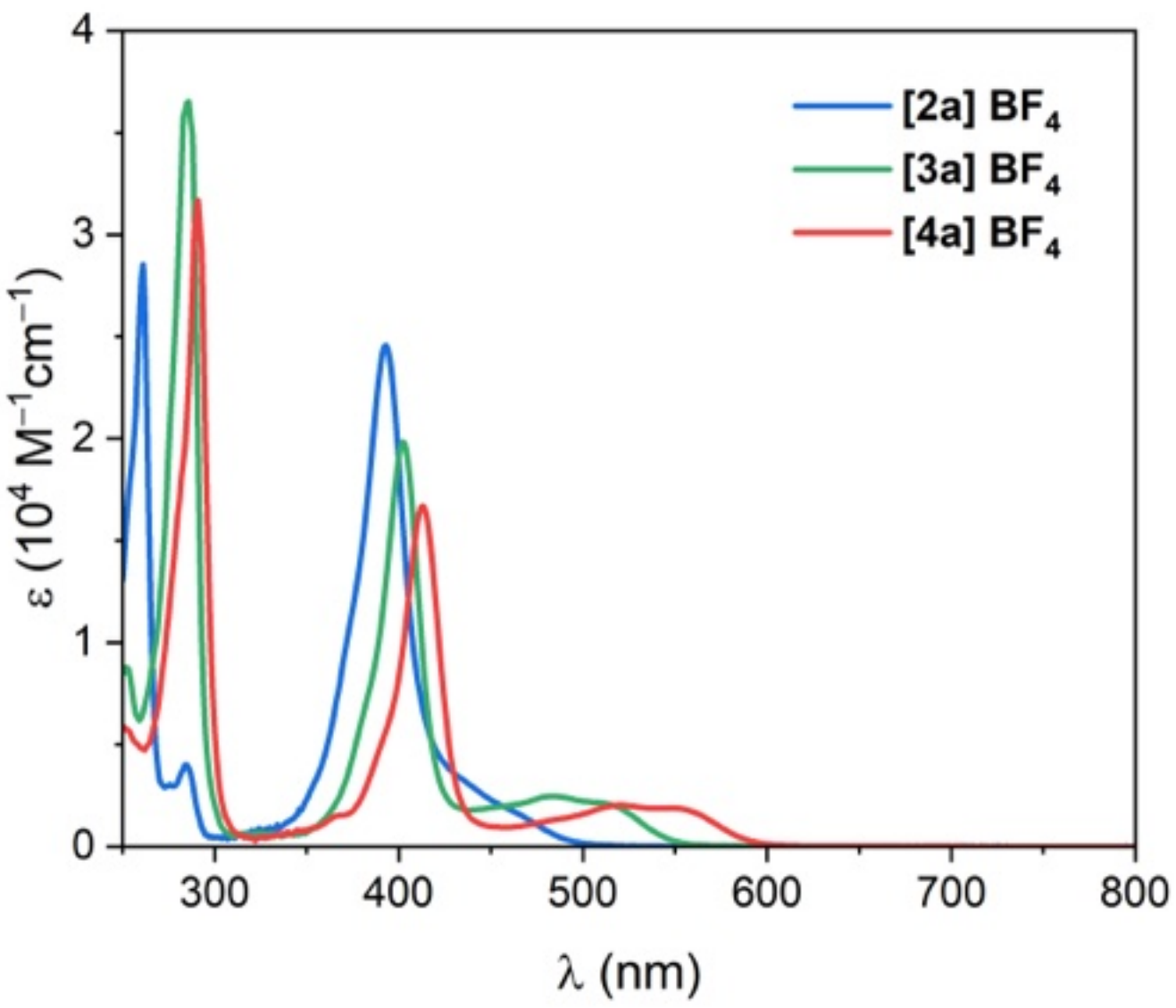

Figure S49. Absorption spectra of **[2a] BF_4** (blue traces), **[3a] BF_4** (green traces), and **[4a] BF_4** (red traces) in CH_3CN.

Absorption comparison – F vs H vs Me vs OMe (i.e., [4a] vs [4b] vs [4c] vs [4d])

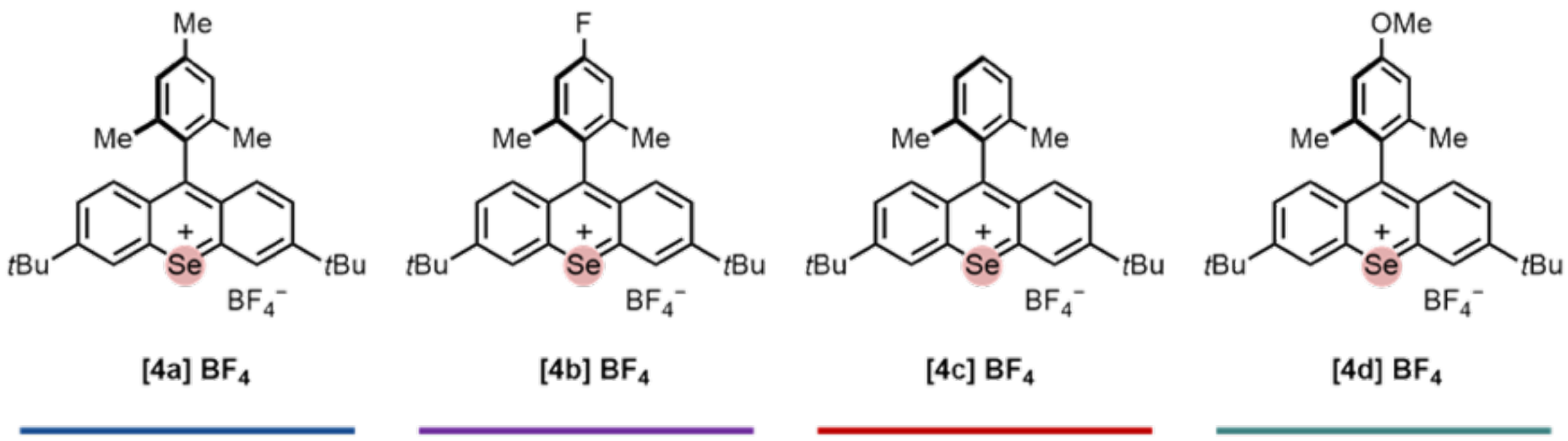

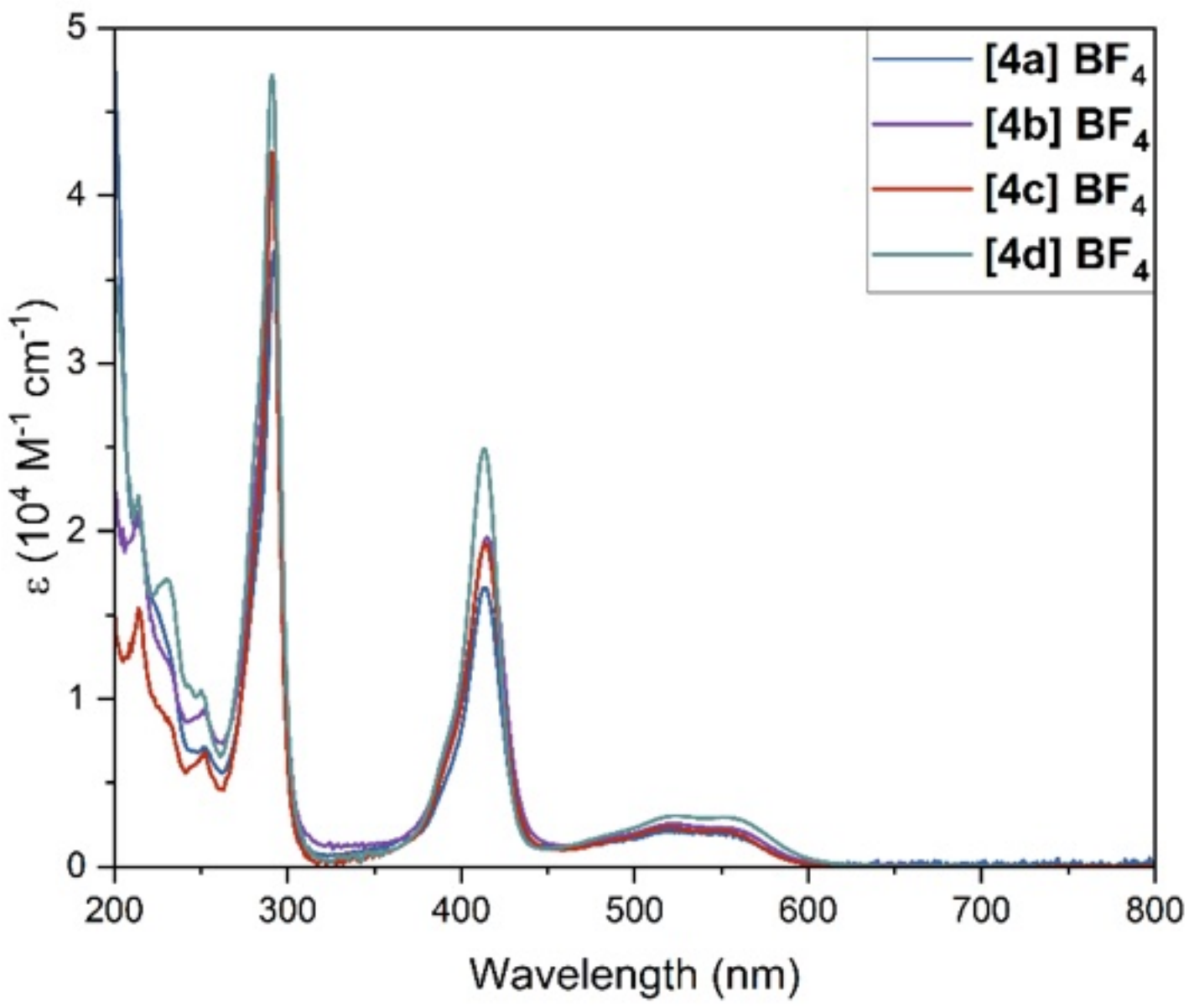

Figure S50. Absorption spectra of **[4a–d] BF₄** normalized to highest wavelength absorption maxima in CH_3CN.

LUMINESCENCE PROPERTIES AT ROOM TEMPERATURE

STATIONARY EMISSION DATA

3,6-Di-*tert*-butyl-9-mesitylacridinium tetrafluoroborate ([1a] BF_4)

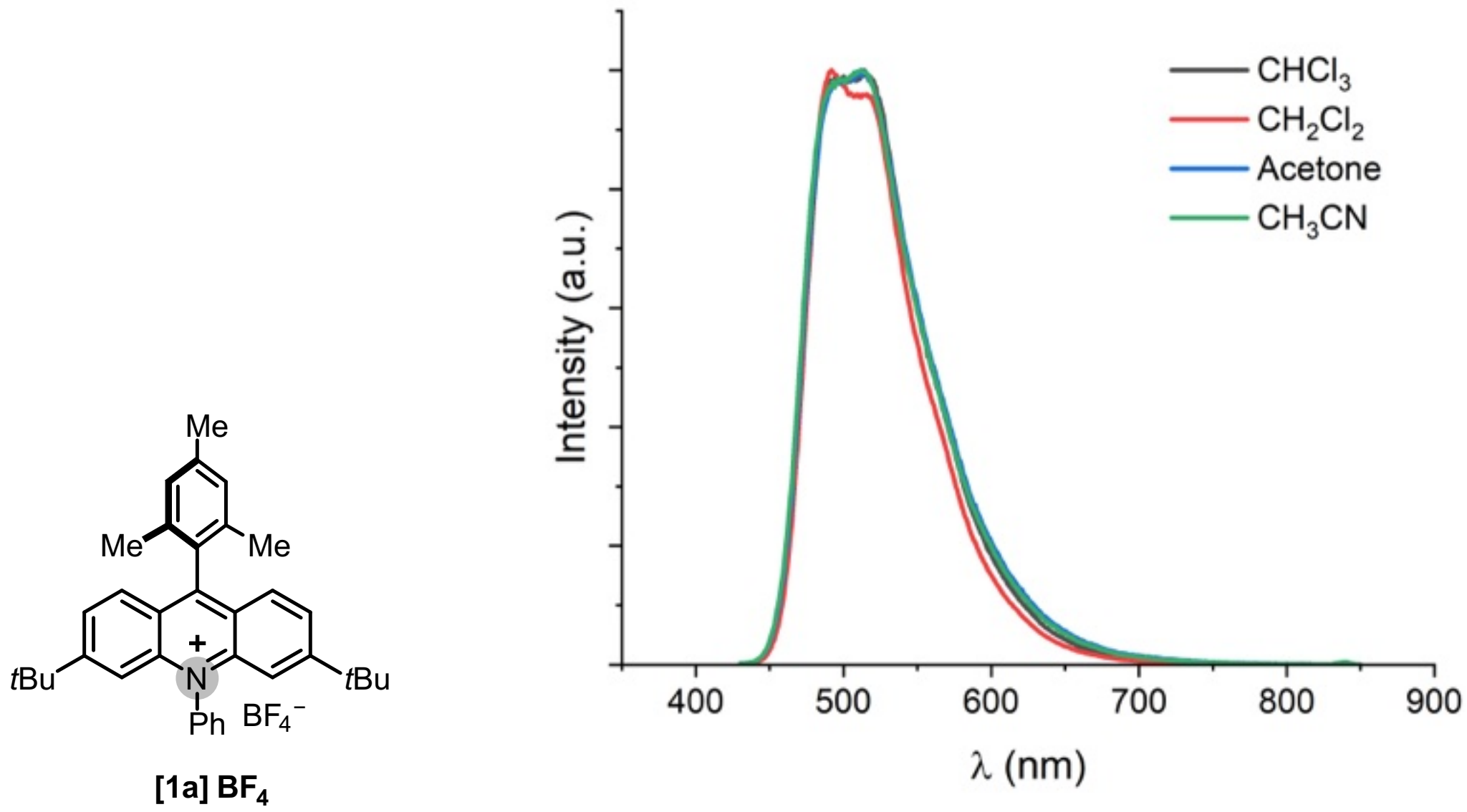

Figure S50. Comparison of the emission spectra (λ_{exc}= 420 nm) of **[1a] BF_4** in different solvents.

3,6-Di-*tert*-butyl-9-mesitylxanthylium tetrafluoroborate ([2a] BF_4)

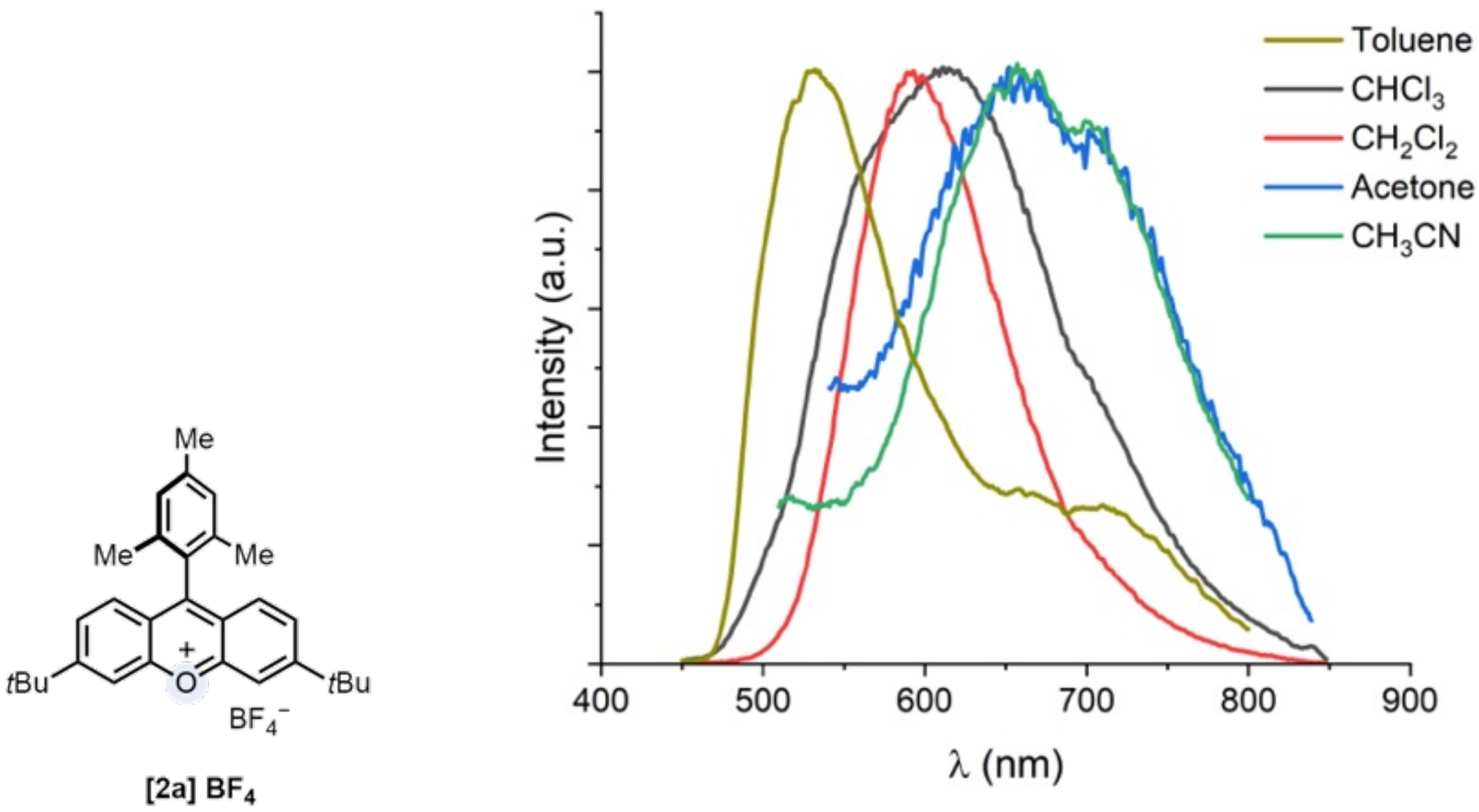

Figure S51. Comparison of the emission spectra (λ_{exc}= 430 nm) of **[2a] BF_4** in different solvents.

3,6-Di-*tert*-butyl-9-mesitylthioxanthylium tetrafluoroborate ([3a] BF_4)

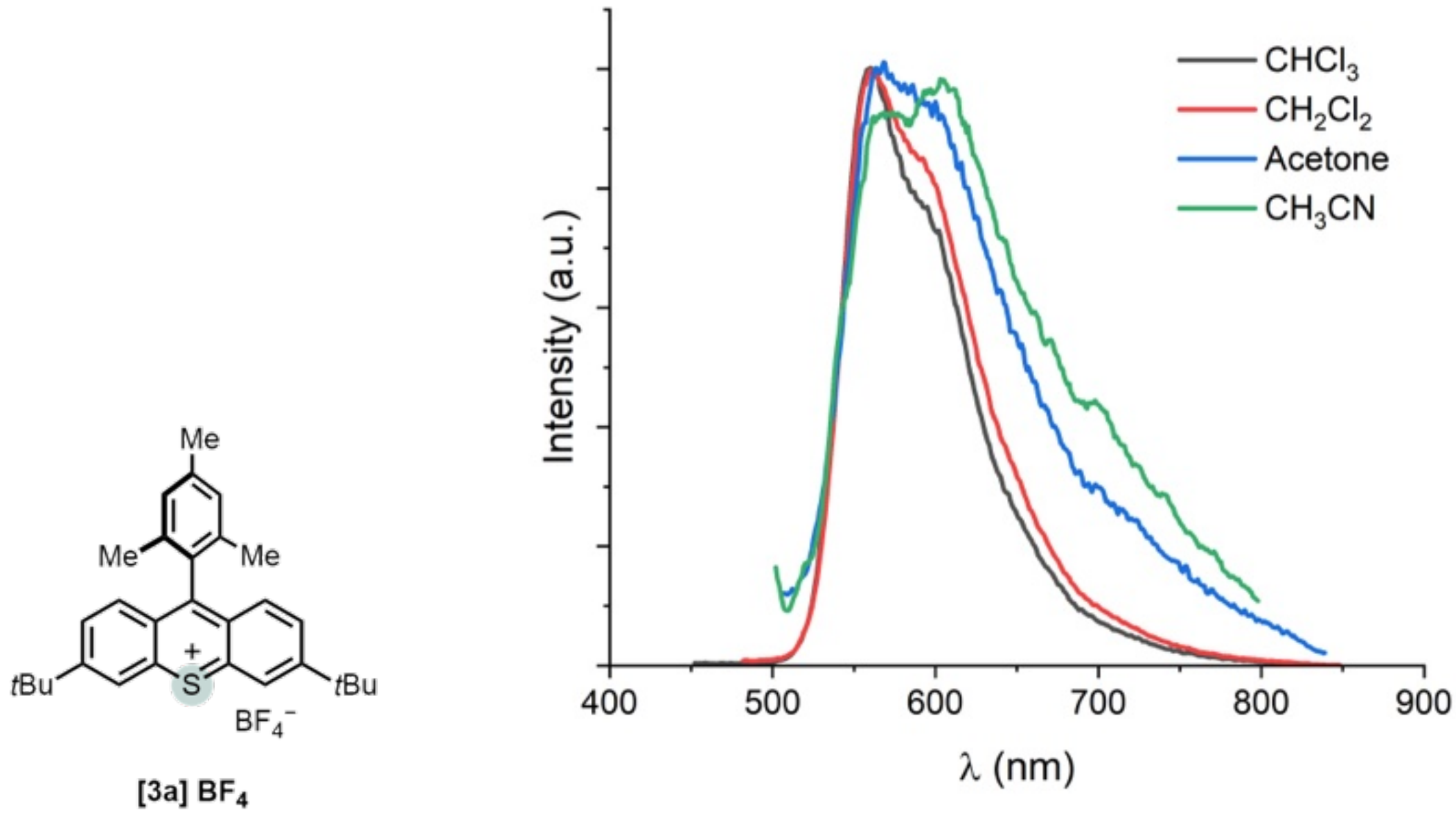

Figure S52. Comparison of the emission spectra (λ_{exc}= 430 nm) of **[3a] BF_4** in different solvents.

3,6-Di-*tert*-butyl-9-mesitylselenoxanthylium tetrafluoroborate ([4a] BF_4)

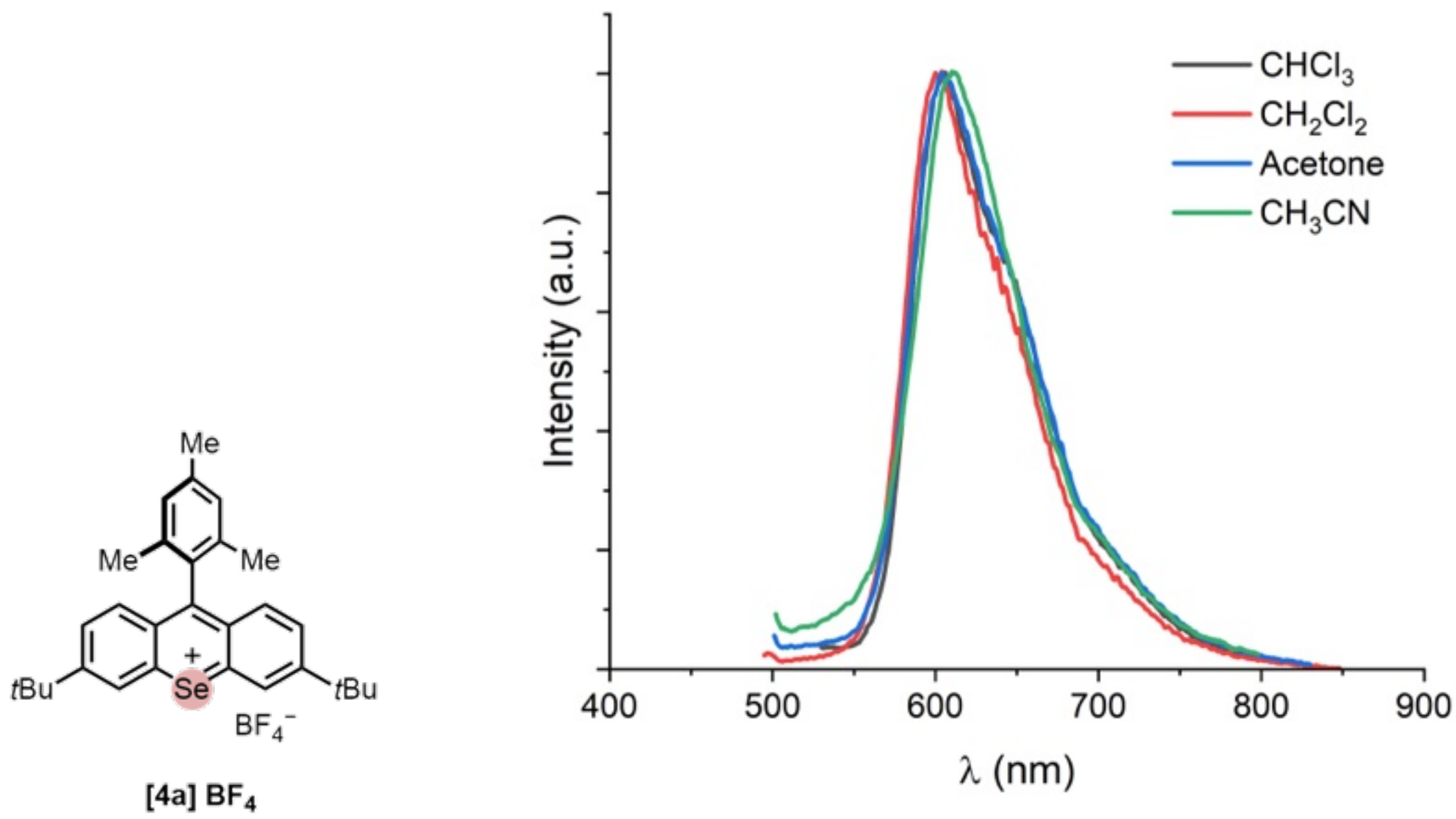

Figure S53. Comparison of the emission spectra (λ_{exc}= 440 nm) of **[4a] BF_4** in different solvents.

3,6-Di-*tert*-butyl-9-(4-fluoro-2,6-dimethylphenyl)selenoxanthylium tetrafluoroborate ([4b] BF_4)

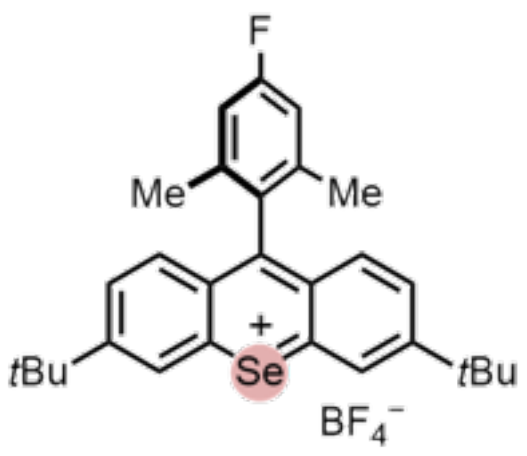

[4b] BF_4

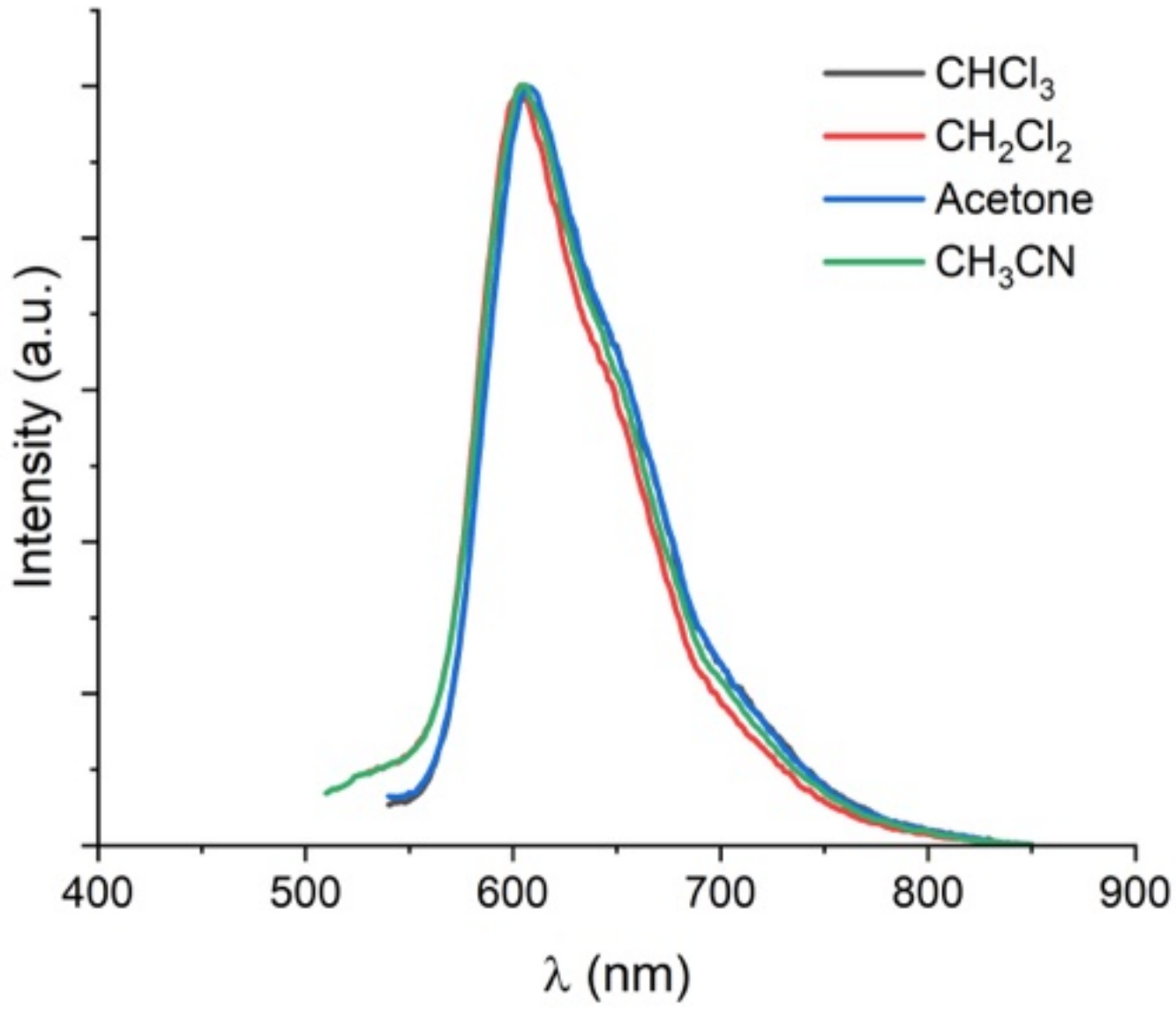

Figure S54. Comparison of the emission spectra (λ_{exc}= 440 nm) of **[4b] BF_4** in different solvents.

3,6-Di-*tert*-butyl-9-(2,6-dimethylphenyl)selenoxanthylium tetrafluoroborate ([4c] BF_4)

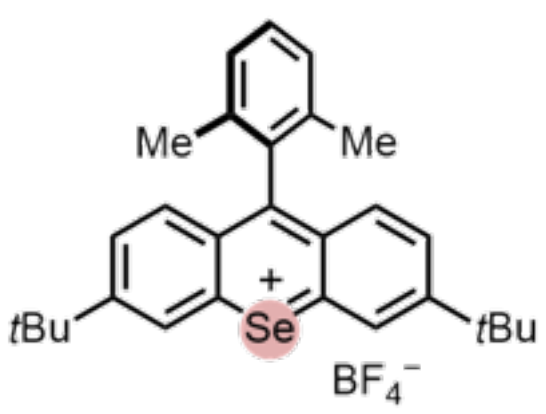

[4c] BF_4

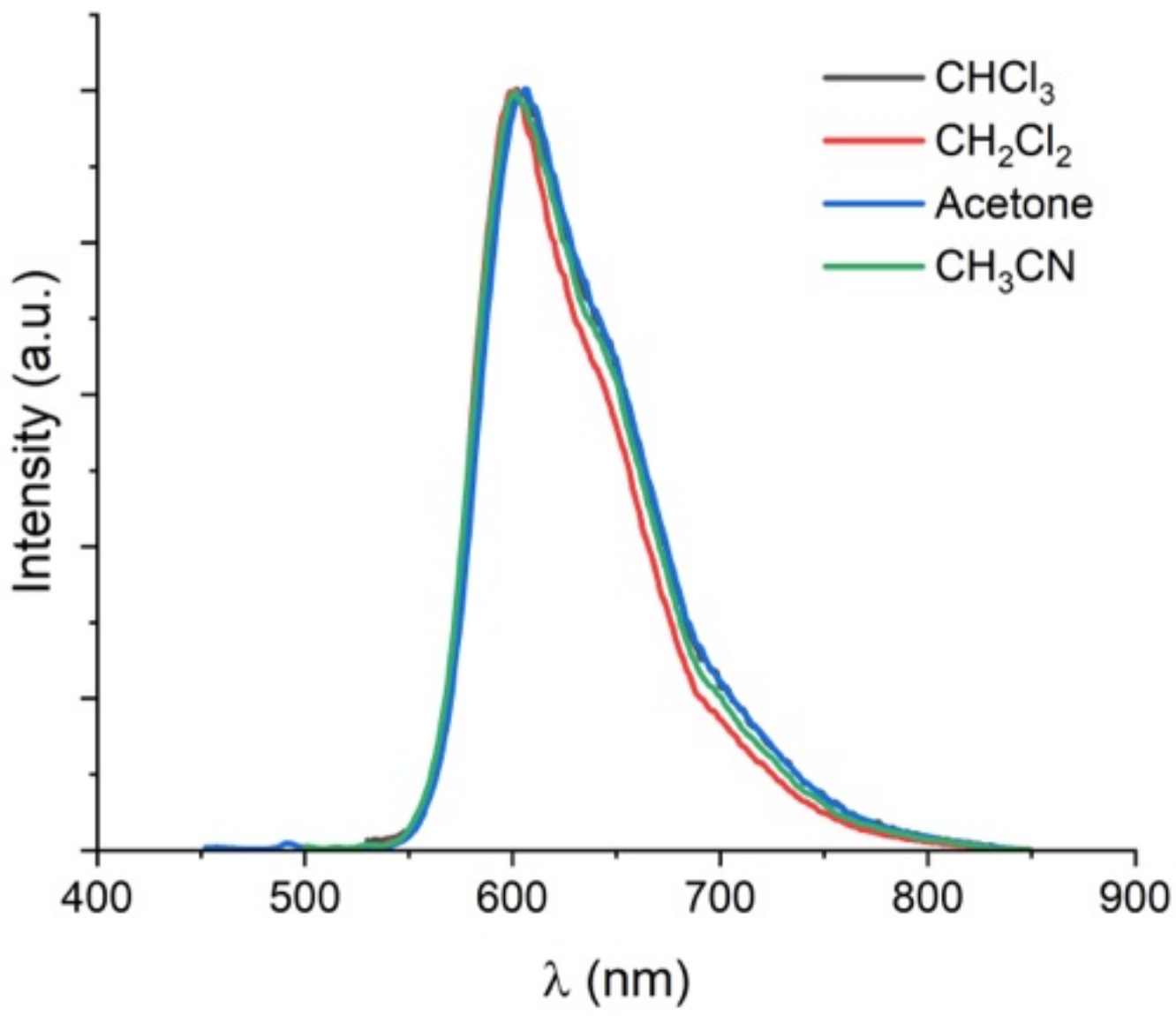

Figure S55. Comparison of the emission spectra (λ_{exc}= 440 nm) of **[4c] BF_4** in different solvents.

3,6-Di-*tert*-butyl-9-(4-methoxy-2,6-dimethylphenyl)selenoxanthylium tetrafluoroborate ([4d] BF_4)

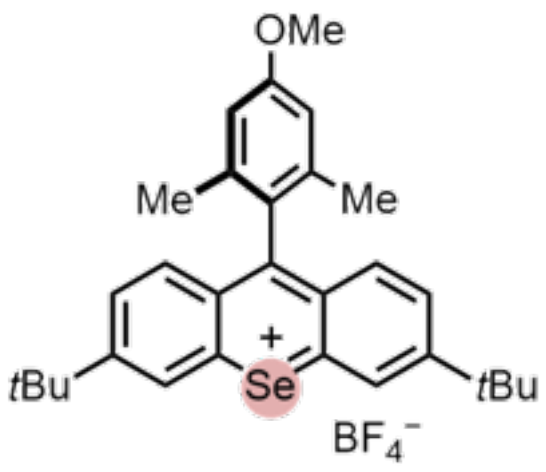

[4d] BF_4

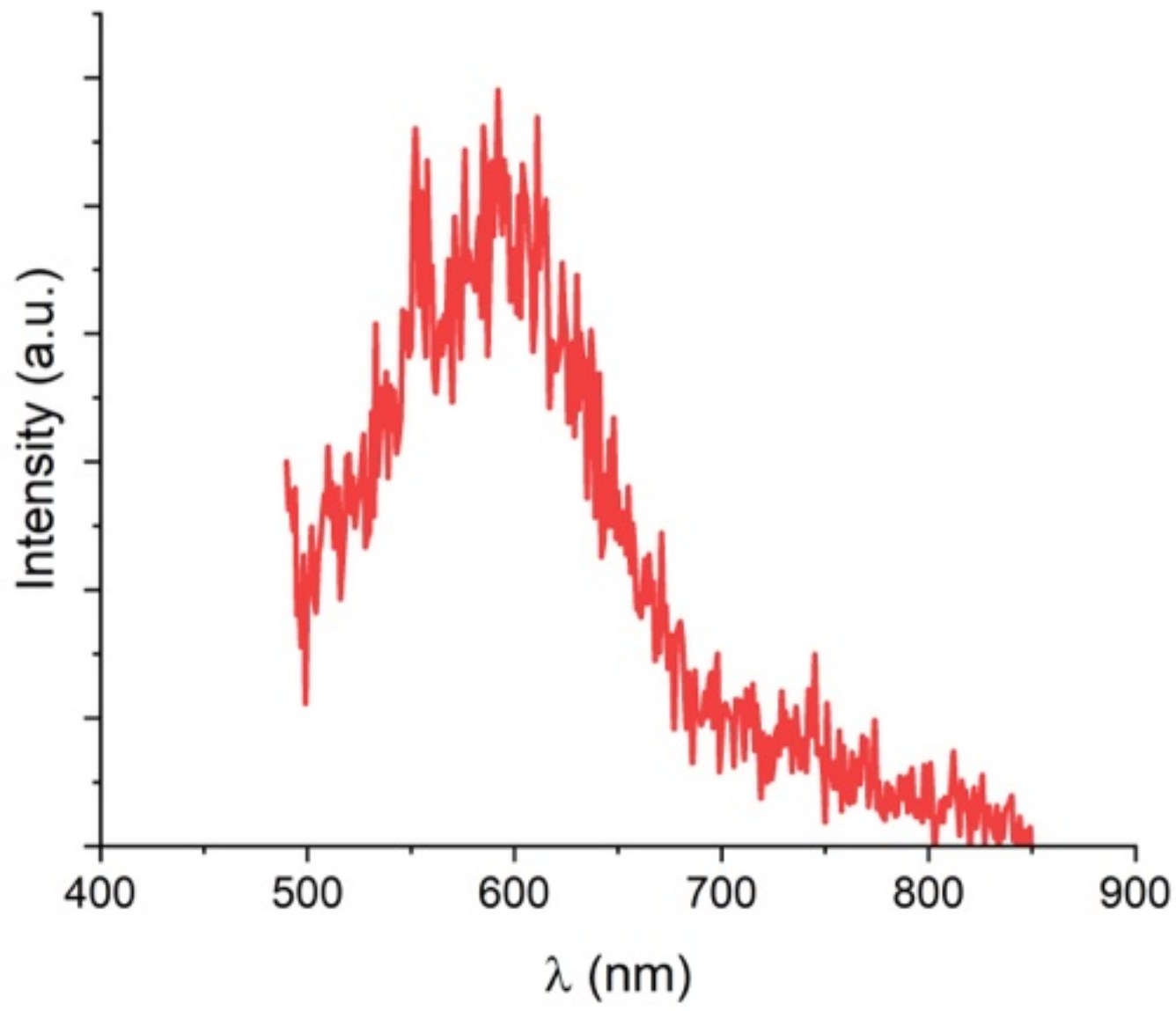

Figure S56. Emission spectrum (λ_{exc}= 440 nm) of **[4d] BF_4** in CH_2Cl_2.

TIME-RESOLVED EMISSION DATA

3,6-Di-*tert*-butyl-9-mesitylacridinium tetrafluoroborate ([1a] BF_4)

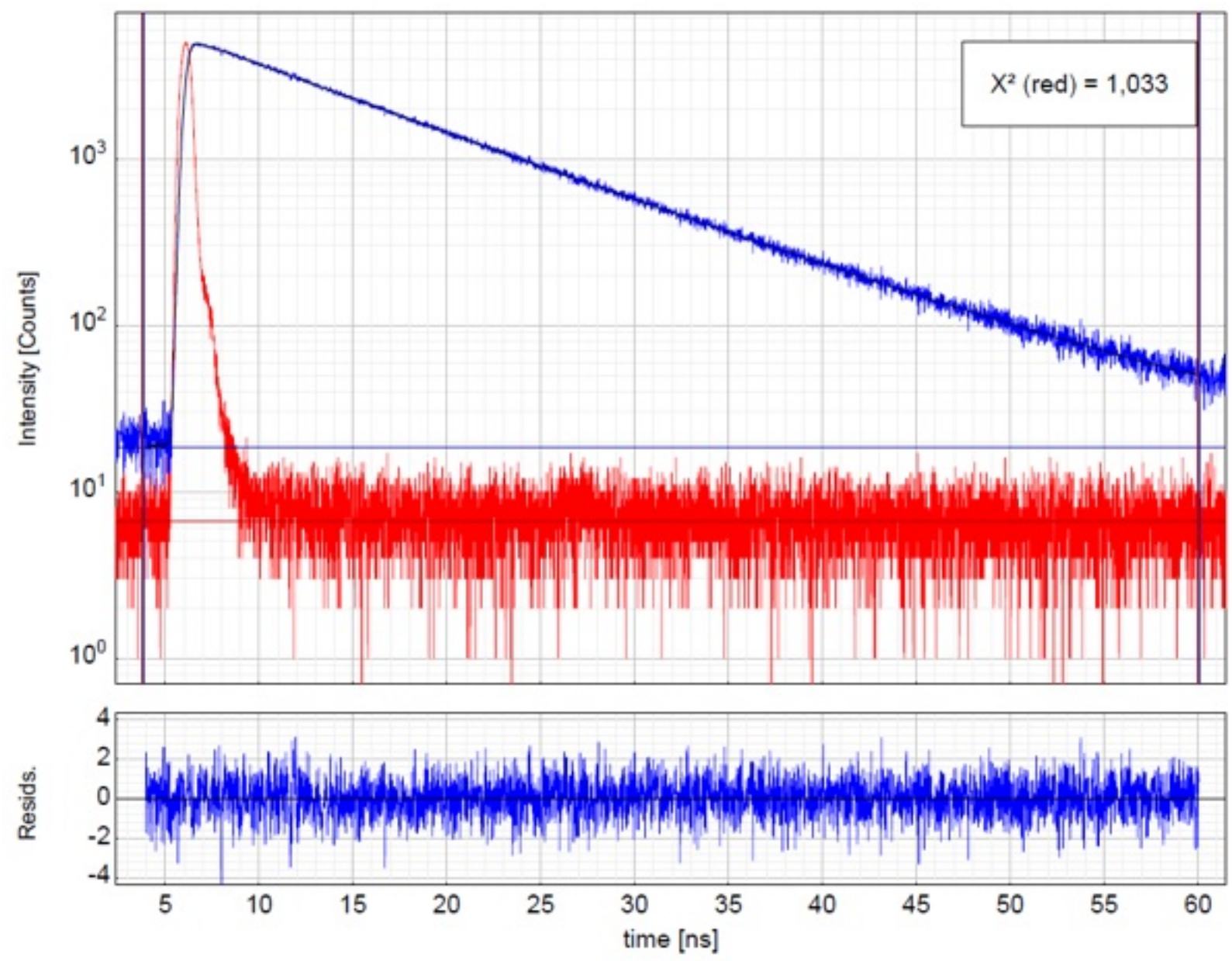

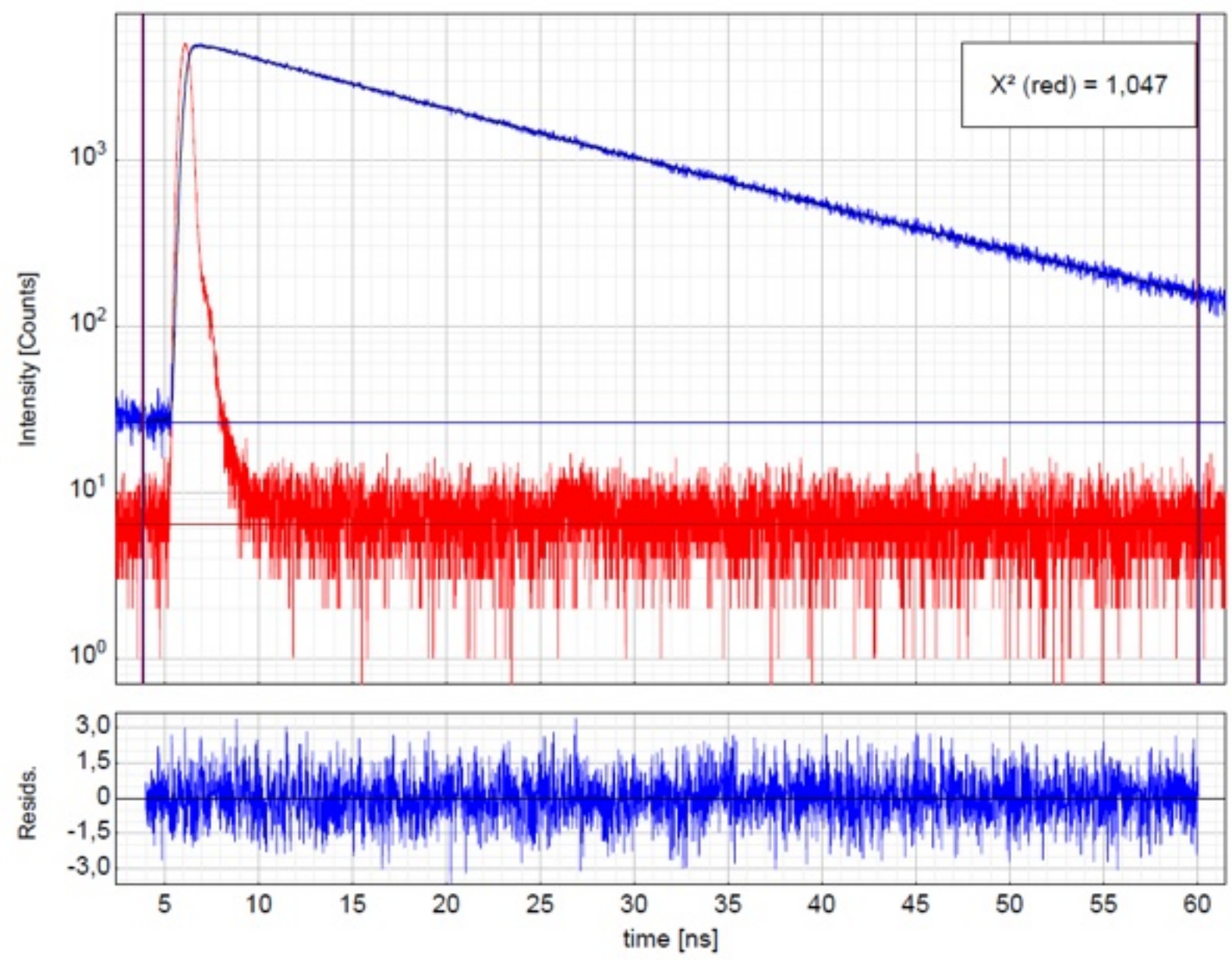

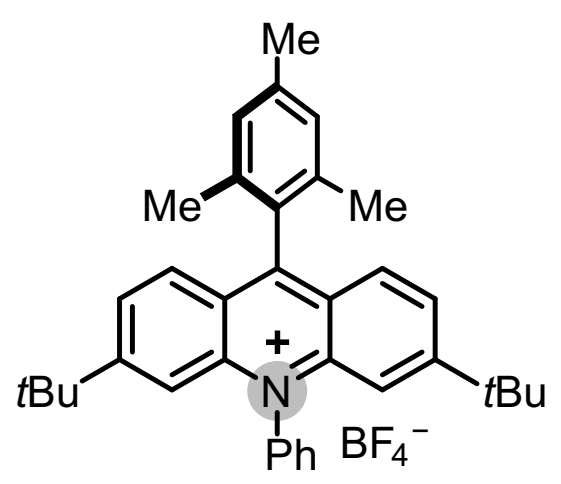

[1a] BF_4

Figure S57. Fluorescence decay of **[1a] BF_4** in CH_3CN (top) and CH_2Cl_2 (bottom) measured by TCSPC: lifetimes of 10.42 ns and 14.34 ns were determined by deconvolution and fitting in CH_3CN and CH_2Cl_2, respectively.

3,6-Di-*tert*-butyl-9-mesitylxanthylium tetrafluoroborate ([2a] BF_4)

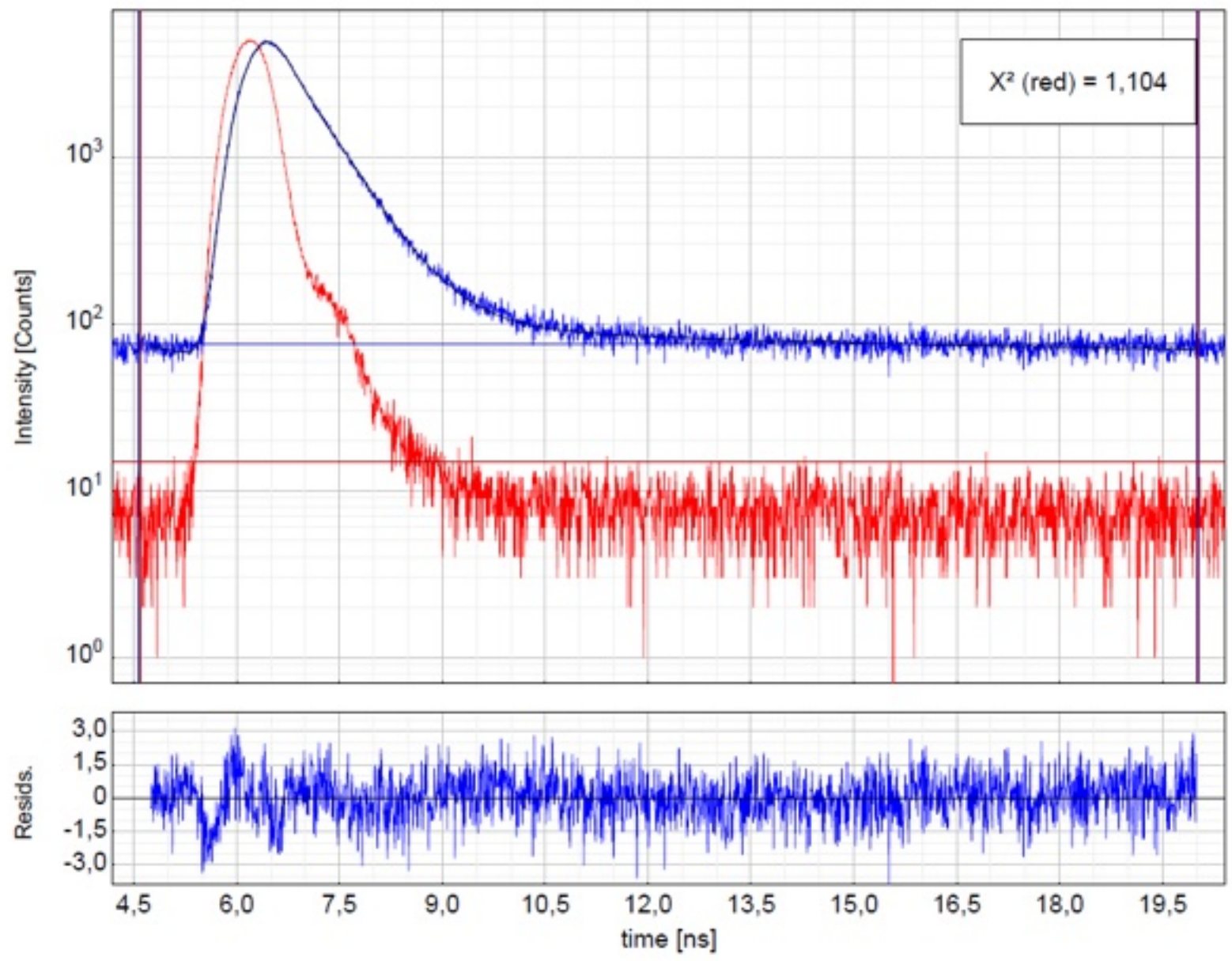

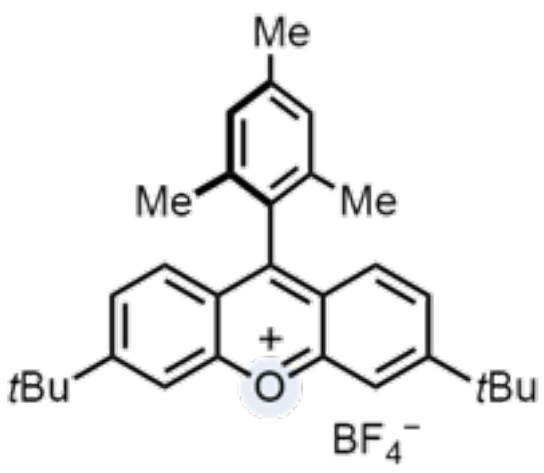

[2a] BF_4

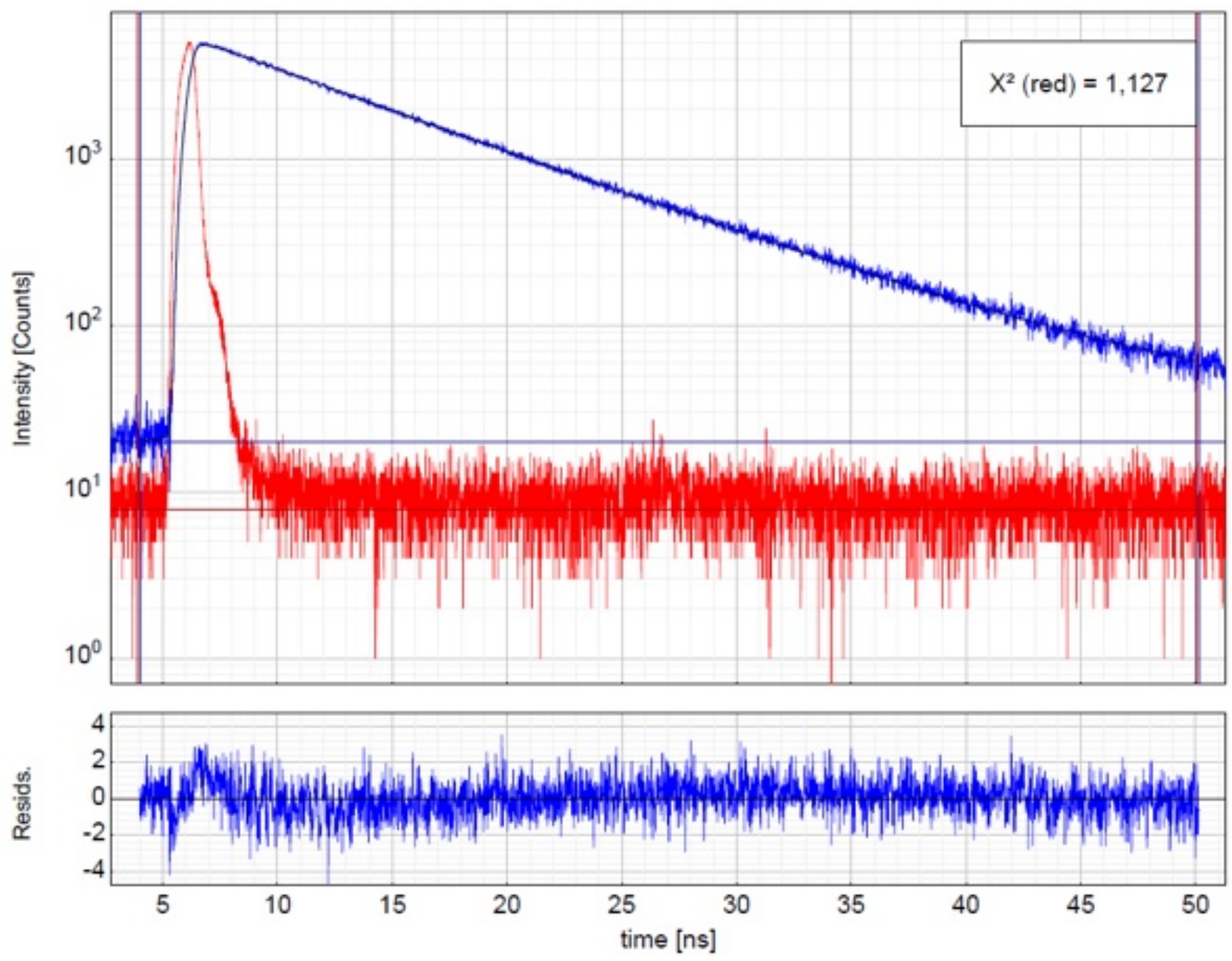

Figure S58. Fluorescence decay of **[2a] BF_4** in CH_3CN (top) and CH_2Cl_2 (bottom) measured by TCSPC: lifetimes of 0.56 ns and 8.56 ns were determined by deconvolution and fitting in CH_3CN and CH_2Cl_2, respectively.

3,6-Di-*tert*-butyl-9-mesitylthioxanthylium tetrafluoroborate ([3a] BF_4)

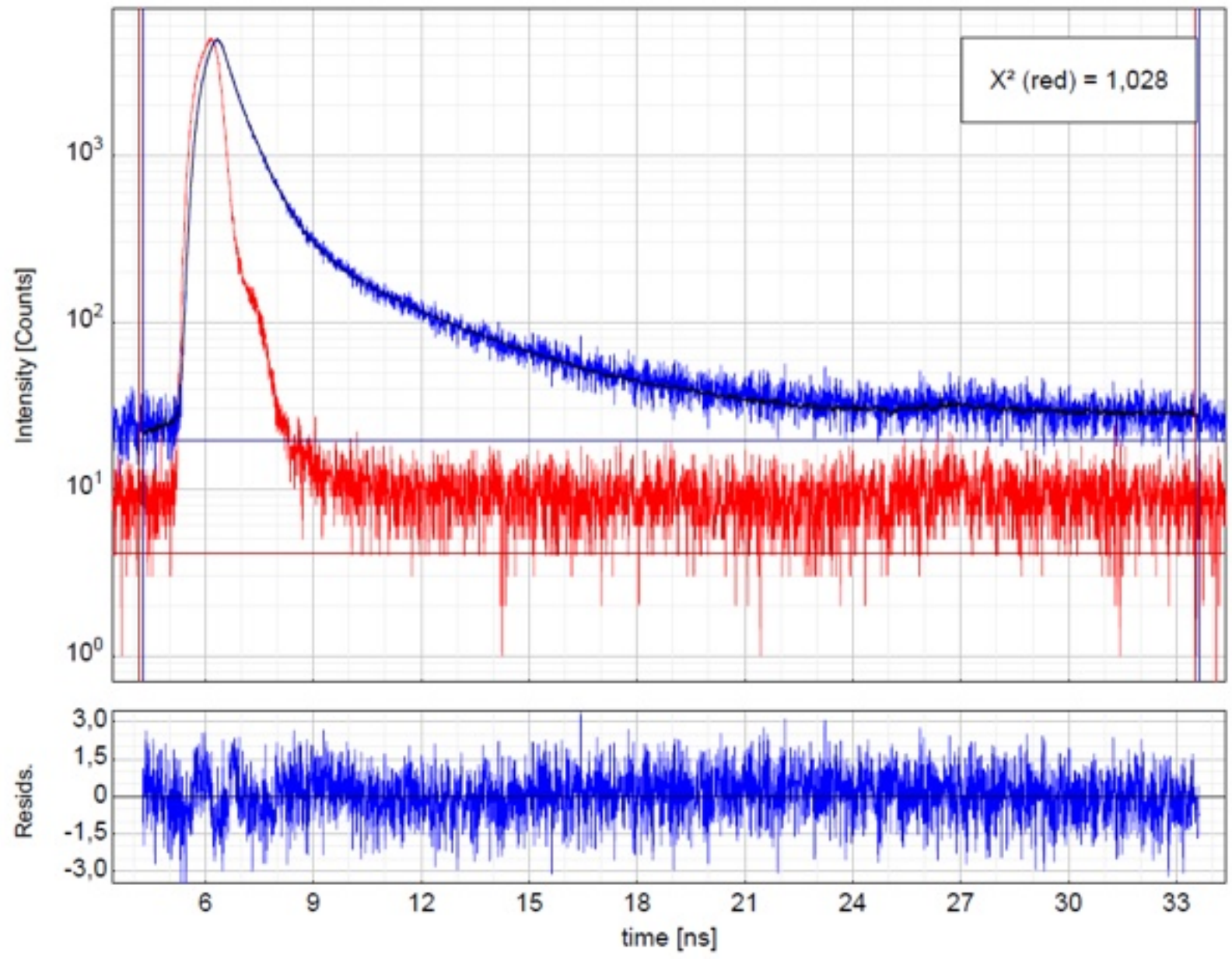

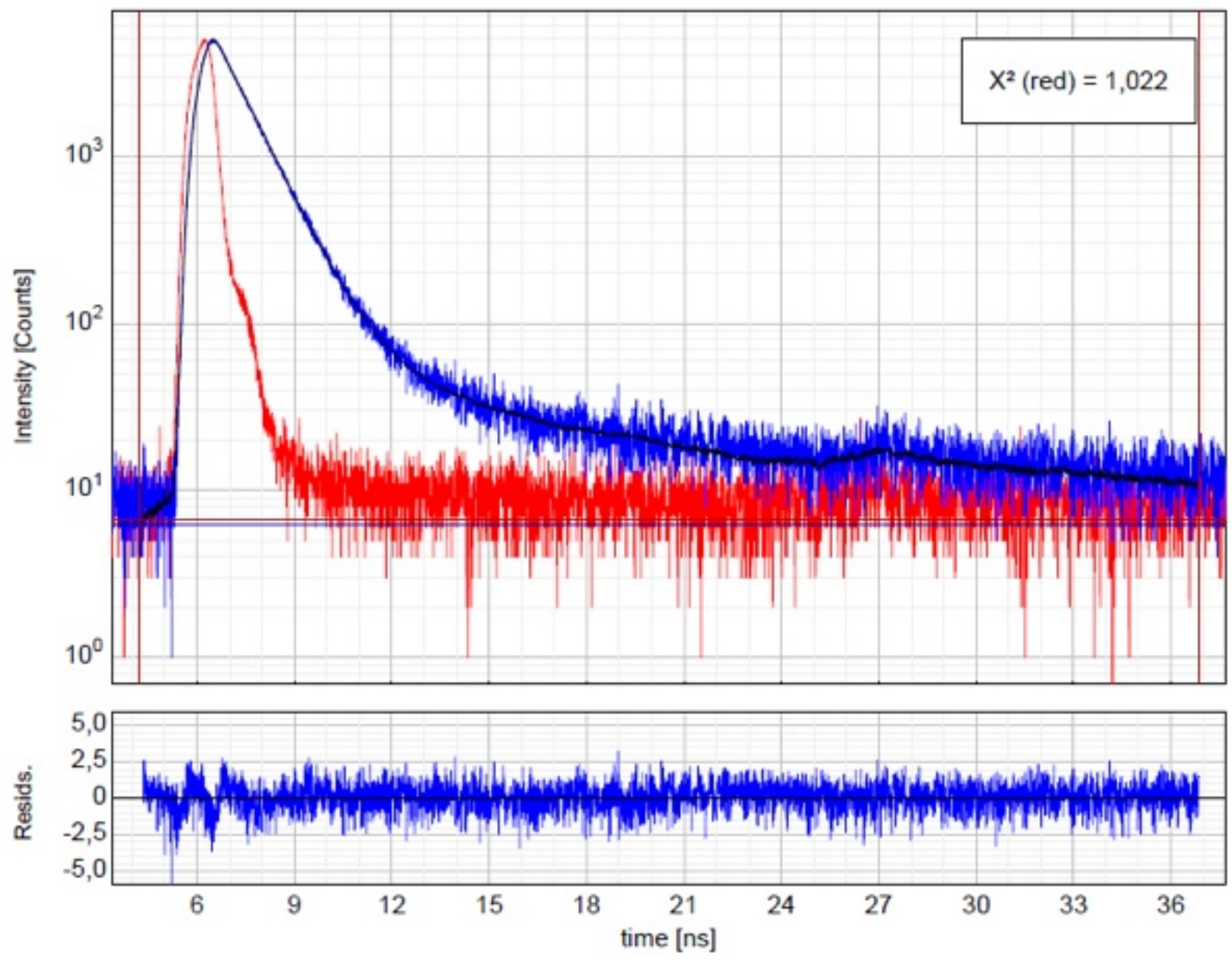

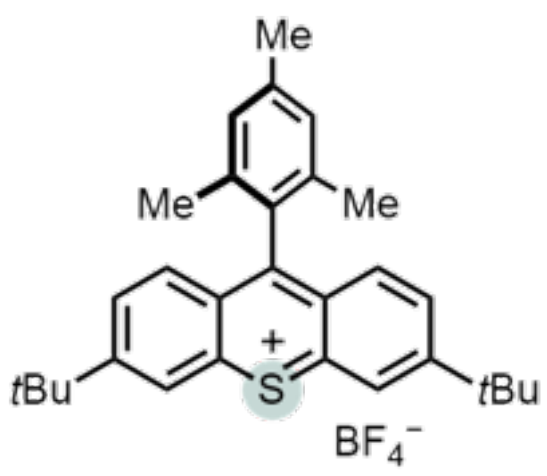

[3a] BF_4

Figure S59. Fluorescence decay of **[3a] BF_4** in CH_3CN (top) and CH_2Cl_2 (bottom) measured by TCSPC: two lifetimes of 0.58 ns (94%), 3.57 ns (6%) and 1.02 ns (99%), 6.69 ns (1%) were determined by deconvolution and fitting in CH_3CN and CH_2Cl_2, respectively.

3,6-Di-*tert*-butyl-9-mesitylselenoxanthylium tetrafluoroborate ([4a] BF_4)

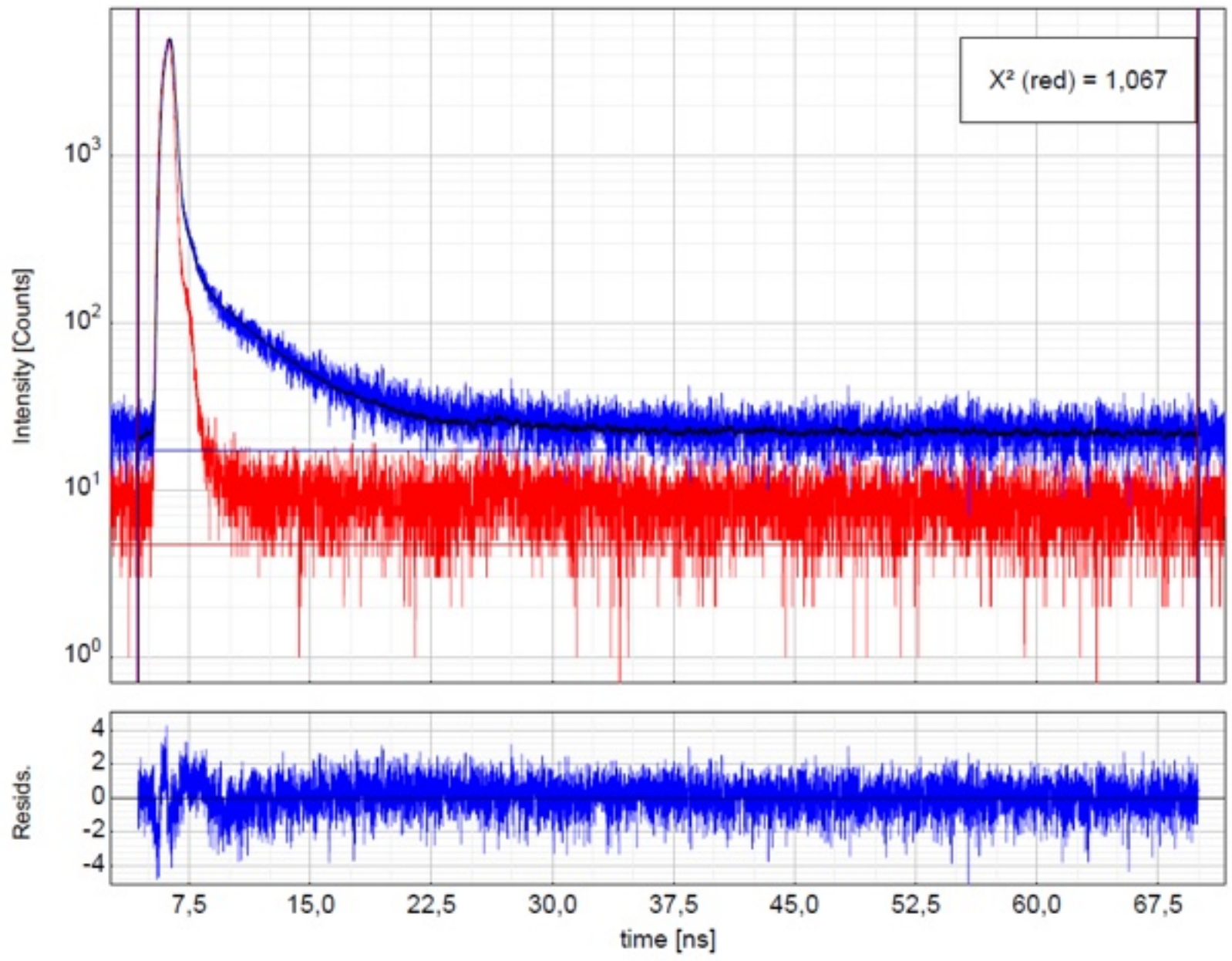

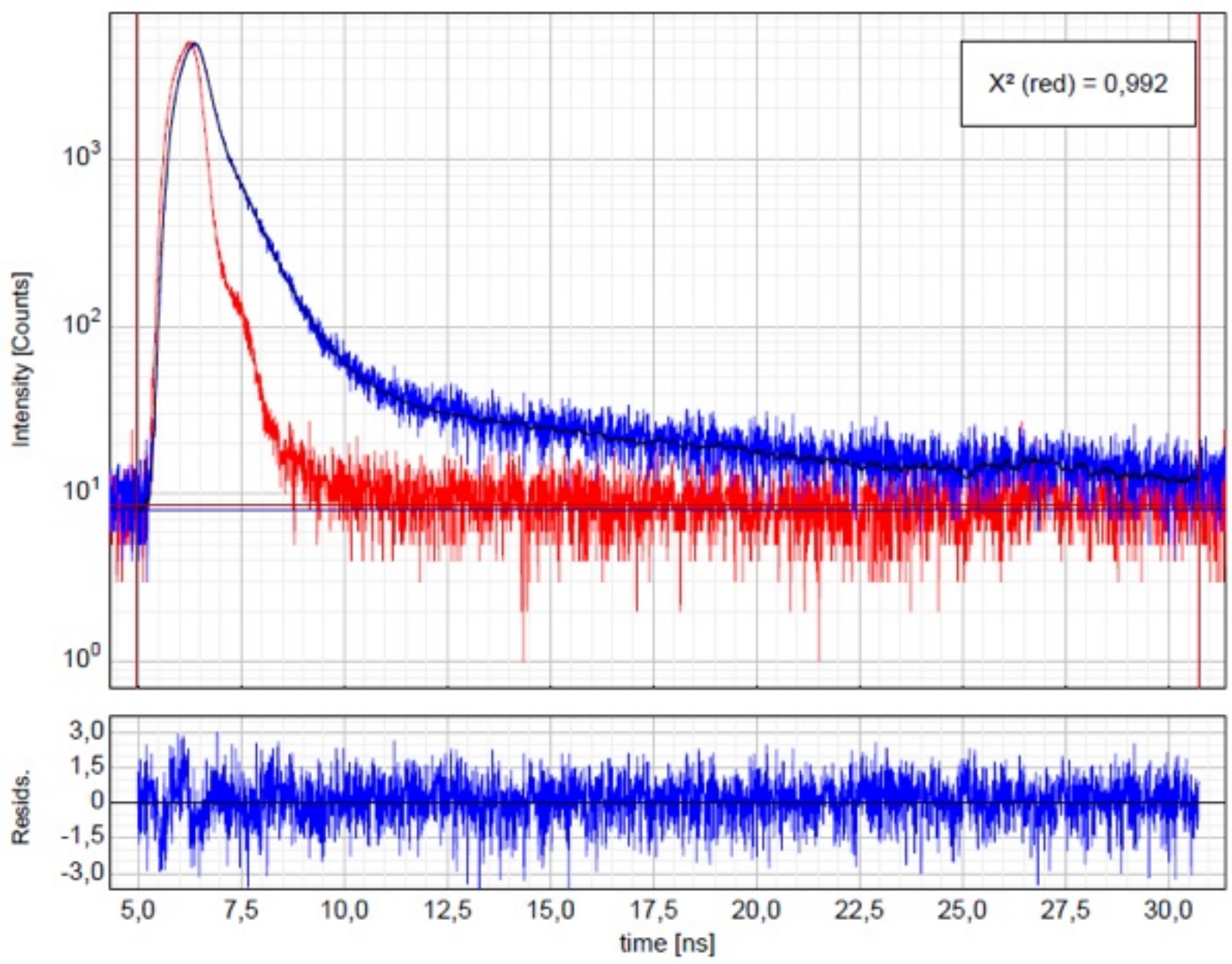

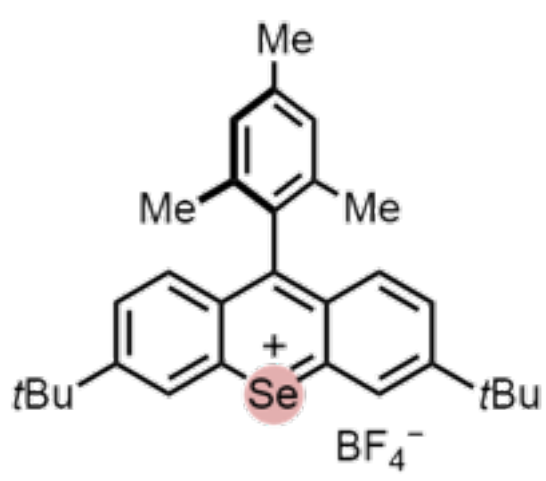

[4a] BF_4

Figure S60. Fluorescence decay of **[4a] BF_4** in CH_3CN (top) and CH_2Cl_2 (bottom) measured by TCSPC: two lifetimes of 0.13 ns (99%), 4.09 ns (1%) and 0.15 ns (87%), 0.77 ns (13%) were determined by deconvolution and fitting in CH_3CN and CH_2Cl_2, respectively (values below 0.2 ns are not quantitatively reliable as they fall below the time-resolution of the TCSPC apparatus).

3,6-Di-*tert*-butyl-9-(4-fluoro-2,6-dimethylphenyl)selenoxanthylium tetrafluoroborate ([4b] BF_4)

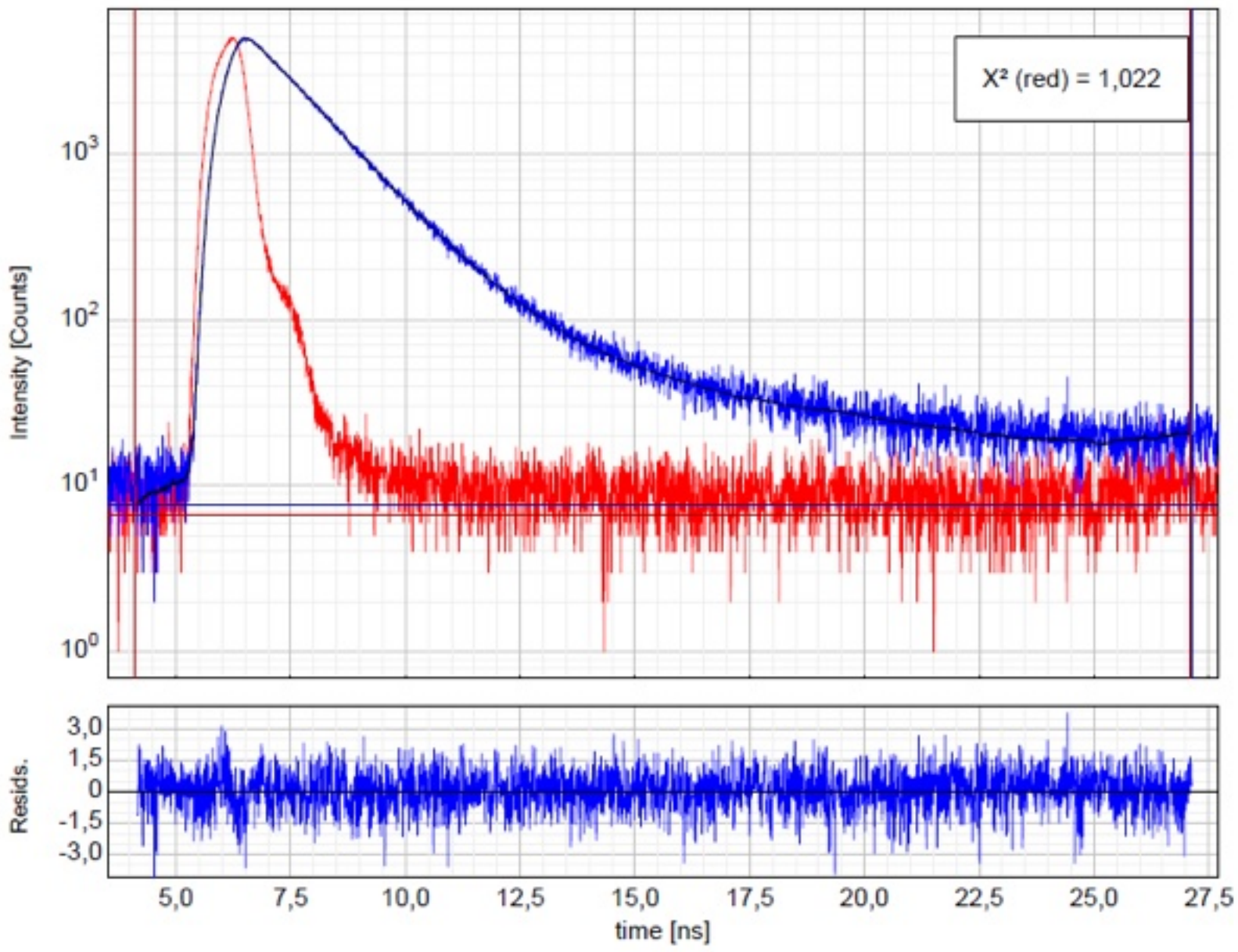

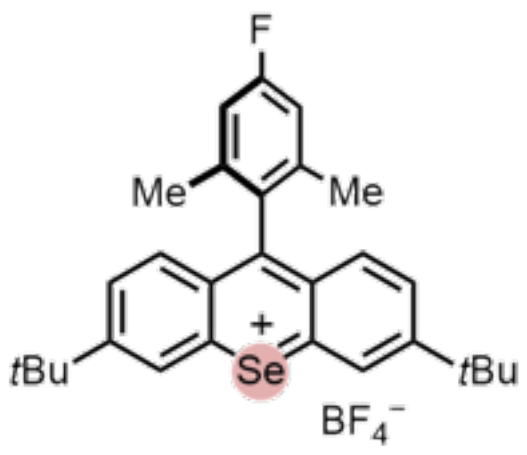

[4b] BF_4

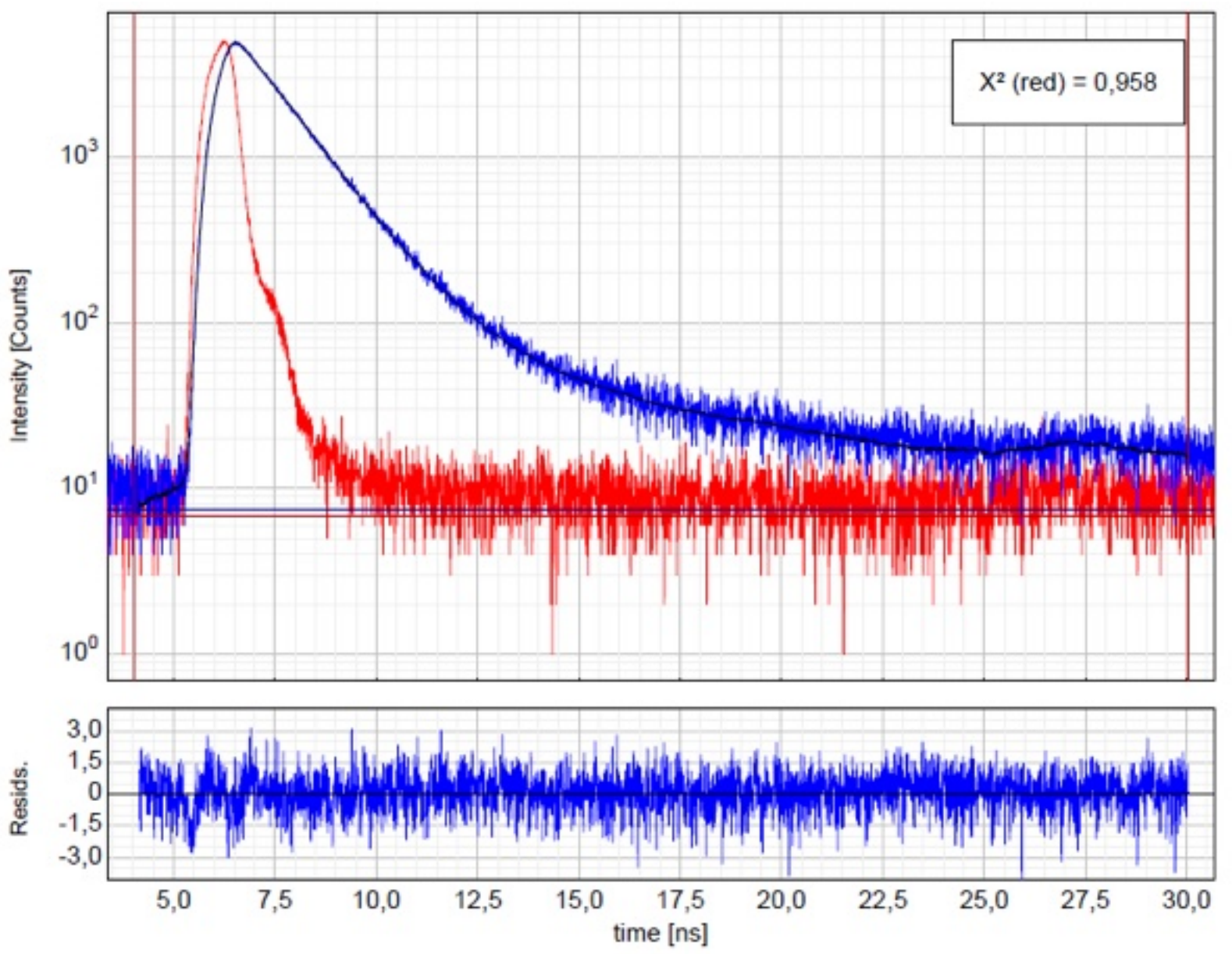

Figure S61. Fluorescence decay of **[4b] BF_4** in CH_3CN (top) and CH_2Cl_2 (bottom) measured by TCSPC: a lifetime of 1.35 ns and 1.25 ns were determined by deconvolution and fitting in CH_3CN and CH_2Cl_2, respectively.

3,6-Di-*tert*-butyl-9-(2,6-dimethylphenyl)selenoxanthylium tetrafluoroborate ([4c] BF_4)

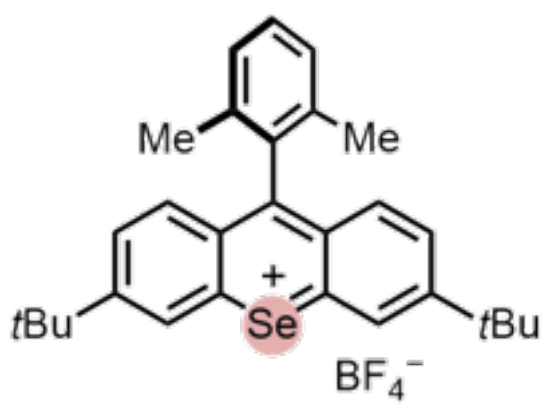

[4c] BF_4

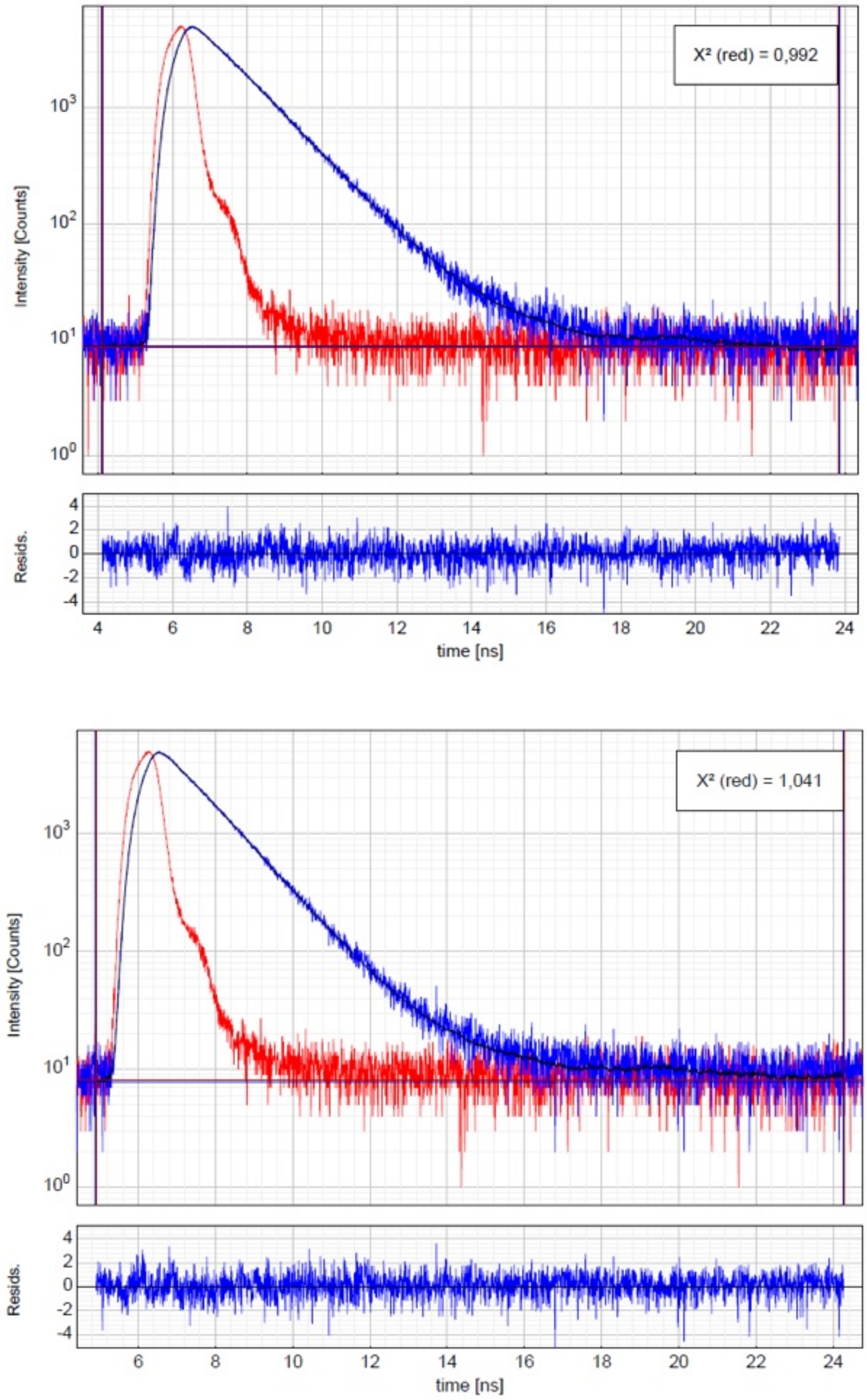

Figure S62. Fluorescence decay of **[4c] BF_4** in CH_3CN (top) and CH_2Cl_2 (bottom) measured by TCSPC: a lifetime of 1.26 ns and 1.16 ns were determined by deconvolution and fitting in CH_3CN and CH_2Cl_2, respectively.

SUMMARY OF PHOTOCATALYST LUMINESCENCE PROPERTIES

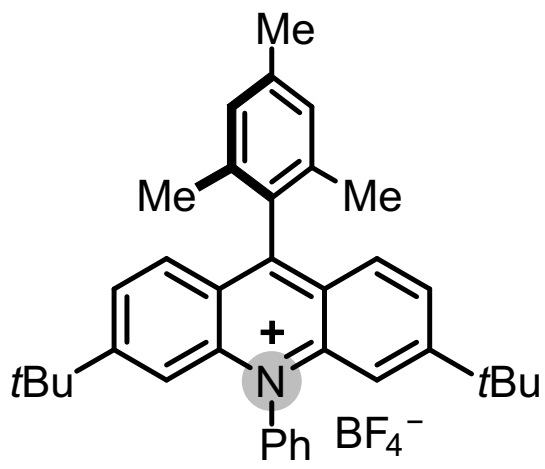

[1a] BF$_4$

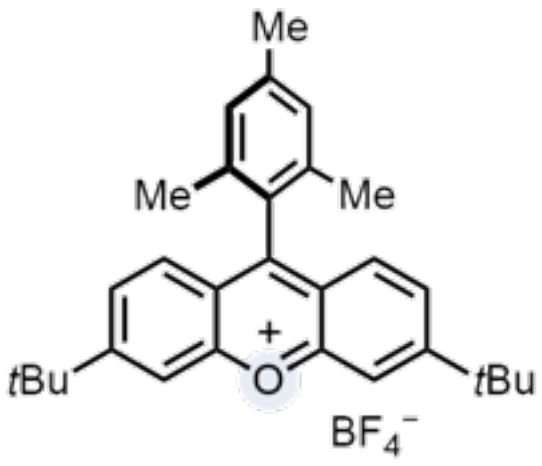

[2a] BF$_4$

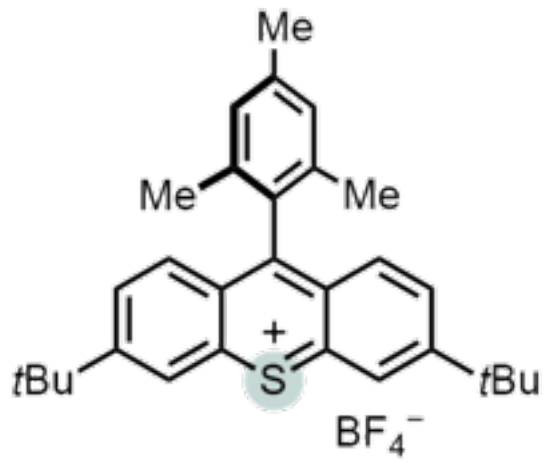

[3a] BF$_4$

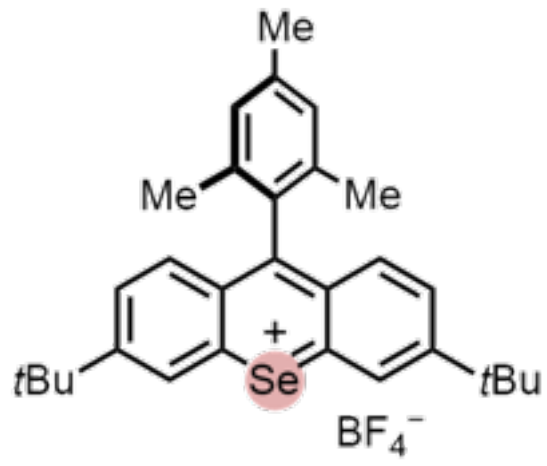

[4a] BF$_4$

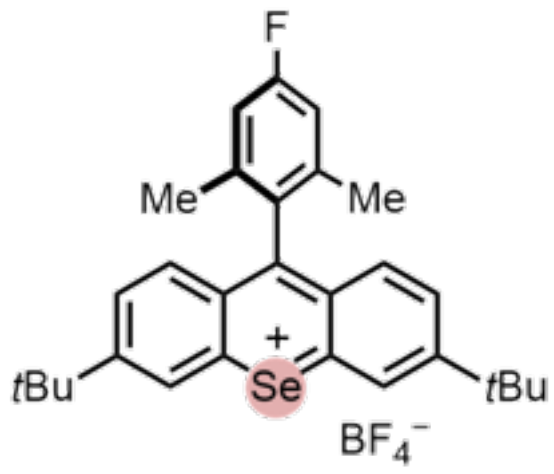

[4b] BF$_4$

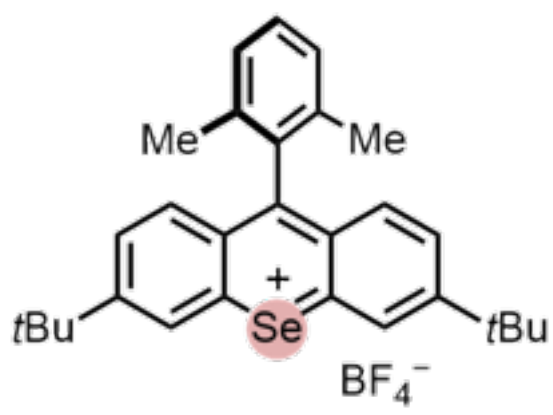

[4c] BF$_4$

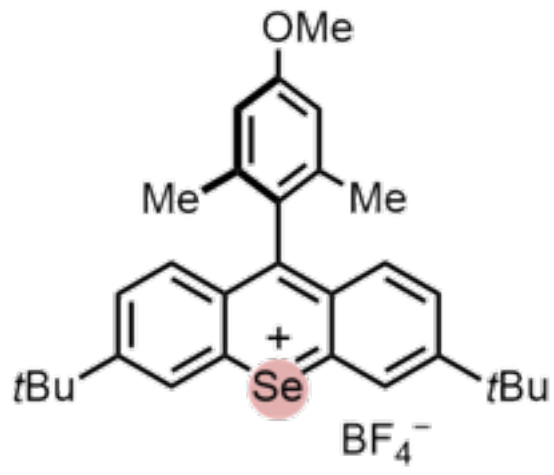

[4d] BF$_4$

Table S11. Summary of the luminescence properties at 298 K of the photocatalysts studied in this work.

	CH_3CN				*CH_2Cl_2*			
	λ / nm	Φ / % [a]	τ_S / ns[b]	k_F / 10^5 s^{-1}	λ / nm	Φ / % [a]	τ_S / ns[b]	k_F / 10^5 s^{-1}
**[1a] BF$_4$**	511	24.1	10.42	231	494	49.8	14.34	347
**[2a] BF$_4$**	660	0.05	0.56	9	591	2.76	8.56	32
**[3a] BF$_4$**	604	0.10	0.58 (94%)<br>3.57 (6%)	17	560	0.93	1.02	91
**[4a] BF$_4$**	609	0.17	<0.2[c]	>85	603	0.28	<0.2[c] (87%)<br>0.77 (13%)	>103
**[4b] BF$_4$**	605	1.23	1.34	92	604	1.40	1.25	112
**[4c] BF$_4$**	601	1.20	1.26	95	601	1.39	1.16	120
**[4d] BF$_4$**	-	-	-	-	599	[d]	[d]	-

[a] Estimated using the relative method with fluorescein in 0.1 M NaOH solution or $[Ru(bpy)_3]^{2+}$ in H_2O as a standard (Φ = 96% and 2.8%, respectively); [b] estimated by TCSPC (excitation at 380 nm for **[1a] BF$_4$** and **[2a] BF$_4$**, at 460 nm for **[3a] BF$_4$** and **[4a-c] BF$_4$**), time components obtained by biexponential fitting with <5% amplitudes were not considered (see **Figures S57-S62**); [c] below the resolution of the TCSPC; [d] too weak emission.

The fluorescence of acridinium **[1a] BF_4** is sharp and appreciable independent of solvent polarity. Large emission yields are accompanied by long excited state lifetime in the order of tens of ns, delivering large radiative constant consistent with an emissive singlet excited state of locally excited (LE) nature.

The fluorescence spectrum of xanthylium **[2a] BF_4** is generally structureless and exhibits a pronounced dependence on solvent polarity. In polar solvents such as acetonitrile and acetone, the emission is broad (FWHM of ca 0.56 eV) and significantly red-shifted, whereas a marked blueshift is observed in less polar media (Figure S51). Both the shape of the emission and the pronounced solvatochromic behavior suggest that the emissive state of **[2a] BF_4** possesses a predominant charge-transfer (CT) character particularly in polar media, consistent with literature reports.[17] The luminescence is weak in acetonitrile (Φ = 0.05%), while it becomes more efficient in dichloromethane (Φ = 2.76%). A similar trend is observed for the excited state lifetime (Table S11). Although a slight increase in the radiative rate constants (k_F) is apparent in dichloromethane compared to acetonitrile, the comparable values suggest that the gain in fluorescence yield and lifetime is mainly governed by energy-gap-law considerations. Nevertheless, considering the expected destabilization of CT states with lowering of the solvent polarity, we cannot definitely rule out a possible contribution from the LE state in the luminescence properties in more apolar media.

The fluorescence of thioxanthylium **[3a] BF_4** is broad and structureless in acetonitrile and acetone, whereas it becomes sharper and more structured upon decreasing solvent polarity (**Figure S52**). The broad profile and weak intensity (Φ = 0.10%) of the fluorescence in acetonitrile suggest that in polar solvents the emissive state displays a predominant CT character. This hypothesis is consistent with the estimated radiative rate constant (k_F), whose value approaches those measured for the parent xanthylium **[2a] BF_4**. In contrast, the sharp and structured band (FWHM of ca 0.30 eV) and the higher emission yield and lifetime measured in dichloromethane (**Table S11**) strongly support a predominant locally excited (LE) nature of the fluorescent excited state under apolar conditions. This attribution is further substantiated by the more than 5-fold increase in radiative constant. The observed interplay between CT and LE states becomes possible due to the decrease of the energy of the π-π^* transition when moving from xanthylium to thioxanthylium, as observed in absorption spectroscopy (see above). In this regard, the observation of two lifetime components in the emission decay in acetonitrile possibly reflects the kinetics of mutual population between the CT and LE singlets and the triplet LE.

The fluorescence of the selenoxanthylium series **[4a-d] BF_4** is always sharp (FWHM of ca 0.30 eV) and insensitive to solvent polarity (**Figures S53-S56**), thus pointing to a locally excited (LE) nature of the fluorescent singlet state for all compounds under all conditions tested. This attribution is consistent with the large radiative rate constants (k_F) measured throughout the whole series (**Table S11**), which markedly differ from those measured for the parent xanthylium and thioxanthylium (under polar conditions) analogues. The weak luminescence yield and short excited state lifetime measured in the case of **[4a] BF_4** possibly indicates an active role of the CT state by providing a potential deactivation channel (see below). Instead, the comparable emission yields and singlet lifetimes measured in the case of **[4b] BF_4** and **[4c] BF_4** suggests that the CT state is not involved in the excited state deactivation.

No detectable emission signal is measured in the case of selenoxanthylium **[4d] BF_4** in acetonitrile and acetone, whereas a very weak emission is measured in dichloromethane, indicating quenching of the LE singlet by the CT state. This also highlights that, in contrast to both xanthylium and thioxanthylium, the CT state is not luminescent for selenoxanthylium.

LUMINESCENCE PROPERTIES AT 77 K

3,6-Di-*tert*-butyl-9-mesitylselenoxanthylium tetrafluoroborate ([4a] BF_4)

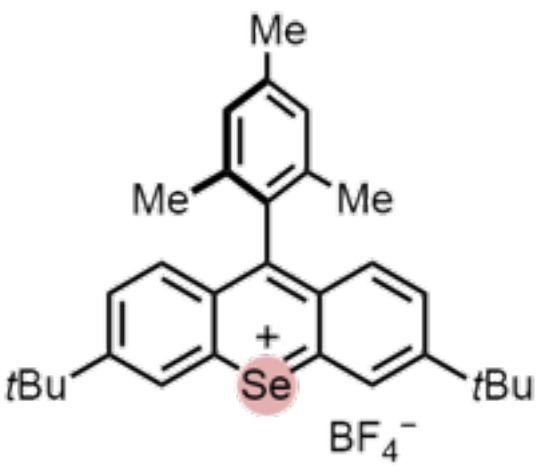

[4a] BF_4

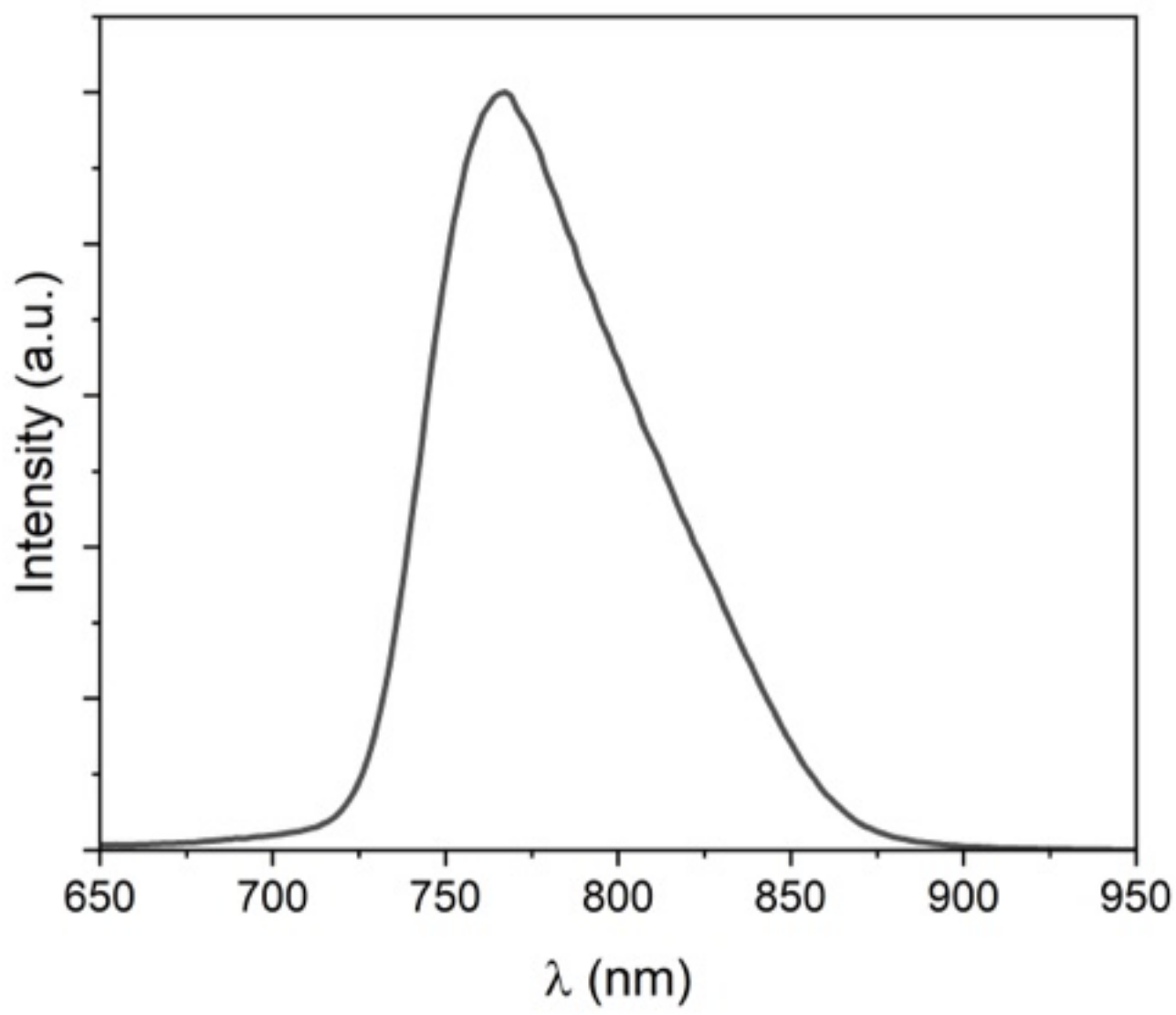

Figure S63. Time-gated emission spectrum of **[4a] BF_4** at 77K in 4:1 EtOH:MeOH glassy matrix (gate delay = 100 μs, gate width = 100 μs).

3,6-Di-*tert*-butyl-9-(4-fluoro-2,6-dimethylphenyl)selenoxanthylium tetrafluoroborate ([4b] BF_4)

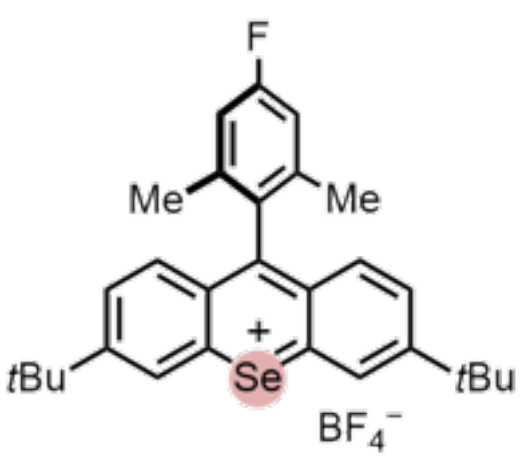

[4b] BF_4

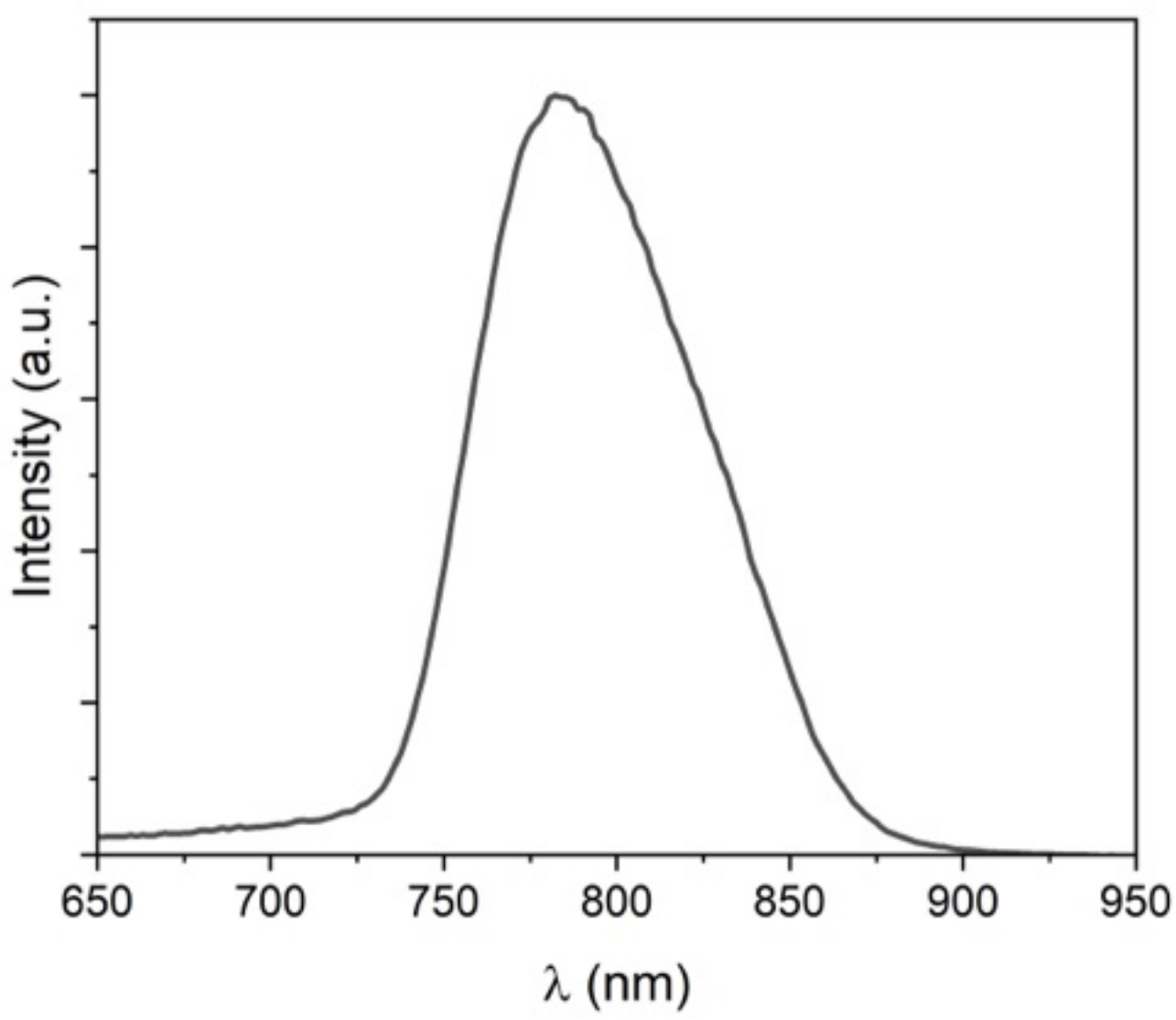

Figure S64. Time-gated emission spectrum of **[4b] BF_4** at 77K in 4:1 EtOH:MeOH glassy matrix (gate delay = 100 μs, gate width = 100 μs).

3,6-Di-*tert*-butyl-9-(2,6-dimethylphenyl)selenoxanthylium tetrafluoroborate ([4c] BF_4)

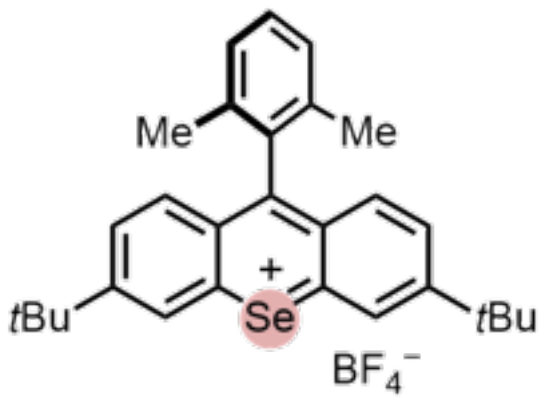

[4c] BF4

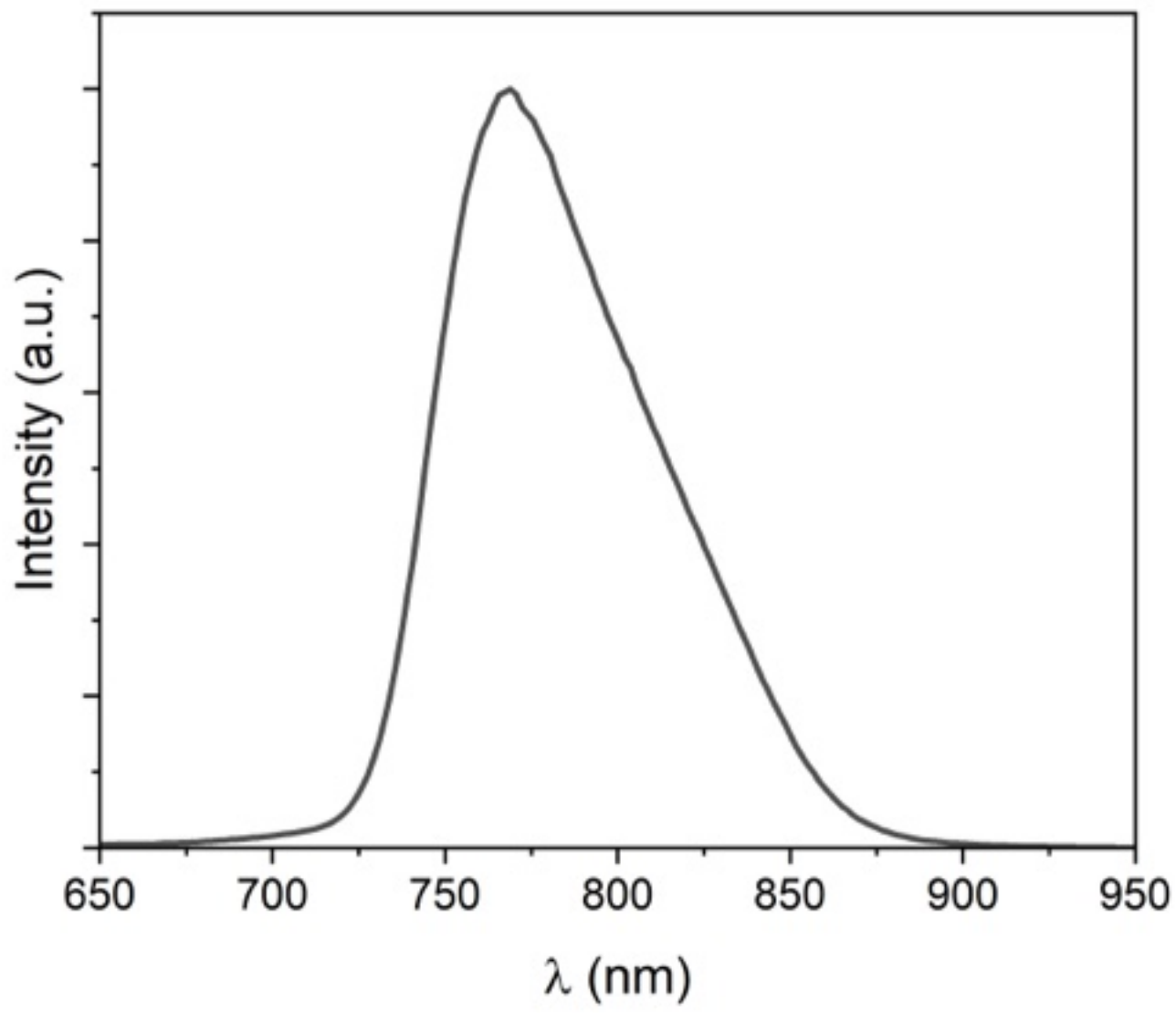

Figure S65. Time-gated emission spectrum of **[4c] BF4** at 77K in 4:1 EtOH:MeOH glassy matrix (gate delay = 100 μs, gate width = 100 μs).

3,6-Di-*tert*-butyl-9-(4-methoxy-2,6-dimethylphenyl)selenoxanthylium tetrafluoroborate ([4d] BF_4)

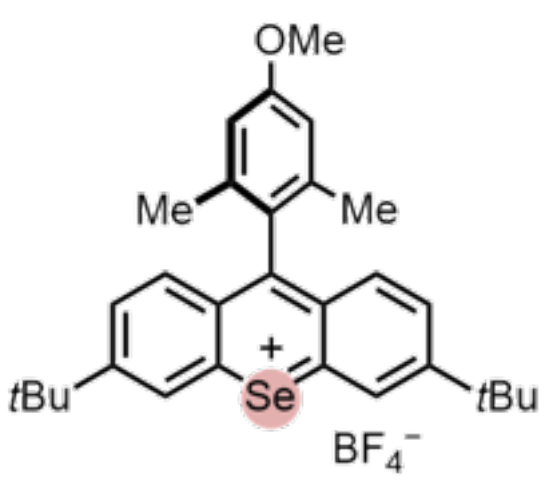

[4d] BF4

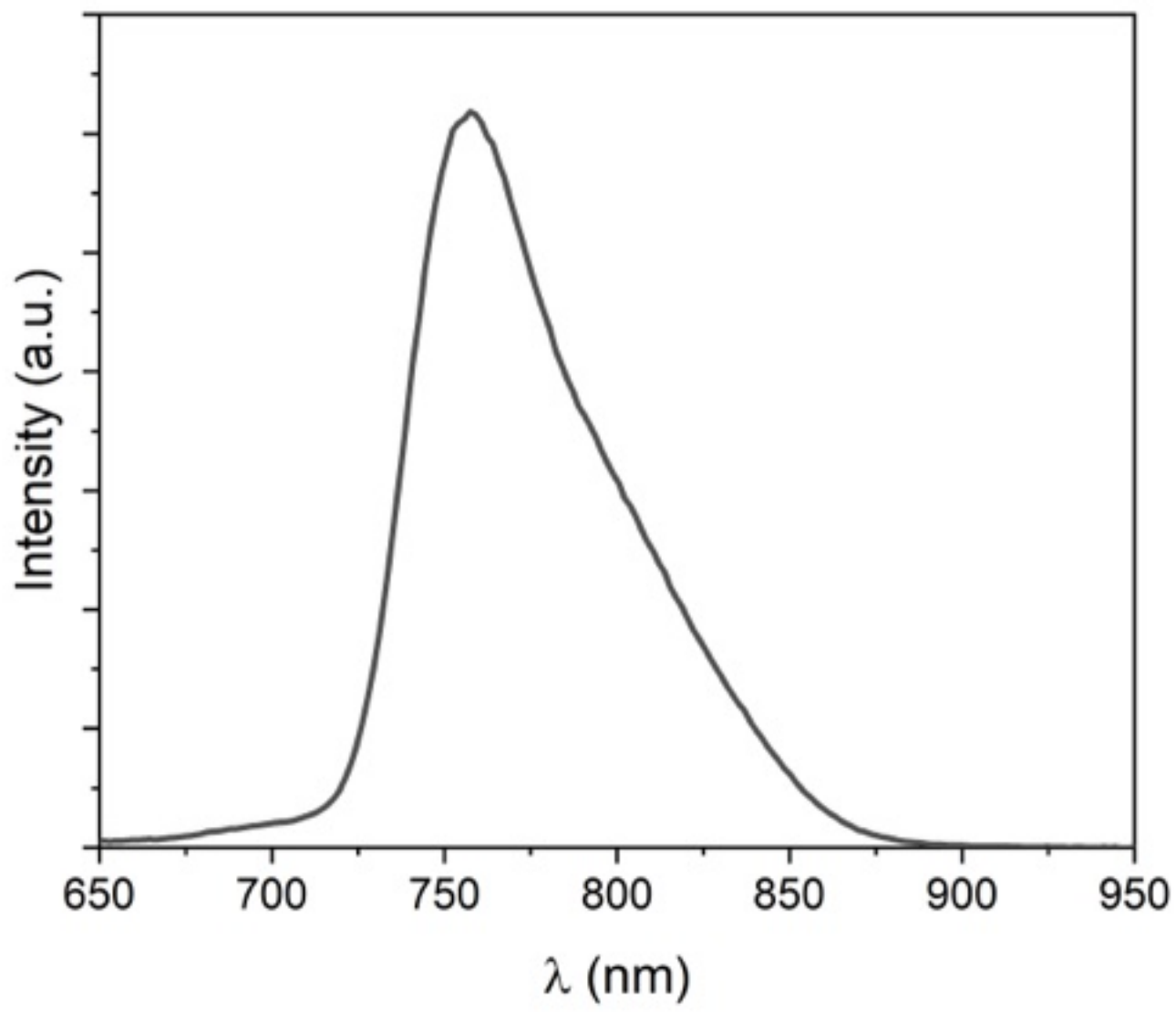

Figure S66. Time-gated emission spectrum of **[4d] BF4** at 77K in 4:1 EtOH:MeOH glassy matrix (gate delay = 100 μs, gate width = 100 μs).

Summary of Photocatalyst Photoredox Properties

Table S12. Summary of the photoredox properties of the photocatalysts studied in this work.

	$E_{p/2}^{ox}$ / V [a]	$E_{p/2}^{red}$ / V [a]	E_S / eV [b]	E^{*S}_{red} / V [a]	E_T / eV [c]	E^{*T}_{red} / V [a]
**[2a] BF_4**	-	–0.16	2.62 [17]	+2.46	-	-
**[3a] BF_4**	-	–0.14	2.30	+2.16	-	-
**[4a] BF_4**	+1.28	–0.08	2.13	+2.05	1.62	+1.54
**[4b] BF_4**	+1.66	–0.06	2.13	+2.07	1.59	+1.53
**[4c] BF_4**	+1.99	–0.08	2.15	+2.07	1.61	+1.53
**[4d] BF_4**	+1.84	–0.07	2.17	+2.10	1.64	+1.57

[a] potential referred to SCE; [b] estimated at the intersection between the normalized absorption and fluorescence spectra; [c] estimated from the maximum of the emission spectrum at 77 K.

ULTRAFAST SPECTROSCOPY

3,6-Di-*tert*-butyl-9-mesitylxanthylium tetrafluoroborate ([2a] BF_4) in CH_3CN

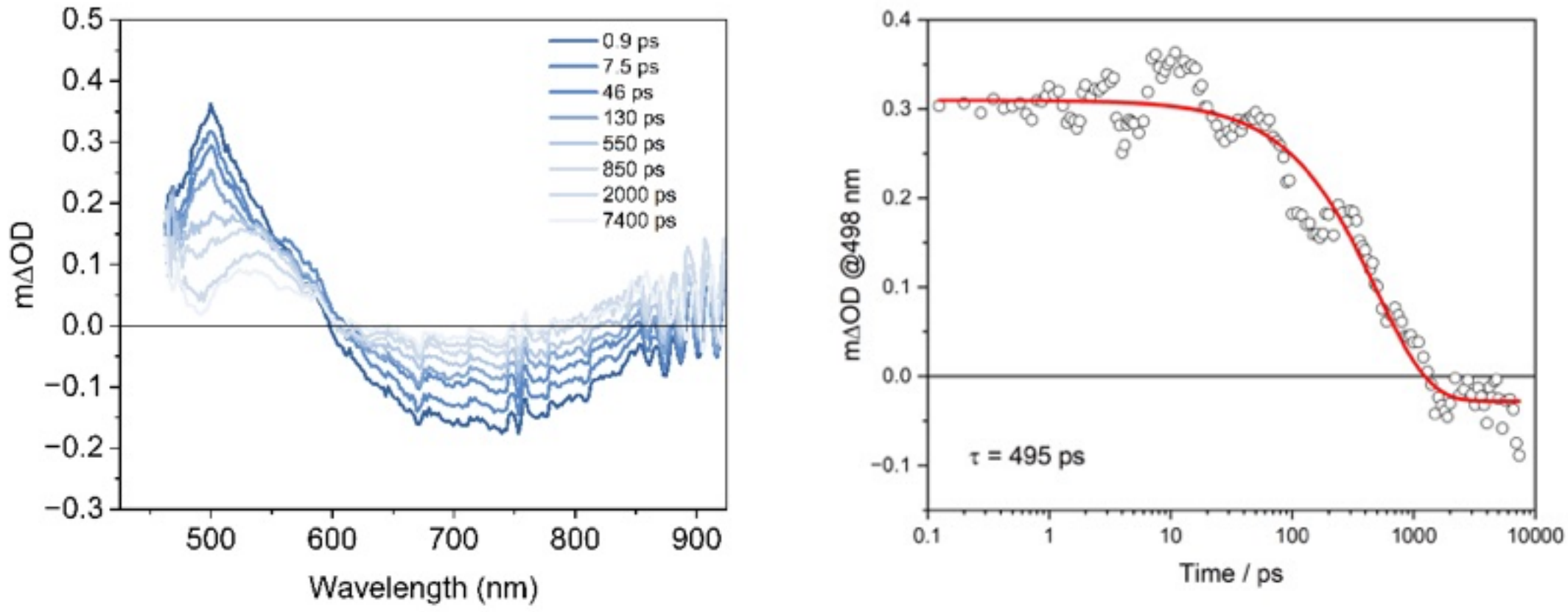

Figure S67. Transient absorption spectra of **[2a] BF4** (λ_{exc}= 343 nm) (left panel) and change of optical density at 498 nm against time (right panel) measured in CH_3CN.

The prompt spectrum is assigned to the CT state, as it displays an absorption at ca. 500 nm, which is characteristic of oxidized mesityl,[18] along with a bleaching of the stimulated emission. The spectrum evolves monotonically toward a new transient feature with maxima at ca. 550 and 850 nm, indicating the population of the LE triplet state. The time constant for this process (τ = 495 ps) is consistent with the lifetime measured by TCSPC (τ = 560 ps), thus supporting this assignment.

3,6-Di-*tert*-butyl-9-mesitylthioxanthylium tetrafluoroborate ([3a] BF_4) in CH_3CN

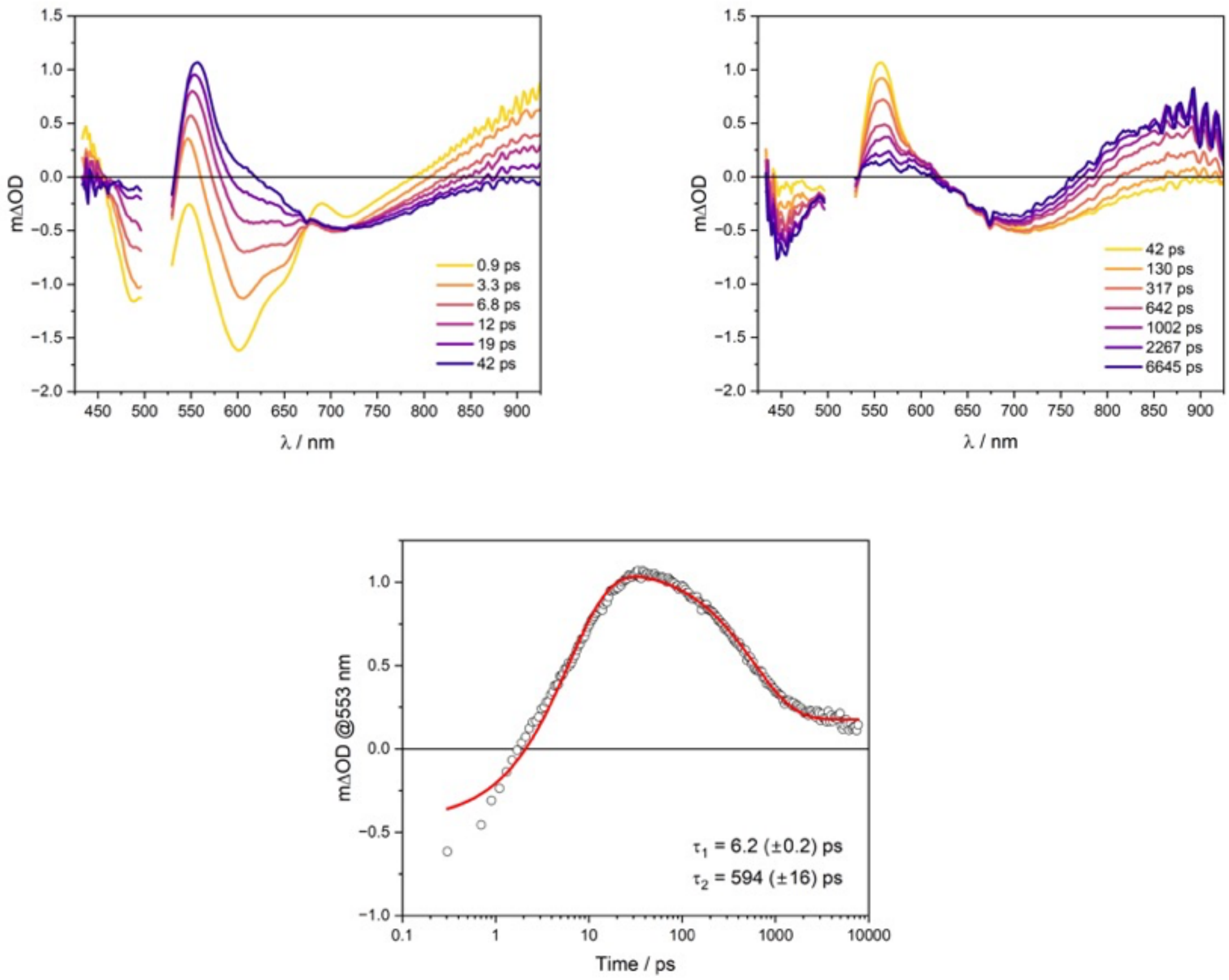

Figure S68. Transient absorption spectra of **[3a] BF_4** (λ_{exc}= 515 nm) (left and right panel) and change of optical density at 553 nm against time (bottom panel) measured in CH_3CN.

The spectral evolution is biphasic, the prompt transient can be assigned to the LE singlet state (GS bleach, narrow and blue-shifted stimulated emission, and absorption above 900 nm), the first process (top left) can be assigned to the population of a CT state from the LE singlet state (growth of the absorption at ca 550 nm and loss of blue-shifted stimulated emission), the second (top right) to the population of the LE triplet state from the CT state. The time constant of this latter process (τ = 594 ps) is consistent with the fluorescence lifetime determined by TCSPC (τ = 580 ps). The long (minor) component of the fluorescence decay (see above) is proposed to originate from repopulation of the singlet LE state from the CT.

3,6-Di-*tert*-butyl-9-mesitylselenoxanthylium tetrafluoroborate ([4a] BF_4)

Acetonitrile

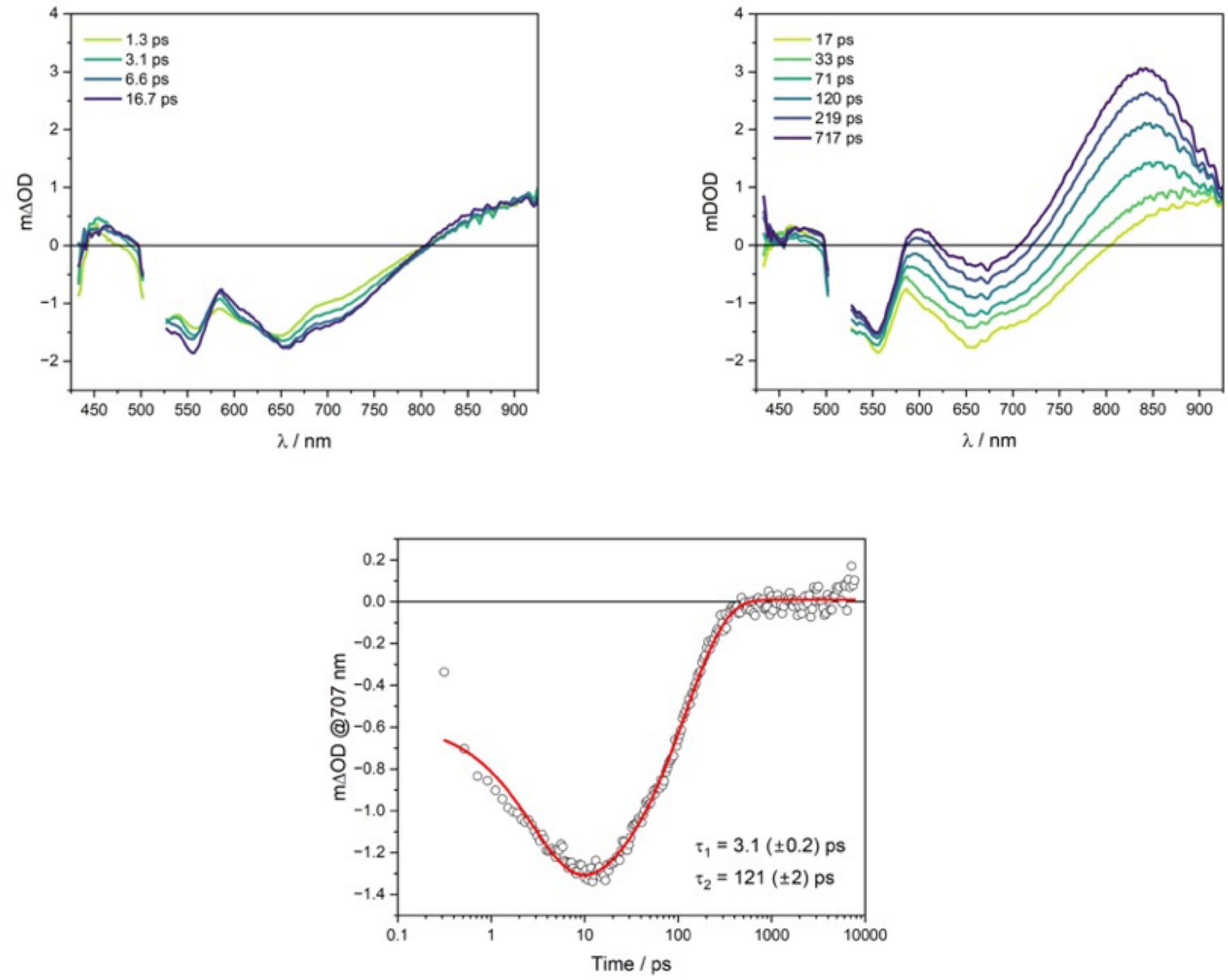

Figure S69. Transient absorption spectra of **[4a] BF_4** (λ_{exc}= 515 nm) (left and right panel) and change of optical density at 707 nm against time (bottom panel) measured in CH_3CN.

Dichloromethane

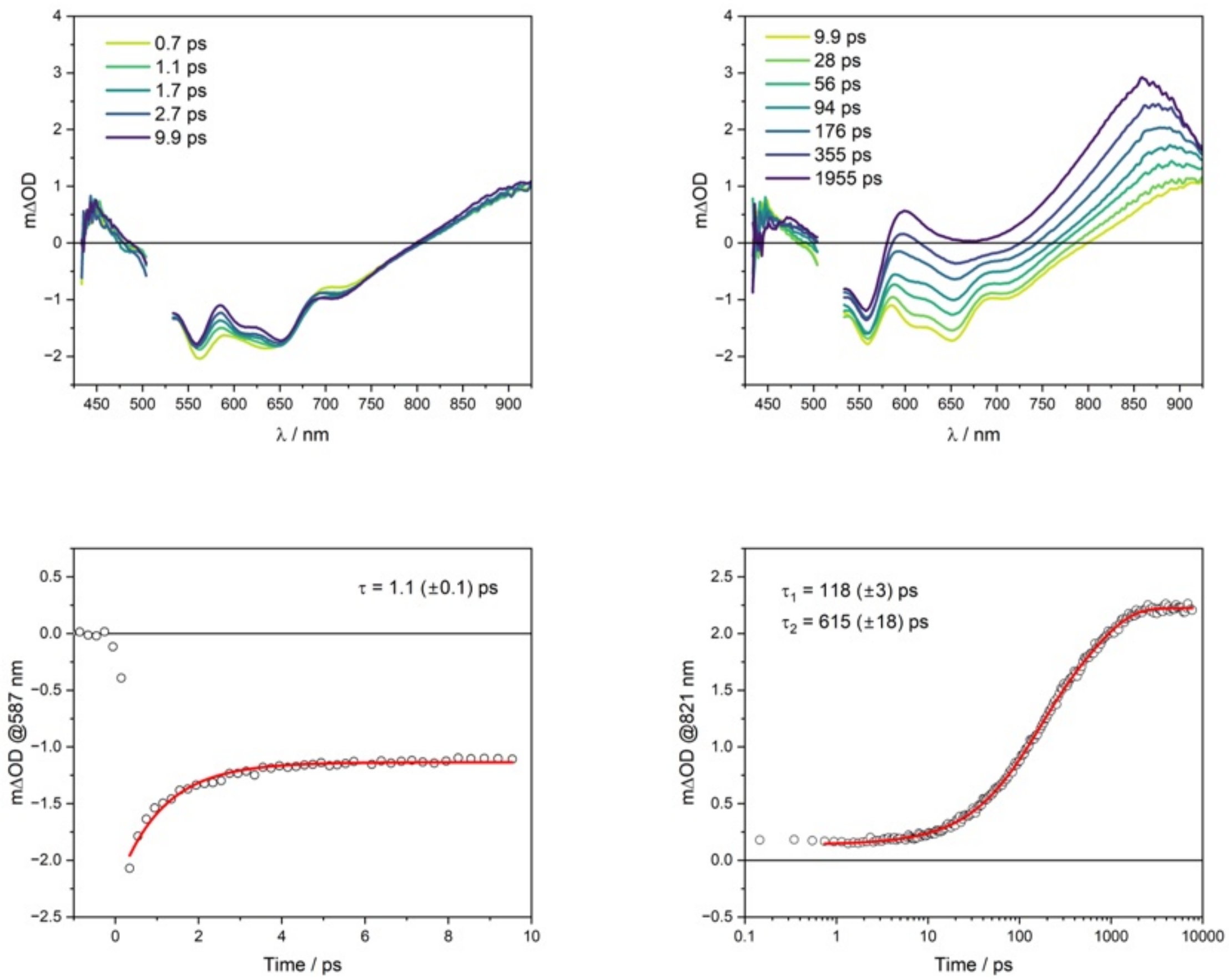

Figure S70. Transient absorption spectra of **[4a] BF$_4$** (λ_{exc}= 515 nm) (top panels) and change of optical density at 587 and 821 nm against time (bottom panels) measured in CH_2Cl_2.

*In both solvents, the spectral evolution is biphasic: the first process (top left panels) involves only a slight enhancement of ground state bleaching and likely arises from vibrational relaxation within the LE singlet excited state (with time constants of 3 ps in CH_3CN, 1.1 ps in CH_2Cl_2). The second process (top right panels) can be assigned to the population of the triplet LE state, as indicated by the loss of stimulated emission and an increase in absorption at ca. 850 nm. This attribution is consistent with the spectral fingerprint of the triplet excited state as detected by laser flash photolysis (see below). The kinetics of this process are dependent on the solvent: a single time constant (τ = 121 ps) is obtained in CH_3CN, whereas two time-components are required to fit the kinetics in CH_2Cl_2 (118 and 615 ps). Both these kinetic results are consistent with the lifetime values extracted by TCSPC data (see above). The kinetics are faster than observed in the parent **[4b,c] BF$_4$**, likely indicating the possible intermediacy of the CT state. In this respect, the two-component kinetics measured in CH_2Cl_2 might reflect a more complex dynamics along the proposed $^{S}LE \rightarrow CT \rightarrow {}^{T}LE$ process, resulting from the change in energy of the CT as a function of solvent polarity (see below for the excited state topology).*

3,6-Di-*tert*-butyl-9-(4-fluoro-2,6-dimethylphenyl)selenoxanthylium tetrafluoroborate ([4b] BF_4)

Acetonitrile

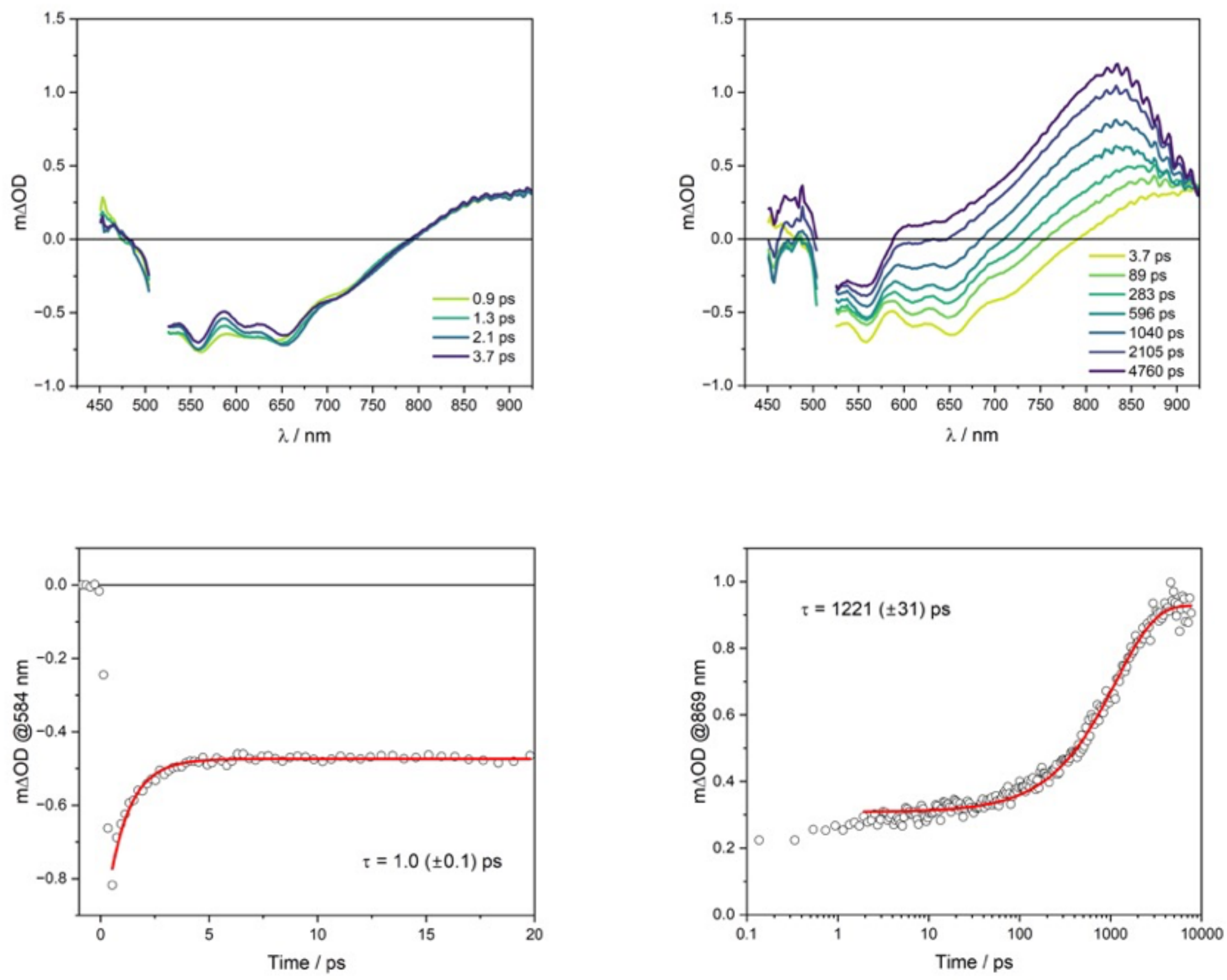

Figure S71. Transient absorption spectra of **[4b] BF_4** (λ_{exc}= 515 nm) (left and right panel) and change of optical density against time (572 nm, bottom left panel; 853 nm, bottom right panel) measured in CH_3CN.

Dichloromethane

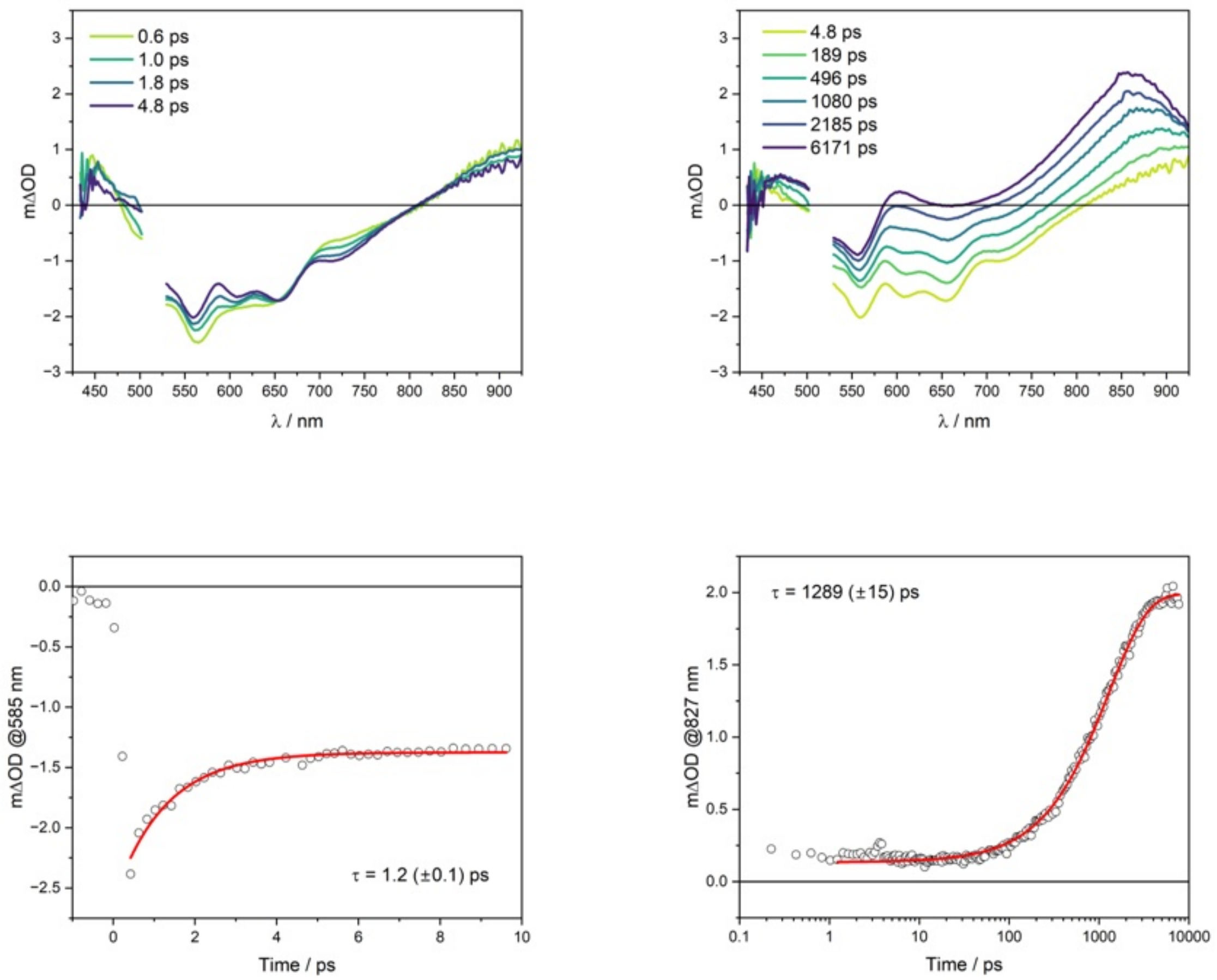

Figure S72. Transient absorption spectra of **[4b] BF$_4$** (λ_{exc}= 515 nm) (top panels) and change of optical density against time (585 nm, bottom left panel; 827 nm, bottom right panel) measured in CH_2Cl_2.

The spectral evolution of **[4b] BF$_4$** *is comparable in both acetonitrile and dichloromethane and is composed of a fast process ascribed to vibrational relaxation (top left panels) and a slower one attributable to triplet population (top right panels). The kinetics of the latter step well agree with the lifetime measured by TCSPC and are slower than those observed in the parent* **[4a] BF$_4$**, *thus supporting a direct ^{S}LE → ^{T}LE intersystem crossing.*

3,6-Di-*tert*-butyl-9-(2,6-dimethylphenyl)selenoxanthylium tetrafluoroborate ([4c] BF_4)

Acetonitrile

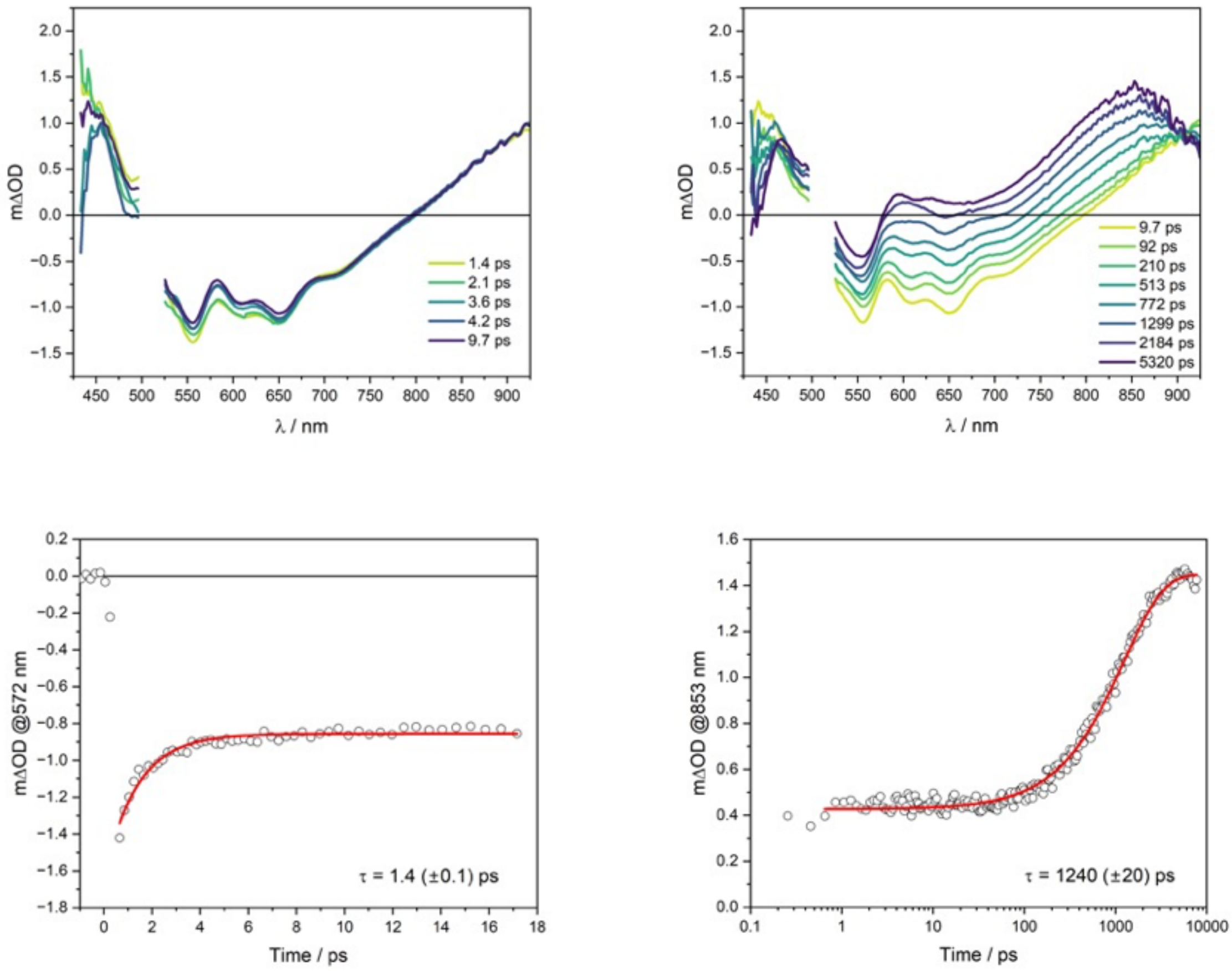

Figure S73. Transient absorption spectra of **[4c] BF_4** (λ_{exc}= 515 nm) (left and right panel) and change of optical density against time (572 nm, bottom left panel; 853 nm, bottom right panel) measured in CH_3CN.

Dichloromethane

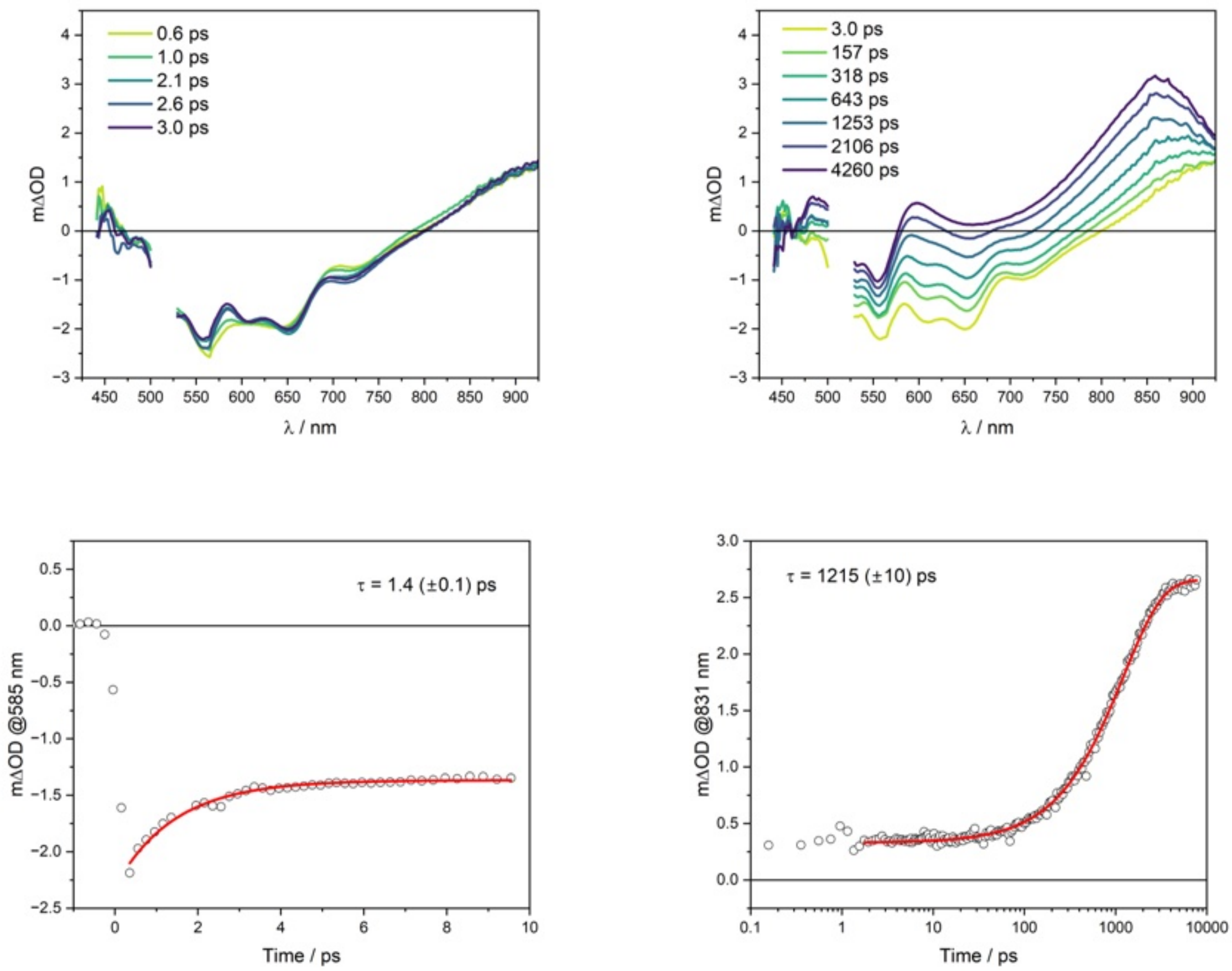

Figure S74. Transient absorption spectra of **[4c] BF₄** (λ_{exc}= 515 nm) (top panels) and change of optical density against time (585 nm, bottom left panel; 831 nm, bottom right panel) measured in CH_2Cl_2.

*The spectral evolution of **[4c] BF$_4$** is comparable in both acetonitrile and dichloromethane and similar with that of **[4b] BF$_4$**. It consists of a fast process (vibrational relaxation, left panels) and a slower one (triplet population, right panels). The kinetics of the latter step agree with the lifetime measured by TCSPC (see above).*

3,6-Di-*tert*-butyl-9-(4-methoxy-2,6-dimethylphenyl)selenoxanthylium tetrafluoroborate ([4d] BF_4)

Acetonitrile

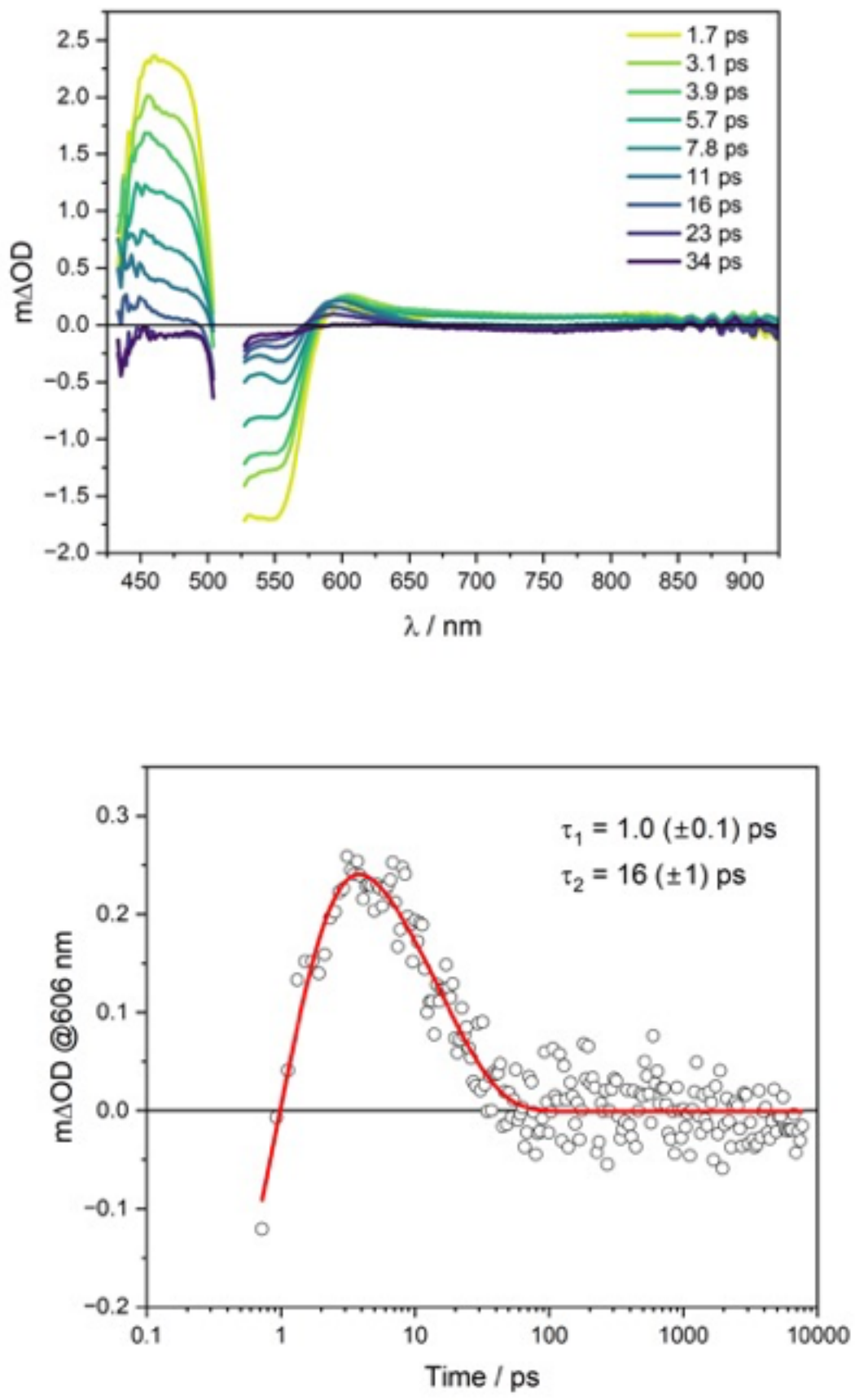

Figure S75. Transient absorption spectra of **[4d] BF_4** (λ_{exc}= 515 nm) (top) and change of optical density against time at 606 nm (bottom) measured in CH_3CN.

Dichloromethane

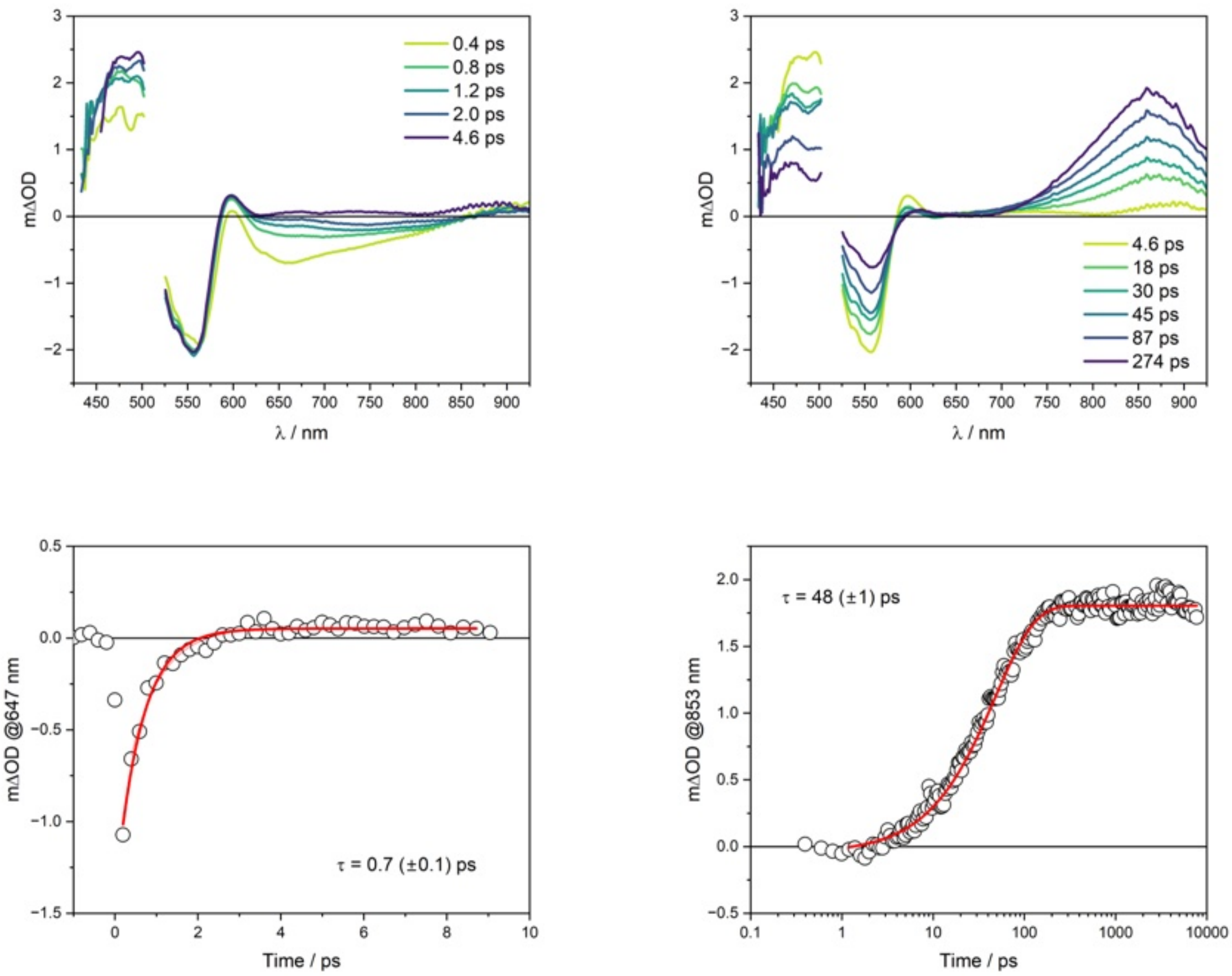

Figure S76. Transient absorption spectra of **[4d] BF$_4$** (λ_{exc}= 515 nm) (top) and change of optical density against time at 647 nm (bottom left) and 853 nm (bottom right) measured in CH_2Cl_2.

*The excited-state behavior differs substantially in the two solvents tested. In acetonitrile, the dynamics are complete within less than 100 ps. The spectral changes mainly involve the decay of both the excited-state absorption at ca. 450 nm and the ground-state bleaching, accompanied by a rise-and-decay feature at ca. 600 nm. This behavior can be interpreted as an ultrafast population of the CT state (τ = 1 ps), followed by a rapid decay (τ = 16 ps) back to the ground state. The dynamics are markedly different from those of the parent **[4a-c] BF$_4$** systems and likely originate from the presence of the electron-donating –OMe group, which facilitates oxidation of the para-methoxyxylyl moiety thus lowering the energy of the CT state. This behavior can account for the absence of any detectable luminescence as well as for the failure to detect any transient species by nanosecond transient absorption spectroscopy in acetonitrile. Ultrafast formation of the CT state is detected also in dichloromethane. However, the CT state does not decay to the ground state as in acetonitrile. Instead, it undergoes fast (48 ps) population of the triplet excited state, highlighting the strong dependence of the CT energy on solvent polarity.*

Nanosecond Laser Flash Photolysis

3,6-Di-*tert*-butyl-9-mesitylselenoxanthylium tetrafluoroborate ([4a] BF_4)

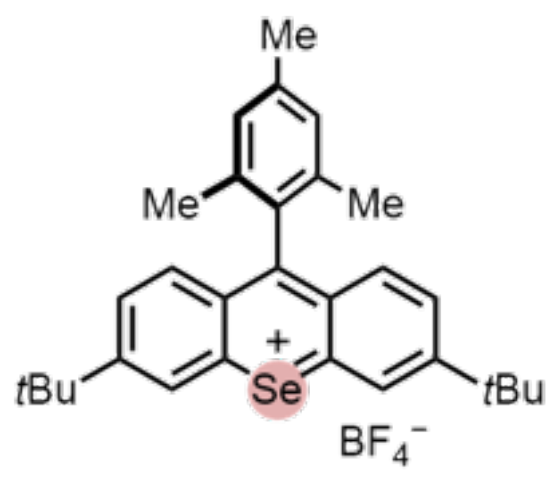

[4a] BF_4

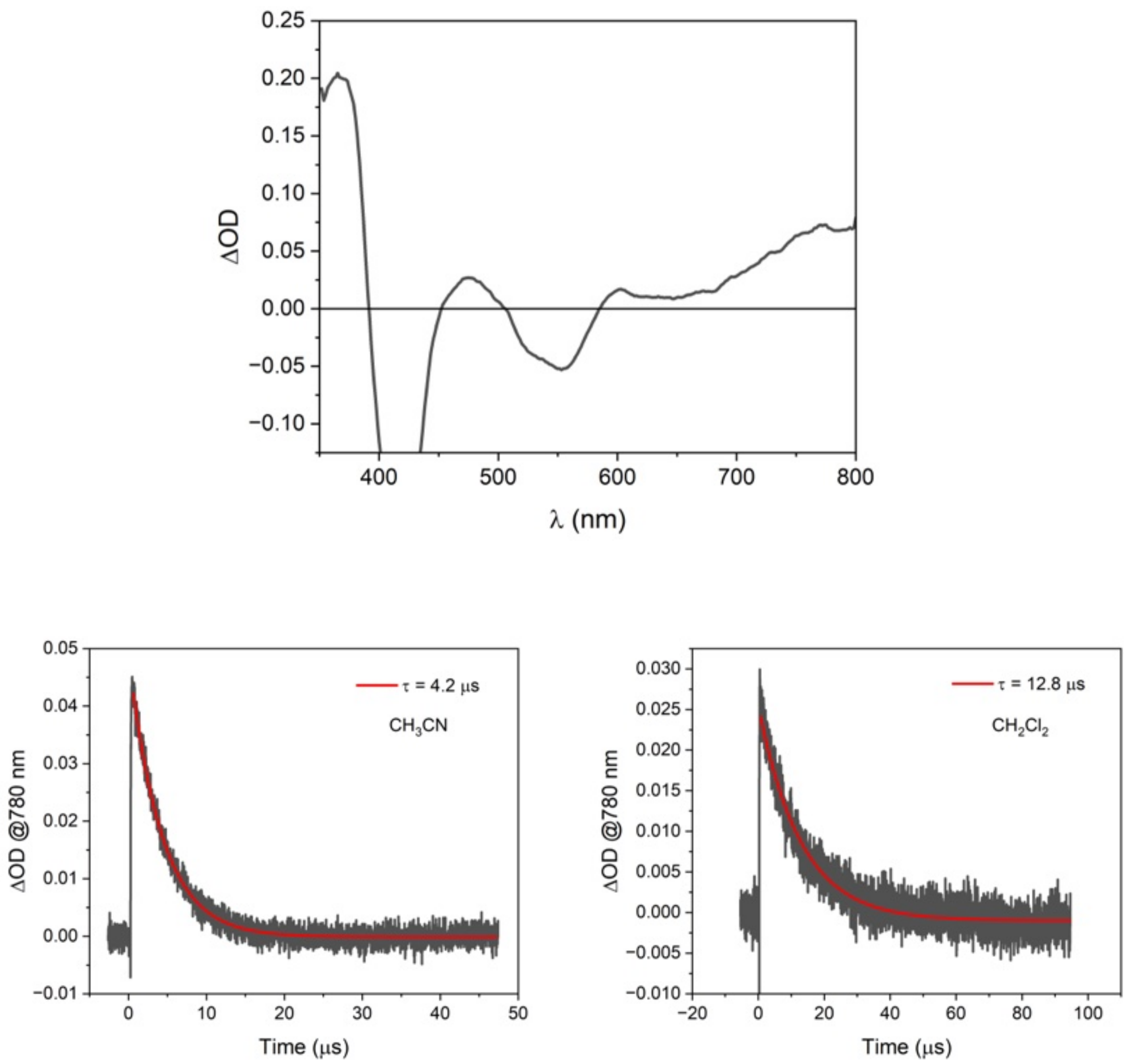

Figure S77. Transient absorption spectrum (gate delay = 100 ns, gate width = 50 ns) of the triplet excited state (top) recorded by nanosecond laser flash photolysis of **[4a] BF_4** in N_2-purged CH_3CN after excitation at 532 nm and kinetic traces at 780 nm in CH_3CN (bottom left) and CH_2Cl_2 (bottom right).

3,6-Di-*tert*-butyl-9-(4-fluoro-2,6-dimethylphenyl)selenoxanthylium tetrafluoroborate ([4b] BF_4)

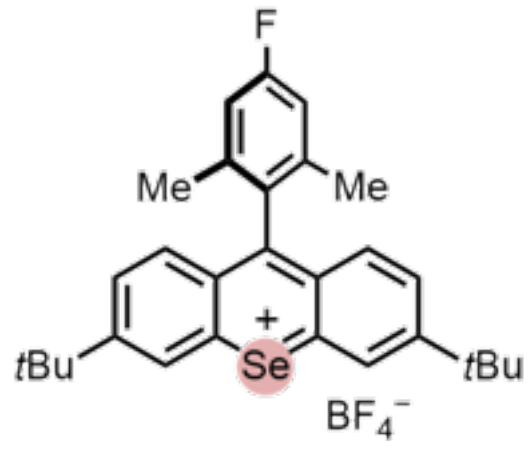

[4b] BF_4

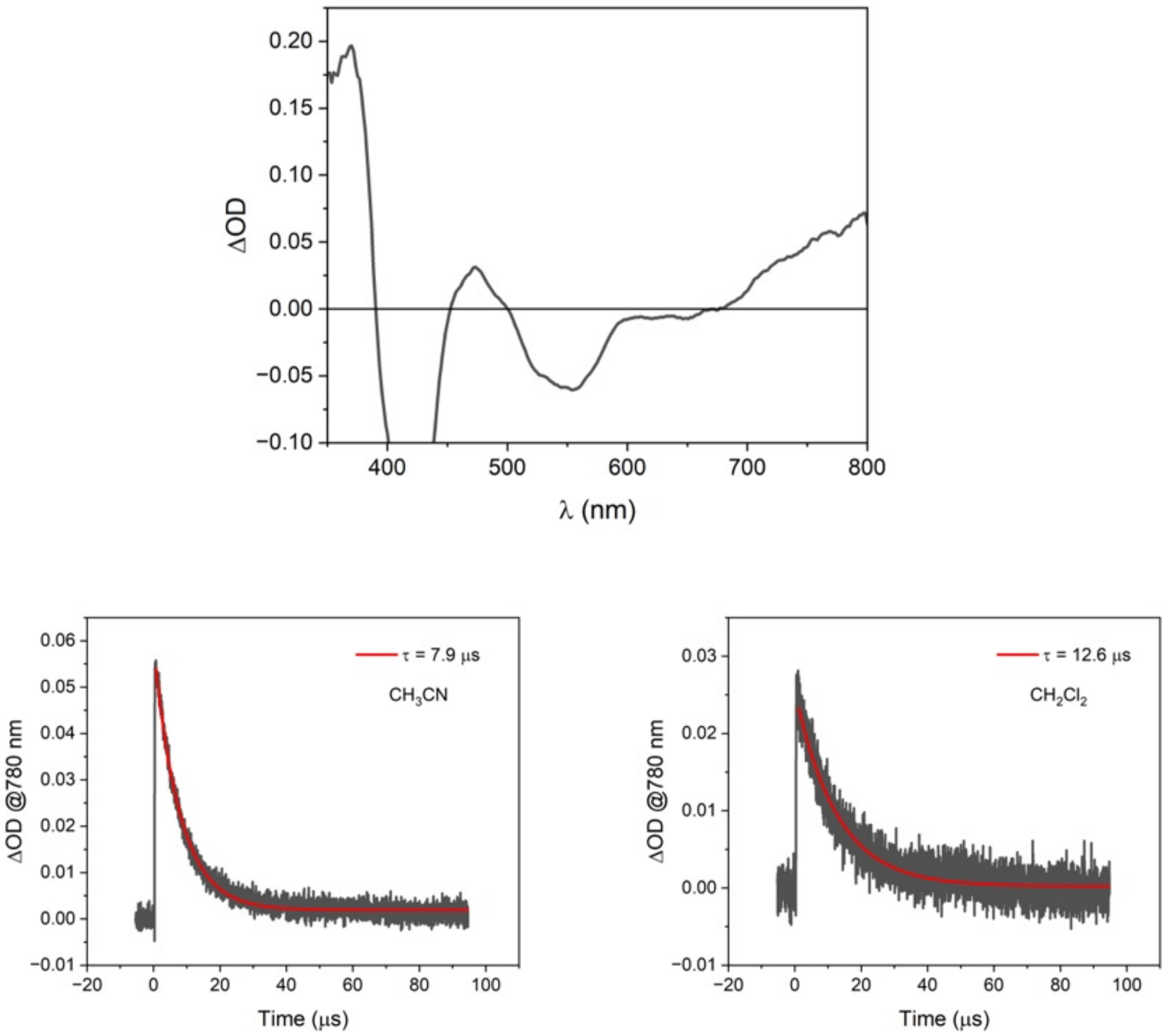

Figure S78. Transient absorption spectrum (gate delay = 100 ns, gate width = 50 ns) of the triplet excited state (top) recorded by nanosecond laser flash photolysis of **[4b] BF_4** in N_2-purged CH_3CN after excitation at 532 nm and kinetic traces at 780 nm in CH_3CN (bottom left) and CH_2Cl_2 (bottom right).

3,6-Di-*tert*-butyl-9-(2,6-dimethylphenyl)selenoxanthylium tetrafluoroborate ([4c] BF_4)

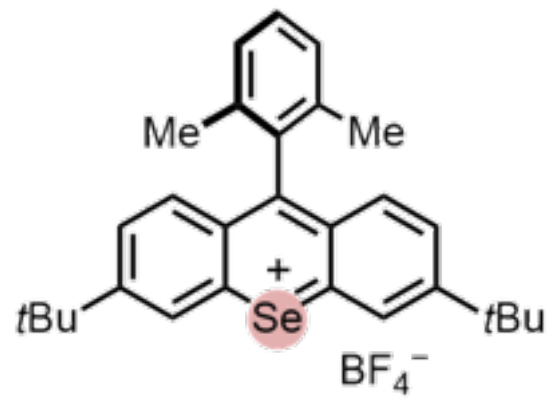

[4c] BF_4

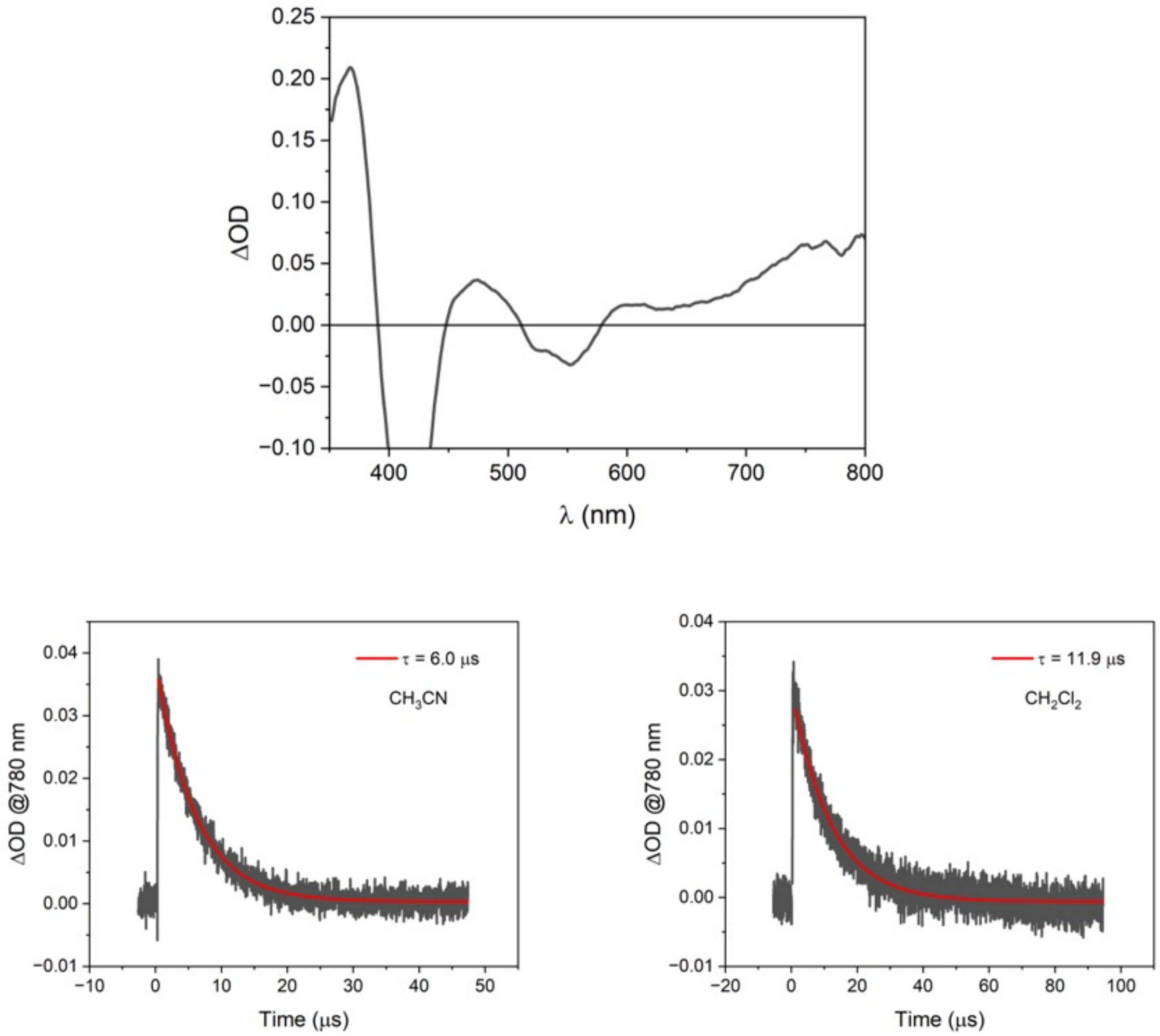

Figure S79. Transient absorption spectrum (gate delay = 100 ns, gate width = 50 ns) of the triplet excited state (top) recorded by nanosecond laser flash photolysis of **[4c] BF_4** in N_2-purged CH_3CN after excitation at 532 nm and kinetic traces at 780 nm in CH_3CN (bottom left) and CH_2Cl_2 (bottom right).

3,6-Di-*tert*-butyl-9-(4-methoxy-2,6-dimethylphenyl)selenoxanthylium tetrafluoroborate ([4d] BF_4)

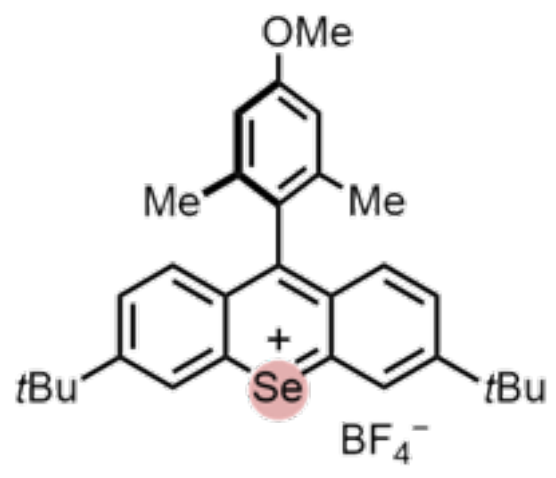

[4d] BF_4

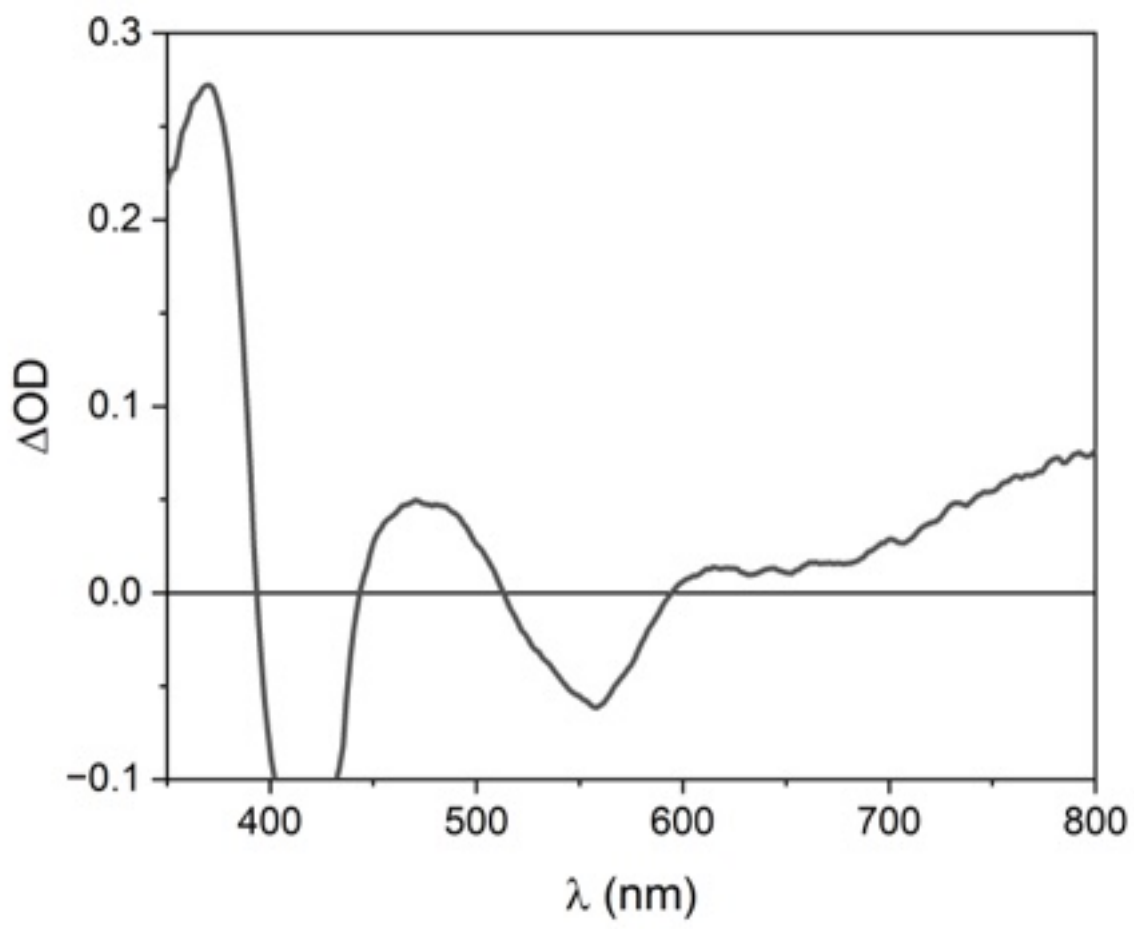

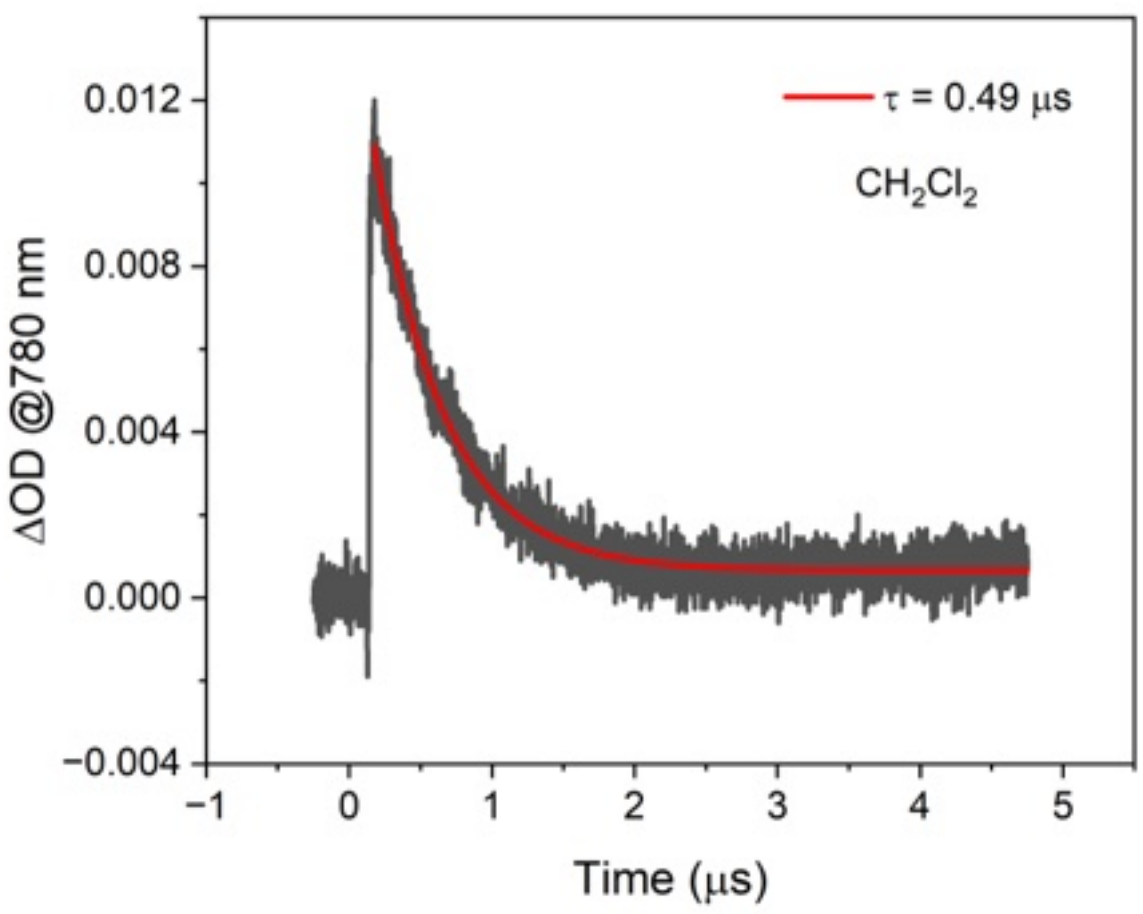

Figure S80. Transient absorption spectrum (gate delay = 100 ns, gate width = 50 ns) of the triplet excited state (top) recorded by nanosecond laser flash photolysis of **[4d] BF_4** in N_2-purged CH_2Cl_2 after excitation at 532 nm and kinetic trace at 780 nm (bottom). No transient species is detected by laser flash photolysis in CH_3CN in agreement with the results by ultrafast spectroscopy (see above).

Summary of Laser Flash Photolysis

Table S13. Summary of the laser flash photolysis at 298 K of the Se photocatalysts studied in this work.

	CH_3CN		*CH_2Cl_2*	
	τ_T / µs [a]	η_T / % [b]	τ_T / µs[a]	η_T / % [b]
**[4a] BF_4**	4.2	0.68 (±0.13)	12.8	0.59 (±0.11)
**[4b] BF_4**	7.9	0.65 (±0.12)	12.6	0.63 (±0.12)
**[4c] BF_4**	6.0	0.66 (±0.12)	11.9	0.61 (±0.11)
**[4d] BF_4**	-	-	0.49	0.22 (±0.04)

[a] measured by laser flash photolysis (excitation at 532 nm); [b] triplet yield estimated via actinometry using $[Ru(bpy)_3]^{2+}$ in H_2O as a reference ($\Delta\varepsilon$ = –11,000 $M^{-1}cm^{-1}$ at 450 nm and η_{ISC} = 1 for the triplet excited state),[19] the given range results from the use of two different $\Delta\varepsilon$ at 780 nm for the selenoxanthylium triplet excited state (a $\Delta\varepsilon$ = 1,300 $M^{-1}cm^{-1}$ was calculated according to method proposed by Hadley and Keller based on the depletion of the singlet excited state absorption,[20] while a $\Delta\varepsilon$ = 1,900 $M^{-1}cm^{-1}$ was estimated by laser flash photolysis through triplet-triplet energy transfer using benzophenone as the sensitizer upon excitation at 355 nm, for sake of simplicity the $\Delta\varepsilon$ was estimated for **[4a] BF_4** and assumed to be comparable within the **[4a-d] BF_4** series).

EXCITED STATE TOPOLOGY OF THE SELENOXANTHYLIUM PHOTOCATALYSTS

3,6-Di-*tert*-butyl-9-mesitylselenoxanthylium tetrafluoroborate ([4a] BF_4)

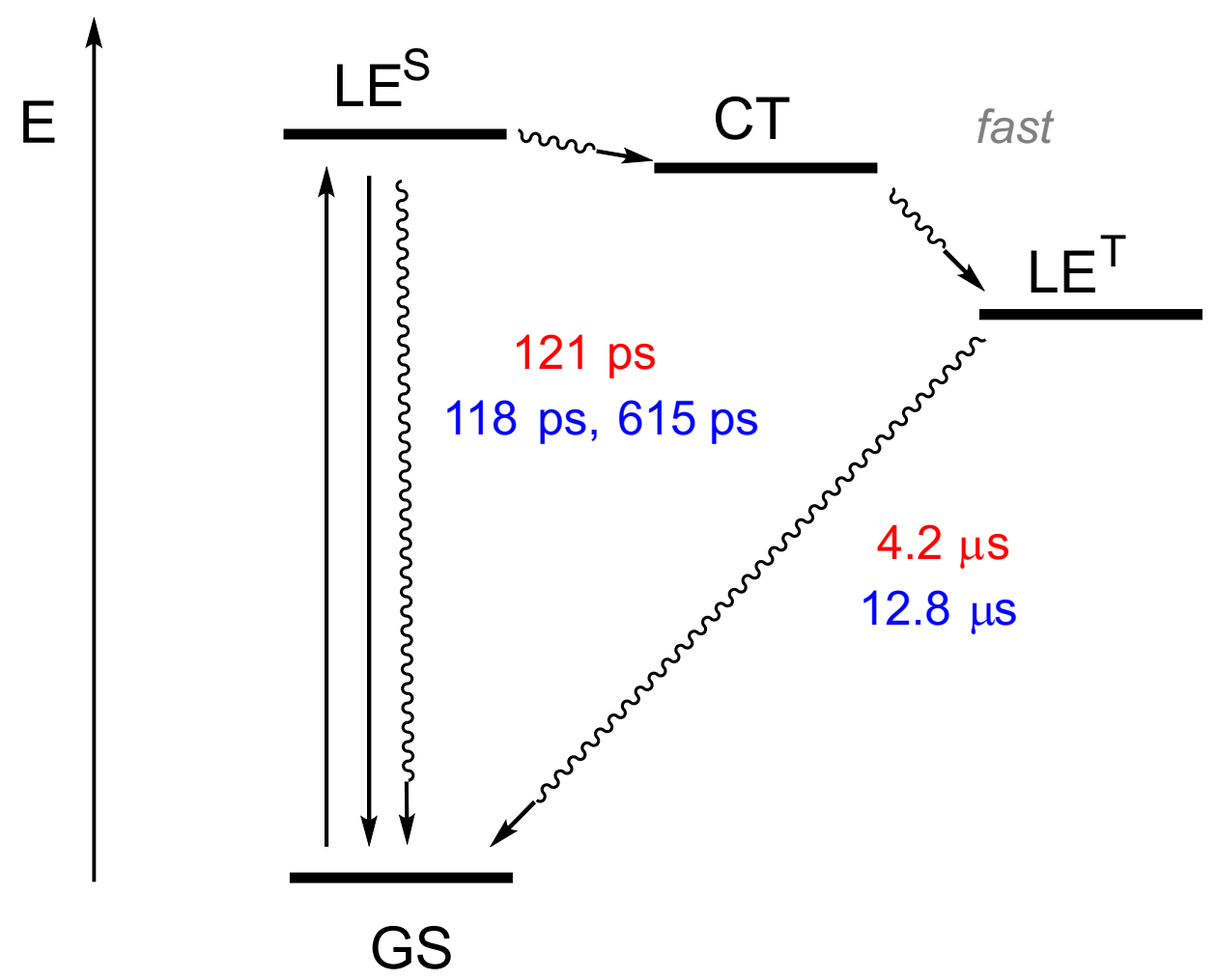

Figure S81. Energy diagram for **[4a] BF_4** with rate constants in CH_3CN (red) and CH_2Cl_2 (blue).

3,6-Di-*tert*-butyl-9-(4-fluoro-2,6-dimethylphenyl)selenoxanthylium tetrafluoroborate ([4b] BF_4)

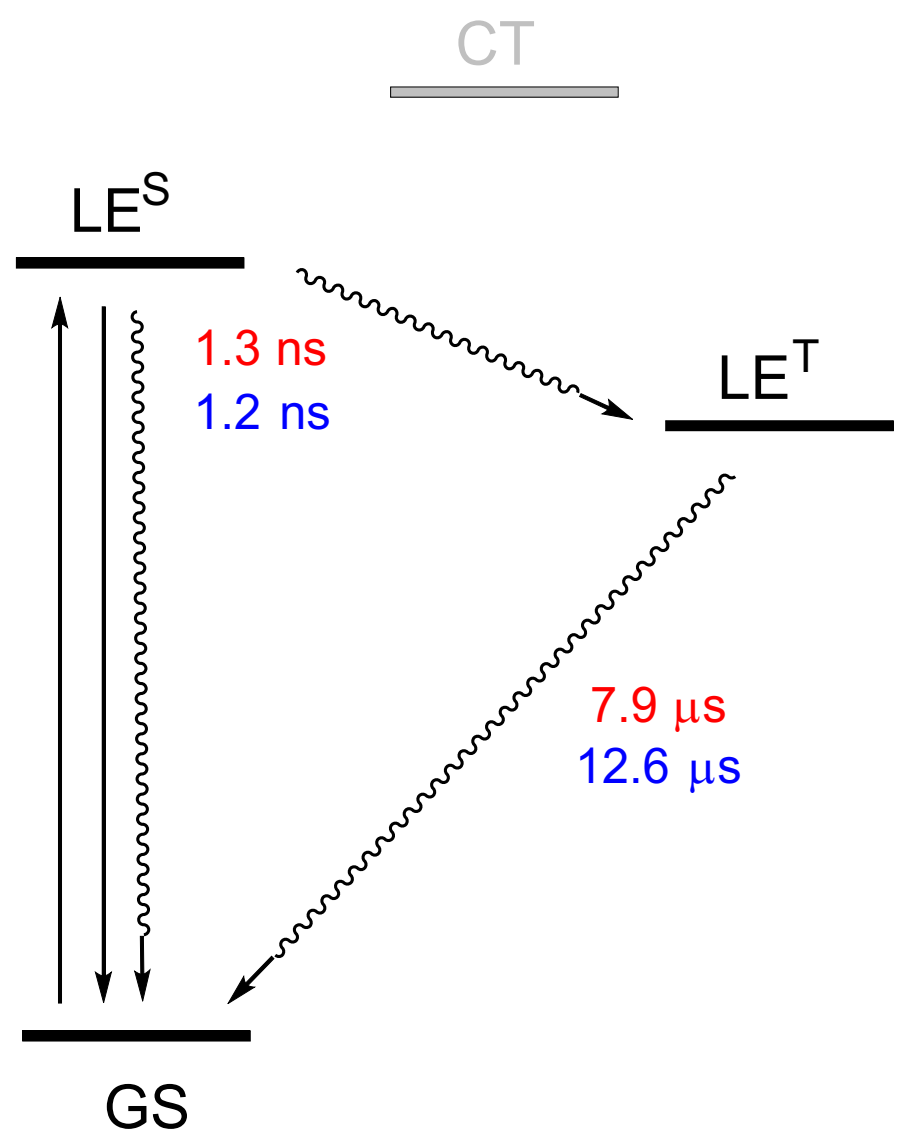

Figure S82. Energy diagram for **[4b] BF_4** with rate constants in CH_3CN (red) and CH_2Cl_2 (blue).

3,6-Di-*tert*-butyl-9-(2,6-dimethylphenyl)selenoxanthylium tetrafluoroborate ([4c] BF_4)

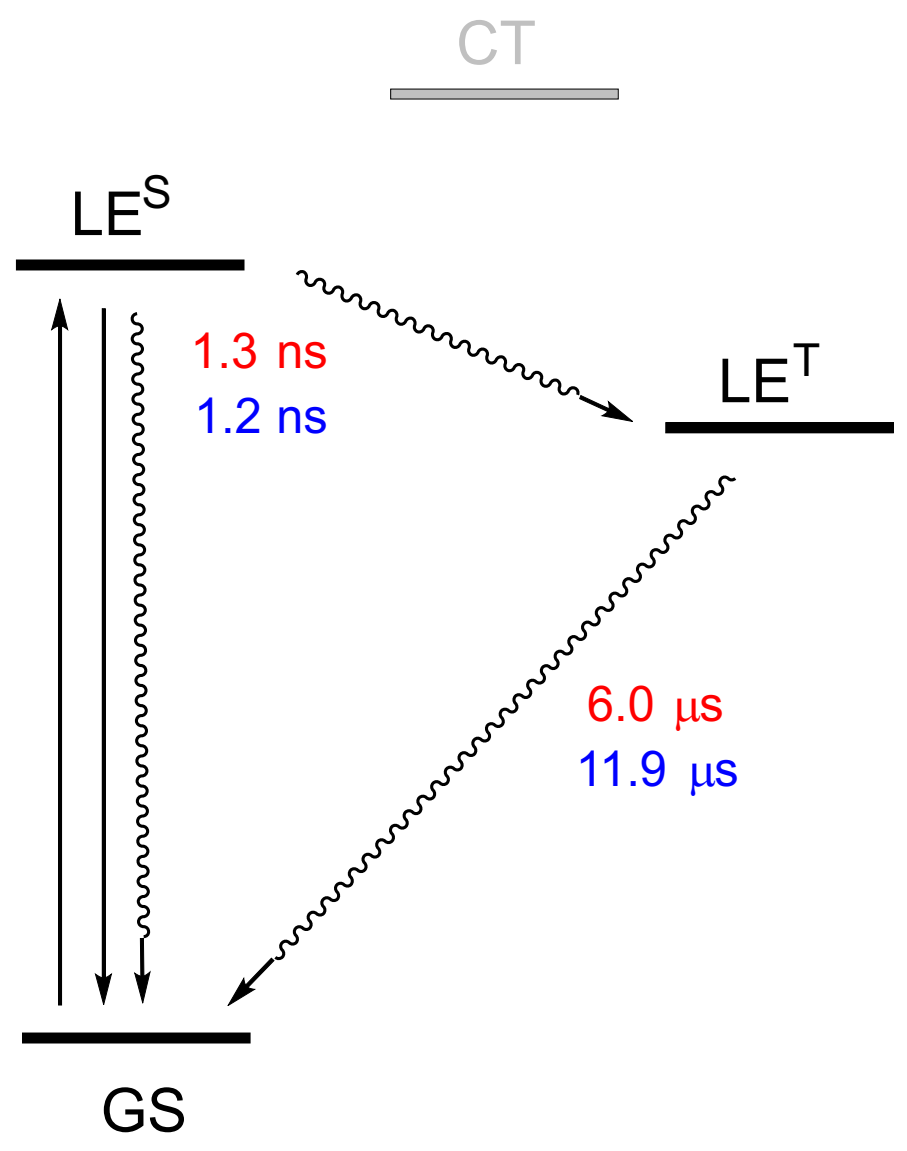

Figure S83. Energy diagram for **[4c] BF_4** with rate constants in CH_3CN (red) and CH_2Cl_2 (blue).

3,6-Di-*tert*-butyl-9-(4-methoxy-2,6-dimethylphenyl)selenoxanthylium tetrafluoroborate ([4d] BF_4)

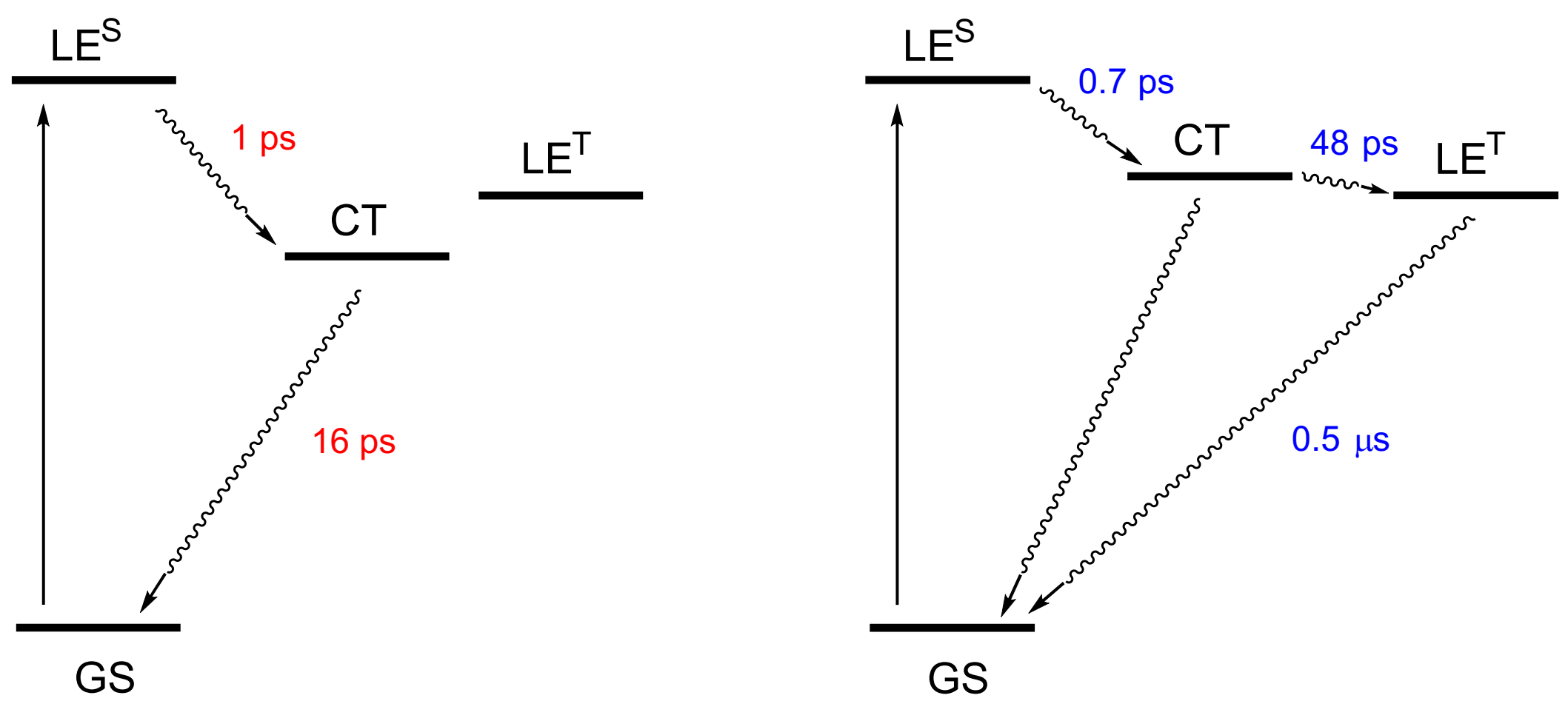

Figure S84. Energy diagram for **[4d] BF_4** with rate constants in CH_3CN (left) and CH_2Cl_2 (right).

Final comments on the photophysics

*The photophysics of the xanthylium derivatives are governed by an interplay between LE and CT states, the energies of which depend on the nature of the chalcogen, the substituent on the mesityl group, and the solvent. The progressive change of chalcogen from O to Se within the mesityl series mainly lowers the energy of the LE states (singlet and triplet), while the CT state remains practically unaltered. This is experimentally supported by the observed switch from CT emission for **[2a] BF_4**, to a combination of LE and CT emission for **[3a] BF_4**, and finally to a pure LE state fluorescence for **[4a] BF_4**. As a note, we have purposely not considered spin multiplicity for the CT state: due to the twisted configuration of the excited molecule in the CT state, the unpaired electrons are indeed expected to be weakly coupled, thereby resulting in negligible exchange energy that makes the singlet and triplet CT states practically degenerate.*

*As far as the selenoxanthylium series **[4a-d] BF_4** is concerned, the chemical nature of the substituent on the group in position 9 of the xanthylium core has a critical role on the photophysics. With an electron withdrawing group such as -F and with -H (i.e., in compounds **[4b,c] BF_4**), the selenoxanthylium behaves as a classical organic chromophore featuring singlet and triplet LE states at lower energies. In both cases and regardless of the solvent, excitation into the singlet LE is followed by fluorescence and concomitant intersystem crossing to the triplet LE. With the mesityl group (i.e., in compound **[4a] BF_4**), similar dynamics are observed, although a noticeable shortening of the singlet LE state lifetime is recorded which is ascribed to a $LE^S \rightarrow CT \rightarrow LE^T$ process, whereby triplet population is attained through the intermediacy of the CT state. On the contrary, with an electron-donating group like -OMe (i.e., in compound **[4d] BF_4**), the CT state is efficiently populated leading to loss of fluorescence. For this latter, subsequent population of the triplet excited state from the CT state is feasible only in a less polar solvent, whereas in more polar solvents such as acetonitrile direct ground state decay is observed.*

*All in all, the generally short singlet excited state lifetime and the effective population of the triplet excited state suggest that the selenoxanthylium chromophores **[4a-d] BF_4** behave as triplet photocatalysts.*

PRIMARY PHOTOCHEMICAL EVENT INVOLVING SELENOXANTHYLIUM PHOTOCATALYSTS

*In order to confirm that the novel selenoxanthylium chromophores behave as triplet photocatalysts, we have performed laser flash photolysis studies using **[4a] BF$_4$** as a reference photocatalyst within two different photoreactions, namely [4+2] cycloaddition and C(sp2)-H amination.*

[4+2] cycloaddition

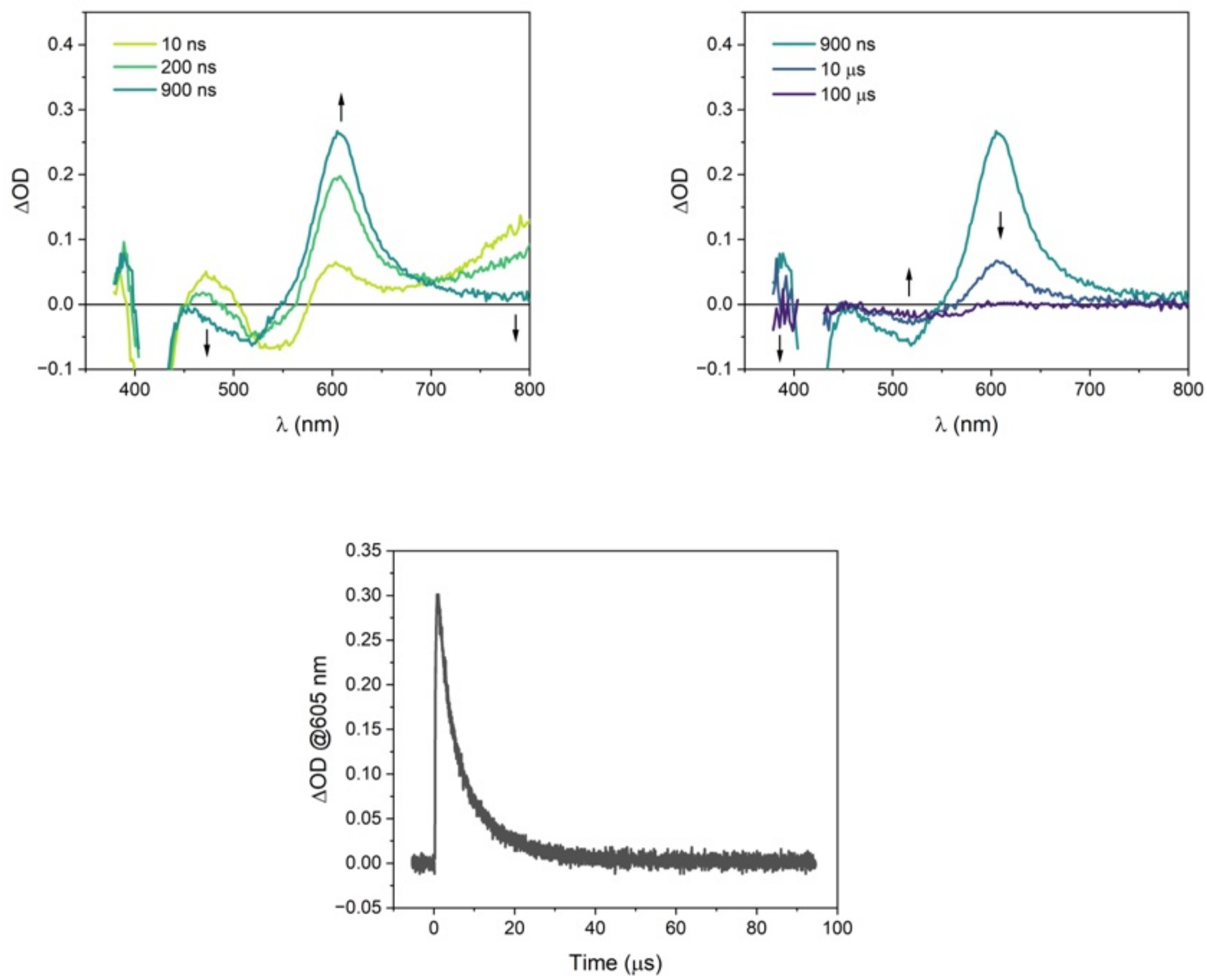

Figure S85. Transient absorption spectra at different time delays (gate width = 50 ns) obtained by laser flash photolysis (excitation at 532 nm) of **[4a] BF$_4$** in N_2-purged CH_3NO_2 in the presence of 0.5 mM **7** (top) and kinetic trace at 605 nm (bottom).

*Laser flash photolysis of **[4a] BF$_4$** in the presence of 0.5 mM **7** is biphasic. The prompt spectrum is the fingerprint of the triplet LE state, indicating efficient population of the triplet even in the presence of the substrate. The first process (**Figure S85**, top left) occurs within ca 1 μs and shows the decrease of the characteristic absorption of the triplet LE state at 780 nm and the concomitant rise of an absorption at 605 nm assigned to the reduced photocatalyst; the second process occurs within the following 100 μs and features a decay of the latter transient signal to the baseline (**Figure S85**, top right). The first process can be assigned to the photoinduced electron transfer between the triplet state of **[4a] BF$_4$** and*

*trans-anethole **7** leading to the reduced photocatalyst **[4a]**$^{\bullet-}$ and the oxidized **7**$^{\bullet+}$, while the second process to the ensuing charge recombination. Stern-Volmer analysis performed on the triplet absorption signal at 780 nm (**Figure S86**) confirms bimolecular quenching of the triplet excited state by **7** which occurs with a rate constant of k = 6.3·10^{9} M^{-1}s^{-1}.*

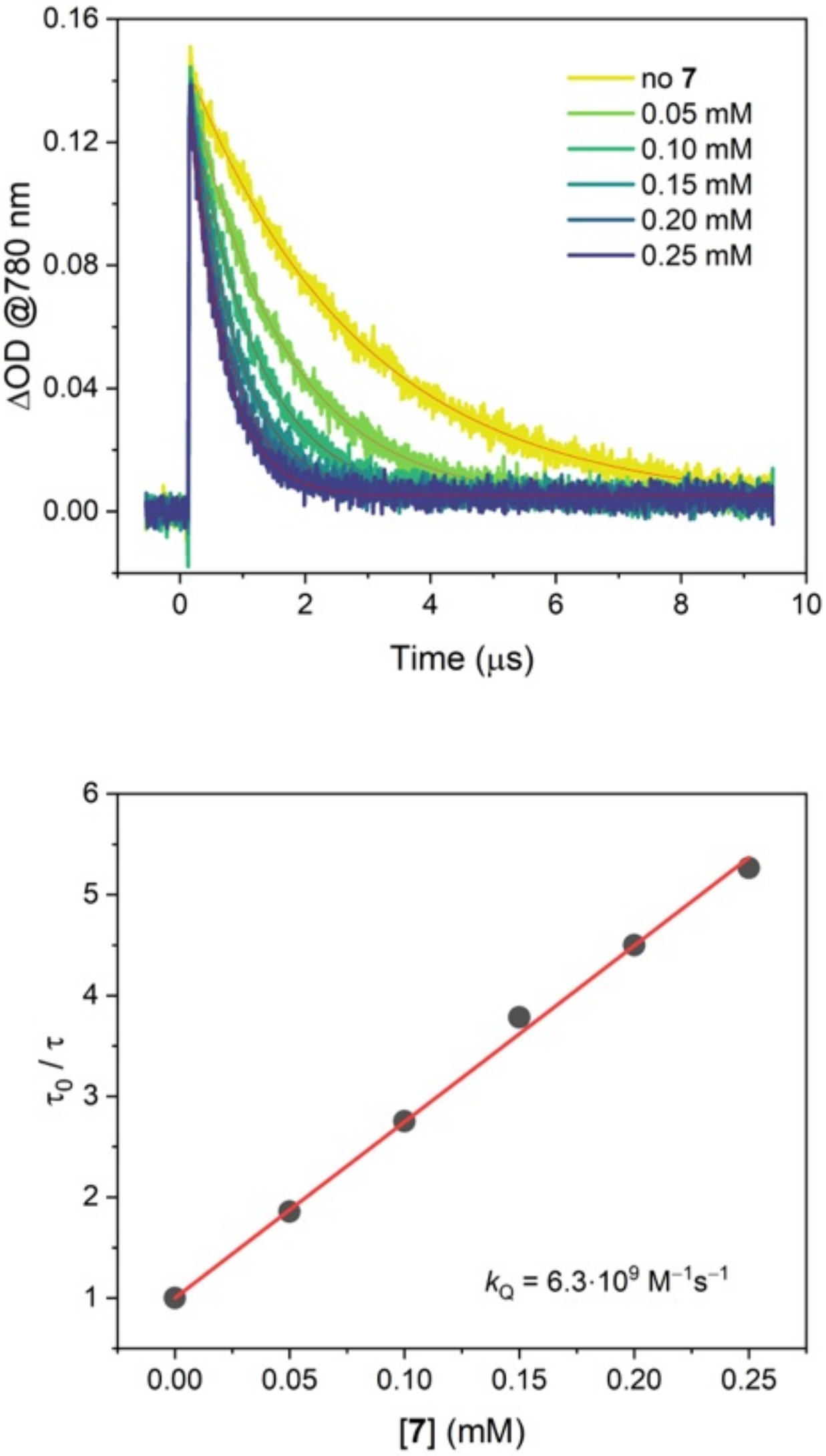

Figure S86. Kinetic traces at 780 nm, associated with the triplet decay of **[4a] BF$_4$,** measured by laser flash photolysis (excitation at 532 nm) of **[4a] BF$_4$** in N_2-purged CH_3NO_2 in the presence of 0-0.25 mM **7** (top) and corresponding Stern-Volmer plot (bottom).

C(sp²)–H amination

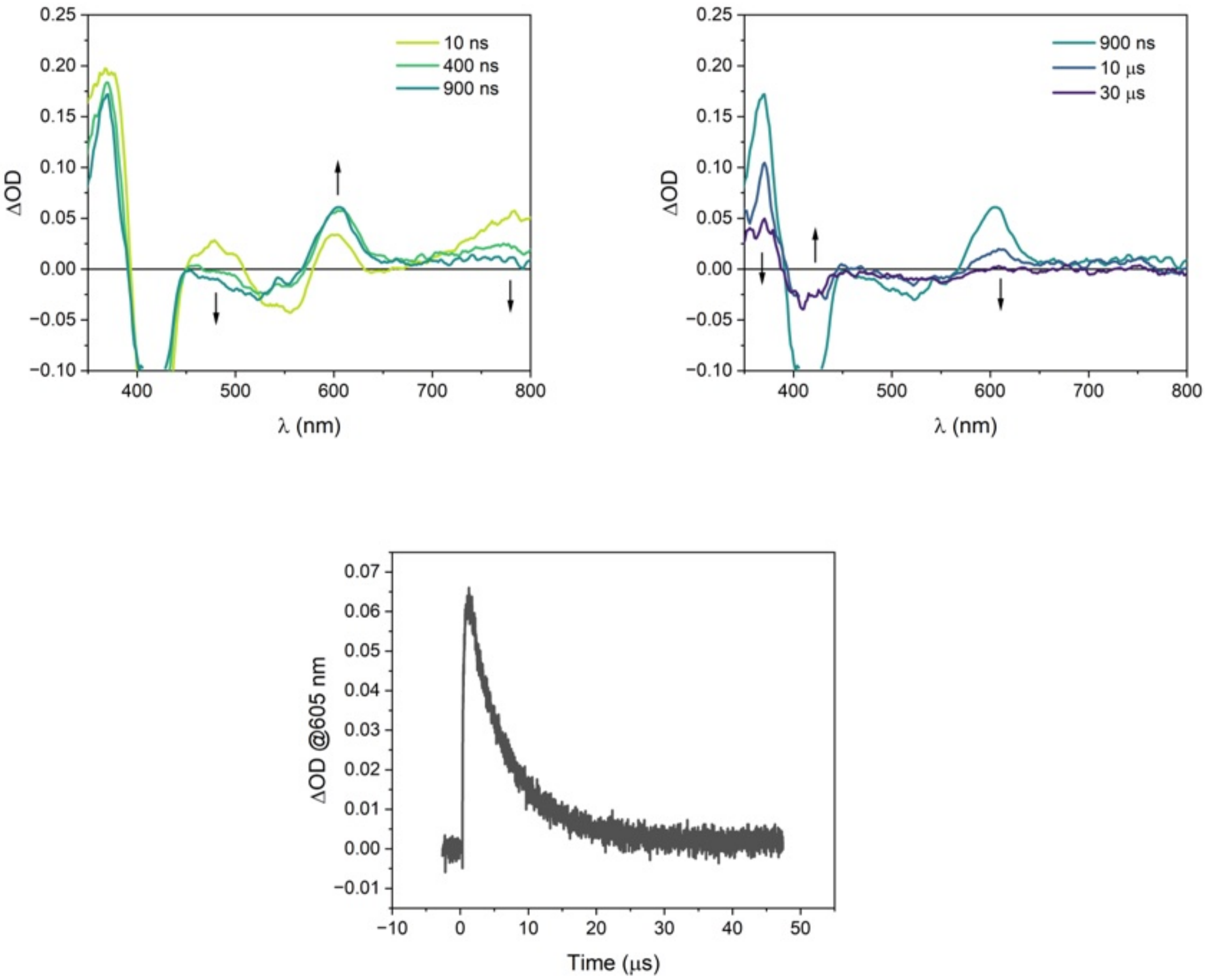

Figure S87. Transient absorption spectra at different time delays (gate width = 50 ns) obtained by laser flash photolysis (excitation at 532 nm) of **[4a] BF₄** in N_2-purged CH_2Cl_2 in the presence of 0.01 M **10** (top) and kinetic trace at 605 nm (bottom).

*The spectral evolution of **[4a] BF₄** in the presence of 0.01 M **10** monitored by laser flash photolysis is qualitatively similar to that observed in the presence of trans-anethole 7 (see above) and is biphasic. The prompt spectrum is the fingerprint of the triplet LE state, still indicating effective triplet formation in the presence of the substrate. The first process (**Figure S87**,top left) occurs within ca 1 μs and shows the decrease of the characteristic absorption of the triplet LE state at 780 nm and the concomitant rise of an absorption at 605 nm assigned to the reduced photocatalyst; the second process occurs within the following 30 μs and features a decay of the latter transient signal to the baseline (**Figure S87**, top right). The first process can be assigned to the photoinduced electron transfer between the triplet LE state of **[4a] BF₄** and 1,3,5-trimethoxybenzene **10** leading to the reduced photocatalyst **[4a]**$^{•-}$ and the oxidized **10**$^{•+}$, while the second process to the resulting charge recombination. Stern-Volmer analysis performed on the triplet absorption signal at 780 nm (**Figure S88**) confirms bimolecular quenching of the triplet LE state by **10** which occurs with a rate constant of k = $2.5\cdot10^8$ $M^{-1}s^{-1}$.*

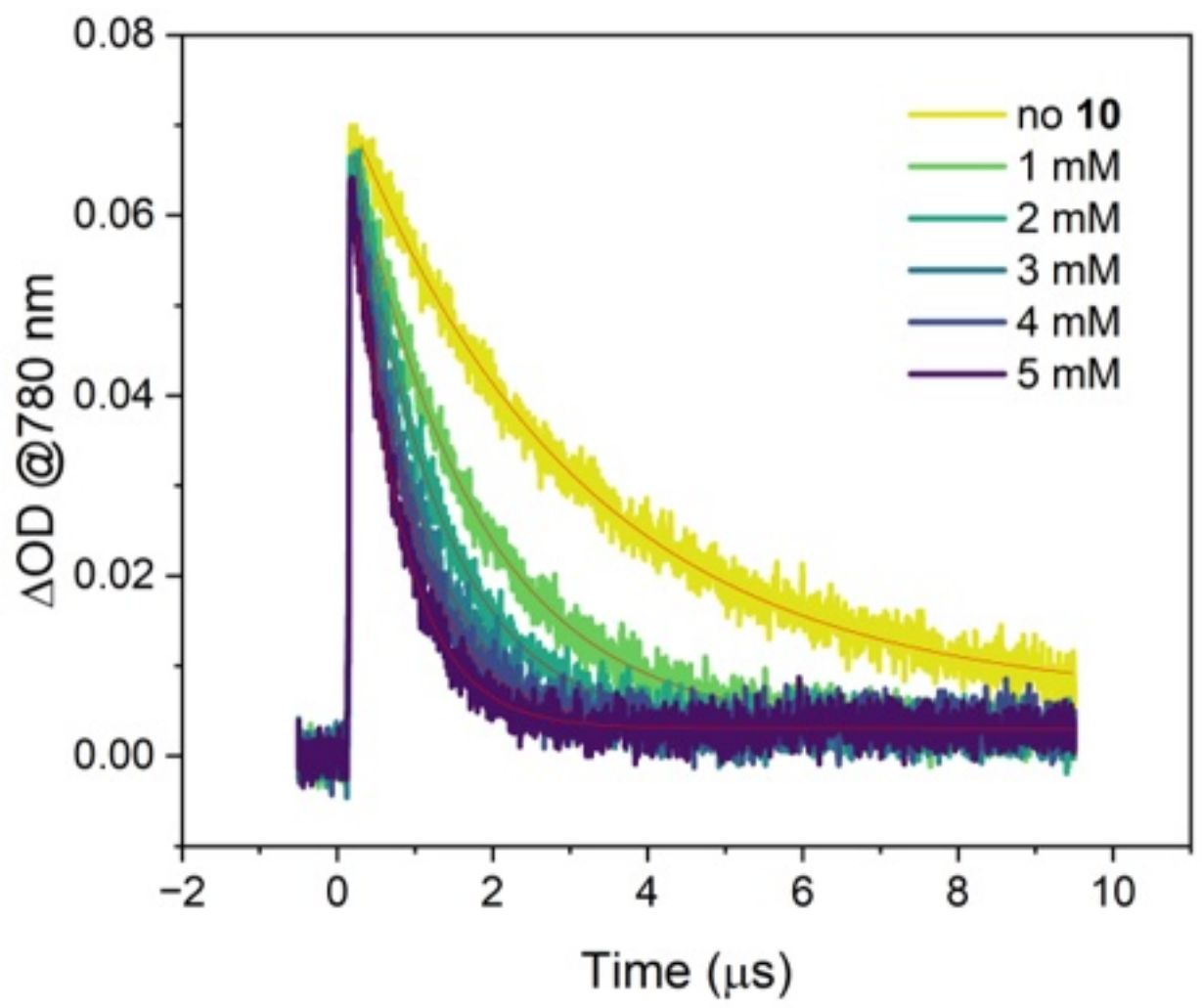

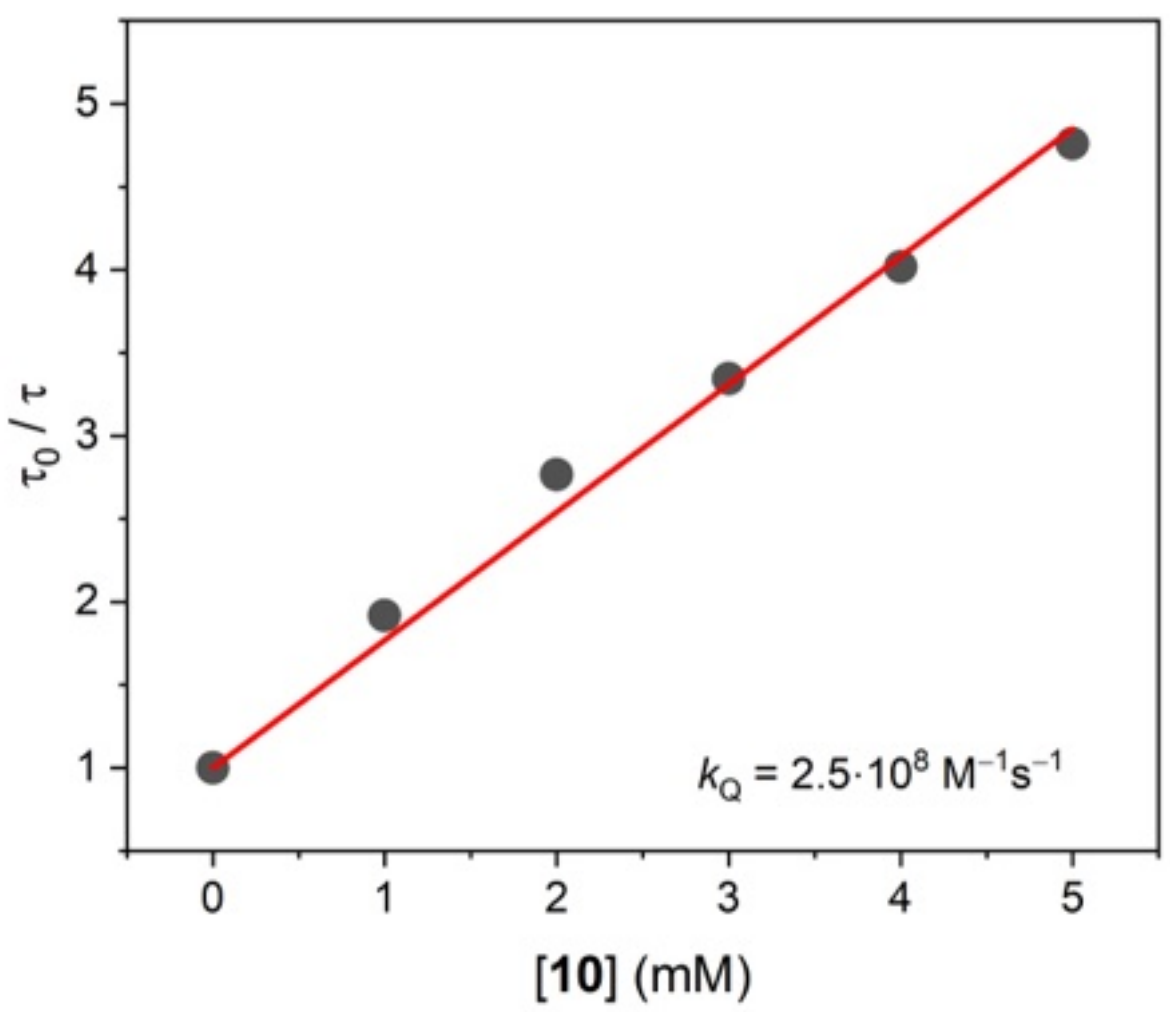

Figure S88. Kinetic traces at 780 nm, associated with the triplet decay of **[4a] BF$_4$,** measured by laser flash photolysis (excitation at 532 nm) of **[4a] BF$_4$** in N_2-purged CH_2Cl_2 in the presence of 0-5 mM **10** (top) and corresponding Stern-Volmer plot (bottom).

PHOTOCATALYST STABILITY

PHOTOCATALYST STABILITY IN THE GROUND STATE

Reaction of [2a] BF_4 and [4a] BF_4 with aniline

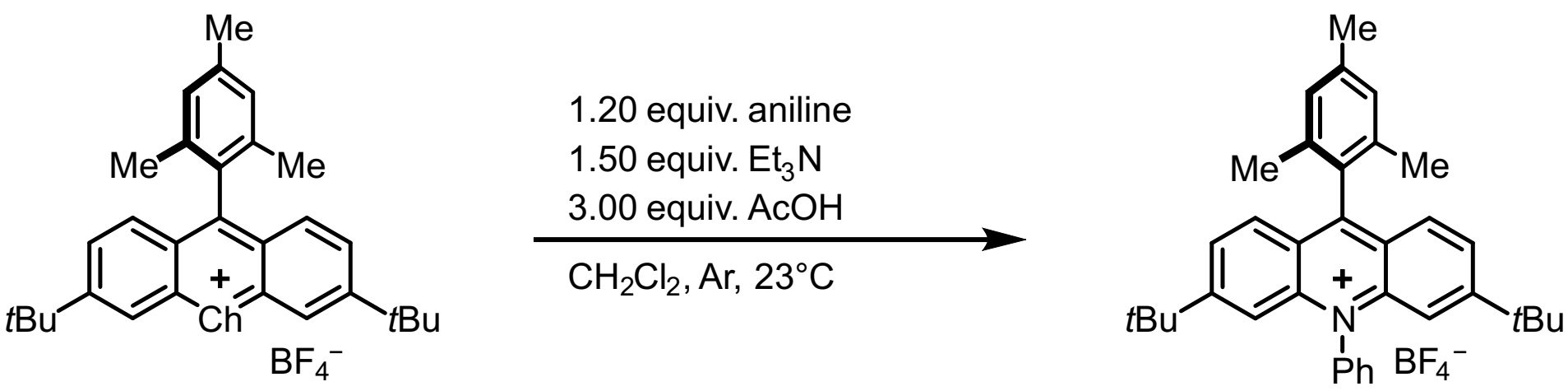

[2a] or [4a] BF_4 **[1a] BF_4**

The stability of xanthylium salts **[2a] BF_4** and **[4a] BF_4** in the ground state was tested in a condensation reaction with aniline.

In a J-Young NMR tube the xanthylium salt (0.05 mmol, 1.00 equiv.) was added. The tube was degassed and refilled with argon three times. Then, dry and Ar-equilibrated CH_2Cl_2 (0.50 mL) was added using a 1.0-mL syringe under an argon shower. The tube was caped, and the mixture was analyzed by ^1H NMR spectroscopy (**Figures S90**, **S93**). After the initial NMR analysis, Et_3N (10.0 µL, 0.075 mmol, 1.50 equiv.) and AcOH (8.6 µL, 0.15 mmol, 3.00 equiv.) were added via microsyringes to the NMR tube under an argon shower. The tube was caped, inverted three times, and the mixture was analyzed by ^1H NMR spectroscopy (**Figures S91**, **S94**). After a second initial point, aniline (5.6 µL, 0.06 mmol, 1.2 equiv.) was added to the mixture, and the tube was quickly caped and shaken. Continuous NMR measurements (162 NMR experiments with 8 scans per analysis and 5 min intervals between the measurements) (**Figures S92**, **S95**).

^{1}H NMR spectrum of acridinium [1a] BF_4 in CD_2Cl_2

The spectrum of **[1a] BF₄** (product of the reaction) in CD_2Cl_2 is shown in **Figure S89**. The singlet signal with the chemical shift of 7.42 ppm (2H) was selected for the reaction monitoring by ^{1}H NMR analysis.

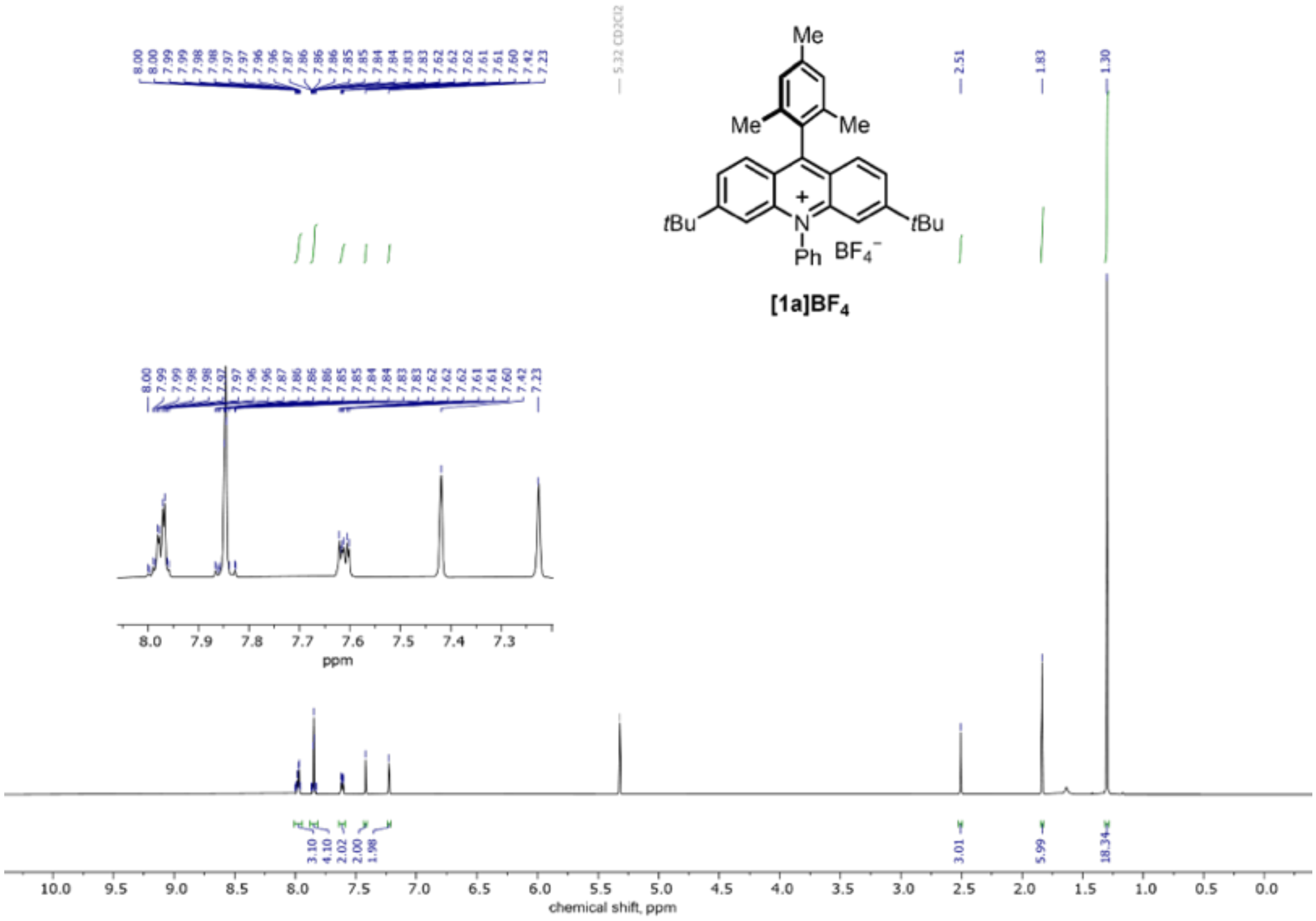

Figure S89. ^{1}H NMR spectrum of **[1a] BF₄**: (500 MHz, CD_2Cl_2, 23 °C, δ): 8.00 – 7.96 (m, 3H), 7.87 – 7.83 (m, 4H), 7.62 – 7.60 (m, 2H) 7.42 (s, 2H), 7.23 (s, 2H), 2.51 (s, 3H), 1.83 (s, 6H), 1.30 (s, 18H).

^{1}H NMR spectra of xanthylium [2a] BF_4 and reaction with aniline

The spectrum of the xanthylium salt **[2a] BF_4** in CD_2Cl_2 before the condensation reaction is shown in **Figure S90**. To calculate the conversion of **[2a] BF_4** to **[1a] BF_4** during the reaction course xanthylium salt signal (s, 7.21 ppm, 2H) and non-deuterated solvent signal (t, 5.32 ppm, 2H) were used.

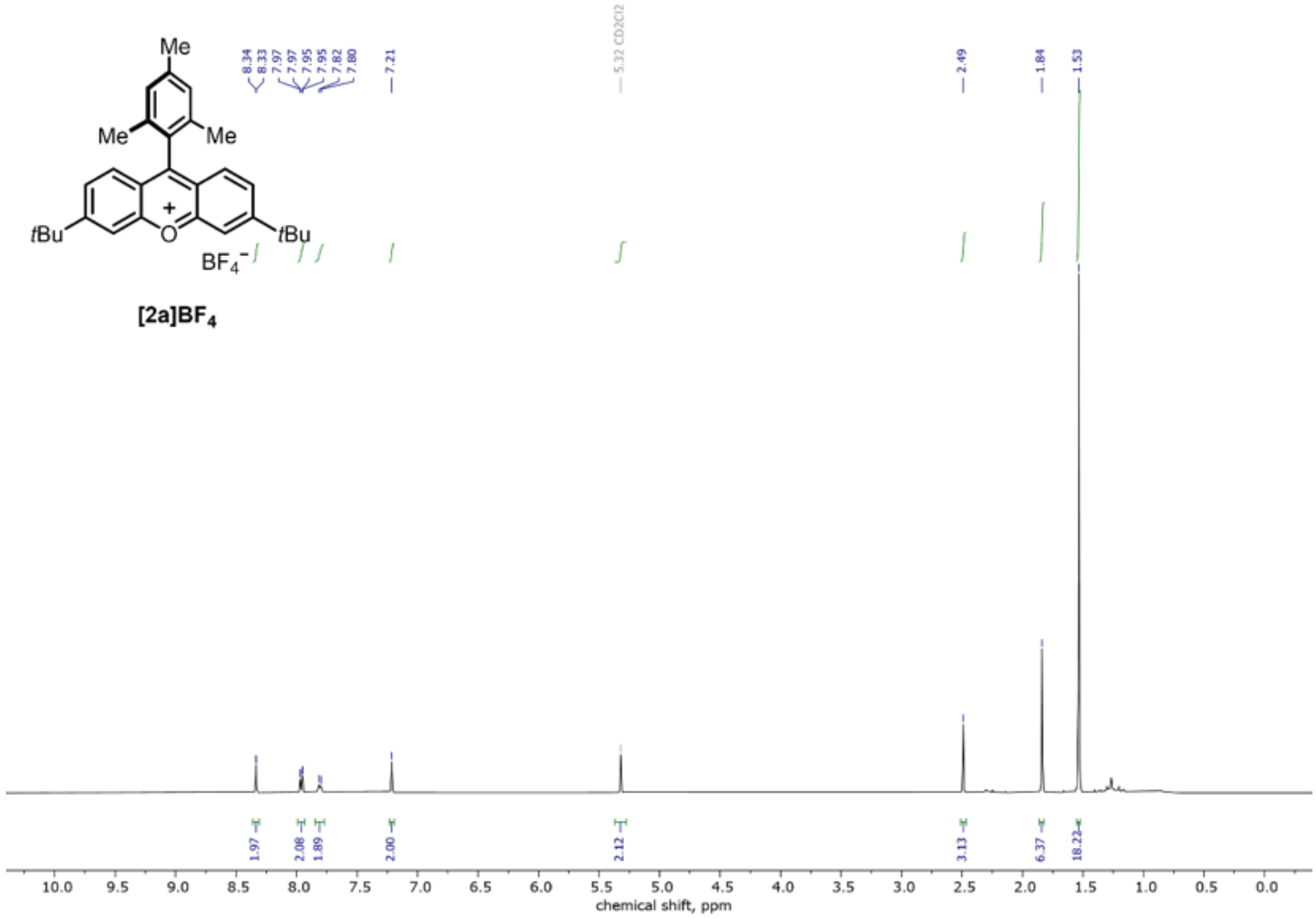

Figure S90. ^{1}H NMR spectrum of **[2a] BF_4** before the reaction with aniline: (500 MHz, CD_2Cl_2, 23 °C, δ): 8.33 (d, J = 1.8 Hz, 2H), 7.96 (dd, J = 8.9, 1.8 Hz, 2H), 7.81 (d, J = 8.9 Hz, 2H), 7.21 (s, 2H), 2.49 (s, 3H), 1.84 (s, 6H), 1.53 (s, 18H).

The reaction mixture after addition of Et_3N and AcOH is shown in **Figure S91**.

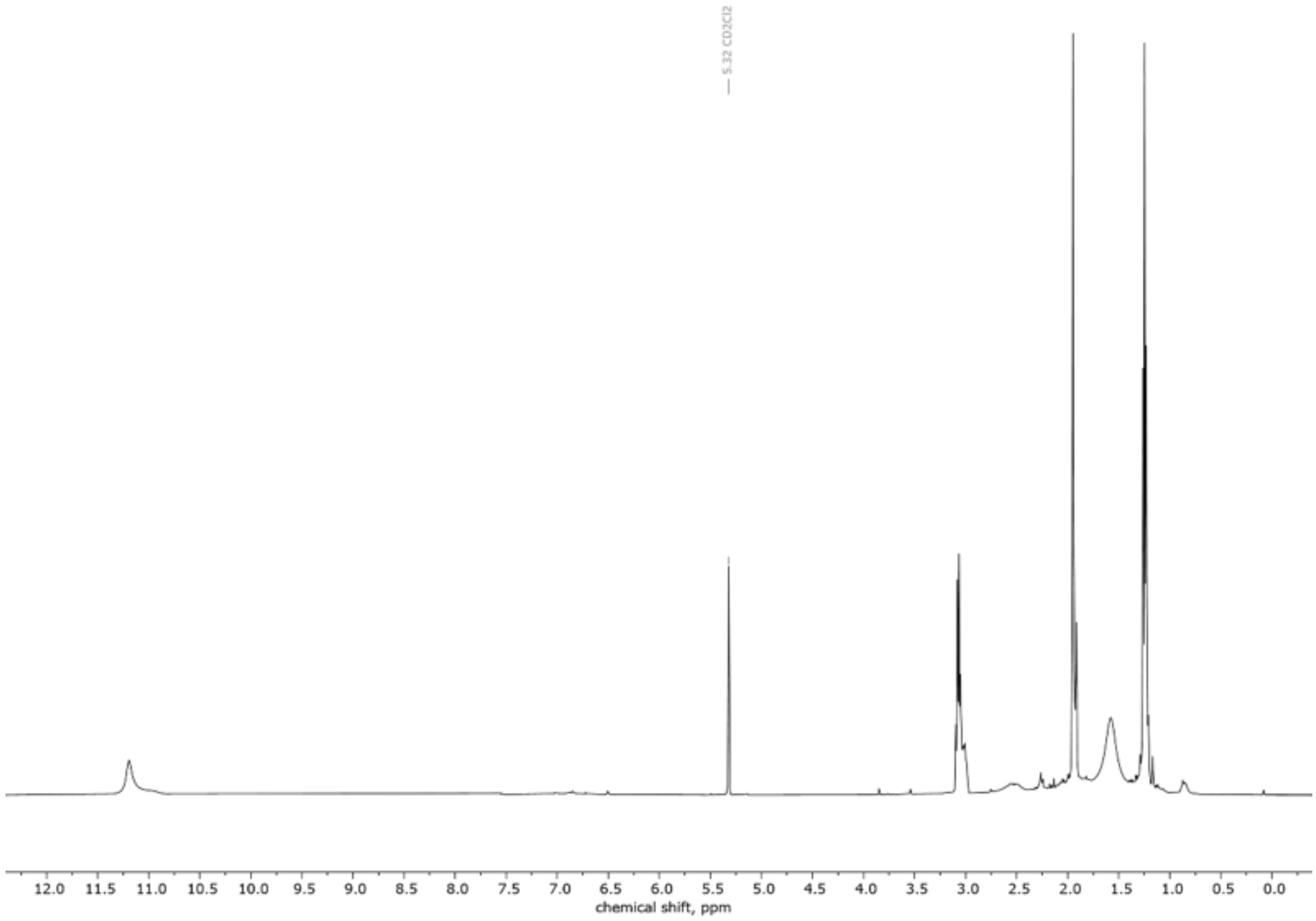

Figure S91. ^1H NMR spectrum of **[2a] BF$_4$**, Et_3N, and AcOH in CD_2Cl_2 before the reaction with aniline.

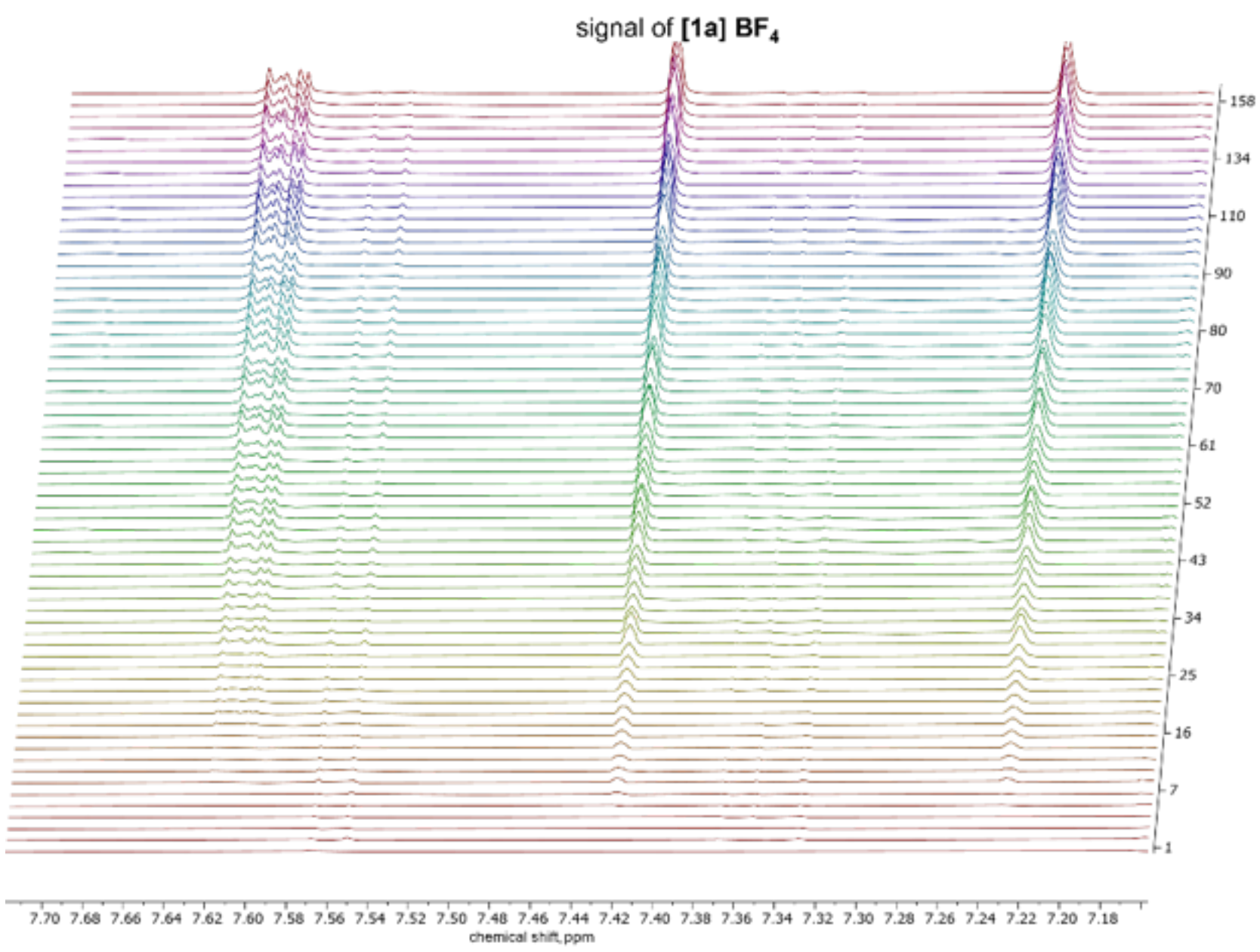

Figure S92. ^1H NMR spectra of the first 16 h of condensation reaction between **[2a] BF$_4$** and aniline.

Figure S92 highlights the region between 7.18 – 7.70 ppm of the ^1H NMR spectra of the reaction mixture between **[2a] BF$_4$** and aniline. The increased signal at 7.42 ppm (**[1a] BF$_4$**) highlight the catalyst deactivation during the first 16 h of the reaction.

^{1}H NMR spectra of selenoxanthylium [4a] BF_4 and reaction with aniline

The spectrum of the selenoxanthylium salt **[4a] BF4** in CD_2Cl_2 before the condensation reaction is shown in **Figure S93**. To calculate the conversion of **[4a] BF4** to **[1a] BF4** during the reaction course xanthylium salt signal (s, 7.19 ppm, 2H) and non-deuterated solvent signal (t, 5.32 ppm, 2H) were used.

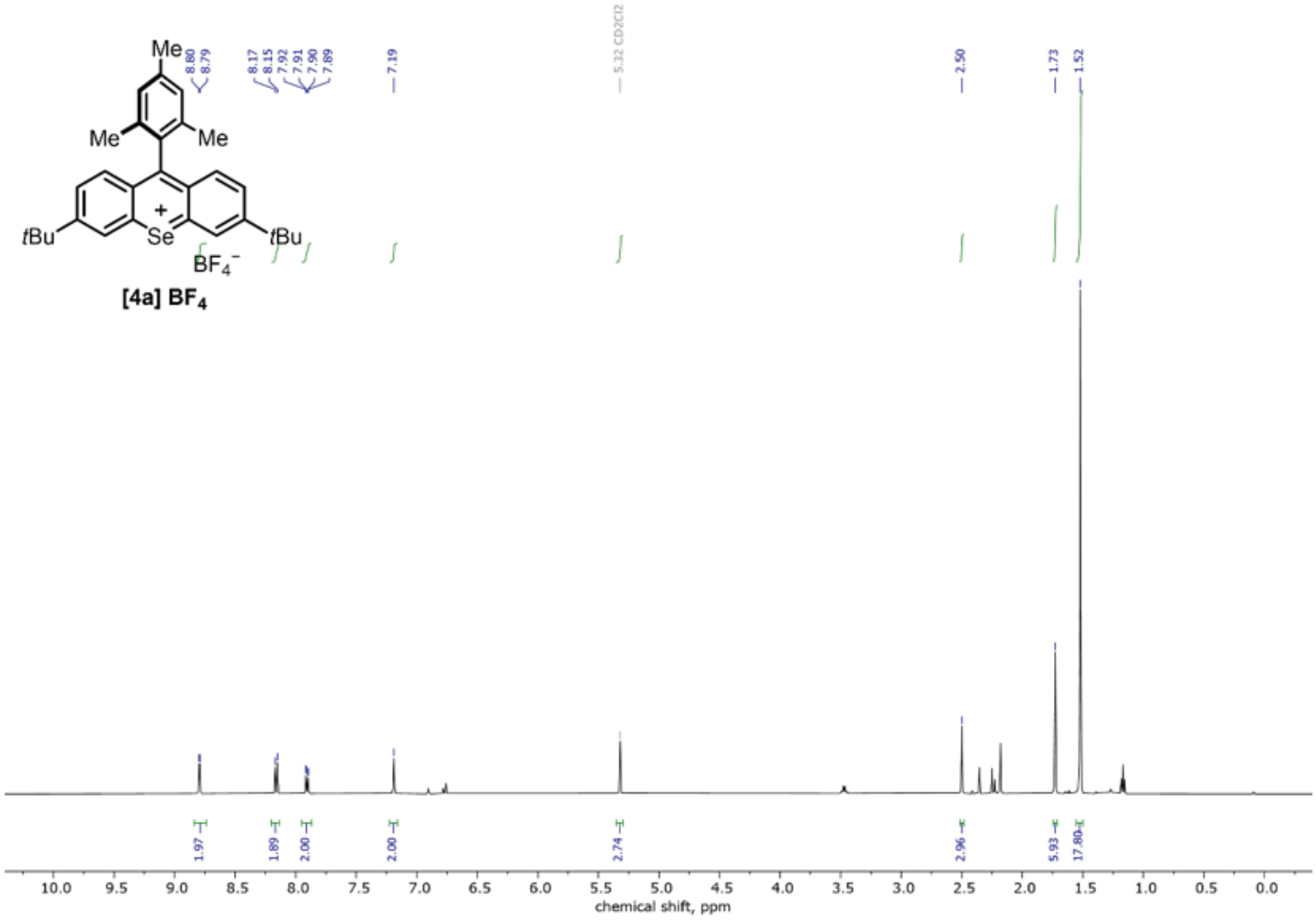

Figure S93. ^{1}H NMR spectrum of **[4a] BF**$_4$ before the reaction with aniline: (500 MHz, CD_2Cl_2, 23 °C, δ) 8.79 (d, *J* = 1.9 Hz, 2H), 8.16 (d, *J* = 9.2 Hz, 2H), 7.91 (dd, *J* = 9.2, 1.9 Hz, 2H), 7.19 (s, 2H), 2.50 (s, 3H), 1.73 (s, 6H), 1.52 (s, 18H).

The reaction mixture after addition of Et_3N and AcOH is shown in **Figure S94**.

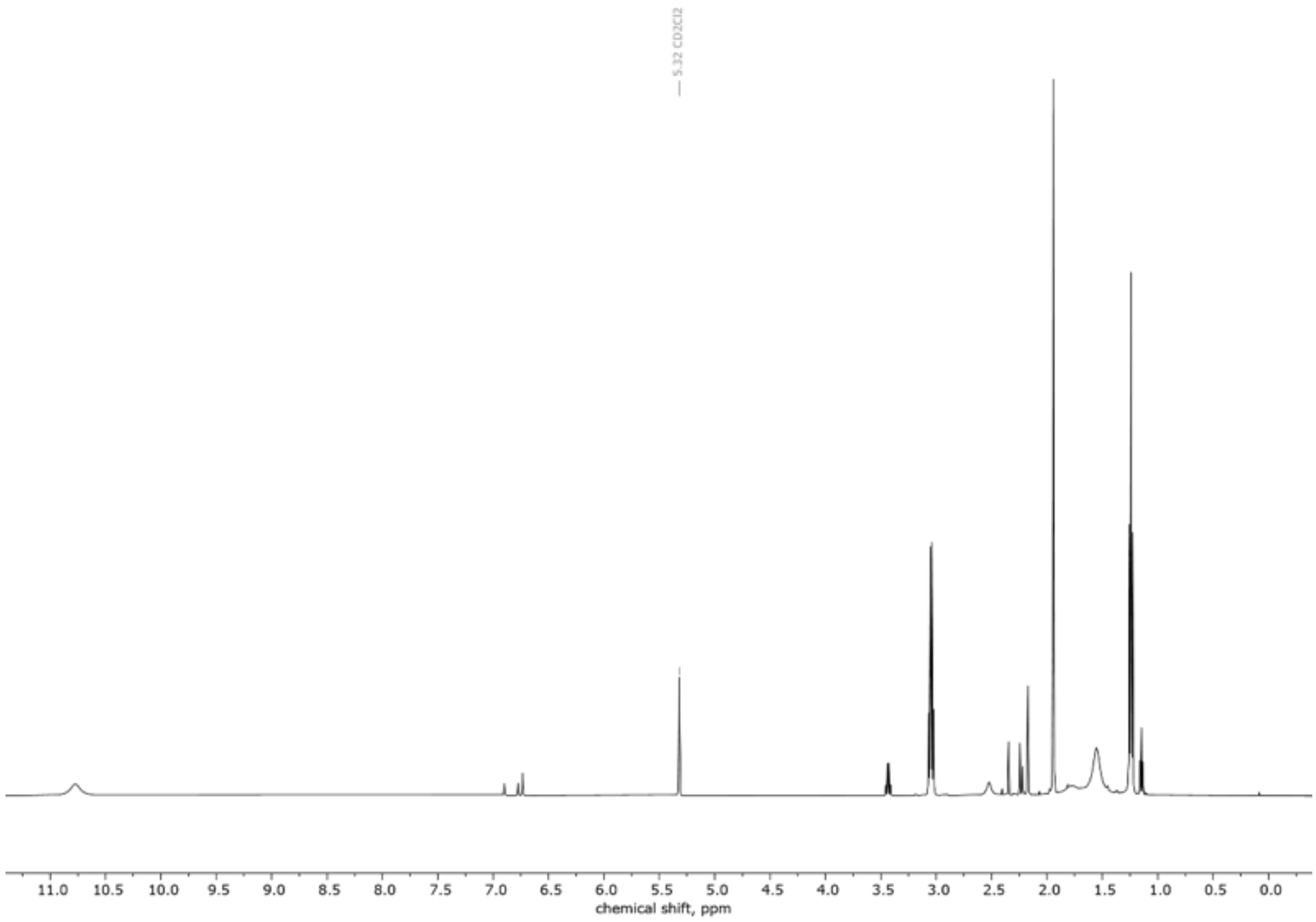

Figure S94. ^{1}H NMR spectrum of **[4a] BF₄**, Et_3N, and AcOH in CD_2Cl_2 before the reaction with aniline.

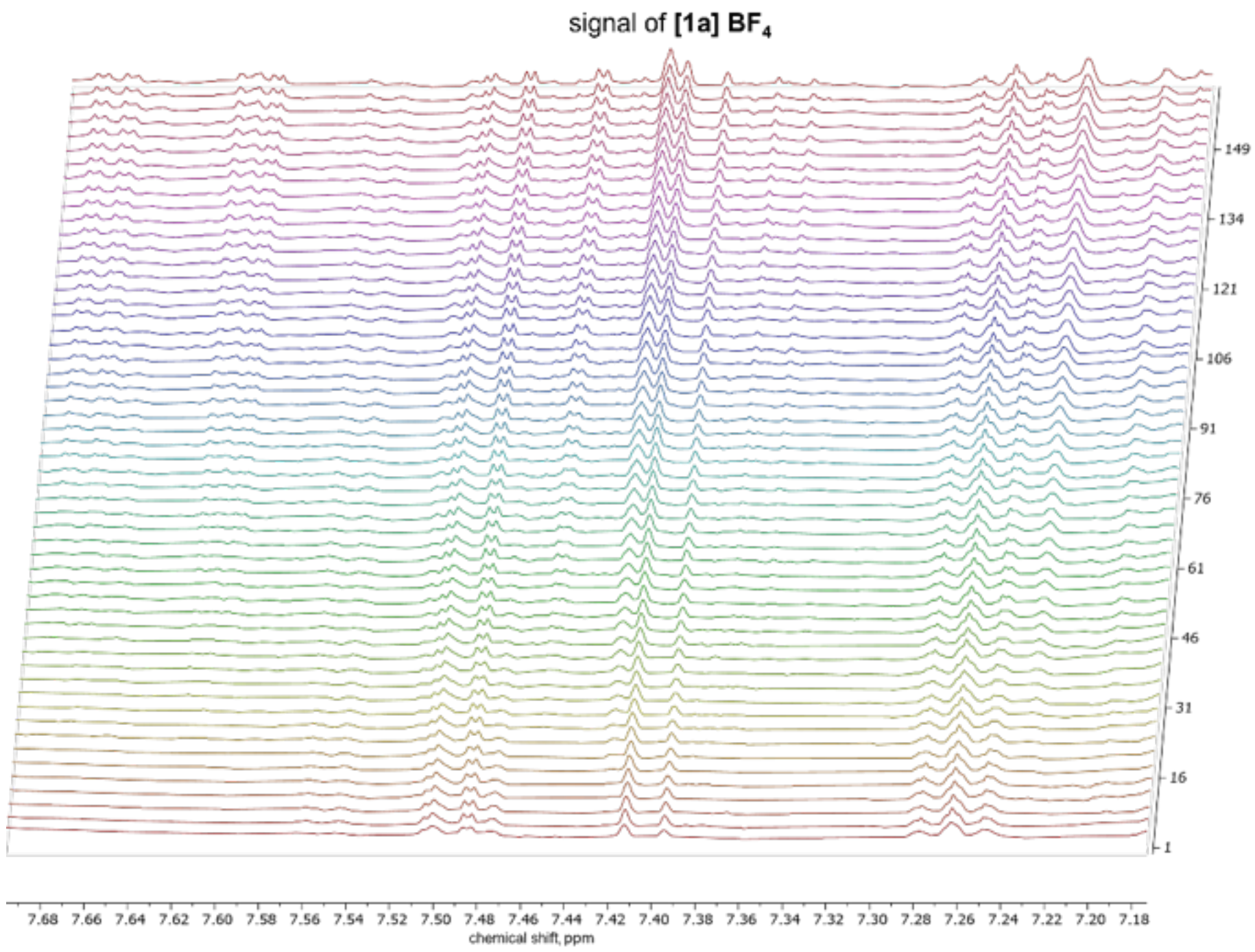

Figure S95. ^{1}H NMR analysis of the condensation reaction between **[4a] BF_4** and aniline.

Figure S95 highlights the region between (7.18 – 7.68 ppm) of the ^{1}H NMR spectra of the reaction mixture between **[4a] BF_4** and aniline over time (16 hours).

Conversion of xanthylium salts to [1a] BF_4

As signals of starting material can't be identified in NMR spectra of the reaction mixture after addition of Et_3N and AcOH (**Figures S91**, **S94**), the conversion of xanthylium salts **[2a] BF_4** and **[4a] BF_4** was estimated by comparing the integrals of characteristic signals of the starting material and product, normalized to the integral of the non-deuterated solvent signal, which was assumed to remain constant through the reaction:

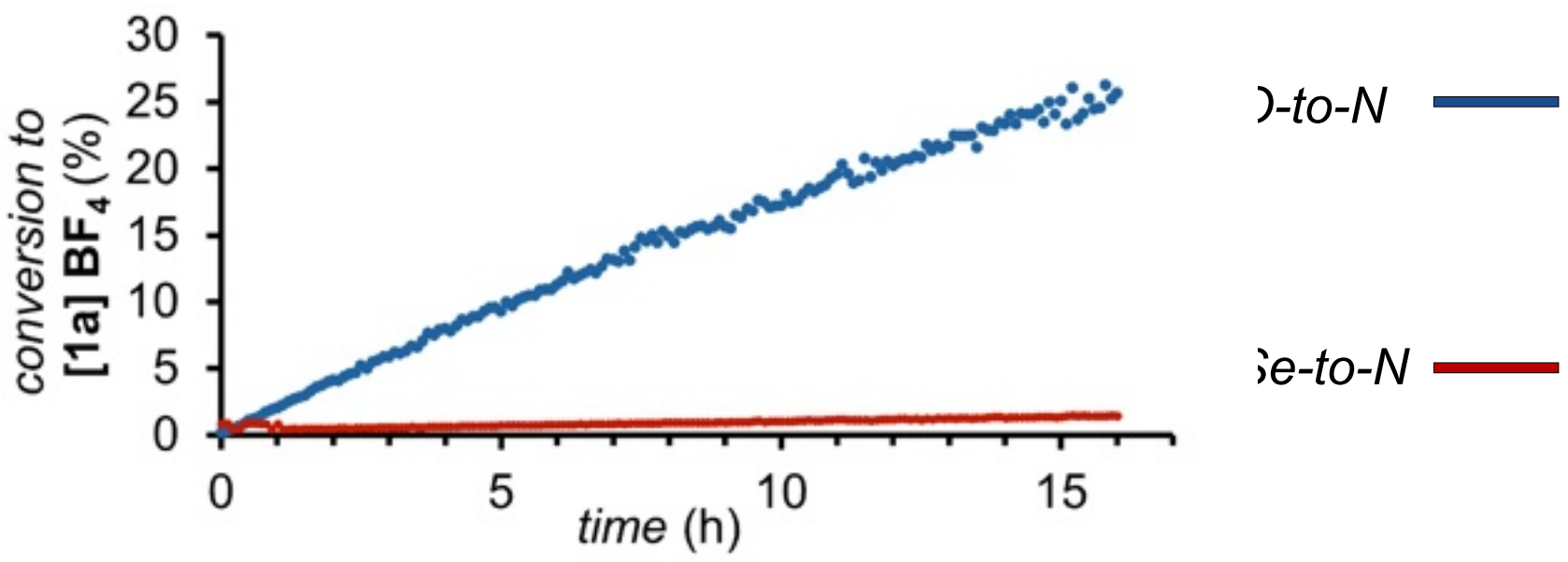

Figure S96. Conversion to **[1a] BF_4**. Comparison between **[2a] BF_4** and **[4a] BF_4** in the presence of aniline as nucleophile.

After 96 h the reaction mixtures were analyzed by ^{1}H NMR spectroscopy. Conversion of **[2a] BF_4** was found 64% while conversion of **[4a] BF_4** was <5% (**Figures S97, S98**).

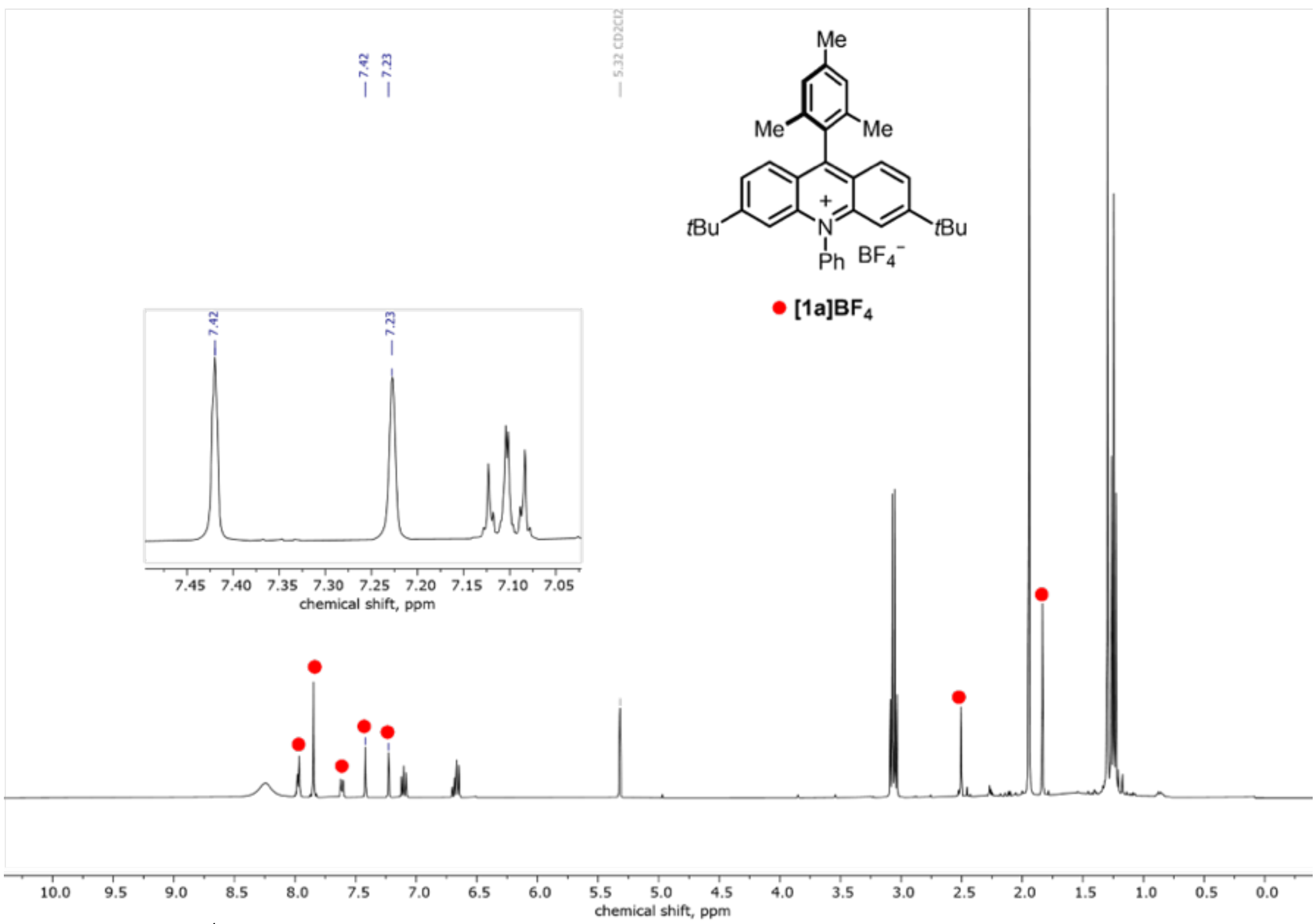

Figure S97.^{1}H NMR spectrum of **[2a] BF_4** reaction with aniline after 96 h.

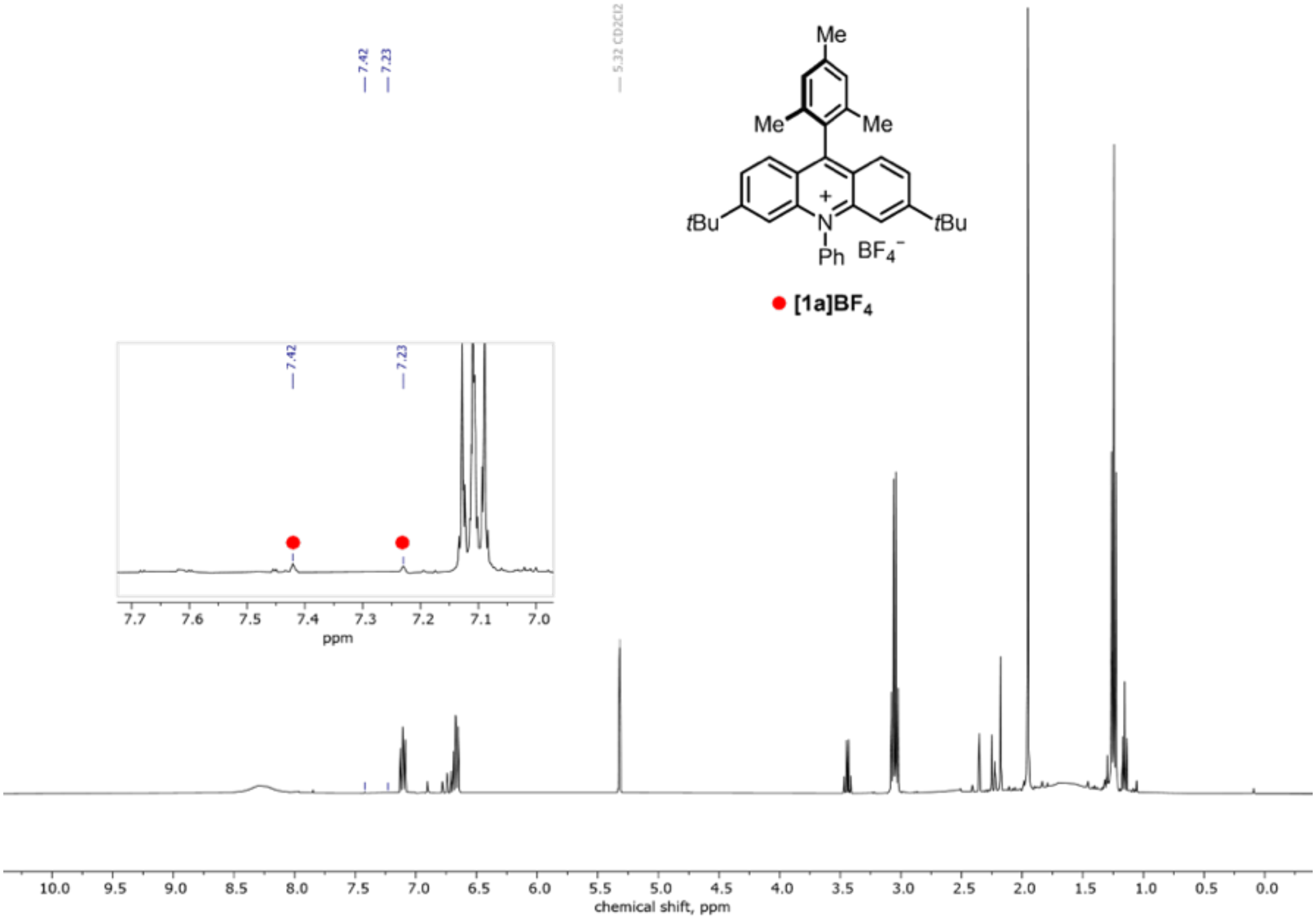

Figure S98. ^{1}H NMR spectrum of **[4a] BF₄** reaction with aniline after 96 h.

PHOTOCATALYST STABILITY UNDER REACTION CONDITIONS

[4+2]-Cycloaddition reaction catalyzed by selenoxanthylium salts at 1.00 mmol scale

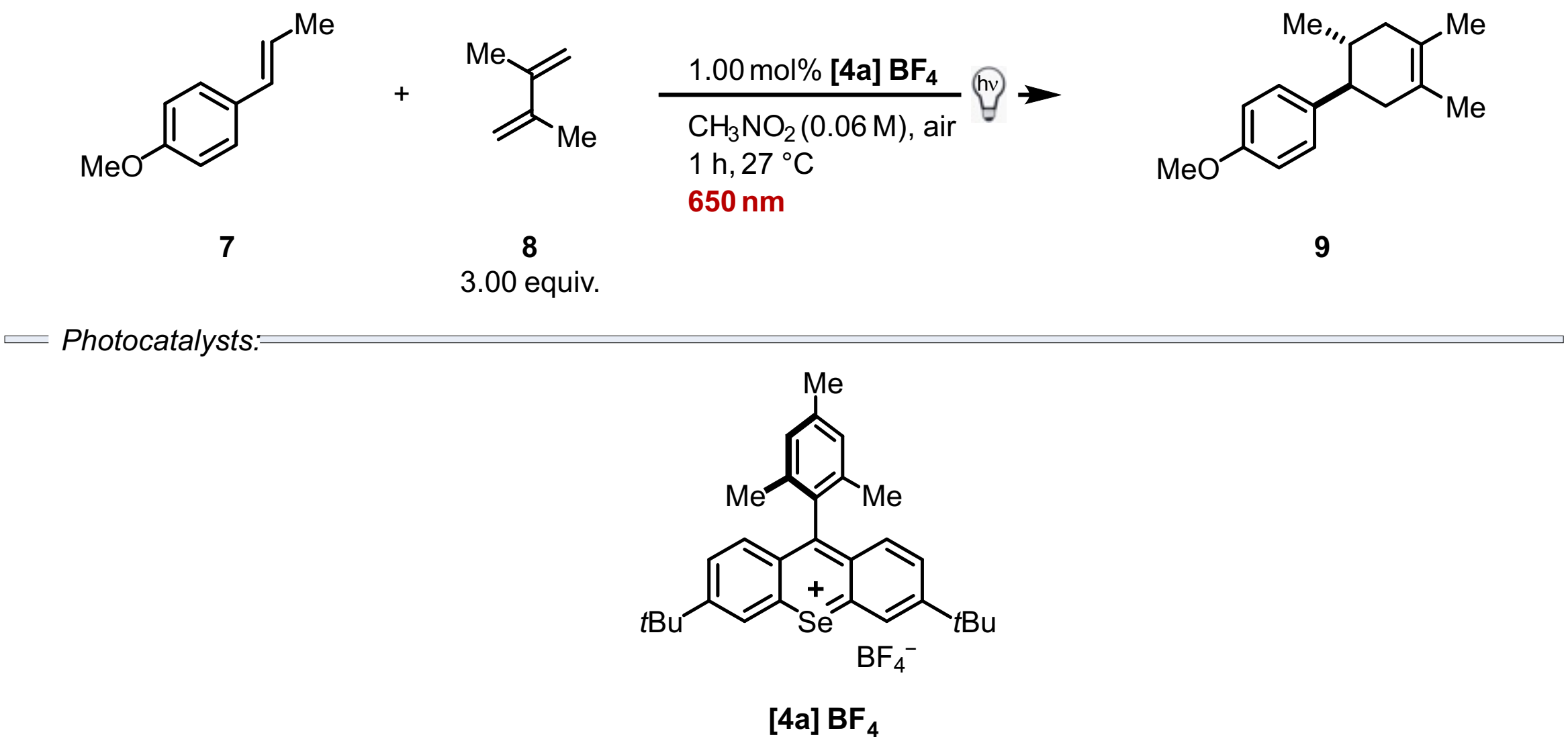

5.8 mg **[4a] BF$_4$** isolated
No decomposition observed

Procedure for the [4+2]-cycloaddition reaction at a 1.00 mmol scale

Under an ambient atmosphere, to a 50-mL round-bottom flask equipped with a magnetic stir bar, trans-anethol **7** (148 mg, 150 µL, 1.00 equiv., 1.00 mmol), photocatalyst **[4a] BF_4** (5.6 mg, 1.0 mol%, 10.0 µmol) and CH_3NO_2 (16.0 mL) were added. Then butadiene **8** (350 µL, 3.00 equiv., 3.00 mmol) was added dropwise via 1.00 mL syringe to the stirring mixture. The resulting solution was irradiated with red-light (red-light irradiation, λ_{max} = 650 nm) while stirred. The reaction was carried out at 27 °C for 1 h. After stirring the reaction for the given time, the reaction mixture was concentrated by rotary evaporation under reduced pressure. The photocatalyst **[4a] BF_4** was purified by washing with diethyl ether, affording **[4a] BF_4** 5.8 mg as a red solid.

^{1}H NMR of [4a] BF_4 after after [4+2]-cycloaddition reaction in $CDCl_3$

The spectrum of the selenoxanthylium salt **[4a] BF_4** in $CDCl_3$ after [4+2]-cycloaddition reaction is shown in **Figure S99**. After the reaction, 5.8 mg of **[4a] BF_4** was recovered following washing crude reaction with diethyl ether, which is slightly higher than the initial amount used in the reaction (5.6 mg) due to trace amounts of residual diethyl ether remaining in the sample. Analysis of the NMR spectrum confirmed that the **[4a] BF_4** was preserved throughout the reaction and showed no evidence of decomposition.

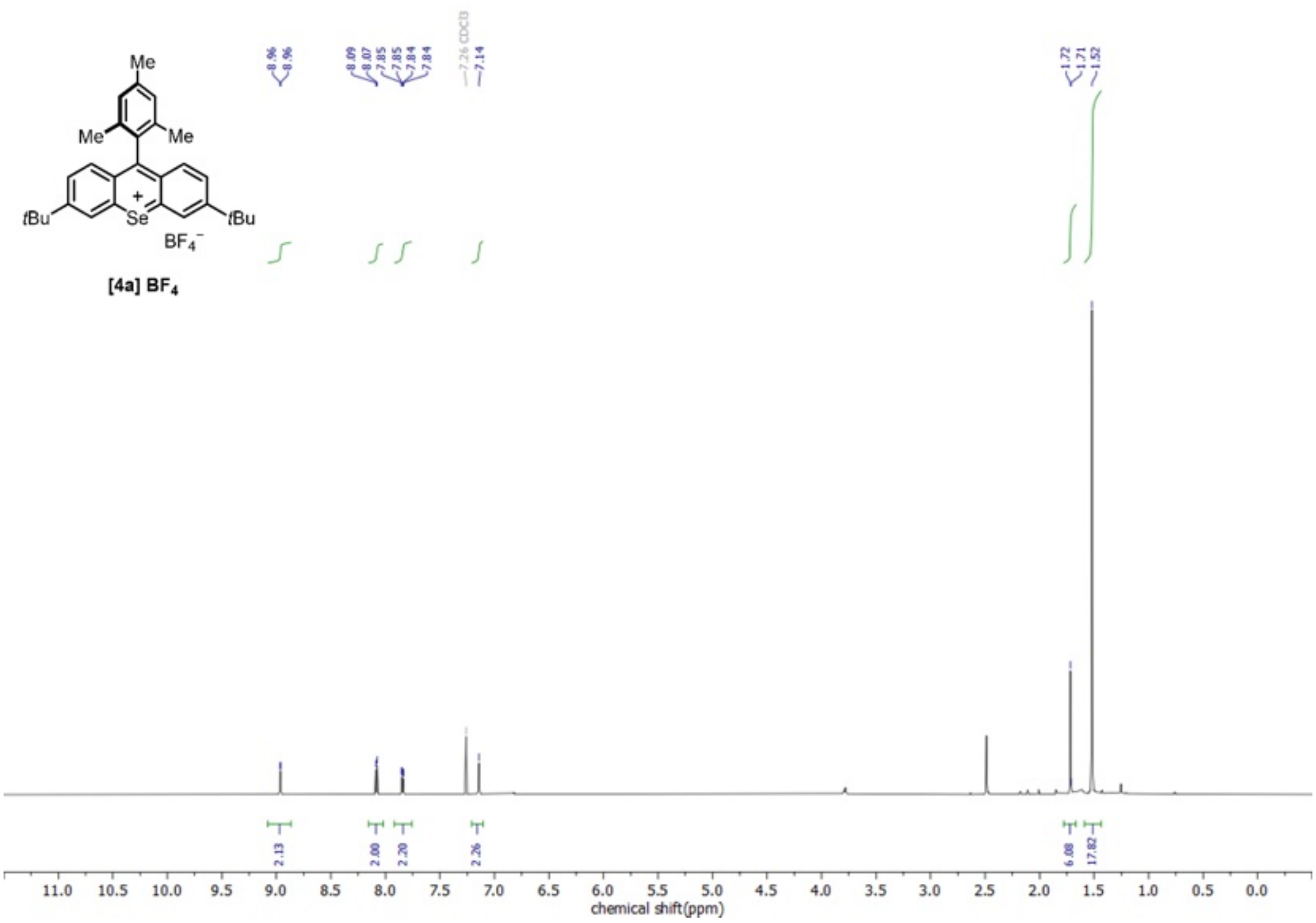

Figure S99. ^{1}H NMR spectrum of **[4a] BF_4** after [4+2]-cycloaddition reaction: (700 MHz, $CDCl_3$, 23 °C, δ) 8.96 (d, J = 1.9 Hz, 2H), 8.08 (d, J = 9.1 Hz, 2H), 7.84 (dd, J = 9.1, 1.9 Hz, 2H), 7.14 (s, 2H), 1.72 (s, 6H), 1.52 (s, 18H).

Desulfinylative trifluoromethylation reaction catalyzed by selenoxanthylium salt at 0.25 mmol scale

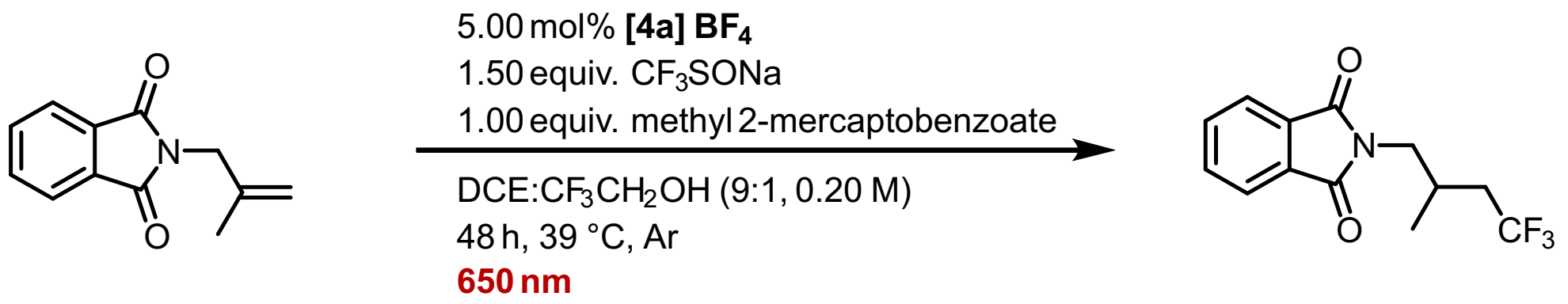

19 **20**

Photocatalysts:

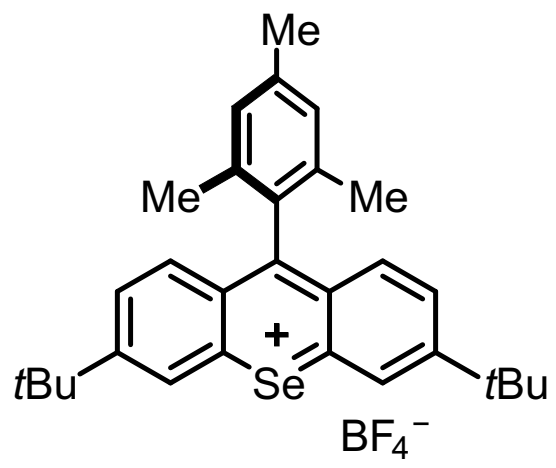

[4a] BF$_4$

No **[4a] BF$_4$** was recovered or decomposition product could be isolated

Procedure for the desulfinylative trifluoromethylation reaction at a 0.25 mmol scale

To a 4-mL borosilicate vial equipped with a magnetic stir bar, 2-(2-methylallyl)isoindoline-1,3-dione (**19**) (20.5 mg, 1.00 equiv., 0.10 mmol), sodium trifluoromethanesulfinate (1.5-2.5 equiv.), and photocatalyst **[4a] BF$_4$** were added. The vial was sealed with a membrane cap, connected via a needle and a Tygon® tubing to a Schlenk line and evacuated and purged with argon three times. Solvent (0.45 mL), TFE (0.05 mL) were added via 1-mL syringes, and methyl 2-mercaptobenzoate (13.8 µL, 1.00 equiv., 0.10 mmol) was added via a microsyringe. The resulting solution was stirred in the photoreactor (red LED irradiation (λ max = 650 nm)) with or without cooling for the reaction time. After stirring, the mixture was concentrated by rotary evaporation under reduced pressure. After washing the crude product with diethyl ether, **[4a] BF$_4$** was not detected. This suggests that **[4a] BF$_4$** had decomposed during the reaction process.

Kinetic Studies of Photocatalytic Reactions

[4+2]-Cycloaddition reaction at 0.10 mmol scale

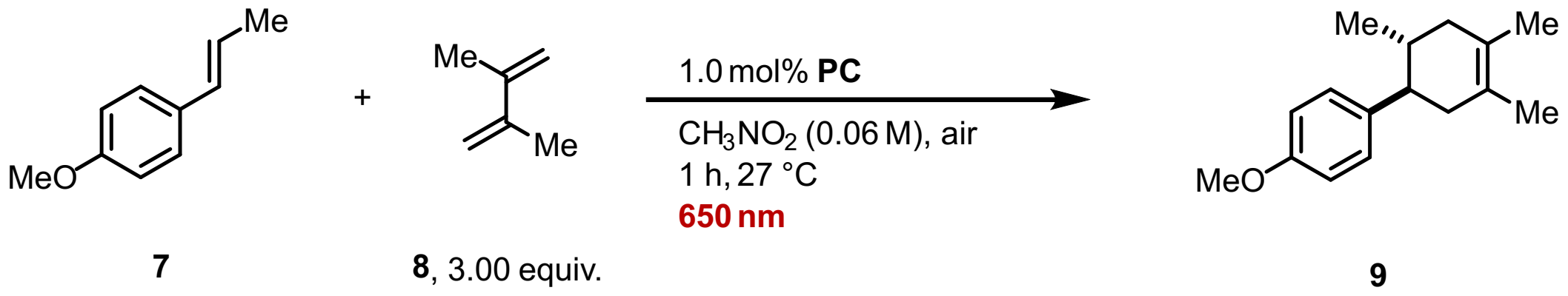

Tested photocatalysts:

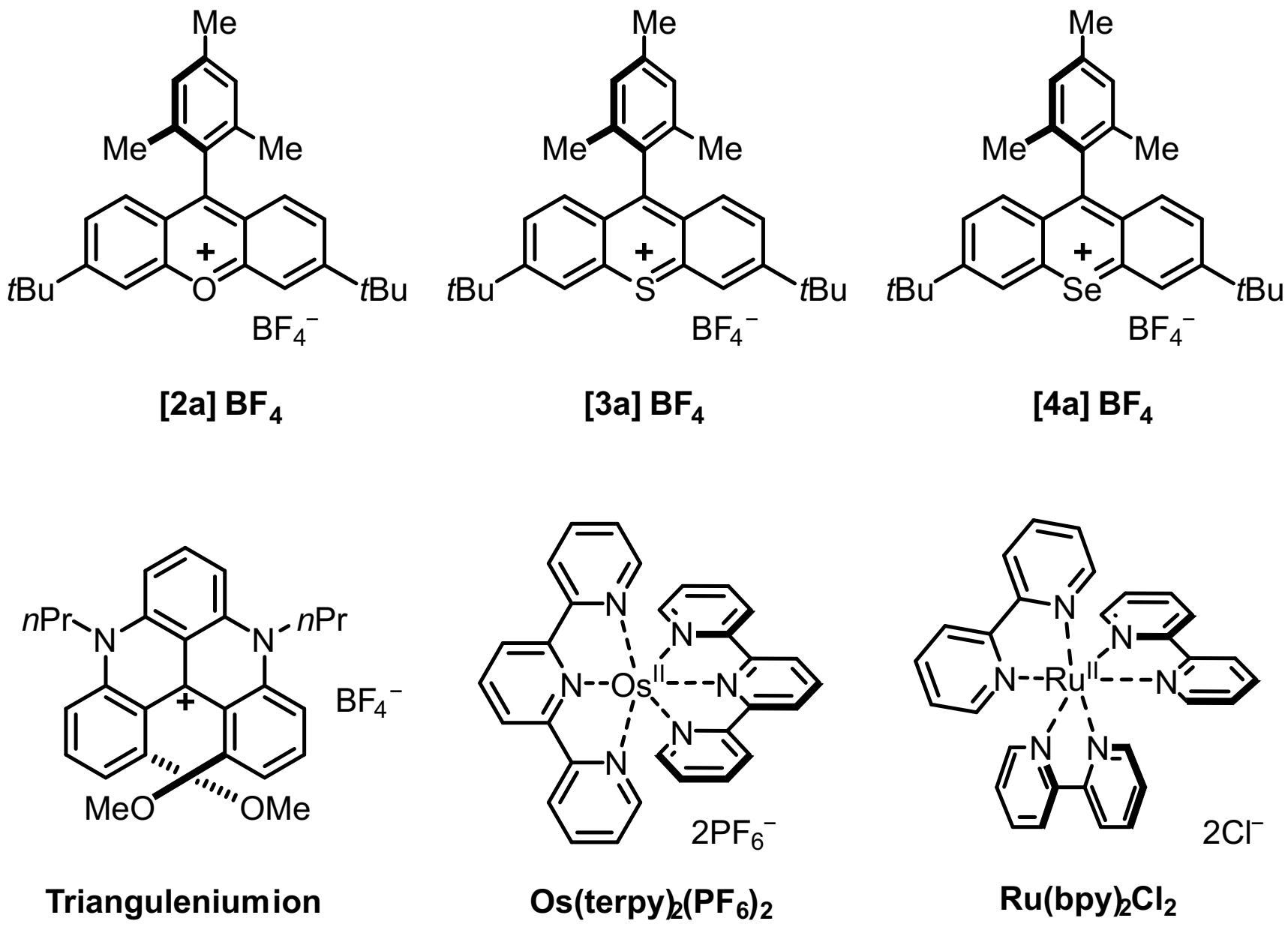

General procedure for the [4+2]-cycloaddition reaction at 0.10 mmol scale

Under an ambient atmosphere, to a 4-mL borosilicate scintillation vial equipped with a magnetic stir bar, trans-anethol **7** (14.8 mg, 15.0 µL, 1.00 equiv. 0.10 mmol), the photocatalyst (1.0 mol%) and CH_3NO_2 (1.6 mL) were added. Then butadiene **8** (35.0 µL, 3.00 equiv., 0.30 mmol) was added to the stirring mixture. The resulting solution was irradiated with the selected irradiation wavelength while stirred. The reaction was carried out at 27 °C for 1 h. After stirring the reaction for the given time, the reaction mixture was concentrated by rotary evaporation under reduced pressure. The residue was fully solubilized in $CDCl_3$ (0.50 mL) and transferred to a NMR tube. CH_2Br_2 (7.1 µL, 0.10 mmol = theoretical maximum yield) was added as internal standard. The yield was determined by ^{1}H NMR spectroscopy using CH_2Br_2 as internal standard by comparing the integration of the CH_2Br_2 signal at 4.93 ppm (s, 2H) to the signal of product **9** at 7.09 ppm (d, 2H).

Photocatalyst comparison tables

Table S14. [4+2]-Cycloaddition reaction using different xanthylium photocatalysts.

PC	**Without PC**	**[3a]BF$_4$**	**[4a]BF$_4$**	**[4a]BF$_4$**
Yield* (%)	n.r.	n.r.	7	93

* *The yield was determined by ^{1}H NMR spectroscopy using CH_2Br_2 as internal standard.*

Table S15. [4+2]-Cycloaddition reaction using different photocatalysts.

PC	**[4a]BF$_4$**	**Triangulenium ion**	**Os(terpy)$_2$(PF$_6$)$_2$**	**Ru(bpy)$_3$Cl$_2$**
Yield* (%)	93	8	13	12

* *The yield was determined by ^{1}H NMR spectroscopy using CH_2Br_2 as internal standard.*

C–H amination reaction at 0.10 mmol scale

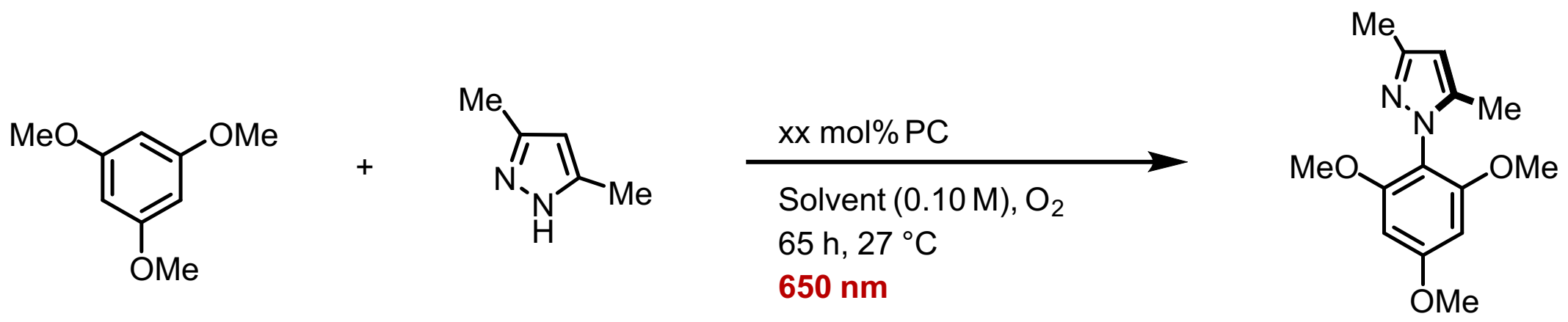

10 | **11**, 5.00 equiv. | **12**

Tested photocatalysts:

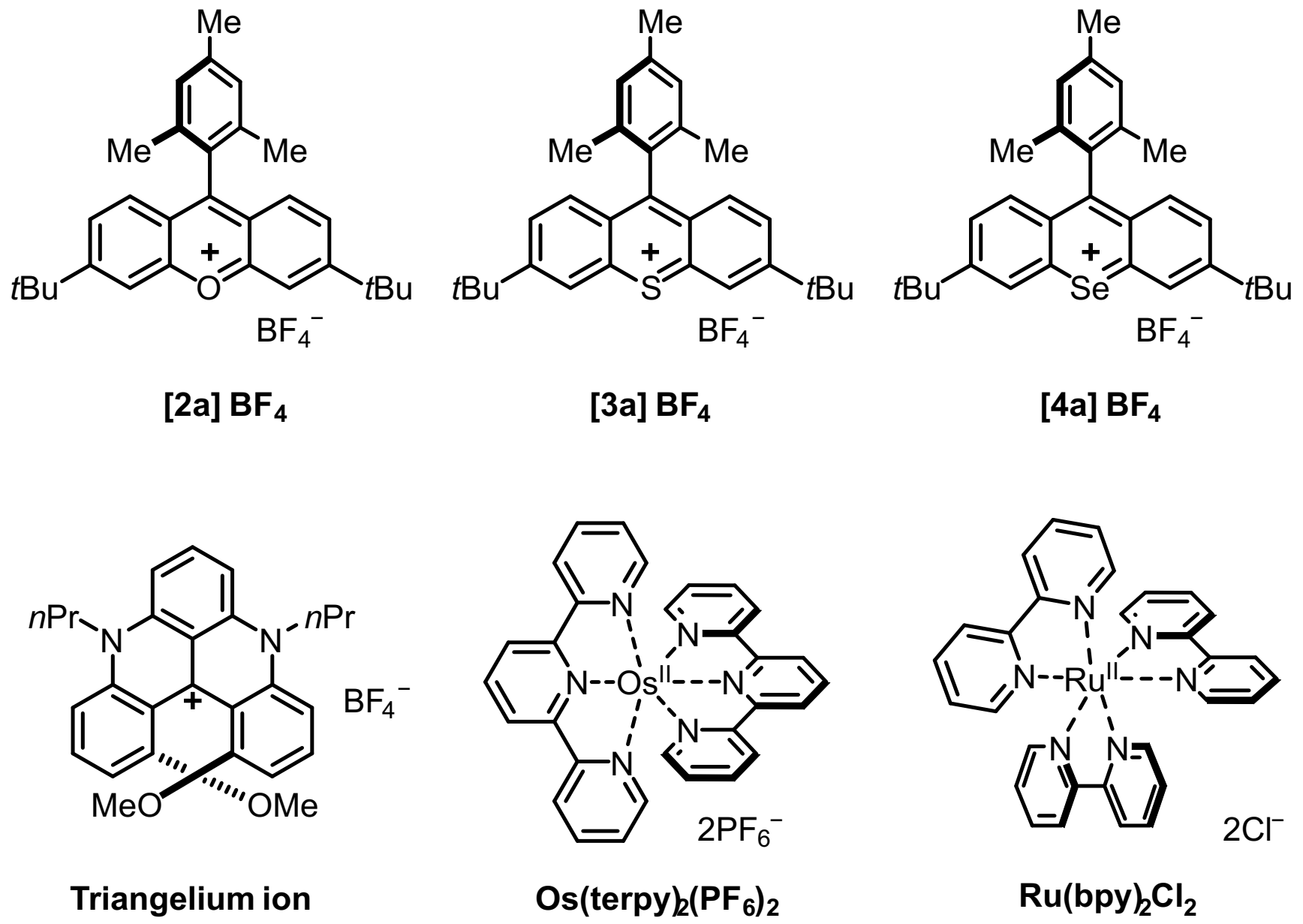

General procedure for the C–H amination reaction at a 0.10 mmol scale

To a 4-mL borosilicate vial equipped with a magnetic stir bar, 1,3,5-trimethoxybenzene (**10**) (17.0 mg, 1.00 equiv., 0.100 mmol), 3,5-dimethylpyrazole (**11**) (49.0 mg, 5.00 equiv., 0.50 mmol), photocatalyst, and dry solvent (1.0 mL) were added. The vial was sealed with a cap equipped with a septum. The

solution was sparged with an O_2 balloon for 5 min. After sparging, the vent needle was removed, and the O_2 balloon was maintained. The resulting solution was irradiated with red-light (red-light irradiation, λ_{max} = 650 nm) while stirred. The reaction was carried out at 27 °C for 65 h. After stirring the reaction for the given time, the reaction mixture was concentrated by rotary evaporation under reduced pressure. The residue was fully solubilized in $CDCl_3$ (0.50 mL), transferred to a NMR tube. CH_2Br_2 (7.1 µL, 0.10 mmol = theoretical maximum yield) was added as internal standard, and the yield was determined by ^{1}H NMR spectroscopy using CH_2Br_2 as internal standard by comparing the integration of the CH_2Br_2 signal at 4.89 ppm (s, 2H) to the signal of **12** at 3.82 ppm (s, 3H).

Table S16. C–H amination reaction using different xanthylium photocatalysts.

Entry	PC	PC loading, mol%	Solvent	Yield **12** (%)[a]
1	**[2a] BF_4**	10	DCE	n.r.
2	**[3a] BF_4**	10	DCE	23
3	**[4a] BF_4**	10	DCE	87
6	**[2a] BF_4**	10	CH_2Cl_2	<5
7	**[3a] BF_4**	10	CH_2Cl_2	26
8	**[4a] BF_4**	10	CH_2Cl_2	53

** The yield was determined by ^{1}H NMR spectroscopy using CH_2Br_2 as internal standard.*

Table S17. C–H amination reaction using different photocatalysts.

Entry	PC	PC loading, mol%	Solvent	Yield **12** (%)[a]
1	**[4a] BF_4**	10	DCE	87
2	**Triangulenium ion**	10	DCE	n.r.
3	**$Os(terpy)_2(PF_6)_2$**	1	DCE	n.r.
6	**$Ru(bpy)_3Cl_2$**	1	DCE	n.r.
7	**[4a] BF_4**	10	CH_2Cl_2	53
8	**Triangulenium ion**	10	CH_2Cl_2	n.r.
9	**$Os(terpy)_2(PF_6)_2$**	1	CH_2Cl_2	n.r.
10	**$Ru(bpy)_3Cl_2$**	1	DCE	n.r.

** The yield was determined by ^{1}H NMR spectroscopy using CH_2Br_2 as internal standard.*

EX SITU IRRADIATION NMR EXPERIMENTS

[4+2]-Cycloaddition reaction at 0.10 mmol scale catalyzed by photocatalysts

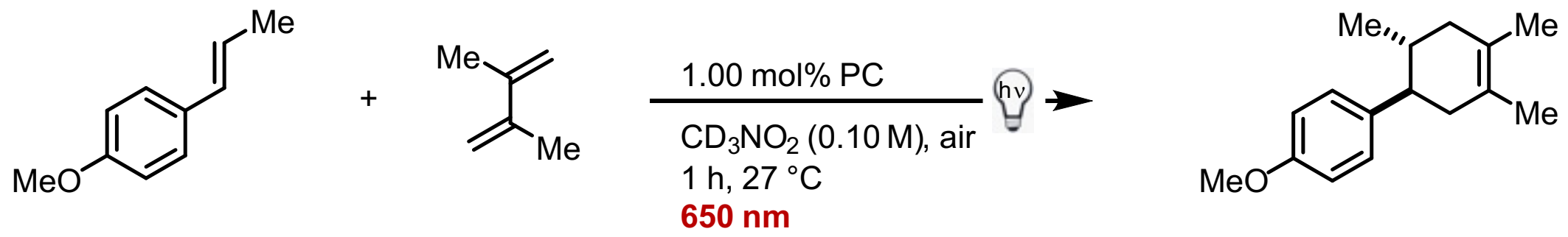

7 **8** 3.00 equiv. **9**

Tested photocatalysts

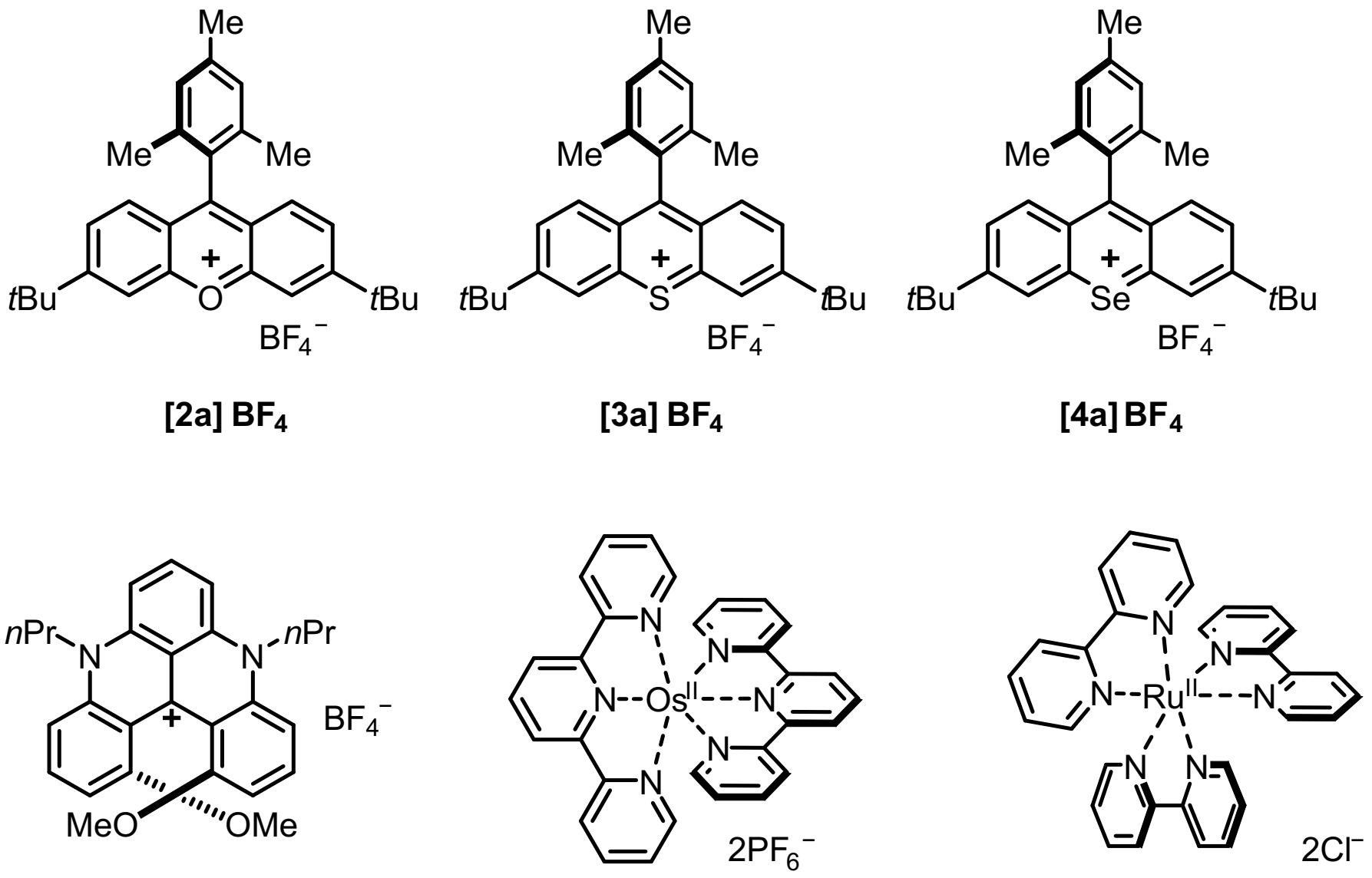

[2a] BF$_4$ **[3a] BF$_4$** **[4a] BF$_4$**

Triangelium Ion **Os(terpy)$_2$(PF$_6$)$_2$** **Ru(bpy)$_2$Cl$_2$**

To a J-Young NMR tube containing photocatalyst (0.6 μmol, 0.01 equiv.), trans-anethole **7** (60 μmol, 1.00 equiv.) and dimethylbutadiene **8** (180 μmol, 3.00 equiv.) were added using a microliter syringe. CD_3NO_2 (0.60 mL) was added to the NMR tube using a 1.0-mL syringe. The tube was capped, and the mixture was analyzed by ^{1}H NMR spectroscopy (see for example **Figure S100**, top panel). The NMR tube cap was irradiated with red-light (red-light irradiation, λ_{max} = 650 nm). After the specified time intervals, irradiation was stopped, reaction progress was monitored by ^{1}H NMR (8 NMR experiments total with 16 scans per analysis), and irradiation with red-light was continued until a total irradiation time of 1 h was reached (see for example **Figure S101**).

To calculate the conversion of **7** to **9**, the signals due to the aromatic hydrogens of trans-anethole were used (**7**: d, 7.30 ppm, 2H and **9**: d, 7.15 ppm, 2H; see for example **Figure S100**, bottom panel). Since no other signals in the aromatic region were detected, the mass-balance corresponding to **10** and **12** was assumed to be constant. Thus, the final concentrations were obtained by calculating the peak area ratio of **12** to **10** and then referencing the relative amounts to the known starting material concentration of **10** (0.10 M).

For catalysts **[3a] BF_4** and **[4a] BF_4**, small amounts of product **9** (<1%) were detected prior to red-light irradiation. This was assigned to be caused by ambient light exposure of the J-Young NMR tubes before measurement of the first ^{1}H NMR spectrum.

^{1}H NMR spectroscopy of [4+2] cycloaddition reaction catalyzed by [2a] BF_4

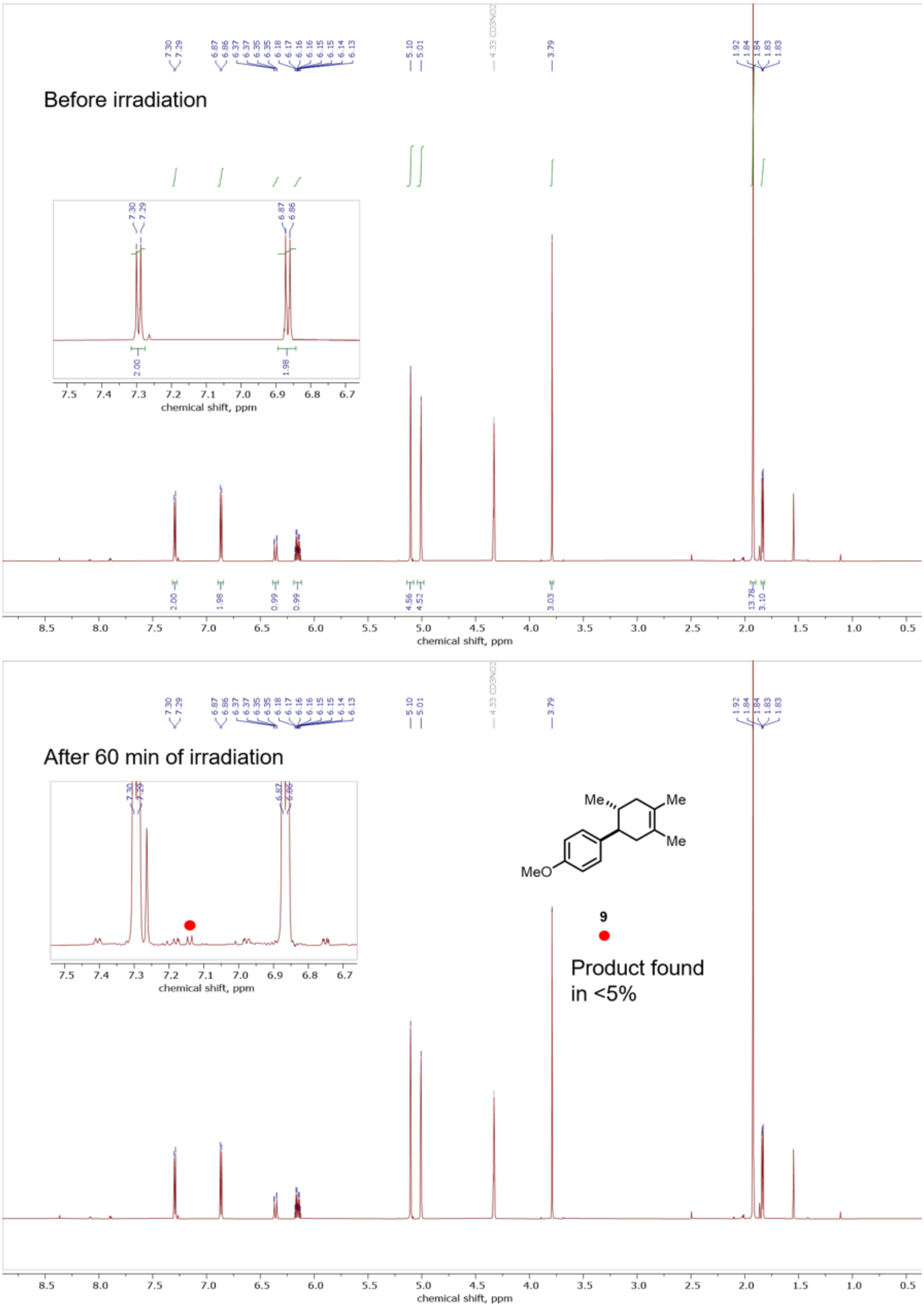

Figure S100. ^{1}H NMR spectrum of reaction mixture with **[2a] BF_4** in CD_3NO_2 before irradiation (top) and after irradiation for 60 min (bottom).

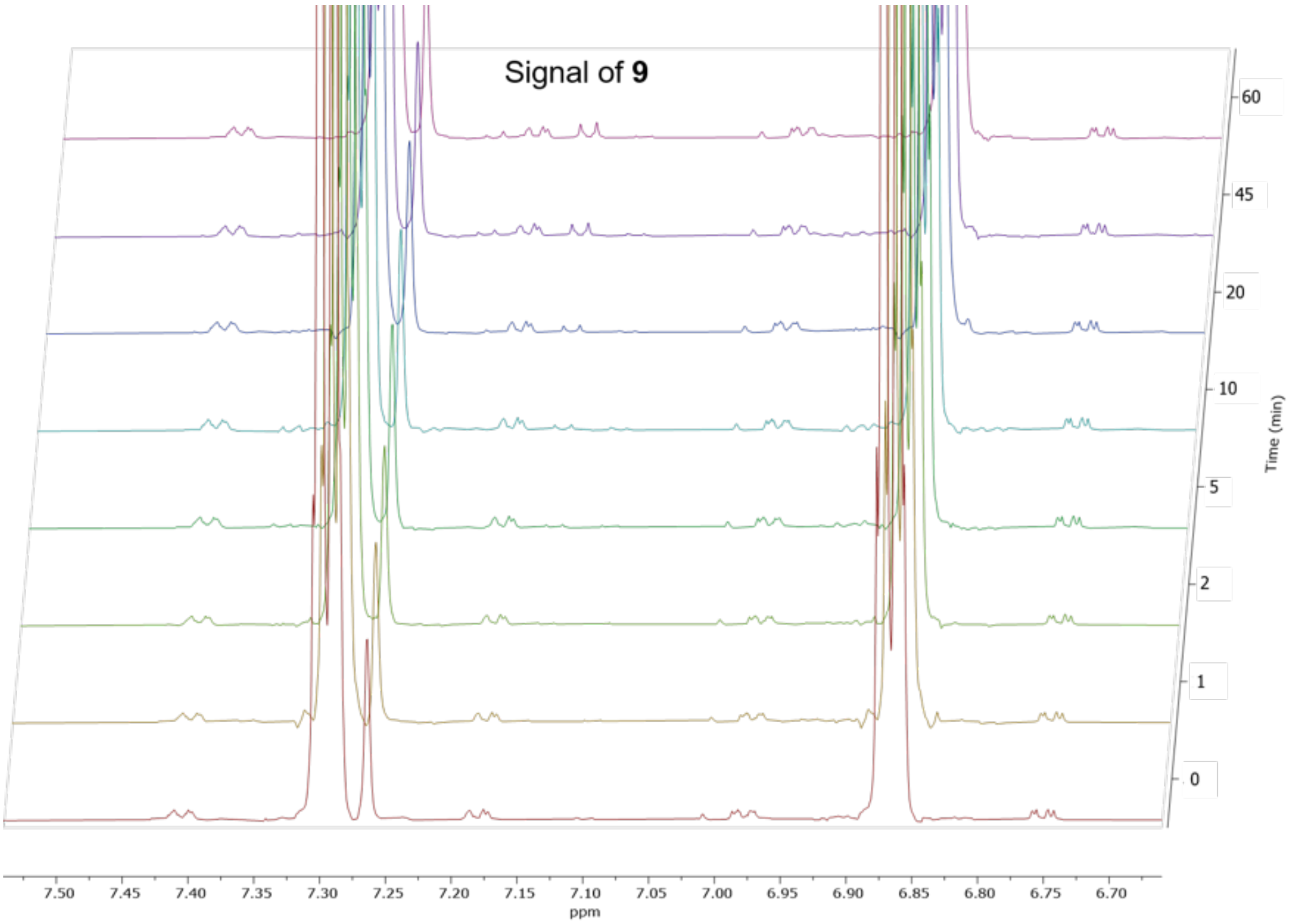

Figure S101. ^{1}H NMR spectra (x-axis) of reaction mixture after different irradiation times (y-axis).

Reaction profile of [2a] BF_4 catalyzed [4+2] cycloaddition reaction

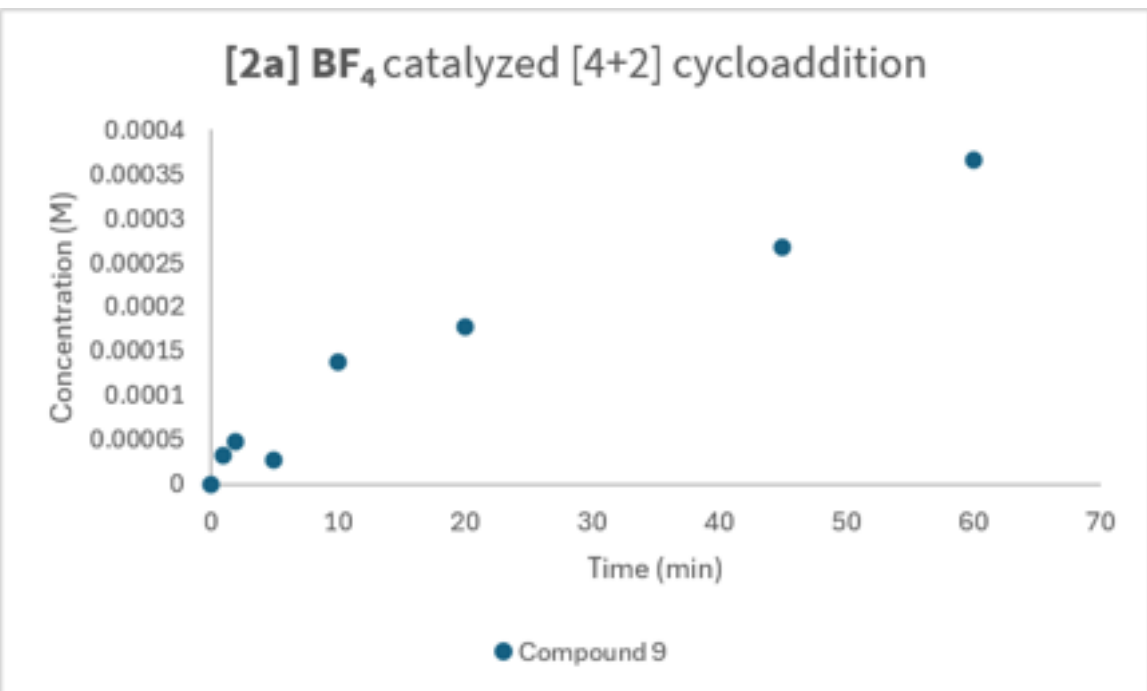

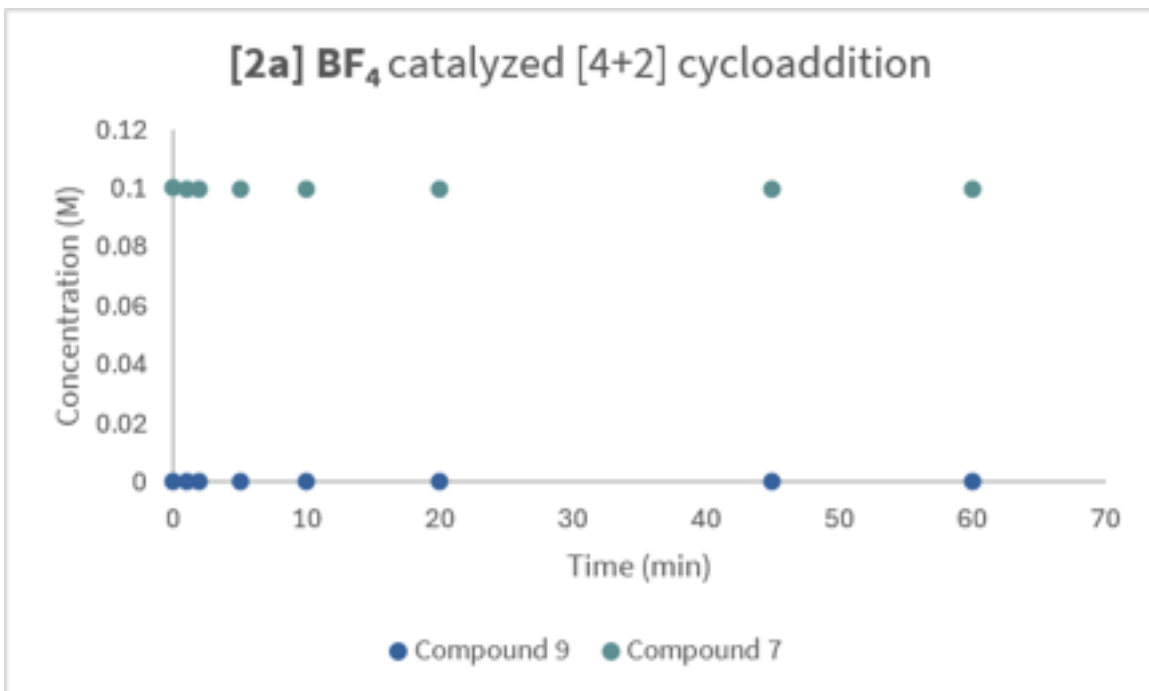

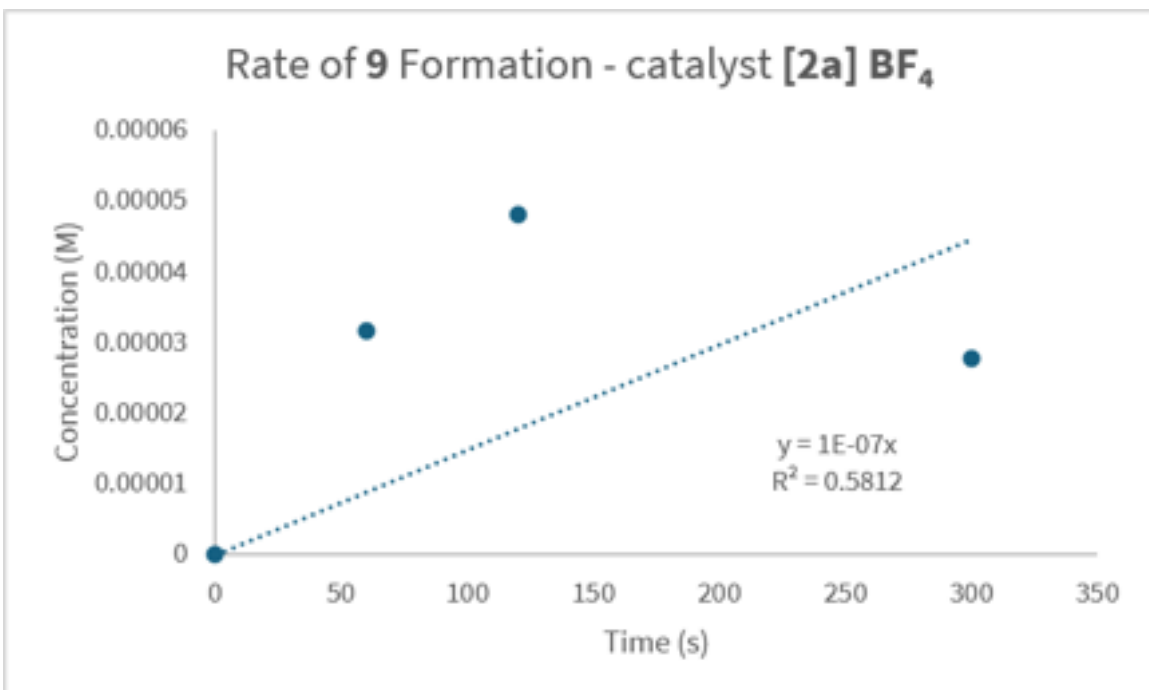

Figure S102. Reaction profiles for **[2a] BF_4** catalyzed [4+2] cycloaddition reaction (top left, top right). Initial rate of the reaction was determined by the slope of the linear fit (bottom).

After 60 min of irradiation, the reaction mixture was analyzed by ^{1}H NMR. **7** and **9** were found to be present in relative amounts of >95% and <5%, respectively. The rate of the reaction was determined by applying a linear fit to the first four concentrations determined by ^{1}H NMR after 5 min total irradiation time. The resulting initial rate was found to be:

$k_{2a} = 1 \cdot 10^{-7}\ M\ s^{-1}$.

^{1}H NMR spectroscopy of [4+2] cycloaddition reaction catalyzed by [3a] BF_4

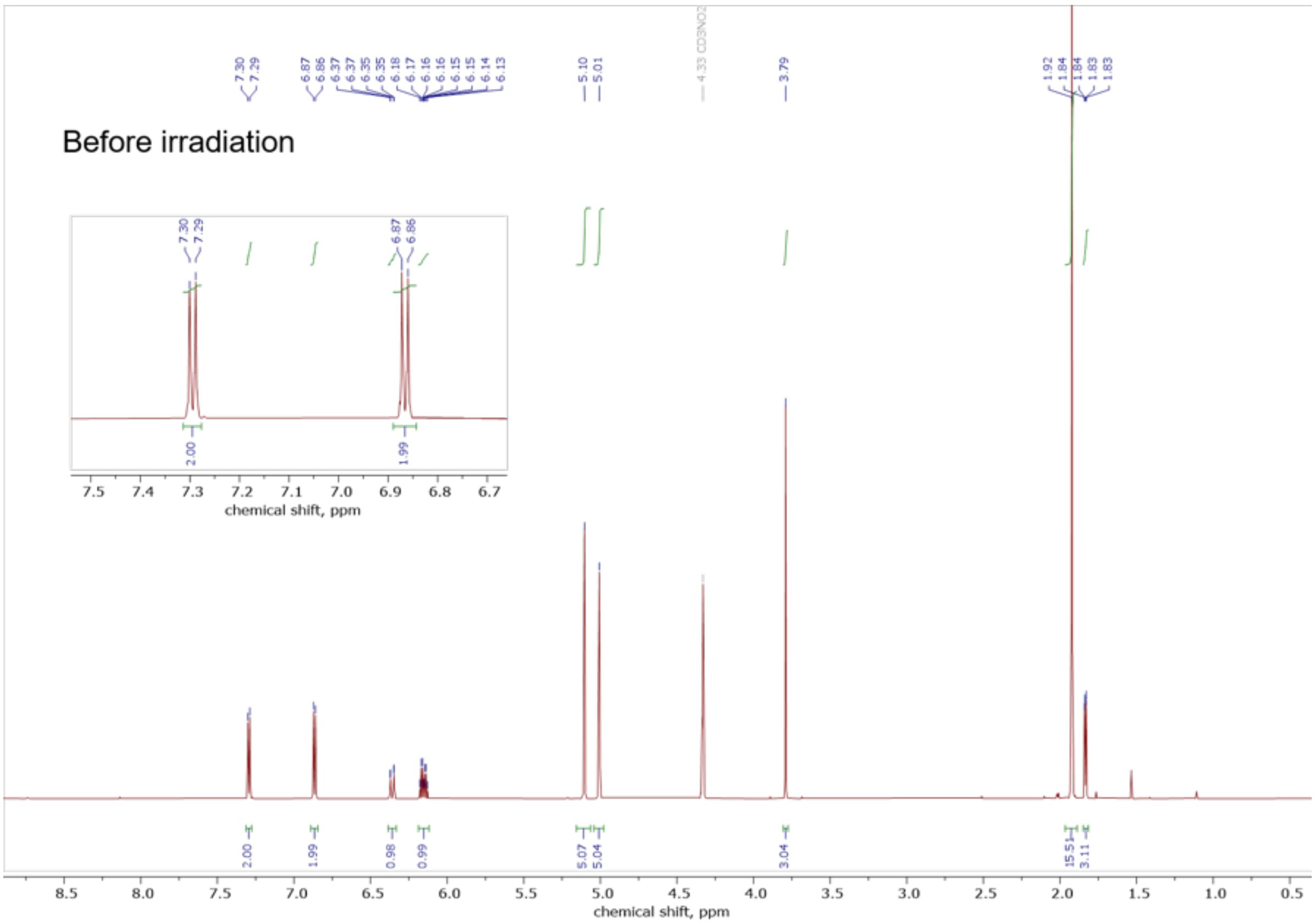

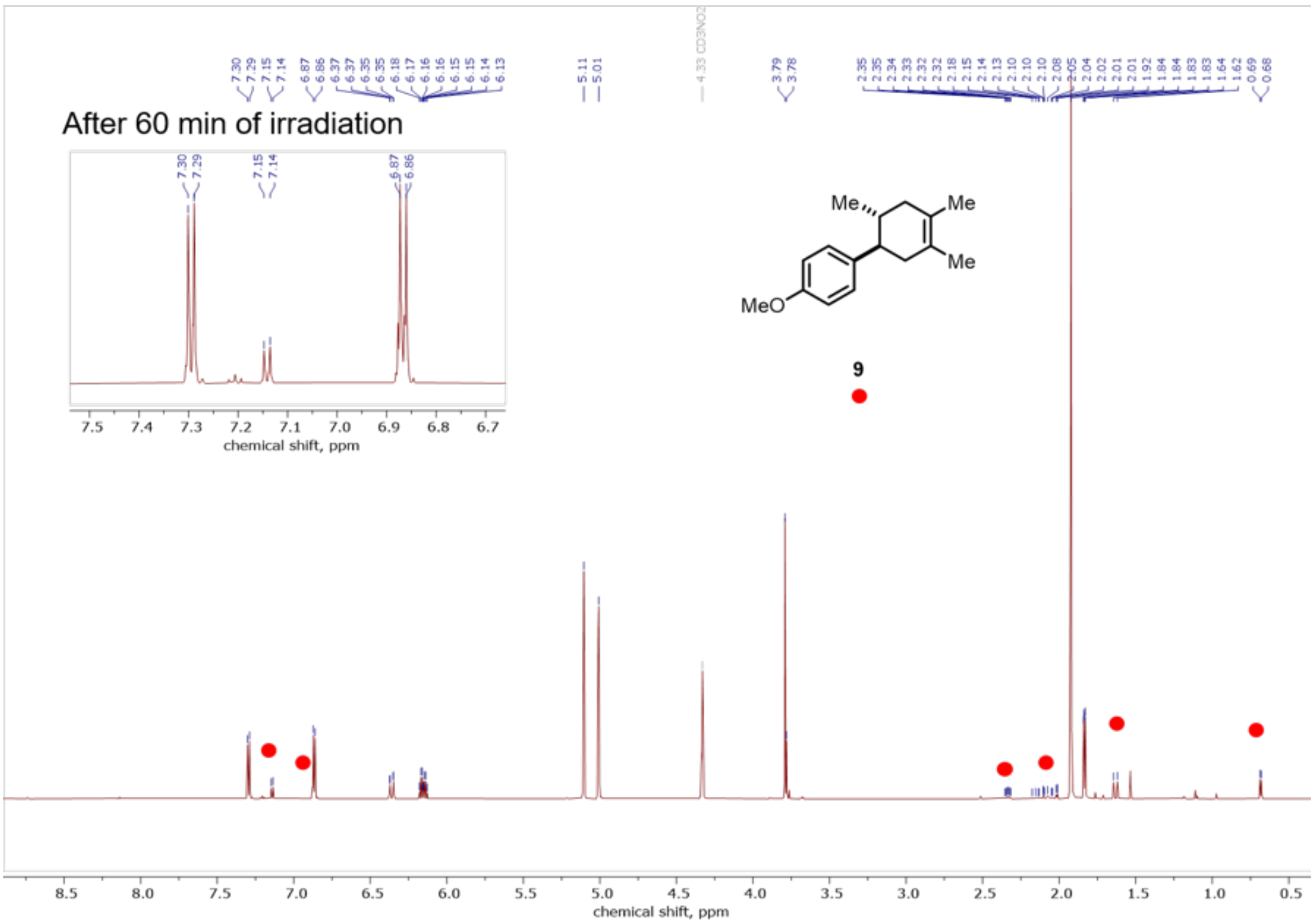

Figure S103. ^{1}H NMR spectrum of reaction mixture with **[3a] BF**$_4$ in CD_3NO_2 before irradiation (top) and after irradiation for 60 min (bottom).

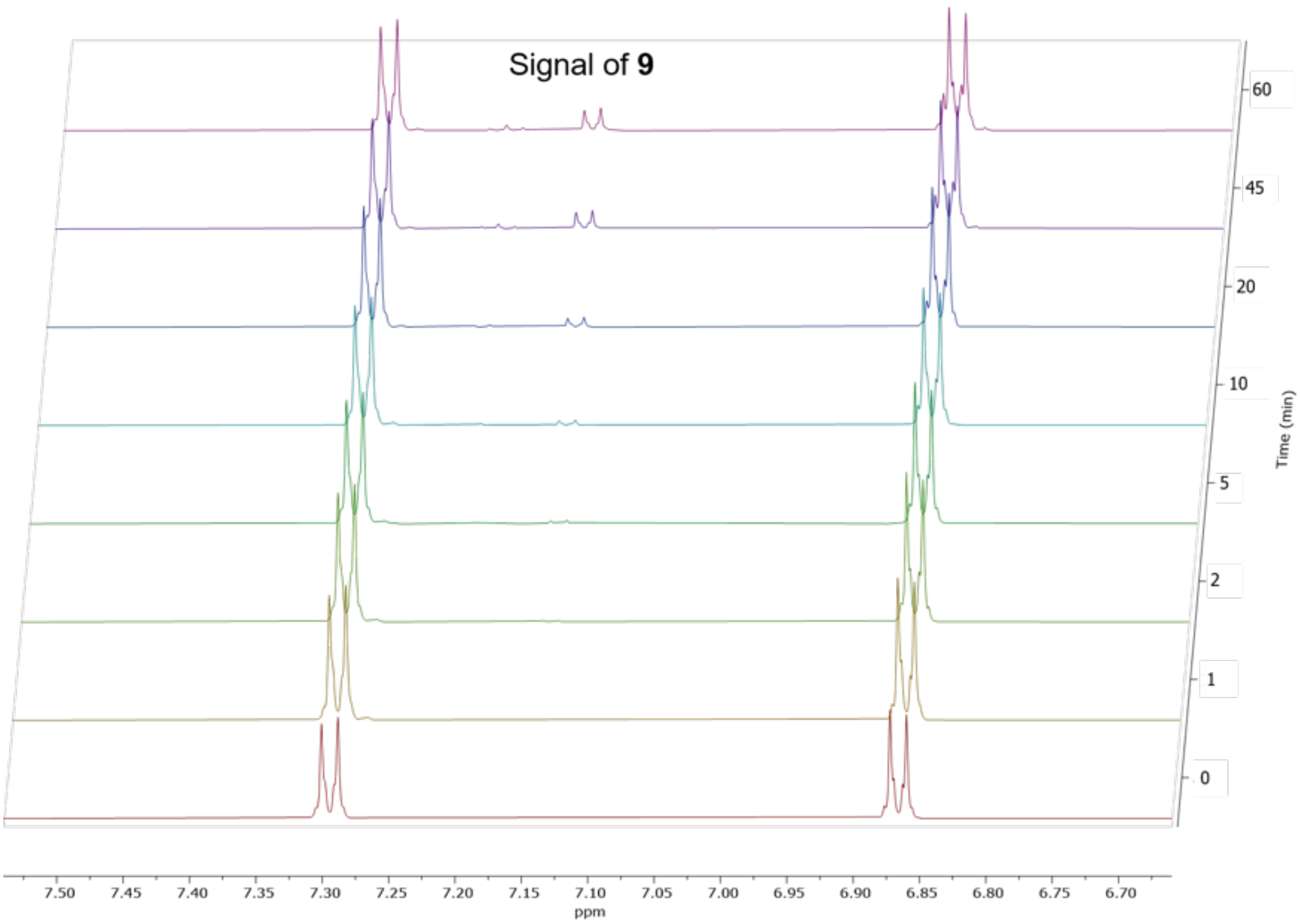

Reaction profile of [3a] BF_4 catalyzed [4+2] cycloaddition reaction

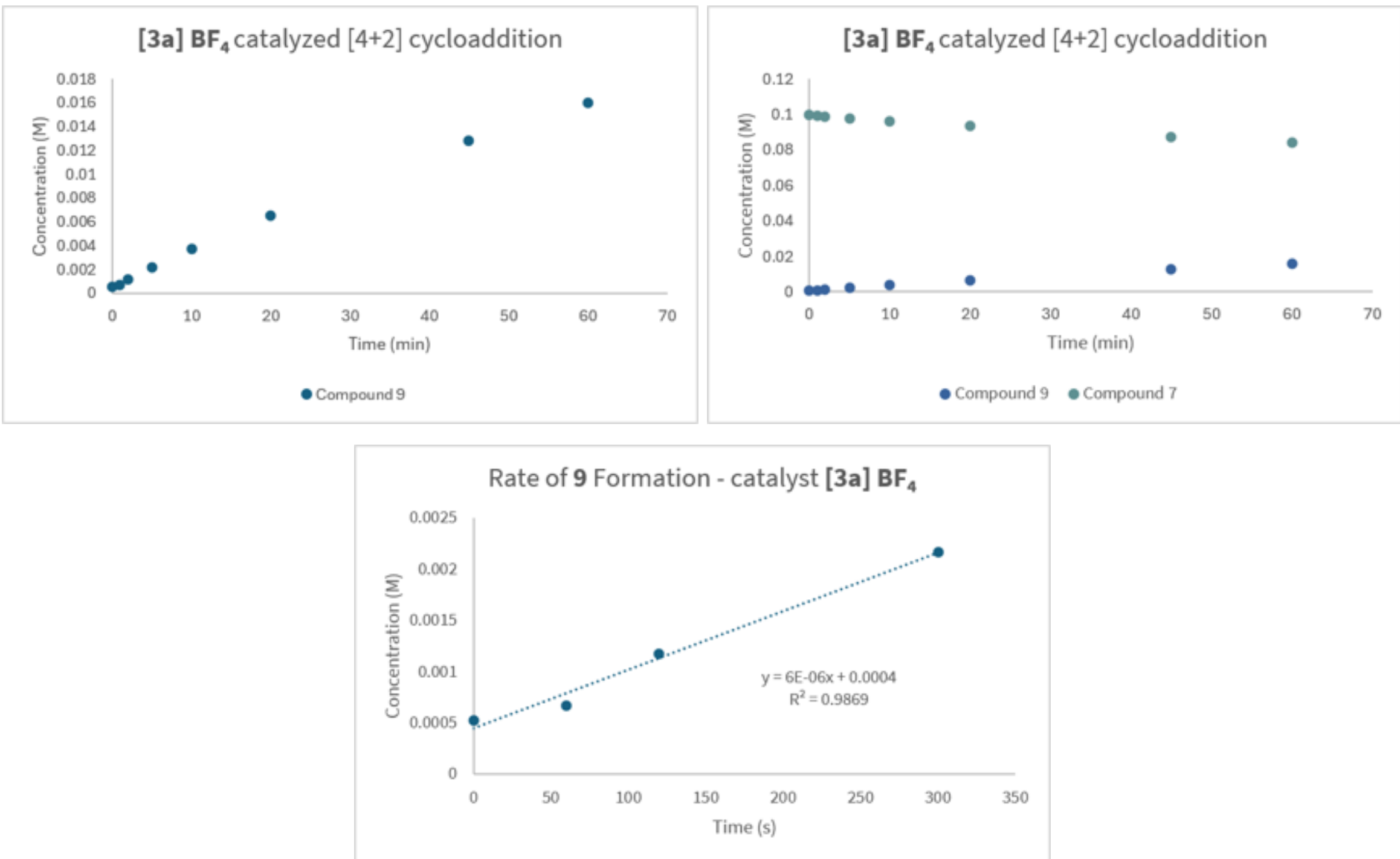

Figure S104. Reaction profiles for **[3a] BF4** catalyzed [4+2] cycloaddition reaction (top left, top right). Initial rate of the reaction was determined by the slope of the linear fit (bottom).

After 60 min of irradiation, the reaction mixture was analyzed by ^{1}H NMR. **7** and **9** were found to be present in relative amounts of 84% and 16%, respectively. The rate of the reaction was determined by

applying a linear fit to the first four concentrations determined by ^{1}H NMR after 5 min total irradiation time. The resulting initial rate was found to be:

$k_{3a} = 6 \cdot 10^{-6}\ M\ s^{-1}$.

^{1}H NMR spectroscopy of [4+2] cycloaddition reaction catalyzed by [4a] BF_4

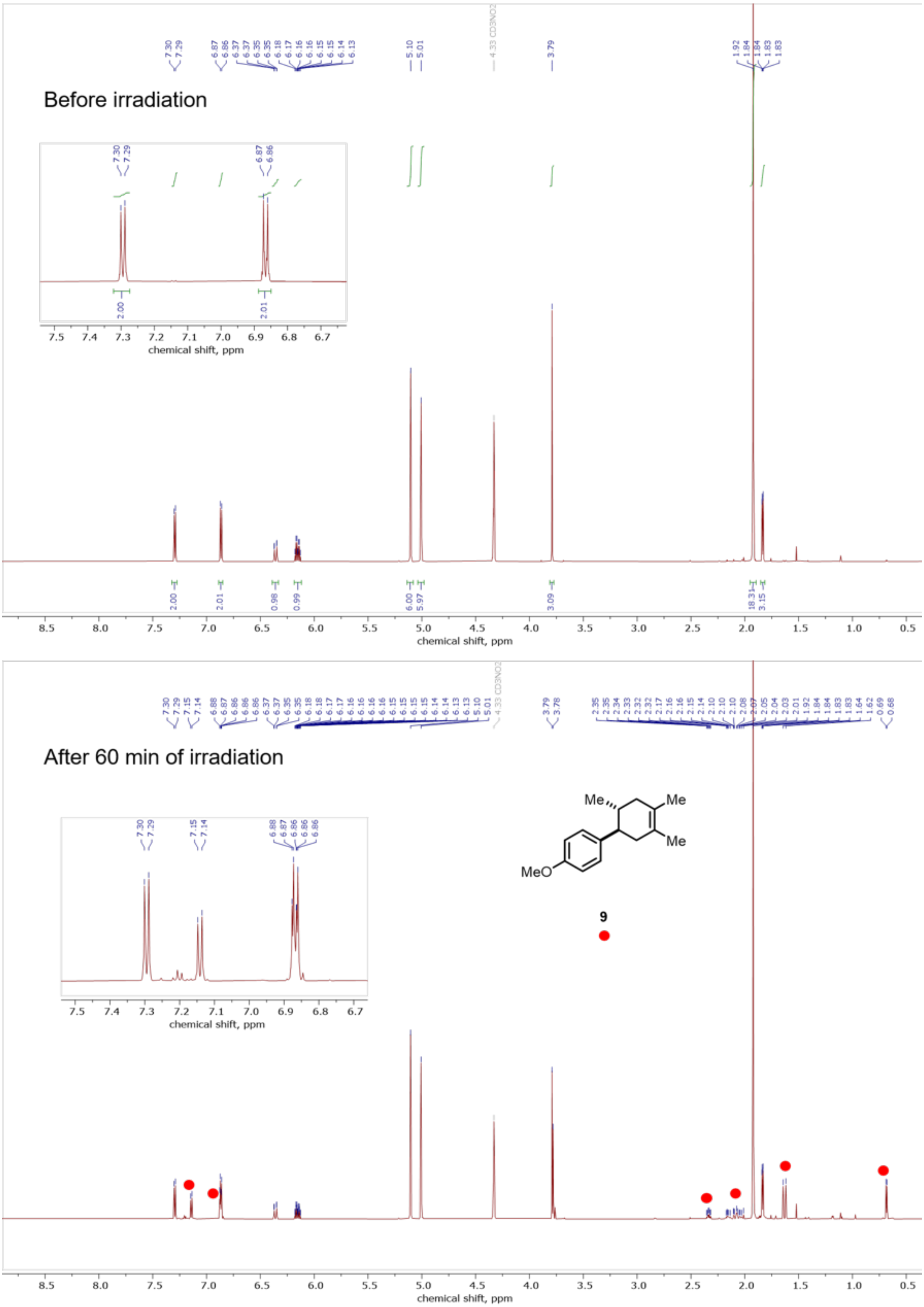

Figure S105. ^{1}H NMR spectrum of reaction mixture with **[4a] BF_4** in CD_3NO_2 before irradiation (top) and after irradiation for 60 min (bottom).

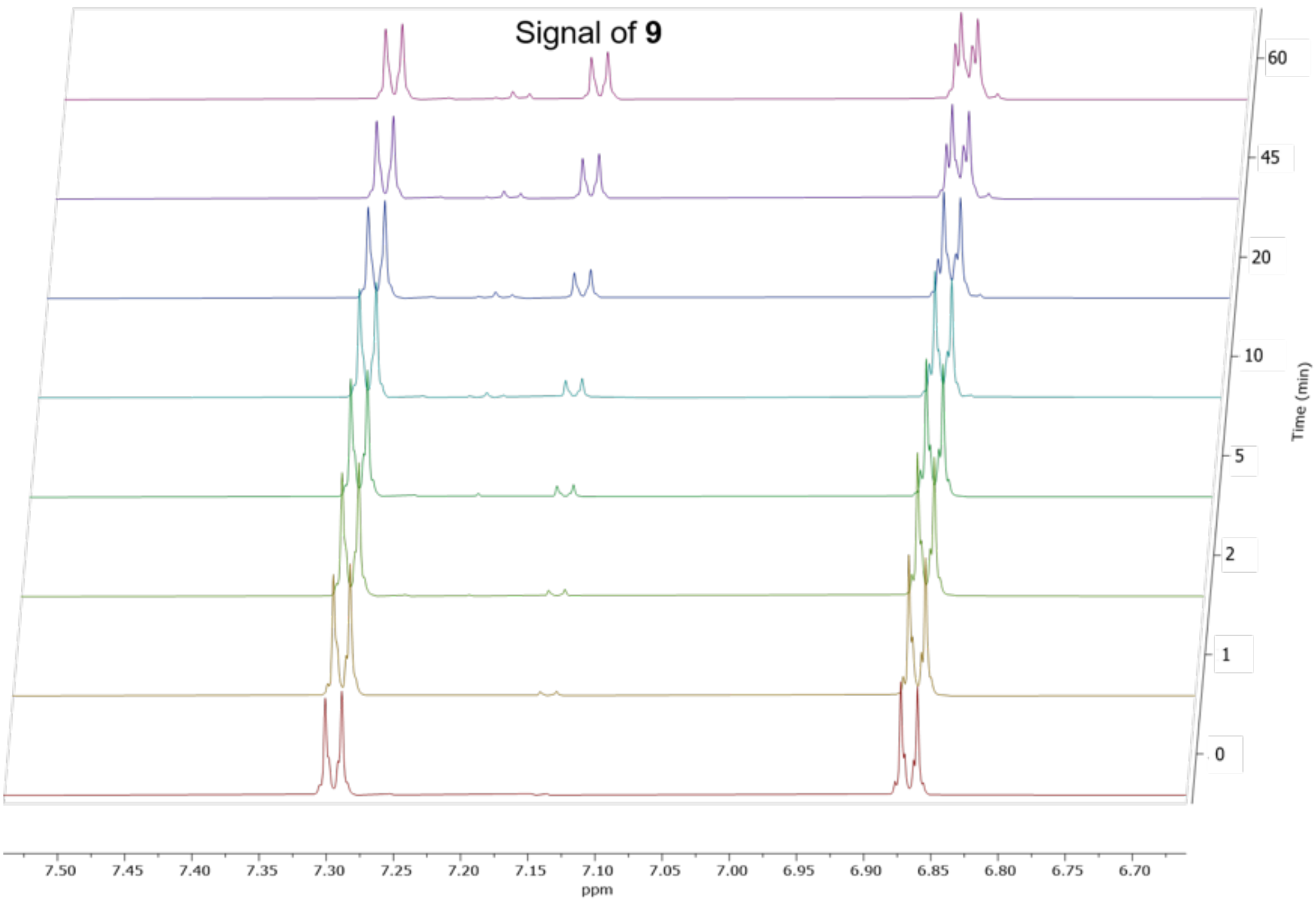

Figure S128. ^{1}H NMR spectra (x-axis) of reaction mixture after different irradiation times (y-axis).

Reaction profile of [4a] BF_4 catalyzed [4+2] cycloaddition reaction

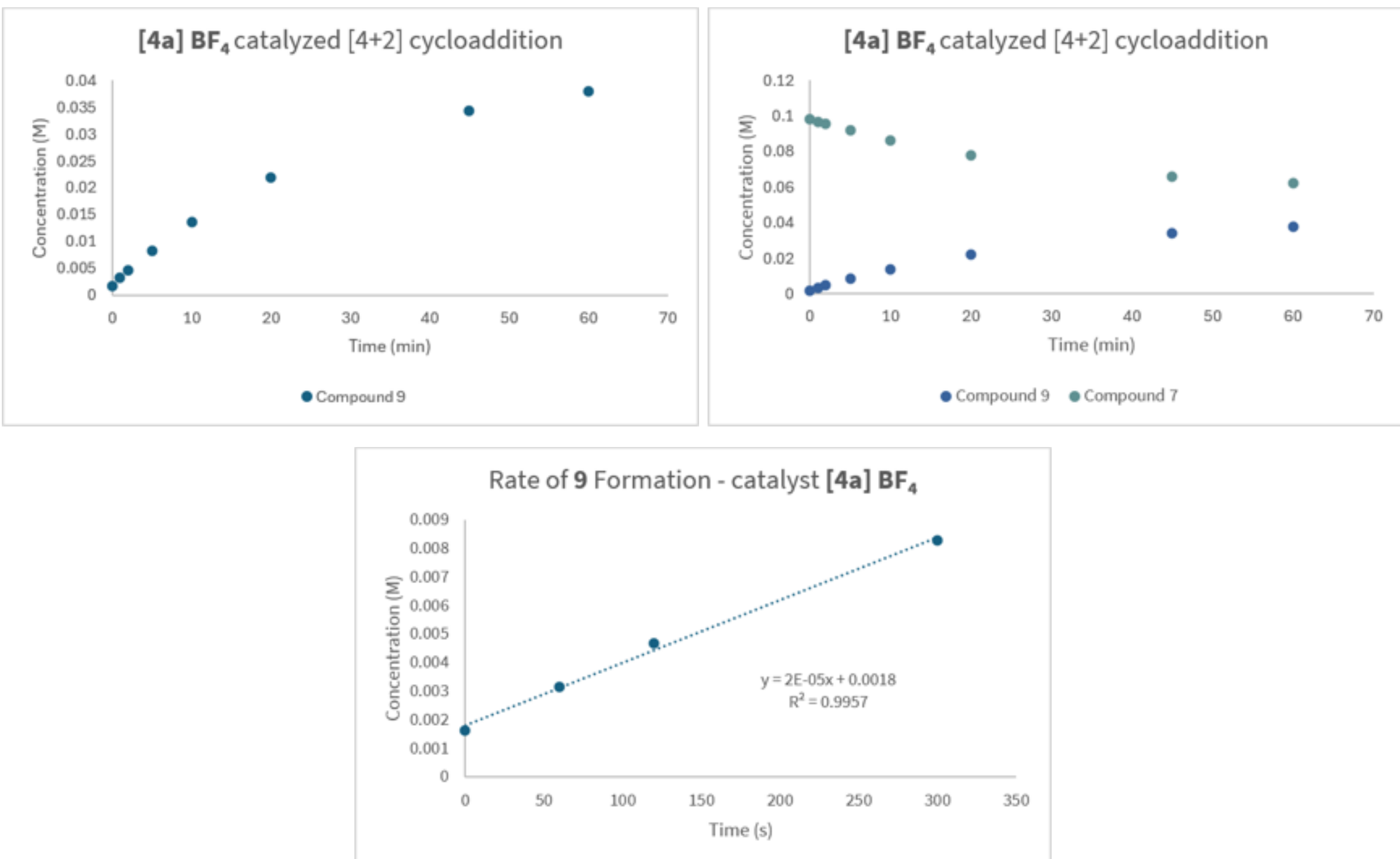

Figure S106. Reaction profiles for **[2a] BF₄** catalyzed [4+2] cycloaddition reaction (top left, top right). Initial rate of the reaction was determined by the slope of the linear fit (bottom).

After 60 min of irradiation, the reaction mixture was analyzed by ^{1}H NMR. **7** and **9** were found to be present in relative amounts of 62% and 38%, respectively. The rate of the reaction was determined by applying a linear fit to the first four concentrations determined by ^{1}H NMR after 5 min total irradiation time. The resulting initial rate was found to be:

$k_{4a} = 2 \cdot 10^{-5}\, M\, s^{-1}$.

^{1}H NMR spectroscopy of [4+2] cycloaddition reaction catalyzed by Triangulenium Ion

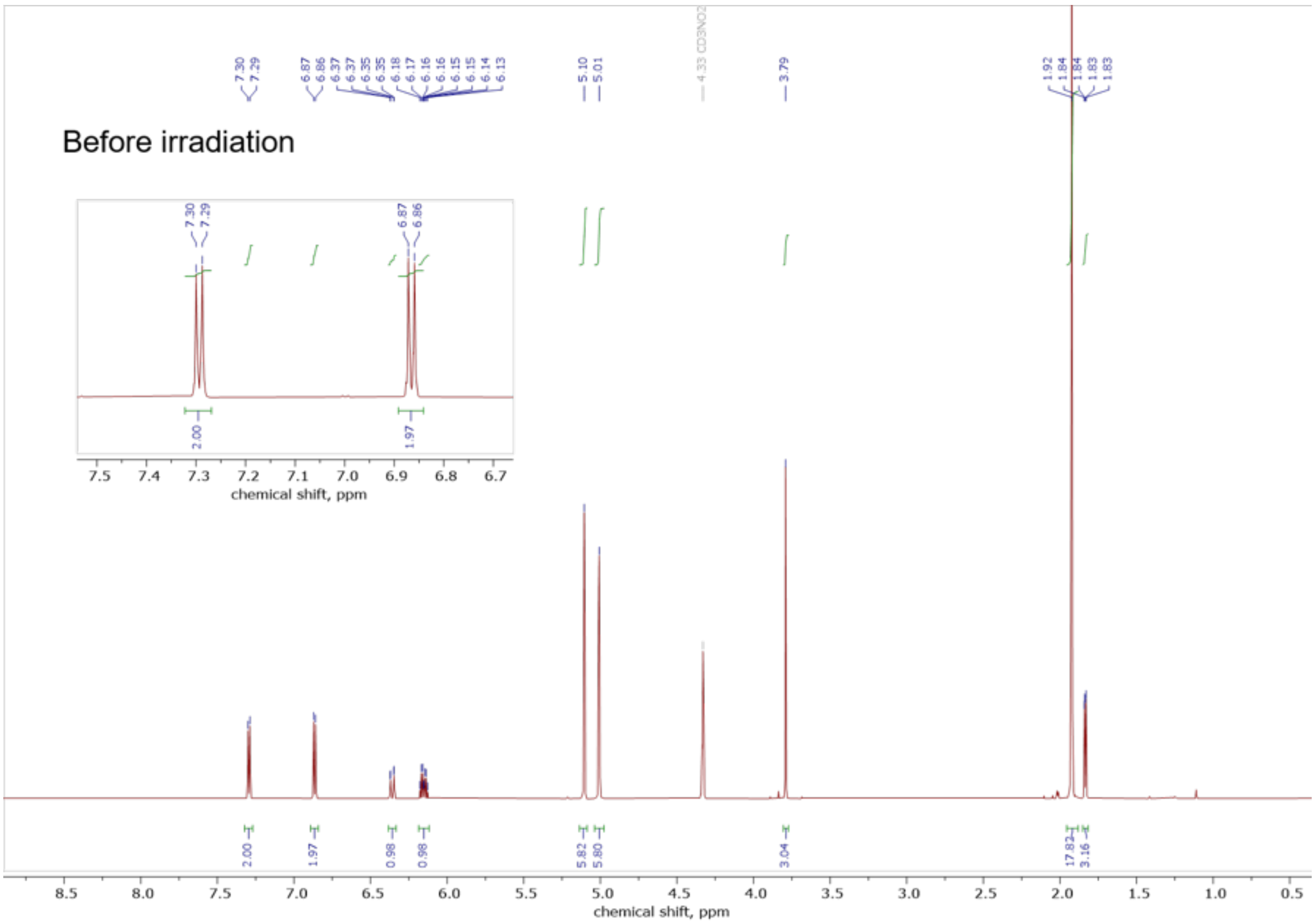

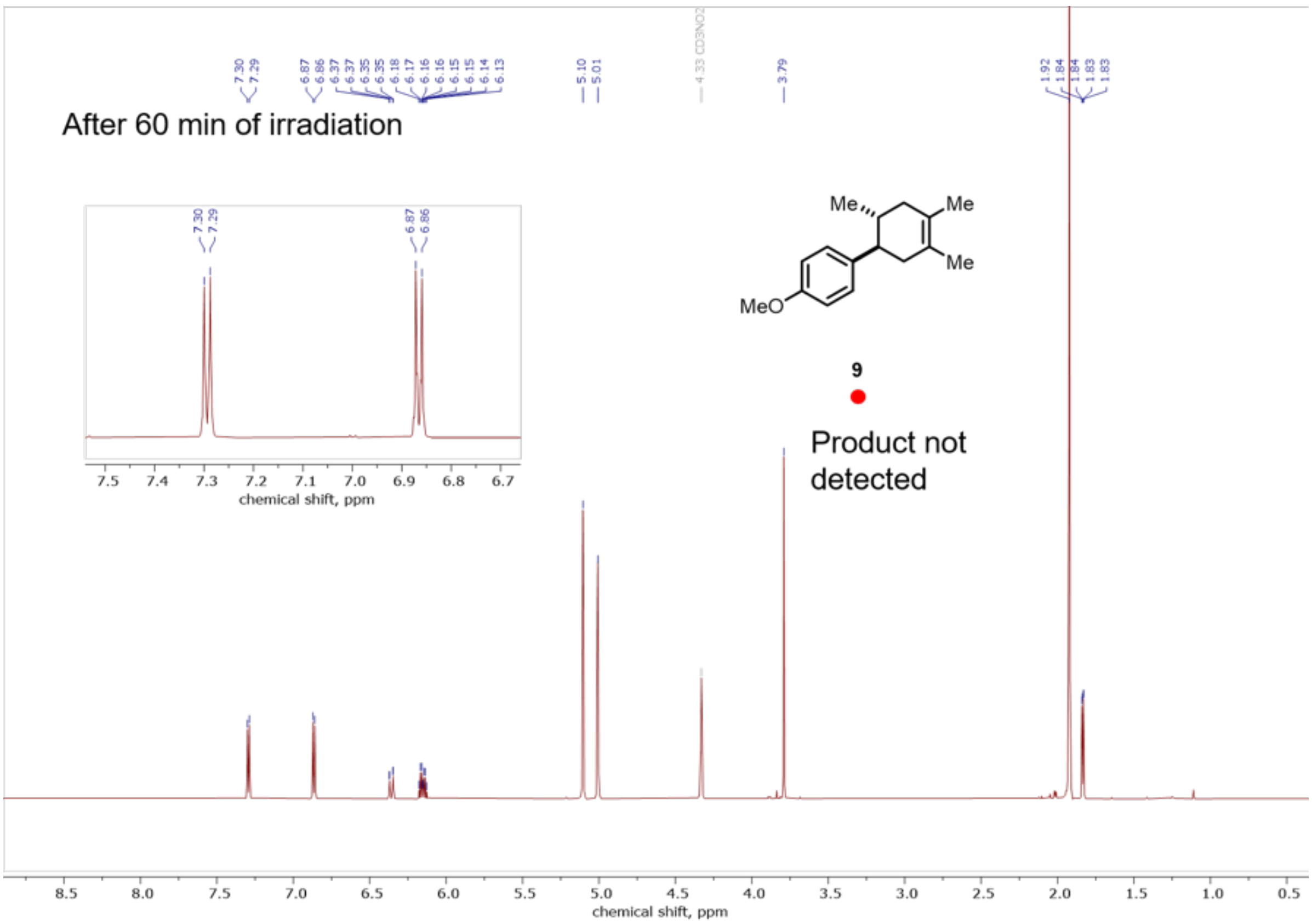

Figure S107. ^{1}H NMR spectrum of reaction mixture with **Triangulenium Ion** in CD_3NO_2 before irradiation (top) and after irradiation for 60 min (bottom).

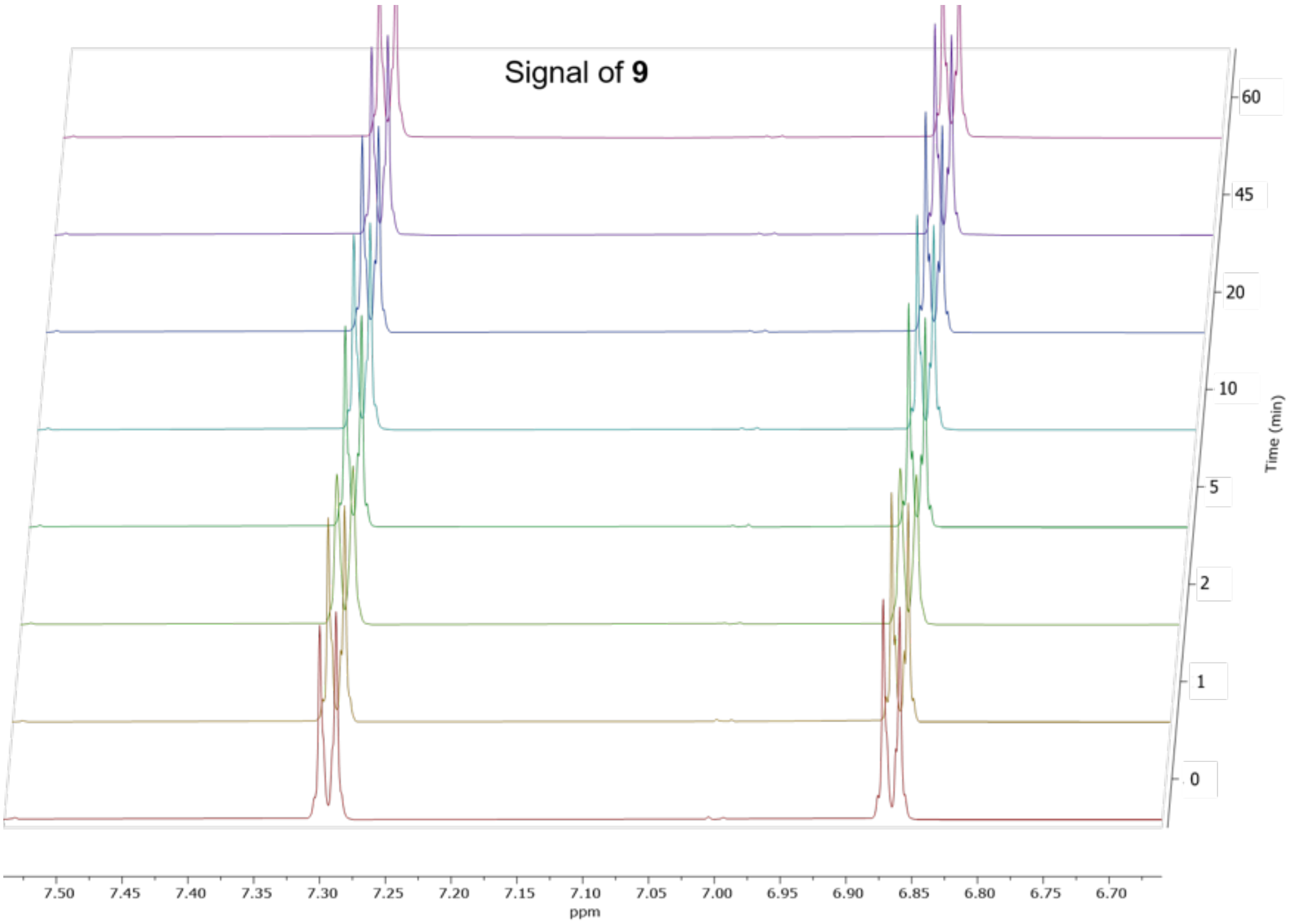

Figure S108. ^{1}H NMR spectra (x-axis) of reaction mixture after different irradiation times (y-axis).

After 60 min of irradiation the reaction mixture was analyzed by ^{1}H NMR. It was found that no conversion of **7** to **9** took place under the reaction conditions and therefore no rate could be determined.

^{1}H NMR spectroscopy of [4+2] cycloaddition reaction catalyzed by Os(terpy)₂

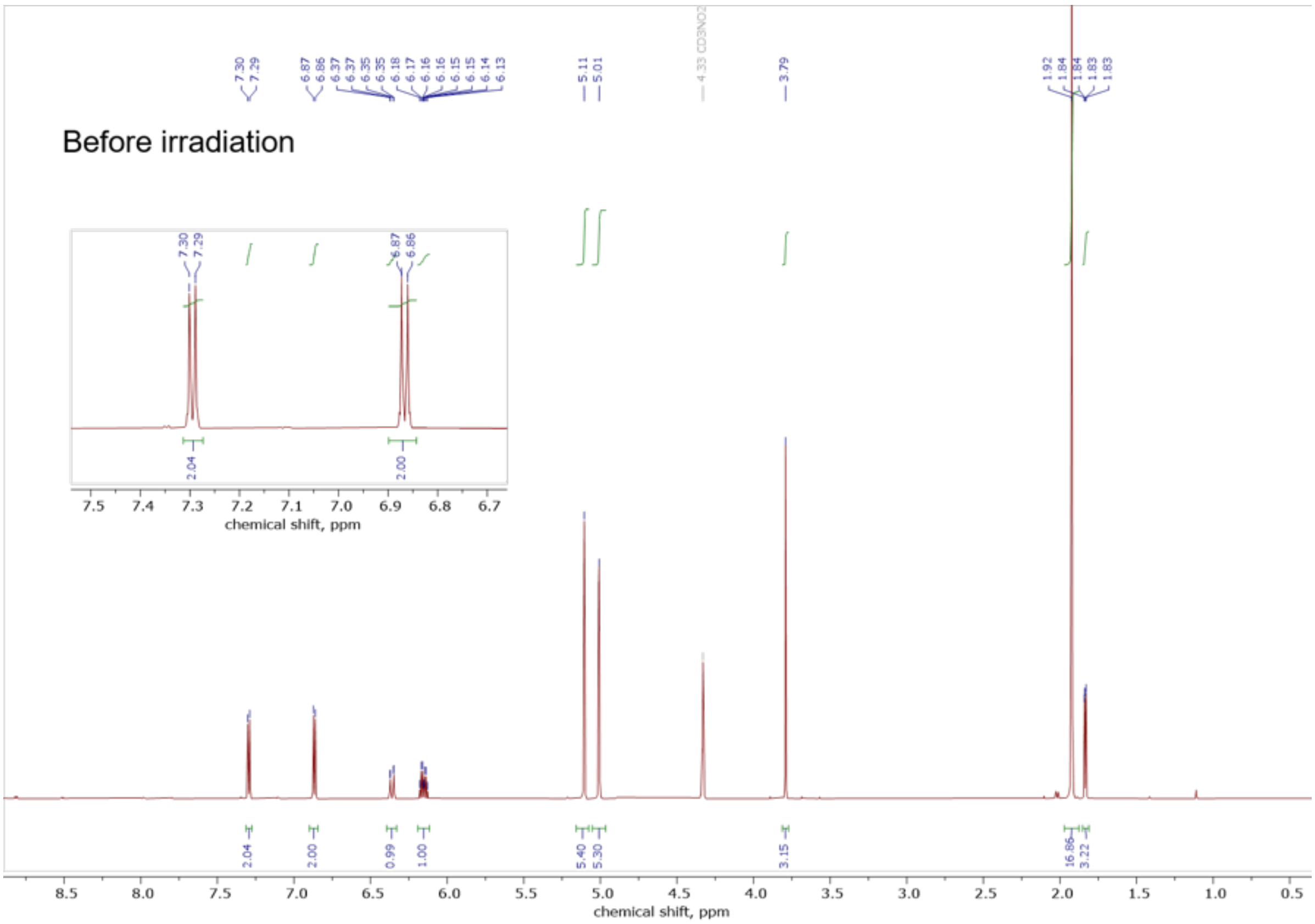

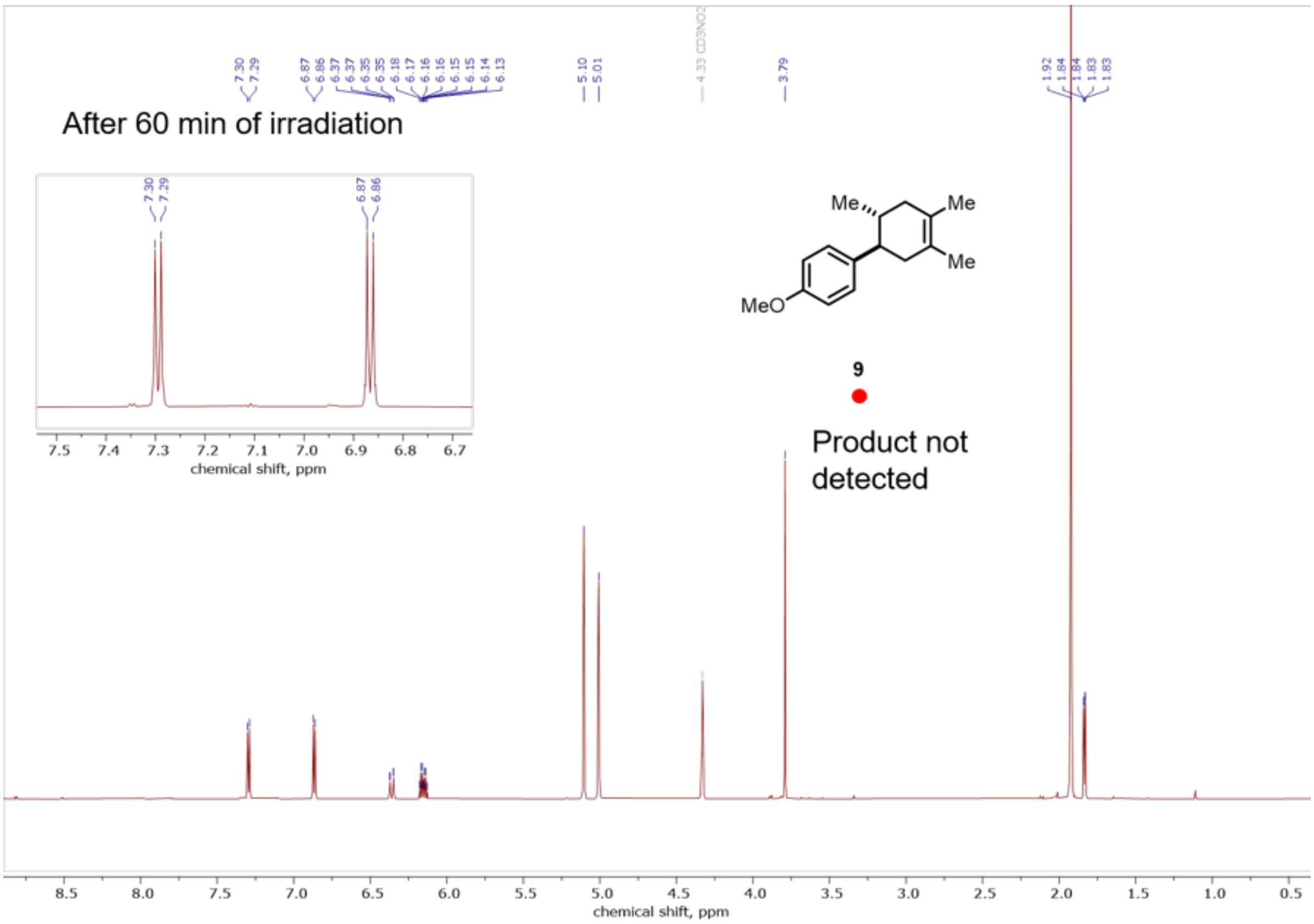

Figure S109. ^{1}H NMR spectrum of reaction mixture with **Os(terpy)₂** in CD_3NO_2 before irradiation (top) and after irradiation for 60 min (bottom).

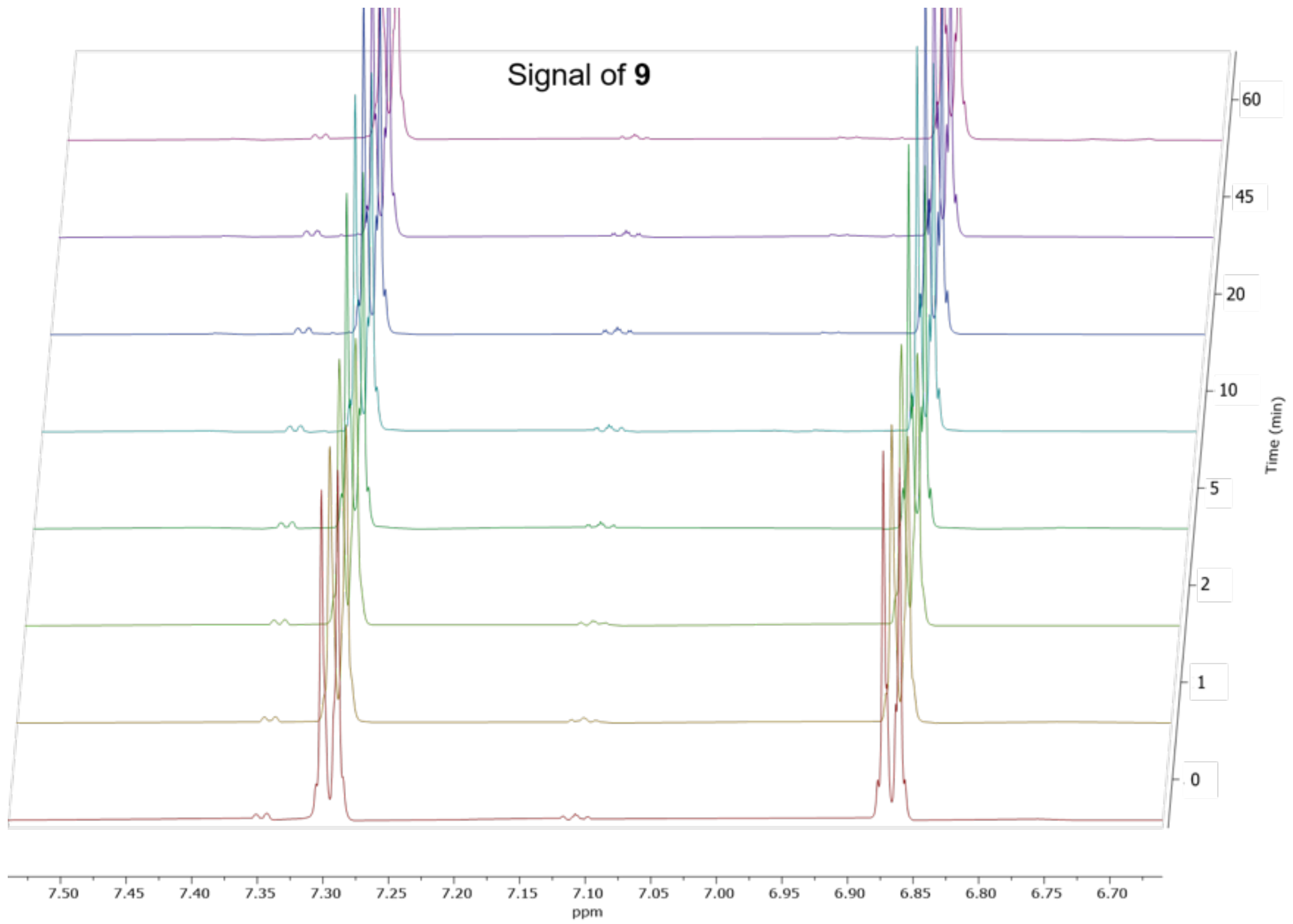

Figure S110. ^{1}H NMR spectra (x-axis) of reaction mixture after different irradiation times (y-axis).

After 60 min of irradiation the reaction mixture was analyzed by ^{1}H NMR. It was found that no conversion of **7** to **9** took place under the reaction conditions and therefore no rate could be determined.

^{1}H NMR spectroscopy of [4+2] cycloaddition reaction catalyzed by Ru(bpy)$_3$

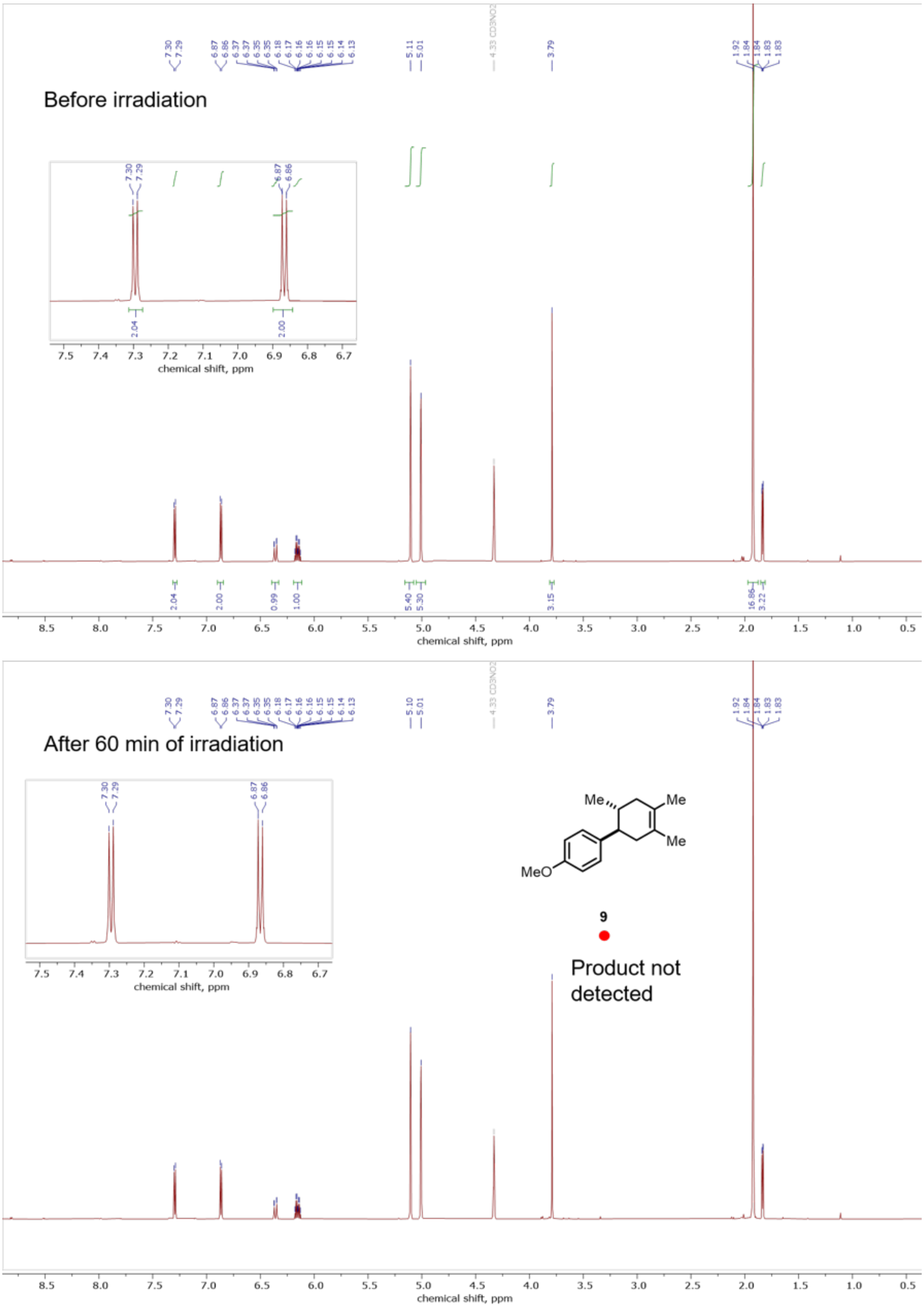

Figure S129. ^{1}H NMR spectrum of reaction mixture with **Ru(bpy)$_3$** in CD_3NO_2 before irradiation (top) and after irradiation for 60 min (bottom).

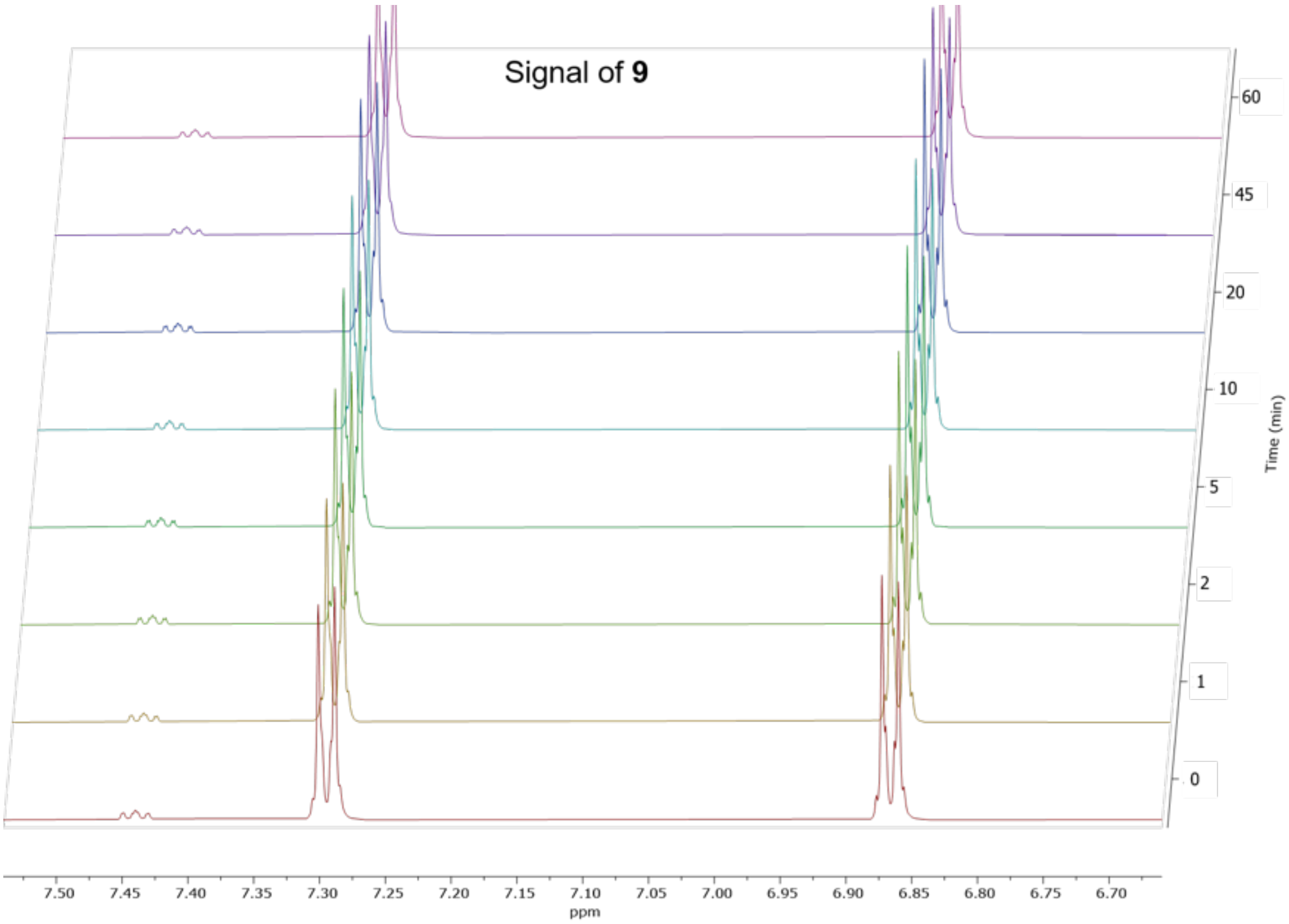

Figure S111. ¹H NMR spectra (x-axis) of reaction mixture after different irradiation times (y-axis).

After 60 min of irradiation the reaction mixture was analyzed by ¹H NMR. It was found that no conversion of **7** to **9** took place under the reaction conditions and therefore no rate could be determined.

SUMMARY OF KINETIC DATA FOR [4+2] CYCLOADDITION REACTION

Table S18. Summary of the kinetic data obtained for the [4+2] cycloaddition reaction.

	*Conversion to **12** (%)*	*k (M^{-1} s^{-1})*
**[2a] BF_4**	<5	$1 \cdot 10^{-7}\ M\ s^{-1}$
**[3a] BF_4**	16	$6 \cdot 10^{-6}\ M\ s^{-1}$
**[4d] BF_4**	38	$2 \cdot 10^{-5}\ M\ s^{-1}$
**Triangulenium Ion**	0	-
**$Os(terpy)_2$**	0	-
**$Ru(bpy)_3$**	0	-

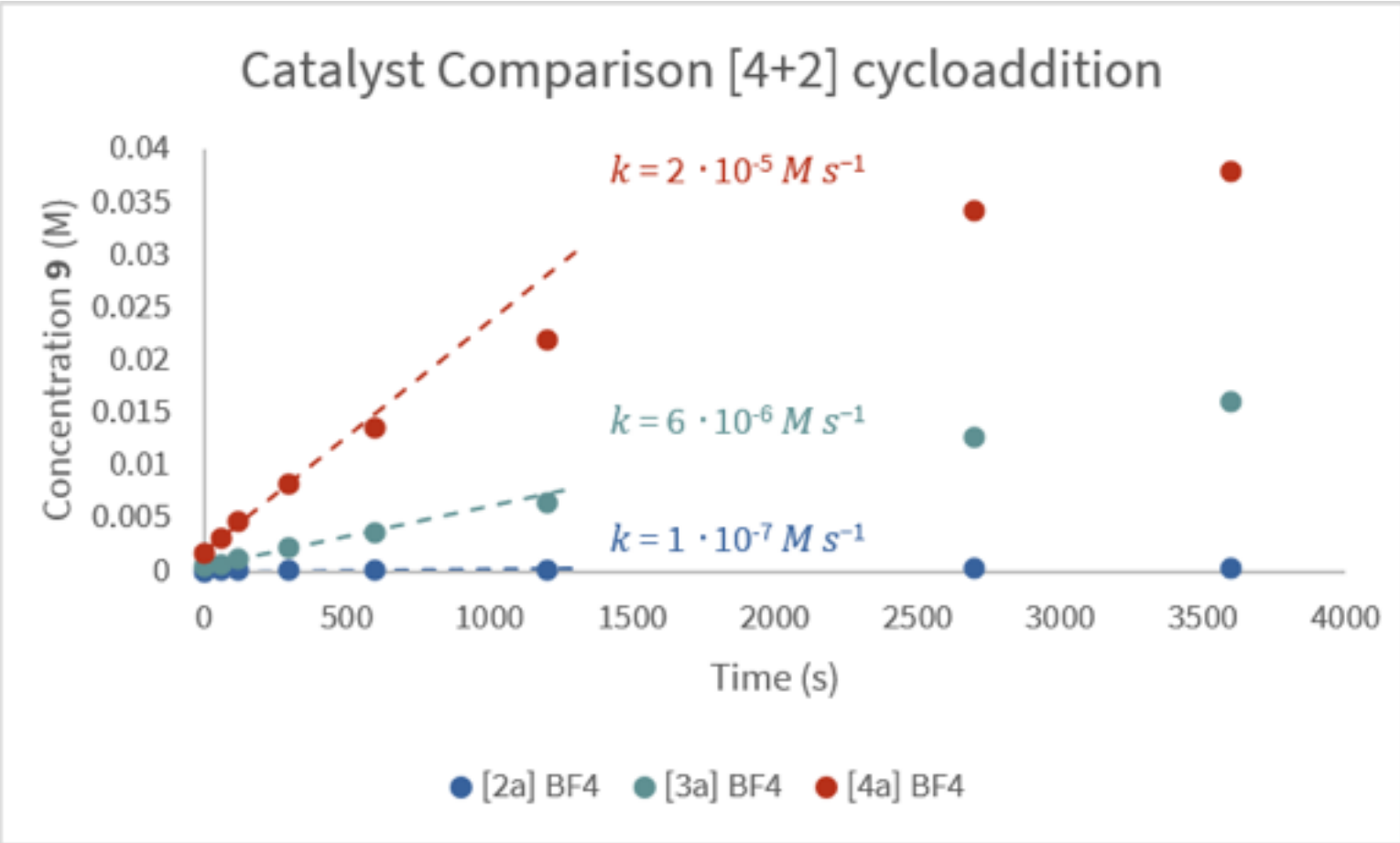

Figure S112. Comparison of rates obtained for the different photocatalysts in the C–H amination reaction.

C–H amination reaction at 0.10 mmol scale catalyzed by photocatalysts

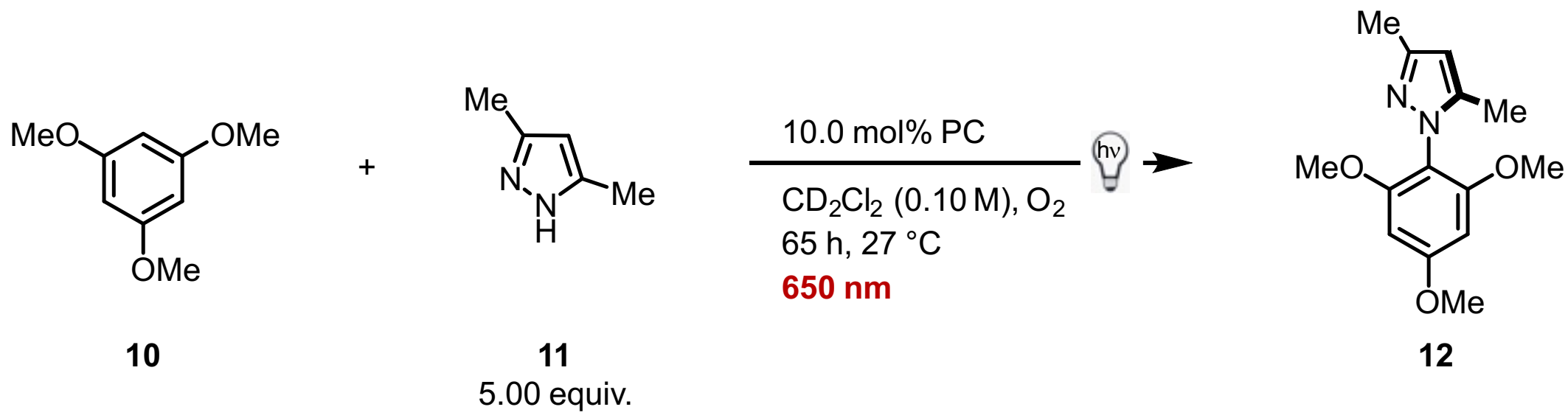

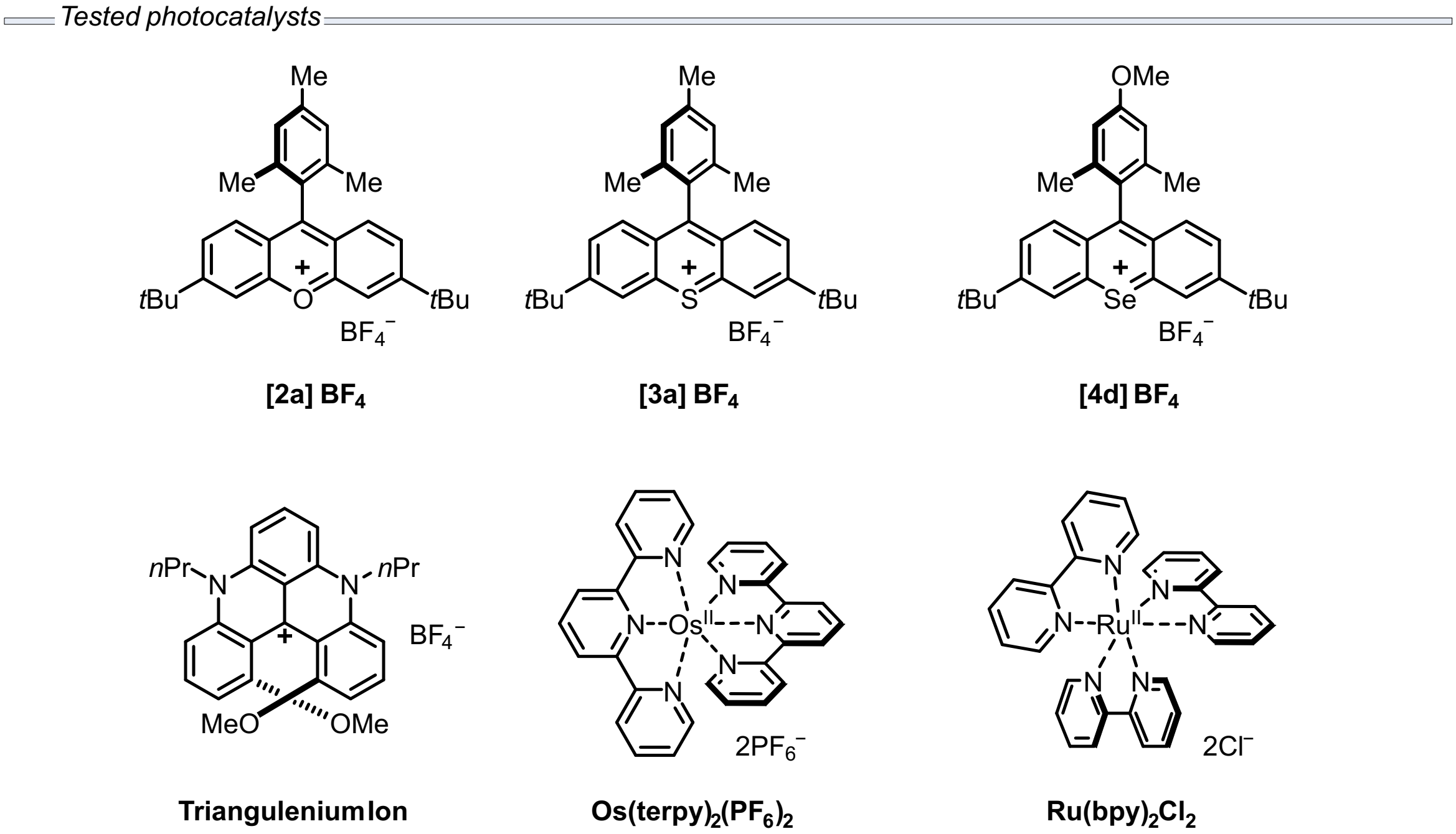

To an NMR tube, trimethoxybenzene **10** (60 µmol, 1.00 equiv.), pyrazole **11** (300 µmol, 5.00 equiv.) and photocatalyst (6 µmol, 0.10 equiv.) were added. The headspace of the tube was flushed with an O_2 balloon for 5 min. Separately, CD_2Cl_2 was sparged with an O_2 balloon for 5 min. Then, oxygenated CD_2Cl_2 (0.60 mL) was added to the NMR tube using a 1.0-mL syringe under oxygen shower. The tube was capped, and the mixture was analyzed by ^{1}H NMR spectroscopy (see for example **Figure S113**, top panel). The NMR tube cap was sealed with parafilm and irradiated with red-light (red-light irradiation, λ_{max} = 650 nm). After the specified time intervals, irradiation was stopped, reaction progress was monitored by ^{1}H NMR (8 NMR experiments total with 8 scans per analysis), and irradiation with red-light was continued until a total irradiation time of 66 h was reached (see for example **Figures S114**, bottom panel).

To calculate the conversion of **10** to **12**, the signals due to the aromatic hydrogens of trimethoxybenzene were used (**10**: s, 6.10 ppm, 3H and **12**: s, 6.22 ppm, 2H; see for example **Figure S113**, bottom panel). Since no other signals in the aromatic region were detected, the mass-balance corresponding to **10** and **12** was assumed to be constant. Thus, the final concentrations were obtained by correcting the peak area for the number of hydrogens of **12**, calculating the peak area ratio of **12** to **10** and then referencing the relative amounts to the known starting material concentration of **10** (0.10 M).

^{1}H NMR spectroscopy of C–H amination reaction catalyzed by [2a] BF_4

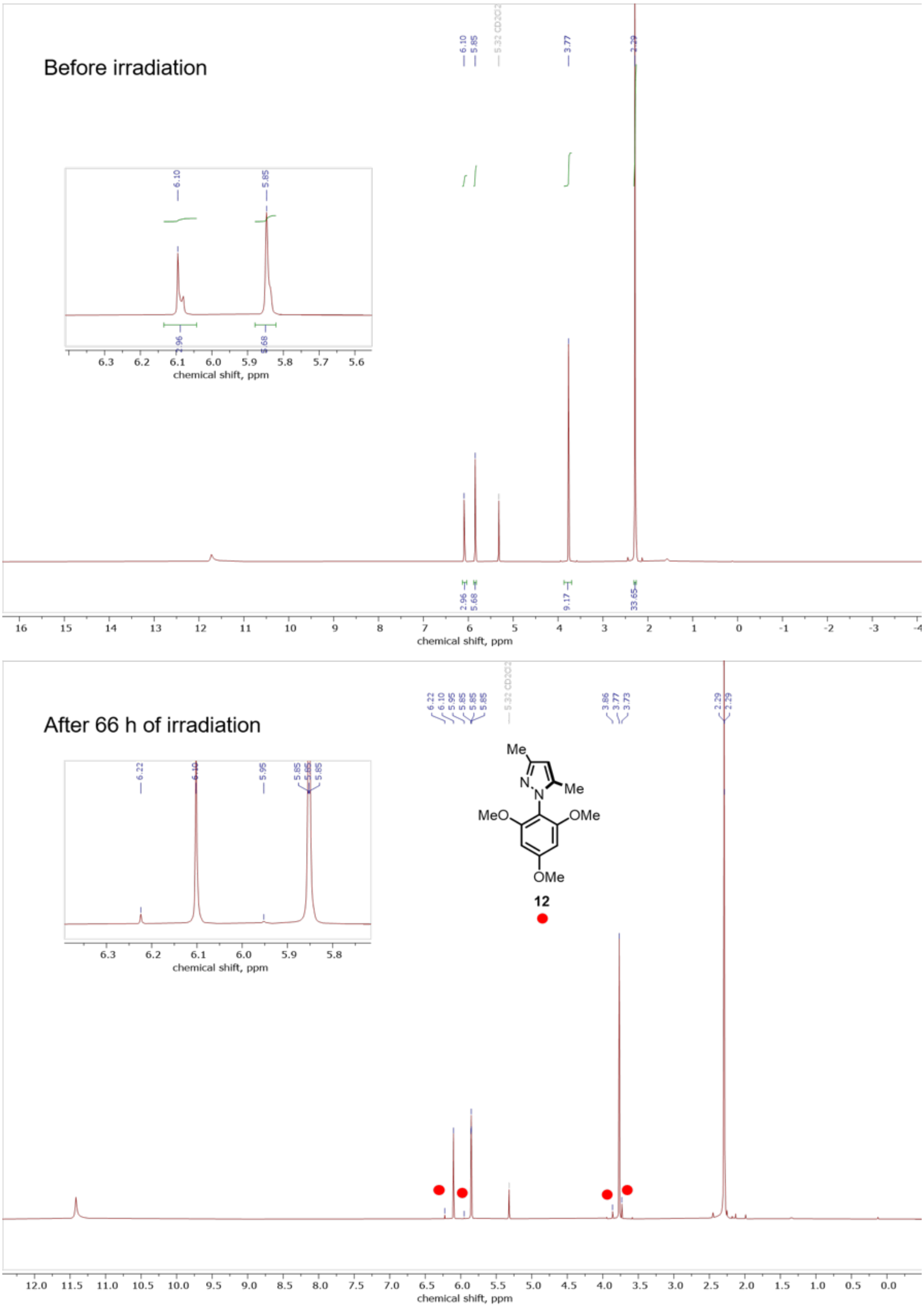

Figure S113. ^{1}H NMR spectrum of reaction mixture with **[2a] BF₄** in CD_2Cl_2 before irradiation (top) and after irradiation for 66 h (bottom).

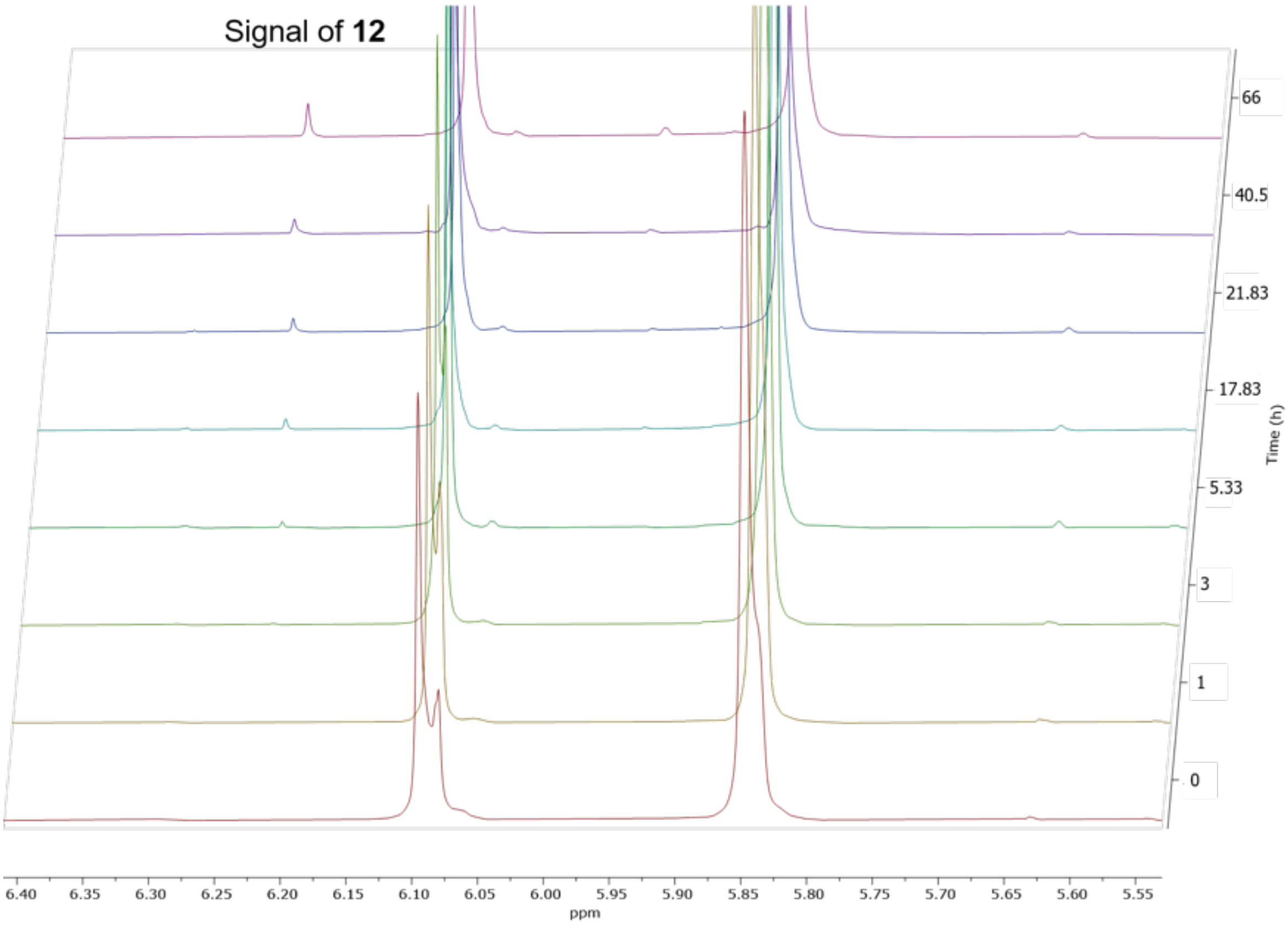

Figure S114. ^{1}H NMR spectra (x-axis) of reaction mixture after different irradiation times (y-axis).

Reaction profile of [2a] BF_4 catalyzed C–H amination reaction

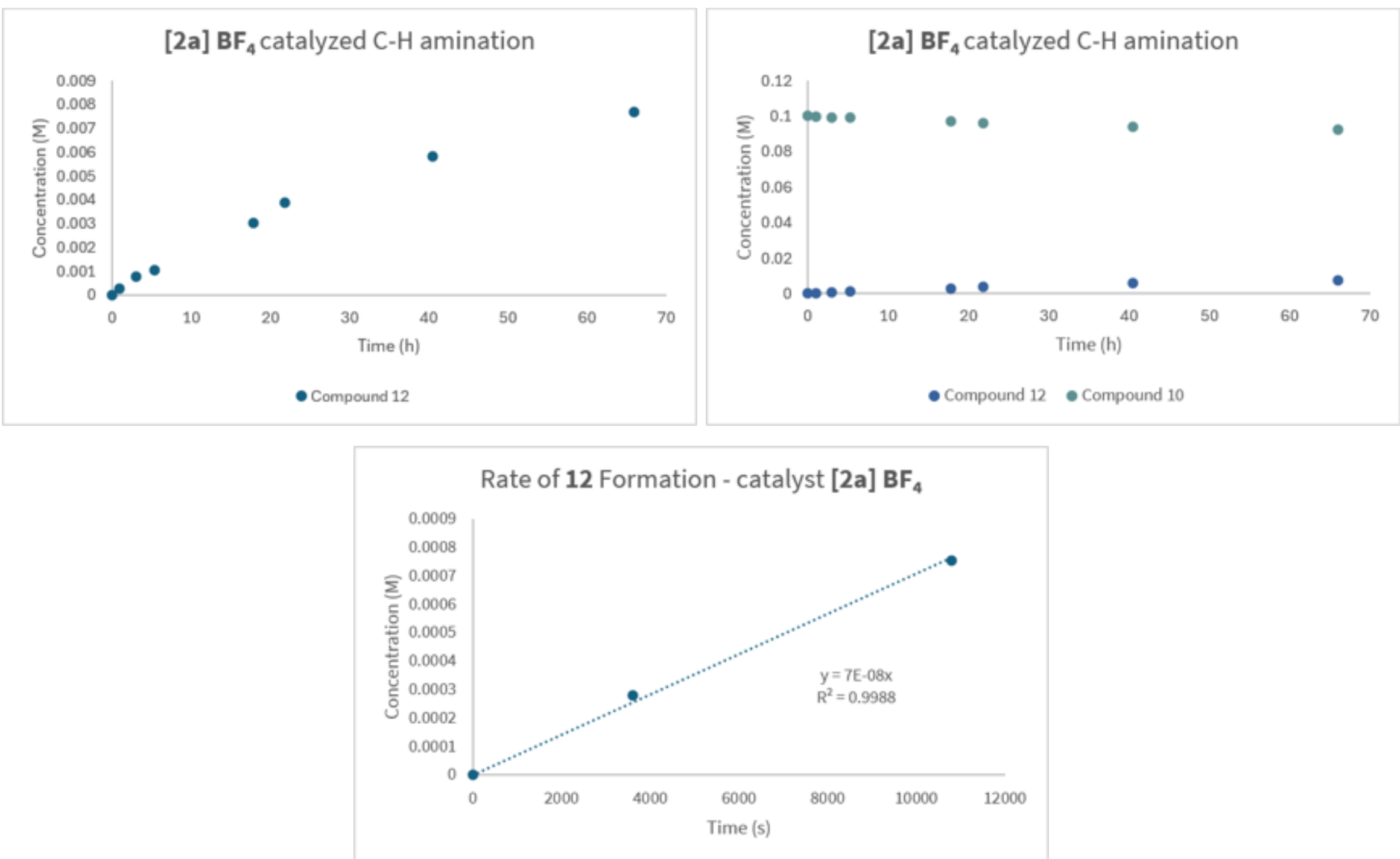

Figure S115. Reaction profiles for **[2a] BF_4** catalyzed C–H amination reaction (top left, top right). Initial rate of the reaction was determined by the slope of the linear fit (bottom).

After 66 h of irradiation the reaction mixture was analyzed by ^{1}H NMR. **10** and **12** were found to be present in relative amounts of 92% and 8%, respectively. The rate of the reaction was determined by applying a linear fit to the first three concentrations determined by ^{1}H NMR after 5.33 h total irradiation time. The resulting initial rate was found to be:

$k_{2a} = 7 \cdot 10^{-8}\, M\, s^{-1}$.

^{1}H NMR spectroscopy of C–H amination reaction catalyzed by [3a] BF$_4$

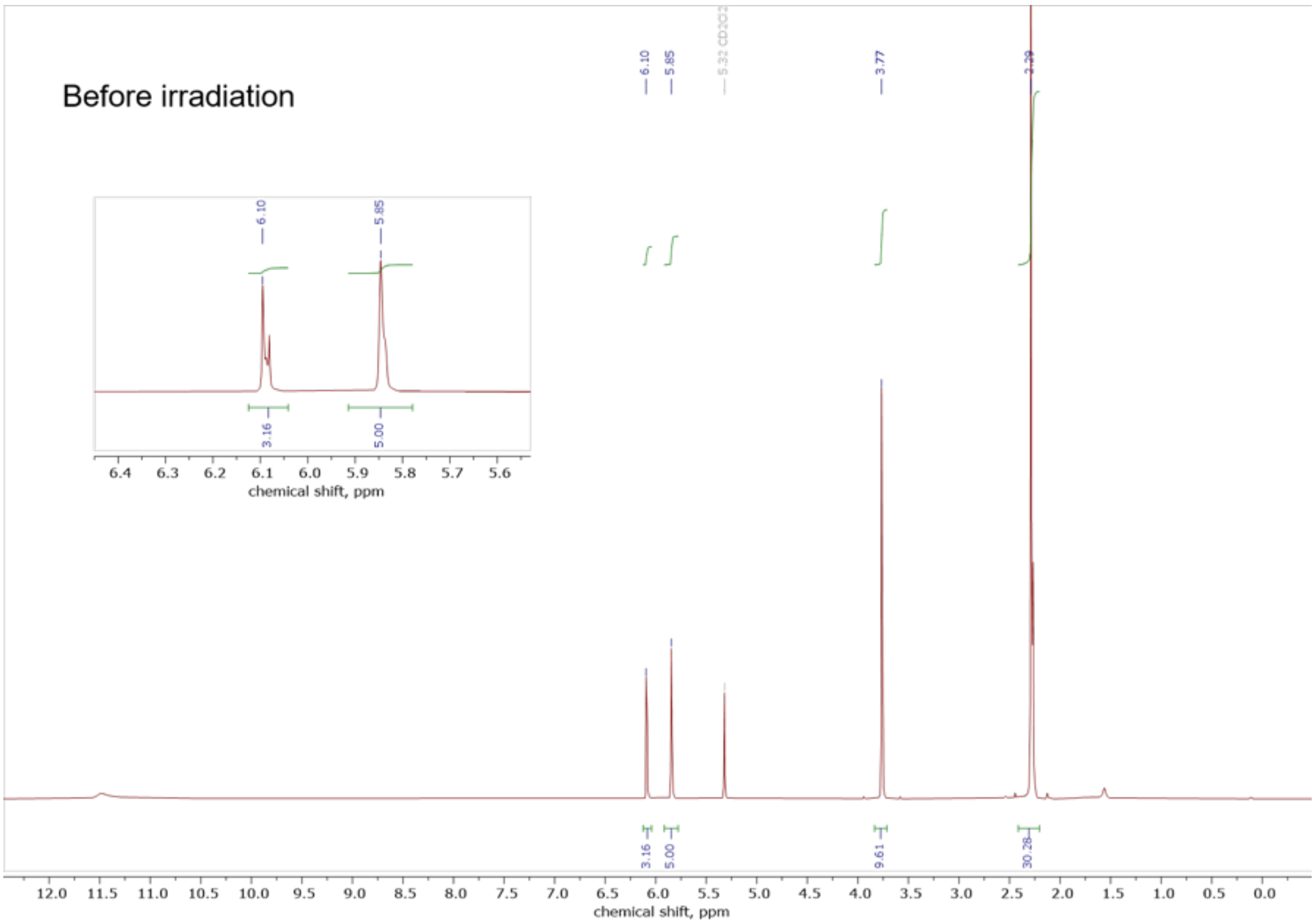

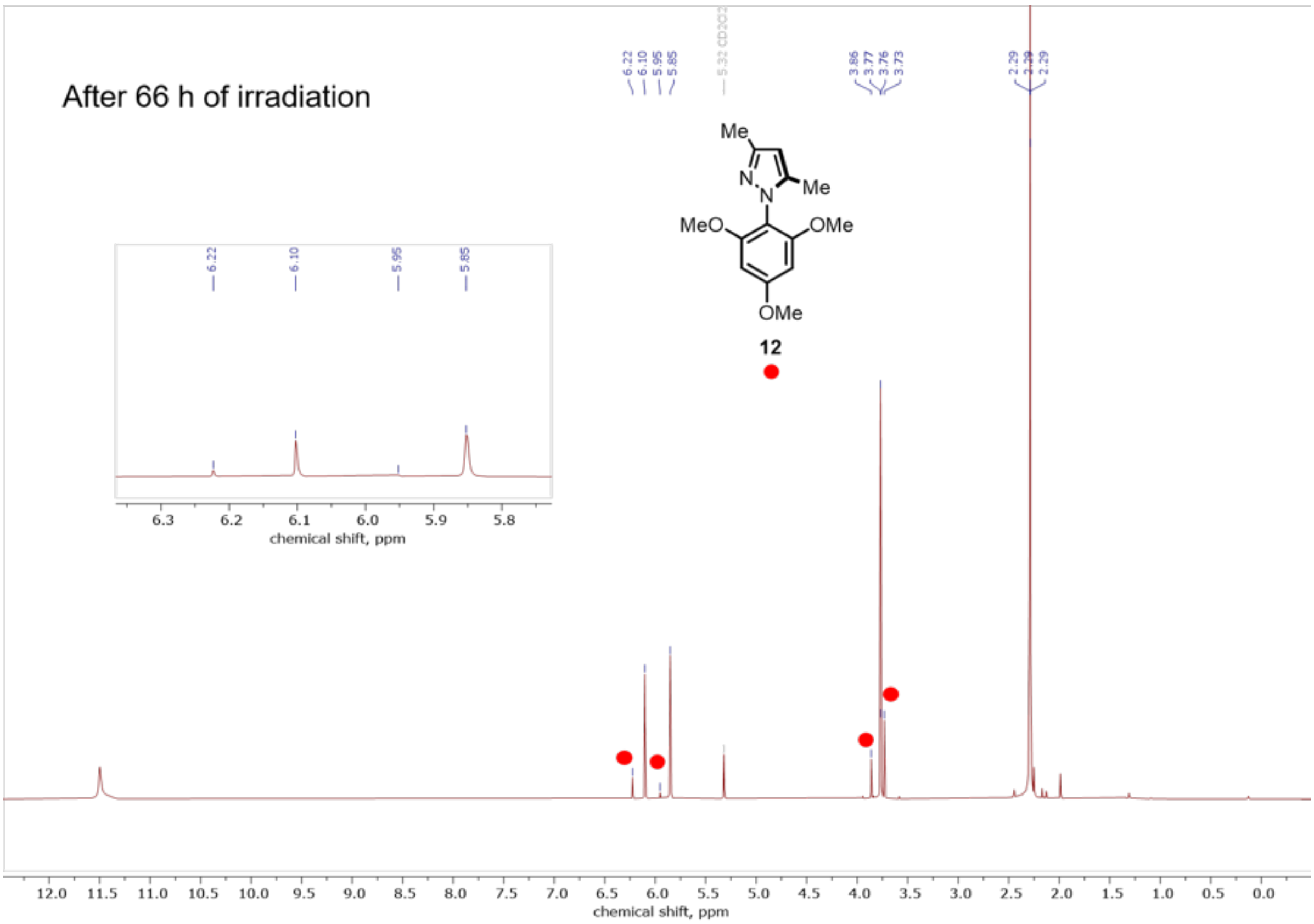

Figure S116. ^{1}H NMR spectrum of reaction mixture with **[3a] BF$_4$** in CD_2Cl_2 before irradiation (top) and after irradiation for 66 h (bottom).

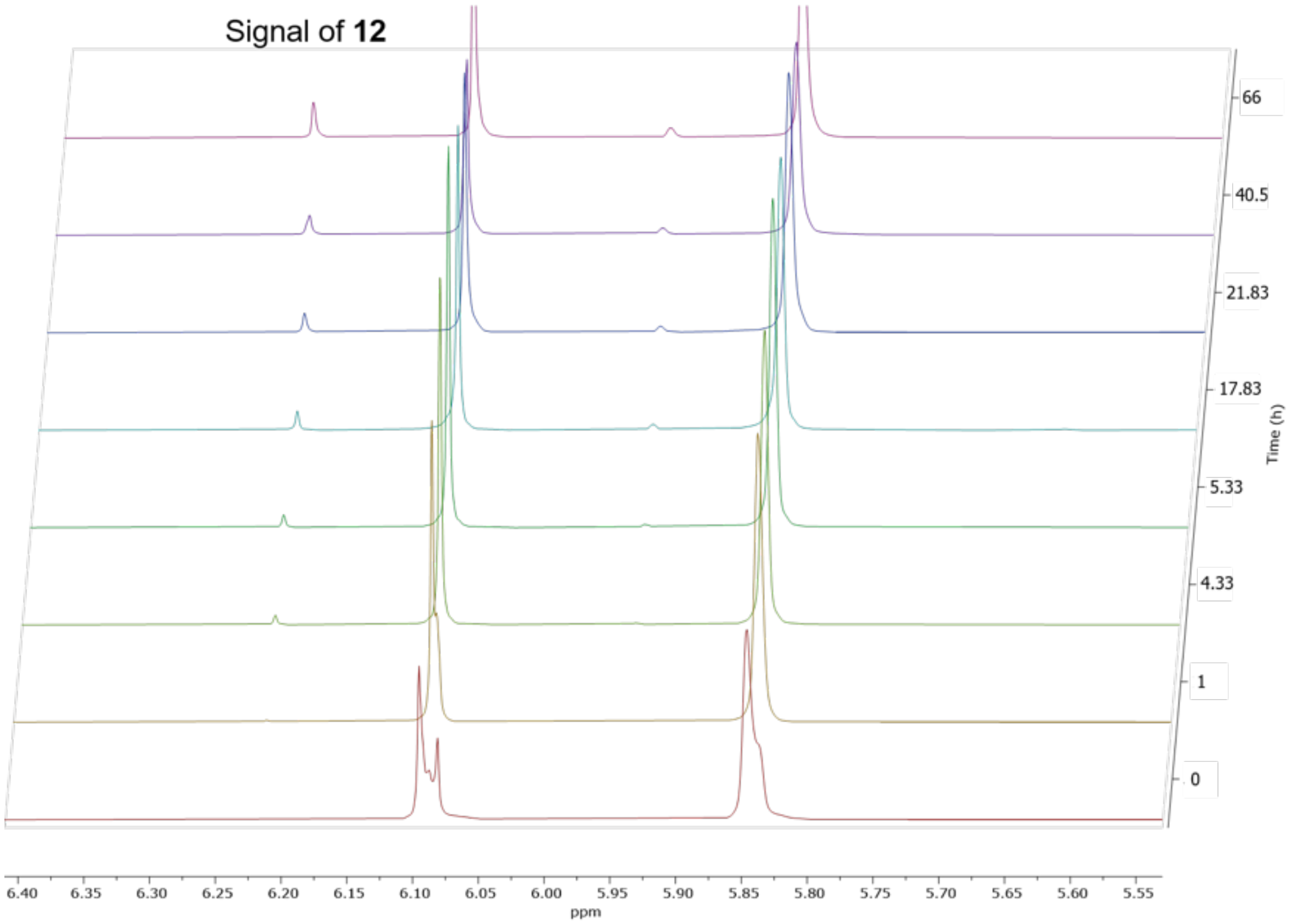

Figure S117. ^{1}H NMR spectra (x-axis) of reaction mixture after different irradiation times (y-axis).

Reaction profile of [3a] BF_4 catalyzed C–H amination reaction

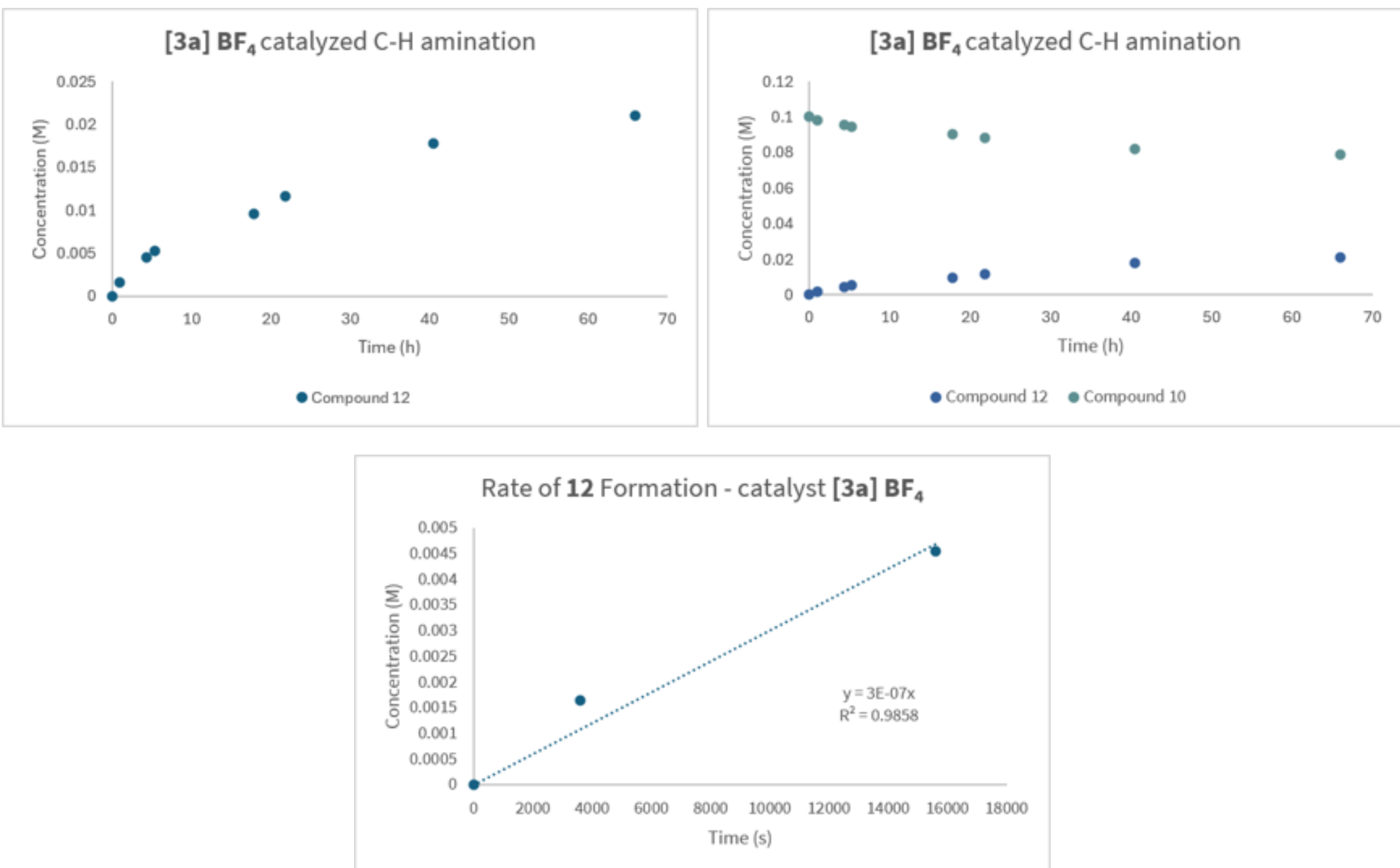

Figure S118. Reaction profiles for **[3a] BF_4** catalyzed C–H amination reaction (top left, top right). Initial rate of the reaction was determined by the slope of the linear fit (bottom).

After 66 h of irradiation the reaction mixture was analyzed by ^{1}H NMR. **10** and **12** were found to be present in relative amounts of 79% and 21%, respectively. The rate of the reaction was determined by applying a linear fit to the first three concentrations determined by ^{1}H NMR after 5.33 h total irradiation time. The resulting initial rate was found to be:

$k_{3a} = 3 \cdot 10^{-7}\ M\ s^{-1}$.

^{1}H NMR spectroscopy of C–H amination reaction catalyzed by [4d] BF_4

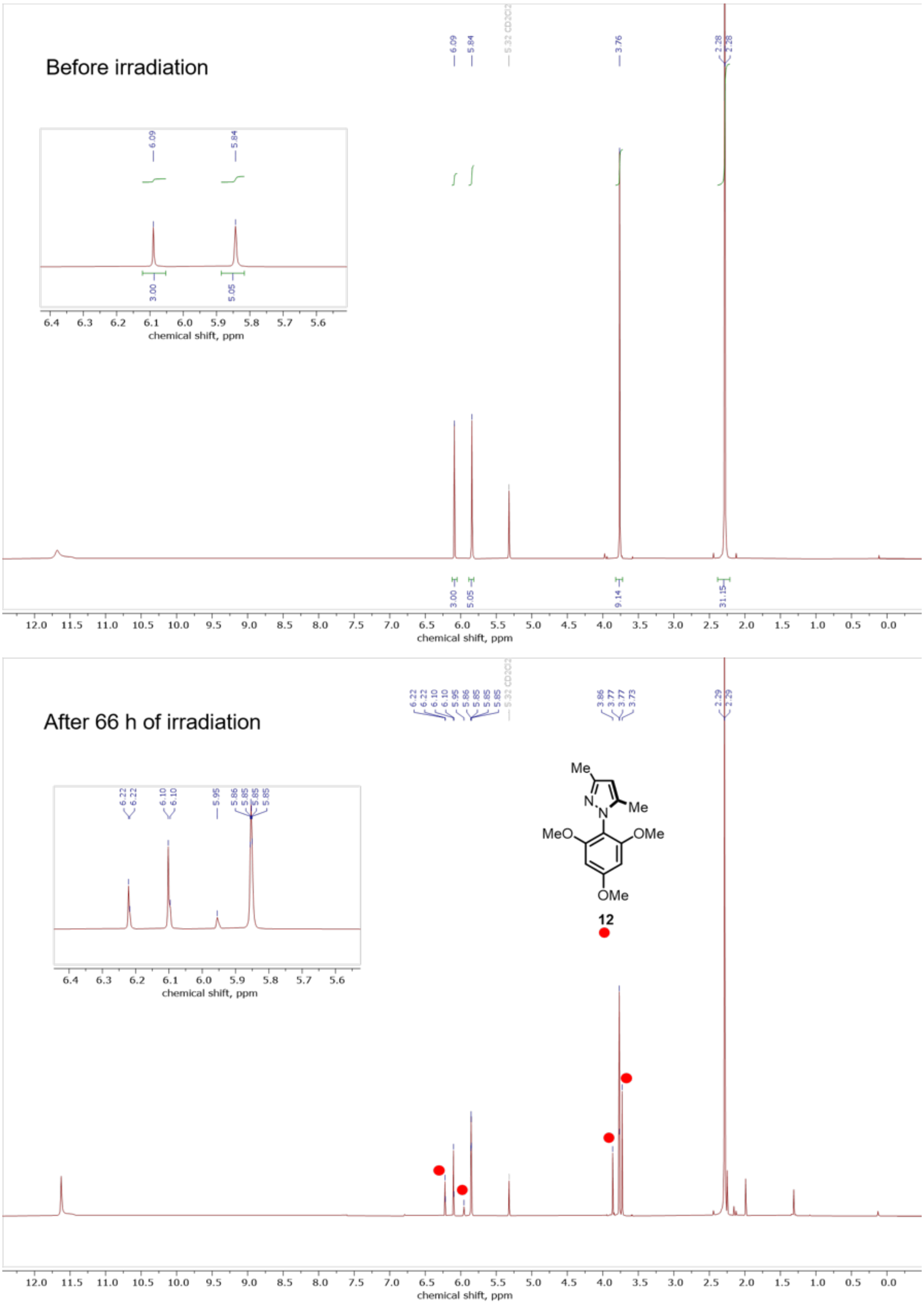

Figure S119. ^{1}H NMR spectrum of reaction mixture with **[4d] BF_4** in CD_2Cl_2 before irradiation (top) and after irradiation for 66 h (bottom).

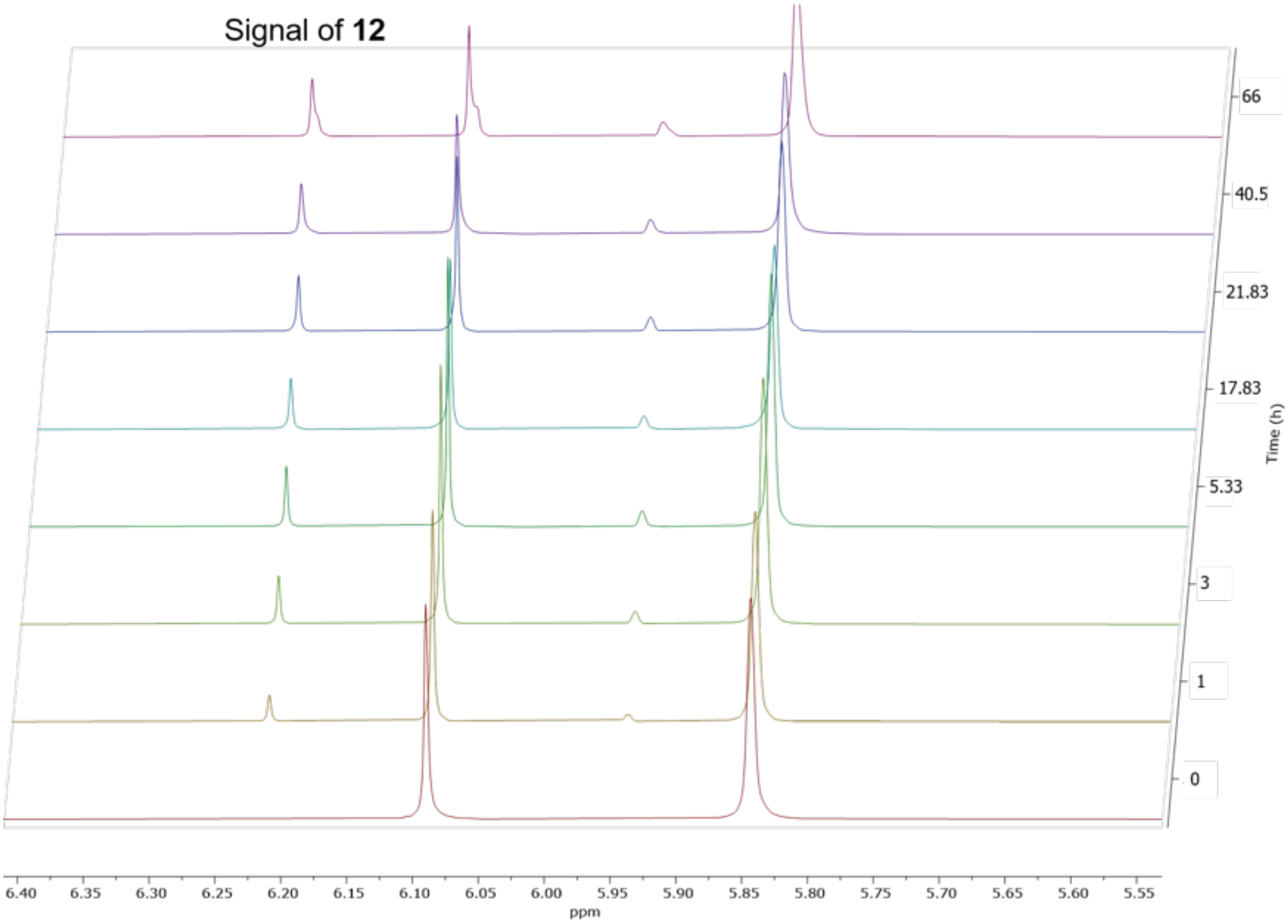

Figure S120. ^{1}H NMR spectra (x-axis) of reaction mixture after different irradiation times (y-axis).

Reaction profile of [4d] BF_4 catalyzed C–H amination reaction

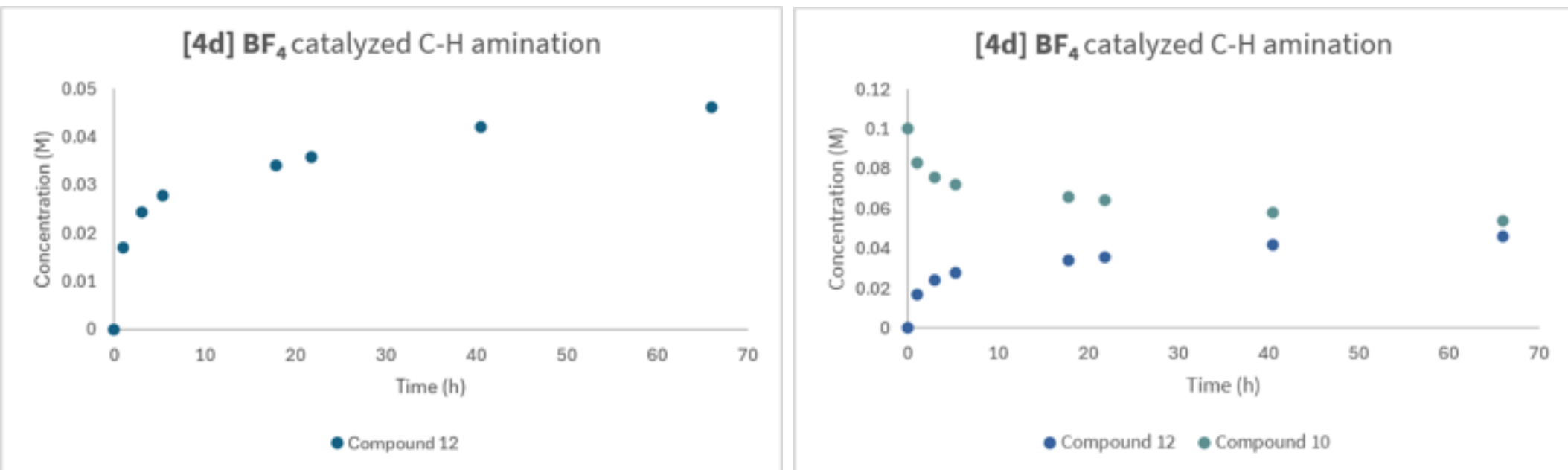

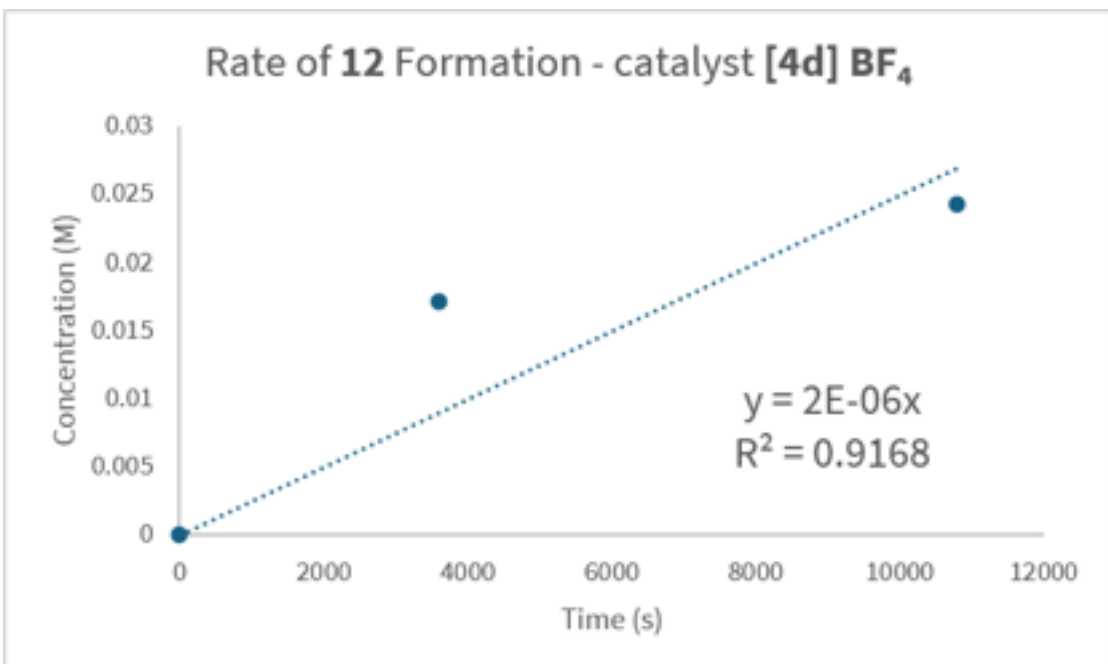

Figure S121. Reaction profiles for **[4d] BF_4** catalyzed C–H amination reaction (top left, top right). Initial rate of the reaction was determined by the slope of the linear fit (bottom).

After 66 h of irradiation the reaction mixture was analyzed by ^{1}H NMR. **10** and **12** were found to be present in relative amounts of 54% and 46%, respectively. The rate of the reaction was determined by applying a linear fit to the first three concentrations determined by ^{1}H NMR after 5.33 h total irradiation time. The resulting initial rate was found to be:

$k_{4d} = 2 \cdot 10^{-6}\ M\ s^{-1}$.

^{1}H NMR spectroscopy of C–H amination reaction catalyzed by Triangulenium Ion

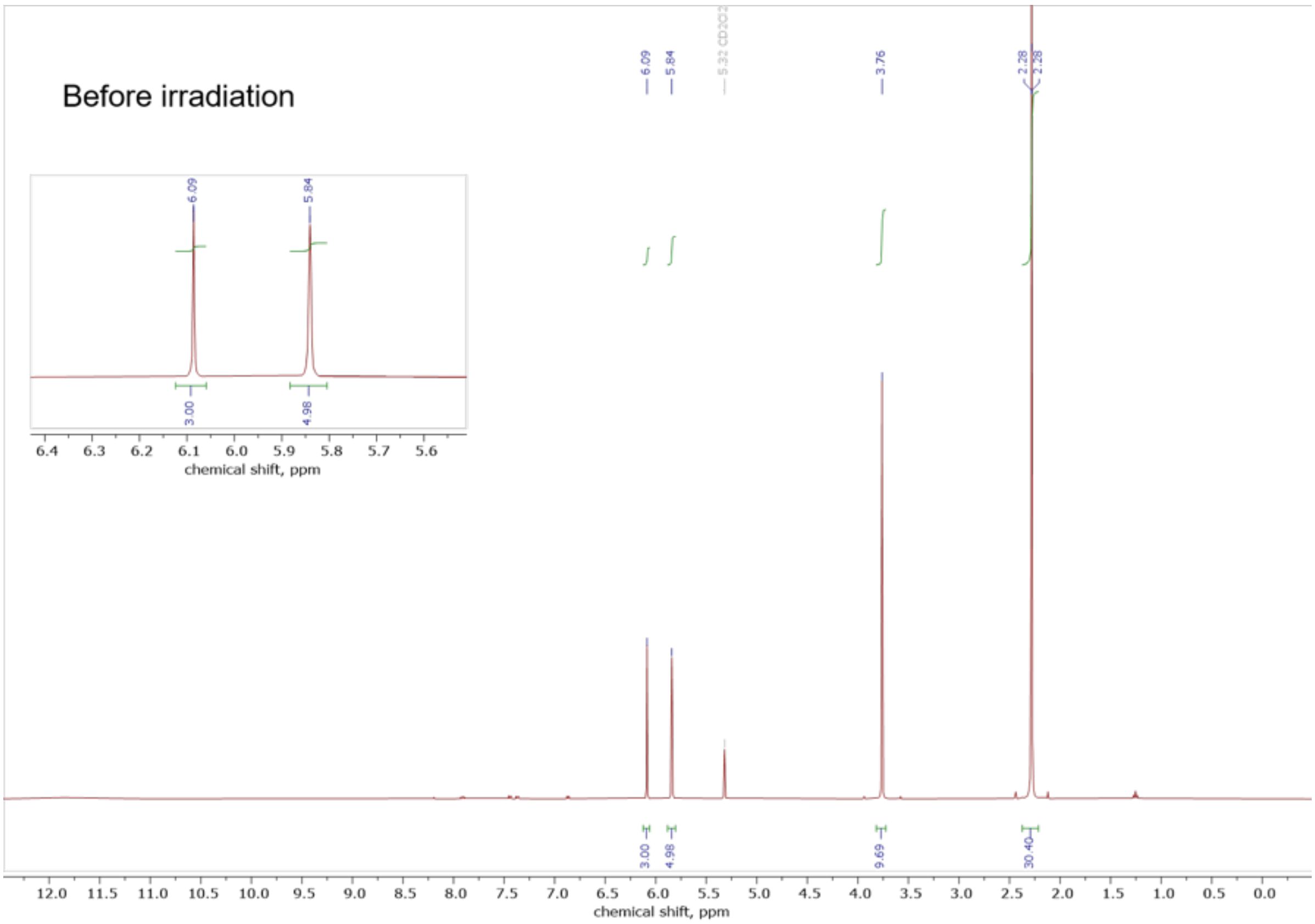

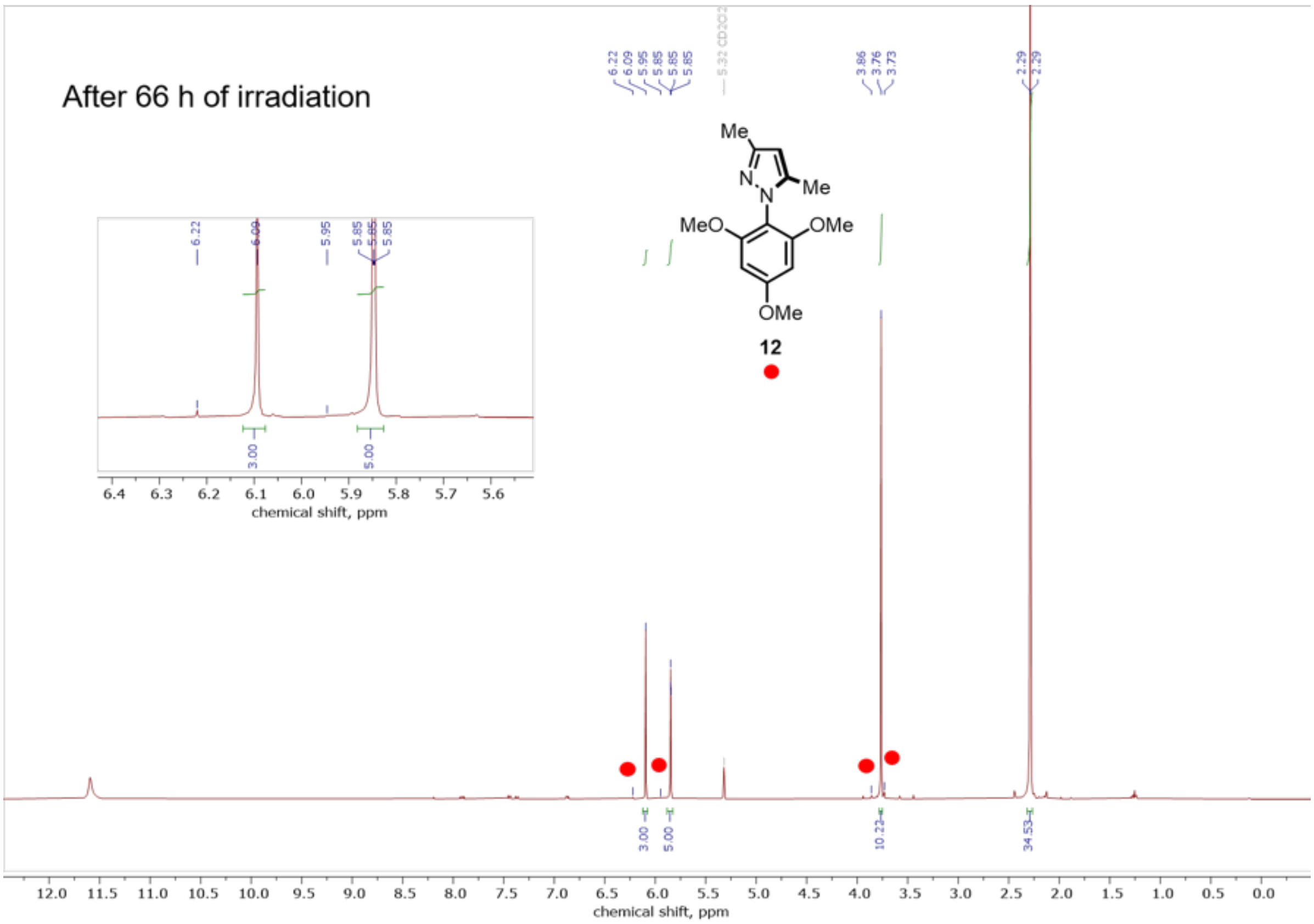

Figure S122. ^{1}H NMR spectrum of reaction mixture with **Triangulenium Ion** in CD_2Cl_2 before irradiation (top) and after irradiation for 66 h (bottom).

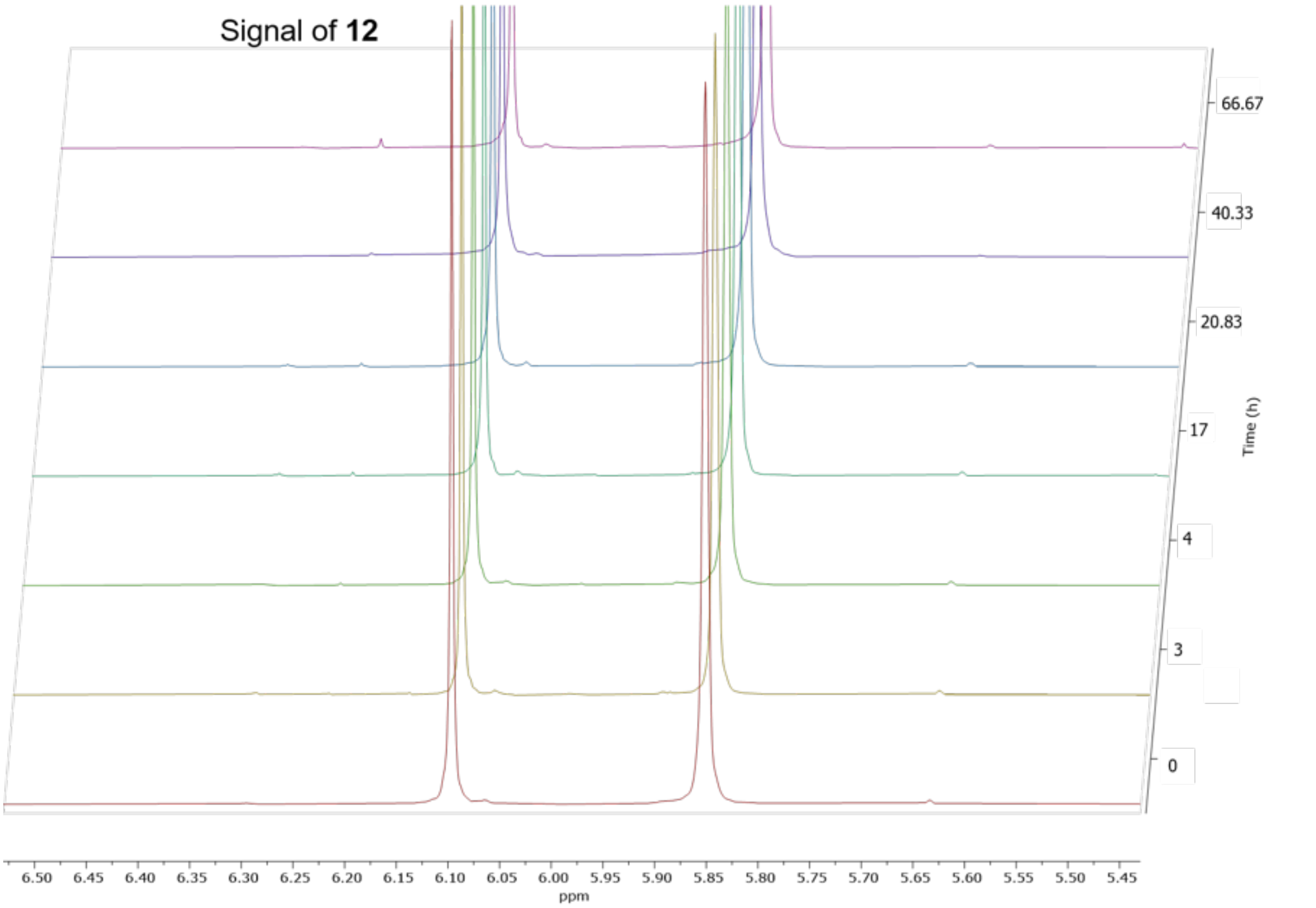

Figure S123. ^{1}H NMR spectra (x-axis) of reaction mixture after different irradiation times (y-axis).

Reaction profile of Triangulenium Ion catalyzed C–H amination reaction

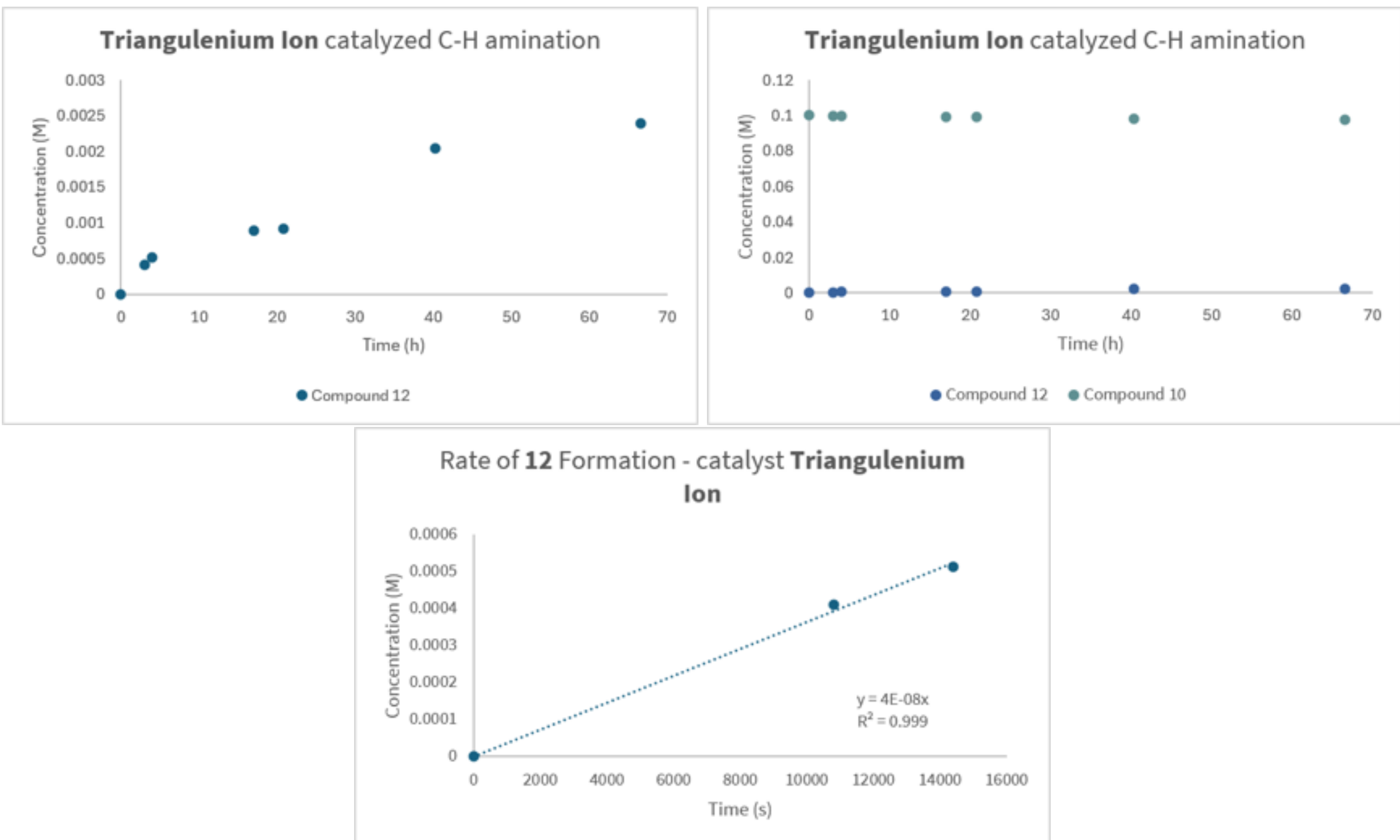

Figure S124. Reaction profiles for **Triangulenium Ion** catalyzed C–H amination reaction (top left, top right). Initial rate of the reaction was determined by the slope of the linear fit (bottom).

After 66 h of irradiation the reaction mixture was analyzed by ^{1}H NMR. **10** and **12** were found to be present in relative amounts of 98% and 2%, respectively. The rate of the reaction was determined by applying a linear fit to the first three concentrations determined by ^{1}H NMR after 5.33 h total irradiation time. The resulting initial rate was found to be:

$k_{Tri} = 4 \cdot 10^{-8}\ M\ s^{-1}$.

^{1}H NMR spectroscopy of C–H amination reaction catalyzed by Os(terpy)$_2$

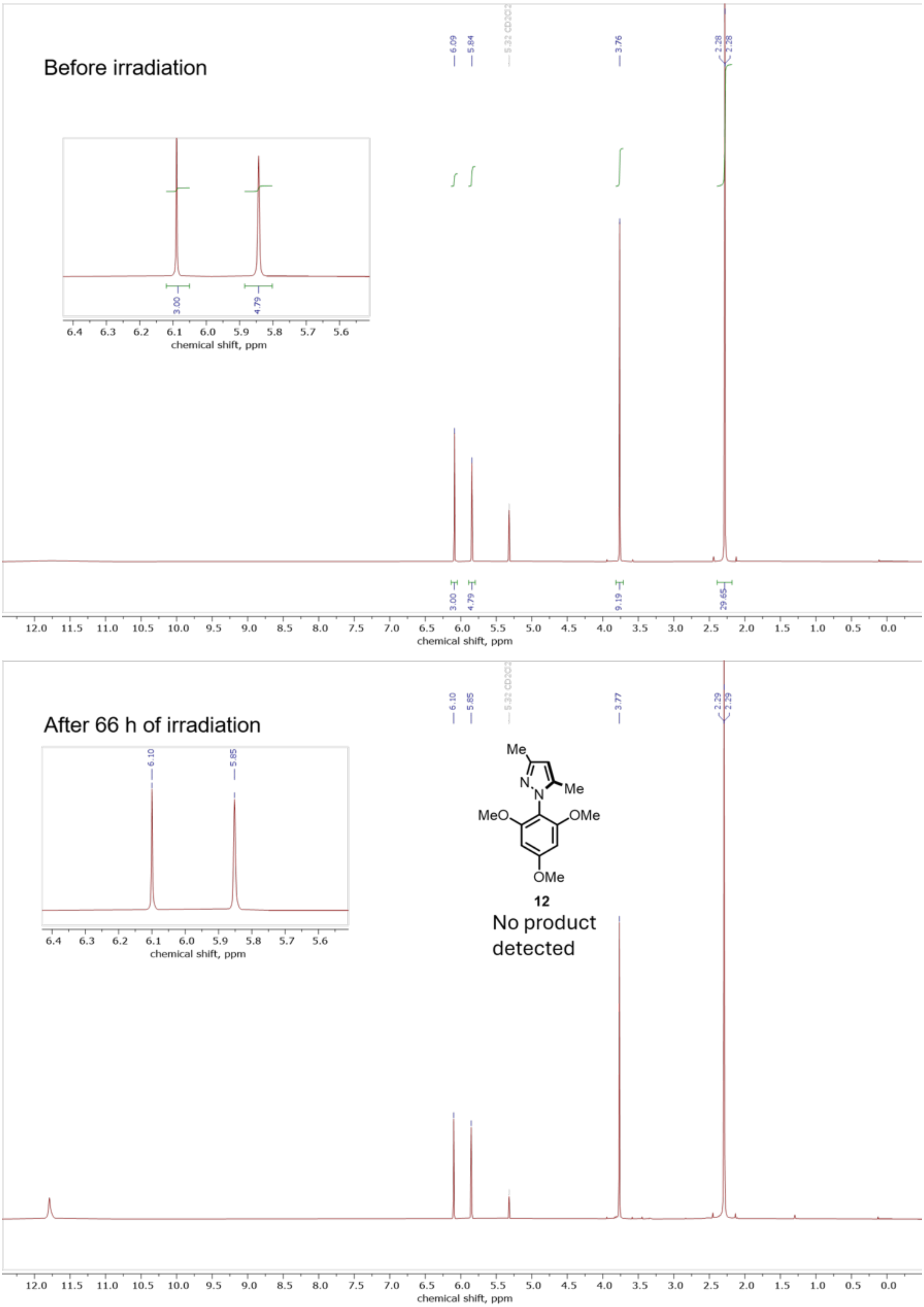

Figure S125. ^{1}H NMR spectrum of reaction mixture with **Os(terpy)$_2$** in CD_2Cl_2 before irradiation (top) and after irradiation for 66 h (bottom).

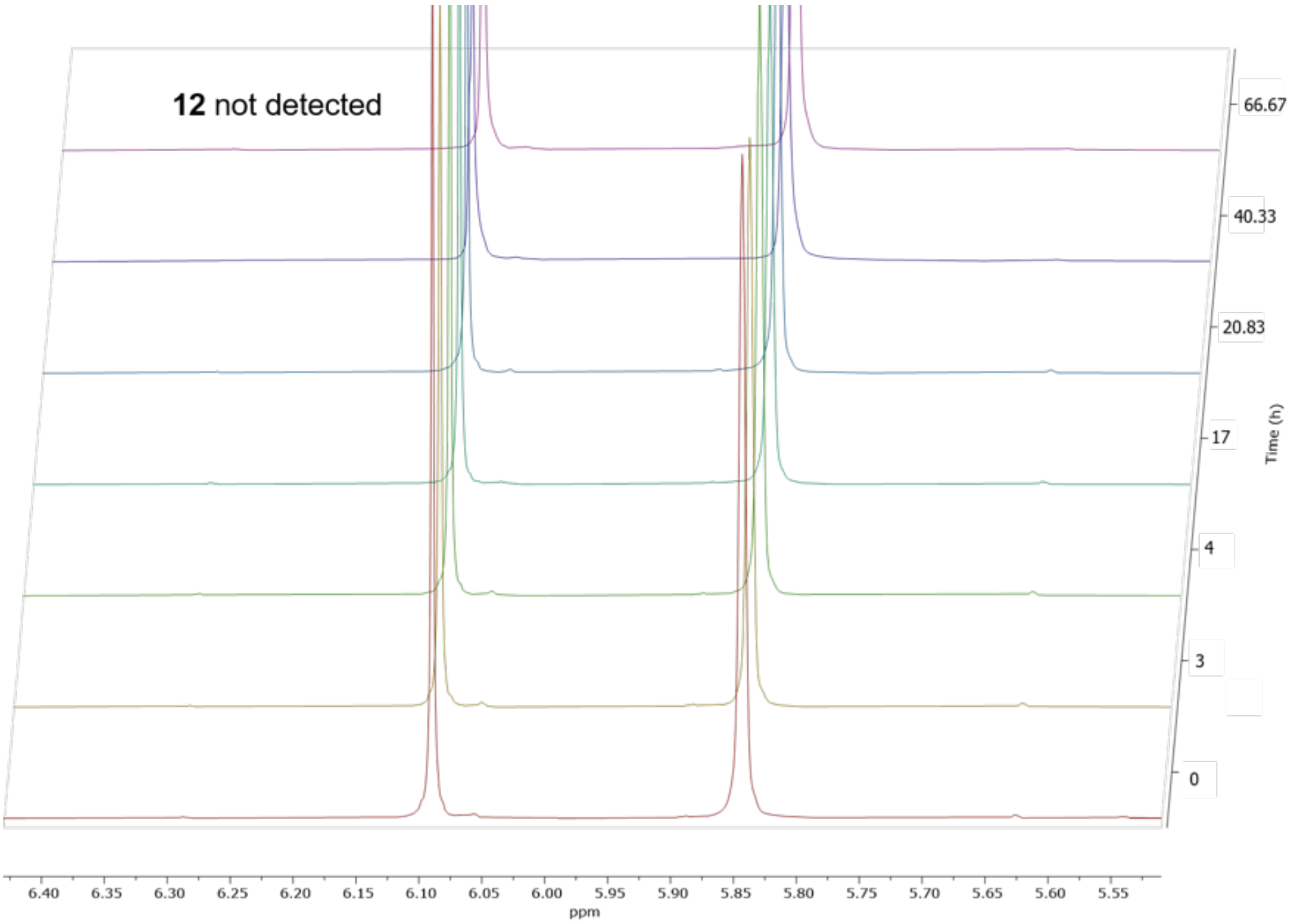

Figure S126. ^{1}H NMR spectra (x-axis) of reaction mixture after different irradiation times (y-axis).

After 66 h of irradiation the reaction mixture was analyzed by ^{1}H NMR. It was found that no conversion of **10** to **12** took place under the reaction conditions and therefore no rate could be determined.

^{1}H NMR spectroscopy of C–H amination reaction catalyzed by Ru(bpy)3

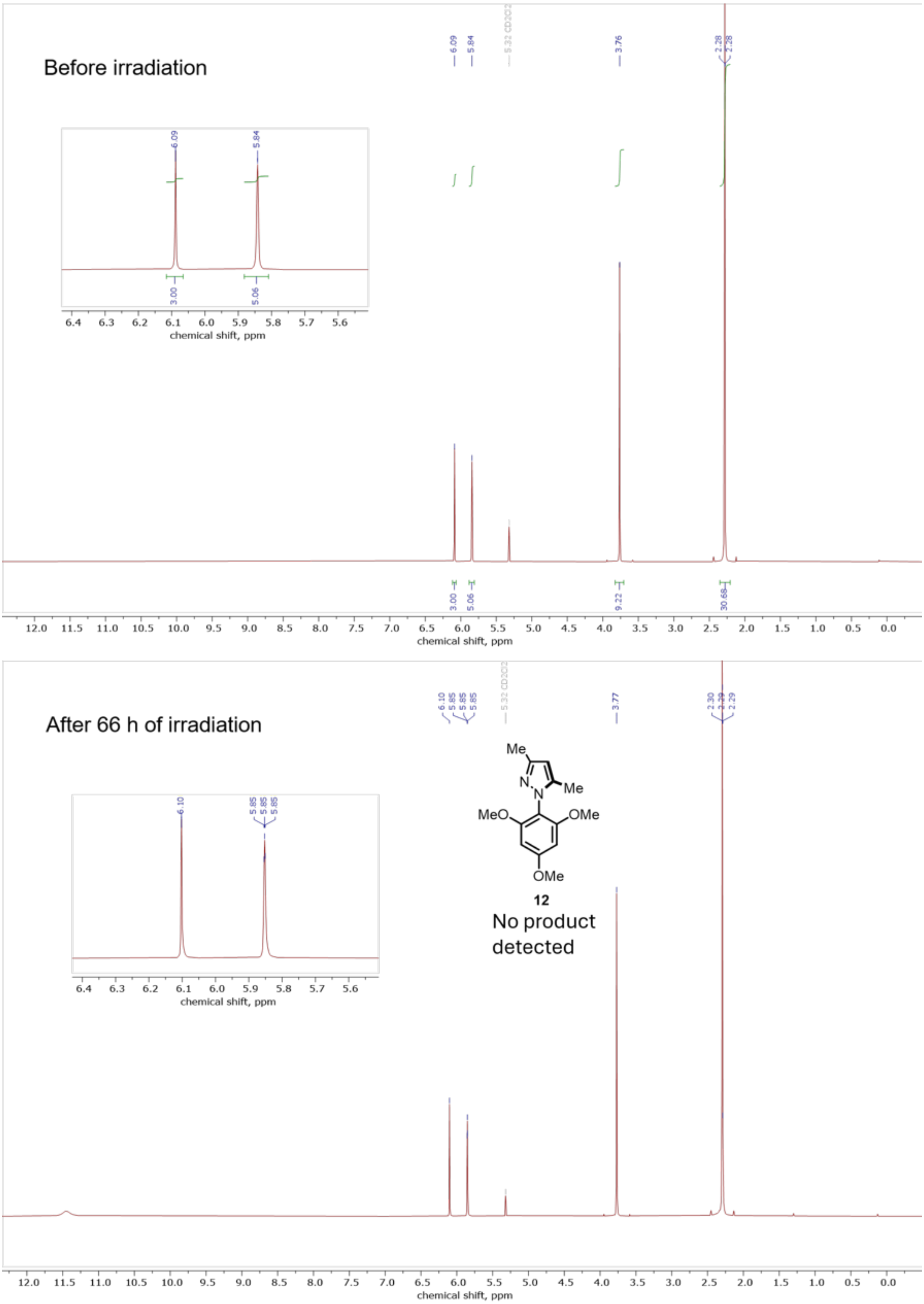

Figure S127. ^{1}H NMR spectrum of reaction mixture with **Ru(bpy)3** in CD_2Cl_2 before irradiation (top) and after irradiation for 66 h (bottom).

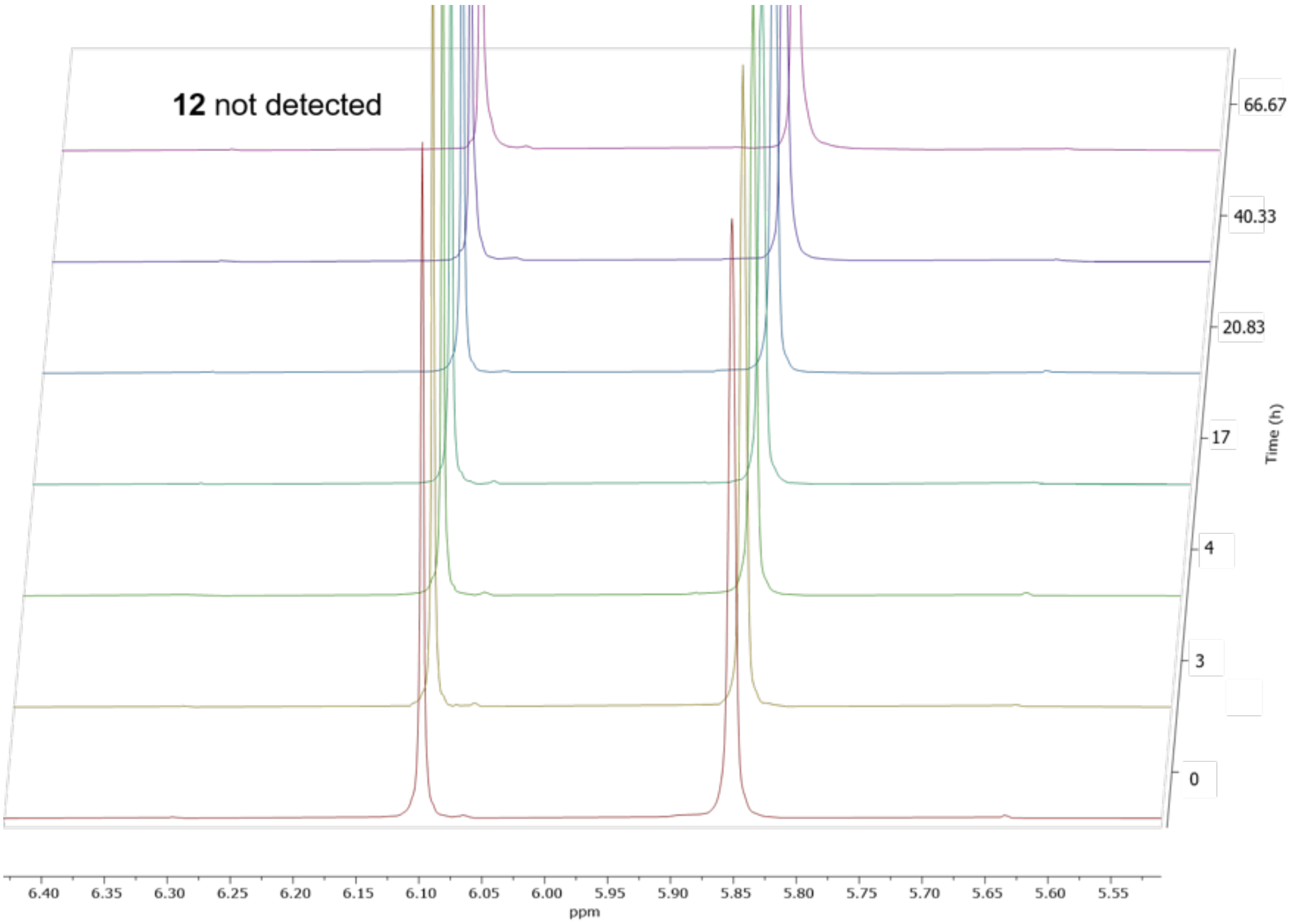

Figure S128. ^{1}H NMR spectra (x-axis) of reaction mixture after different irradiation times (y-axis).

After 66 h of irradiation the reaction mixture was analyzed by ^{1}H NMR. It was found that no conversion of **10** to **12** took place under the reaction conditions and therefore no rate could be determined.

SUMMARY OF KINETIC DATA FOR C–H AMINATION REACTION

Table S19. Summary of the kinetic data obtained for the C–H amination reaction.

	*Conversion to **12** (%)*	*k (M^{-1} s^{-1})*
**[2a] BF_4**	8	$7 \cdot 10^{-8}\ M\ s^{-1}$
**[3a] BF_4**	21	$3 \cdot 10^{-7}\ M\ s^{-1}$
**[4d] BF_4**	46	$2 \cdot 10^{-6}\ M\ s^{-1}$
**Triangulenium Ion**	2	$4 \cdot 10^{-8}\ M\ s^{-1}$
**Os(terpy)$_2$**	0	-
**Ru(bpy)$_3$**	0	-

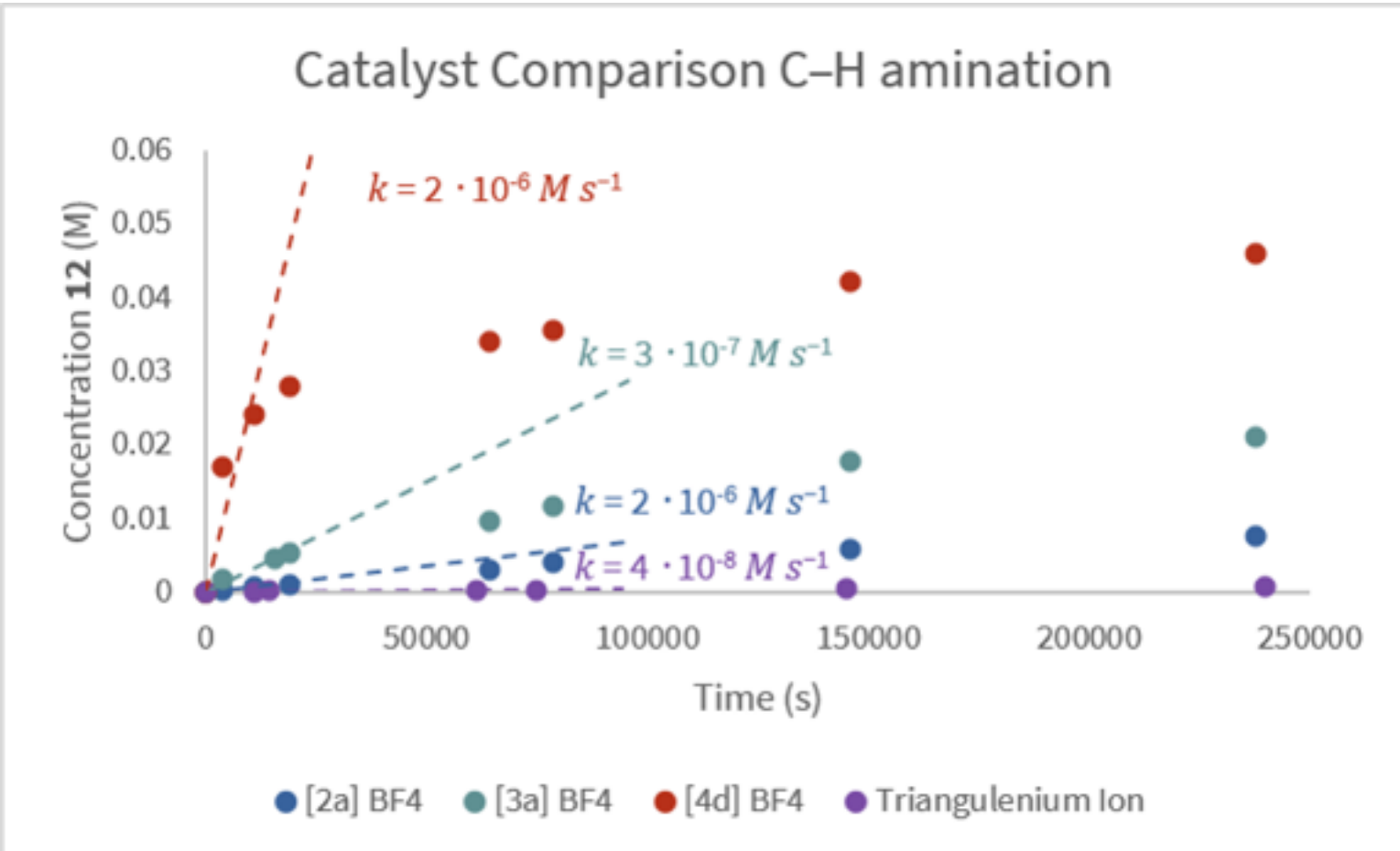

Figure S129. Comparison of rates obtained for the different photocatalysts in the C–H amination reaction.

X-RAY CRYSTALLOGRAPHIC ANALYSIS

SELENOXANTHYLIUM SALT [4A] BF_4 (CCDC 2544588)

Single crystals suitable for X-ray analysis were obtained by slow gas phase diffusion of pentane (excess) into a concentrated CH_2Cl_2 solution of selenoxanthylium salt **[4a] BF_4** (10 mg in 1 mL of CH_2Cl_2) at –20°C for 100 h.

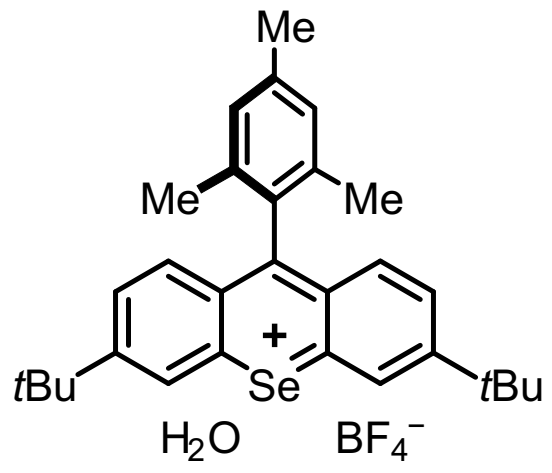

[4a] BF_4·H_2O

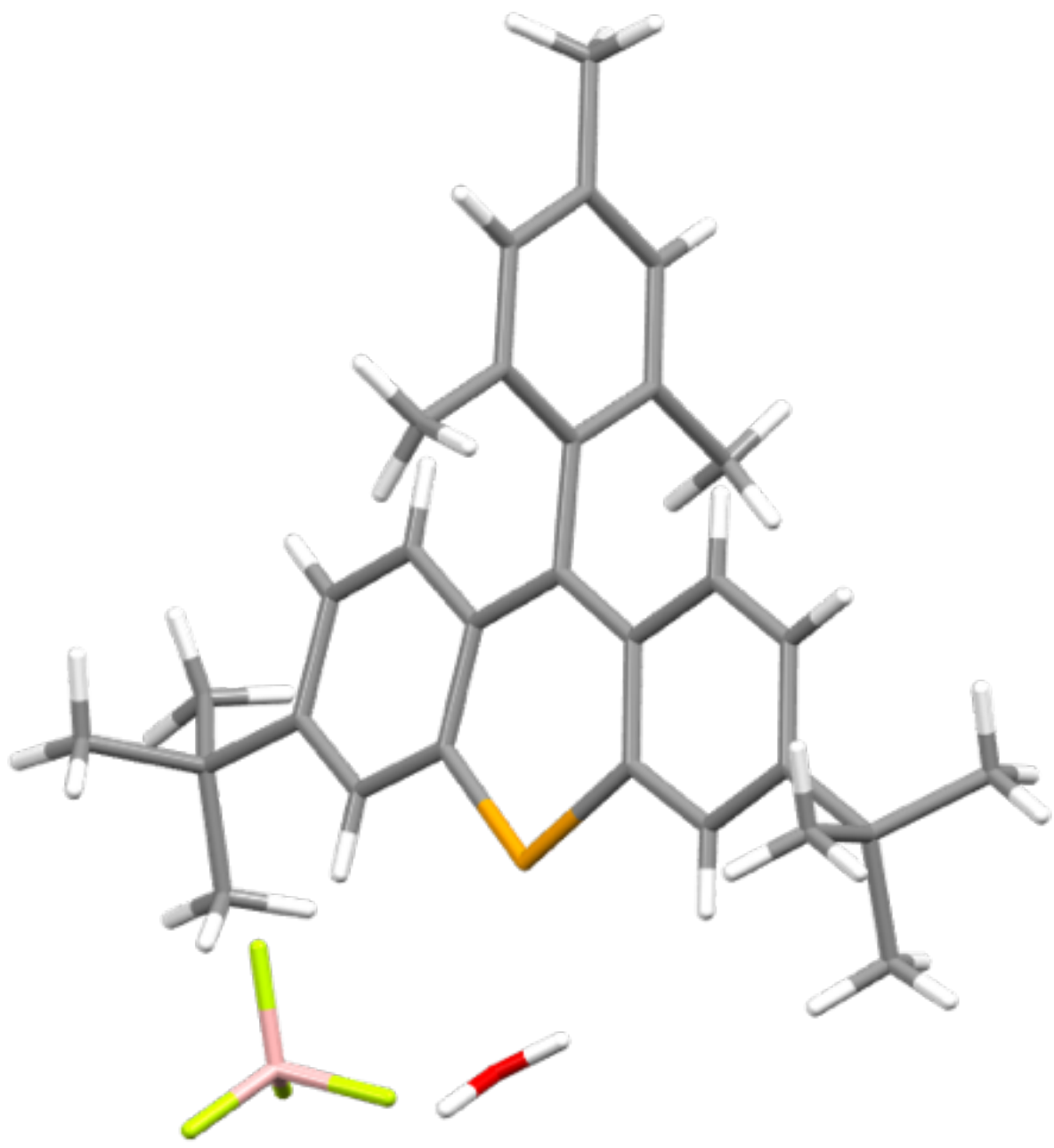

Table S20. Crystal data and structure refinement for **[4a] BF_4**.

Identification code	swu-007_3d_a	
Empirical formula	C30 H35.37 B F4 O0.18 Se	
Formula weight	564.67	
Temperature	100(2) K	
Wavelength	0.71073 Å	
Crystal system	Monoclinic	
Space group	I2/a	
Unit cell dimensions	a = 23.182(5) Å	a= 90°.

	b = 8.3870(17) Å	b= 105.24(3)°.
	c = 29.130(6) Å	g = 90°.
Volume	5464(2) $Å^3$	
Z	8	
Density (calculated)	1.373 Mg/m^3	
Absorption coefficient	1.419 mm^{-1}	
F(000)	2335	
Crystal size	0.100 x 0.060 x 0.020 mm^3	
Theta range for data collection	2.534 to 25.677°.	
Index ranges	-14<=h<=28, -10<=k<=8, -35<=l<=28	
Reflections collected	39798	
Independent reflections	5111 [R(int) = 0.1560]	
Completeness to theta = 25.242°	98.8 %	
Absorption correction	Semi-empirical from equivalents	
Max. and min. transmission	0.9868 and 0.4956	
Refinement method	Full-matrix least-squares on F^2	
Data / restraints / parameters	5111 / 518 / 343	
Goodness-of-fit on F^2	1.028	
Final R indices [I>2sigma(I)]	R1 = 0.0972, wR2 = 0.2273	
R indices (all data)	R1 = 0.1612, wR2 = 0.2743	
Extinction coefficient	n/a	
Largest diff. peak and hole	0.954 and -0.845 e.$Å^{-3}$	

Computational Data

Comparison between TD-DFT and experimental absorption spectra

Xanthylium [2a]+

Optimized structure

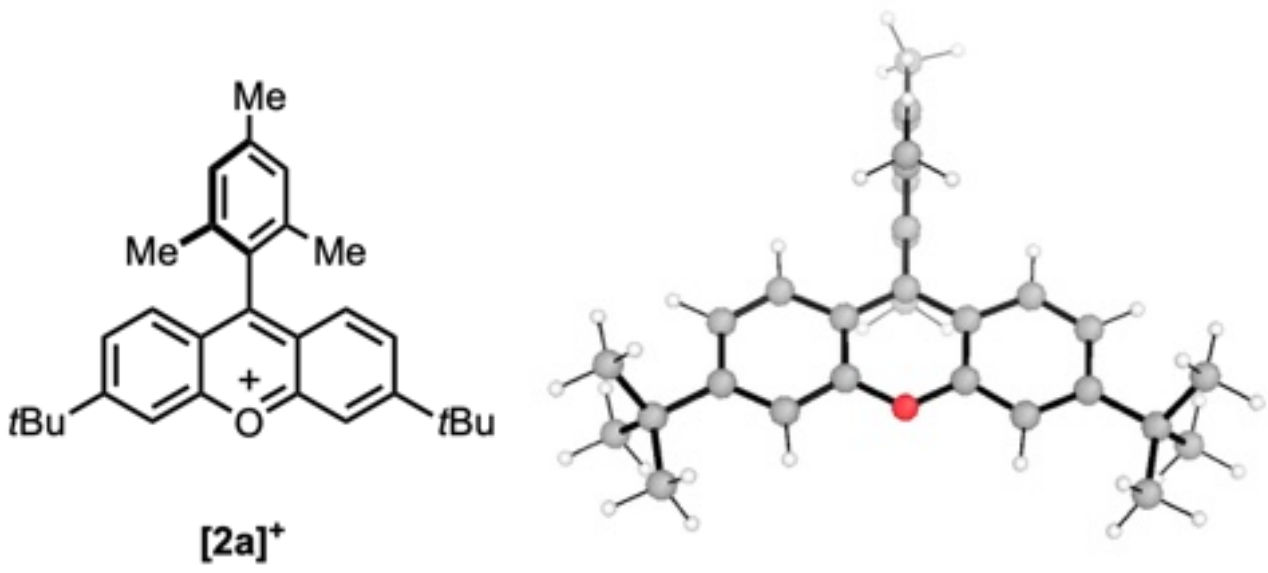

[2a]+

TD-DFT benchmarking

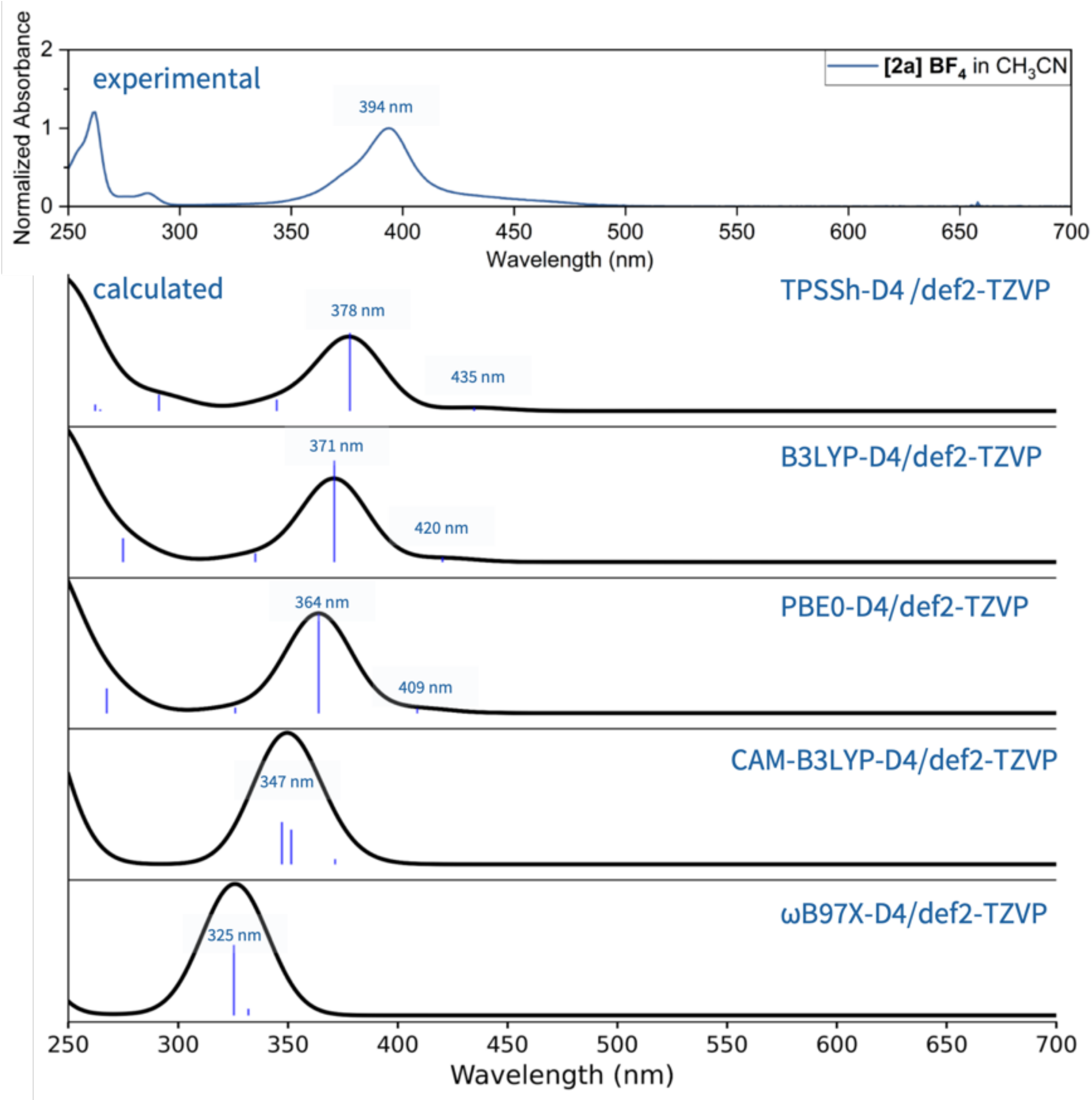

Figure S130. Benchmark of TD-DFT functionals for reproducing the experimental UV–Vis absorption spectrum of **[2a]**$^+$ in CH_3CN. Calculated spectra were obtained at the TPSSh-D4/def2-TZVP, B3LYP-D4/def2-TZVP, PBE0-D4/def2-TZVP, CAM-B3LYP-D4/def2-TZVP, and ωB97X-D4/def2-TZVP levels of theory. Simulated absorption spectra were constructed by Gaussian broadening of the calculated vertical excitations (σ = 15 nm) and normalized to their maximum intensity. Blue sticks represent individual singlet excitations with heights proportional to their oscillator strengths.

Calculated transitions

Table S21. TD-DFT results for **[2a]**$^+$ at the TPSSh-D4/def2-TZVP level of theory. Reported values include vertical excitation energies, absorption wavelengths, oscillator strengths (f_{osc}), and the dominant one-electron orbital transitions with their corresponding weights. The lowest triplet state (T_1) is included for comparison.

State	Energy (eV)	λ (nm)	f_{osc}	Dominant orbital transition
S_1	2.300	549	0.0002507	HOMO → LUMO (99.76%)
S_2	2.404	516	0.0002062	HOMO –1 → LUMO (99.90%)
S_3	2.851	435	0.03045	HOMO –2 → LUMO (98.27%)
S_4	3.278	378	0.6541)	HOMO –3 → LUMO (89.51%)<br>HOMO –4 → LUMO (6.949%)
T_1	2.300	539	-	HOMO → LUMO (96.45%)

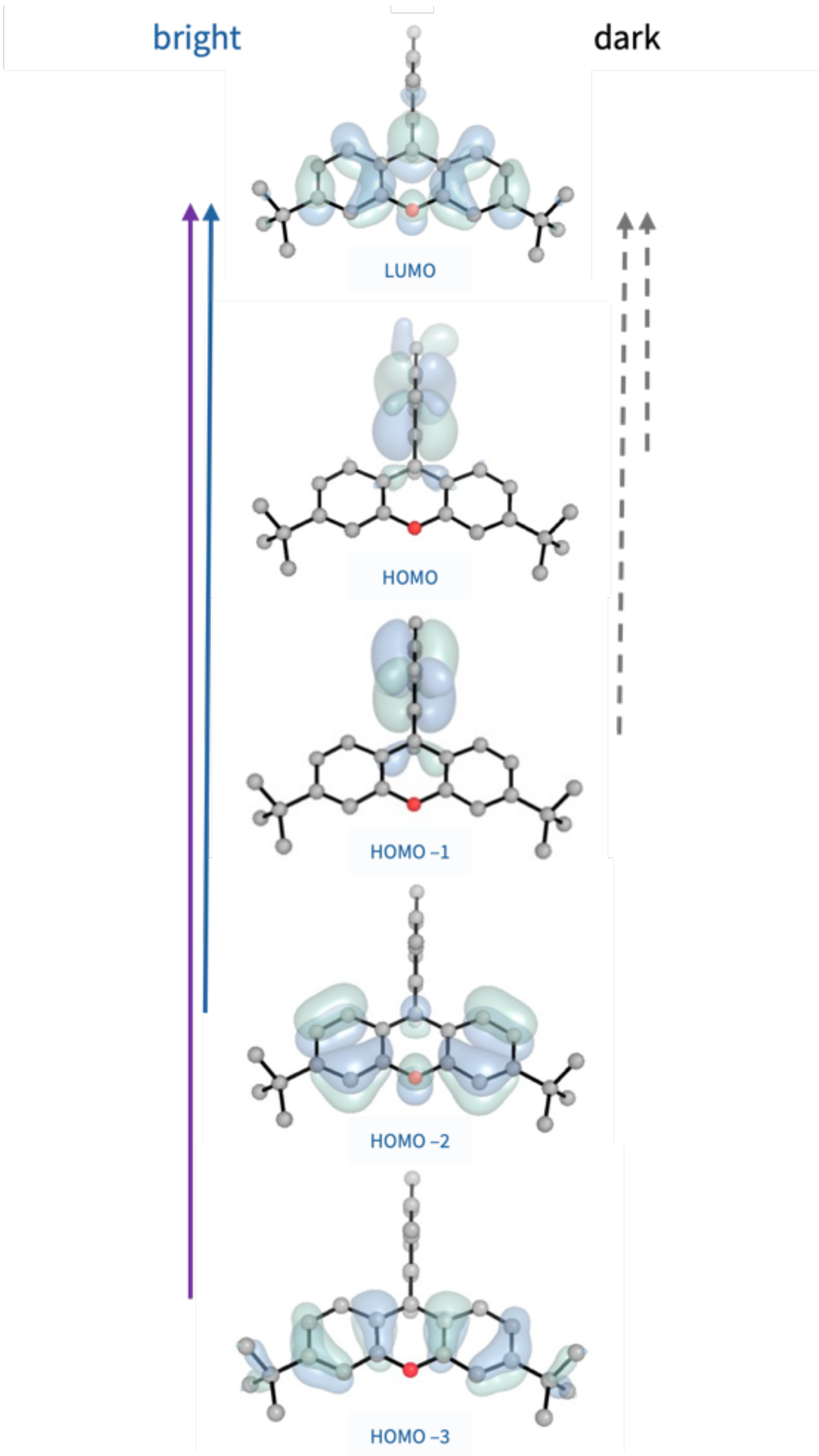

Figure S131. Frontier molecular orbitals of **[2a]⁺.** Solid arrows indicate bright transitions, whereas dashed arrows indicate dark transitions. Orbital isosurfaces are plotted at an isovalue of ±0.03 a.u.

394 nm transition has CT character

Thioxanthylium $[3a]^+$

Optimized structure

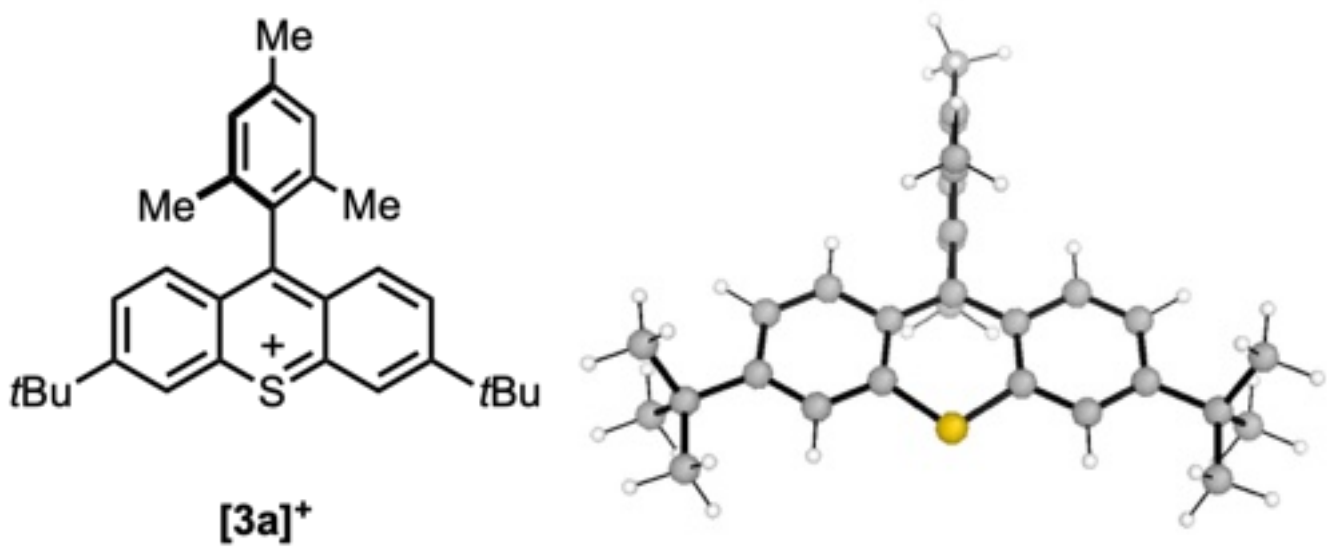

TD-DFT benchmarking

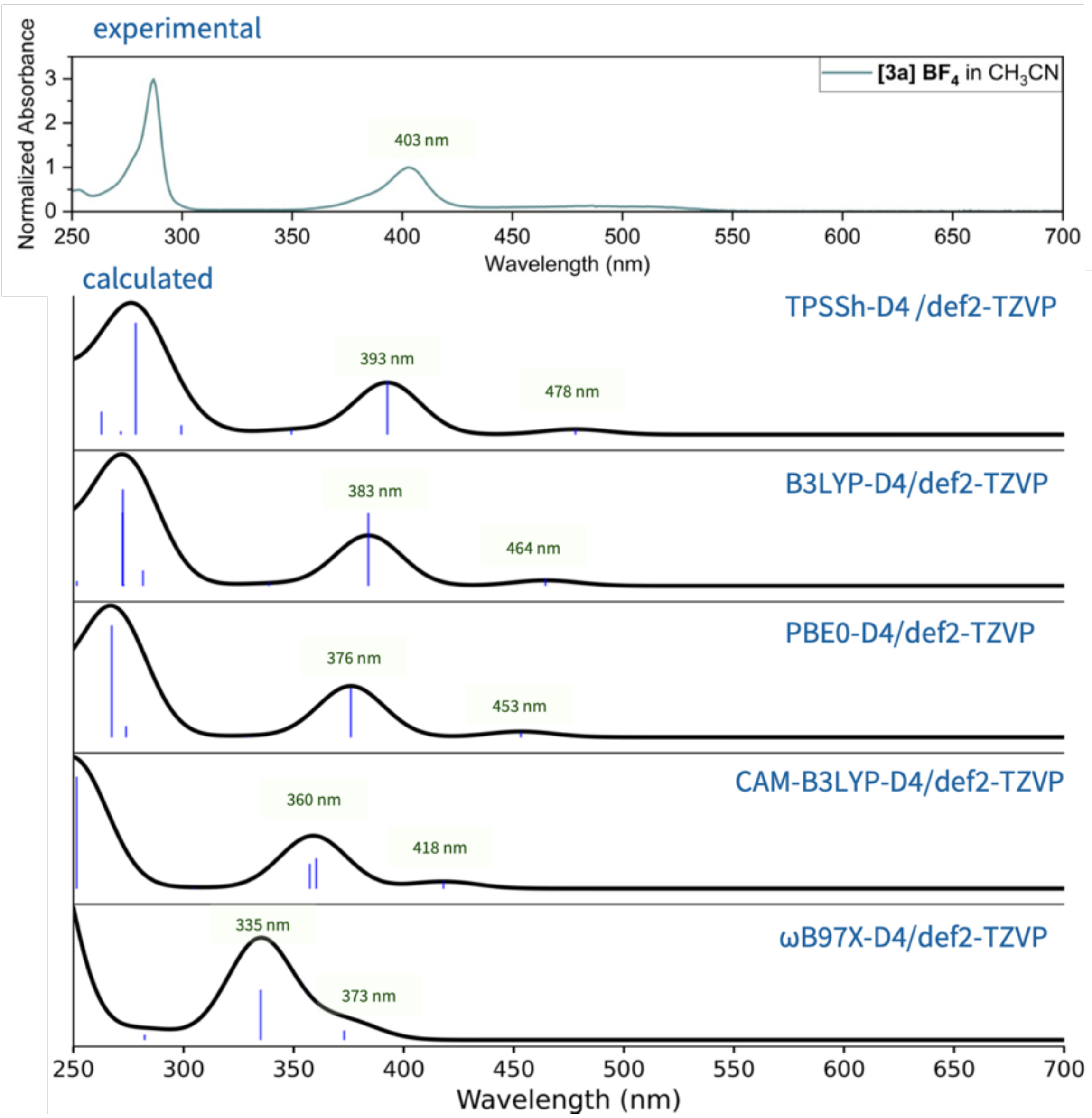

Figure S132. Benchmark of TD-DFT functionals for reproducing the experimental UV–Vis absorption spectrum of **[3a]**$^+$ in CH_3CN. Calculated spectra were obtained at the TPSSh-D4/def2-TZVP, B3LYP-D4/def2-TZVP, PBE0-D4/def2-TZVP, CAM-B3LYP-D4/def2-TZVP, and

ωB97X-D4/def2-TZVP levels of theory. Simulated absorption spectra were constructed by Gaussian broadening of the calculated vertical excitations (σ = 15 nm) and normalized to their maximum intensity. Blue sticks represent individual singlet excitations with heights proportional to their oscillator strengths.

Calculated transitions

Table S22. TD-DFT results for **[3a]⁺** at the TPSSh-D4/def2-TZVP level of theory. Reported values include vertical excitation energies, absorption wavelengths, oscillator strengths (f_{osc}), and the dominant one-electron orbital transitions with their corresponding weights. The lowest triplet state (T_1) is included for comparison.

State	Energy (eV)	λ (nm)	f_{osc}	Dominant orbital transition
S_1	2.201	563	0.00004426	HOMO → LUMO (99.78%)
S_2	2.325	533	0.0001208	HOMO –1 → LUMO (98.01%)
S_3	2.594	478	0.04894	HOMO –2 → LUMO (95.93%)
S_4	3.159	393	0.4798	HOMO –3 → LUMO (90.09%)
T_1	1.777	698	-	HOMO → LUMO (96.45%)

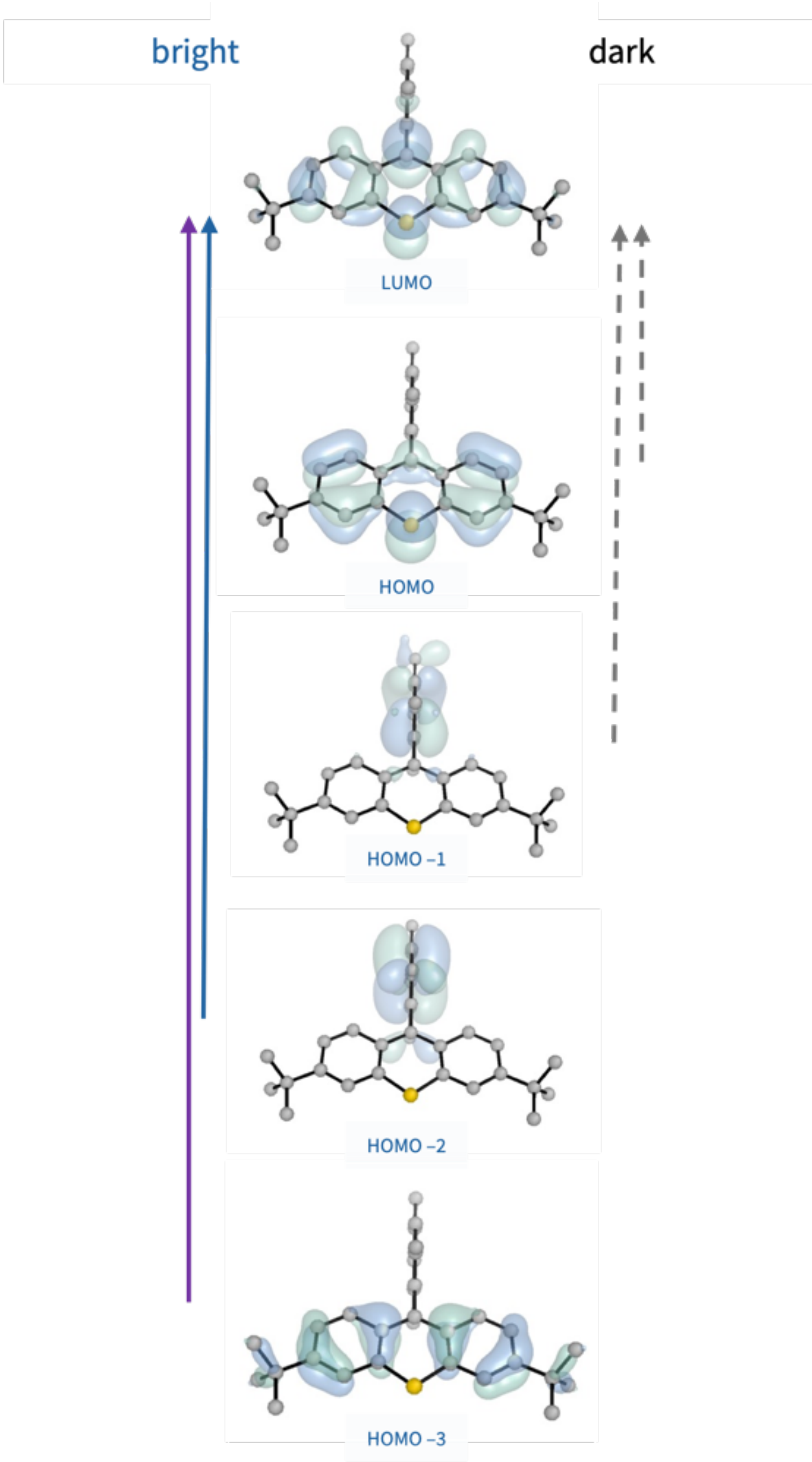

Figure S133. Frontier molecular orbitals of **[3a]$^+$.** Solid arrows indicate bright transitions, whereas dashed arrows indicate dark transitions. Orbital isosurfaces are plotted at an isovalue of ±0.03 a.u.

Selenoxanthylium [4a]$^+$

Optimized structure

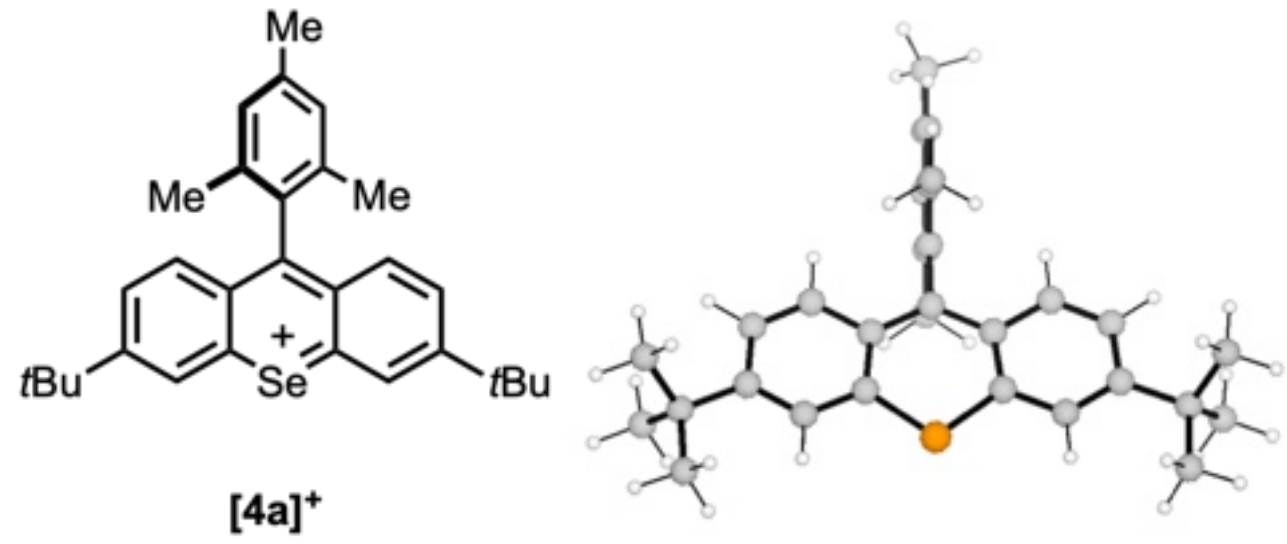

TD-DFT benchmarking

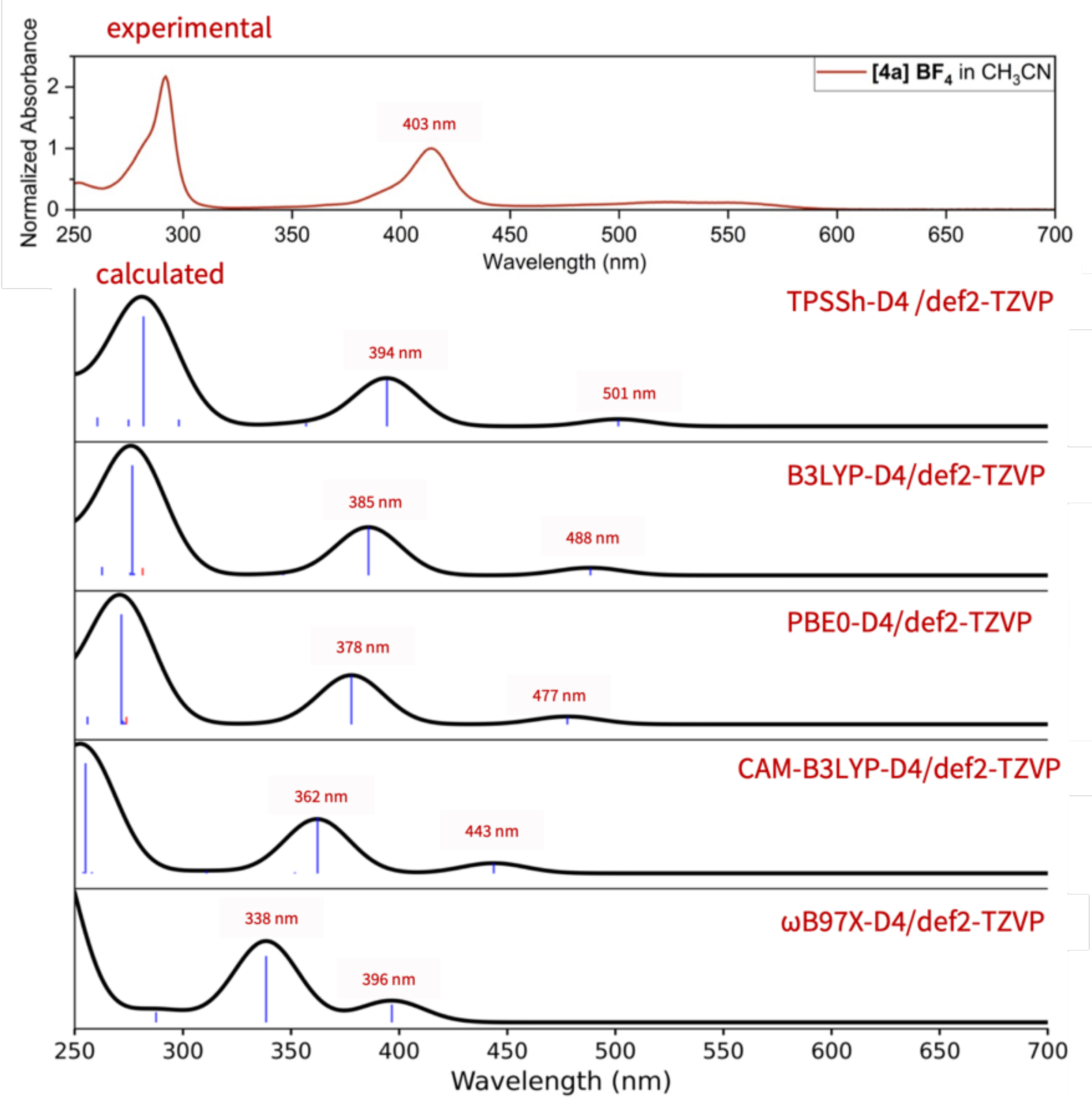

Figure S134. Benchmark of TD-DFT functionals for reproducing the experimental UV–Vis absorption spectrum of **[4a]$^+$** in CH_3CN. Calculated spectra were obtained at the TPSSh-

D4/def2-TZVP, B3LYP-D4/def2-TZVP, PBE0-D4/def2-TZVP, CAM-B3LYP-D4/def2-TZVP, and ωB97X-D4/def2-TZVP levels of theory. Simulated absorption spectra were constructed by Gaussian broadening of the calculated vertical excitations (σ = 15 nm) and normalized to their maximum intensity. Blue sticks represent individual singlet excitations with heights proportional to their oscillator strengths.

Calculated transitions

Table S23. TD-DFT results for **[4a]**$^+$ at the TPSSh-D4/def2-TZVP level of theory. Reported values include vertical excitation energies, absorption wavelengths, oscillator strengths (f_{osc}), and the dominant one-electron orbital transitions with their corresponding weights. The lowest triplet state (T_1) is included for comparison.

State	Energy (eV)	λ (nm)	f_{osc}	Dominant orbital transition
S_1	2.252	551	0.00005431	HOMO –1 → LUMO (99.77%)
S_2	2.396	517	0.0001478	HOMO –2 → LUMO (99.90%)
S_3	2.475	501	0.06944	HOMO → LUMO (97.76%)
S_4	3.144	394	0.4650	HOMO –3 → LUMO (89.52%)<br>HOMO –1 → LUMO+1 (5.474%)
T_1	1.594	778	-	HOMO → LUMO (97.32%)
$\Delta E_{ST(calc)}$	0.66			
$\Delta E_{ST(exp)}$	0.43			

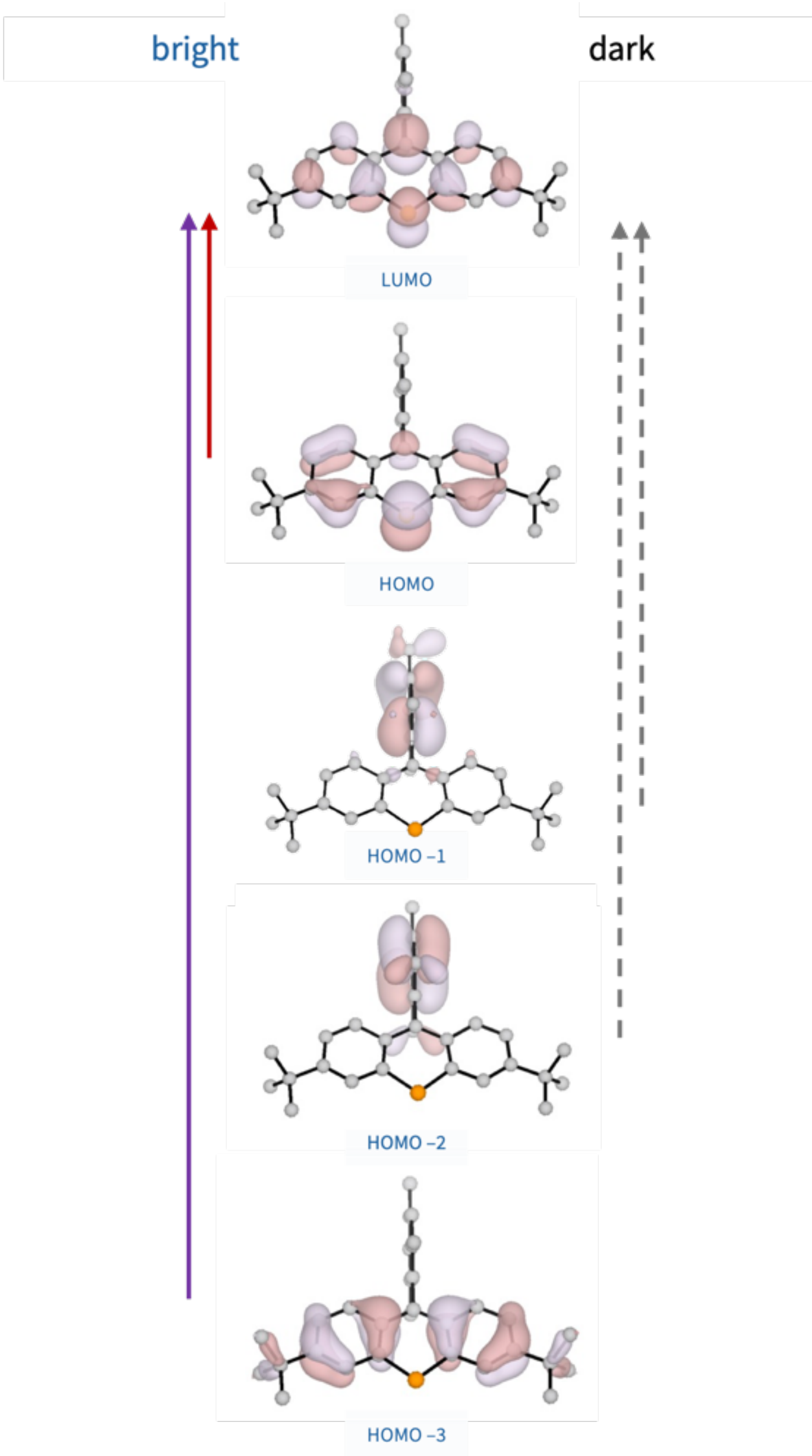

Figure S135. Frontier molecular orbitals of **[4a]⁺.** Solid arrows indicate bright transitions, whereas dashed arrows indicate dark transitions. Orbital isosurfaces are plotted at an isovalue of ±0.03 a.u.

Low-energy transition has LE character

SOC COMPARISON

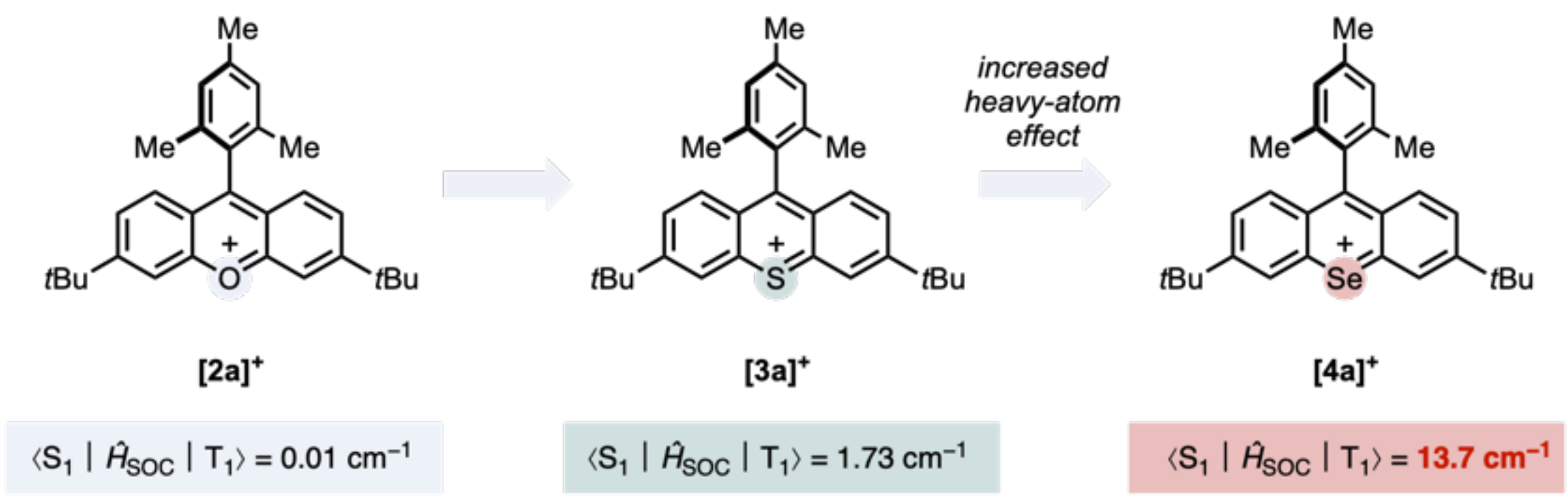

Figure S136. Spin–orbit coupling matrix elements across the chalcogenoxanthylium series.

Spin–orbit coupling matrix elements (SOCMEs) between singlet and triplet states were computed using the SOMF(1X) approach as implemented in ORCA.[10] SOC calculations were performed on TD-DFT states obtained at the TPSSh-D4/X2C-TZVPall level. Reported SOCME values correspond to $\langle S_n|\hat{H}_{SO}|T_m\rangle$ elements and are given in cm^{-1}.

Spin–orbit coupling matrix elements (SOCME) were computed using TDDFT and the state interaction approach as implemented in ORCA 6.0.1. SOC values $\langle S_n|\hat{H}_{SOC}|T_1\rangle$ were obtained using the mean-field spin–orbit Hamiltonian (DOSOC) and are reported as $|H_{SOC}| = \sqrt{H_x^2 + H_y^2 + H_z^2}$ *in* cm^{-1}.

Table S24. Calculated SOCME magnitudes for **[2-4a]+**

	T	S	Z (cm^{-1})	X (cm^{-1})	Y (cm^{-1})	|HSO| (cm^{-1})	$\langle S_n\|\hat{H}_{SOC}\|T_1\rangle$
**[2a]+**	1	1	-0.01	0.01	0.00	**0.00**	**$\langle S_1\|\hat{H}_{SOC}\|T_1\rangle$**
**[3a]+**	1	1	-1.17	1.27	-0.12	**1.73**	**$\langle S_1\|\hat{H}_{SOC}\|T_1\rangle$**
**[4a]+**	1	1	-9.14	10.11	-0.98	**13.7**	**$\langle S_1\|\hat{H}_{SOC}\|T_1\rangle$**

COORDINATES FOR XANTHYLIUM [2A]+

O	11.51325990868896	6.05883524230786	16.69001523405624
C	8.35838907495878	5.78283661261315	19.56456296009021
C	8.50797823860081	6.41559225555187	20.81124722262946
C	7.46437045243846	6.31280163838673	21.73504494666730
H	7.57442943218009	6.80141577227328	22.70907935648320
C	6.29322811814419	5.60483802620597	21.44957549558521

C	6.17954862229371	4.98400950973769	20.20158970354949
H	5.27207566221857	4.41954316599829	19.96169954237288
C	7.19477843350520	5.06009015887186	19.24479493840947
C	9.45737649800734	5.87755438063380	18.56261717467156
C	9.47619922262882	6.91878106151991	17.60790760160224
C	10.53633208956553	6.97360415217016	16.67436697652365
C	11.52977954042661	5.05893328075844	17.57948160443729
C	10.50567925625236	4.93102373274204	18.54567853976175
C	9.76012200977237	7.18219037266100	21.14771374588889
H	10.64911784208282	6.53149858639647	21.11241602941936
H	9.69606181437448	7.61559242334418	22.15554940970726
H	9.93152743219732	8.00389545035805	20.43347878747880
C	5.17017036846866	5.53454708287430	22.44937737224519
H	4.44162882239718	6.34333038567182	22.27254387306200
H	5.54172006812082	5.64541031022885	23.47881292952007
H	4.62547430402301	4.58155834750324	22.37443984288848
C	7.04627471092386	4.38211589364073	17.90803711307295
H	7.10775214233590	5.10900456186687	17.08184435373343
H	6.07914413205082	3.86534253959073	17.83495722496337
H	7.84366482205319	3.63871479441318	17.74547105423076
C	8.47763496965689	7.93382993063823	17.51937044692376
H	7.64595326019486	7.91910599869957	18.22796369871313
C	8.56038586498558	8.90675939109333	16.56228431062486
H	7.78057128071122	9.67049886611716	16.51737340007545
C	9.63829468134891	8.94717712050013	15.62366341800221
C	10.61884944183040	7.97270236890718	15.69622977832303
H	11.46823272236185	7.94523032920307	15.01299176034379
C	12.59985456325506	4.15853927204716	17.50202341750285

H	13.34900360302780	4.32636351298057	16.72753922767479
C	12.67506265014158	3.10123124444249	18.39234598091429
C	11.64674639544822	2.95788401186227	19.37559410145486
H	11.69349151349627	2.12760339111642	20.08400015026377
C	10.60075966430808	3.83482591387905	19.45381736386621
H	9.82381744597651	3.70844406279866	20.21167789736253
C	9.66886906882837	10.05852279433286	14.57523677383367
C	9.70268692659259	11.41739972018353	15.29500423456618
H	10.59960163612605	11.50901578242554	15.92872185038576
H	8.81638245749015	11.57229766881786	15.92953471407284
H	9.72715711906118	12.22983401511576	14.55117510605478
C	13.81745674928924	2.08682255080749	18.35862018316401
C	14.82207093479794	2.38904840138567	17.24427126310637
H	15.62235521155759	1.63298167592575	17.25898567731289
H	15.29421475552963	3.37594540207162	17.37309190819872
H	14.35258995414281	2.35783927783567	16.24824724687117
C	8.39718611736255	9.95894021325309	13.71558750133458
H	8.40413618234431	10.75210254336267	12.95082813241294
H	7.48162406177149	10.08059116830261	14.31492268388969
H	8.34238010513396	8.98750985247271	13.19807643861734
C	10.89184225964707	9.95394359712381	13.66102418803173
H	10.90638934498148	9.00594336668259	13.10018372952785
H	11.83458981186055	10.03673866886158	14.22475581482207
H	10.86968852498272	10.77421546574553	12.92691702373460
C	13.22749258535123	0.68526372238896	18.12728854527185
H	12.53461753043266	0.38958864426896	18.93022116757958
H	14.04018775067757	-0.05808505607974	18.09685896621585
H	12.68471332653015	0.63567062492105	17.16947116795020

C	14.55039233761687	2.12432887019005	19.71039051664615
H	14.97300535769708	3.12323399045075	19.90536937656039
H	15.38028037632178	1.39955821857576	19.70193077728326
H	13.88702000589443	1.86259353602134	20.54918498184135

Coordinates for thioxanthylium [3a]+

S	11.95438436970590	6.08993359088778	16.26866365337062
C	8.54374594322165	5.79810648210327	19.39086255823637
C	8.68935165822538	6.44426393104038	20.63096140595460
C	7.64917862842163	6.34203936090749	21.55894412925137
H	7.75799498157546	6.84129020091171	22.52776959709562
C	6.48321206413327	5.62151094301857	21.28445875571701
C	6.37250573678716	4.98734233655247	20.04305092397539
H	5.46948675657871	4.41250386774101	19.81087685548890
C	7.38527069142434	5.06223978621042	19.08334679921643
C	9.64413700869119	5.89111037003409	18.38172043362977
C	9.61085514576553	6.95667707284554	17.44481466761540
C	10.60677386097255	7.13450046181691	16.44680676302199
C	11.76232676500719	4.91378318209803	17.50144712878232
C	10.67099433527981	4.91208541471202	18.41171486774837
C	9.93543877734848	7.22428264159879	20.95900370703153
H	10.82991453217383	6.58069651921351	20.92927964230016
H	9.86866393755787	7.66671197466871	21.96277676171898
H	10.10000725358156	8.04067307322351	20.23699101706766
C	5.36335537910077	5.55153176943292	22.28797943467859
H	4.62720467870517	6.35216150622359	22.10557884061317
H	5.73666530348588	5.67547933197420	23.31530719508467
H	4.82668297684414	4.59317672245223	22.22398650841201
C	7.23810908207243	4.36724537018533	17.75519306104376
H	7.29433431422727	5.08391683937143	16.91968632691416
H	6.27370271870118	3.84424288435723	17.69012023490034
H	8.03916594611363	3.62604502675755	17.60019984309329
C	8.54850731966158	7.91455553907696	17.46847677378034

H	7.76568393132775	7.81086757754474	18.22208223331683
C	8.49801938187983	8.94653980917441	16.57397540434012
H	7.66715370637126	9.65378429738878	16.63201844582046
C	9.50090964372556	9.11677597848255	15.57509123336762
C	10.53897356252655	8.20361565055622	15.53093091328480
H	11.32863563159147	8.29576513812356	14.78224424555959
C	12.76606077516587	3.92624810372588	17.56475129763258
H	13.58480859013776	3.97243141269275	16.84366397029479
C	12.72046341243462	2.92104812772848	18.51388535077371
C	11.62722585153191	2.91014226350133	19.42885777027830
H	11.56443159893177	2.12653802556422	20.18785680250638
C	10.64691978195160	3.86120100332004	19.38212509360805
H	9.82262243624954	3.82654118539322	20.09689280549707
C	9.39563360843321	10.28892001031125	14.60062204737735
C	9.40234065821595	11.59971966001534	15.40539590705862
H	10.33508610969438	11.70677210509897	15.98244158436532
H	8.55569463734322	11.65878166907059	16.10679335918205
H	9.32702511729168	12.45739571092225	14.71783002103609
C	13.78977369380745	1.83390759326725	18.61110604756405
C	14.88708548355041	2.00699024912563	17.55817404646202
H	15.62935634621378	1.20144748904778	17.66921181167705
H	15.41757358867231	2.96594378006185	17.67069515044762
H	14.48656506207807	1.95107993053379	16.53346393718605
C	8.07436710121375	10.16586412976974	13.82281875793605
H	7.98185954931577	11.00410991390644	13.11368096975684
H	7.19813521885747	10.19368336517277	14.48888448646565
H	8.03822211213515	9.22702572452438	13.24680802004456
C	10.55568015645178	10.31611150653513	13.60264906724484

H	10.58461242148311	9.40722145577461	12.98080727740477
H	11.52940583243712	10.42223727118068	14.10674761920188
H	10.43572767001048	11.17707733574289	12.92689616843563
C	13.11883632049809	0.46481142078652	18.40748079420055
H	12.35166448394586	0.26205355324113	19.17085752188763
H	13.87600511175249	-0.33284056053916	18.47436492225455
H	12.64207545548555	0.39955886129393	17.41605528706764
C	14.42991595486427	1.89489085614029	20.00836337542447
H	14.90799367829883	2.87219371819481	20.18375002267654
H	15.20493228615566	1.11652866996991	20.09530082466425
H	13.69422544007980	1.72400967628867	20.80948547533696

Coordinates for selenoxanthylium [4a]$^{+}$

Se	12.11039362269843	6.11803848486706	16.15627040524902
C	8.60492470019331	5.80656407314571	19.34275372267718
C	8.74404940531822	6.45486967404328	20.58239995746792
C	7.70177520276331	6.35227175278304	21.50827244156665
H	7.80694038885040	6.85370289029363	22.47644796185378
C	6.53802186668151	5.62874170240417	21.23250658752687
C	6.43274795166120	4.99203215954123	19.99198114086289
H	5.53173377143623	4.41454647364378	19.75828235137514
C	7.44844968314051	5.06765163358545	19.03521550736185
C	9.70938853140721	5.90137542179932	18.33403993099162
C	9.65758109341689	6.97402688165624	17.40197495939439
C	10.63193524544218	7.19244921139312	16.38925508909056
C	11.84351778656544	4.87970521019179	17.49476719618653
C	10.73161700222772	4.91384292158538	18.38111023710255
C	9.98704688745142	7.23879602682356	20.91334565510935
H	10.88349633264809	6.59782118941770	20.88708413750905
H	9.91620295954763	7.68246560279566	21.91637514211115
H	10.15172752698358	8.05464828029332	20.19075105552767
C	5.41533468727500	5.55822262844089	22.23302173465023
H	4.67771733286823	6.35682336345739	22.04754413176196
H	5.78538398377790	5.68453713830749	23.26127473247676
H	4.88087544127416	4.59856860005650	22.16933780296669
C	7.30550756832356	4.36862920853592	17.70859171342084
H	7.36321180204776	5.08277725127443	16.87101999222223
H	6.34188282075417	3.84418604600412	17.64230399842544
H	8.10784986580837	3.62794874936561	17.55771400298182
C	8.57304741298311	7.91063456093034	17.45327034741579

H	7.80340203835594	7.78132464947429	18.21594910164642
C	8.48134411790671	8.95436387795350	16.57660941138624
H	7.63454794711102	9.63996685079616	16.66074306902938
C	9.46188376612113	9.16370308081770	15.56289643915361
C	10.51799335544296	8.27493138298937	15.49096008127812
H	11.29406380972032	8.39614100117447	14.73190040907962
C	12.82216706130062	3.86741485882320	17.59566057671753
H	13.65480694593231	3.88828569926535	16.88865981811382
C	12.73723862097614	2.87323796051228	18.55205309156745
C	11.62595998323886	2.89981807781150	19.44484578819827
H	11.52855874127429	2.12795873795172	20.21237041441237
C	10.66992810154385	3.87250149799875	19.36483368701068
H	9.83284921044719	3.86050994412342	20.06478379198736
C	9.31177405226139	10.34809584215006	14.60893975233339
C	9.30860963070333	11.64678406678288	15.43305582713170
H	10.25103981708801	11.76476461000382	15.99204866450779
H	8.47643612455301	11.67692987499626	16.15342906643115
H	9.20054292807069	12.51368505038427	14.76144345192480
C	13.77656675997735	1.76025509488182	18.68120590027219
C	14.89775844170432	1.89442565331832	17.64785570148472
H	15.61786382683747	1.07210880462572	17.78103847238802
H	15.44958270329629	2.84126358973396	17.76018240910861
H	14.51538112261594	1.83749862428460	16.61631833475671
C	7.97757116423413	10.21066613051632	13.85630856116656
H	7.85231119728920	11.05787047729571	13.16286190771826
H	7.11508166077568	10.20941997287678	14.54070633639109
H	7.94830460114343	9.28052503280095	13.26589383274772
C	10.44969502144291	10.41465985483664	13.58753688101165

H	10.48562601440196	9.51476048293640	12.95311390824158
H	11.43125935237990	10.53506529826865	14.07284469039948
H	10.29739354165964	11.28179972781279	12.92616420349693
C	13.07542717546060	0.40633044431437	18.47814307491090
H	12.28829213216389	0.23155225678347	19.22807394317654
H	13.80978787048858	-0.41032781667758	18.56779512978844
H	12.61619550733058	0.34279087406186	17.47830707436014
C	14.39170226091452	1.81810351547509	20.08967153897761
H	14.89237249556289	2.78410503784587	20.26483909240174
H	15.14376257371213	1.02032367363594	20.20020169452991
H	13.63622901849002	1.67560893777651	20.87792088985784

Spectroscopic Data

^{1}H NMR of [2a] BF_4

CDCl3, 23 °C

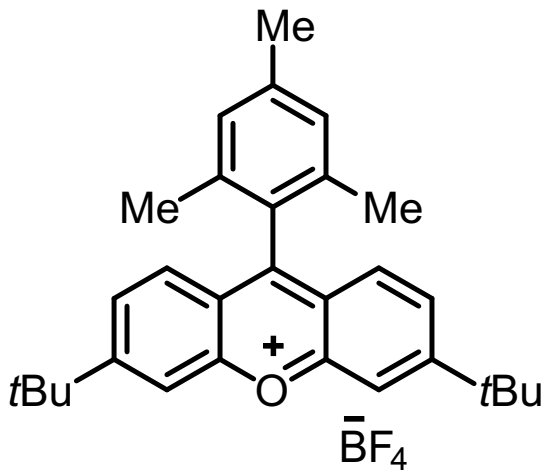

[2a] BF_4

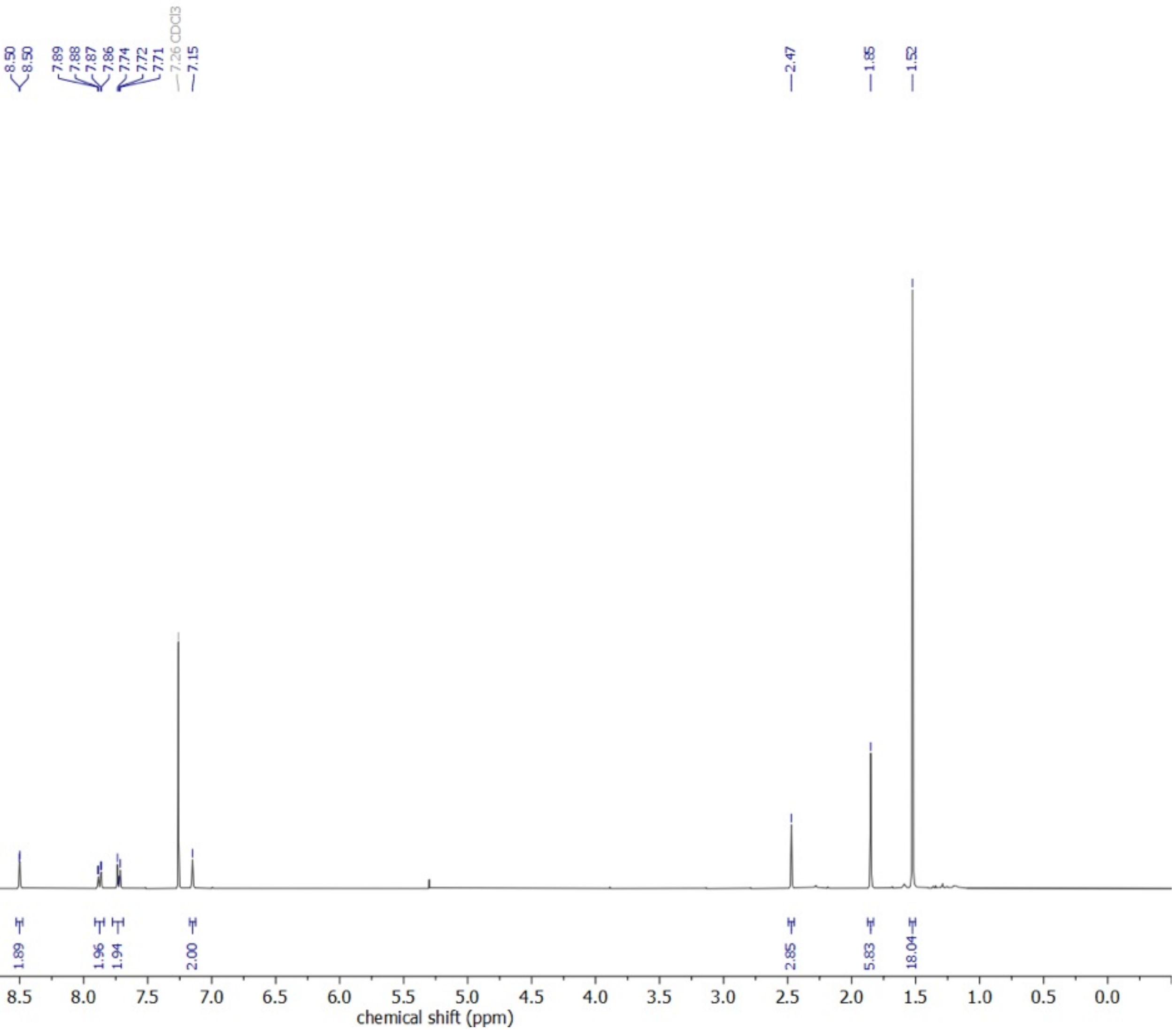

^{13}C NMR OF [2A] BF$_4$

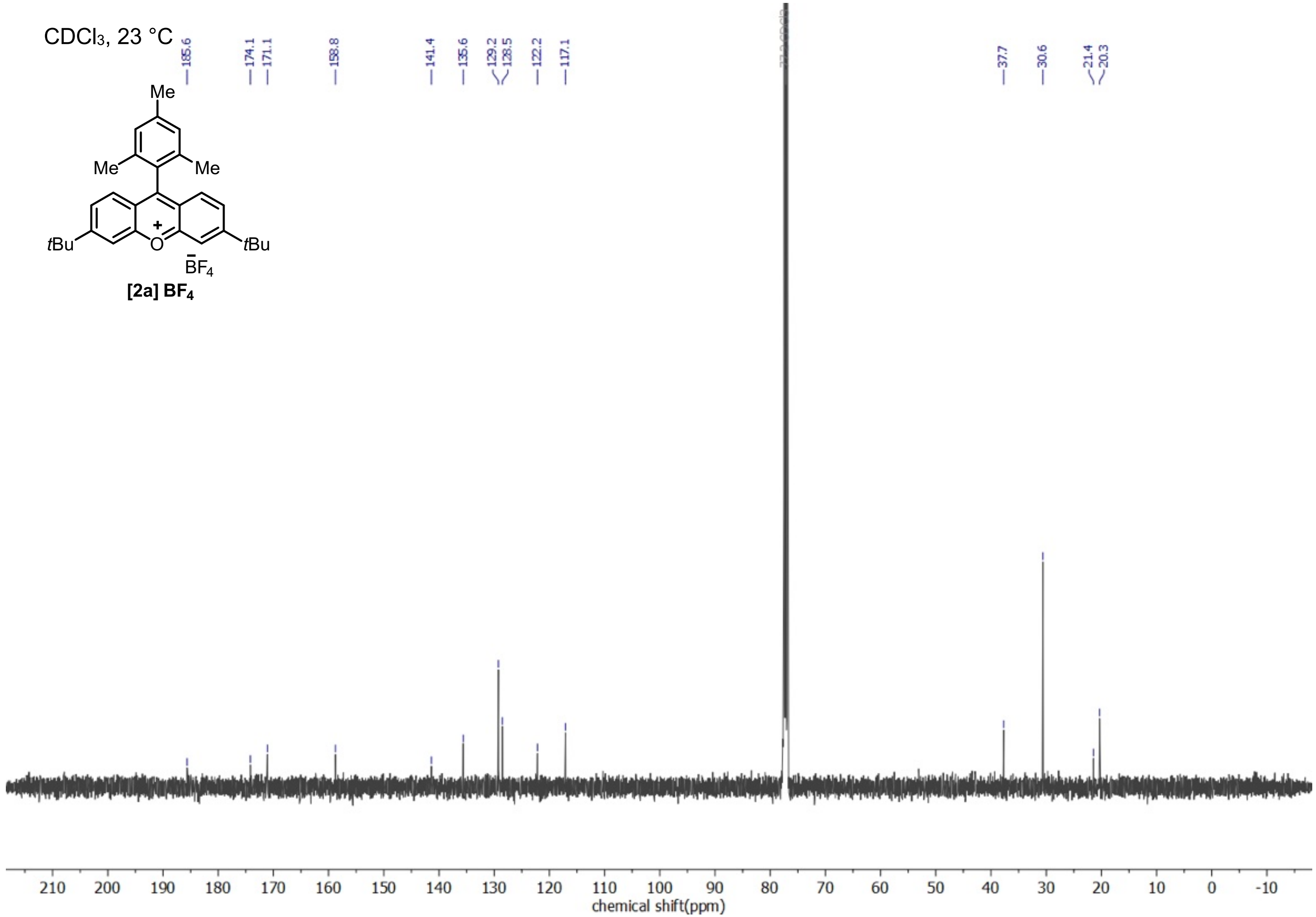

^{19}F NMR OF [2A] BF$_4$

CDCl$_3$, 23 °C

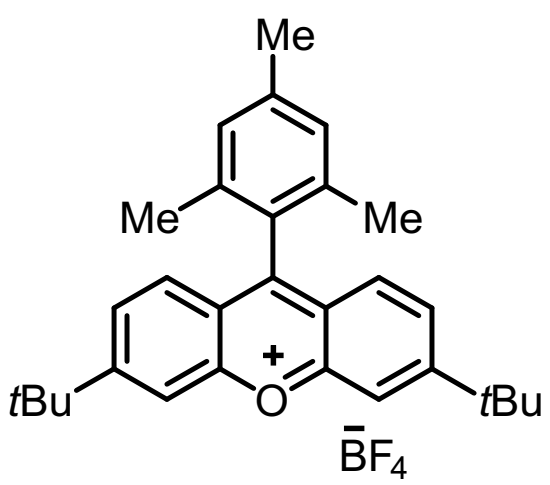

[2a] BF$_4$

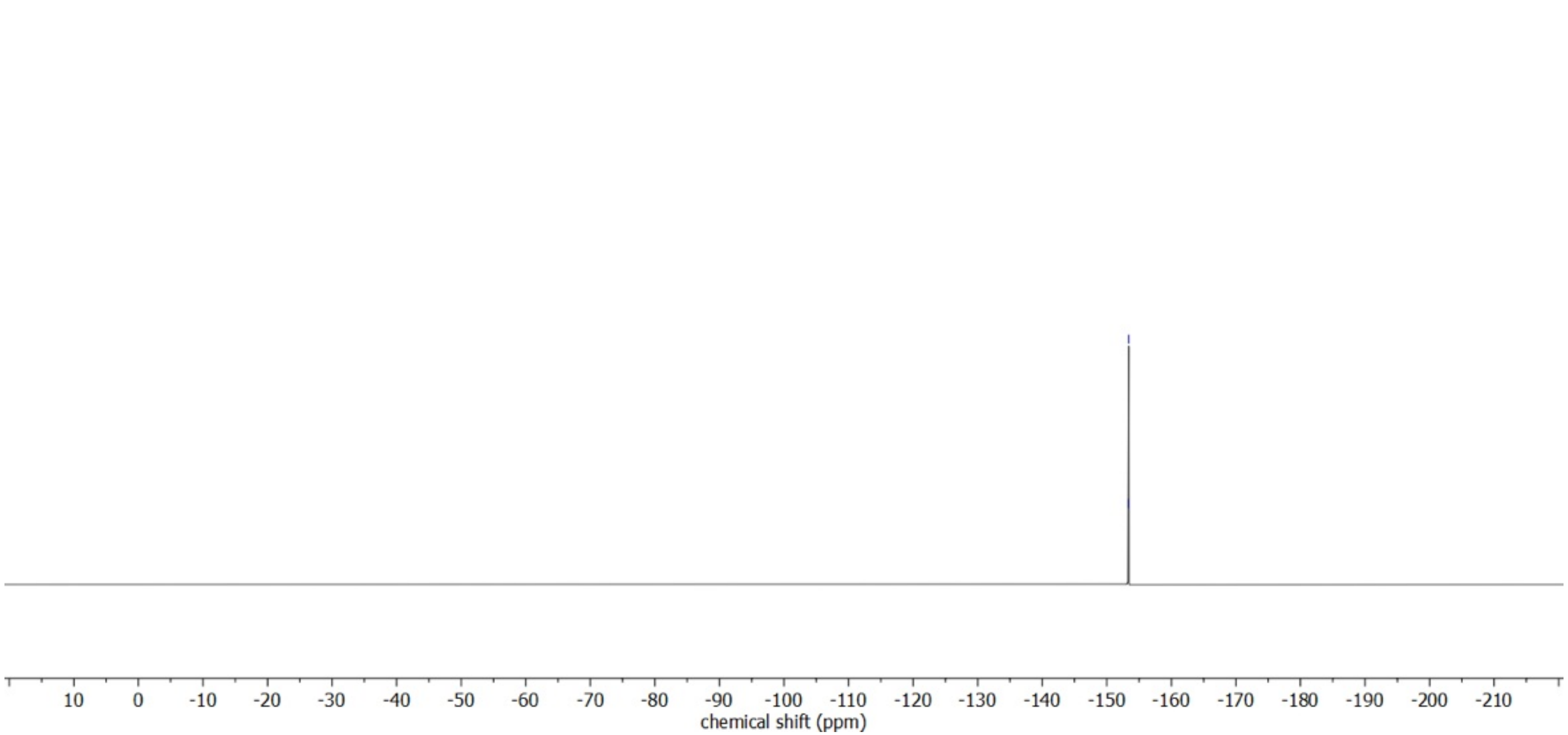

^{1}H NMR of Os(terpy)$_2$ (PF_6)$_2$

DMSO-d6, 23 °C

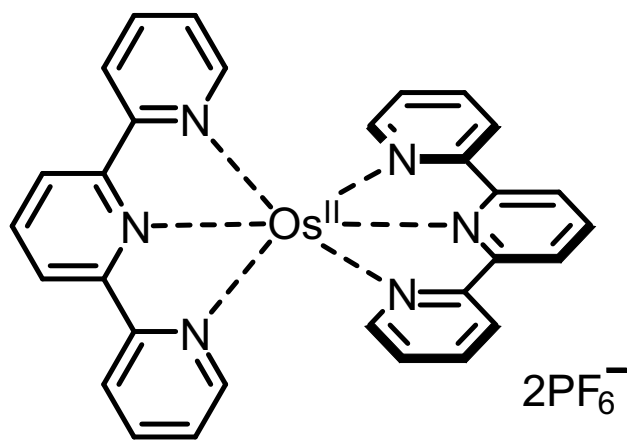

Os(terpy)$_2$

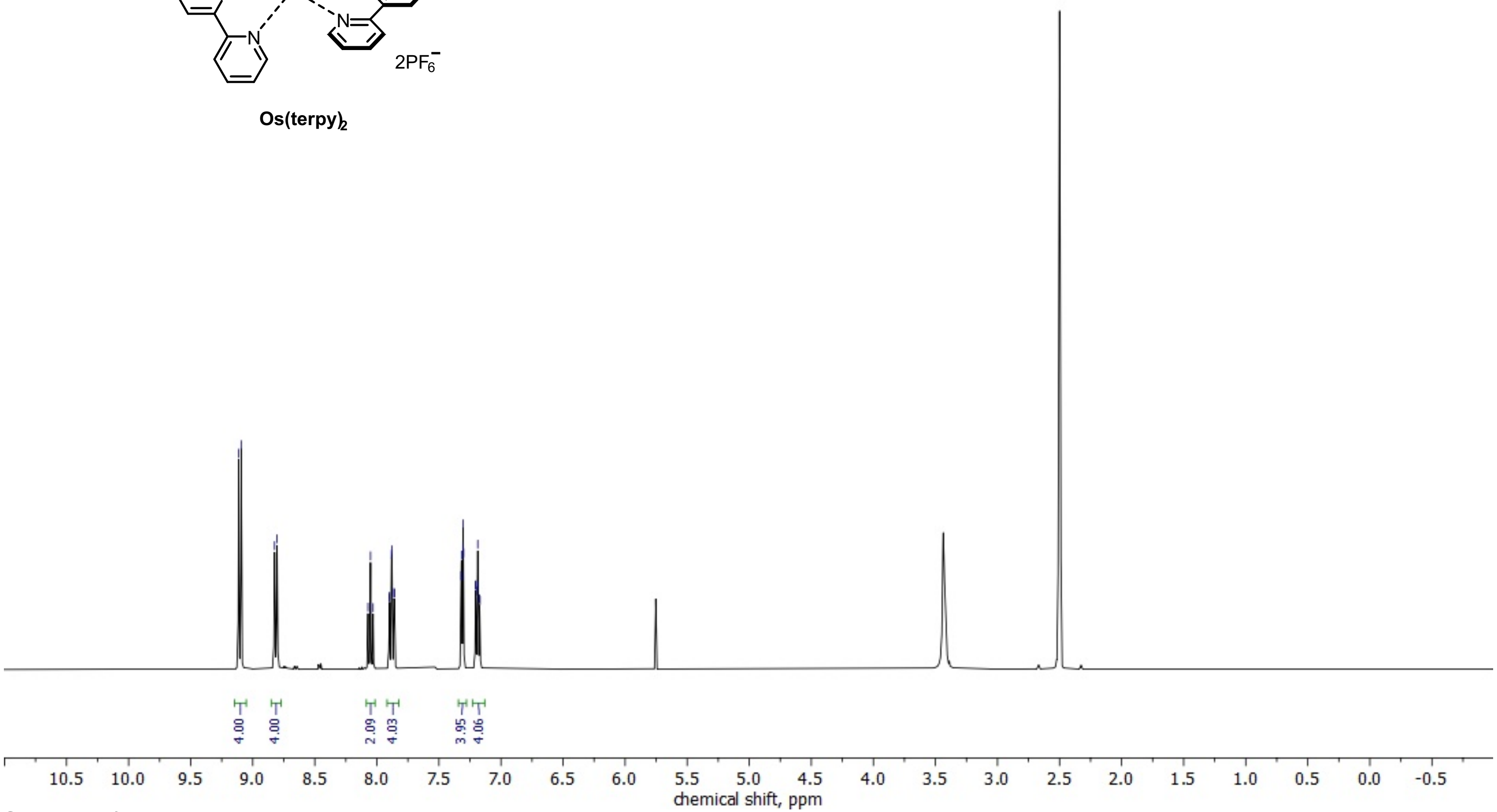

^{13}C NMR of Os(terpy)$_2$ (PF$_6$)$_2$

DMSO-d6, 23 °C

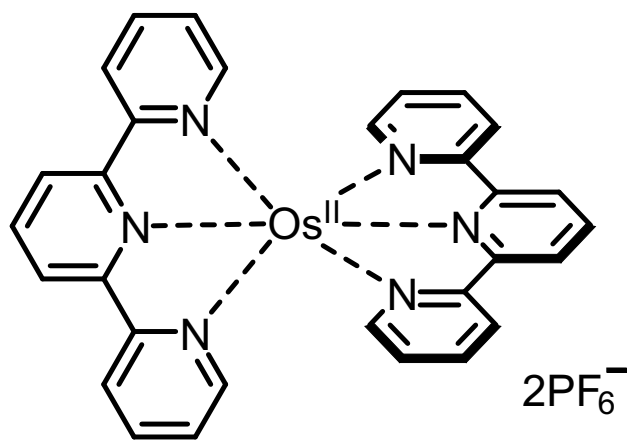

Os(terpy)$_2$

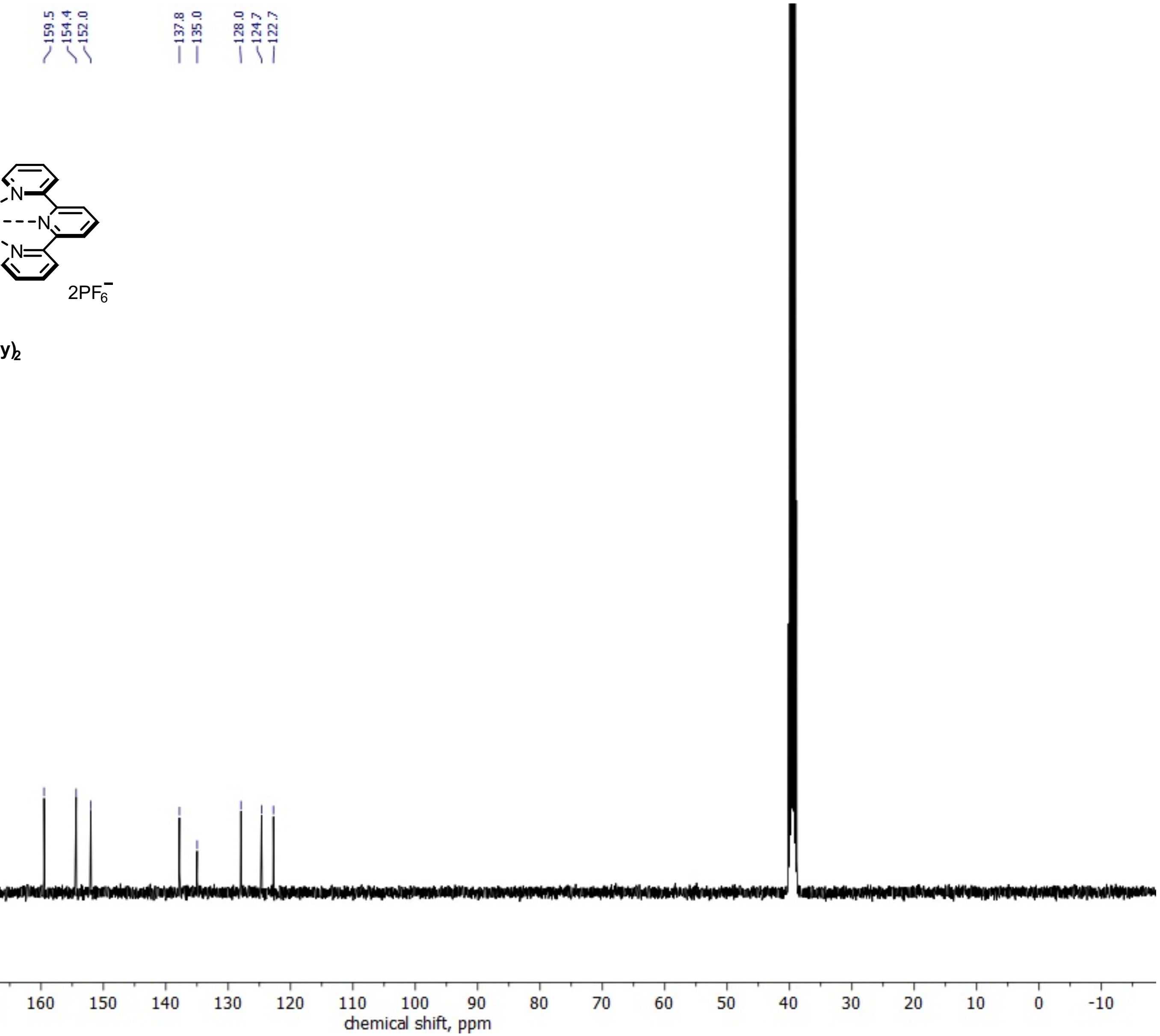

^{19}F NMR of Os(terpy)$_2$ (PF$_6$)$_2$

DMSO-d6, 23 °C

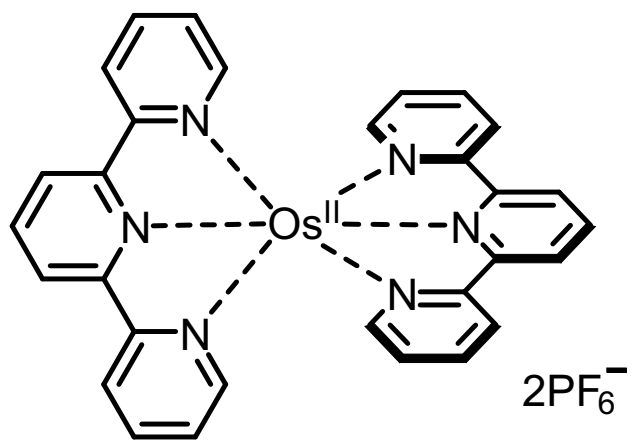

Os(terpy)$_2$

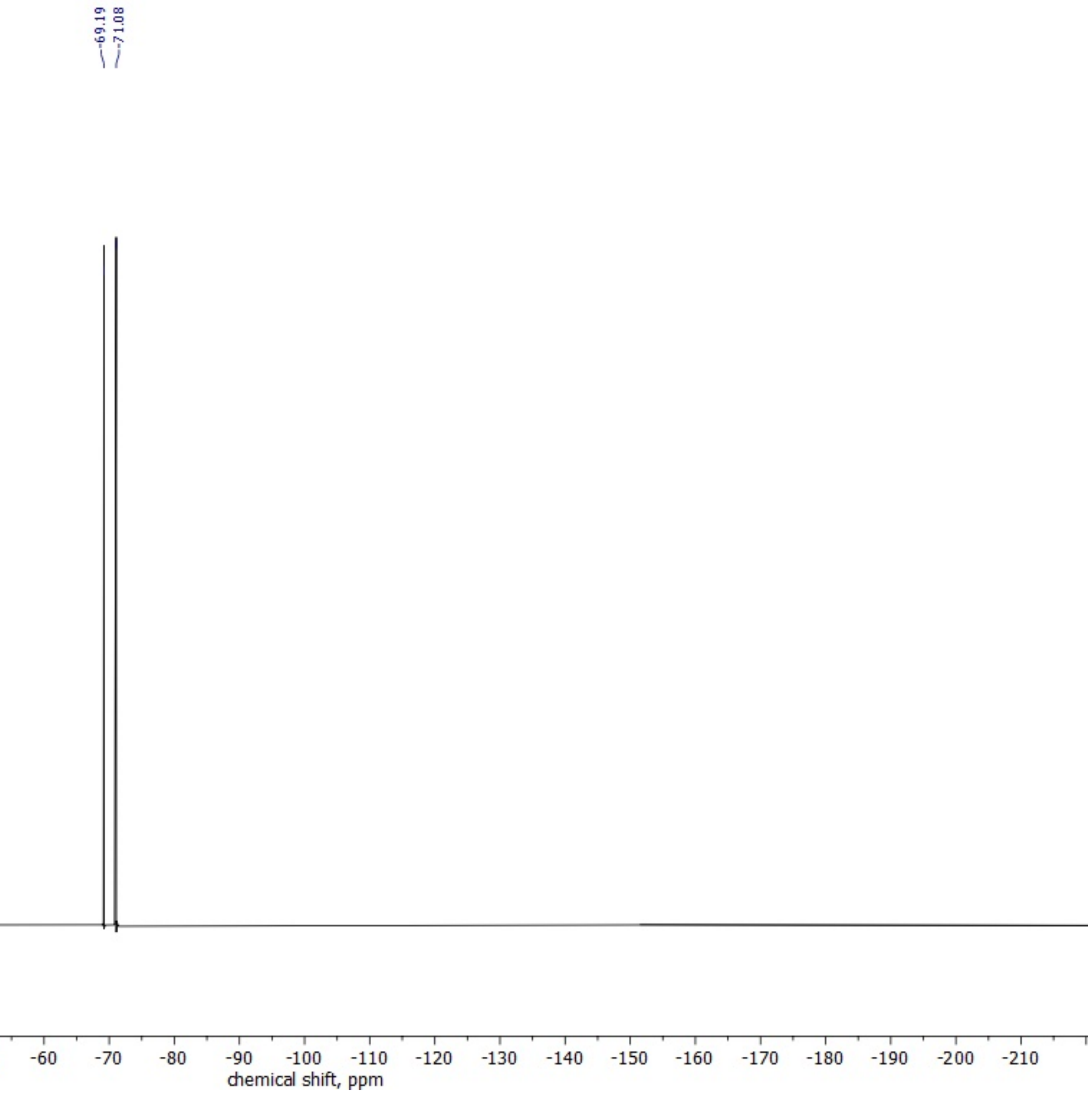

^{1}H NMR OF [3A] BF$_4$

CDCl$_3$, 23 °C

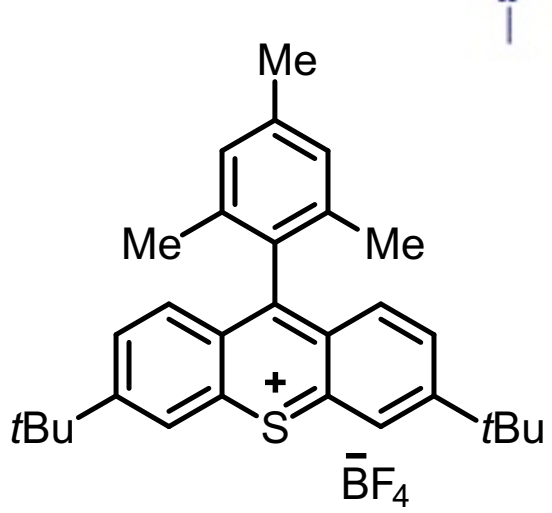

[3a] BF$_4$

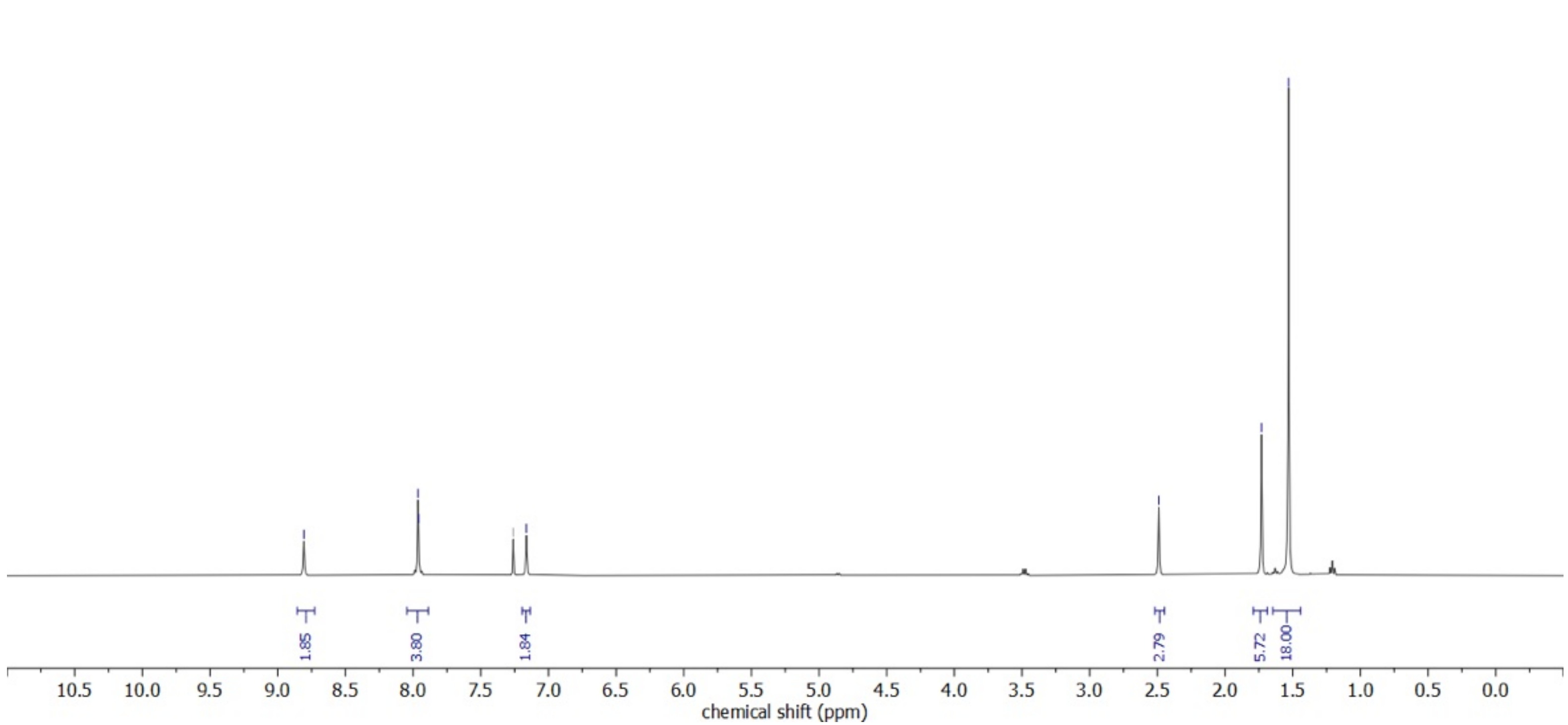

^{13}C NMR OF [3A] BF$_4$

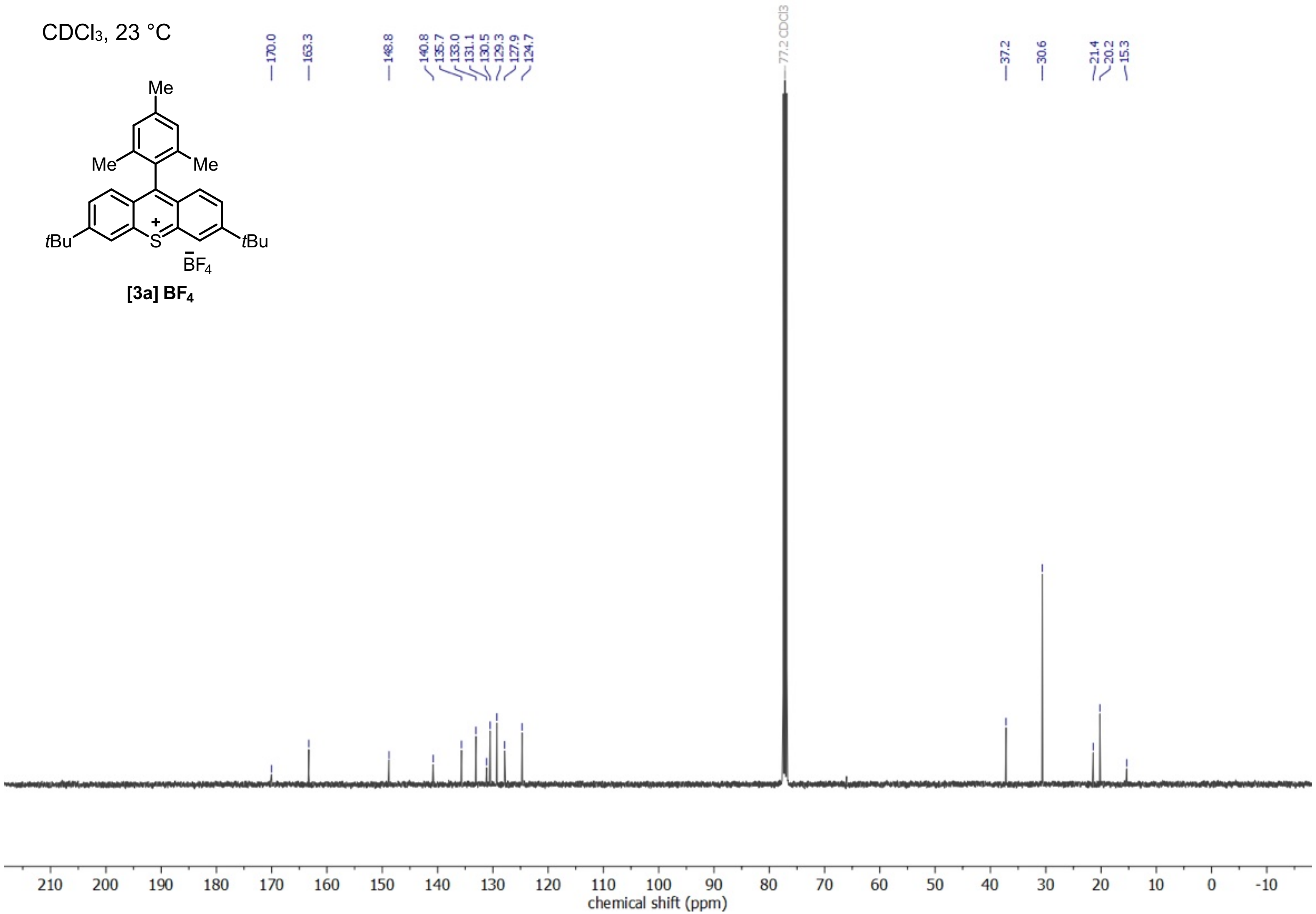

^{19}F NMR OF [3A] BF$_4$

CDCl$_3$, 23 °C

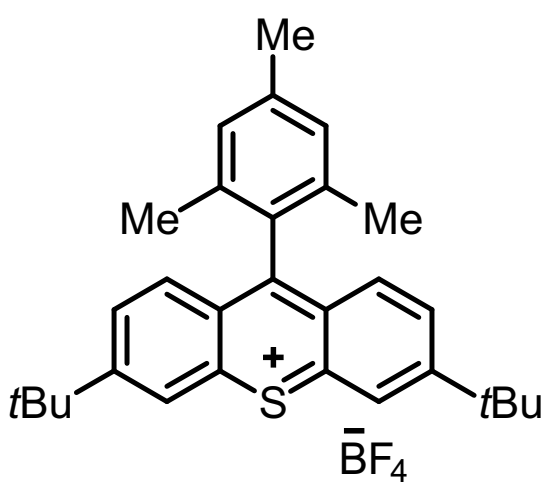

[3a] BF$_4$

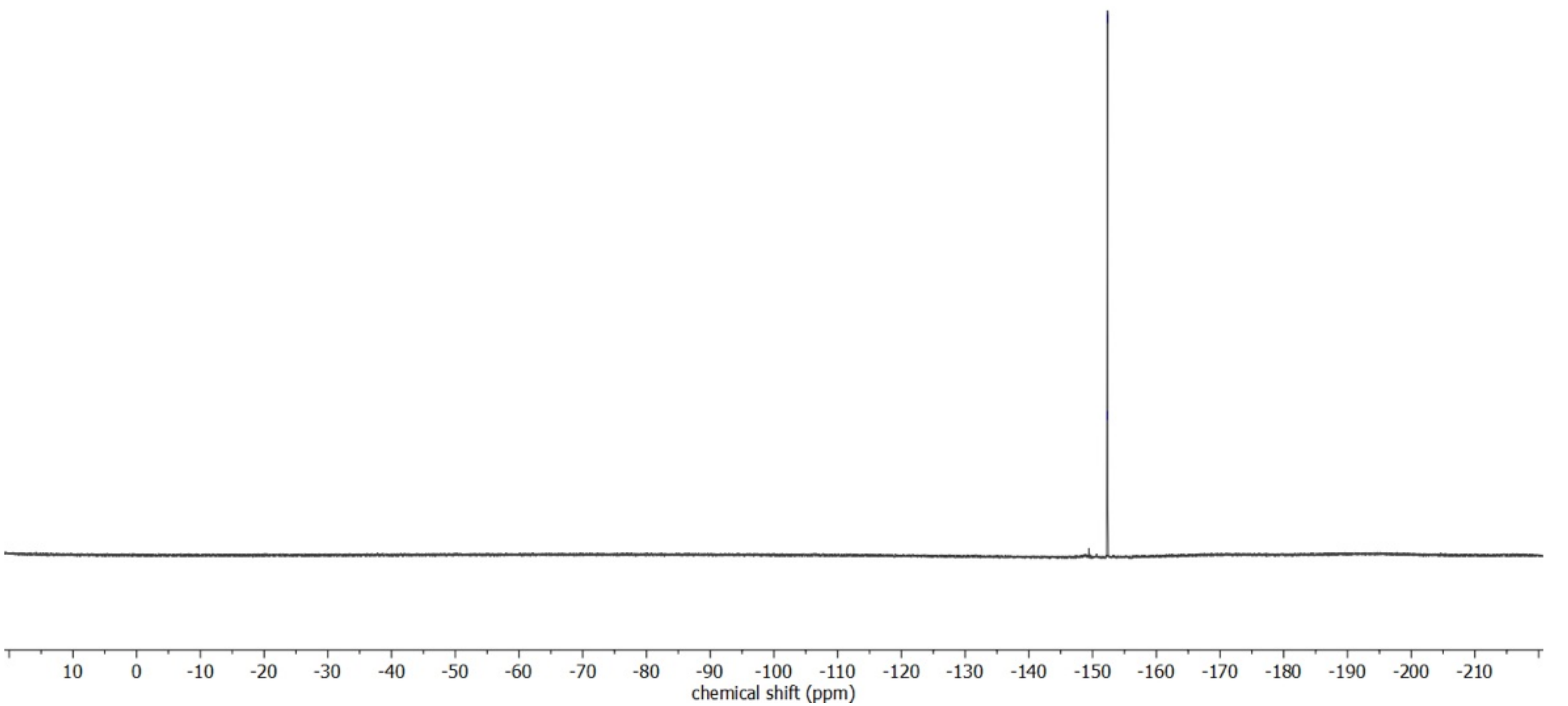

^{1}H NMR OF 3,6-DI-*TERT*-BUTYL-SELENOXANTHEN-9-ONE (5)

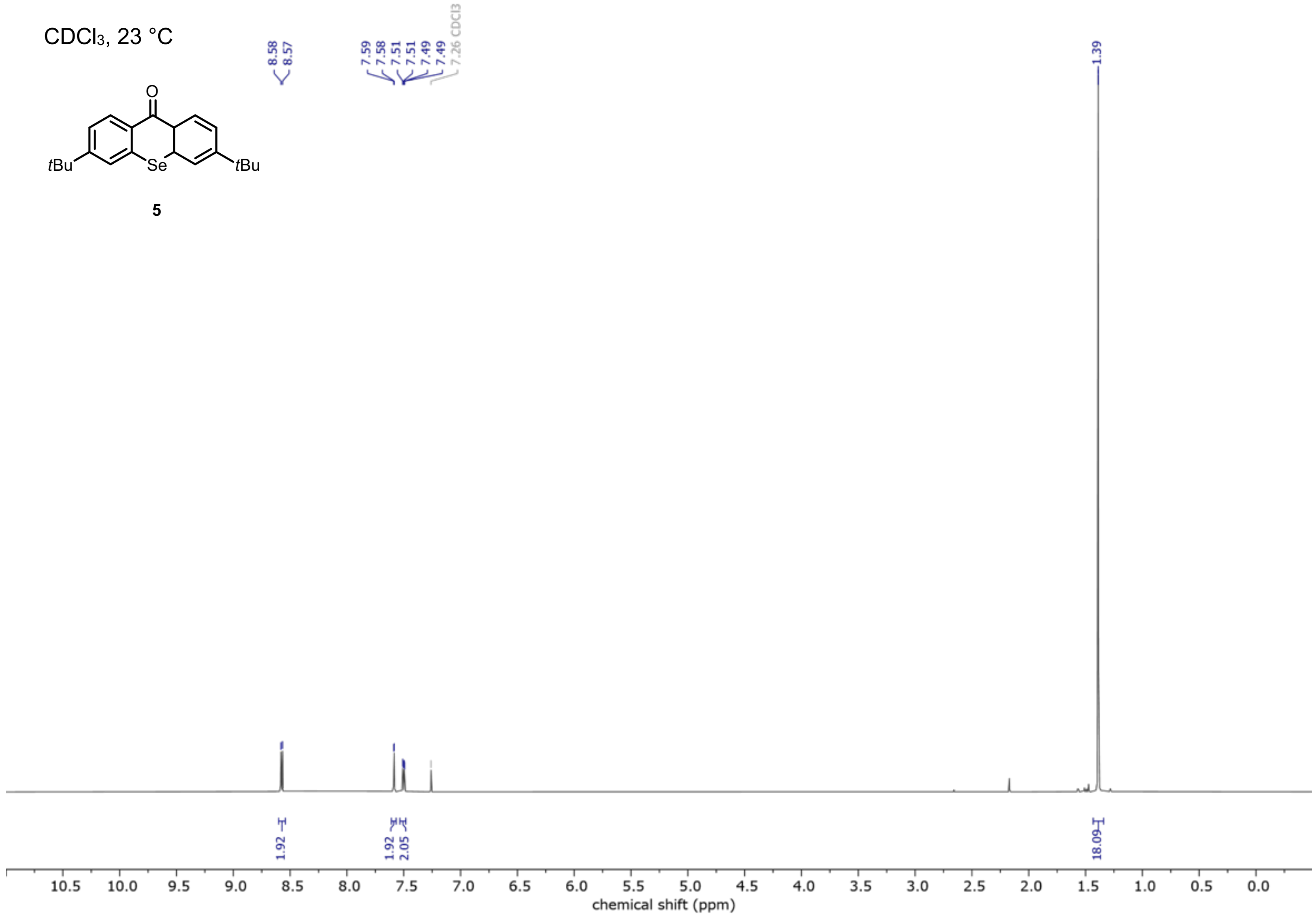

^{13}C NMR OF 3,6-DI-*TERT*-BUTYL-SELENOXANTHEN-9-ONE (5)

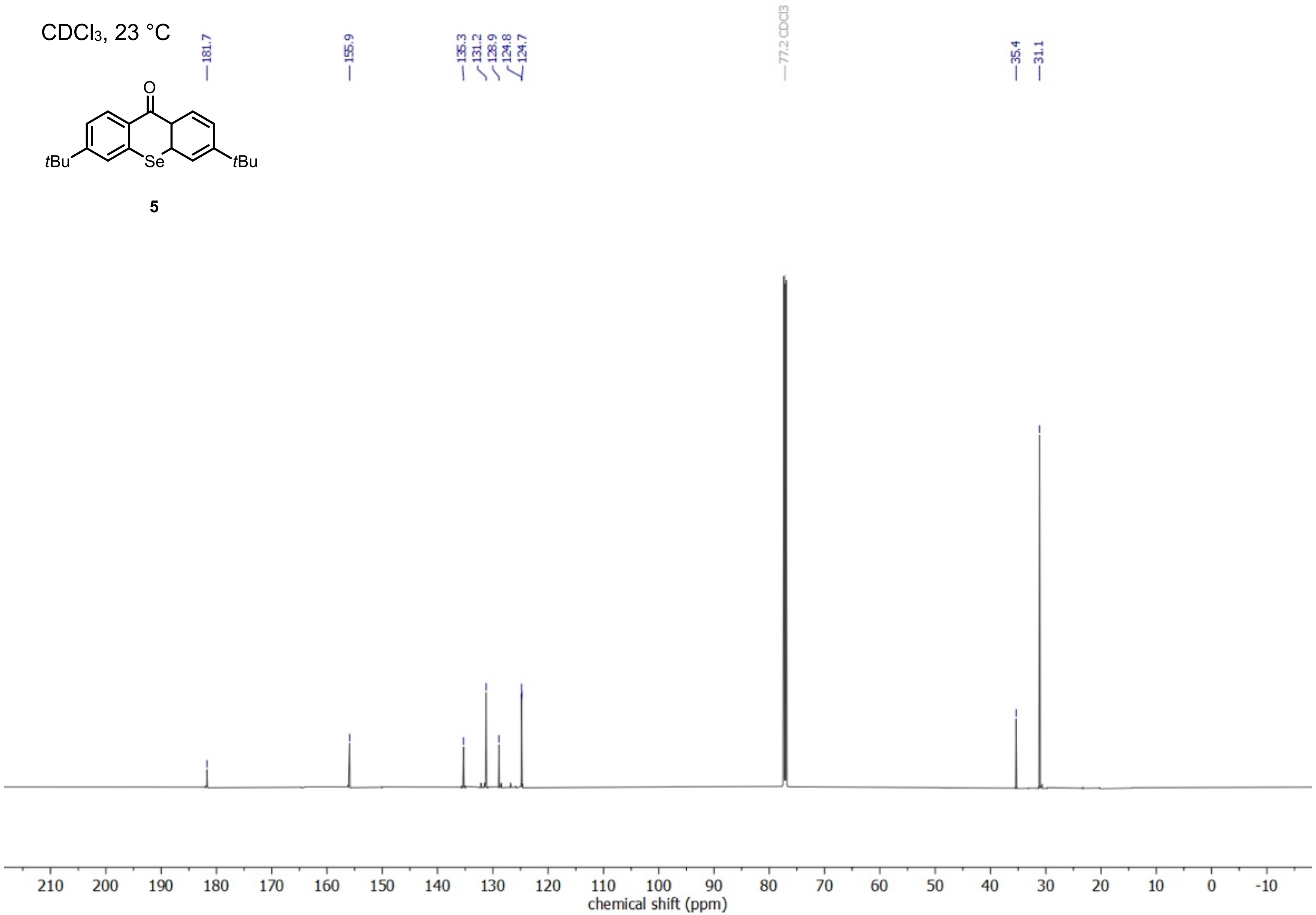

⁷⁷Se NMR of 3,6-di-*tert*-butyl-selenoxanthen-9-one (5)

CDCl$_3$, 23 °C

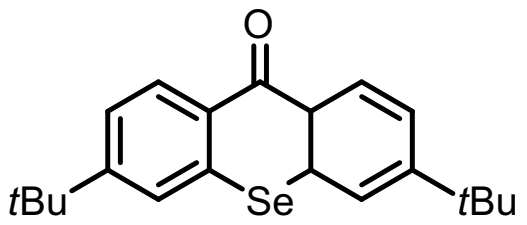

5

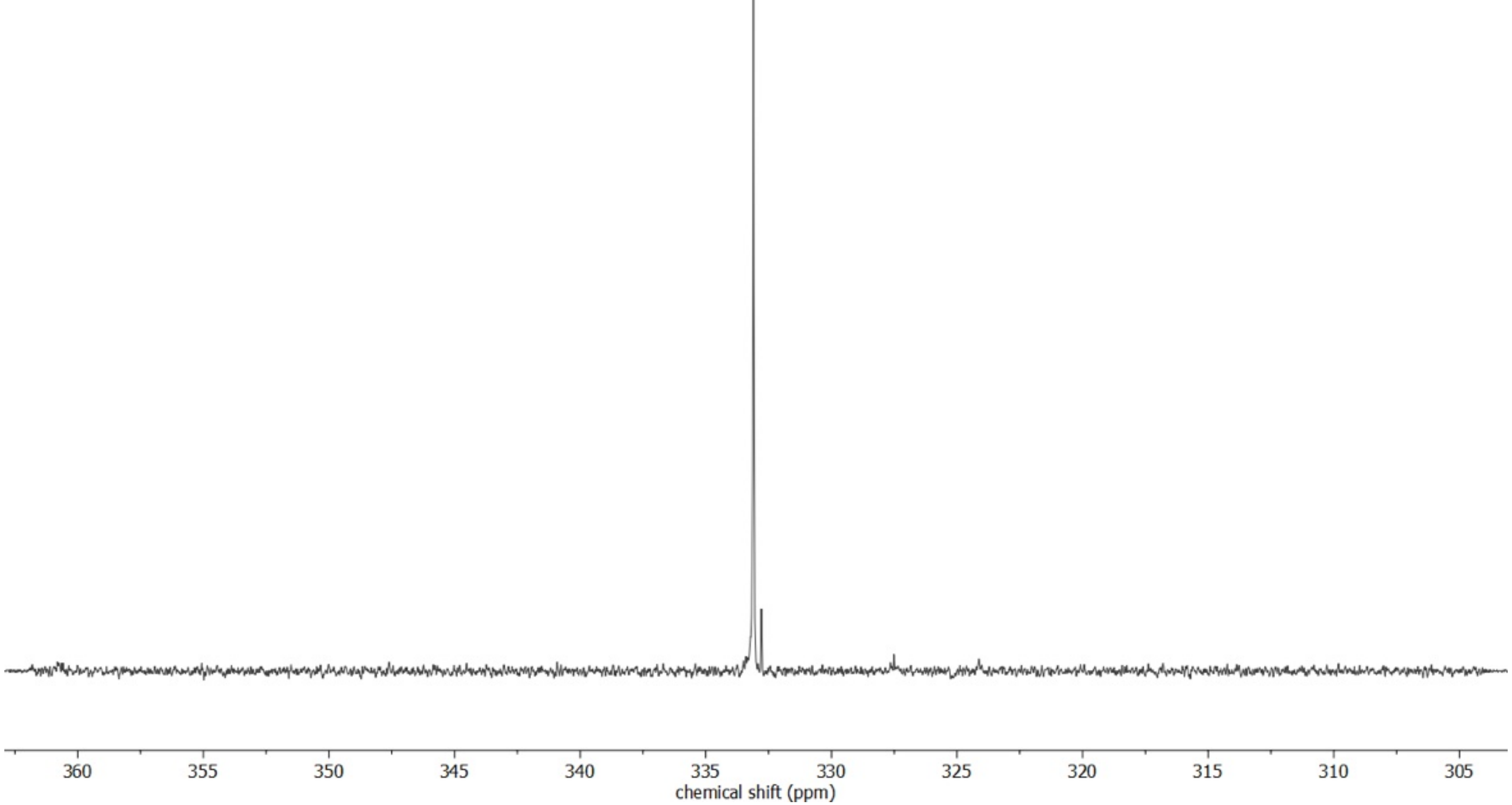

^{1}H NMR OF [4A] BF$_4$

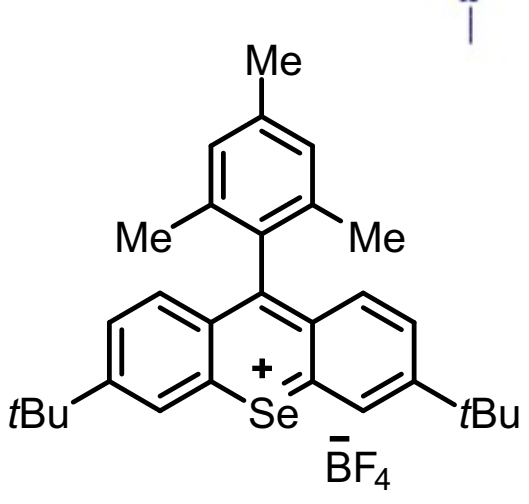

[4a] BF$_4$

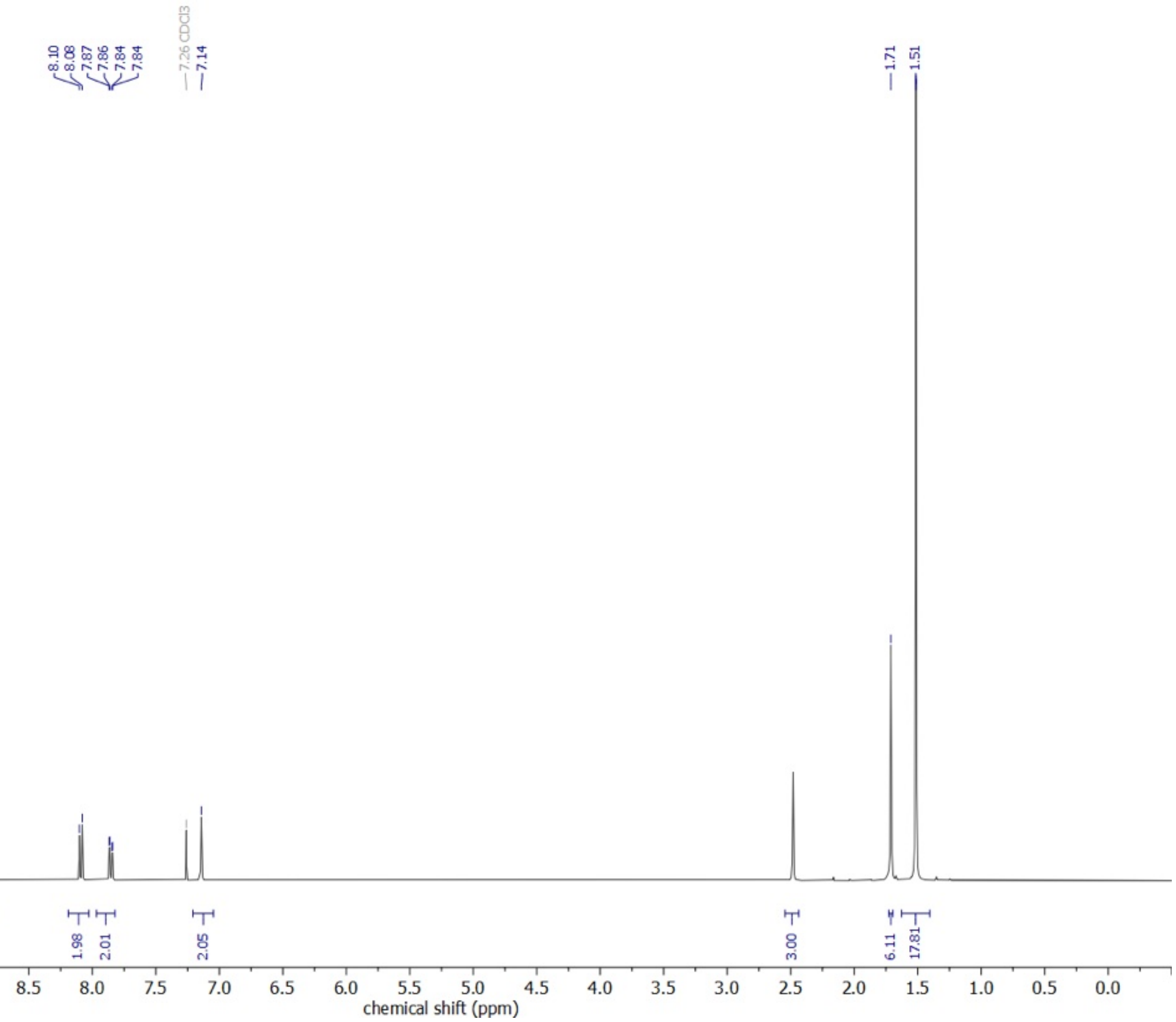

^{13}C NMR of [4a] BF_4

CDCl3, 23 °C

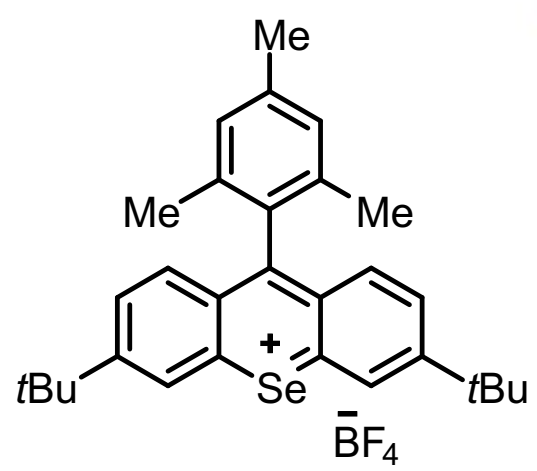

[4a] BF_4

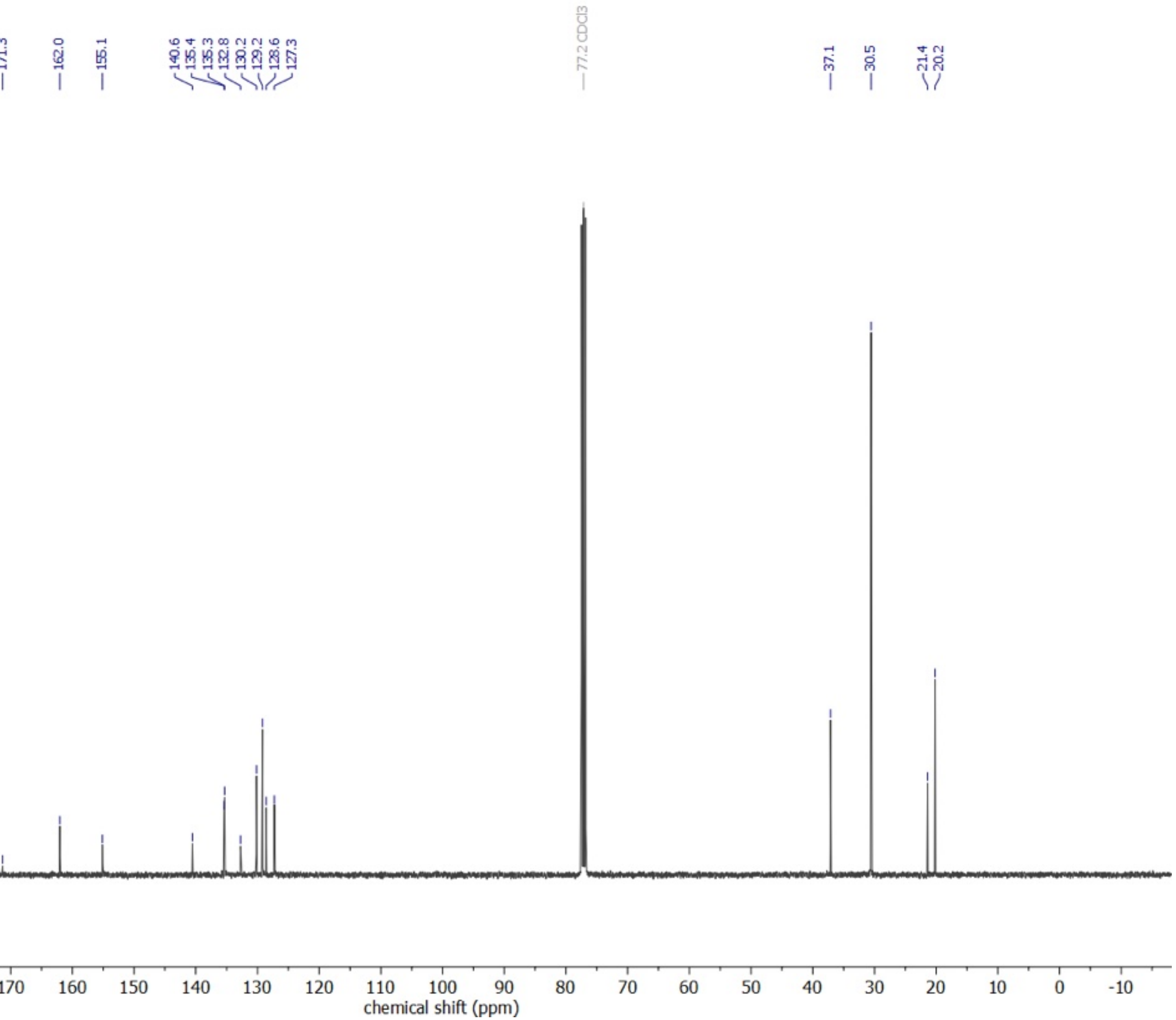

^{19}F NMR OF [4A] BF$_4$

CDCl$_3$, 23 °C

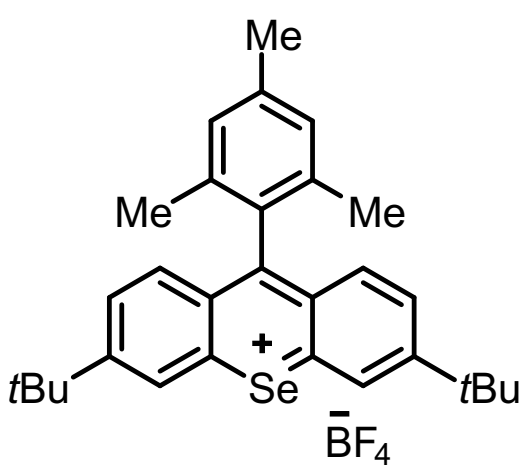

[4a] BF$_4$

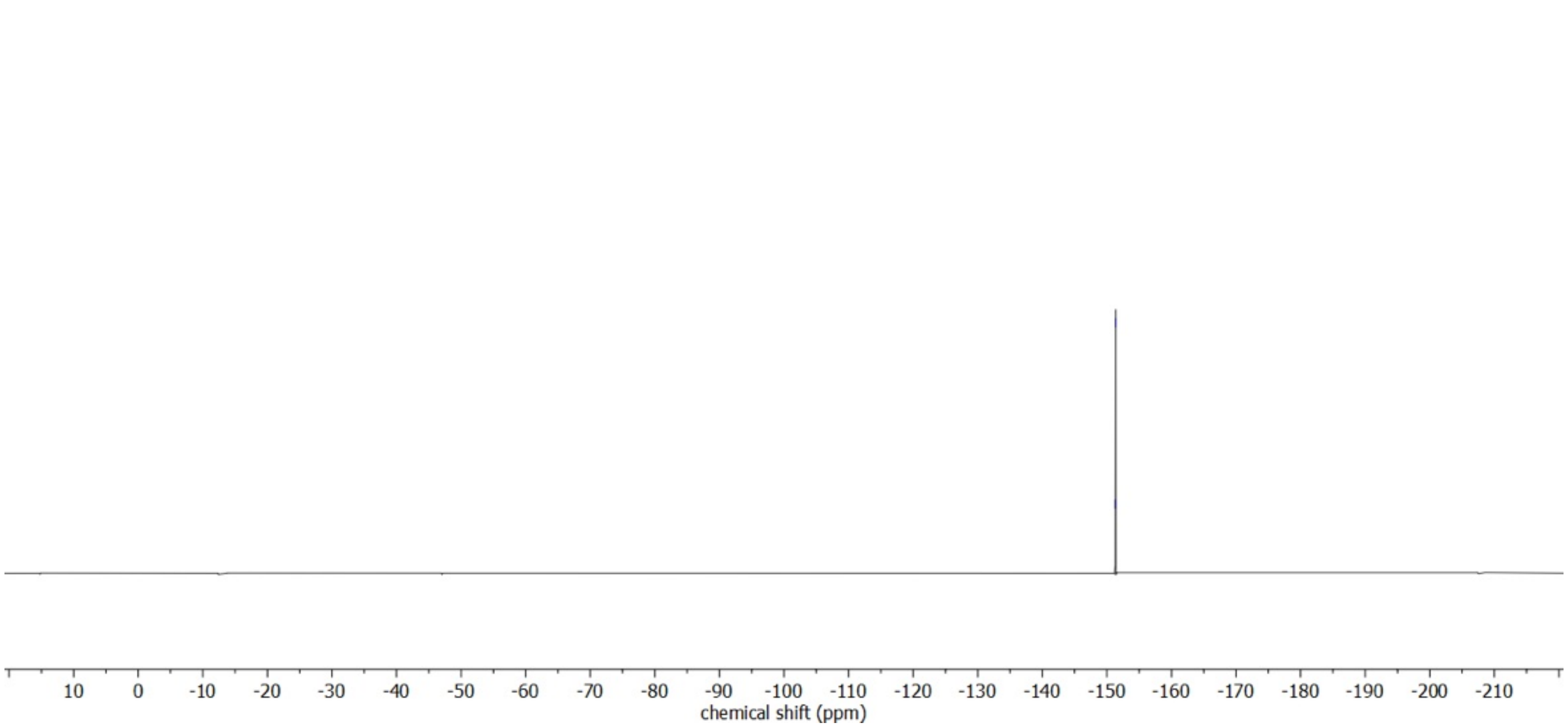

^{77}Se NMR of [4a] BF_4

CDCl3, 23 °C

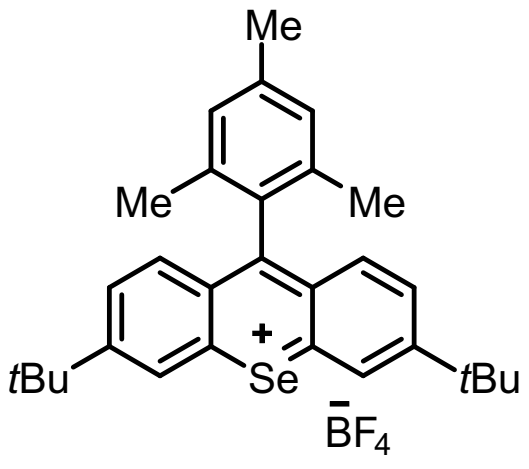

[4a] BF_4

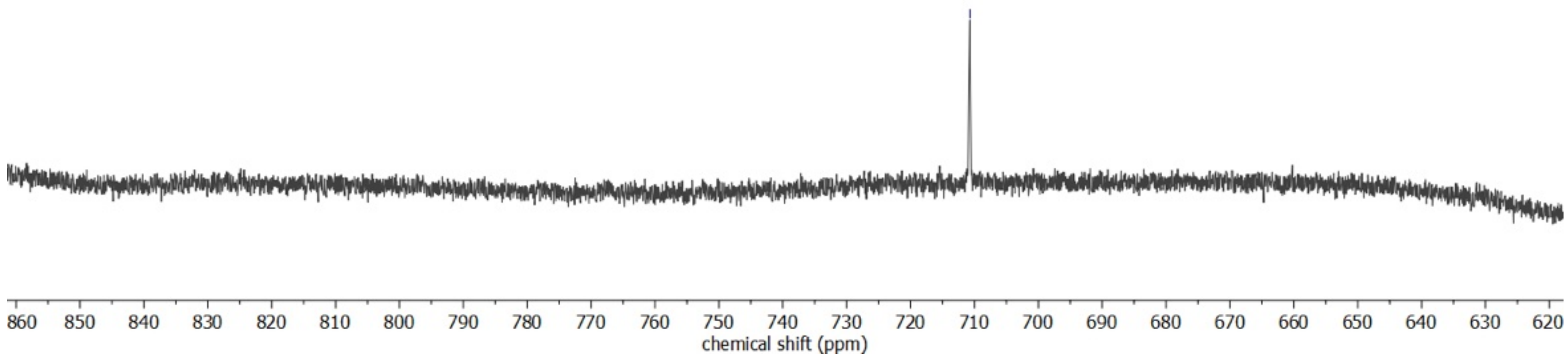

^{1}H NMR OF [4B] BF$_4$

CDCl$_3$, 23 °C

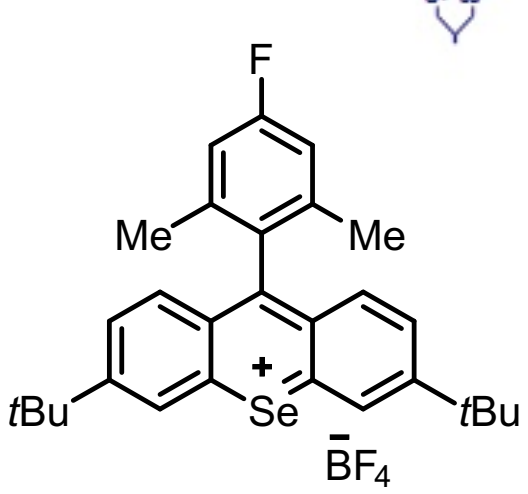

[4b] BF$_4$

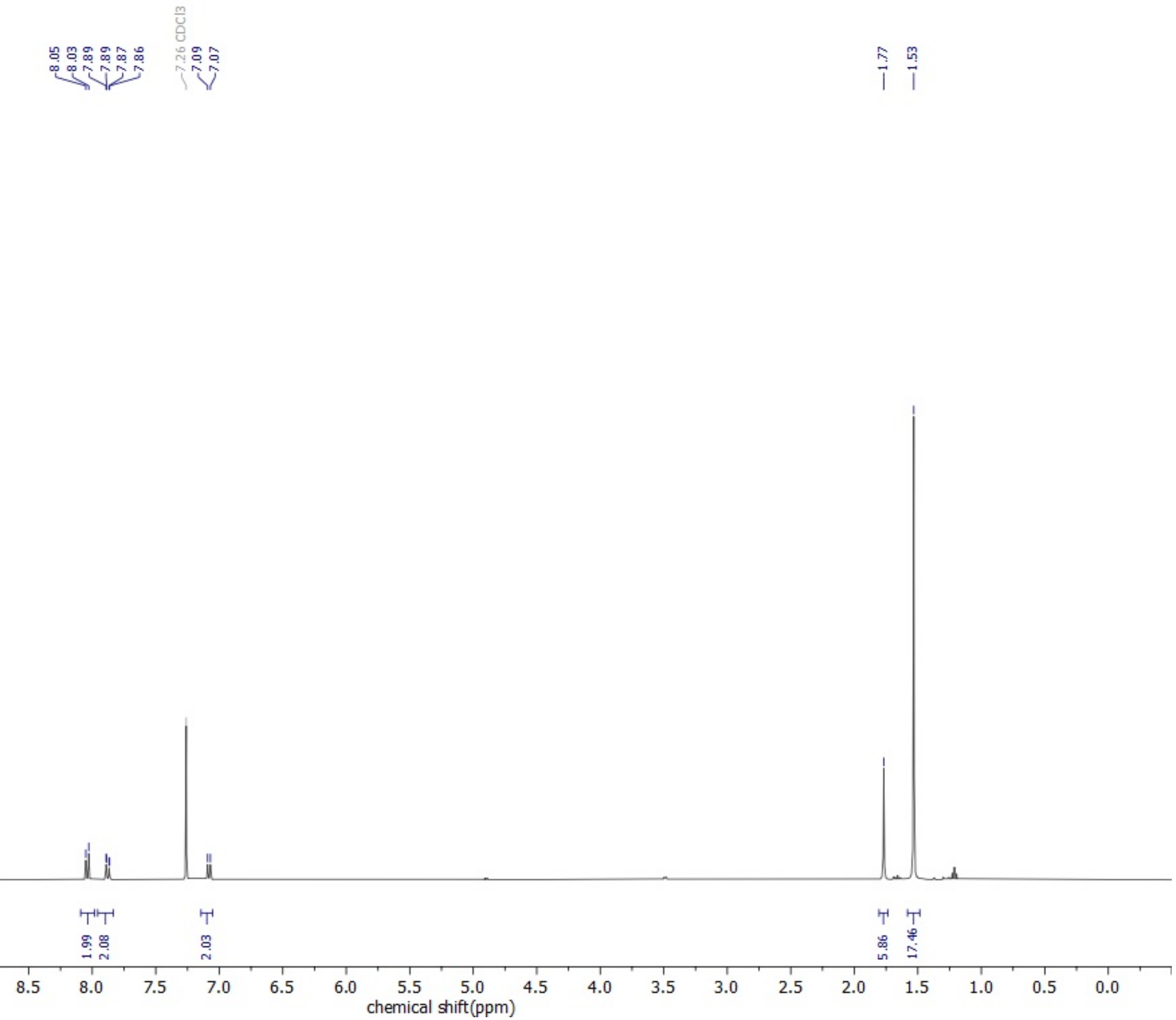

^{13}C NMR of [4b] BF$_4$

CDCl$_3$, 23 °C

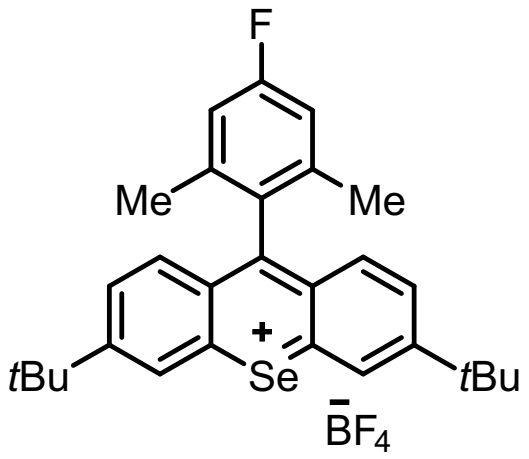

[4b] BF$_4$

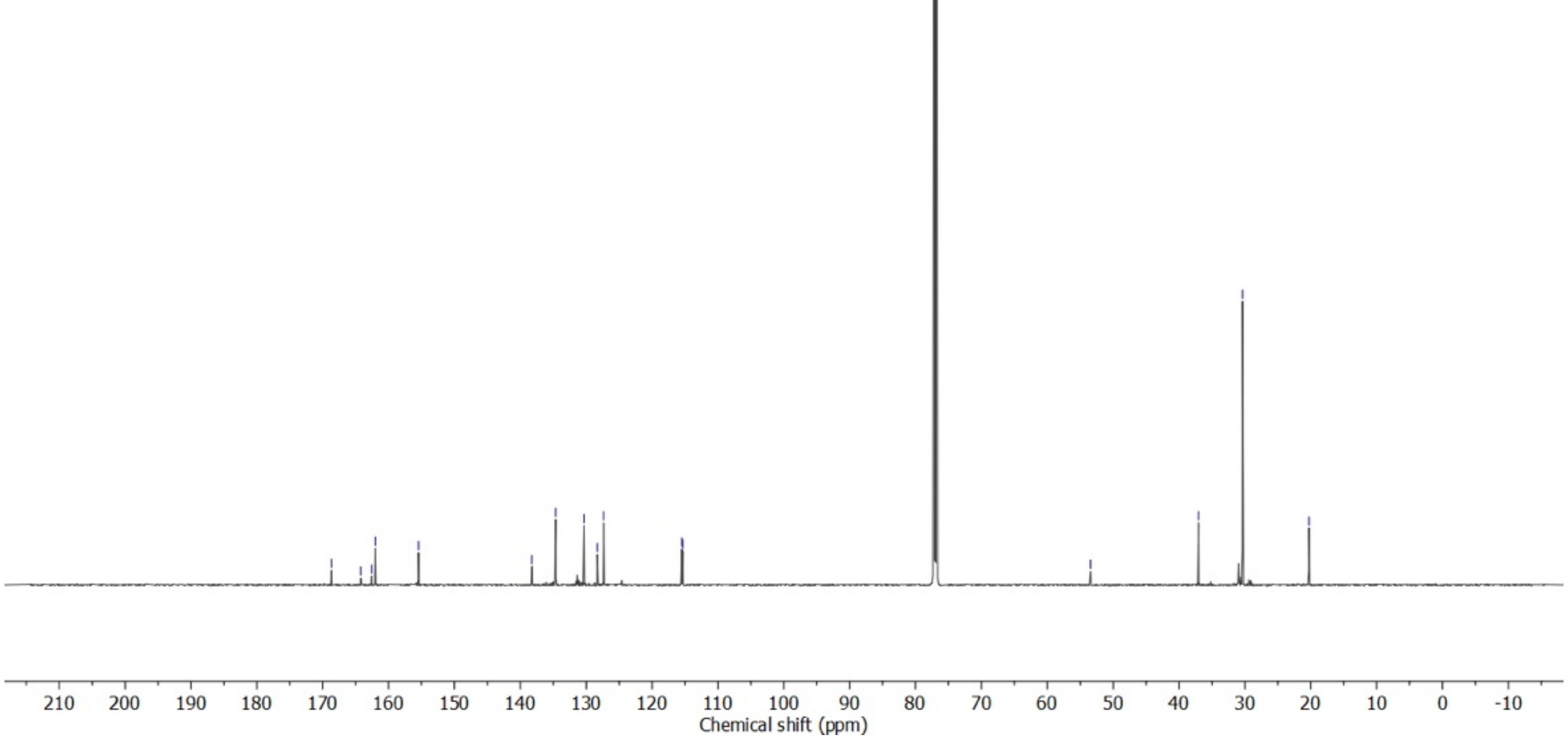

^{19}F NMR OF [4B] BF$_4$

CDCl$_3$, 23 °C

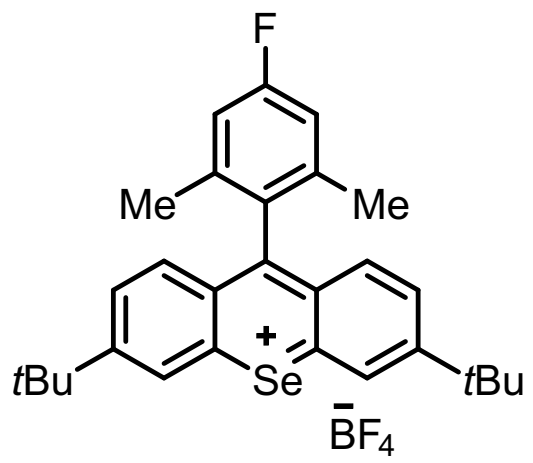

[4b] BF$_4$

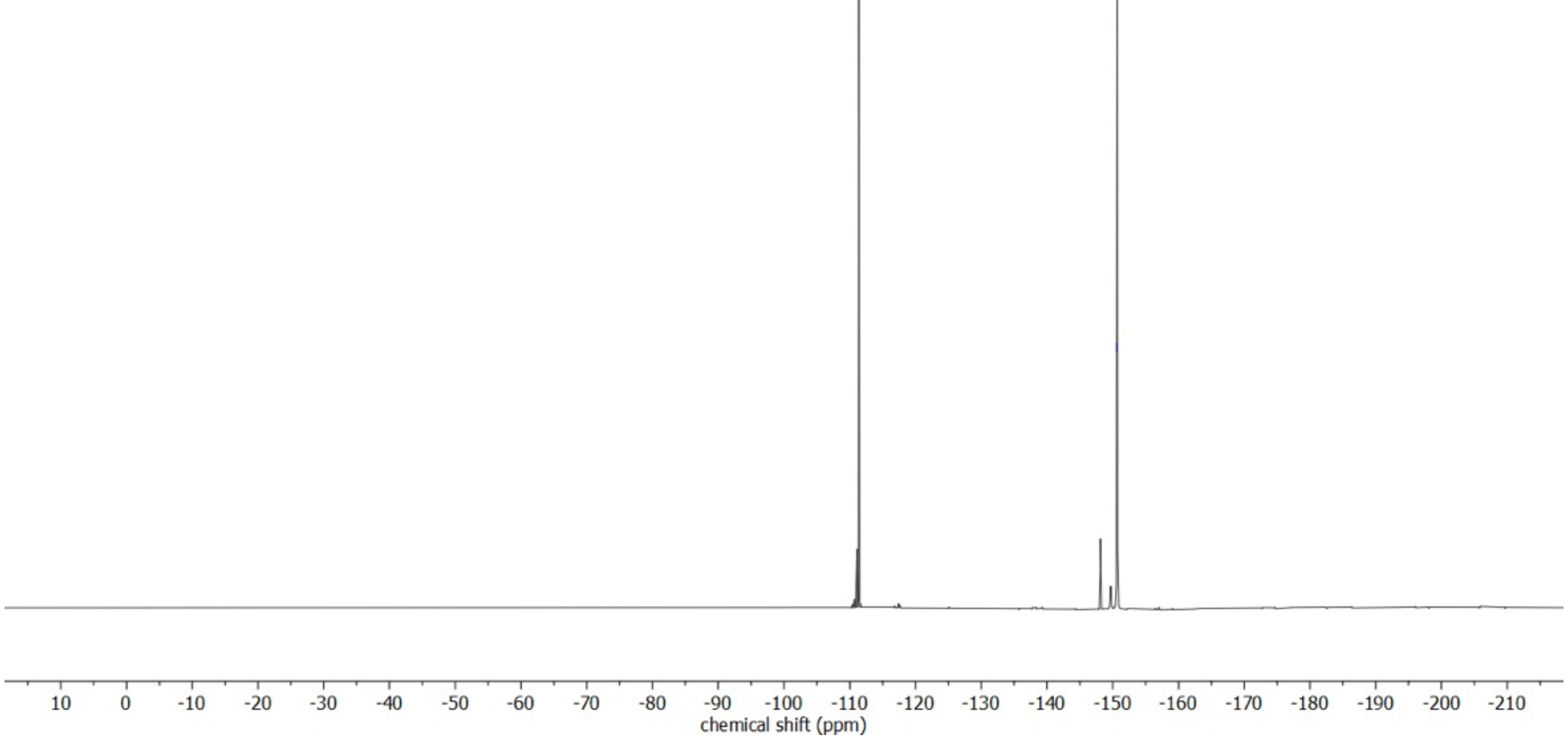

^{77}Se NMR of [4b] BF$_4$

CDCl$_3$, 23 °C

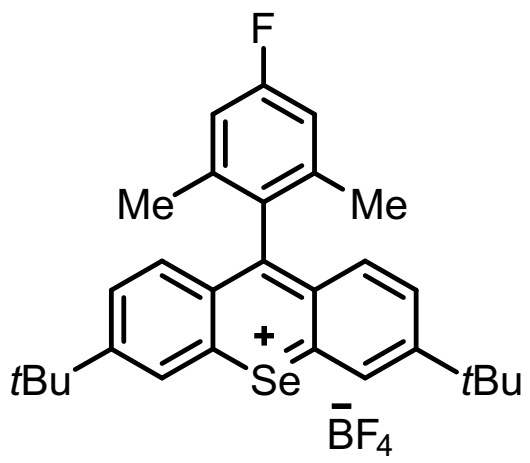

[4b] BF$_4$

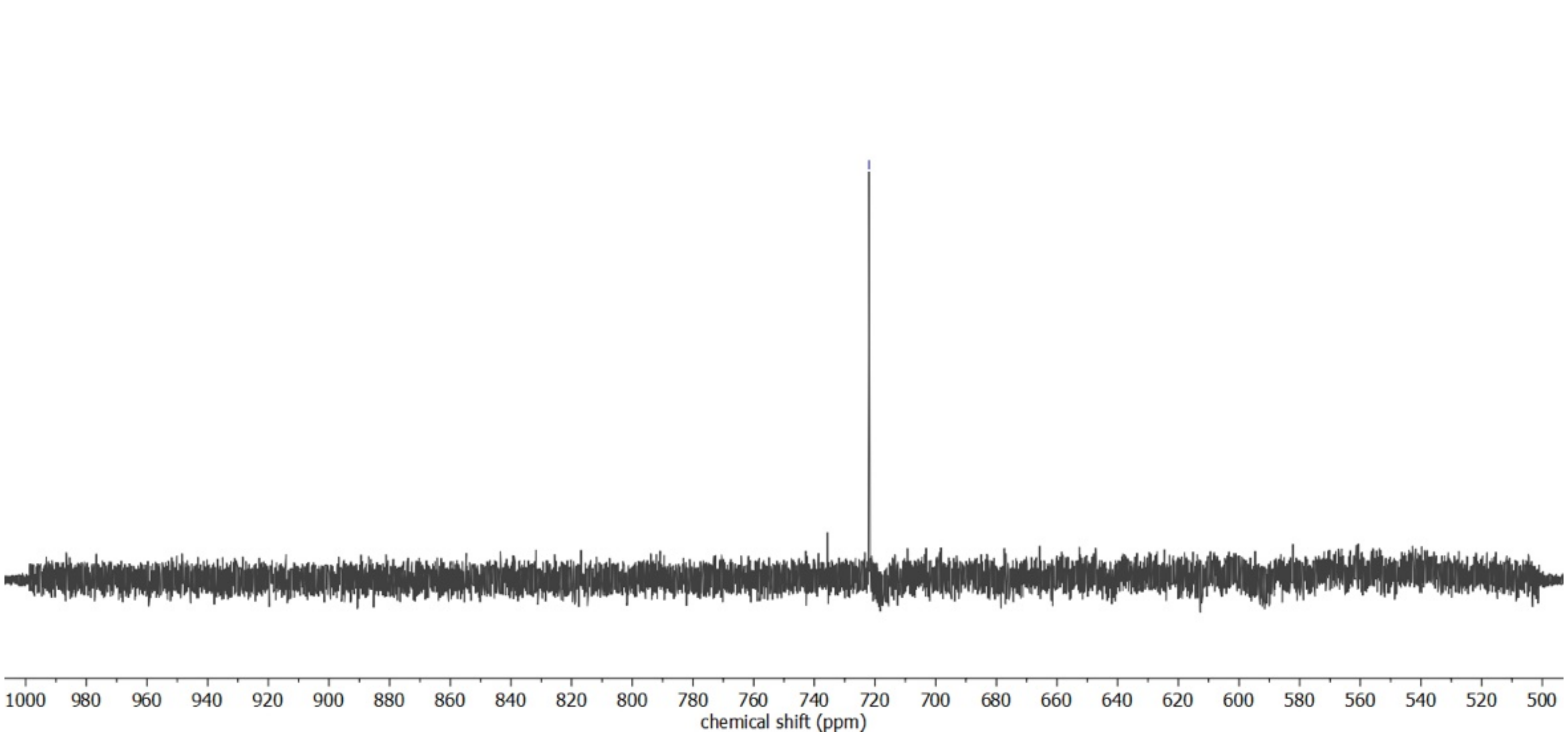

^{1}H NMR OF [4C] BF4)

CDCl3, 23 °C

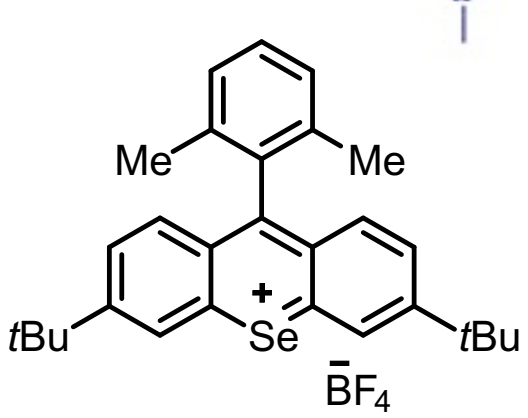

[4c] BF4

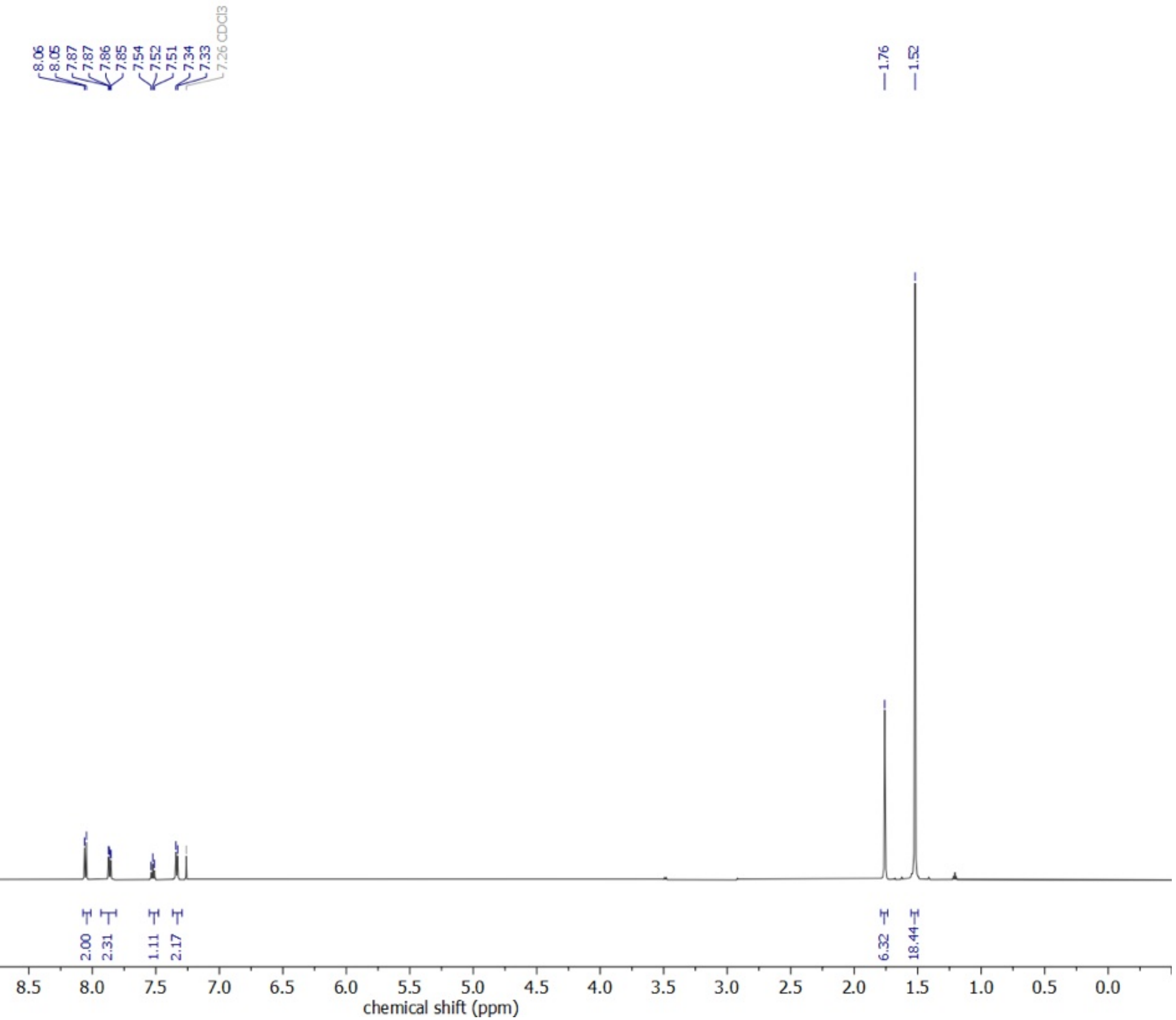

^{13}C NMR of [4c] BF_4

CDCl$_3$, 23 °C

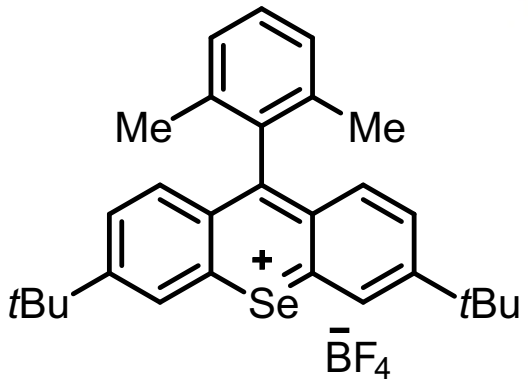

[4c] BF_4

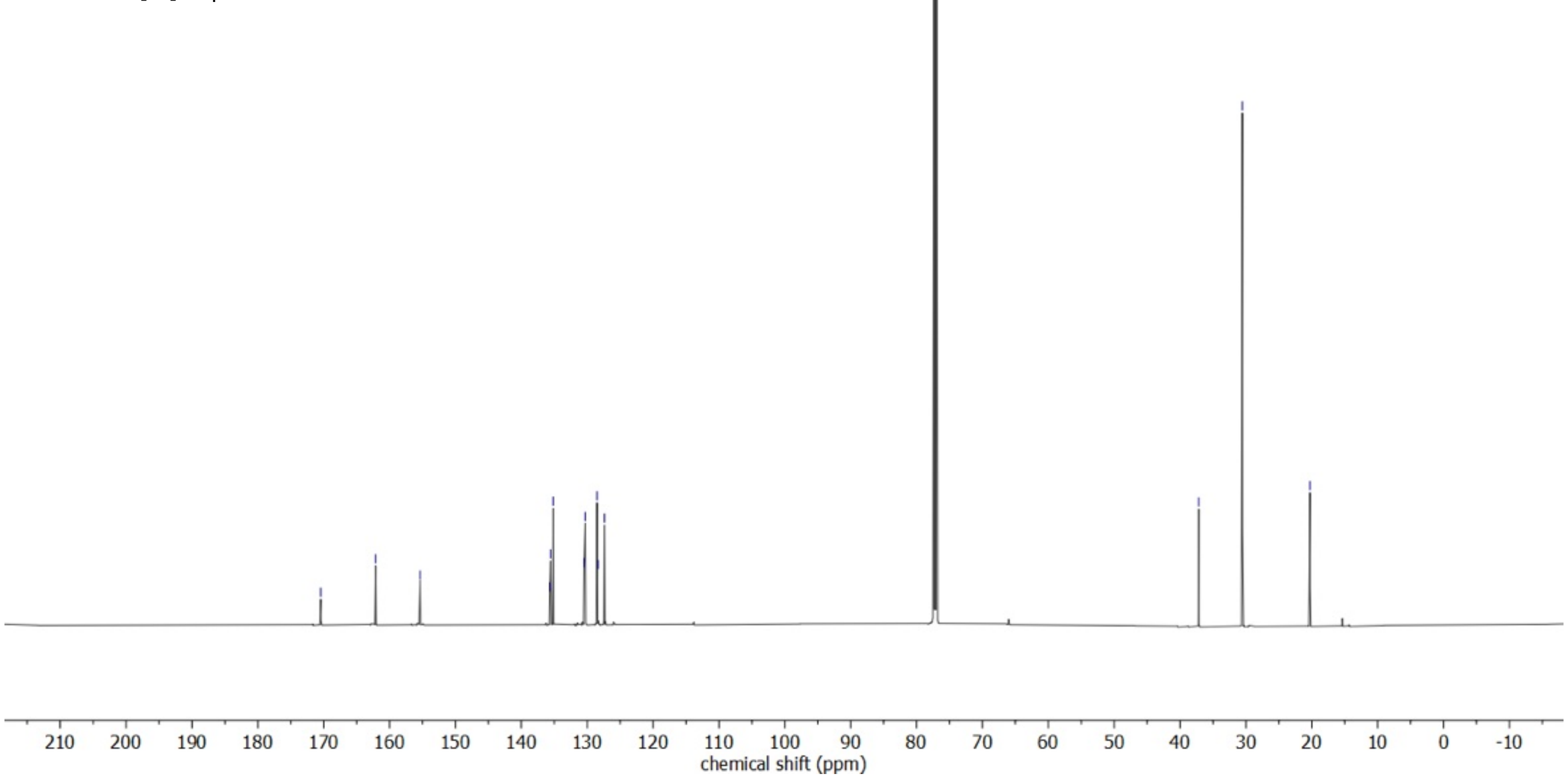

^{19}F NMR of [4c] BF$_4$

CDCl$_3$, 23 °C

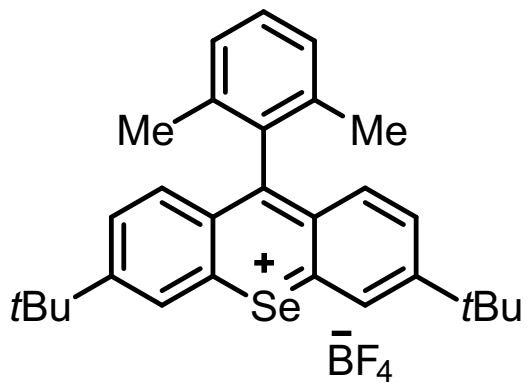

[4c] BF$_4$

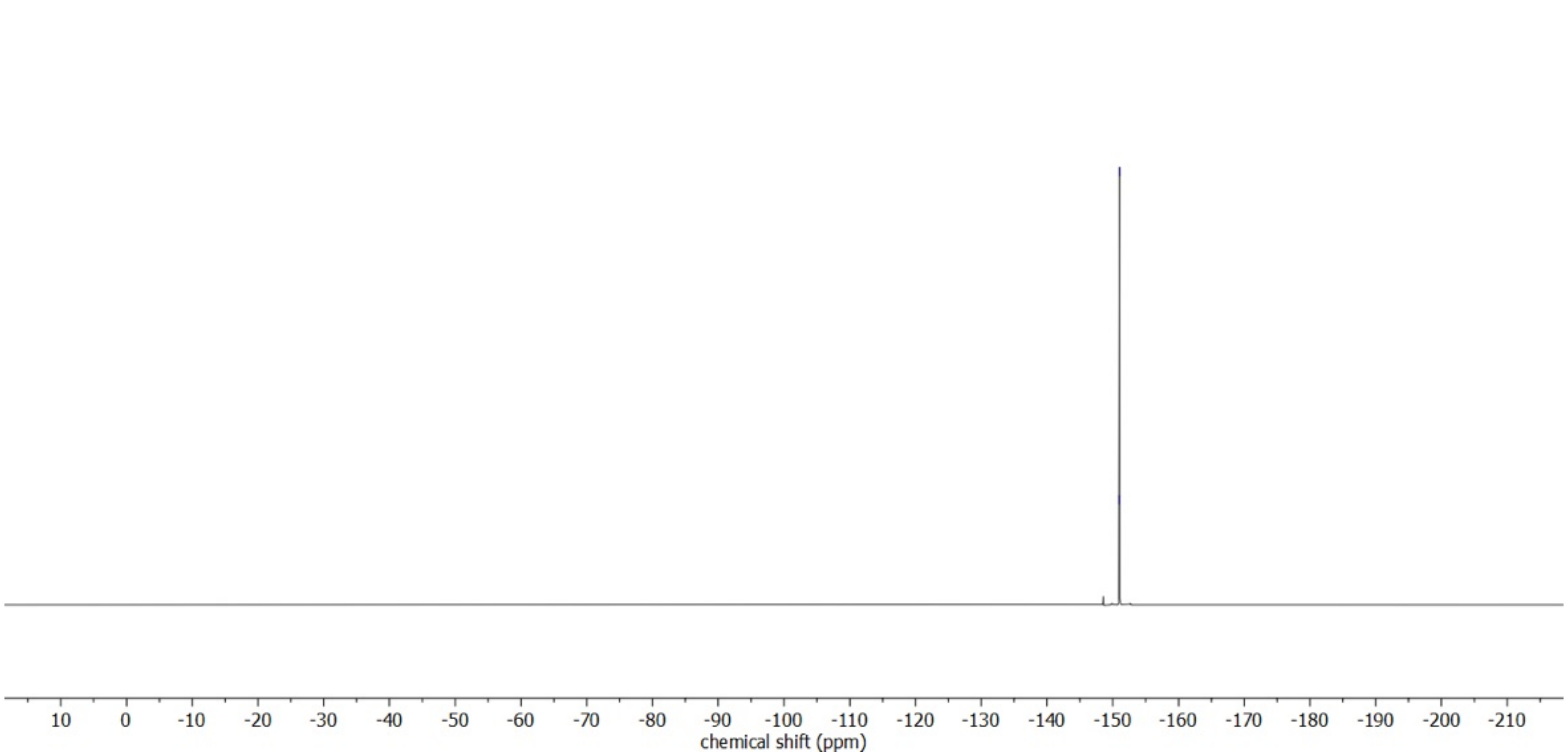

^{77}Se NMR of [4c] BF$_4$

CDCl$_3$, 23 °C

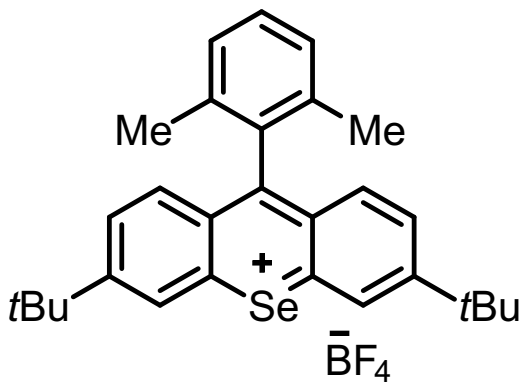

[4c] BF$_4$

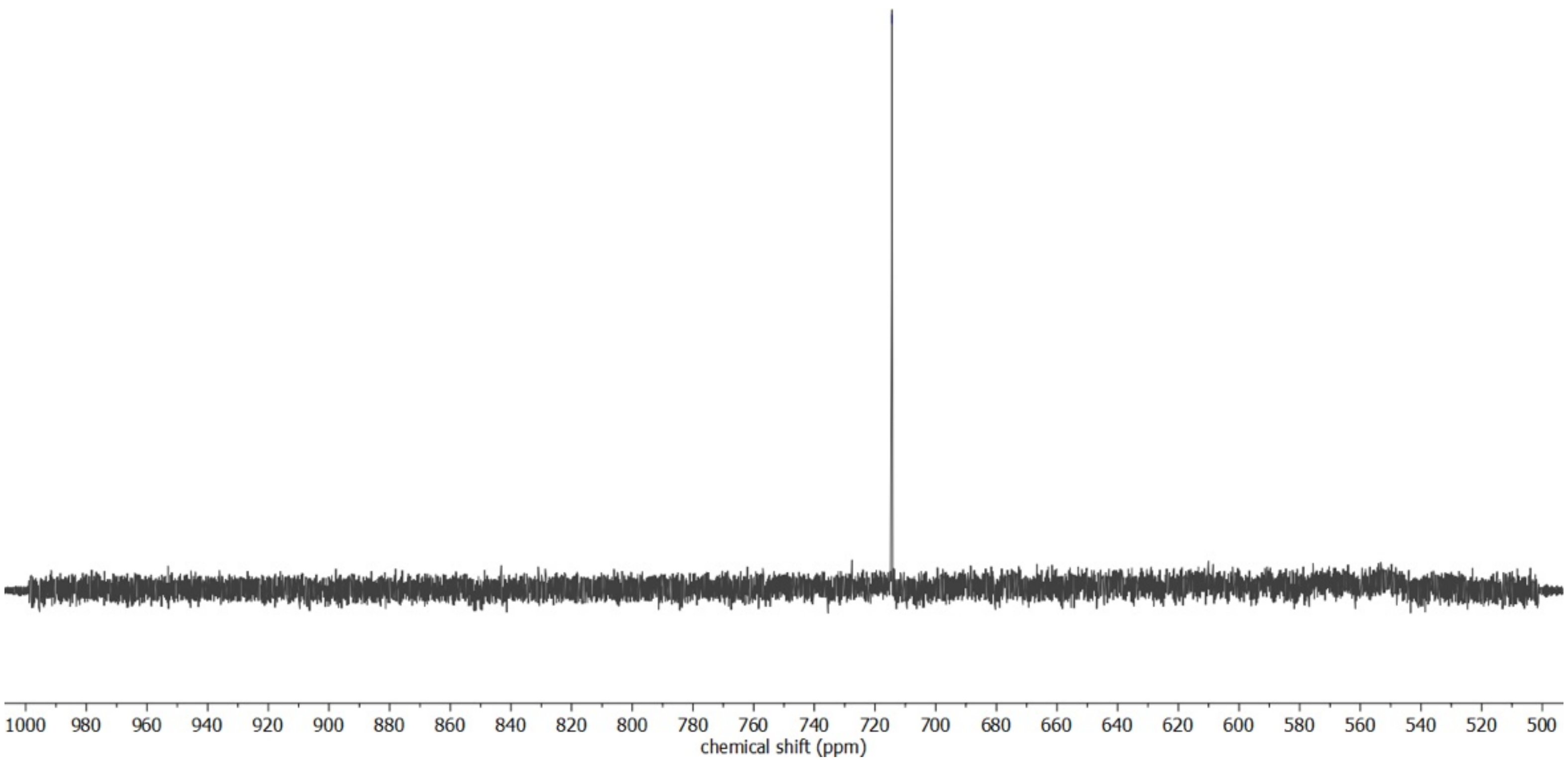

^{1}H NMR of [4d] BF_4)

CDCl3, 23 °C

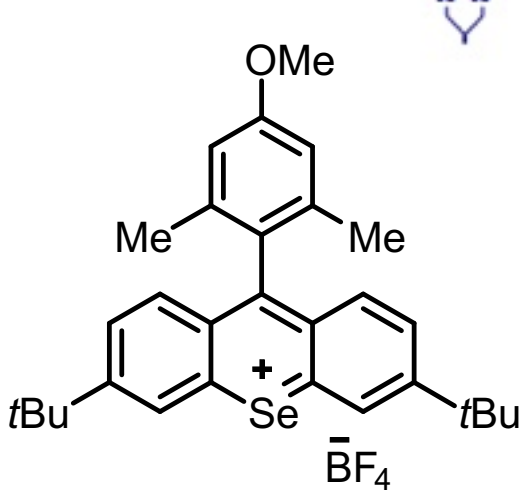

[4d] BF_4

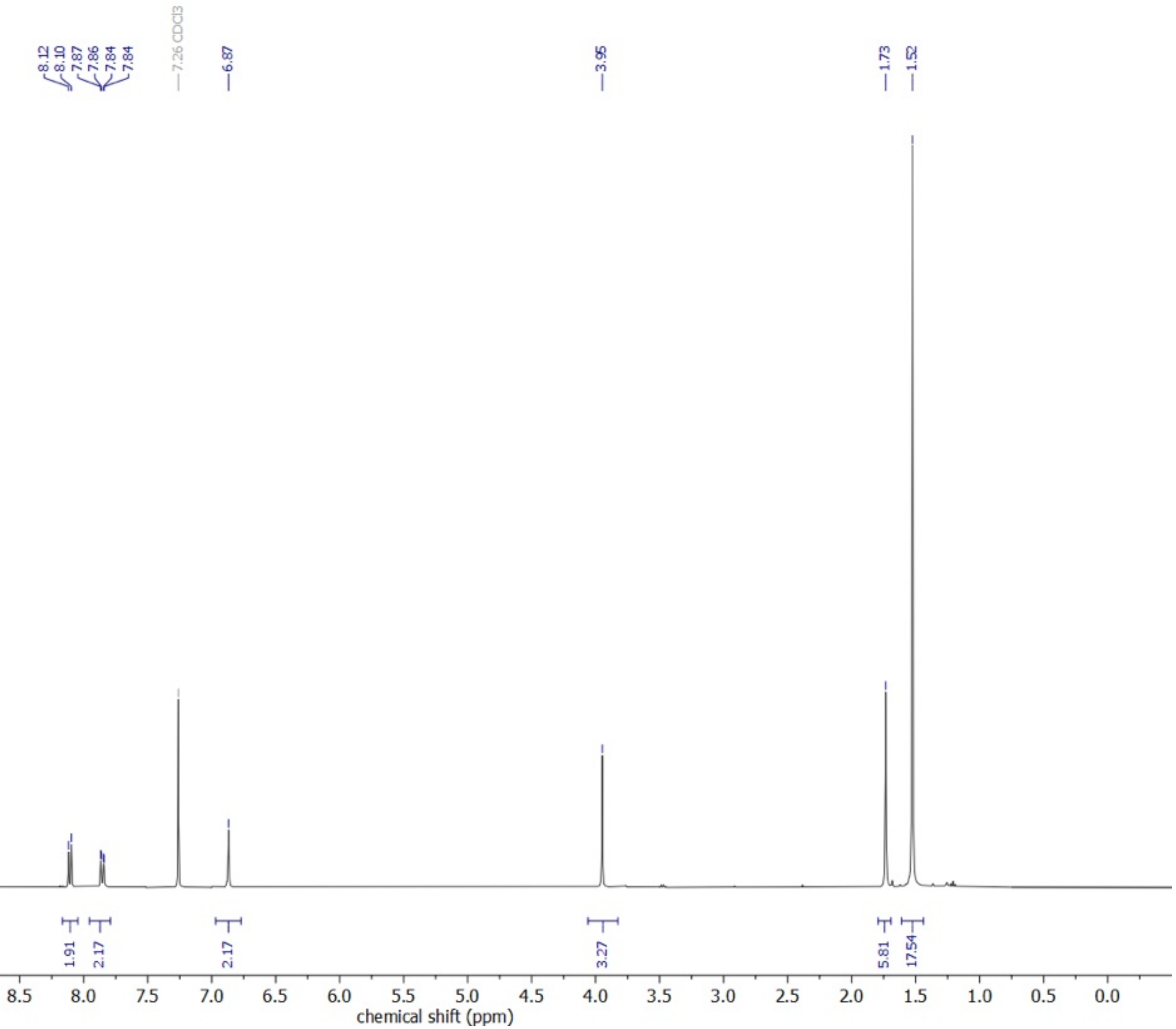

^{13}C NMR of [4d] BF_4

CDCl₃, 23 °C

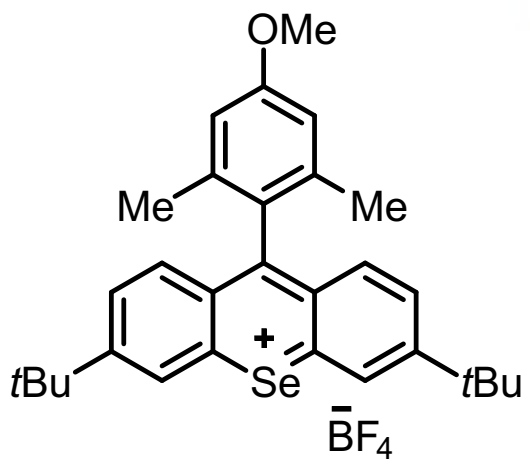

[4d] BF_4

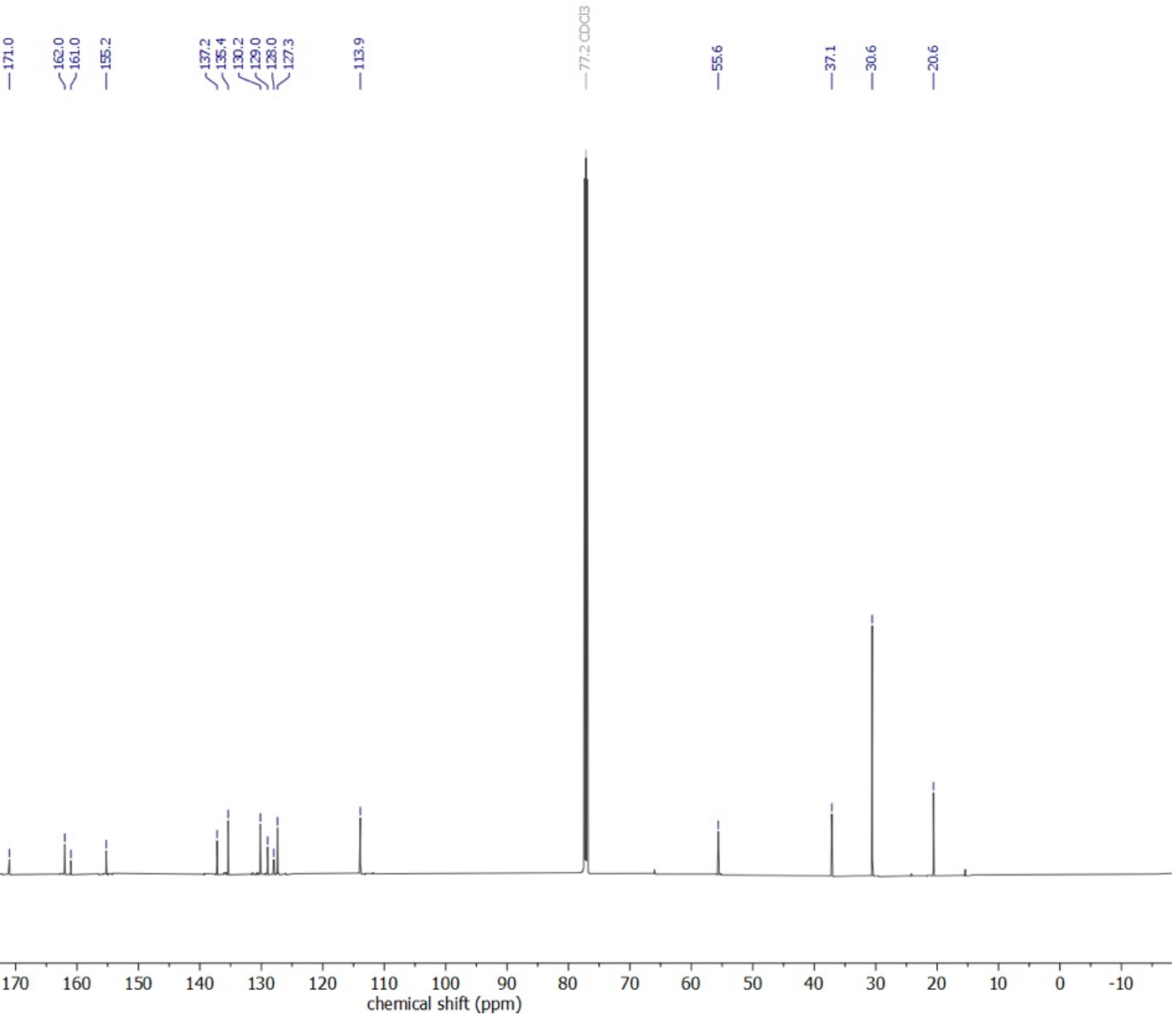

^{19}F NMR of [4d] BF$_4$

CDCl$_3$, 23 °C

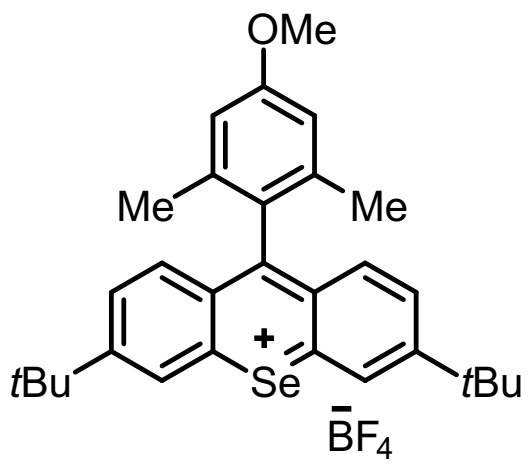

[4d] BF$_4$

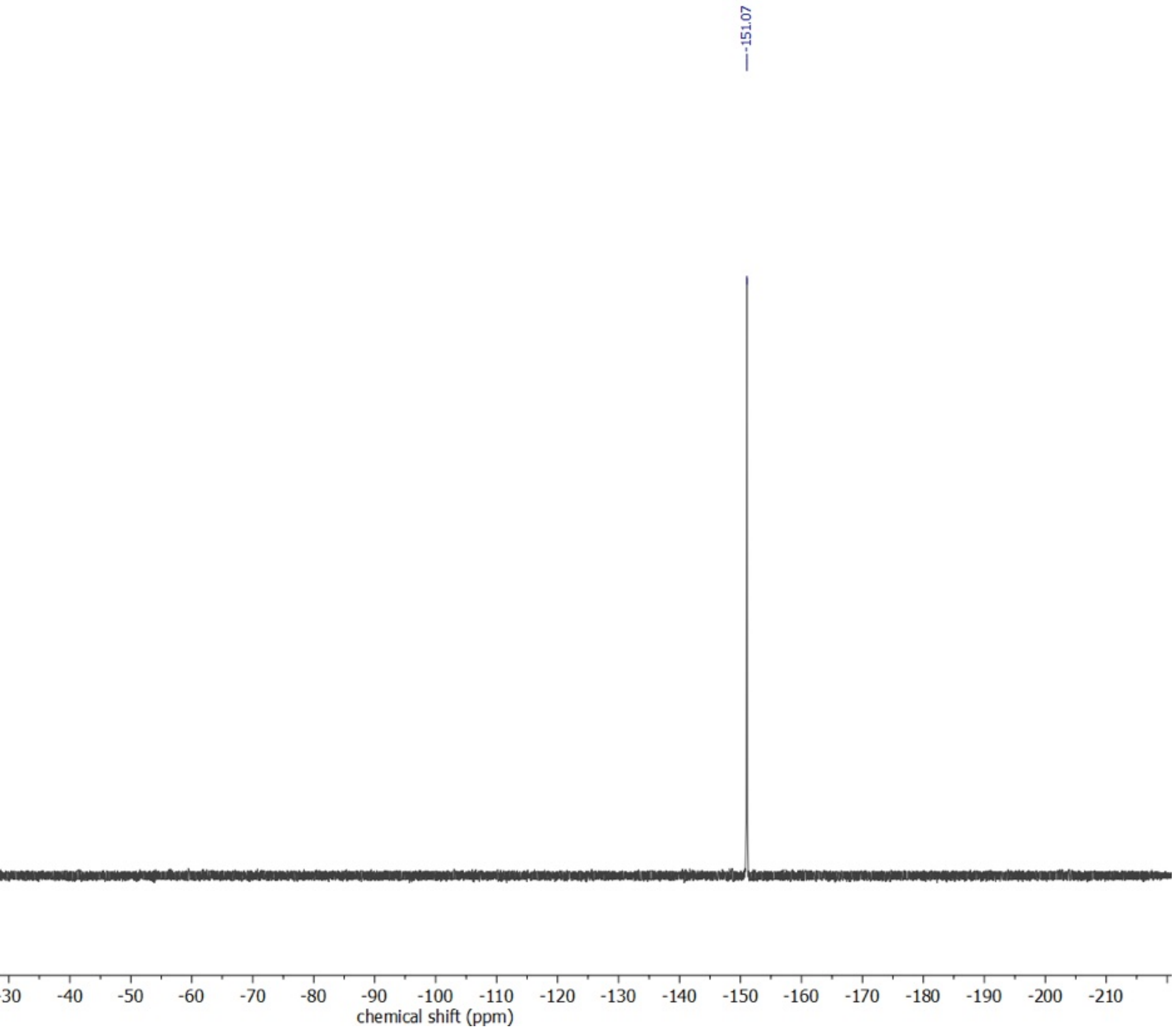

^{77}Se NMR of [4d] BF_4

CDCl3, 23 °C

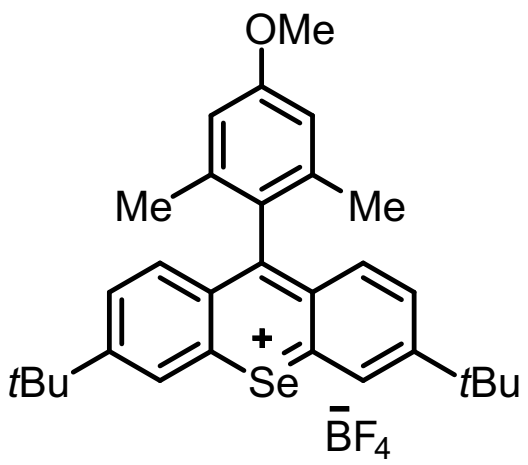

[4d] BF_4

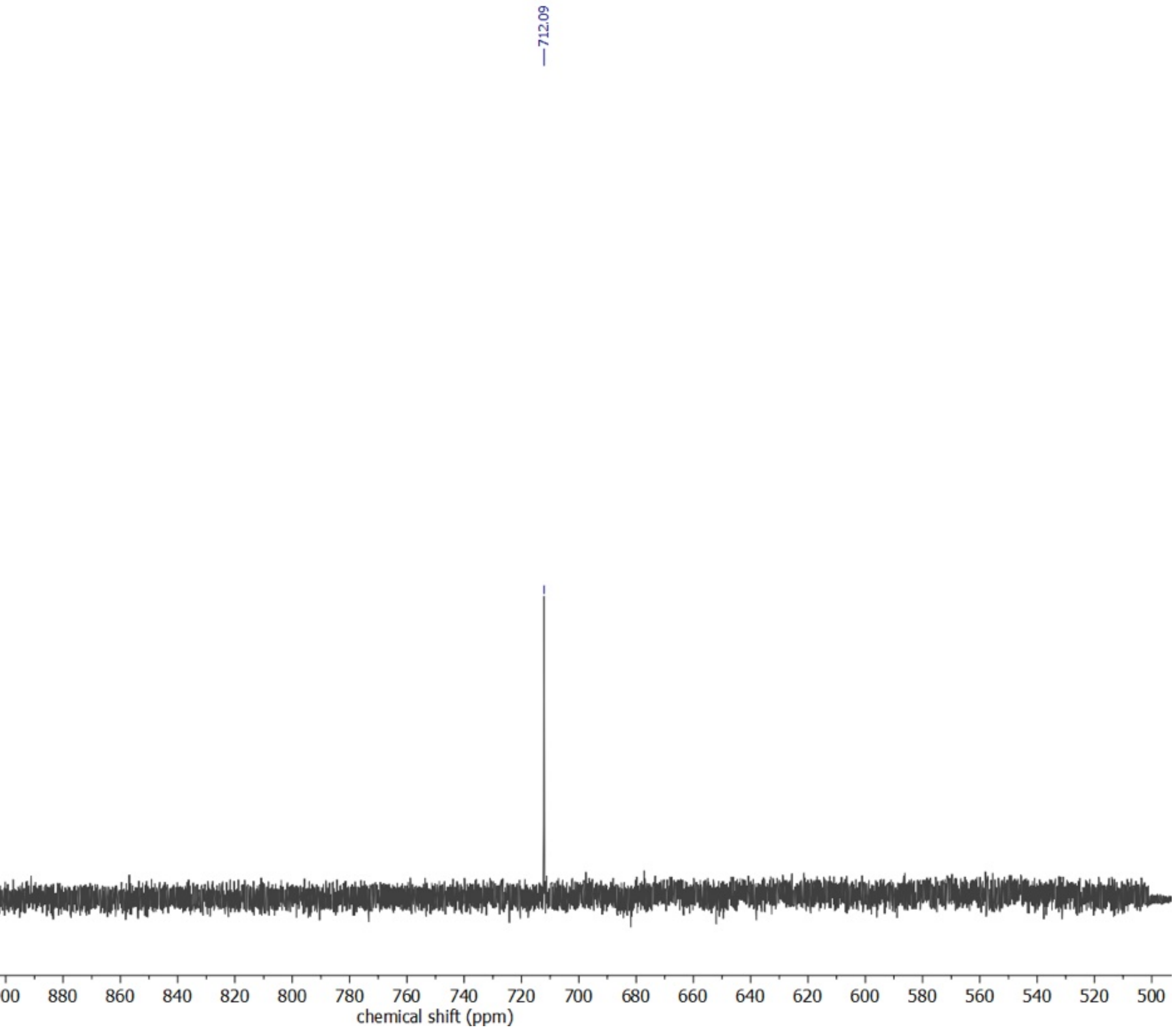

^{1}H NMR OF (1*R*,2*R*)-4'-METHOXY-2,4,5-TRIMETHYL-1,2,3,6-TETRAHYDRO-1,1'-BIPHENYL (9)

$CDCl_3$, 23 °C

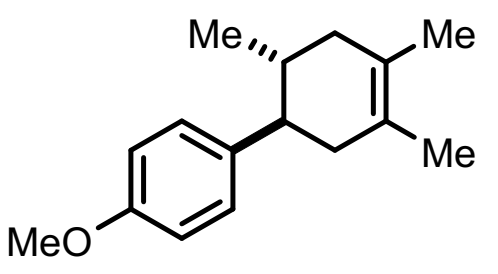

9

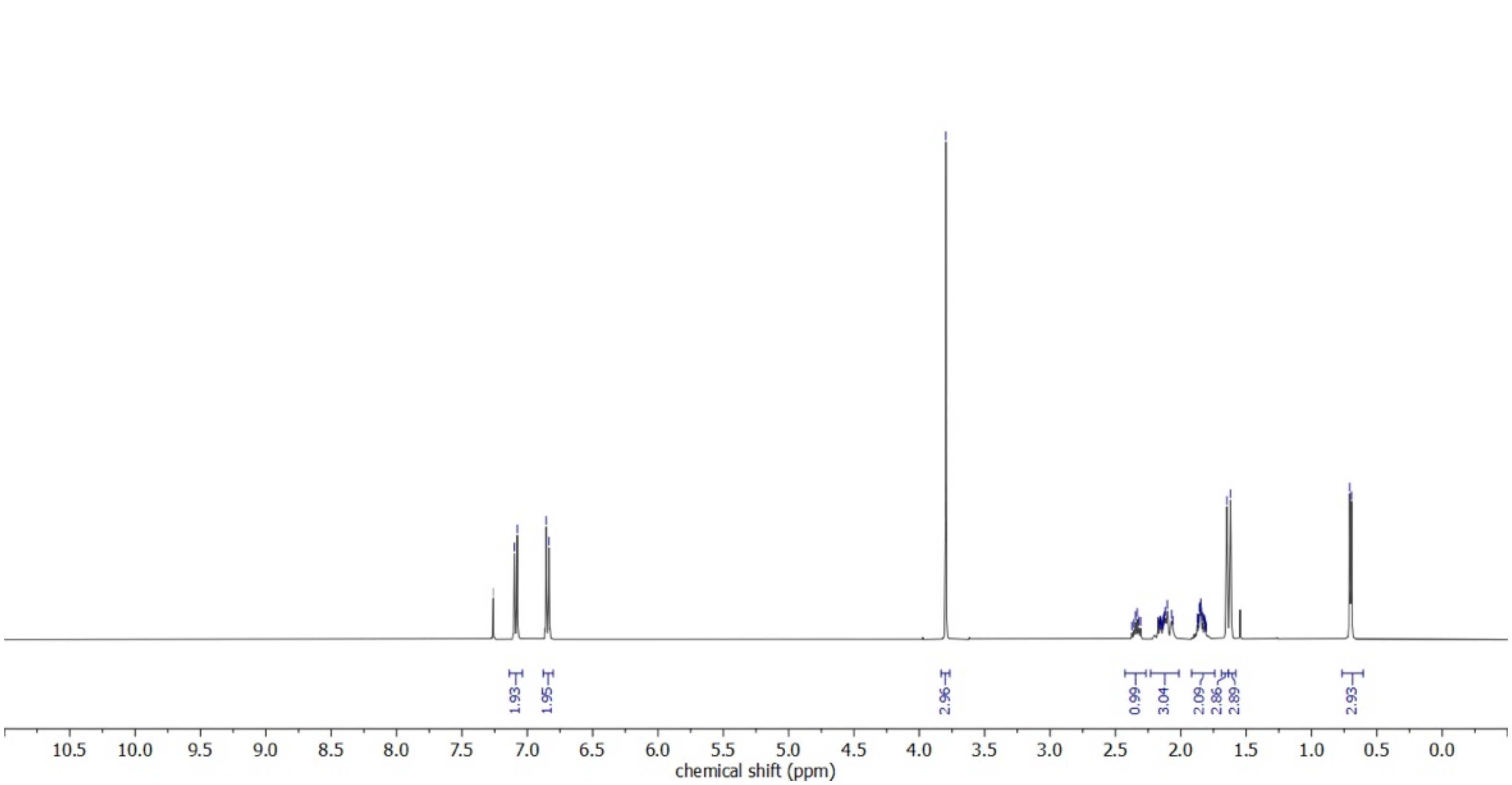

^{13}C NMR of (1*R*,2*R*)-4'-methoxy-2,4,5-trimethyl-1,2,3,6-tetrahydro-1,1'-biphenyl (9)

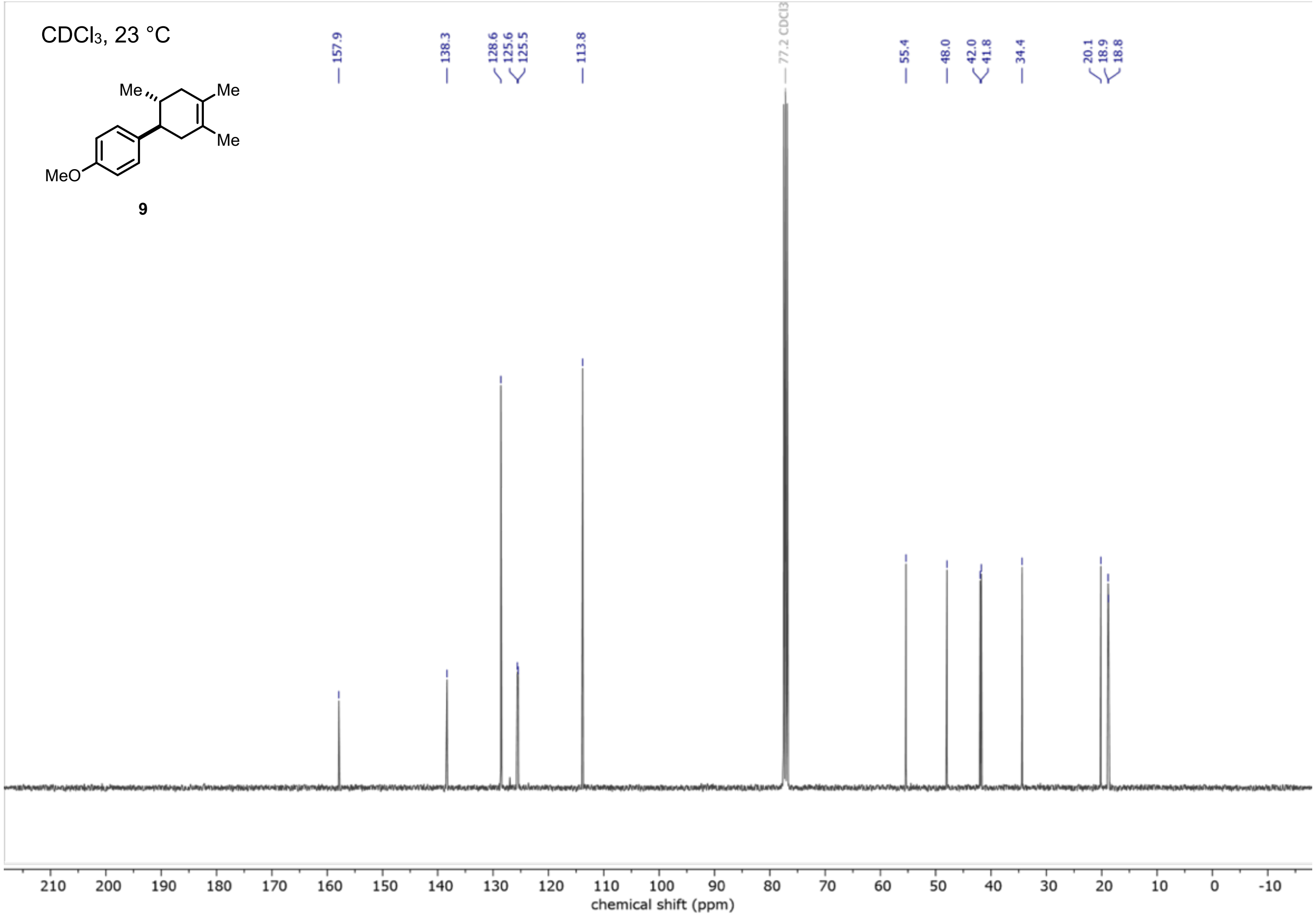

^{1}H NMR OF 3,5-DIMETHYL-1-(2,4,6-TRIMETHOXYPHENYL)-1H-PYRAZOLE (12)

CDCl3, 23 °C

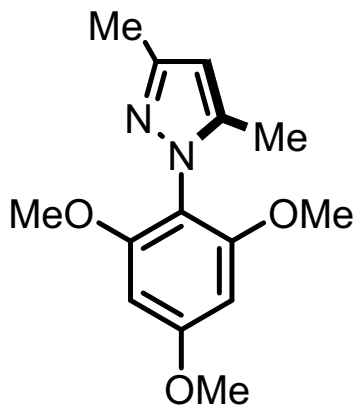

12

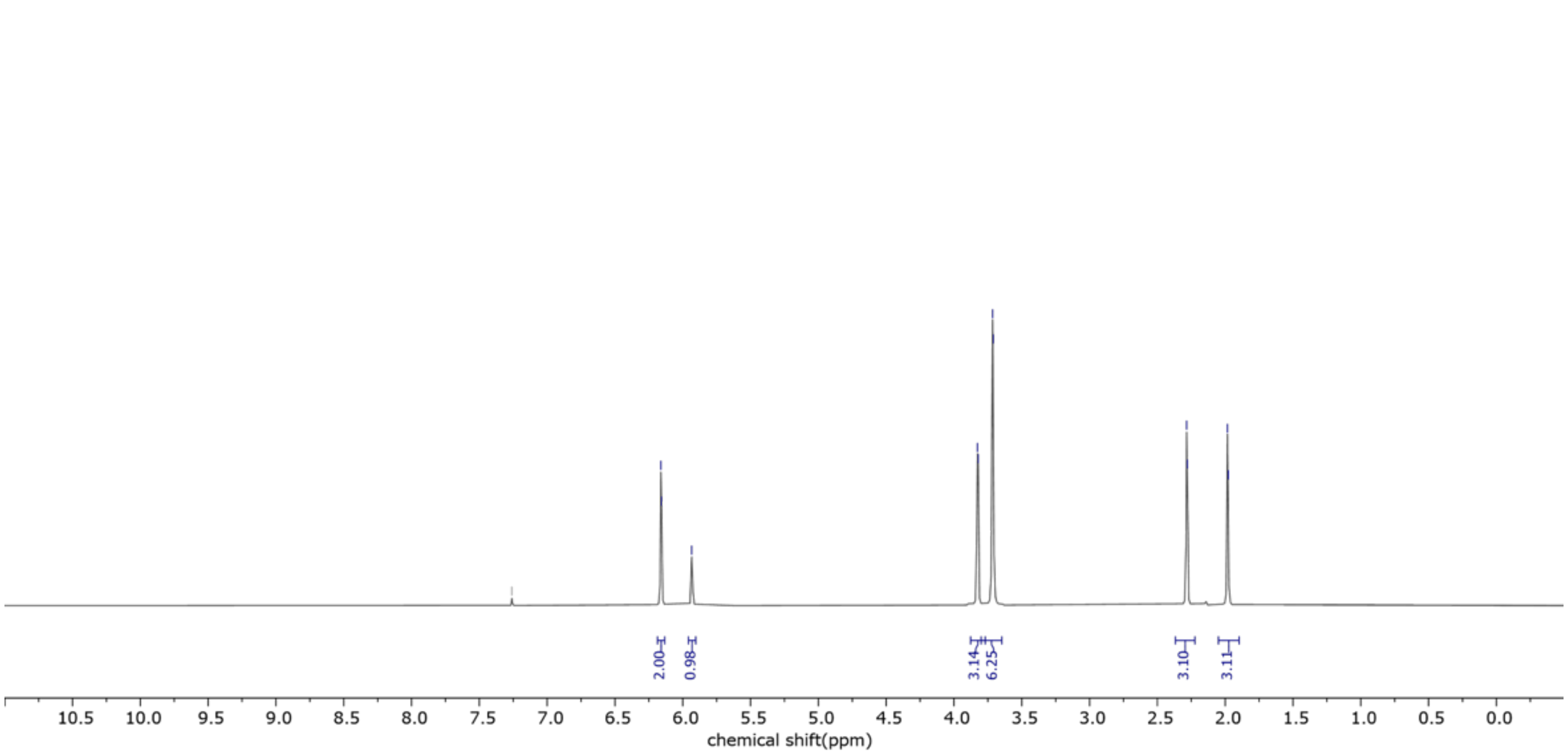

^{13}C NMR OF 3,5-DIMETHYL-1-(2,4,6-TRIMETHOXYPHENYL)-1H-PYRAZOLE (12)

CDCl3, 23 °C

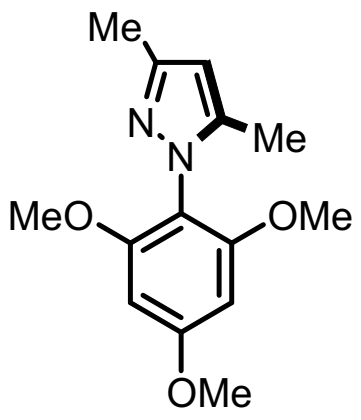

12

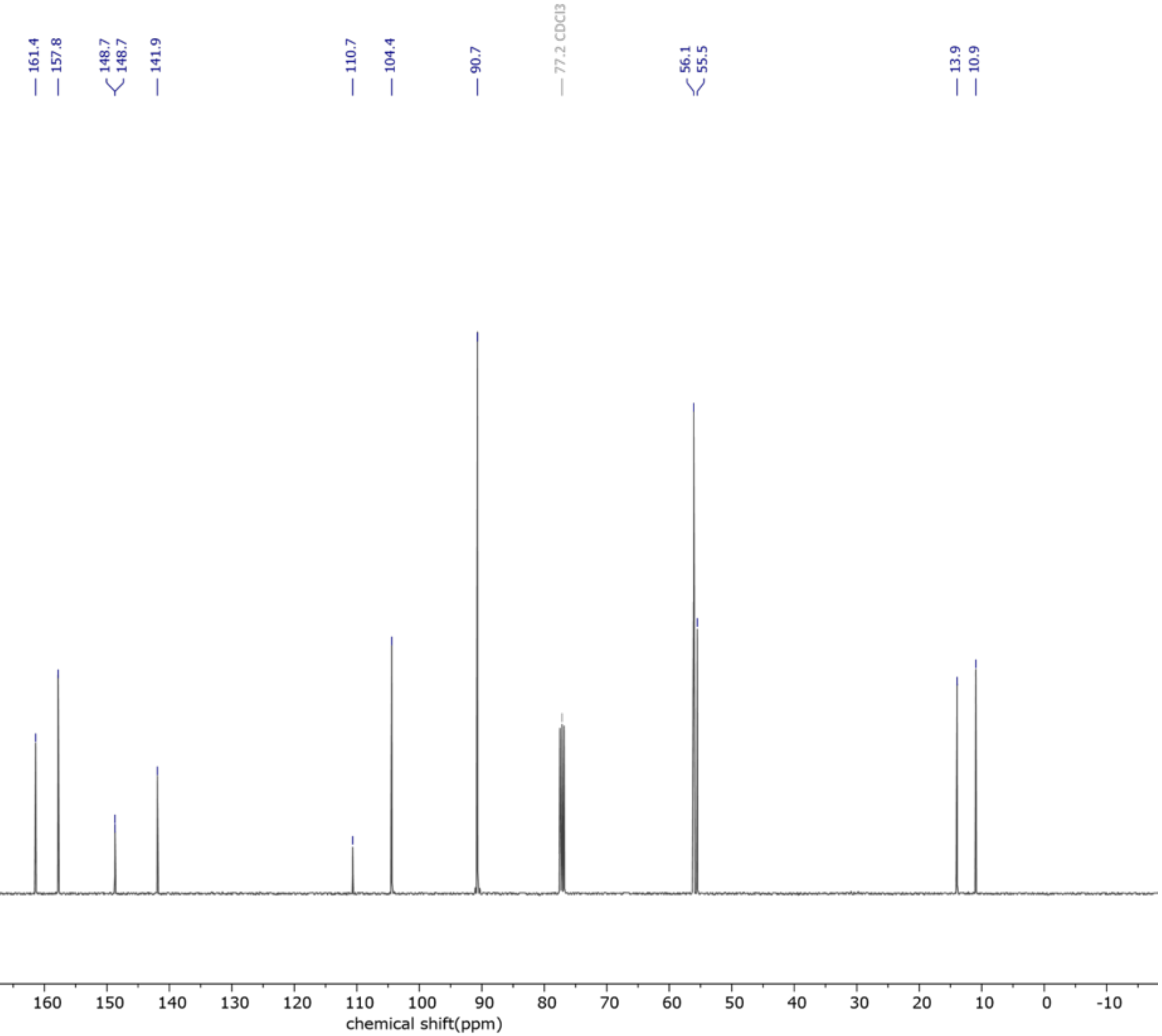

^{1}H NMR OF 1-METHOXY-4-((1*S*,2*R*,4*S*)-2-METHYL-4-PHENYLCYCLOBUTYL)BENZENE (14)

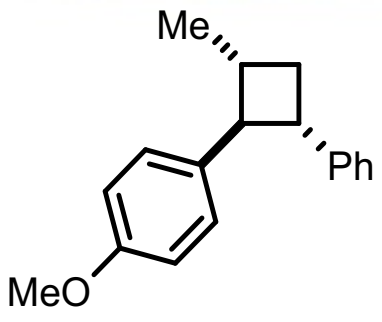

14

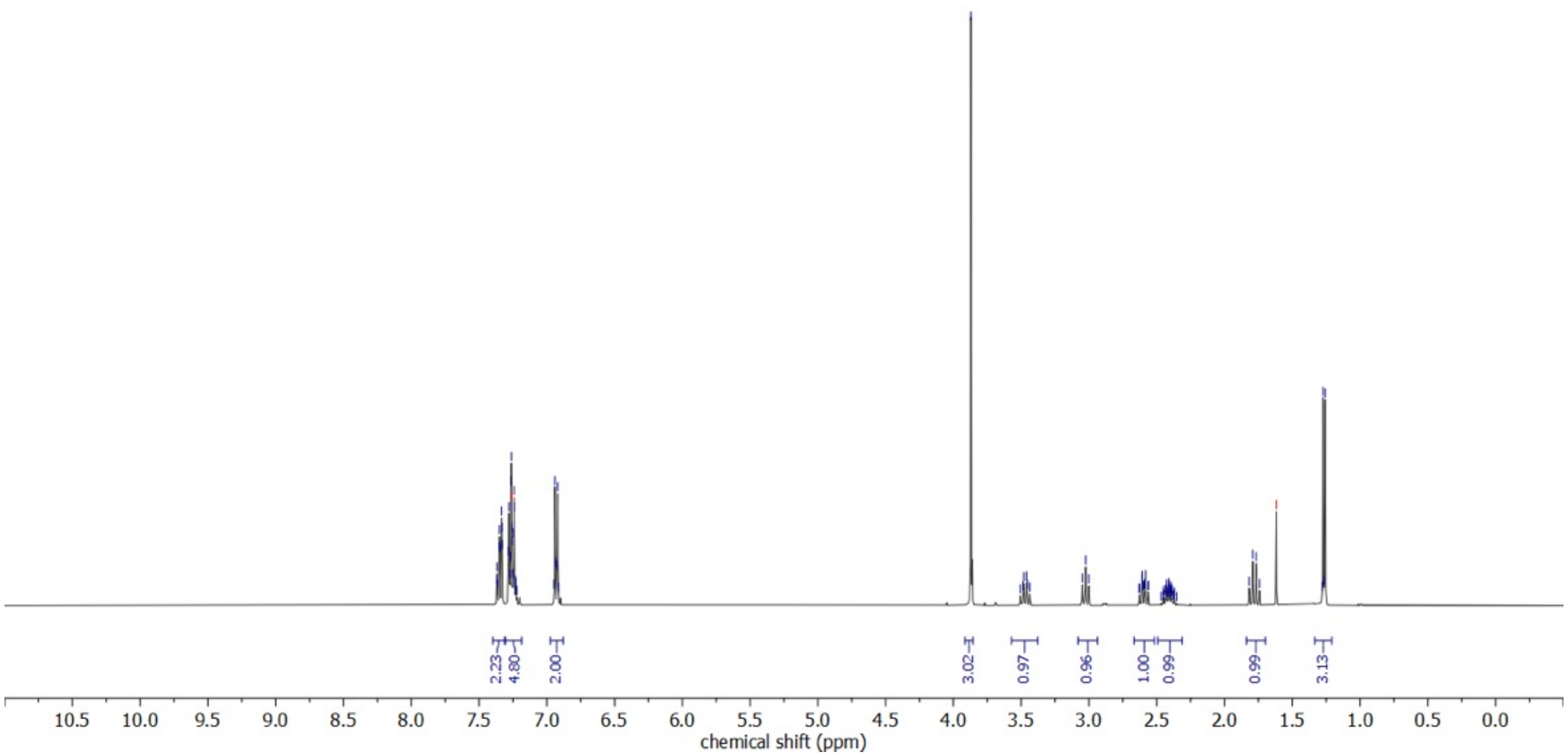

^{13}C NMR OF 1-METHOXY-4-((1*S*,2*R*,4*S*)-2-METHYL-4-PHENYLCYCLOBUTYL)BENZENE (14)

CDCl$_3$, 23 °C

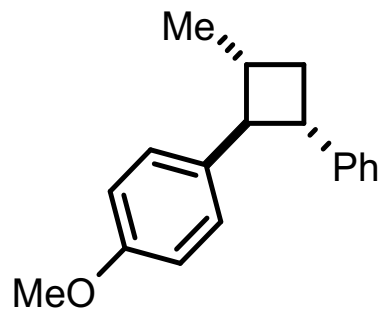

14

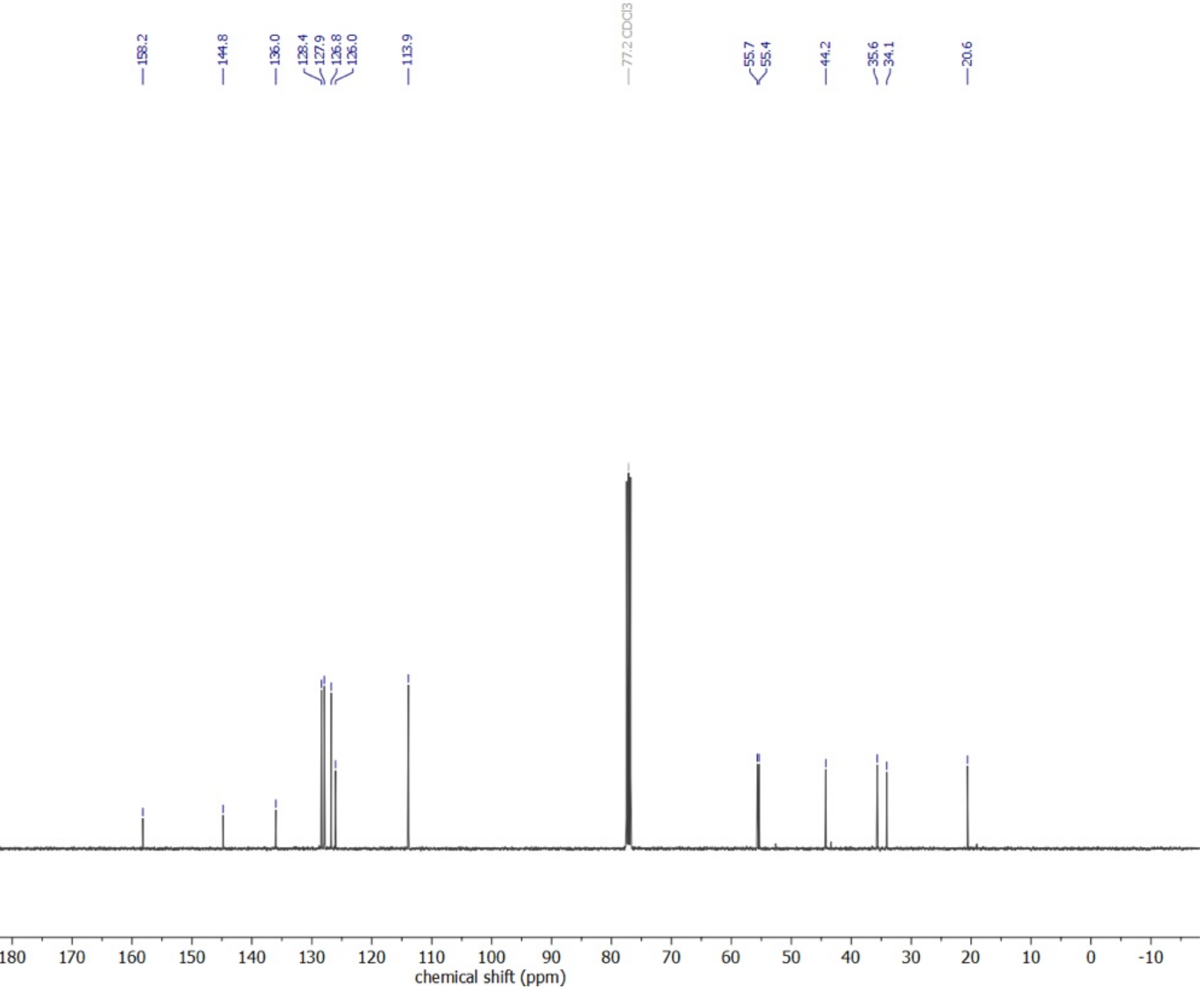

^{1}H NMR OF 4-(*TERT*-BUTYL)PHENOL (16)

CDCl3, 23 °C

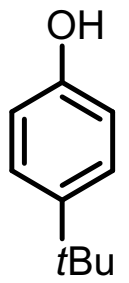

16

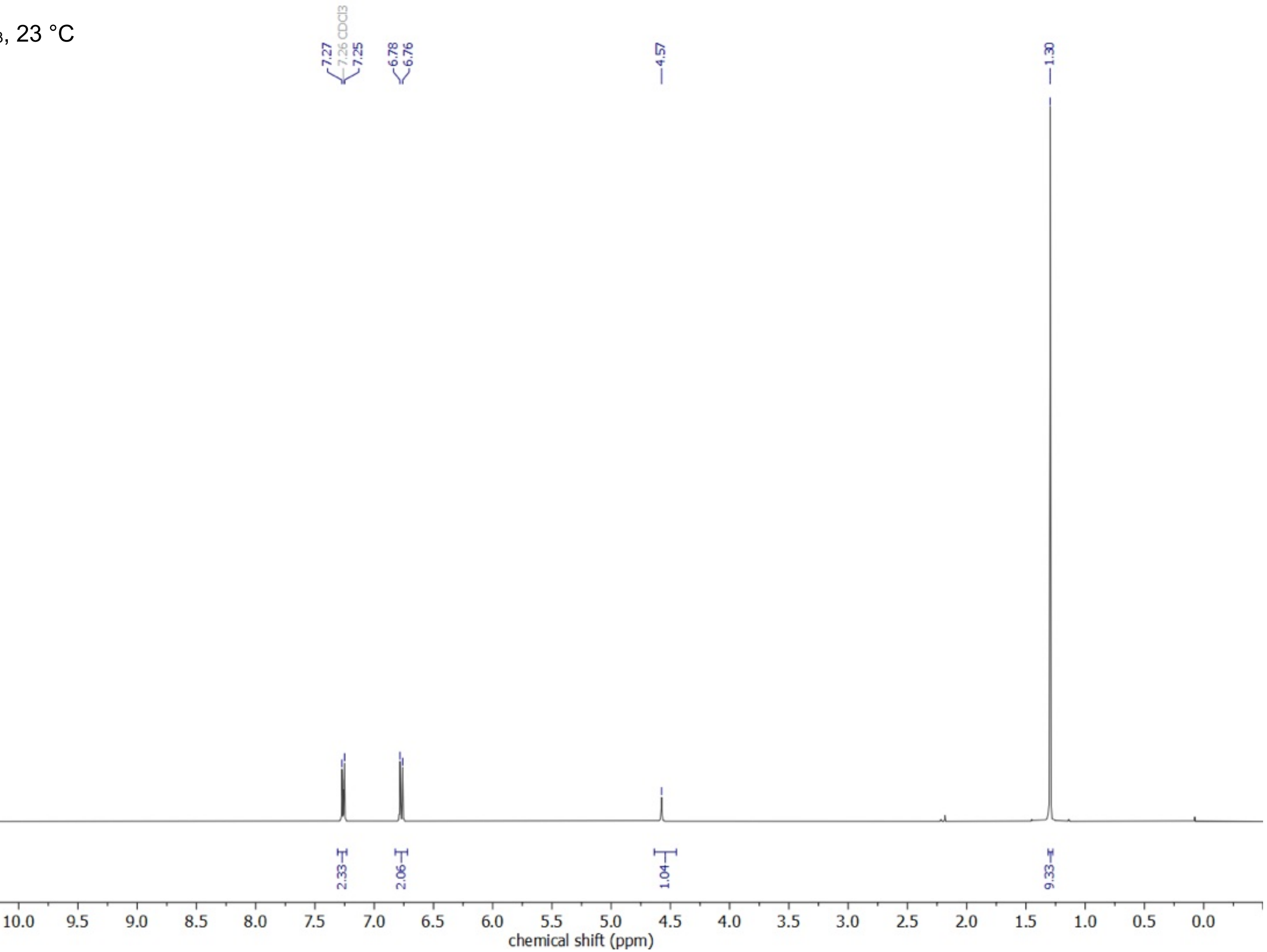

^{13}C NMR of 4-(*tert*-butyl)phenol (16)

CDCl₃, 23 °C

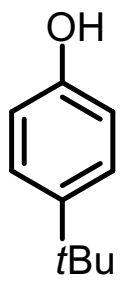

16

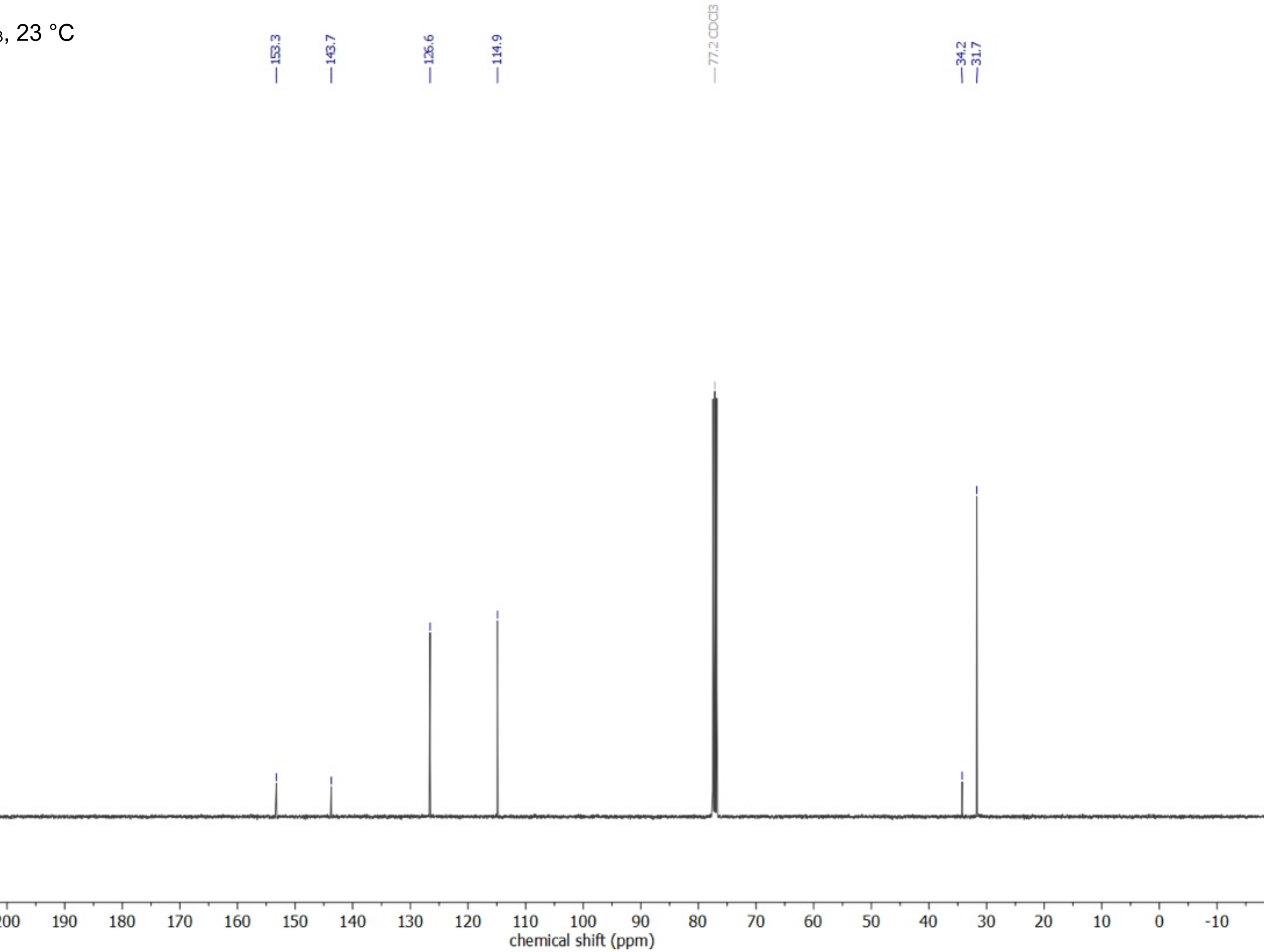

^{1}H NMR OF 1-(NITROMETHYL)-2-PHENYL-1,2,3,4-TETRAHYDROISOQUINOLINE (18)

CDCl3, 23 °C

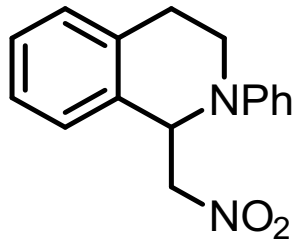

18

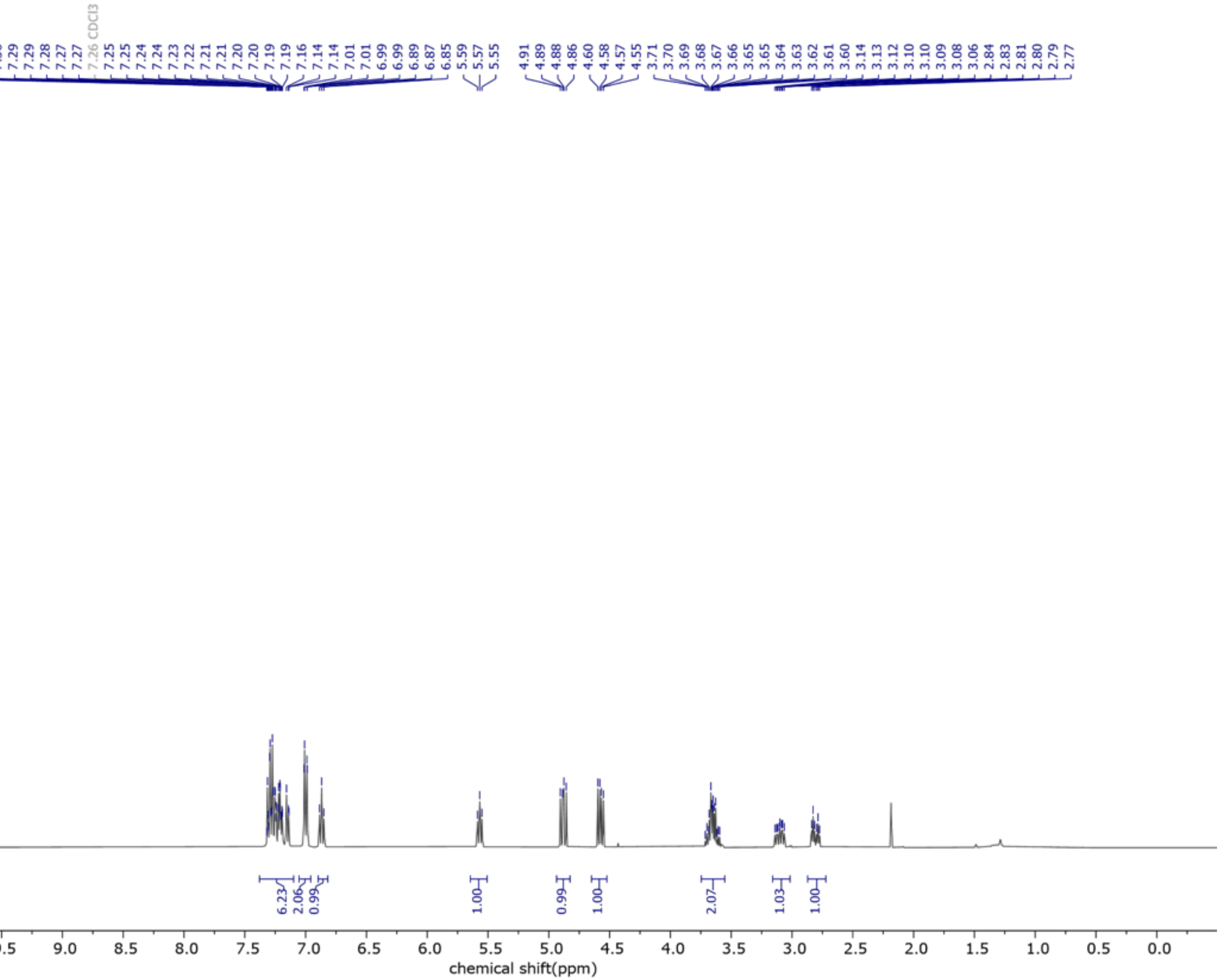

^{13}C NMR of 1-(nitromethyl)-2-phenyl-1,2,3,4-tetrahydroisoquinoline (18)

$CDCl_3$, 23 °C

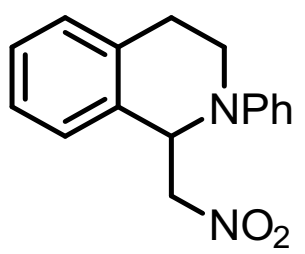

18

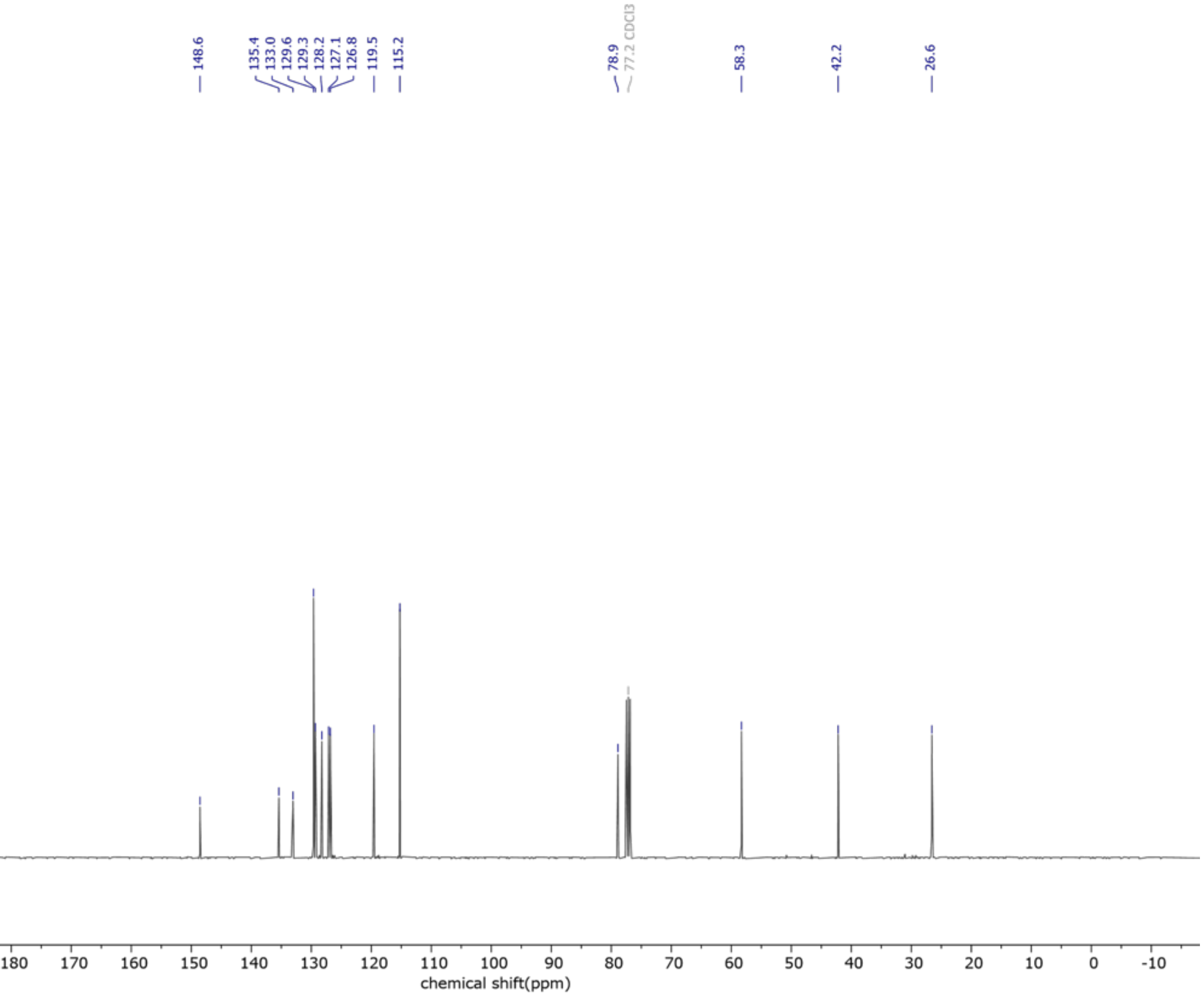

^{1}H NMR OF 4-(*TERT*-BUTYL)-2-IODO-1-METHYLBENZENE (S2)

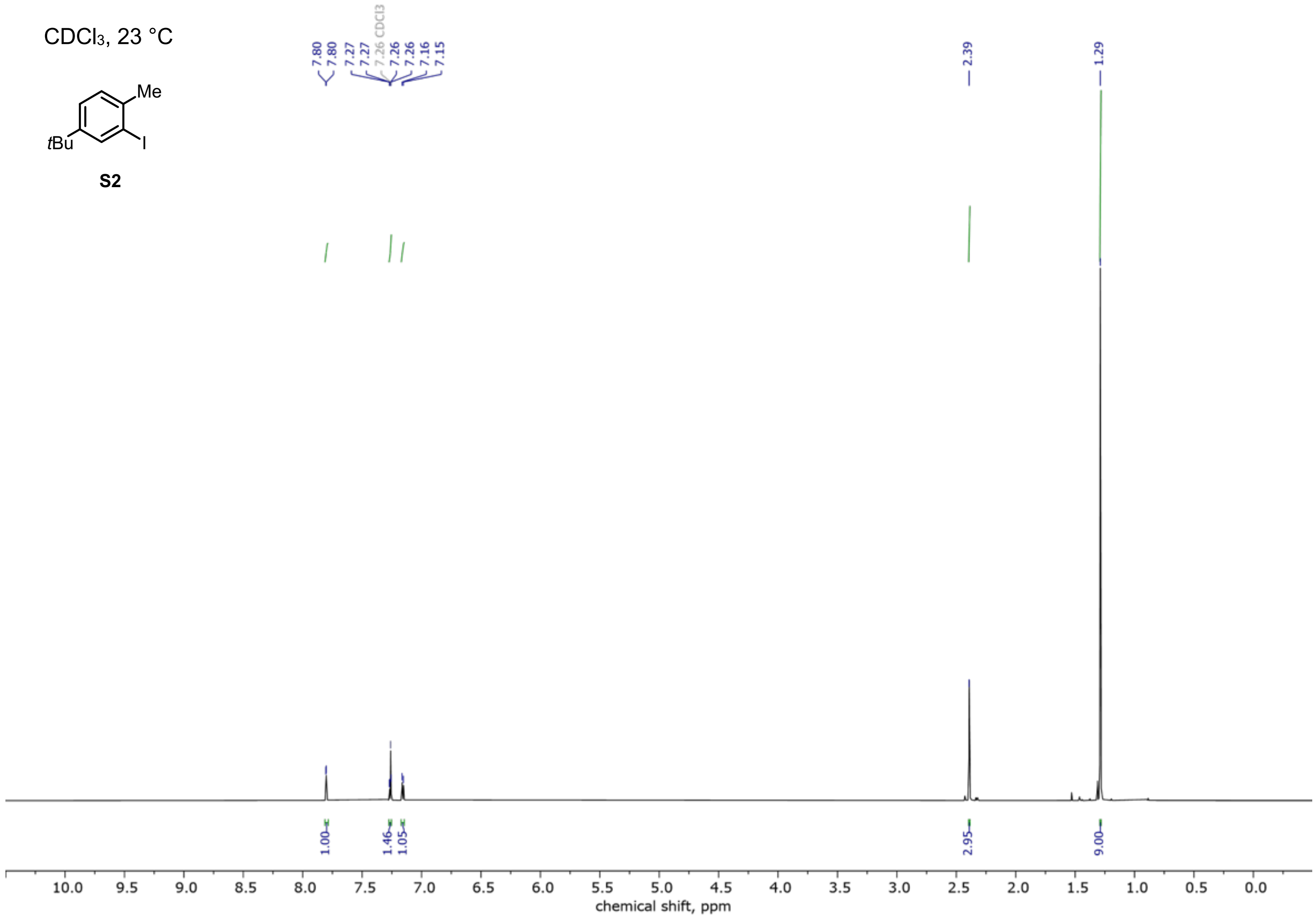

^{13}C NMR OF 4-(*TERT*-BUTYL)-2-IODO-1-METHYLBENZENE (S2)

CDCl$_3$, 23 °C

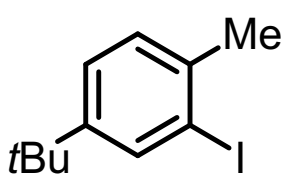

S2

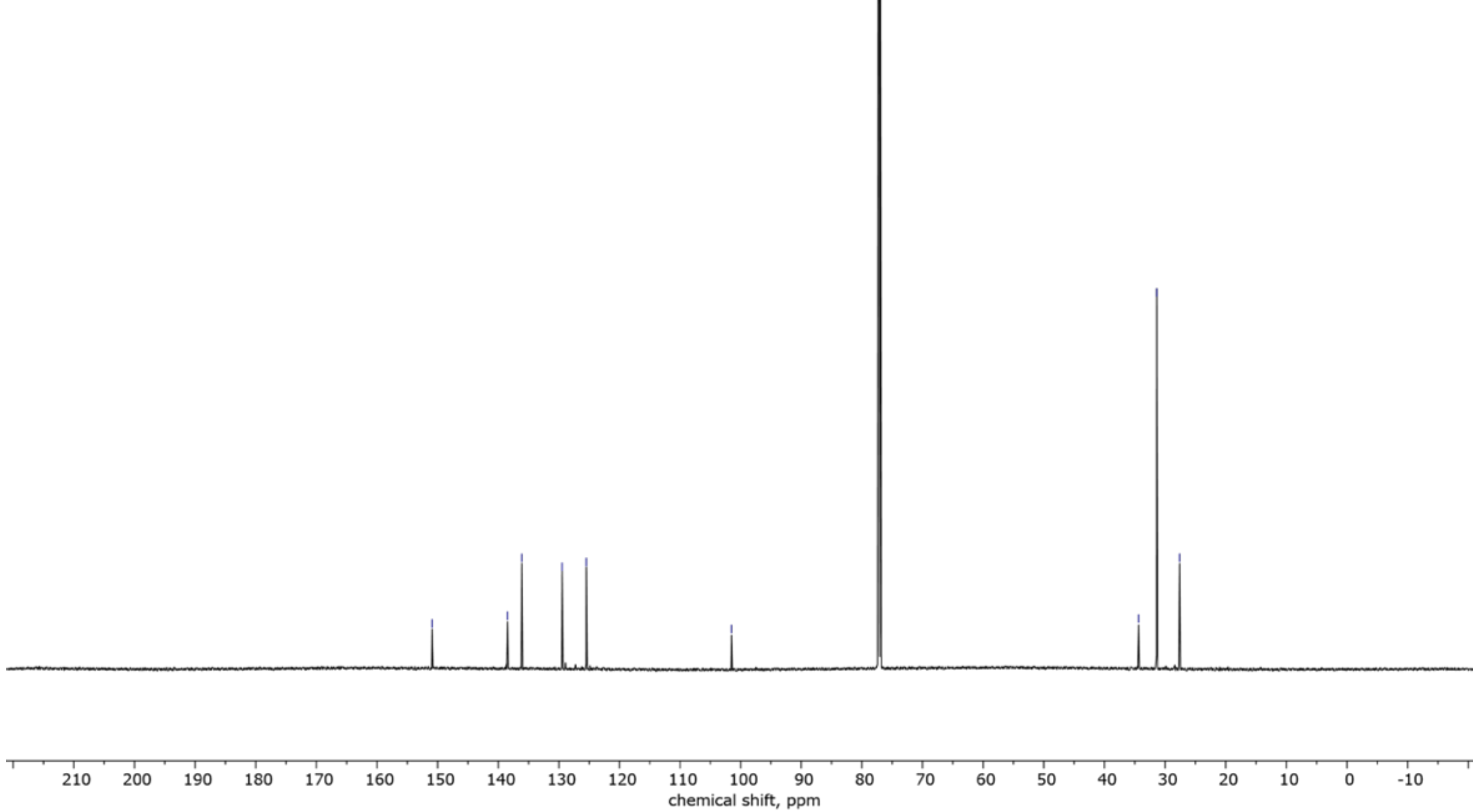

^{1}H NMR OF METHYL 4-(*TERT*-BUTYL)-2-IODOBENZOATE (S4)

CDCl3, 23 °C

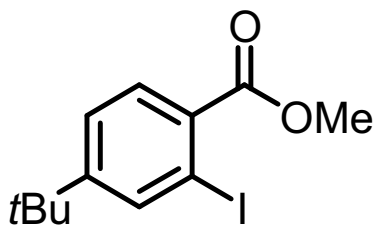

S4

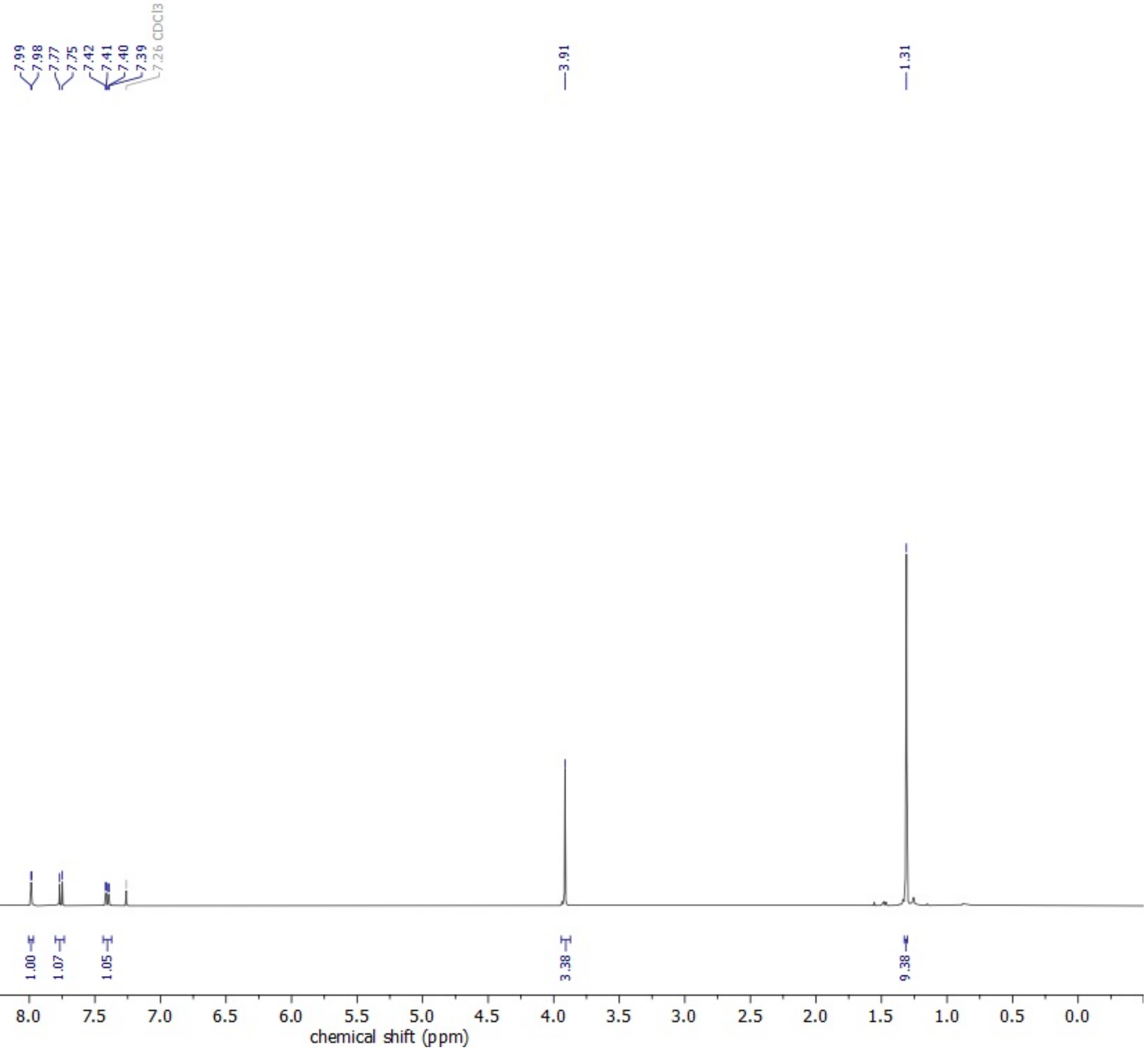

^{13}C NMR OF METHYL 4-(*TERT*-BUTYL)-2-IODOBENZOATE (S4)

CDCl$_3$, 23 °C

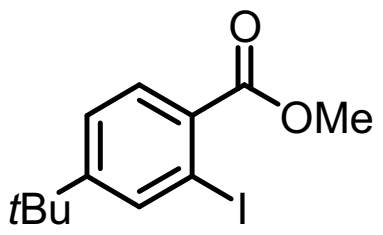

S4

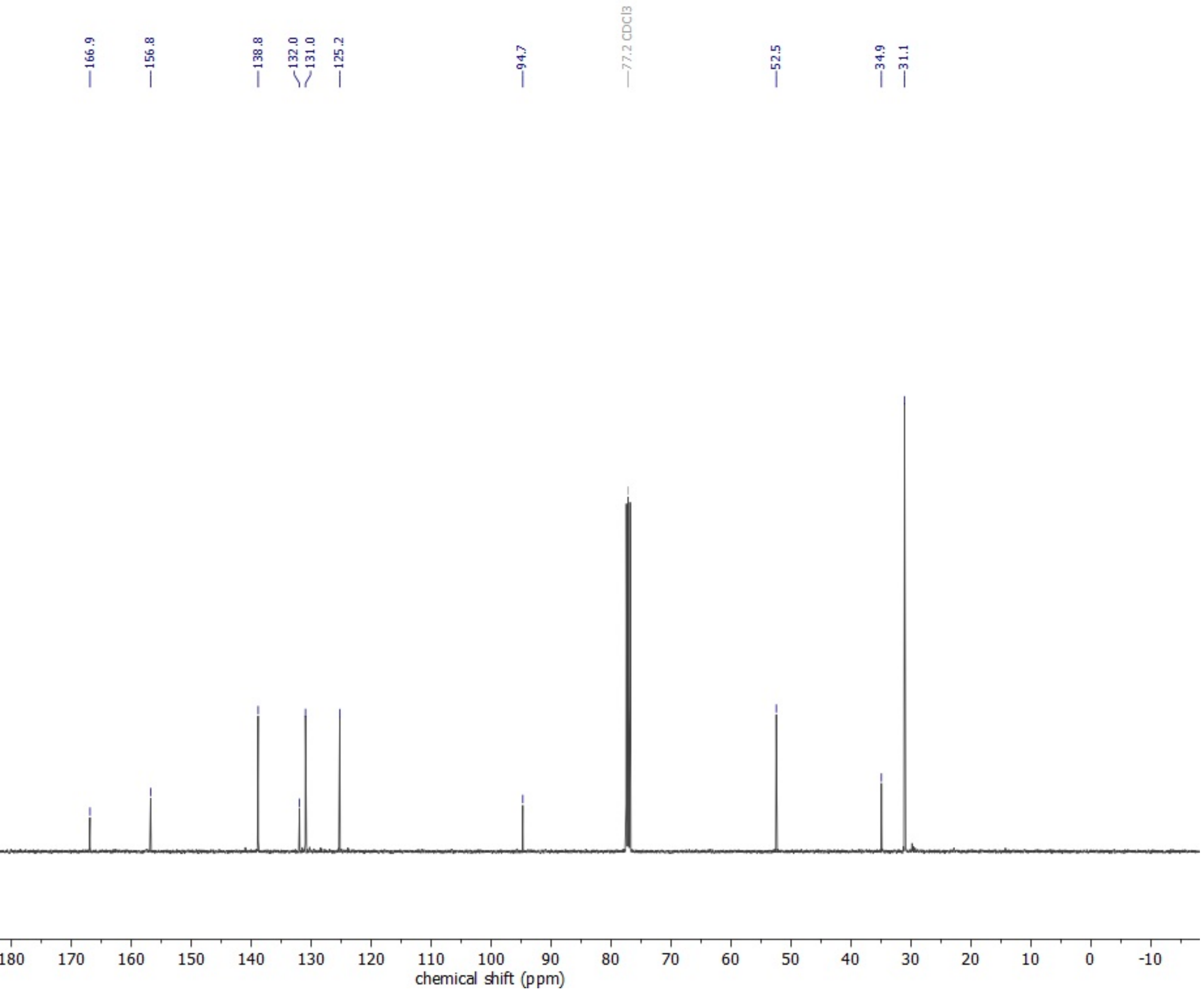

^{1}H NMR OF METHYL 4-(*TERT*-BUTYL)-2-((3-(*TERT*-BUTYL)PHENYL)THIO)BENZOATE (S5)

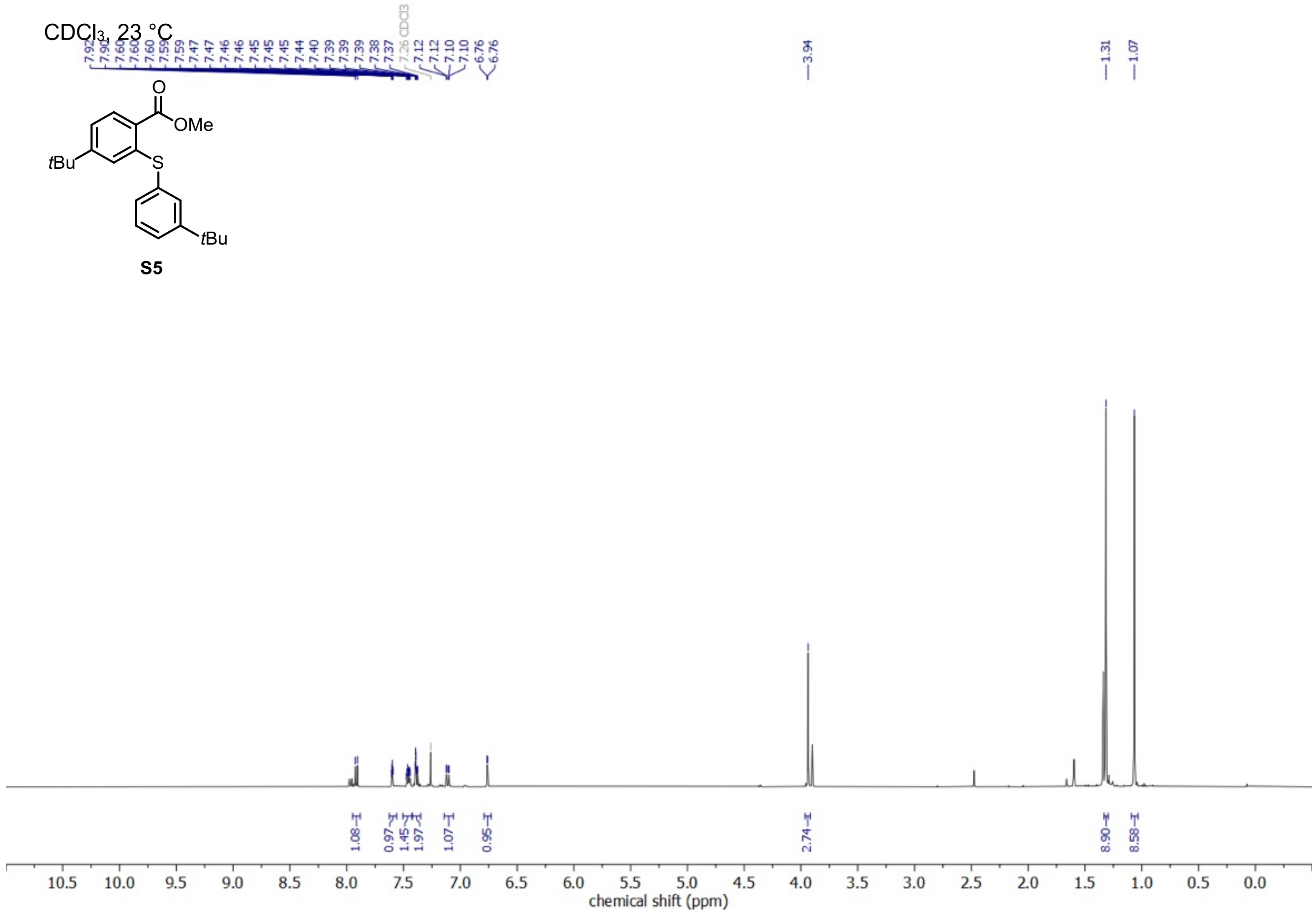

^{13}C NMR OF METHYL 4-(*TERT*-BUTYL)-2-((3-(*TERT*-BUTYL)PHENYL)THIO)BENZOATE (S5)

$CDCl_3$, 23 °C

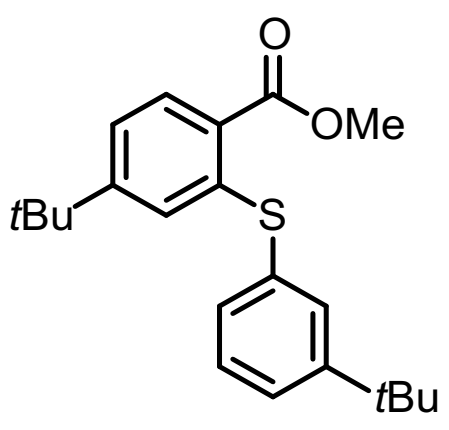

S5

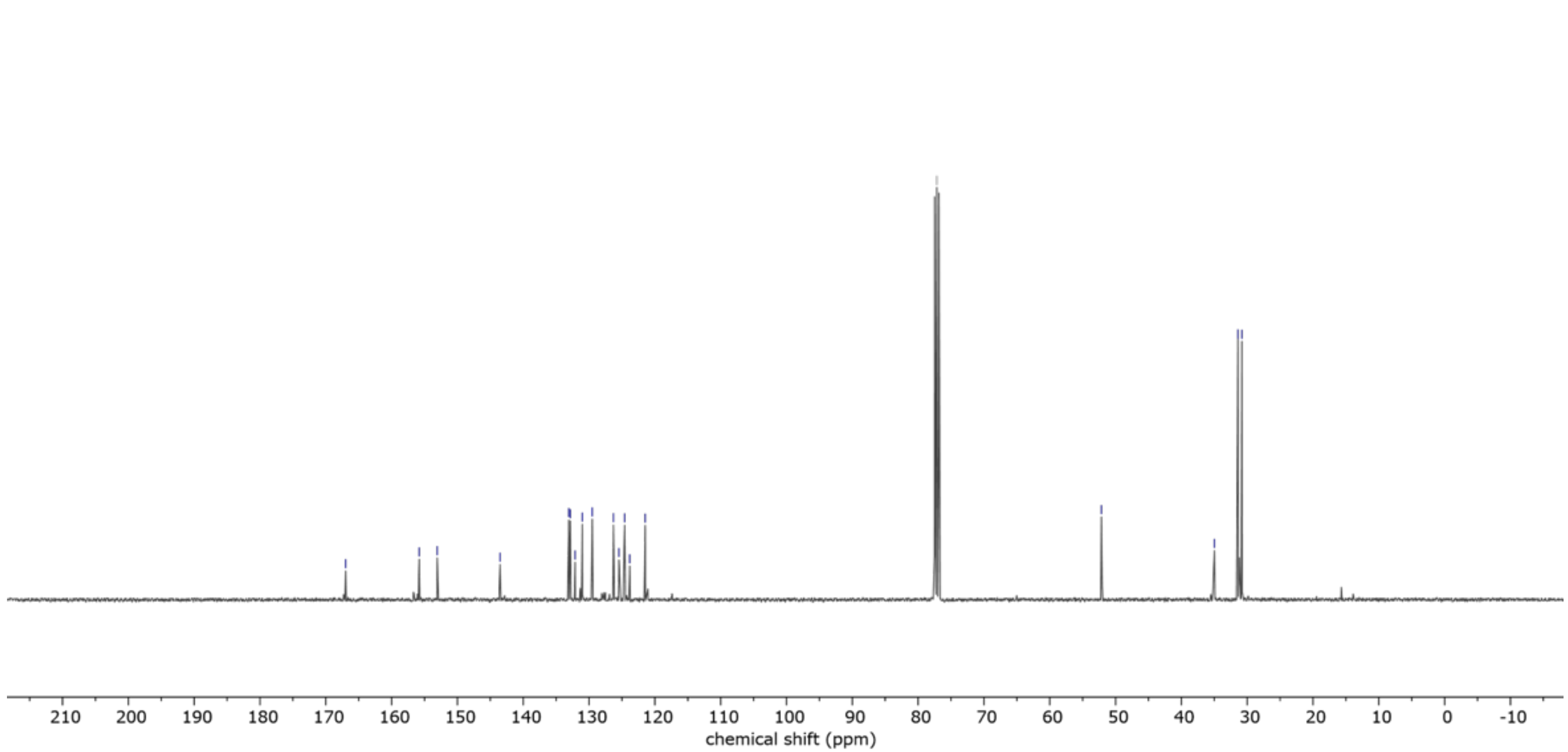

^{1}H NMR OF 3,6-DI-*TERT*-BUTYL-THIOXANTHEN-9-ONE (S6)

CDCl3, 23 °C

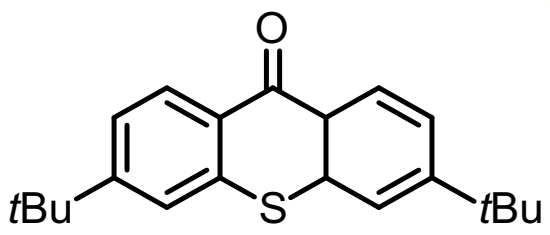

S6

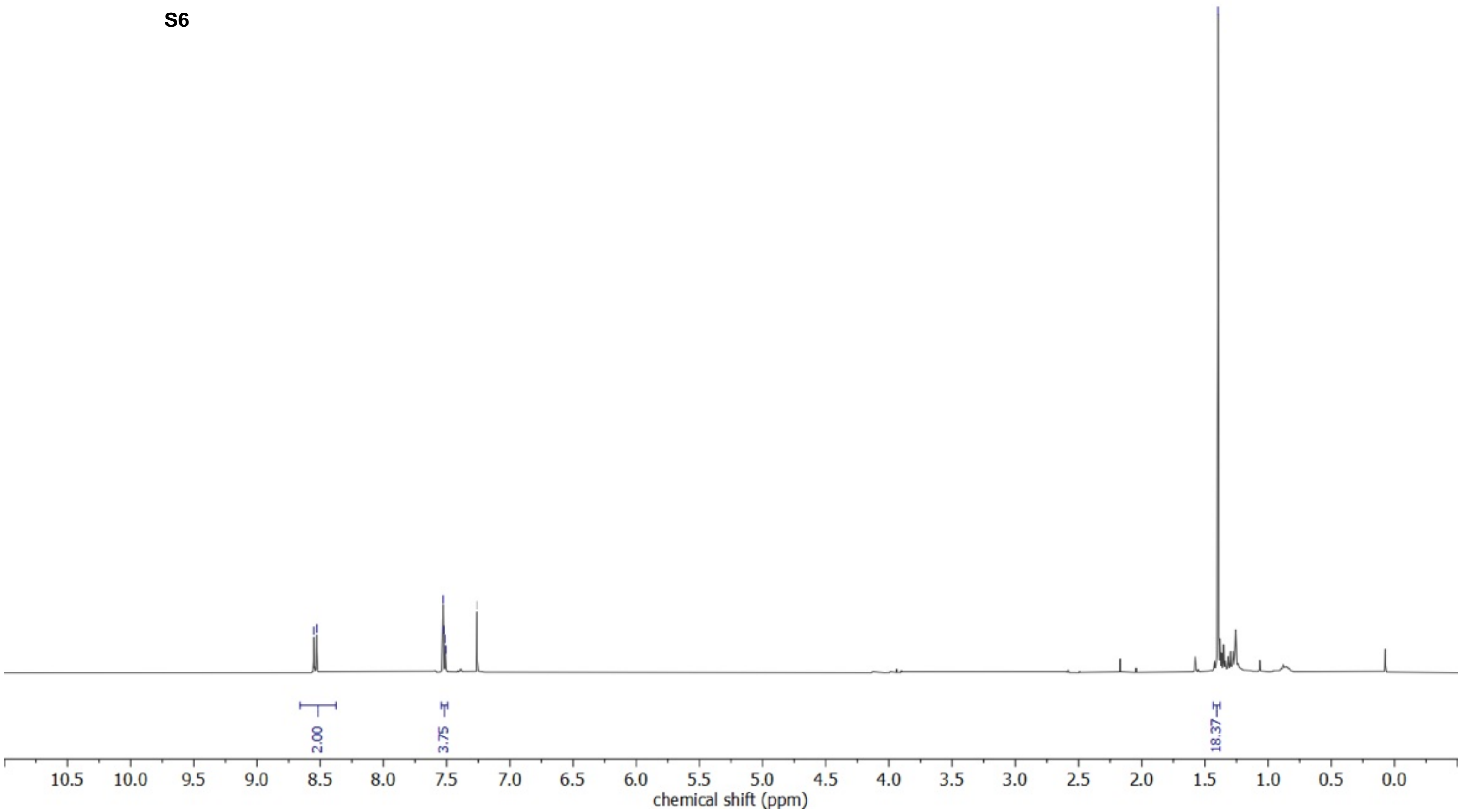

^{13}C NMR OF 3,6-DI-*TERT*-BUTYL-THIOXANTHEN-9-ONE (S6)

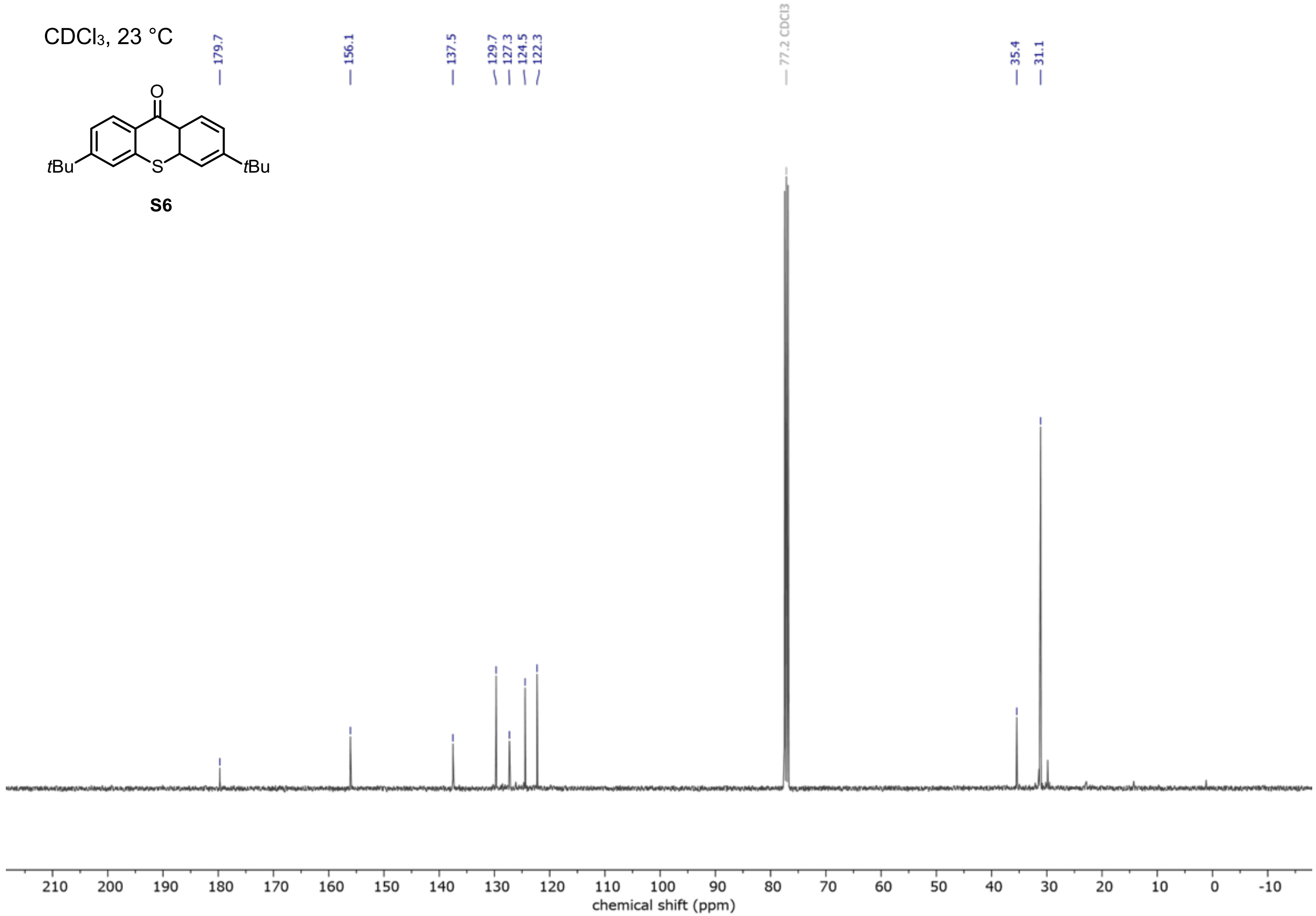

^{1}H NMR OF METHYL 4-(*TERT*-BUTYL)-2-((3-(*TERT*-BUTYL)PHENYL)SELANYL)BENZOATE (S8)

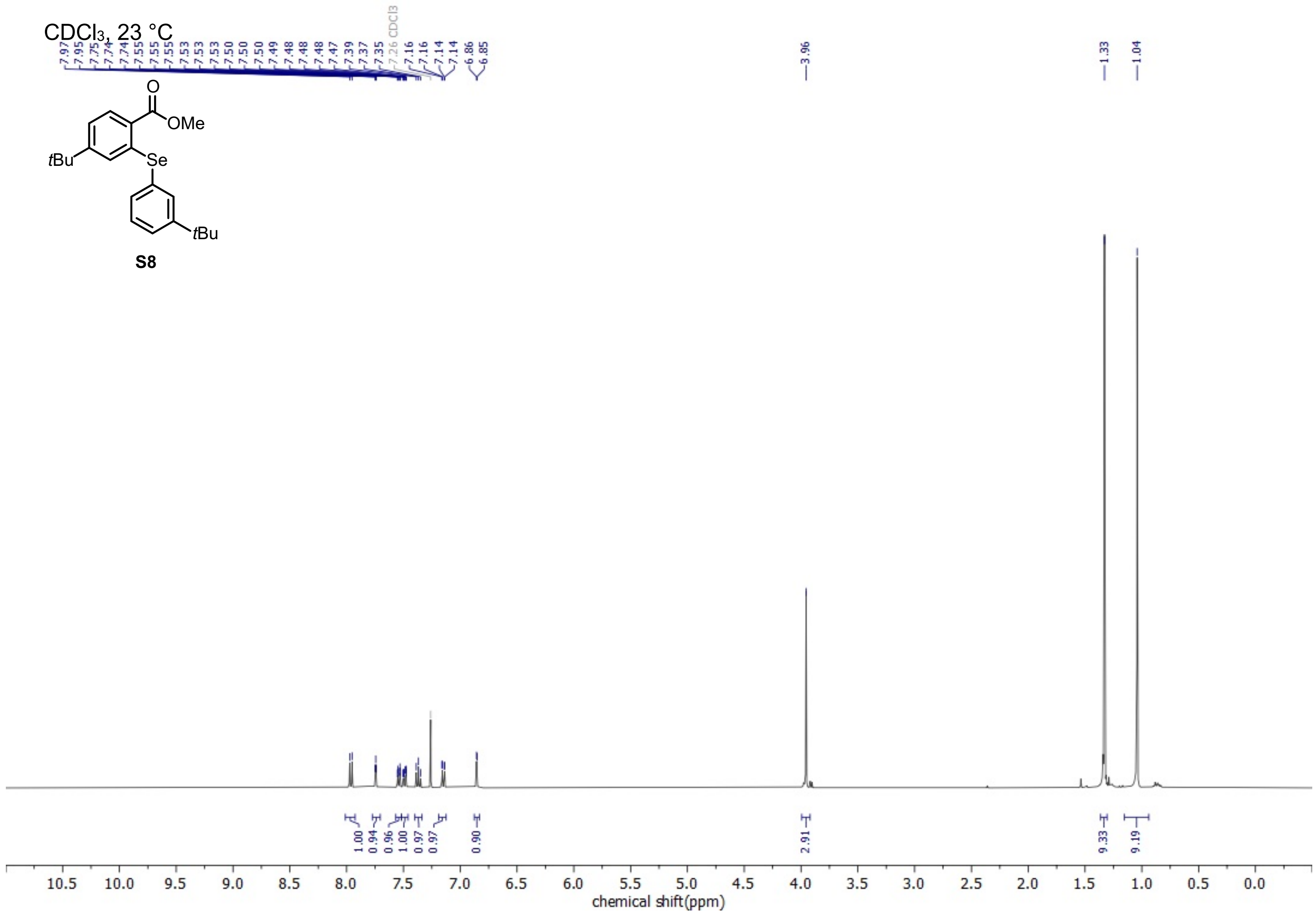

^{13}C NMR OF METHYL 4-(*TERT*-BUTYL)-2-((3-(*TERT*-BUTYL)PHENYL)SELANYL)BENZOATE (S8)

CDCl$_3$, 23 °C

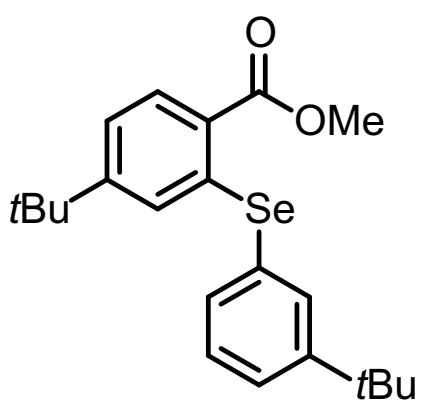

S8

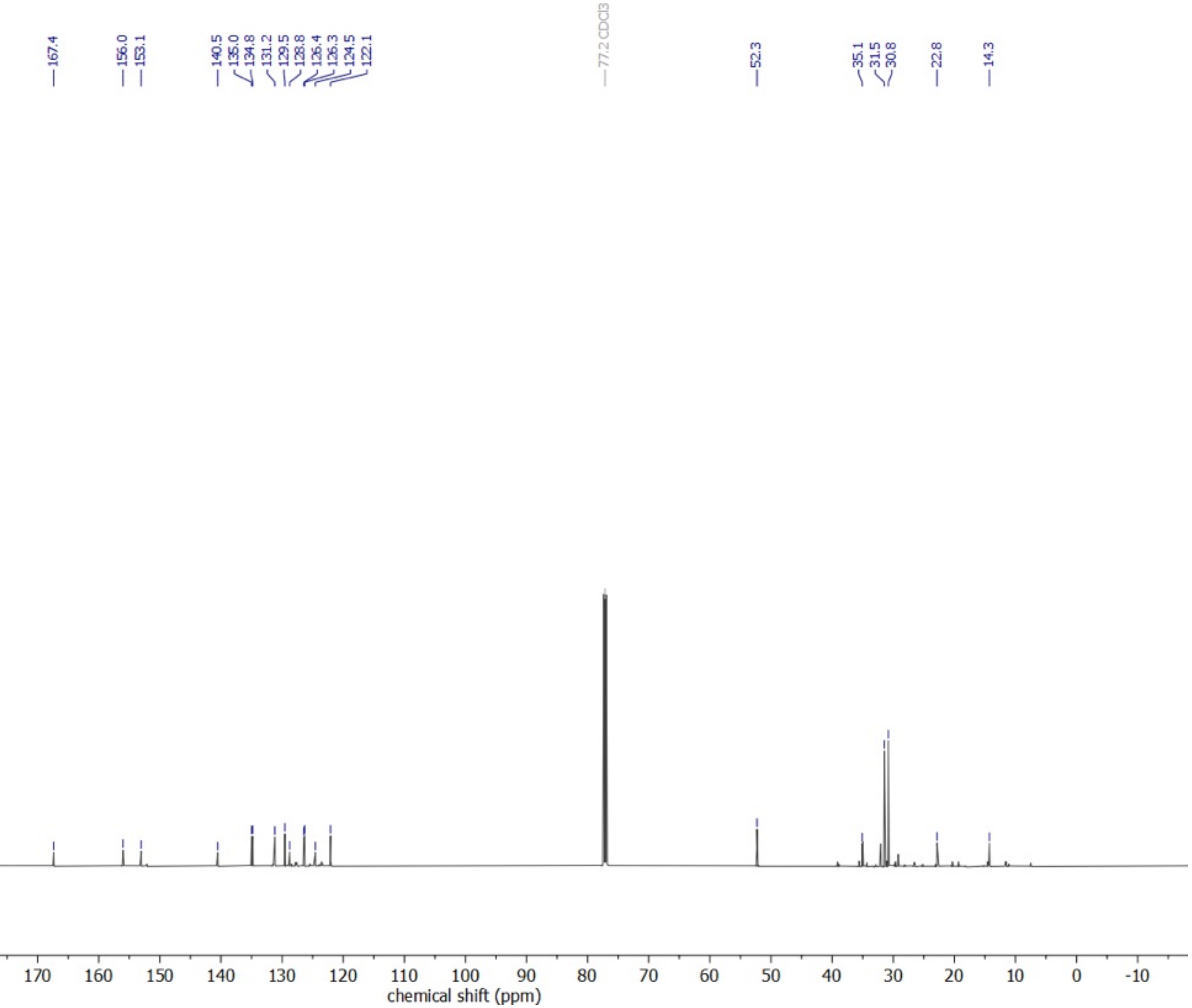

^{77}Se NMR of methyl 4-(*tert*-butyl)-2-((3-(*tert*-butyl)phenyl)selanyl)benzoate (S8)

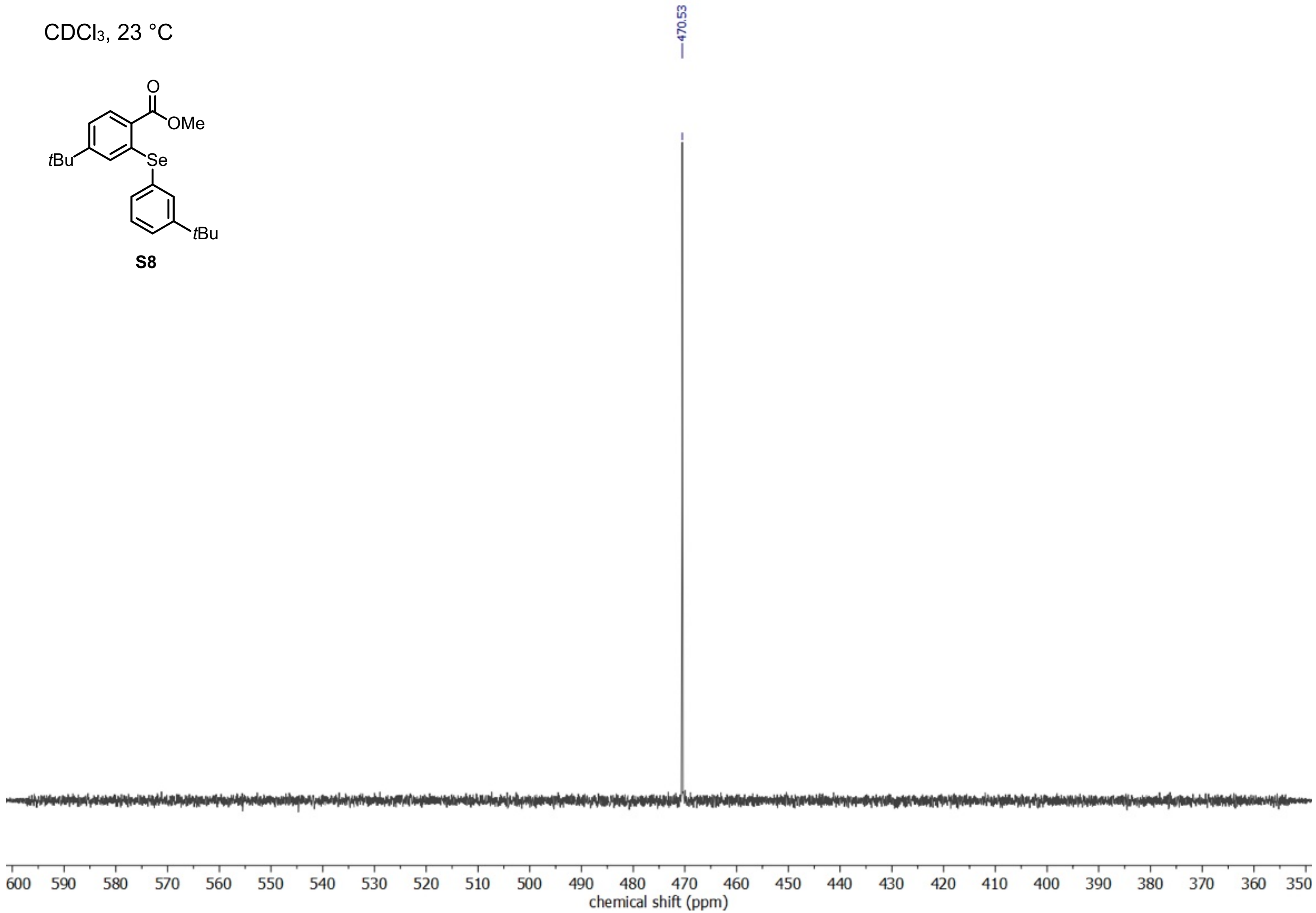

References

[1] R. K. Harris, E. D. Becker, S. M. Cabral De Menezes, P. Granger, R. E. Hoffman, K. W. Zilm, *Pure and Applied Chemistry* **2008**, *80*, 59–84.
[2] G. M. Sheldrick, *Acta Crystallogr A Found Adv* **2015**, *71*, 3–8.
[3] G. M. Sheldrick, *Acta Crystallogr C Struct Chem* **2015**, *71*, 3–8.
[4] O. V. Dolomanov, L. J. Bourhis, R. J. Gildea, J. A. K. Howard, H. Puschmann, *J Appl Crystallogr* **2009**, *42*, 339–341.
[5] C. B. Hübschle, G. M. Sheldrick, B. Dittrich, *J Appl Crystallogr* **2011**, *44*, 1281–1284.
[6] V. V. Pavlishchuk, A. W. Addison, *Inorg. Chim. Acta* **2000**, *298*, 97–102.
[7] F. Neese, *WIREs Comput Mol Sci* **2025**, *15*: e70019.
[8] F. Weigend, R. Ahlrichs, *Phys. Chem. Chem. Phys.* **2005**, *7*, 3297-3305.
[9] E. Caldeweyher, J.M. Mewes, S. Ehlert, S. Grimme, *Phys. Chem. Chem. Phys.* **2020**, *22*, 8499-8512.
[10] P. Pollak, F Weigend, *J. Chem. Theory Comput.* **2017**, *13*, 8, 3696–3705.
[11] W. Kutzelnigg, W.Liu, *J. Chem. Phys.* **2005**, *123*, 241102.
[12] National research council, Ed., *Prudent Practices in the Laboratory: Handling and Management of Chemical Hazards*, National Academies Press, Washington (D.C.), **2011**.
[13] B. D. Ravetz, N. E. S. Tay, C. L. Joe, M. Sezen-Edmonds, M. A. Schmidt, Y. Tan, J. M. Janey, M. D. Eastgate, T. Rovis, ACS Cent. Sci. **2020**, *6*, 2053–2059.
[14] Y. Yu, Y. Fu, F. Zhong, *Green Chem.,* **2018***, 20,* 1743-1747.
[15] P. Luo, E. C. Feinberg, G. Guirado, S. Farid, J. P. Dinnocenzo, *J. Org. Chem.*, **2014**, *79*, 19, 9297–9304.
[16] A. Casado-Sanchez, M. Uygur, D. Gonzalez-Munoz, F. Aguilar-Galindo, J. L. Nova-Fernandez, J. Arranz-Plaza, S. Diaz-Tendero, S. Cabrera, O. G. Mancheno, J. Aleman, *J. Org. Chem.* **2019***, 84,* 10, 6437–6447.
[17] V. A. Pistritto, M. E. Schutzbach-Horton, D. A. Nicewicz, *J. Am. Chem. Soc.* **2020**, 142*, 40,* 17187–17194.
[18] K. Ohkubo, H. Kotani, S. Fukuzumi, *Chem. Commun.,* **2005***, 36,* 4520-4522.
[19] A. Yoshimura, M. Z. Hoffman, H. Sun, *J. Photochem. Photobiol. A: Chem.,* **1993***, 70,* 29-33.
[20] S. G. Hadley, R. A. Keller, *J. Phys. Chem.*, **1969**, *73*, 4351–4355.
